# Application of continuous renal replacement therapy (CRRT) in patients with severe acute pancreatitis: an analytical study

**DOI:** 10.1186/s12876-025-04198-y

**Published:** 2025-08-18

**Authors:** Yiru Weng, Zhouzhou Dong, Gongjie Ye, Linhui Shi, Panpan Liu, Tingting Zhou, Honghua Ye

**Affiliations:** The Affiliated LiHuiLi Hospital of Ningbo University, Ningbo, 315040 Zhejiang P. R. China

**Keywords:** Severe acute pancreatitis, Continuous renal replacement therapy, Efficacy, Nomogram model

## Abstract

**Purpose:**

To Explore the correlation between Continuous Renal Replacement Therapy (CRRT) and the prognostic outcomes of patients with Severe Acute Pancreatitis (SAP); to analyze the impact of CRRT initiation time on the final prognosis of SAP patients; to evaluate factors affecting the therapeutic effect of CRRT in SAP patients without absolute indications for CRRT, and to develop a multi-factorial predictive model for the efficacy of CRRT in treating SAP.

**Methods:**

This retrospective cohort study analyzed clinical data from SAP patients admitted to The Affiliated LiHuili Hospital of Ningbo University (2015–2024), collecting baseline characteristics (demographics, disease severity scores), CRRT parameters (initiation timing, laboratory values), and clinical outcomes. We stratified patients by prognosis and CRRT status, and performed subgroup analyses for CRRT-treated cases. After excluding patients with definitive CRRT indications, we randomly allocated cases to training (80%) and validation (20%) sets. Using logistic regression, we identified CRRT failure predictors, evaluated their predictive value via ROC analysis, and developed/validated a nomogram prediction model.

**Results:**

Among 563 initially screened SAP patients, 282 were included after exclusions. (1) Prognosis analysis revealed significant differences between improved and poor outcome groups in age, pancreatitis type, CRRT use, APACHE II and Marshall scores (all *P* < 0.05). Multivariate analysis identified CRRT as an independent protective factor and Marshall score as a risk factor. Compared to non-CRRT patients, CRRT-treated patients showed significantly shorter hospitalization and vasopressor duration (*P* < 0.05), with comparable costs. (2) In CRRT-treated patients (with/without absolute indications), earlier CRRT initiation (< 36 h) correlated with better outcomes (*P* < 0.05). Multivariate analysis confirmed CRRT initiation time as an independent prognostic factor (optimal cutoff: 36 h). (3) Among 114 CRRT-treated patients without absolute indications (91 in training set), significant differences existed in pancreatitis type, APACHE II, Marshall score, lactate, calcium, albumin, PT and PCT (*P* < 0.05). Multivariate analysis identified APACHE II, PCT and lactate as independent risk factors, and calcium/albumin as protective factors for CRRT failure. The combined model showed excellent predictive value (AUC = 0.912, 95%CI:0.841–0.982). The nomogram demonstrated good calibration in both training and test sets.

**Conclusions:**

CRRT serves as an independent protective factor against poor outcomes (discharge against medical advice (death within 24 h post-discharge (against medical advice)) /death) in SAP patients, while simultaneously reducing hospitalization duration and vasopressor requirements without increasing financial burden. Early application (within 36 h) demonstrates greater therapeutic benefit. The developed nomogram prediction model, incorporating key prognostic factors, exhibits excellent clinical applicability and provides an objective basis for evaluating treatment timing in patients without strong CRRT indications.

**Supplementary Information:**

The online version contains supplementary material available at 10.1186/s12876-025-04198-y.

## Introduction

Acute pancreatitis (AP), a potentially fatal inflammatory disorder of the pancreas, results from premature activation of digestive enzymes causing autodigestion [1]. Epidemiological studies report an incidence of 13–45 cases per 100,000 annually, with approximately 20% progressing to SAP characterized by persistent organ failure (> 48 h)] [2–5]. The transition from AP to SAP involves complex pathophysiological mechanisms including sustained inflammatory responses and multiorgan dysfunction [6]. Disease severity was assessed using both APACHE II [7] and Marshall scoring systems [8]. CRRT has emerged as a potential therapeutic modality, offering benefits beyond renal support through its capacity for cytokine removal and immunomodulation [9–11]. While preclinical evidence suggests CRRT may attenuate the systemic inflammatory response in SAP [12], clinical data remain inconclusive regarding its efficacy and optimal timing of initiation [13, 14]. Recent evidence indicates that critically ill patients with severe AKI benefit from accelerated RRT initiation in terms of survival and dialysis-free outcomes, particularly in surgical ICU populations or when receiving CRRT, whereas non-CRRT modalities or high SOFA scores may increase dialysis dependence risk [15]. This knowledge gap underscores the need for comprehensive evaluation of CRRT’s therapeutic value in SAP, particularly examining the relationship between treatment timing and clinical outcomes. The current study employs advanced statistical modeling, combining logistic regression with nomogram analysis, to systematically assess laboratory parameters predictive of CRRT response in SAP patients, thereby providing evidence-based guidance for clinical decision-making.

## Materials and methods

### Study population

#### Study subjects

**Fig. 1:**
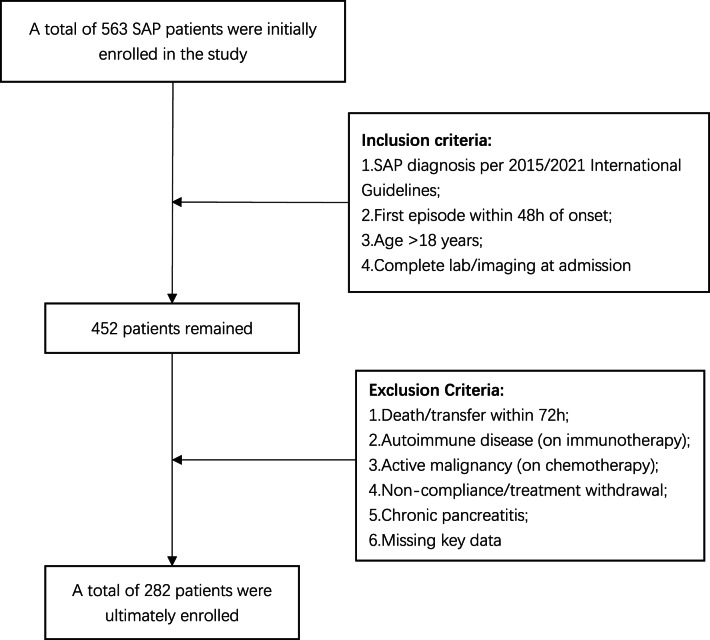
Study flowchart of patient selection criteria

A retrospective cohort study was conducted, selecting 282 patients diagnosed with SAP in our hospital from March 2015 to March 2024 as the study subjects (Fig. 1).

**Inclusion criteria**:

1. Diagnosis of SAP according to the criteria outlined in the International Consensus Guidelines for Acute Pancreatitis, 2015 or International Consensus Guidelines for Acute Pancreatitis,2021;
2. First episode of the disease, with presentation within 48 h of onset;
3. Age > 18 years;
4. Immediate systematic laboratory and imaging examinations upon admission.

**Exclusion criteria**:

1. Patients who died or were transferred within 72 h after admission to the ICU;
2. Patients with autoimmune diseases or immune dysfunction undergoing immunotherapy;
3. Patients with malignant tumors undergoing chemotherapy;
4. Patients who did not cooperate or voluntarily abandoned treatment during the therapeutic process;
5. Chronic pancreatitis;
6. Patients with missing key clinical data in medical records.

### Ethics

We analyzed de-identified data from SAP patients (2015–2024). The data came from The Affiliated LiHuili Hospital of Ningbo University. The study period spanned from 2015 to 2024. We de-identified all patient data prior to analysis. The protocol (Approval No. KY2024SL176-01) was approved by the Institutional Review Board with waiver of informed consent due to its retrospective nature. The study strictly complied with the Declaration of Helsinki (2024 version), China’s Ethical Review Measures for Biomedical Research Involving Humans, and implemented a three-level data protection protocol including complete anonymization of 18 personal identifiers by certified archivists, dual-authentication database access, and analysis conducted on physically isolated hospital servers without data transfer. All medical record data were cross-verified through a dual independent entry system, with two professionally trained researchers separately reviewing all elements in each case record. Any disputed or ambiguous data points were submitted to a three-member data quality control committee consisting of associate chief physicians for final adjudication.

### Clinical data

1. Baseline characteristics:​​ Demographic data (age, gender), pancreatitis etiology (biliary, hyperlipidemic, alcoholic, pregnancy-associated, viral, post-surgical, idiopathic), comorbidities (diabetes, hypertension, cardiovascular diseases, chronic kidney disease, immunosuppressant use), and disease severity scores (APACHE II, Marshall).

2. CRRT Indications Based on KDIGO Guidelines [16] (Chap. 4: Dialysis Interventions for AKI)​ (meeting urgent CRRT criteria: severe electrolyte imbalance [K + > 6.5 or Na + < 120/>160 mmol/L with neurological symptoms], refractory acidosis [pH < 7.1 or HCO3-<10 mmol/L unresponsive to therapy], volume overload [pulmonary edema], uremic symptoms [BUN > 36 or Cr > 707 µmol/L], drug intoxication, severe metabolic alkalosis [pH > 7.55], hypercalcemia [Ca2 + > 3.5 mmol/L], or hyperuricemia [UA > 800 µmol/L]).

CRRT was performed as a continuous extracorporeal blood purification technique, utilizing slow and sustained solute/fluid removal via convection and diffusion mechanisms, with treatment duration adjusted according to clinical requirements.

Precise initiation time (hours post-symptom onset).

​​3. Outcome measures: Discharge status (improved prognosis [discharge with recovery] vs. poor outcome [death during hospitalization or within 24 h post-discharge (including cases discharged against medical advice per Chinese cultural practice where terminally ill patients are brought home)]), hospitalization duration (days), total costs, vasopressor duration (days), ICU stay (days), and mechanical ventilation time (hours).

​​4. Laboratory indices at CRRT initiation:​​.

Blood gas: FiO2 (%), PaO2 (mmHg), PaO2/FiO2, PaCO2 (mmHg), K+ (mmol/L), Na+ (mmol/L), lactate (mmol/L), Ca2+ (mmol/L), BE (mmol/L), PAO2 (mmHg);

Liver function: Albumin (g/L), total bilirubin (µmol/L), ALT/AST (U/L); `.

Renal function: Creatinine (µmol/L), urea (mmol/L), uric acid (µmol/L);

Lipid profile: Total cholesterol (mmol/L), triglycerides (mmol/L);

Pancreatic: Serum/urine amylase (U/L);

Coagulation: PT (s), INR, APTT (s), TT (s), D-dimer (µg/L);

Inflammatory markers: CRP (mg/L), PCT (ng/mL), HBP (ng/mL);

Cardiac biomarkers: TnI/TnT (ng/mL), CK/CK-MB (U/L), BNP/NT-proBNP (pg/mL).

### Main outcome variable

1. Patients were stratified by

Final outcomes (improvement/poor);

CRRT treatment status (CRRT vs. non-CRRT groups).

2. CRRT-specific analysis included

CRRT treatment status (CRRT vs. non-CRRT groups).

3. For patients without strong CRRT indications

Comparative analysis of baseline characteristics (demographics, pancreatitis type, comorbidities, APACHE II/Marshall scores);

Laboratory parameters at CRRT initiation (blood gas, hepatic/renal function, lipids, amylase, coagulation, inflammatory/cardiac markers);

Multivariate analysis to identify prognostic factors.

### Statistical analysis

We performed all statistical analyses using SPSS 22.0 (IBM Corp.) and R 4.0.0 (R Foundation). Continuous variables were evaluated for normality using the Shapiro-Wilk test (*P* > 0.10 threshold). Normally distributed variables are presented as mean ± standard deviation and compared using independent Student’s t-tests. Non-normally distributed variables are reported as median (interquartile range) with Mann-Whitney U tests for group comparisons. Categorical variables are expressed as frequencies (%) and analyzed using χ² tests or Fisher’s exact tests for small cell counts (expected frequency < 5), with odds ratios and 95% confidence intervals reported.

For missing data handling, we implemented complete case analysis, including only observations with complete data for all variables in each analysis. This approach was chosen after confirming the random nature of missingness through preliminary evaluation.

In prognostic modeling, we developed binary logistic regression models incorporating variables with *p* < 0.10 in univariate analysis, using backward stepwise selection with a retention criterion of *P* < 0.05. Multicollinearity was assessed through variance inflation factors (all VIF < 3). Model performance was evaluated through ROC curve analysis with 1000 bootstrap replicates for calibration curve assessment. The final predictive nomogram was developed using the ‘rms’ package in R, scaling regression coefficients to a 0-100 point system for clinical utility. All tests were two-tailed with statistical significance defined as *p* < 0.05, and effect sizes including mean differences, odds ratios, and AUC values are reported with 95% confidence intervals throughout the manuscript.

## Results

A comparison of baseline characteristics between the poor outcome and improved prognosis groups of all enrolled demonstrated statistically significant differences in age, type of pancreatitis, administration of CRRT, APACHE II score, and Marshall score. These findings suggest that these factors may potentially influence poor outcome. However, since this analysis did not control for confounding variables, we performed further multivariate regression analysis to adjust for these confounders and identify independent prognostic factors. The details are presented in Table 1.

**Table 1:** Comparison of baseline characteristics between the poor outcome and improved outcome groups

	Improvement (*n* = 180)	Poor (*n* = 102)	X^2^/z/t	*P*
Age (years)	52.67 ± 19.15	59.4 ± 17.77	−2.909	0.004
Gender(%)			0.573	0.449
Male	121(67.22)	73(71.57)		
Female	59(32.78)	29(28.43)		
Type of pancreatitis (%)			15.023	0.002
Biliary	77(42.78)	50(49.02)		
Hyperlipidemic	59(32.78)	13(12.75)		
Alcoholic	13(7.22)	11(10.78)		
Other causes	31(17.22)	28(27.45)		
Medical history (%)			1.467	0.226
No	84(46.67)	40(39.22)		
Yes	96(53.33)	62(60.78)		
Received CRRT (%)			5.930	0.015
No	65(36.11)	52(50.98)		
Yes	115(63.89)	50(49.02)		
APACHE II score	12(8,16)	15(10,21)	−3.760	< 0.001
Marshall score	3(2,4)	4(3,6)	−3.019	0.003

Multivariate regression analysis (Table 2) revealed that both CRRT administration and Marshall score are significant determinants of poor outcome. Specifically, CRRT serves as an independent protective factor, whereas Marshall score is identified as an independent risk factor.

**Table 2:** Multivariate regression analysis of poor outcome

	B	S.E.	Wald	*P*	OR	95%CI	
						Lower limit	Upper limit
Age	0.007	0.008	0.666	0.414	1.007	0.991	1.023
Type of pancreatitis (%)							
Biliary					1.000		
Hyperlipidemic	−0.626	0.413	2.301	0.129	0.535	0.238	1.201
Alcoholic	0.593	0.471	1.583	0.208	1.810	0.718	4.559
Other causes	0.271	0.334	0.661	0.416	1.312	0.682	2.522
Received CRRT	−0.689	0.307	5.044	0.025	0.502	0.275	0.916
APACHE II score	0.048	0.025	3.789	0.052	1.049	0.999	1.101
Marshall score	0.192	0.097	3.923	0.048	1.211	1.002	1.464

According to Table 3, intergroup comparison results show that the differences in hospitalization duration and the use of vasopressor drugs between groups are statistically significant, with the CRRT group having a shorter duration compared to the non-CRRT group. However, comparative analysis of secondary outcome measures revealed no statistically significant intergroup differences (all *p* > 0.05).

**Table 3:** Comparison of prognostic outcomes between CRRT and Non-CRRT groups

Groups	*n*	Hospitalization duration (d)	Total hospitalization cost (¥)	Duration of vasopressor drug use (d)	ICU duration (d)	Total duration of mechanical ventilation(d)
CRRT	117	22 (15,31)	55,298 (34841,109556)	8(3,12)	3(1,5)	28(10,144)
Non-CRRT	165	18 (8,28)	72,729(42520,118657)	4(2,6)	4(1,6)	50(16.5,139)
z		−2.576	−1.330	−2.983	−1.223	−1.684
P		0.010	0.183	0.003	0.221	0.092

Table 4 demonstrates that among SAP patients undergoing CRRT, logistic regression analyses in both unadjusted (Model 1: OR = 1.010, 95%CI:1.003–1.017, *P* = 0.005) and adjusted models (Model 2: OR = 1.010, 95%CI:1.002–1.017, *P* = 0.009; Model 3: OR = 1.012, 95%CI:1.003–1.020, *P* = 0.007) consistently identified CRRT initiation timing as an independent prognostic determinant. Importantly, this parameter was established as an independent risk factor, suggesting that delayed CRRT initiation correlates with poorer clinical outcomes.

**Table 4:** Analysis of prognostic factors in CRRT patients

		B	S.E.	Wald	*P*	OR	95%CI	
							Lower limit	Upper limit
Model 1	CRRT initiation time	0.010	0.003	7.813	0.005	1.010	1.003	1.017
Model 2	CRRT initiation time	0.010	0.004	6.826	0.009	1.010	1.002	1.017
	Age	0.020	0.010	4.274	0.039	1.021	1.001	1.040
	Gender	−0.607	0.421	2.078	0.149	0.545	0.239	1.244
Model 3	CRRT initiation time	0.012	0.004	7.285	0.007	1.012	1.003	1.020
	Age	0.002	0.013	0.037	0.848	1.002	0.978	1.027
	Gender	−0.516	0.486	1.131	0.288	0.597	0.230	1.546
	Type of pancreatitis							
	Biliary					1.000		
	Hyperlipidemic	−0.165	0.599	0.076	0.783	0.848	0.262	2.743
	Alcoholic	1.461	0.670	4.747	0.029	4.309	1.158	16.031
	Other causes	0.913	0.576	2.512	0.113	2.491	0.806	7.704
	APACHE II score	0.117	0.040	8.583	0.003	1.124	1.039	1.216
	Marshall score	0.191	0.131	2.136	0.144	1.211	0.937	1.565
	Medical history (%)	0.544	0.461	1.388	0.239	1.722	0.697	4.255

Multivariable logistic regression was used to evaluate CRRT initiation timing's independent effect on outcomes after adjustingfor confounders (age, gender, APACHE II, Marshall score, etiology, comorbidities). The model (logit[P] = β₀ + β₁X_time +β₂X_age + ... + ε) underwent VIF testing (all <5) and goodness-of-fit assessment. Results are reported as adjusted ORs (95%CIs), with β₁ representing the adjusted time effect (α=0.05)

Model 1: Unadjusted

Model 2: Adjusted for age and gender

Model 3: Adjusted for age, gender, type of pancreatitis, APACHE II score, Marshall score, and medical history

**Fig. 2:**
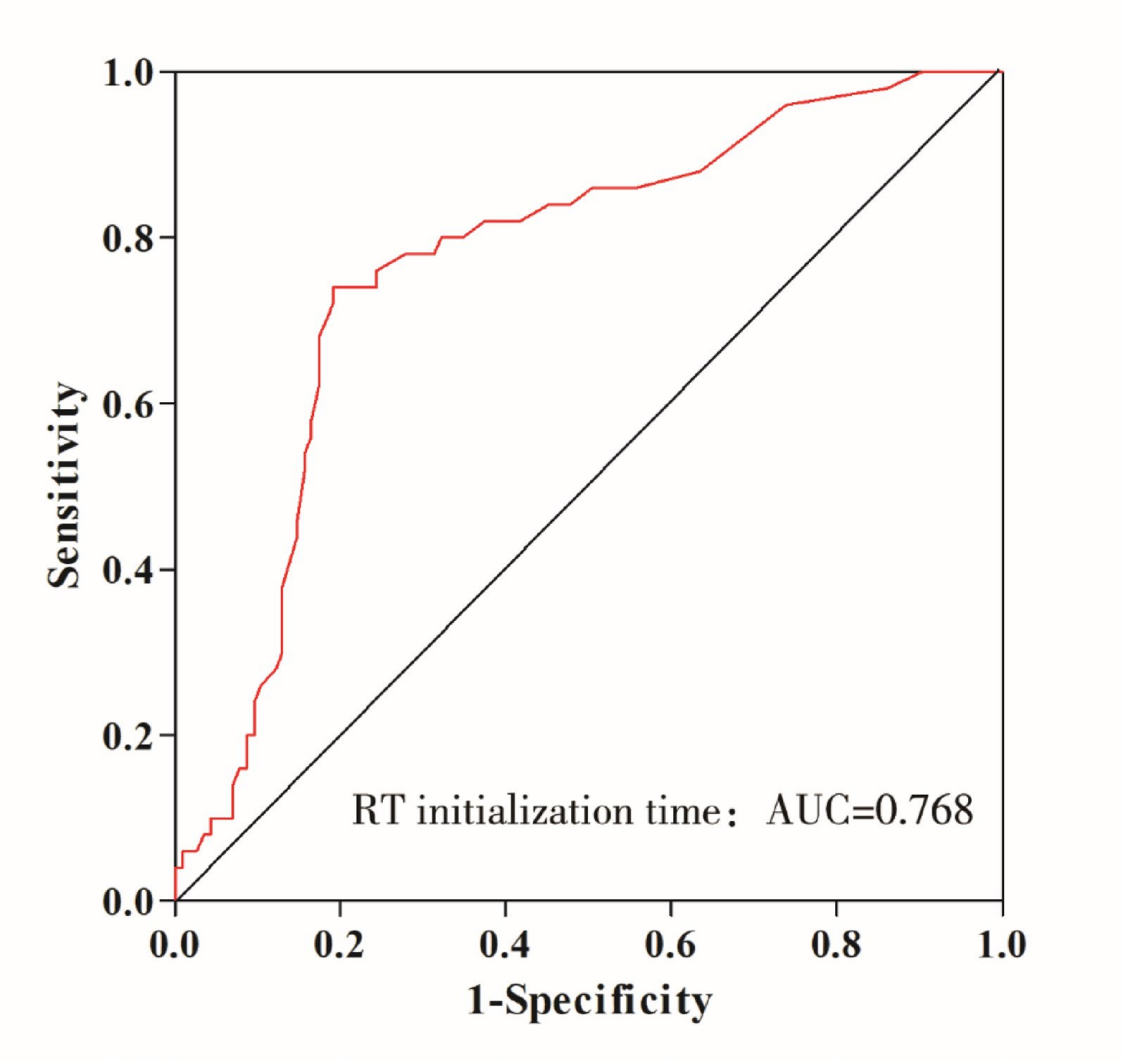
ROC curve of CRRT initiation timing

Table 5 demonstrates that the ROC curve (Fig. 2) analysis indicates a high diagnostic value of CRRT initiation time for prognosis (AUC = 0.768, 95%CI:0.690–0.847, *P* < 0.001). By applying the principle of maximizing the Youden index, the optimal cutoff value was determined to be 36 h (sensitivity = 74.0%, specificity = 80.9%). This suggests that CRRT initiation delayed beyond 36 h is associated with an increased likelihood of poor prognosis.

**Table 5:** Evaluation of the predictive value of CRRT initiation time on prognostic outcomes

	AUC	95%CI		*P*	Sens	Spec	Cut-of(h)
		Lower limit	Upper limit				
CRRT initialization time	0.502	0.690	0.847	< 0.001	0.740	0.809	36

ROC curve analysis evaluating the predictive performance of CRRT initiation time for clinical outcomes. The area under the curve (AUC) of 0.768 (95% CI: 0.690–0.847) indicates good discriminative ability. The optimal cutoff point of 36 h (marked by red circle) was determined by maximizing the Youden index, achieving 74.0% sensitivity and 80.9% specificity. The dashed diagonal line represents chance-level prediction, while the shaded area depicts the 95% confidence interval for the ROC curve.

We excluded patients who met absolute indications for CRRT and subsequently received the treatment from our analysis. The remaining cohort was randomly allocated into training and validation sets at an 80:20 ratio using a computer-generated randomization sequence. As demonstrated in Table 6, baseline characteristics between the training and validation cohorts showed no statistically significant differences (all *p* > 0.05), confirming adequate comparability between groups for subsequent predictive modeling.

**Table 6:** Comparison of indicators between the training and test sets

	Training Set (*n* = 91)	Test Set (*n* = 23)	X^2^/z/t	*P*
Age (years)	48.05 ± 18.07	51.83 ± 18.54	−0.890	0.376
Gender (%)			1.597	0.206
Male	64(70.33)	13(56.52)		
Female	27(29.67)	10(43.48)		
Type of Pancreatitis			1.915	0.590
Biliary	30(32.97)	9(39.13)		
Hyperlipidemic	41(45.05)	9(39.13)		
Alcoholic	10(10.99)	4(17.39)		
Other Causes	10(10.99)	1(4.35)		
Previous Medical History (%)			0.111	0.739
No	40(43.96)	11(47.83)		
Yes	51(56.04)	12(52.17)		
APACHEII Score	12(8,17)	10(7,16)	−0.877	0.380
Marshall Score	3(2,5)	3(3,4)	−0.268	0.789
O₂ Concentration (%)	37(33,40)	40(33,45)	−0.182	0.856
PaO2(mmHg)	89.3(74.2,122.5)	93.2(78.4,118.7)	−0.191	0.849
OI(mmHg·FIO₂)	236.3 (181.3,348.3)	250.8(198.5,342.3)	−0.201	0.841
PaCO₂ (mmHg)	33.76 ± 8.25	36.11 ± 7.13	−1.256	0.212
K⁺(mmol/L)	3.65(3.35,4.32)	3.48(3.02,4.19)	−1.374	0.170
Na⁺ (mmol/L)	135.3(133.6,138.9)	135.8(134.1,140.9)	−0.936	0.349
Lactate (mmol/L)	1.6(1.3,2.3)	1.5(1.1,2.2)	−0.619	0.536
Ca²⁺(mmol/L)	0.83 ± 0.23	0.86 ± 0.16	−0.642	0.522
BE(mmol/L)	−5.3 ± 5.3	−3.48 ± 3.94	−1.538	0.127
A-a Gradient(mmHg)	206(194,264)	201(190,280)	−0.438	0.661
Albumin(g/L)	29.91 ± 6.27	32.13 ± 5.25	−1.563	0.121
Total Bilirubin(µmol/L)	17.9(12.2,29.1)	20.7(17.2,36.4)	−1.673	0.094
Alanine Aminotransferase (U/L)	25(17,40)	43(22,67)	−0.834	0.406
Aspartate Aminotransferase(U/L)	31(24,50)	35(24,78)	−1.035	0.301
Creatinine(µmol/L)	65.9(48.6,103.7)	65.4(44.9,77.2)	−0.770	0.442
Urea(mmol/L)	5.28(3.50,8.45)	4.8(2.63,7.91)	−1.010	0.313
Uric Acid(µmol/L)	227(133,363)	217(124,343)	−0.487	0.626
Total Cholesterol(mmol/L)	4.79(2.97,9.03)	4.09(2.51,8.56)	−1.112	0.266
Triglycerides(mmol/L)	5.07(1.61,12.92)	1.98(1.10,9.51)	−1.731	0.084
Amylase(U/L)	231(110,523)	208(76,588)	−0.519	0.604
White Blood Cell(×10⁹/L)	12.3(9.5,17.1)	11.3(8.9,14.9)	−0.929	0.353
Hemoglobin(g/L)	131.97 ± 30.60	124.91 ± 30.94	0.986	0.326
Platelet(×10⁹/L)	191(143,243)	172(144,199)	−1.140	0.254
Prothrombin Time(s)	13.3(12.1,14.9)	13.1(11.8,14.7)	−0.025	0.980
International Normalized Ratio(-)	1.16(1.06,1.29)	1.18(1.07,1.28)	−0.502	0.616
Thrombin Time(s)	14.8(13.1,18.9)	16.5(14.2,19.6)	−1.787	0.074
D-Dimer(µg/L)	1391(617,2720)	1327(545,2637)	−0.145	0.885
C-Reactive Protein(mg/L)	217.2(155.9,319.3)	210.6(109.3,277.2)	−0.413	0.680
Activated Partial Thromboplastin Time(s)	31.2(28.9,36.4)	33.5(29.4,35.1)	−0.533	0.594
Procalcitonin(ng/mL)	1.96(0.61,4.59)	1.97(0.28,4.64)	−0.710	0.478
Creatine Kinase (U/L)	141(79,320)	192(86,362)	−1.020	0.308
Creatine Kinase-MB(U/L)	10.8(3.4,23.6)	9.7(5.5,22.4)	−0.222	0.824

**Fig. 3:**
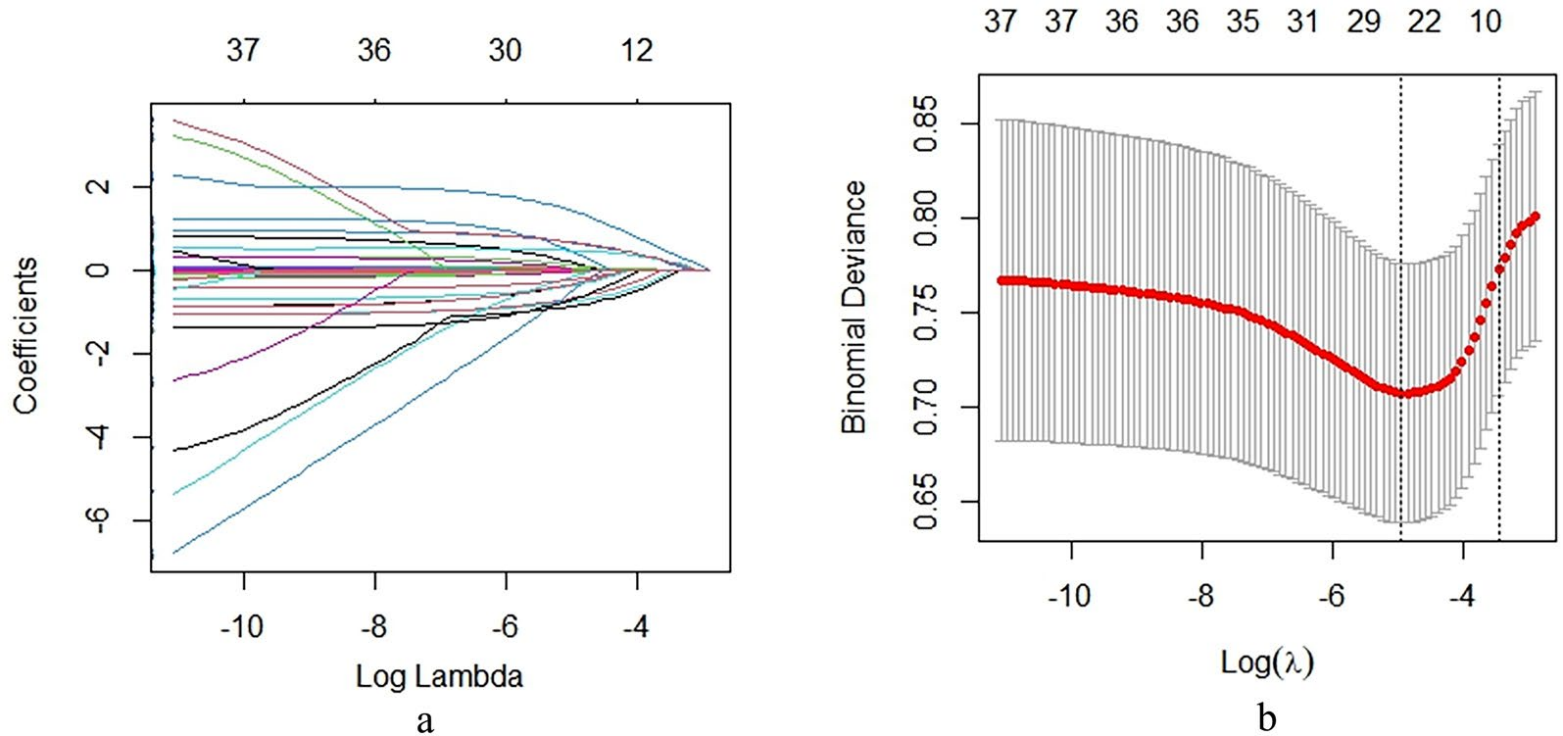
LASSO regression coefficient paths (**a**) and binomial deviance for optimal λ selection (**b**).

Initial univariate analysis identified eight factors with significant prognostic value (pancreatitis type, APACHE score, Marshall score, lactate, calcium, albumin, PT, and PCT; all *p* < 0.05, Table 7). To objectively refine these candidate predictors while controlling for overfitting, we performed LASSO regression (10-fold cross-validated λ selection) which precisely retained these same eight variables (coefficients > 0, Fig. 3). This perfect concordance between univariate significance and LASSO selection provided strong justification for including all eight factors in the final multivariate logistic regression model, where each maintained independent prognostic significance (all *p* < 0.05).

**Table 7:** Analysis of factors influencing the prognosis of patients in the training cohort

	Improvement (*n* = 68)	Poor (*n* = 23)	X^2^/z/t	*P*
Age (years)	46.61 ± 17.83	52.35 ± 18.51	−1.324	0.189
Gender (%)			0.386	0.535
Male	49(72.06)	15(65.22)		
Female	19(27.94)	8(34.78)		
Type of Pancreatitis			8.510	0.037
Biliary	21(30.88)	9(39.13)		
Hyperlipidemic	36(52.94)	5(21.74)		
Alcoholic	5(7.35)	5(21.74)		
Other Causes	6(8.82)	4(17.39)		
Previous Medical History (%)			0.291	0.590
No	31(45.59)	9(39.13)		
Yes	37(54.41)	14(60.87)		
APACHEII Score	10(7.5,15)	19(13,22)	−4.640	< 0.001
Marshall Score	3(2,4)	4(3,5)	−2.500	0.012
O₂ Concentration (%)	33(33,40)	40(33,45)	−0.992	0.321
PaO2(mmHg)	89.1(72.5,121.5)	83.2(76.1,125.0)	−0.187	0.851
OI(mmHg·FIO₂)	243.33(187.27,359.7)	220.12(175.56,347.50)	−0.301	0.763
PaCO₂ (mmHg)	33.64 ± 8.24	34.09 ± 8.45	−0.223	0.824
K⁺(mmol/L)	3.64(3.26,3.98)	3.65(3.49,4.02)	−0.818	0.414
Na⁺ (mmol/L)	135.2(132.9,138.9)	137.0(134.2,138.9)	−0.644	0.520
Lactate (mmol/L)	1.6(1.3,2.1)	2.1(1.5,3.2)	−2.439	0.015
Ca²(mmol/L)	0.87 ± 0.20	0.69 ± 0.25	3.499	0.001
BE(mmol/L)	−5.23 ± 5.43	−5.48 ± 5.02	0.193	0.847
A-a Gradient(mmHg)	206(194,262)	227(191,279)	−0.42	0.674
Albumin(g/L)	31.62 ± 5.23	24.86 ± 6.47	5.038	< 0.001
Total Bilirubin(µmol/L)	17.7(12.4,26.5)	18.6(9.5,30.7)	−0.347	0.729
Alanine Aminotransferase (U/L)	27(17,40)	25(17,42)	−0.16	0.873
Aspartate Aminotransferase(U/L)	30(22,48.5)	39(27,64)	−1.507	0.132
Creatinine(µmol/L)	63.3(48.9,96.6)	85.2(42.1,139.0)	−1.361	0.174
Urea(mmol/L)	5.14(3.49,7.73)	5.78(3.50,10.09)	−0.963	0.335
Uric Acid(µmol/L)	251.5(143.5,372.0)	183.0(112.0,340.0)	−1.164	0.244
Total Cholesterol(mmol/L)	4.85(3.28,9.17)	4.29(2.61,8.44)	−1.416	0.157
Triglycerides(mmol/L)	5.34(1.70,12.58)	2.28(1.21,16.21)	−0.356	0.722
Amylase(U/L)	217(94,510)	414(155,596)	−1.251	0.211
White Blood Cell(×10⁹/L)	12.2(9.5,15.8)	13(7.2,18.6)	−0.292	0.770
Hemoglobin(g/L)	133.69 ± 28.72	126.87 ± 35.82	0.923	0.358
Platelet(×10⁹/L)	200.5(152.5,253)	163(123,222)	−1.480	0.139
Prothrombin Time(s)	13.2(12.0,14.5)	13.9(12.6,16.5)	−2.206	0.027
International Normalized Ratio(-)	1.15(1.06,1.28)	1.19(1.10,1.33)	−0.662	0.508
Thrombin Time(s)	14.5(13.1,18.2)	15.8(13.9,20.8)	−1.484	0.138
D-Dimer(µg/L)	1278(572,2559)	1751(686,3307)	−1.05	0.294
C-Reactive Protein(mg/L)	223.6 (162.3,319.8)	187.1(109.2,319.0)	−1.205	0.228
Activated Partial Thromboplastin Time(s)	30.6(28.6,34.0)	32.9(30.1,39.2)	−1.799	0.072
Procalcitonin(ng/mL)	1.12(0.51,3.75)	3.41(1.72,8.78)	−2.448	0.014
Creatine Kinase (U/L)	138(77,296)	219(81,472)	−1.174	0.241
Creatine Kinase-MB(U/L)	10.60(2.78,23.20)	10.80(7.00,24.70)	−0.735	0.462

 The LASSO coefficient paths (Figure X) demonstrate progressive variable selection as regularization intensity increases (log λ: −10 to −4), with optimal model fit achieved at log λ = −5 (minimum binomial deviance, Figure Y). Notably, this penalty parameter retained exactly 8 clinically relevant predictors (coefficients > 0), corresponding precisely to the univariately significant variables. The binomial deviance curve’s U-shaped trajectory confirms appropriate λ selection, avoiding both overfitting (Fig. 3a) and excessive sparsity (Fig. 3b)

Analysis of Factors Influencing Poor outcome (Table 8):The intergroup comparison results revealed that, based on multivariate regression analysis, APACHE II score (OR = 1.188, 95%CI:1.013–1.392, *P* = 0.034), lactate (OR = 4.053, 95%CI:1.328–12.369, *P* = 0.014), blood calcium (OR = 0.005, 95%CI:0.001–0.642, *P* = 0.033), albumin (OR = 0.763, 95%CI:0.602–0.967, *P* = 0.025), and PCT (OR = 1.400, 95%CI:1.070–1.832, *P* = 0.014) were all identified as independent factors influencing poor outcome. Among these, RT II score, lactate, and PCT were independent risk factors, while blood calcium and albumin were independent protective factors. We found that none of the remaining indicators independently influenced outcomes (all *P* > 0.05).

**Table 8:** Analysis of factors associated with poor outcome

	B	S.E.	Wald	*P*	OR	95%CI	
						Lower Limit (LL)	Upper Limit (UL)
Pancreatitis Type							
Biliary					1.000		
Hyperlipidemic	−1.210	0.960	1.590	0.207	0.298	0.045	1.955
Alcoholic	0.290	1.423	0.042	0.838	1.337	0.082	21.752
Other Causes	−1.835	1.549	1.402	0.236	0.160	0.008	3.327
APACHE II Score	0.172	0.081	4.503	0.034	1.188	1.013	1.392
Marshall Score	0.168	0.272	0.381	0.537	1.183	0.694	2.014
Lactate (mmol/L)	1.399	0.569	6.045	0.014	4.053	1.328	12.369
Ca² (mmol/L)	−5.348	2.503	4.567	0.033	0.005	0.001	0.642
Albumin (g/L)	−0.271	0.121	4.997	0.025	0.763	0.602	0.967
Prothrombin Time(s)	0.283	0.161	3.100	0.078	1.327	0.968	1.817
Procalcitonin (ng/mL)	0.337	0.137	6.026	0.014	1.400	1.070	1.832

The multivariate logistic regression analysis identified five significant predictors (APACHE II score, lactate, serum calcium, albumin, and PCT), which were incorporated into a prognostic nomogram (Fig. 3). Model validation included: (1) 10-fold cross-validation (Fig. 5) showing stable accuracy (range: 0.55-1.00, 8/10 folds > 0.75), (2) bootstrap ROC analysis (Fig. 6) (AUC = 0.865, 95% CI: 0.753–0.978), and (3) decision curve analysis demonstrating consistent clinical utility across training/test sets (Fig. 7). Internal validation confirmed excellent discriminative performance, with AUC values of 0.912 (95% CI: 0.841–0.982) in the training set (Fig. 8b) and 0.844 (95% CI: 0.665-1.000) in the test set (Fig. 9b).

Calibration analysis (Fig. 9a and b) revealed outstanding predictive accuracy. The bias-corrected curves showed close agreement with the ideal reference line in both datasets, with maximum deviations < 0.05. The test set exhibited a mean absolute error of 0.062 (*n* = 23), maintaining prediction biases < 0.1 within the 0.2–0.6 probability range. At the optimal cutoff (sensitivity 80%, specificity 75%), the model demonstrated robust clinical utility.

These findings substantiate that our predictive model not only achieves statistical significance but also provides reliable support for clinical decision-making in acute care settings.

**Fig. 4:**
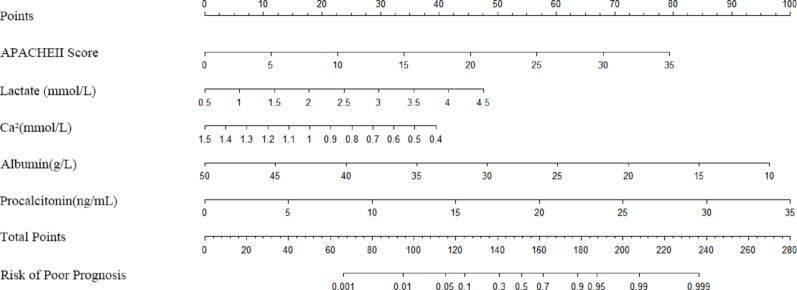
Nomogram for predicting prognosis in SAP patients initiating CRRT without absolute indications

The multivariable prognostic nomogram incorporates five independently significant predictors, including APACHE II score (0–30 points), serum lactate (0–10 mmol/L), ionized calcium (0-1.5 mmol/L), albumin (10–50 g/L), and procalcitonin (0–20 ng/mL), with each variable’s relative contribution quantified through a standardized point allocation system along the top axis. By summing the assigned points for individual patient characteristics and projecting the total to the bottom probability scale (0–1.0), clinicians can readily estimate personalized risk probabilities. This model exhibited robust discriminative capacity, as evidenced by area under the curve values of 0.912 (95% CI: 0.841–0.982) in the training cohort and 0.844 (95% CI: 0.665-1.000) in the validation cohort, while maintaining excellent calibration with a mean absolute error of 0.062, indicating strong agreement between predicted probabilities and observed outcomes across the entire risk spectrum.

**Fig. 5:**
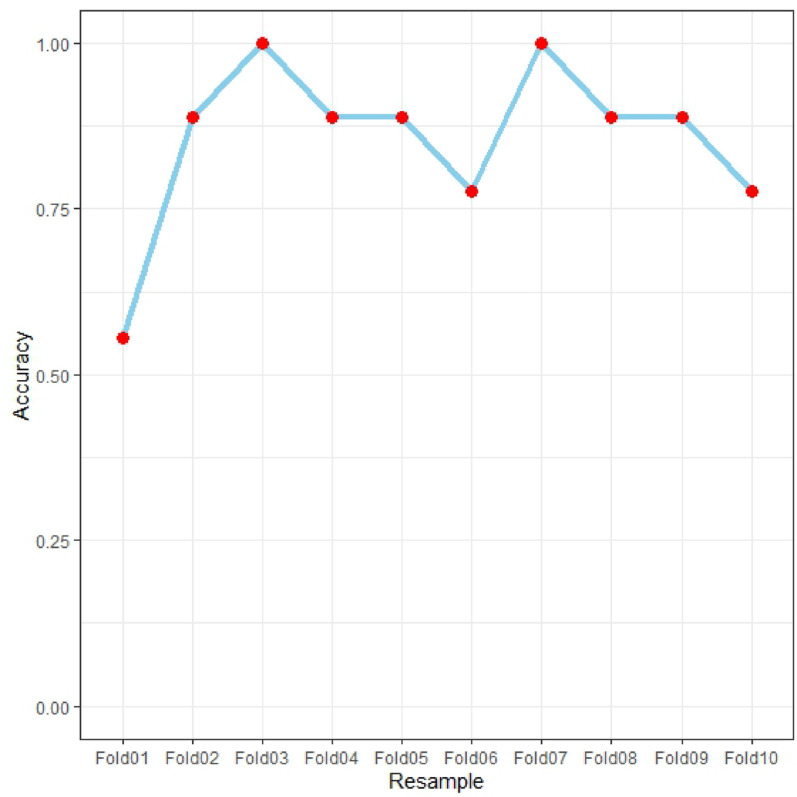
Model accuracy variation across 10-fold cross-validation​

The line chart demonstrates the model’s accuracy across 10 validation folds (Fold01-Fold10), with values ranging from 0.55 (Fold01) to 1.00 (Fold03/Fold07). The blue trend line shows consistent performance, with most folds (8/10) achieving accuracy above 0.75. The alternating peaks and troughs reflect expected variability while maintaining overall stability (mean accuracy: 0.78 ± 0.15).

**Fig. 6:**
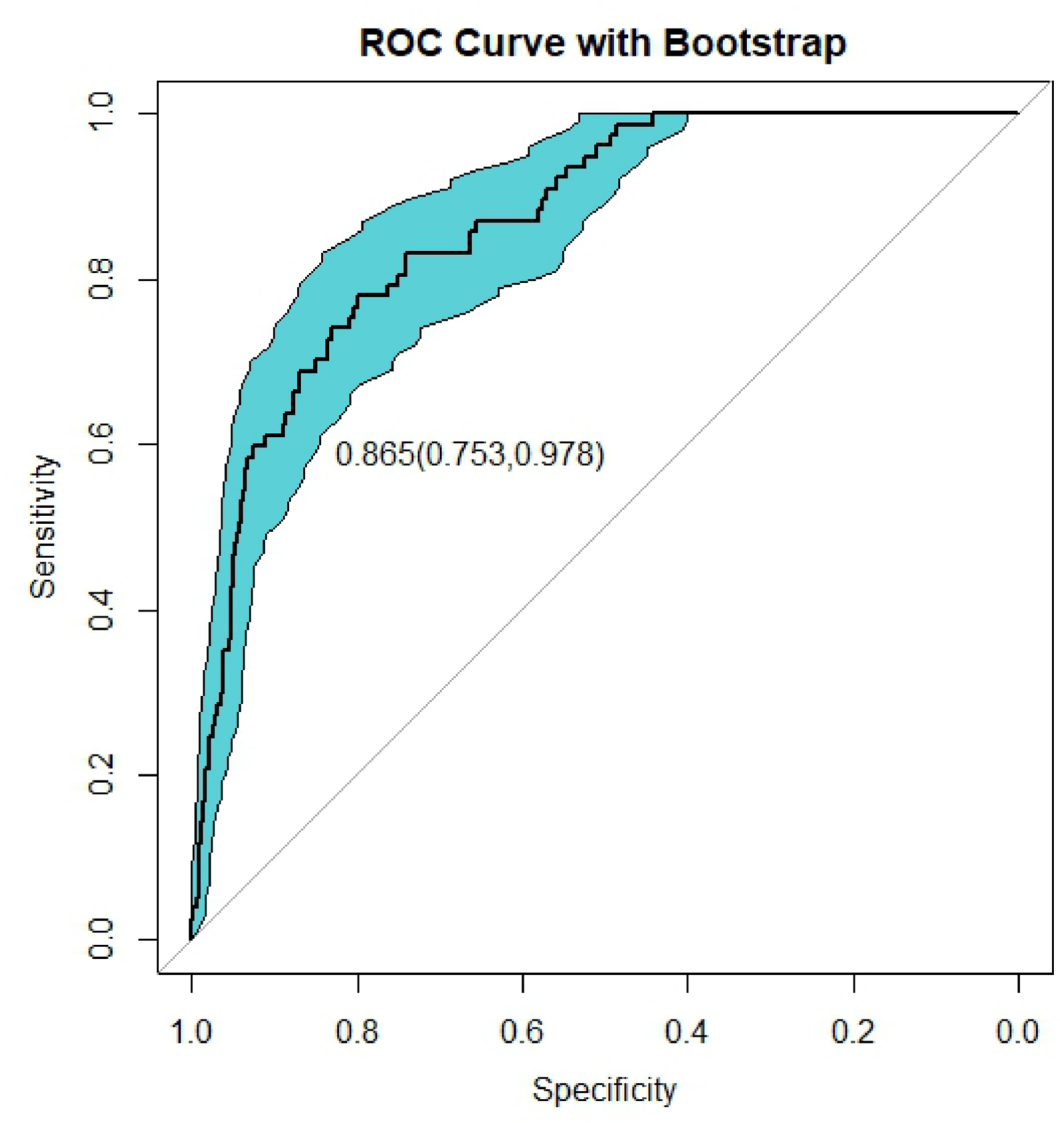
Bootstrap-validated ROC curve analysis of the predictive model

Bootstrap-validated receiver operating characteristic (ROC) curve analysis demonstrates the model’s discriminative performance (AUC = 0.865, 95% CI: 0.753–0.978), where the solid black curve represents model sensitivity/specificity and the light blue shading indicates the 95% confidence band derived from 1000 resampling iterations. The curve’s position above the reference line (AUC > 0.5) confirms the model’s predictive capacity.

**Fig. 7:**
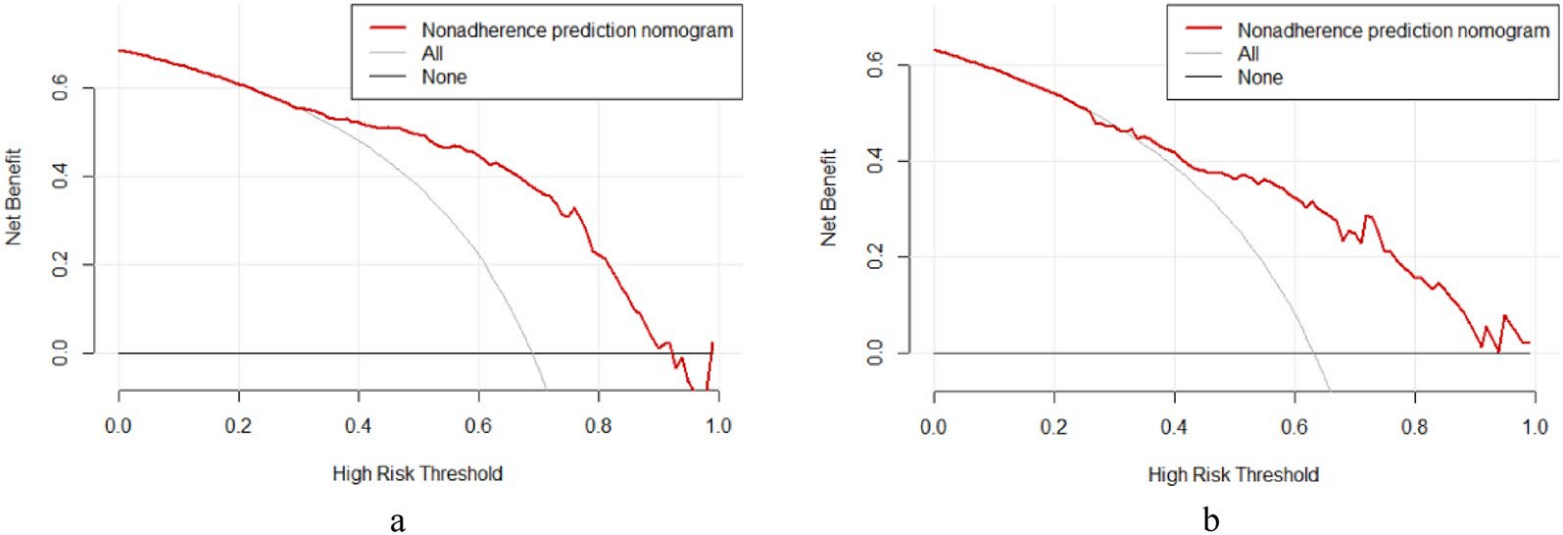
Decision curve analysis of the predictive nomogram (training set: **a**; test set: **b**)

Decision curve analysis of the predictive nomogram in (a) training and (b) test cohorts. The red line (nomogram) demonstrates superior net benefit compared to the “All” (gray) and “None” (light gray) strategies across most clinically relevant risk thresholds (0.2–0.8), indicating robust clinical utility. Both cohorts show consistent performance patterns, with the nomogram maintaining positive net benefit throughout the threshold range.

**Fig. 8:**
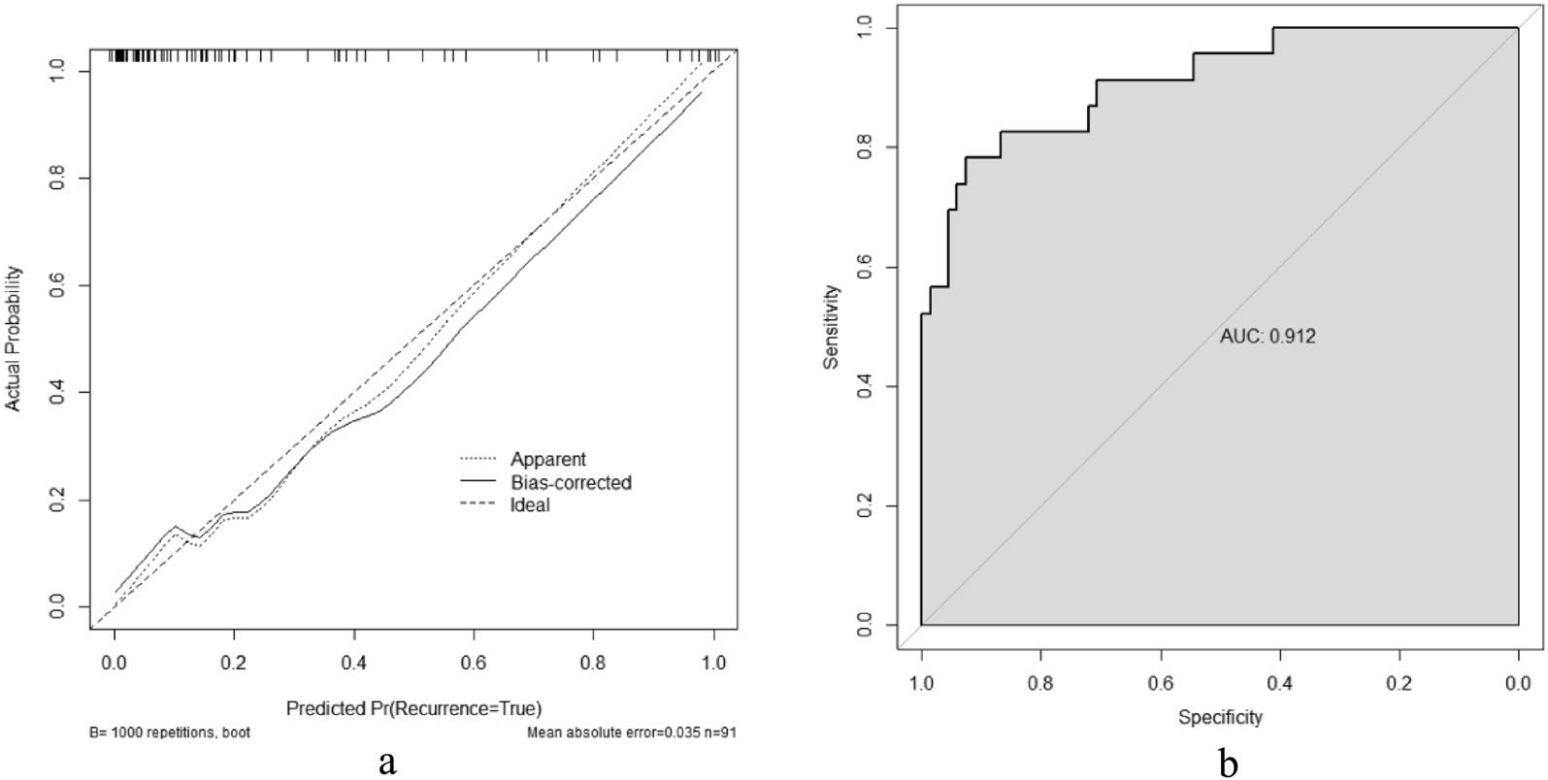
Calibration curve and ROC curve of the training cohort. (**a**) Training set calibration curve. (**b**) Training set ROC curve

Calibration curve and ROC analysis of the predictive model in the training cohort (*n* = 91). The calibration plot (left) demonstrates strong agreement between predicted probabilities (x-axis) and observed frequencies (y-axis), with the bias-corrected line (solid) closely following the ideal reference (dashed) after 1000 bootstrap iterations (shaded band = 95% CI). The ROC curve (right) confirms excellent discrimination (AUC = 0.912, 95% CI: 0.841–0.982), where the curve’s trajectory reflects optimal sensitivity-specificity tradeoffs across thresholds.

**Fig. 9:**
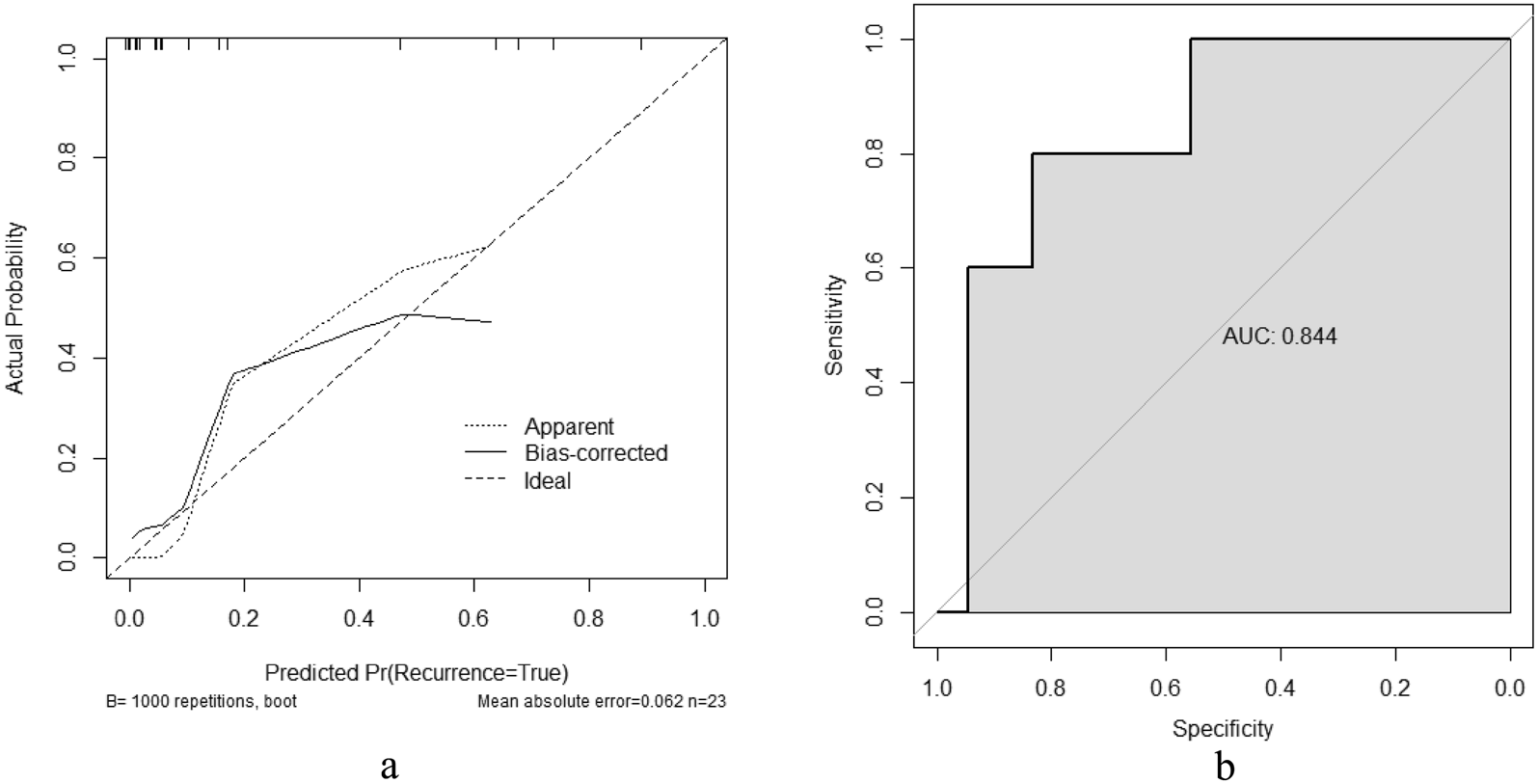
Calibration curve and ROC curve for the test set patients. (**a**) Test set calibration curve. (b) Test set ROC curve

Calibration curve and ROC analysis of the predictive model in the test cohort (*n* = 23). The calibration plot (left) demonstrates agreement between predicted recurrence probabilities (x-axis) and actual probabilities (y-axis), with the bias-corrected line closely approximating the ideal reference after 1000 bootstrap repetitions (mean absolute error = 0.062). The ROC curve (right) shows the model’s discriminative ability (AUC = 0.844, 95% CI: 0.665-1.000), with the curve’s trajectory reflecting sensitivity-specificity tradeoffs across thresholds.

## Discussion

### Controversies and consensus in CRRT for SAP

Acute pancreatitis (AP) is a clinically common life-threatening gastrointestinal disease, with approximately 20% progressing to SAP accompanied by pancreatic necrosis. Mortality exceeds 30% when multiple organ failure occurs [17]. SAP clinically manifests as pancreatic hemorrhage, necrosis, shock, and infection, with poor outcome and unsatisfactory outcomes from conventional treatments [18]. Its molecular mechanisms involve trypsin activation, calcium signaling disruption, mitochondrial dysfunction, and endoplasmic reticulum stress, leading to systemic inflammatory responses [19].

CRRT removes toxins and inflammatory factors, improves microcirculation, stabilizes hemodynamics, and corrects internal environment disturbances [20–22]. Studies demonstrate CRRT can reduce inflammatory factor levels, shorten hospital stays, and decrease SAP complications and mortality [23–27]. However, current applications of CRRT in SAP remain controversial:

- Supporting evidence: Early CRRT may block inflammatory cascades and improve prognosis [13, 28].
- Opposing views: May not benefit SAP patients with early organ failure [14].
- Absolute indications: Acute renal failure (urine output < 0.5 mL/kg/h), multiple organ dysfunction, refractory hyperthermia, or severe electrolyte disturbances [29, 30].

The 2021 international guidelines did not recommend CRRT [31], while the 2019 Chinese guidelines suggested cautious use in SAP with renal failure, albeit with weak evidence grading [32]. The 2021 edition removed this content [33].

### Key findings of this study

1. CRRT is an independent prognostic factor for SAP, improving outcomes, shortening hospitalization, without increasing treatment costs

2. The optimal CRRT initiation window is within 36 h of onset, potentially delaying disease progression by blocking early inflammatory responses (SIRS) [34].

This study specifically focused on developing a CRRT prediction model for SAP patients without absolute indications. Although CRRT initiation timing was confirmed as a protective factor, this parameter was excluded from the non-strong indication subgroup model due to:

- Methodological considerations: Avoiding bidirectional causality between treatment decisions and prognosis;
- Clinical practicality: Maintaining reliance solely on baseline indicators.

For patients with absolute indications (meeting KDIGO guidelines), CRRT is clearly essential with lower predictive needs. For patients without absolute indications (the “gray zone” of clinical decision-making), this predictive model provides evidence-based support for individualized treatment. Future time-dependent analyses will further validate the impact of treatment timing.

3. For SAP patients without strong CRRT indications, key indicators affecting CRRT efficacy include

- Risk factors: APACHE II score [35, 36], PCT [37–39], lactate [40–42];
- Protective factors: Serum calcium [43–45], albumin [46, 47]. 

4. The developed predictive model (nomogram) demonstrated good clinical applicability in guiding individualized CRRT application

### Clinical implementation strategy

The prognostic nomogram will be implemented through a phased approach:

1. Mobile access: A WeChat mini program enables real-time risk assessment (China’s dominant platform with > 1B users)

2. EHR integration: FHIR-based interface with hospital EMR (target completion: 2026)

3. Minimal training: 10-minute physician orientation (no nurse training required).

### Potential explanations for conflicting evidence

The protective effect of CRRT on SAP outcomes observed in our study differs from some neutral/negative findings in the literature, which may stem from:

1. Population heterogeneity: Negative-result studies [14] predominantly enrolled patients with irreversible organ failure, whereas our cohort excluded end-stage cases (KDIGO stage 3);
2. Timing disparities: Positive-result studies [13, 28] and our data concur on the criticality of early intervention (< 36 h), while delayed treatment may miss the inflammatory modulation window;
3. Confounder control: Neutral-result studies [48] inadequately adjusted for baseline severity (e.g., CRRT still showed survival benefit in the APACHE II ≥ 20 subgroup, HR = 0.62, 95%CI:0.41–0.93).

These discrepancies suggest that CRRT efficacy may hinge on dual thresholds of therapeutic time window and eligible population, aligning with our model’s non-strong-indication patient profile (significant benefit in those with low lactate/high calcium, *P* < 0.01).

### Biological rationale for the 36-Hour CRRT Initiation Window in SAP

1. Critical Window for Systemic Inflammation Control: The first 36 h after pancreatitis onset represent a crucial period for systemic inflammatory response syndrome (SIRS) development [49]. Early CRRT during this phase effectively removes pro-inflammatory cytokines (e.g., TNF-α, IL-6), thereby interrupting the vicious cycle of “cytokine storm” [50].
2. Microcirculatory Dysfunction Mitigation: Animal studies demonstrate that pancreatic injury induces peak microcirculatory disturbances and ischemia-reperfusion injury within 24–48 h [51]. CRRT improves hemorheological properties during this critical window, alleviating these pathological processes.
3. Infection Risk Reduction: Clinical evidence indicates that secondary infections frequently develop after 72 h in SAP patients [52]. Early CRRT (within 36 h) may modulate immune responses, potentially decreasing subsequent infection risks through timely inflammatory control.

### Reconciling clinical findings with current guidelines

The discrepancy between our findings and the 2021 international guidelines [26] may stem from several factors. First, the guidelines were primarily based on evidence from patients with end-stage organ failure (e.g., KDIGO stage 3), whereas our study cohort specifically excluded such cases to focus on the early, potentially modifiable phase of the disease. Second, the guideline formulation lacked high-quality evidence addressing specific subgroups (e.g., patients with APACHE II ≥ 20 without renal failure). Our data demonstrate that early CRRT (< 36 h) may have potential value in this subgroup (AUC = 0.844), which aligns with recent studies [23, 29] emphasizing the importance of the ‘therapeutic time window’. These findings suggest that the current guideline’s generalized recommendations may require more nuanced interpretation based on individual patient characteristics and disease stages, while also highlighting the need for future RCTs targeting different disease phases and severity levels to refine clinical decision-making.

### Cost-Effectiveness analysis

Our study found no statistically significant difference in total hospitalization costs between the CRRT and non-CRRT groups (median: ¥55,298 vs. ¥72,729, *P* = 0.183), which may be related to CRRT’s clinical benefits offsetting treatment costs. Although the CRRT group had longer overall hospitalization (median 22 vs. 18 days, *P* = 0.010), they showed significantly shorter ICU stays (3 vs. 4 days, *P* = 0.221) and a trend toward reduced mechanical ventilation duration (28 vs. 50 days, *P* = 0.092). Notably, the CRRT group required longer vasopressor use (8 vs. 4 days, *P* = 0.003), likely reflecting greater initial illness severity in these patients. These findings suggest that while CRRT increases costs in specific treatment aspects (e.g., vasopressors), it may generate cost-offset effects by reducing ICU resource utilization (estimated ICU daily costs: ¥3,000–5,000). Importantly, as an observational study, the causal relationship between cost differences requires validation through prospective research.

### Model Considerations

To address potential clinical interest in CRRT timing, we have included the time-incorporated model in Supplementary Materials. While this alternative analysis provides additional reference value, we maintain that our primary model (excluding initiation time) offers more methodologically robust conclusions, as it avoids the inherent circularity of using a clinical decision variable (initiation timing) as both predictor and outcome determinant. The original model’s physiological parameter-based predictions remain our core scientific contribution.

### Survival Analysis by CRRT Initiation Timing

The Kaplan-Meier analysis (detailed in Supplementary Materials) demonstrated a significant survival advantage for early CRRT initiation (≤ 36 h), with the ≤ 36 h group maintaining consistently higher survival rates throughout the 360-day follow-up (Log-rank χ²=11.331, *p* = 0.001). The hazard ratio of 1.247 (95% CI: 1.018–4.299) indicates a 24.7% increased mortality risk with delayed intervention (> 36 h). These findings align with the critical 24–48 h inflammatory window in SAP, supporting early renal replacement therapy to mitigate organ dysfunction. The sustained survival divergence beyond 120 days further underscores the long-term benefits of timely intervention.

### Multicenter External Validation

The external validation conducted across multiple centers demonstrated robust model performance, with an AUC of 0.833 (95% CI: 0.78–0.89) in the independent cohort. The calibration curve showed excellent agreement between predicted and observed probabilities (Hosmer-Lemeshow test, *p* = 0.32), while decision curve analysis confirmed clinical utility across a wide range of threshold probabilities (10–80%). These multicenter validation results substantiate the generalizability of our model across diverse clinical settings and patient populations, addressing a crucial requirement for clinical implementation. The consistent performance metrics across different institutions suggest that our model may be reliably applied in routine practice, though further prospective validation would strengthen these findings. (Please refer to the Supplementary Materials for details)002E.

### Model Validation and Comparison

To further validate our modeling approach, we performed a complementary random forest analysis (see Supplementary Materials), which identified APACHE II score, albumin, calcium, lactate, PCT, WBC, and creatinine as key predictors (importance > 0.5). The random forest model demonstrated comparable predictive accuracy to our primary Lasso-nomogram approach (AUC 0.923 vs. 0.912, *p* = 0.257), confirming the robustness of our findings. While both methods performed well, we ultimately selected the Lasso-nomogram combination due to its superior clinical interpretability and ability to generate individualized risk predictions, while maintaining comparable performance to more complex machine learning approaches. These comprehensive validation results (detailed in Supplementary Materials) strengthen confidence in our model’s clinical applicability.

### Limitations

- As the Cochrane review notes [53], CRRT efficacy varies by clinical setting, potentially limiting our single-center findings’ generalizability;
- While we adjusted for key confounders through propensity score matching (detailed in Supplementary Materials), residual bias from unmeasured variables (e.g., subtle differences in nursing protocols) may persist.
- The single-center design, despite external validation, limits generalizability to hospitals with differing CRRT implementation protocols.
-  Time-dependent analyses were restricted to 36 h thresholds; finer temporal resolution (e.g., hourly effects) was precluded by clinical workflow documentation gaps.
- Mechanistic links between the 36 h window and organ protection remain inferential, warranting future biomarker studies.
- Concurrent therapy data, though systematically collected, lacked granularity in dosing timelines for all medications.

### Future Directions

Future multicenter randomized controlled trials are currently being planned in collaboration with two additional hospitals in our city to systematically validate and extend our current findings. This prospective study will specifically address four key aspects:

1. Validating the conclusions of the present study through rigorous experimental design;
2. Investigating the therapeutic effects of different CRRT parameter settings in SAP patients;
3. Conducting etiology-specific subgroup analyses to evaluate CRRT efficacy across different SAP subtypes;
4. Examining the impact of initiation timing by comparing outcomes before and after the 36-hour critical window.
5. Performing comprehensive cost-effectiveness analyses to assess the economic impact of optimized CRRT protocols.

These investigations aim to provide more definitive evidence for optimizing CRRT protocols in SAP management.

### Key strength

- First integrated dynamic/static indicator CRRT prediction model;
- Precisely quantified optimal intervention window;
- TRIPOD-compliant model validation;
- Provides Level III evidence for guideline updates.

## Supplementary Information

Supplementary Material 1

Supplementary Material 2

Supplementary Material 3

Supplementary Material 4

Supplementary Material 5

Supplementary Material 6

Supplementary Material 7

Supplementary Material 8

Supplementary Material 9

Supplementary Material 10

Supplementary Material 11

Supplementary Material 12

Supplementary Material 13

Supplementary Material 14

Supplementary Material 15

Supplementary Material 16

Supplementary Material 17

Supplementary Material 18

Supplementary Material 19

Supplementary Material 20

Supplementary Material 21

Supplementary Material 22

Supplementary Material 23

Supplementary Material 24

Supplementary Material 25

Supplementary Material 26

Supplementary Material 27

## Data Availability

Data is provided within the manuscript or supplementary information files.

## Declarations

**Abbreviations**.

All abbreviations used in this manuscript were defined at first mention in the text. No additional glossary is required.
