# Supplementary material for: Application of continuous renal replacement therapy (CRRT) in patients with severe acute pancreatitis: an analytical study
Source: BMC Gastroenterol. 2025 Aug 18;25:592. doi: 10.1186/s12876-025-04198-y (PMC12359950; doi:10.1186/s12876-025-04198-y)
Supplement: Supplementary file 2 — Supplementary Material 2 [file 12876_2025_4198_MOESM2_ESM.doc]

探索


附注	
已创建输出	25-JUN-2025 15:16:18	
注释		
输入	数据	H:\u盘文件个人\1U盘3.0\2023年\9月份\20230901-1200-自己\20240418-1600-自己\未标题1.sav	
	活动数据集	数据集1	
	过滤器	<无>	
	宽度(W)	<无>	
	拆分文件	<无>	
	工作数据文件中的行数	282	
缺失值处理	对缺失的定义	因变量的用户定义缺失值视为缺失。	
	已使用的个案	统计信息基于个案（无任何所使用因变量或因子的缺失值）。	
语法	EXAMINE VARIABLES=年龄 APACHE评分 Marshall评分 BY 是否好转
  /PLOT NPPLOT
  /STATISTICS NONE
  /CINTERVAL 95
  /MISSING LISTWISE
  /NOTOTAL.	
资源	处理器时间	00:00:05.72	
	用时	00:00:03.65	


是否好转


个案处理摘要	
	是否好转	个案	
		有效	缺失	总计	
		数字	百分比	数字	百分比	数字	百分比	
年龄	好转	180	100.0%	0	0.0%	180	100.0%	
	不良	102	100.0%	0	0.0%	102	100.0%	
APACHE评分	好转	180	100.0%	0	0.0%	180	100.0%	
	不良	102	100.0%	0	0.0%	102	100.0%	
Marshall评分	好转	180	100.0%	0	0.0%	180	100.0%	
	不良	102	100.0%	0	0.0%	102	100.0%	


常态性检验	
	是否好转	Kolmogorov-Smirnov(K)a	Shapiro-Wilk	
		统计	df	显著性	统计	df	显著性	
年龄	好转	.072	180	.122	.971	180	.101	
	不良	.078	102	.137	.971	102	.125	
APACHE评分	好转	.089	180	.001	.876	180	.000	
	不良	.084	102	.072	.934	102	.000	
Marshall评分	好转	.206	180	.000	.821	180	.000	
	不良	.187	102	.000	.899	102	.000	

a. Lilliefors 显著性校正	


年龄


正态 Q-Q 图


_÷íñ#¸w<HVHVHV@²@²@²¬¬¬ddn§ªªÊÃÃãÔ©Swð*÷ð2÷¸é|÷Ýwûøð¤¤$·©S§v»rsssZ§¬îTVV~ôÑGòðåËßîÇíØ±CVhooïõ·Z¶lY¿ñøÃ_|Ñ÷õ+**´Q[[+?ºï#ïííëÖ­×<y²LÖmÈ]²üÂ¼øA²óõð¸Ý½N§ÓmåCÉíâÅeÑø×ë÷ß¿?qæÌn=·Kë×¯ß¸qÃÜc³Ùîèìúän®öñÇK#88xÚ´iú?þgdQ~nLLÌí~ÊåË»~·¸];vLVÎÉÉöÍ7¥ýÕW_õúÈPÈÓ§Oïõd|MÙ³g,¶¶¶výcóóóyñdî½¿þõ¯ZAv[Ù¬X±BîýýïoþDnllüäOÂÂÂÞï=Y<ú´öïÜ¹Sn¥xíù'JØÈjûöí3z.^¼(=~úiçUþ¢¹Ìõ0j8i¼ýöÛÒ#ínUóçÏ×TÌëW¯^öÁ¿ÿþû¤Nn ùç¾õÖ[Ò#%i¯a/m£½Ýß"O(u¹¬mþ¡Û¶mëúlF²»QÜËo®ýÜÀ¬ÀÌp«]zÝpªa#Ùf®tS°ôµl0a|ÐÇÅÅIçºuëtK¬OZÌõ/Y¥°3Wä©©©'N¸ÝC$2õçæåå½þúëÒØ´iþ¶gÏÕÔ×?Ðü¨k×®¹ýiRìö°áÔ©S²ruuµÑÓÒÒ"='Oìú-ÄíéËöj#YõkñÅH·$m$+0/Ö.AØC²JÝ¦ñ£¥Ï1ctQRGê××^Íøôï!»÷¤(ñ#)¼ÌëH¼u?ùú¬n¤¼'Ôí¨Òîv7oÃÆ¨óäöÂrûÙguÝ¦ª¹%wýØ¹ÓÔÏÏOçÏ×ÖäÒøðÃ»-Ù333ÝúåGK¿þ&=ü!òûhûv±m$«~mÒßÿôéÓúõåâÅ$+HV` H½8wîN·d5ÂÌmQrN7ÿøÓþEý¯¯¯×hn5>Ð°16 ïÙ³Çm÷Þ¯ëÐ-YÈý­tïï÷ßßÃNzoDDþ,M>ã3gêÞÊ+W®¸=ê«¯¾*++¿H"ù*·ÞzKcU·»výCìv·¿|#å_Ðm!.w-]ºÔxÜÜÜuß¾:2ò`Ú´iÒyìØ1ó:$+HVà[Ã!§îº*MJOÝkìd5ïÿÄÕÏå	&HÛétÐ¦M4N$_«««»=xÇ-YäâÏÃ·KÖ9sæhûã?Ö_RMÚÝ°úTr[UUe|	èº]WÈ/¬Æuù¤ó^Ð­©«V­ýÍ7ßE3ùóeôÉÍÅwWr¯·¢±T÷þj,)®e×ÝÕï¼ótêÒºòäÉ¶ü]UÛR¹êöm·c¸HV¬À@ÊFJê¶fu£¥päèdÞ_(µQ¯¸Êú!!!nûSu°QMö¬½nXü¶Z­ºSW¼×í®º]ÚØú*¿QyI8c[·¹u#êçç'_ôGÈ«««Ó|ó3¹û÷ï×´[=ÄZÆÐøõ26¤¿ÝÈû»lT5kV·Éªÿº£ß0RRR4³IV¬À@9xð y2k×d5hº²|ôëAªº÷Q>©:«Þ®sZº­YÃ ¤½páÂÚÙuªeUBNâ$))É¼%VILkt%÷JÎRµë¯­Ë¢4«n®Ü%=F-¥¿D©|·ÐòÇÎ¯FA/î6&nCÝítÚ ­É½yóæ>&«þ9BRYþR­wå÷×Öâ[ëxdîù ´0f'­ÁnSV´¶ÓdÜ<ÐÍ°ºÑ1Ýè¶q²dÊm=îfãÆwS³îÄÕ3jk)Ýr­Ú°Ñ%/µ[%ãðe|ÈÔ=º/SÜØÛí®Vãþìg?3/¦§§»­£ô¨f·¡ë9YÙPJ·.h0ëwjV¬À½×í~Ï[5Yu³­|èëñGRðÕÕÕé^UÝÚÓÛ£·dÕñ£>ºdÕOc^7,_:::´ßxÝ­?×Ï©ÛuõO>yò¤lì<îöÄF3gÎtëéö:OÉ|¬²Û¼acùm»MÓ¾lï¿ÿ¾>³q±É¸ú¬úùòe©,54º=2¨k@÷³º«áÇNõg>µ/Éj£§4Ü@²nÕM¾zì®Nä56ÒÉªÇèîß¿_âÓn·/P+iÛªµàíûíöÈÞn×Ô¹¼ºþ%«&´Ãíl$+HVà~Ð]·KVãt?òI-Á£Ak¤©¿j>¹R¯ñ`ãP$22R_ÓXßápÌ?_cXúõ`Z=¨Ø8ªVË2ãQz|¯Â·ë.[¨jnï2jÖììì:¥^ÑHÖCõð×é)&z&¿î:5ztgjÏ©ì6tòåC+--Íøµo¬ÆKYYÉ¸¯ôdC·KÖ+W®Ô××6né½É8~Øí=vÉmÚ¨Û	îNQRòN>½ÝÀæCD]]Õj5ÿnò=àv§!üñ§=Äæg¸ÝÏÒ,iºtéÒ®W8è6»¦¬äe×K)¸ÍÑ|éó$¾N Yþûì³ÏBBBé[×TÒM0áÍ7ß¼£ëîÛ¸ï+ÞÞÞnò?ZÜëùo÷«voÏßo~üéØ.^ð Y YÉÉÉHVHVHV@²@²@²@²¬Àà|£zx¼ýöÛ½®¦×3?xð Ûc?úè£ÁõsÑõûßÿÞÜÓÞÞ>«SöOöìÙS]]-k¾÷Þ=<Ëå		Y¸páÒN7ò¼¼<ó:çÏOïd<³0Ú²¾<ÜXùÖ­[Ò³cÇ·êµµµÆCz.^¼hôÜ¼ySzä×îÇ<¨¬À ±ÿ~sz?÷e±²²²k)¦°°°©S§öñ§vM-55Õ¬¯½öy	É-sÍ9³ÛKå'+TUUÉíÙ³gîÛ·Oz. ?Tþ¨Ï>ûLúO:%ýðÃõ/uKë=¨¬À c$¥YTTd~ü±Ü:t¨ëû¬æçÅÌÌÌÊe­»-¥XÔ5+**´_bìØ±cæ5¯_¿.+,[¶L~o-_ýõþ¬äç$+0´´´¤¥¥I£¶¶V>úO>ÝsÍz7ÉêV³._¾üvkJQ(e_LLÌÕ«W¥Lôóóknnv^ùU;::¤ñ×¿þÕètÛ(ÝÞÞ.'Oo²(ù§?ú^üuÒþâ/îàÓáýdÕ+ÞÃCrKn?üðÃ+W®[5ÍÉ*%×ÔNÆ¦cÅ"áª»BuK²¸Ý§Õûö¹·Õêêê-Ò®ÑÑÑÚöìYé·ÛíæßY¾È:o¾ù¦¹ÈÖÍ­ú$ÒÇ³nÝ:i?^rQ]Kd7·«øs²^ýus &%%u­YíÆwT³:NK»ÐLÓ5¥k<PrÔüüZË+Bý ¯¤¾7o6ÿtM2«Õ:gÎsì$ï¿ÿ¾ï£ô ~.@²[ïß/mÝíz÷[5µääMëGDD¤¤¤G±yú¿F-÷J·SÁÁÁ³fÍw:ÆVe=IþØ¾×¬÷üç$+04I¥Ù ·æÂôîg§¼ÔdÕy¤¡[JÃk5ö³u¸ÛÆäÆÆÆùóçw=*Êpgò¨eËi®)ýÞà6Ù´/ÔÏHV`ÐÐ£X[ZZ¾ÿþ·óÜ«du¹ßQ2¦×µÉªdu®dX·	§ZGGGHHqò±cÇ¤@8öl?ëAýdÏË½3'ÜÚ			Z õÕW]ròäIéïû¤L#¡5ÝêT·u[«ÛÖ`Ãa¬sùòeãX¡ÖÖÖ/Î5ë/¾pÛHk0ïï4ÒQHÞÑX=¨¬À a>CÙ7òòòzÈÝ[VVÖóóëaGÆì;7J¾æççKdJ=§SS®^½jNÖ®qk<ÆqôùÉÛ¥µÛY)´Óí$PÕûÿs¬¬¬¬ddí÷ß?"""À¸qãþö·¿îdýõ¯=úôÓ<zê)óöÁ¬ÿüÏÿüýìg$+$+É YIVÉJ²@²@²¬ddd%Y$+É YIVHV@²¬d`P'kccãÔ©S-KBBBmm-É Yïjuuµ4ä·Óçd¬wfÿþý?üðGwfÏÍÿ@²öÉÍ7%>wìØ!Î.^~ùejVÉzg<8fÌö³HÖÆb±¬õ®ÄÄÄ466JãØ±c³fÍ"Y$ë]9qâDBBT«Ó§Oïèè Y$+g¬$+$+$+É YIVHVHV@²¬dd%Y$+É YIVHVHV@²¬¬¬$+d%Y$+ÉÉÉJ²HVd¬$+wõõõ«W¯þúë¯IVpWrss===#""Æ'yóæ¬$+ ***¼¼¼¶lÙ¢555eåÊ$+ÉèÙ³g$VCCCIVÐÅÅÅæ)[IVÐÏ5==ÝÜGÍJ²úý¬$+às;6Ø­%YIVÀ«¯¯_ÝéÈ#ÃðÏ'Y YIVÉJ²HVd¬$+àïZ[[wïÞ]YYÙÒÒÂh¬»²aÃÐÐÐÌNV«Õí¼Á$+É¸R§FGG744èbSSÝnß¾;ÉJ²ú###£¼¼ÜÜ#±vOÜáp$''º9¬þKLL:ÕÜ#±±±wù´­­­ÙÙÙ>>>)))G%YIVF5«Û¶ß]»vÝMÍêr¹JJJÂÃÃívii©,º1!Yýwo÷³îÞ½;>>ÞßßùòåN§s	É¸ã²R²sÕªUyyy6l°ÙlzlphhhQQQ?S²Y*]3gÎêñ!Yw@JI@©,%P¥BÍÍÍmii©ìÔPlkköññINN>pàÀ¢=Y;6yòdÅxâÄ	¬5kÖ¤¤¤j%%eKJJú÷l¥¥¥áé.ÕA¬111úûíØ±#..dKbµ¦¦ÆÜS^^y§Ïãp8ä©¤TUây(Ñ`Ú ·Ovz¯æNzl¯¤öîÝ;wîÜ¾?CKKÎ¨n·§"Yï«ºººeËIã÷]$%%¬0 NçñãÇO<ùòË/çççïZ´h[Ïí¸ÂÂBÍ½÷î¡:V#Y¯^½uíÚ5¶ÀýWRR"q(eLbbbxxxHHHAAÁ¥KÚÚÚV­Z%1ÙÚÚÚëHÚívÿ5kÖÈcðpdmoo_ºtiGGÇÀÕÈÈH©Vu±²²2(((%%E²644TÊ^¯ocÌ¨äæßA¬cÖ¬Y/_fÖ<ñññna¤èÌÍÍíËc5]d`¢¢¢<LHV¸Ï¤6uëìõèË¥3jäáRõ5C!Y9S<ðÕíj3ÅÅÅ=×¬½ò()Ueµ!6£dÜ­6HLÇ(=zT*QÃÑíÊMMMYYY©©©Ç#F²zg³Ù$2çÎ+·²*§Ó¹fÍ«ÕÝí$+Éø_gÎÙµkWeee·l$JÉ0°?:|fÔ¬ÒÖÖëãã#Õê>¡É¸^õUOOOîèíí]]]m¾×åréYDaaá°QC²ú«¨>úè$Sýüü$eõ^Q#+dgg÷zö%d|'9*±ÚµÇQìv!9¬ÛìjÕÜ3~üx«Õ:Ä.QN²¬pÕ¼cuïÞ½«Ò?O¨D²îÅbñõõý®sF^£F<õÔSÉèÃëÁr«Õj×òdôUyyùØ±c½½½¥x1ccB²úÃáp$''3£d%Yànµµµ-Z´ÈÇÇ'%%åv×±ÉèÉ%Kâââ$¤ÞiË-Ì¨!YIV¸cß|ó§^^^£Gööööðð8q"3jHVú)888000--MòuæÌ/¾ø¢ëçÎÈ¬$+Ü1§Ó)9kÆjµr0ÉJ²À«¬¬d-,,5ú£££ì1Æd%Y ¯=ª(ÏÌÌÛuëÖïõööÎÈÈ`HVz×ÖÖççç:uêÔÜÜO===5;'U¬4+d¸6H ÚívIVÿ§~züøñ«rëñÅòå_2+É=©©©·Z­>ú¨D©±cuýúõ®_ýµ*¥*ÉJ²@/Î9åããÙÔÔ$=^^^ÍÍÍæu$hsss+dt©   44411q÷îÝ»víZ¹råÛo¿íííí¶fjjêìÙ³1dÛ*//X-))9öìèÑ£-ÝnòððX»v­yåààà¼¼<d%Y ÇÔÇÇ'77·µµUz"##Í;V#""$ÊÊ¤ÝÜÜhÏd¿kkk4Ld|ÕÎúúzOOO·C¼¼¼¤ÓÛÛ[n%weFd%Yà¹Í]^^þ]çÌÔ+W¦wv[ßn·Ë§å×_M©J²¬àîÀñññþþþkÖ¬¹téôHdZ,	Z)^ÃÂÂ<<<JKKÍ·lÙÂÐ¬$+üMMMYYYrÛÐÐ`ôK¦&''²Îÿ÷ëâ¼yó$Y¹NÉJ²Àÿr:R¡Z­V©V¥f5ßU__ïããcÞÌîÜ9Ý«ªÇK9[SSÃ¬$+üCyy¹d¤¦%%%.K;,Xàçç'	*Á)ÉêöY?--M>!7nÜÈHVþÁ<£Æ¼9799YbuëÖ­ÒÞ°aGll¬¹À¸­¨¨`IVþ¡Û5êÈ#RªgÎL8QÂµ¨¨è»Î£ÆÁ¬$+üËå*,,4Ï¨Q7nLHH=z´¤æ1cÜ%%¬«$±®¬ÆñJ$+É·÷nÝ¥jÌ¨QòY'^\<aÂ	Qi(»zõjf¬¬$+üCCCCZZDfvvvKKô´¶¶nß¾½¨¨¨´´TbÕzîÜ9YMz»dÑí6 YIVÃT[[[^^Orr²1=FáááYYYR¼J*÷ê%áÔúõë%££srr¼¼¼Idï¤ï$ËU__¿uëVùh¬¬¬Ôu$5G!¹kL¹V«uòäÉ'N=6ÓUIV¾s8)))RJÁ*eë×_m³ÙdQn===Gilì»¤GÒ÷Ì3ÚSQQ!=¦D²¬ðw---ÙÙÙ¢iiizBÉHÅ"U©¦éo¼áçç7eÊã!3fÌ(]°`§999ÞÞÞrËH¬$+áÎQc·ÛwïÞmô¯^½Z:ÅÊÊÊú§2£$Ñëëë&;zôèõë×3$+É`¸3fÔH¸êÓçnäÈþ$JÍèåå%Ñ«©$+ÉÿÐÐÐiQ#tZê´iÓ^yå½:ÍÚµk|þùçÒ3vìØÔÔT	cy yz+HVÀ0%q¸jÕ*©AÍGð®[·NSOüûÝOW§ºèt:Ç 1|äÈÖÖVFd%Yw.«¤¤$22R*Î6¸iÊ)aaaæ;vHÖÙívÅ2zôhý%YÄåË£¢¢HVËâãã¥T]±b[@~òÉ'¯¾úªÜîö(IÖwÞygåÊRËrBu@ÔÖÖÊO^jFOE3fÌHKKã	à!qæÌQâp8Ìw­]»Ö£§§§6¾üòKãÞck0HÖ~öìY#Yøá .,ËìÙ³ù_xà¤Ê,..¶Z­v»ûöíæó%i©*f<òÈ¾ë¼pë%KdåI&I[ªUd½Ì5+[<ÊÊÊÂÃÃ%VÝ®Qc=z´Tæyóæ×4d0|=z455U>©²²²³vãÖ)bIVþAjÓÜÜQ³wïÞWÕßßßÜóê«¯¬$kRðí·ßÖÆÔ©Sõ($yÍåääìÙ³'**jòäÉ$+! ¤¤$<<Üf³mÙ²Åmj·t?«|ìêb]]§§§Ä-#I²öï¿ÿ¾6¤Q]]-í.H»²²Òjµ¬µ#G¤¤¤HÚuF)-ÃtIz,4äVfÉÚ=ùþ%Á©ª¬¬,¹­¨¨Û/j²q¦TSSSFFÎ¨ÑkÔôL5&&F>5bñ_Ì7ïÉ'äx%µw·nÝÒú2$$Dõ¥^=ökVÀ`téÒ¥¼¼<«ÕÛuFM·äÃP>ý¤l5z¼½½Ù±J²ögkð~(yýM6ÍHVù¾&Å2þ|Àà²k×.»Ýîïï_TTÔLUºÕ×Ü#)K²¬ýIÖnkV¶ÒÒÒ|||rssQcÖÐÐ°eË6¬$ë@Õ¬²$+A¤­­M7ÿ&&&<y²5=ZRR"QzäÈâââÐÐÐ+VäççyyÉ§ßáÃ5ÙL²ÞdÝ¶mtÊ×=¶WQQÍf.++ëyóoaa¡F©Ä°4ÿë¿þKïr:]`3g#L²öÕW_¥~%P¥±|ùriËKóµ×^Û±c®CÍà!·wï^ã5½^iüÀ¾ÆAÂK,yäG¤x5VØµkä+#L²Þ7nh¾îÛ·ïæÍÆóÊé^ Y<ä$ù$S-ZÔÃÃjêíí-lÜ5wîÜõë×Ï9Ó¼¾Ûy@²Þy©I*mÃ!í;wÊíÉ'IV-óÝ»w÷°¦GÉ××7¯î7Ý¸q£Þ»jÕª¥KÊ¸UÀ0ÉÚÏdmooWd§ÑsâÄ	éÙ³g´[[[u[±Q¿¬[¶líaFqÅbX5ú%J:Uï#xâ	»Ý^^^Î ¬ýIÖ7nÔÕÕu­_%PóçÏkK²x444è5jrssØ¥*ñ)Ñ»ª¬bÜ%Q*®q¸¯Ãá9rdbbbxxxd§]»v1Î$ëÃx­À½ÕÖÖ&1éïï/1)qØu7ÞxcÒ¤IS¦LY¸pa||¼	ÚïééTYYi¬©×X4MNNÂW+T§ÓÙóùA²¬Ë¥×¨Ñ²ëæßsçÎéJ²NXX¤æØ±cm6ÜmôxKÖï$ÊãÞ'ë¡CªªªHV¡½÷JqiµZo·ùWÕÇÇGòUõê4ºØÜÜ¬óg~ÖIÏQWWÇØb`kVyýá0Ú³fÍÊî¤G0íß¿dp544Ì;W>fÎÙuFÍ7ß|óØcHµ*ëüû¿ÿ»qWff¦Ýn06z~ûÛßÊjÞBCCU`²VWWgeeUVVçÞ·oÜFEEI¦ÉzâÄ	Àãr¹òóóýýýccc8Ðu/¿üÒÓÓS²sÉ%RÊÇÔÈ#<Ö8ý¯T½RÔö|²C¬÷ò*rò¢lnn6×¯¯¿þºyÑ8É` é5jl6[3jGe,J%*A»xñb£GÖËË+©Sdd¤ùð%``Ut¬qqqæÕ8kÉ`à?~|æÌòÓÒÒòÊ+¯èY$5%)Ý¨ßu^Ü¼EWÖi···O4Iÿã?þcð õÔ©Sñññ/^ÅÏ>ûÀÓÚÚëãã²uëÖ3gÎdeeéQHUUUþÌÉjZjéWòxÓ¦M*X²êUqåÊÍÑôôti~]Ö^zÉ¸Éà3®Q.«Õjpv;¾ä«T®K,ÑEÅ2eÊóóH*O6MÙo¾ùQÅNVqúôi¹½yófHHÈo¾év×åËu` ,[¶LQBTêÑ'x"11Q/T^__/±±±æý¬QQQ			Ú^·n^¤K÷Ë/¿9r¤Ô©nU,ð`õûï¿OéÝ©Yóóóu[1Éàjii	&TTTHIúøãë½Û·o72jÔ¨I&®R¹ê&·ÀÀ@ÉWFKÍzíÚ5·­´)À©¬¬:uª©òióÊ+¯hg[[[xx¸Ô[·nÕ©e¥-,,ÔÅªª*Yÿ>`1õÖ­[	tb«¼výüü*;é×IÀ=qáÂ=ÿáðáÃÆ½¬S¦LyþùçuQªUYAªR©JÒ`18Õ¸þùíjV·¢dÐÇ×Ï9³¥¥¥´´TÃUâVWÈÎÎ5jTff¦.îÝ»×f³=ñÄÒµråJÆ#YµZÕ©«ÆìUy5»õ@?TTTL8QO@(Þÿíw:´/¾ø¢ö|ùå²øÈ#äçç/Z´H>z¾9ð&ëõë×¹Ö®¾¾^nóßxyyùøøX­V)=%8Í¥çúõë¥Gî*..^°`NfÝµkWaaaYY$¾d5ïm¸1åæÚµk²(/n@ßI%,Å¨Ä¤¤¬¹¹¹­­­ÕÕÕz6%]%=!!!¡¡¡111«W¯f1¤µ¶¶V^âÇ3'ëÀ]Zd$»Ý.I¹sçÎ´´4ÍÑÉ'÷ê©ÿõ_ÿUõ¬IÍÍÍf²Êë[¾Q<x033³²²rÛ¶mz2&iüñÇÒÞ¼y3É «µk×=ÚßßÔ¨QR¡Ê»[Öèèè^x!<<Õ8C]]îm5æ¡nÜ¸ÄÐLV­PÝ®W¯^	ZjV=xòÉ'å³"(((&&F7ÿJaºfÍK.9rDw£ST¿þúkYGÊYyÔ/¾hÁdýðÃå°gÏiKcÓ¦MÒcl>A?É@òÉ'òù°téRÃ¢ÍifÏ-Ï?ÿ|MMÍÊ+ýüüRSS7ýd½qãÆüùóåÕñâEÝ,é¶¡xÛ¶m$+7'N´X,òÜÇÇ'>>þ?ÿó?õDEEEº¼Ó¥=z´¯¯ohh(sR1U·Ëí_|¡Z¿ç³rVÝjT¨6­¤¤DO¿qãF=Ü7''GrWV¨¨¨`Ä0¼õÂaaaæ°v[³rVJïøÔ/â±±±Æ½zJÂgyFbuöìÙGaÄ0kV·ø¤fÐÓélnn1c| $&&ÊmjjêñãÇõ£ººZÖ)**ö#.¬?R³¸	Ô=ùæhtttyy¹ÞkL¤Q6#~A²R³èF»¤¦4BBBÆÿoÿöoºcuáÂÆjß~û­ôüå/²LÉz5kccã­[·HV`8øüóÏävâÄ:oùòå:cÕX9,,Lz4¬Ý'ë¶mÛk²ºÕ¬«V­ÛÚÚZÚÎ;'QþÍ7ß455É?44´¦¦FWÖ£~úi???iKÜ2n YÝÝ¼ySÞ~úé½-LIV`°¨««öÙgåÃE¾Xûúú:Î5kÖX­V)^%>ÍEj[[D¯ÜJNN£õ¡@²ÉTÝê«Ç(ICòUÚyyyû÷ï×mÂÆÊ&MAÉÚù®:§SV§Êh[OÍ/môò±"íãÇKÁ*rÛÐÐ +¼ôÒKÒ3~üøÔÔÔÀÀÀàà`óeàµûMÁnå]ôáR³CØ+¯¼n±XüüüÚÚÚrss¥NÕëEj»Õj1bÜ^PP k2z Yûs4Éaaaa			Ó¦MÓÍ¿AAA6­°°Ðåréiôe1::ÚËËK¢×¸@²¬þaõêÕÉÉÉS¦Lyì±Ç$J¿üòKé<rä^*Õ××·¥¥E×|ñÅ¥gÆqqq/&VA²öÇ­[·äôÚk¯uMÖ7n¬À`tôèÑ6H|ÆÄÄøøøÌ7oÁ«£õõõYYYÒùè£êæ_=Ï¡0""Ézæ³iªô&m¬ÀÃOÂ²  @jSi¬Zµ*22Rnóòò½½½O8¡«é,aæÕW_ÕpÕÃU¬¬w³5XÞÀò¶X,			"Yûàùçì$IÍÖÖV½kÜ¸q¡¡¡R¼JûÀºùWncfÏ-A+ÕªD¬Ñ Y®db·¢¢B7o^¸p!É4©S||öíÛ§S§NõòòÚ²e¬K,IIIIMMwwrr²g'	Ñööv½|¬À0dx5,,L§ñÜ¸q#**ê~°v!ò5ÿ%ÐoR_êÆ[aÜø_übâÄºøÌ3ÏKÖÚíöíÛ·»YAõR­¾ñÆ'HÖ:Y-[»¢ùÆÿèw¬êÕÜ¤î<|ø°ù"©"33ó¹çý®óRä<òÜëçç÷·¿ý­­­-==]"Ù(p<do¸wôTòØhË­ÁÀ=qôèQù>*y.Ñ(±jÜ5zôh©AK;__ß1cÆ<ñÄ>>>Ó§O		5jng¨ªªb<JÖÊ×[=8))É8Øhúé§z°CßSÞÏ:WGn¥M²ý¶dÉ¸¸¸gy¦  Àf³mÙ²E¾é¶¶¶Ê»2>>þÒ¥KºÚêÕ«õüøºX^^®!*ïÔØØXãJ5<Y¯_¿~ÏgÔ,^¼XòXr»¬@ÏÚÛÛ¥ît9r¤F©q¯,&$$=ºNdd¤°4ËÊÊÚÚÚôpu ÔÖÖFDDÈ×gy;vdú!::ÚÇÇÇ8ÿ¿¿¿äåºuëtQQ¹×¸0êô*ªBússs	T`H%+gú§ººzÄ^^^z5·­[·wÙíö±cÇN0A£$å¬4ôXß¹sç9s®d7ç9sHVà>[»v­¼û¤6öÙg'N¨5¨qï+dqäÈºØÖÖ9~üø©_½½½ccc9Â0i²¾ûî»æï¿ÿd§§çèÑ£Íòf¸ÕÅK.I|JnÙ²¥¨¨Hbµ  `ûöíºcµ¸¸Øår1ÀC¬c~'=xÇòf6mqô²²2½*2ÉÜs.7êã?.o´ææfã®é1T½ÜÛ5k¤x]µjÕÿøGYÁÇÇG¹`*ðp%ëõë×Í=ß¶m[XXXff&[VWW§ûJG¡;VöYóºëTJUB£/µ¶¶ê%ÊO<É0]²v½Ü¡Ct¶É=¾W¯Ýö]çD±ûv£Åÿüçk×®Ýºuk»tw*//gó/0UßØÚÞ¿¿n"Y"o±ÃßØzdê'|¢z3r÷À)))roaa!3jA¬×¯_·tXX©ªªÊWùZöìY¸'ZZZÆçëë,ï/·u°ut·kSS^¢<##5À HÖ+W®ìÜ¹3$$¤®®NöìÙ#ïêS§NI»££#//OÏå-k¬ÀÝxï½÷ËËhÃf³Wðôô|òÉ'E§Ó¹fÍ«Õ/5+d½|ù²|qÖ<yRâóâÅònÿäOÌ«Ý¼y¸©nª¼¿ë<óeÉ8<¸¼¼Ün·Kô°KL5«4u8wµ|GV­­­¨½ÒÞ¼y3ÉÜ¥>úHÞM---nYkÐÕËÀ?~<55UORÈ`P&«¼¥÷íÛ§Éª»T%_õÔ©SÒxýõ×IV >øàððp©G-K×«¦Ò¿ûv=RIrTgÔH²J¾2zÀ LVÝ*¥ª9YUYY,ýï½÷Éô]kk«Ü®ROíûÊ+¯7N¯ÏèV³êyWIIÍfËËË@`p×¬Æ6aÝ$µ¸ÓÂ¥Â¬ ï$ åÛjhh¨¤ÜÊ(==Ý¸WOXXXXø]çAÂzS]]Ýâãã¥®]³f3j¡¬è·µk×fddÈí¯~õ«3gêÜ>ø@¢tÑ¢EÆj¦ÿ|ÍÊÊÜ6440ÀLÖS§N<xÐØÿZ]]M²=øüóÏ¥ôôòòÖtÿþýz×¦M¤?22Ò|B«ÕúÔSOM6íÍ7ßr%3j¡¬æÓ0IÃHYèÄêØ±cõÔ»víòóóóõõÕ»Î;'o¢ÄÄDãÃÒ#Ë`¸$«Ãá·mm­.=Vn"Yn­]»VªRUQSS£g(Õ3fÈbBBësÏ='Emxx83já¬:uÕíàoC²b°tÔ¿rk>ó|»¤ç7ÞÐÝ»wøûûËåGaF0UÏüÚk¯u·YYY$+ ôÞÄÄÄ«Õ*mISãÞ?ýéOÒ3wîÜÒÒÒ+VÈ]÷îu¹6Ín·KÖ2ÀÐOÖk×®éÉ"K =ÿè£¦O®;_O8a¾+ÉaH·îJ|=Z¼îÜ¹SÚß|óMpp°_QQQnn®¤iSSD©îREv©Ã%YåsáôéÓzV]:uªÞUUU%:Õ522R2dÅ0ÔÞÞ®ój,ù.ISýê©'¤GïjhhHKKÎììl·âÉÚ5hÿûßkÛ¶m²xõêU¶cØÚ´iîO5æÕ;wÎ¼ôüò¿|ã7ÚÚÚòòò$kkjjC`X'ëùóçÍm¤ÏL¶¤42e.þæ7¿Ñ+ÈDz¾ýö[£§´´4¼4Øü¬Ò9sæHVC.|øpFF1?Uê?~úi???Ý¢w9)U¥`eF@²þÝ¡CäcB>PÌÉI²bX@5®L®;PÝV*ÖËËK7çää|×yÚÂììlY3--$ë?|öÙgò!¢1>ZËÊÊî4L$+Z¯Ò0a¼ôQã^=¬Ï8;ËåÚ°a3jÕý*7aaaòyÑõÒ7Ë-ãìVöYÝr£Æ¡¿¥_~ù¥¯¯¯¼_ôÞ$&&2£ Y¹Öà.++käÈþþþ^^^æ»ôd¿Æ¼YMRöäÉ©©©zfÔ$+Éü3fÌxåWtëÖ­[¥§¼¼|íÚµR³ê	¬V«T«÷îeôµ7nÜ^zdÅ0¡'ÊÈÔÅO>ùDÞR¹+H	++hûöí±±±6­¬¬Í¿ÉÚ'zÔÒ©S§Ìßÿ=ÉÁ®®®îÙg7ÏÆÍýcÇMHH0÷<ýôÓºíWTO»_]]íp8õ5zas$ëm¯g.¸²²R>GäÓDæ³/ÚJ²b0zòÉ'å5ìíí­ûJc$Y'Md^Y¯«:qâÄÑ£GÏ1ãÐ¡CòF¦¤¤=zÁHÖ^Ü¼yÓhËs=áÙ³gâ$+W_U^Ø/¾ø¢.ÊëPIM]Ô­ÁæJ	«'v:ÅÅÅV«5>>~×®]lþHÖþF¯Ïúé§ÐéIVÜOûöíDô÷÷5÷/]ºT^áÆb@@³+W®Ü¾\ÜµiÓ¦Ý»wGGGËcóóó%bLd½c¯½ö| ,_¾¼²²ÒÏÏO>k*;IÖ¬Y$+õë×KXJ.ÉÛn·»­`NV!êãã#Åk`` Dlff¦,Î;]ªÉÚOn;Sí&&&¬Djjj¼¼¼$ uÑb±Hd+èöá®ÚT¾¦Êk]ªÉÚÿd=tèähmm­|Ö466vMÖ8)?É3eÊÔÔT·õóóÓE·ý¬ÊårFFFÚl¶v©$ë]¬%©4HV^7n4FÎ9æcbbÌÇÇ)Oõ5ùùù Yï*Y/_¾ÜÚÚjD,ÉAJOï0räÈÃ777k|+¬_¿>88øÙg6m¹¿¡¡AgÔÌ9Í¿Éz`2'kTTT\'éLKK#Yñ0óõõ1b±X\¬ª.ÊËØßßÿùç7?ÄårÉjÒXYYÉæ_d½÷Éªg½xñ¢´###%YoÝºõî»ïJçæÍIV<Ì<==Íèw?mþ4iRrr²DlBByæLyy¹Ýn·Z­ìR0Éj&É*LÚÖ+¶¬xØlß¾=  ÀØ±:sæLó½:cuÁRªOgØÐÐ kÊ]-bFû¬ò¹Í9ðÐÒËO0áNR³Ê¢ùÊ3R§ÒÖÖ¦3j$Y9Â¸¯ÉÊUäð³ÙlQQQÆ¢ ä½1cÆ///i×ÕÕé½:£&:::<<|Ë-lþ@²ÿ[ªfee-^¼X²Ó|	Uñ/ÿò/R¹zw5bõÀñññV«uÍ5.]b¬À?;V§¢úûûKãÑG5ß!w$u×»T¬Àÿ1iÒ$óõÉG)i¿ð»Î«S¦LÑ¶ÓéUêTQÃè Yw«o¼ñ±¨PzaK§öööï~Qc³ÙÙ¥`è$ëåË£¢¢HVÜ%cÇj×è/Y²Dâ6 Óc=öÍ7ß?~<55UÖÌÍÍmmmeôd­­­7®#ÖvÆÿ=Ð«V«Õ××W¯¹d¾7!!aäÈÚnkk4õññdeF!¬ééégÏ5'ë]¬èAtt´yÇê1cäµlÙ2]n,¾ñÆ.«¤¤Äf³EFF3nf²§`k0úMbuíÚµæ¥¨ÇÏ=[gÔH©Ê$+ÉnlÜ¸188X2U/VsîÜ9ó½Ï<óÕjî¹ç²²²vîÜ©3j-ZÔÐÐÀÐÉj¾dEÿTTTH¦¾øâN§óë¯¿×Åb1_-UB7..NztFT«Û·ogÜP³¬ø_­­­ùùùRzJXJ¬wI§¼uqöìÙ²(+Ûl6	Wóµkd%Y/):9ræÌ£GJL._¾|ïÞ½eeeòÉËË3¯9jÔ(½Ìªp?**Jn333Í,£dåLèjÅR¦¤¤DGGOþ 	|üøq£'===11ñw¿ûÝSO=åããäp8C$+É(**¤ÔSøJÝ)c&Én·nØ°A-Ë/~ñÍÆ5j¬$+º!iÇéÒ¥ÈÈÈgyÆ8©¾¾ÞËËkÔ¨Që×¯Äª¯¯¯ªË/gó/d;IÇèèhsÔ¯óçÏ/,,4²vÌ1ñññ®³fÍbFd»	&Ì1cäÈ---F¿ÃáðóóËÊÊø¬©©INN^ºti^^Ô©±±±»víbè¬$+þ¿üå/zX¯>IDFF³eÊËË%kùË_J"õ«Ô¬:£* YIVtÃ³Ó·ß~«zîßQ£F-Z´HÖ³ç=zTå®®Qd%YÑ½?ýéOR¶ºemDDT«555R¶µµ­X±ÂÇÇÇn·Kd%Yq[,èzQU??¿ÀÀ@mÚ:0£ÉJ²¢?5«ô7îèÑ£ÉÉÉRªæææ2£ÉJ²¢¯t?ëÿügiûí·R­J²¦¦¦ê%ÊÍ§[dE÷"##ýüüF-%©···ÅbæåHV"Q*EjNNNMMM^^äè3¤f2eJhh¨Ä-3j¬$+úª­­MbuãÆFä«ô<ñÄR­ê!%$+É¾ªªª2ûN§sÕªU©R­8pñ@²¬è	&X,í,--´Ùl#FøÝï~Ç( YIVôÉÊ+½½½gÏ]\<oÞ<©PccctFÍÿøGOOÏ¯¿þ@²¬èÝ¹sç$8·lÙ¢­­­ãÇpþío+Aëåå%ÿhÉJ²¢OÖ¯_!Ë%5«Íf³ÛíAAAcÇ8q¢º@²¬è]AAÁ¸qãvíÚ%*±ZXX(+ek^^d%YÑW,ðóóóôô´X,zþììl½ðj½ôWUU1JHV ±úÑGIa*½öªnõ­¨¨4i£d%YÑ'zæµk×FFFþùÏX__ßôôtãÂæ@²¬èÅK/½$ñéãã#«qààà­[·28HVâr¹Ö¬Y#*©···ÛI%k÷íÛÇ@ YIVôÉ9sÆ/©Ýn_»v­ëôéÓåÿ(ÉÊ`$+É>Y½zµÅb±Z­:£F$ããsrr&NèééYQQÁ@ YIVôâäÉ3gÎâ'Ìw%&&>ýôÓ«³gÏ®©©a¬¬$+zât:þy)Fbcc¥í¶tr=8$+É>Ù²eKPPª?þøË/¿)mó¡¿²$«ndÅm9äädooo©VþÄÄDéÉËË+--ÍÎÎ¬=räÃd%Yq[----òññIIIyþùçcbbÌ÷¶µµI²¾õÖ[Ë//**Ò³ÉJ²¢.]ZµjÕjÞ¾»Ëå7oÞÓO?í¶n=Ãd%YÑ²²²ÈÈHÍVPP «ë×¯÷óó3ÏO­¨¨ðööf¸¬$+nëÈ#IIIækÔIÖ?¾¹¹YÚUUUþþþ/¾ø"d%YÑ¶¶¶åËûøø$''Dvnnî¼yó¤~LdõôôÕävöìÙd;ËUTTdëTXXhÞÞ»~ýzIPIÓÔÔÔÀÀÀàà`Ý«*·¶ÉJ²¢÷îõððÂ´µµÕ|T«««W¯6zìvHHd%YÑÉÔ´´´nç¡JÖJµjîinn¬5.$+É¿»téÒ+|||¤ZÝµk×íVc¬è]YYÝn÷÷÷ÏÏÏ7fÔtkýúõ²Ól¬$+ºwôèÑÔÔTQsæÌ¾<Ä<ÍFbUV¦Ù YIVüFnþMLLÜ»woß(¡Ól¤Z]°`	d%Y5ËURR"¥gtttiiiÿ.Dãt:Ù·d%YñÝâãã¥Üµç]ªnîÆóòòªªªC YñwiiiYYYwt)rÉTIâ»ÝîååÃ!¬$ë°&µ©:£f÷îÝwôX·SC;wÎf³¥§§3ªHVuª¬¬@-**êûæ_hBB¹§¢¢Â××@²>¼ÉzìØ±É'[,ÄÄÄ'N¬÷Î¨Rµï3jº4iRNN[§Ãdx5&&F¿;vÄÅÅIãÿëâå_&Yû®µµUgÔH²vÂ;ªY'NhîÙ²e5+uÐløá<ºÃåÉúÂétnØ°!¼SiiéÝ?¡îg²UZª©©	7oCdÉZWW·lÙ2iüµçµWºKÕßß?//¯»ToG¯g.uªÍf:¡@²d½zõjVVÖµk×ØÏÚ'O;w®^£¦ß»T&ÕêÖ­[Ï;ÇhÀÃ¬Æ6^]loo_ºtiGGÇß)©M×¬Y#Õd||ü fýÑápÌ5ëòåËÌº¹SeeeÑÑÑV«µ¸¸¸')ìÓé´NIIIJJÊÎÎnjjb¨`0%kTTùH%µ/jjjtFÍòåËïíæ_Iè´´4Uùùùù¡¡¡wy1¬)âá%9*u¤dªûQÇÇÇÏ_XTT$?u¨j²  Àf³Ùíö~_£¦Wyyy6l0÷HÊÊå$ë"¥¤ª¿¿ÿªU«ôøùùùÅÅÅæ¶¶¶ÐÐPÞH@²r&:pà@tttkk«Ñ#YÅ	HÖAOjÓ¼¼<«Õ*ÕjMMÍû¹Ë/*yïÞ½- miiá$ëàVRRi³Ù¤1@»TPZZ*:wîÜüü|sý Y%	6¶¶6^Á@²¬wKÔ£GÞÃ'ljjÊÌÌÍÈÈhhhà$+óYûéÌ3©R;Î¢¢"ÉWÂHVµrrròóóÍ=²¸hÑ"FHVôGrr²[*¬è·ÿÊbRR#$+úcÍ5sçÎ5OÝÉÌÌÌËËcddE8N)[ÓÒÒ*++wïÞ!ë¥K YÑOR°gv*,,$Vd%Y$+É YIVHVHV@²¬¬ÃËåÚ°aCVVN³q:	¬$k?O!fÎ)íûùt YIÖ!¢ëé%hKJJ YIÖþu»pú®]»233 YIÖþHNN>yò¤¹gïÞ½RÅ22@²¬ýãv)ììl·HVµ¯Î9^PPp©Ó5kìvkk+#$+ÉÚOMMMsçÎíÖÐÐÀÉJ²HV@²¬¬¬$+d%Y Y YIVÉJ²HVd¬$+d%Y Y YIVÉJ²HVd¬$+dÐ§Nj±XjkkIVÉzW$P«««¥!¿eXXÉ Yïýû÷'&&Jcav»=--ÿ%dí7oxxìØ±d¬÷ÆÁÇÃÖ`ÉzÇ<~âÖo±XHVÉzWbbb¥qìØ±Y³f¬õ®8q"!!AªÕéÓ§wtt¬3EHVd¬$+$+$+É YIVÉJ²@²¬d¬$+$+$k¿¼þúëAAA1÷Ýn0aBú<Çgúè±ÇfúèñÇg¸îãÐ÷á·ä½N«Õ:èõüùóÿï^öÙg%YÿúæW¿úUHHãÐG¿þõ¯=<<>zë­·d¸>øà¢/Þÿ®µk×2±uëV®W_õÞ>íþô'Ë5¸u üîw¿6mÚèM6=òÈ#CUUUÉqè£ºº:®ööv¢/ÚÚÚd¸þö·¿1ñ?ÿó?2ÕÕ÷çÇ¬$+ÉJ²¬$+ÉJ²¬$+ÉJ²¬$+ÉJ²¬$+HVd%YA²¬$+ÉJ²¬wâ>X°`/»>Ú¶mÛÏþsÆ¡¾øâØØXÆ¡d¸N'CÑ2E_ÜºuKëðáÃ$+ÉÉÉú°9qâDdd¤ÅbIHH8tè/n;vlòäÉ2J2b[_<xÐ8páêÁõë×.]êçç7aÂÃÁpõJF)>>^Ç§¶¶»Ë/GEEõði?Ðã6|5++«¢¢B7o^¸p!¯ÅnÅÄÄèY1wìØÇ¸õêæÍò]ÄHV«ï¼óÎï½wëÖ-	»ÝÎpõ*,,ìÂÒ[MF¬+ùÎ!ß?Ìåw¥7áü·´4nÜ¸aþvÛ	`Üzõî»ïê	]yõJÊææfÞ'ß?tVÜêwF¬«ôôô³gÏµë(ô¸ßdµX,Ý¶Ñ­ºººeË1n=J"))IÞ±Æ»áêù=¸qãFùÆ&!Q__ÏpõêÄ2ê[yK2b=e)Y»Ò@ÛðMVooo£íççÇ±W¯^ÍÊÊºvíãÖ³ÌÌÌ¯¾úÊü®f¸z~IãôéÓòáêÕÔ©SµÊÕSÜ0bIÖ®£4Ðã6|uÌ17nÜÐ­Òæx;íííK.íèè`Üúòf6c¸zºÕW¯U>#Ödí:J=nÃ7Y/^üé§JCn¥ ãØ-Ã1kÖ¬Ë/3nýW3X±bÅÎ;¥ÑØØÂpõJêT=éÒ©S§¤~eÄú¬]Gi Çmø&kmmmDD··wddä±cÇx!v+**Ê­cÜîè]ÍpõàÊ+R%%%=áêUKKªÜJëc²v¥7ÎÉÉÉHVHVHV@²@²@²@²À½xL:5«Seeå¶mÛ¤ç¯ýkëëµez ×oóÍ7uñÚµk²¨S@²CÊÁ3;IJÚÕ××Ë­^Ëûöí3zâãã%z³;íÙ³§ººZV3gÑó?üAz®b<¤¶¶VzËèsVUU1þÉ5×¯_7'OÛºº:mÜ¼ySSPXW»pá,^¼xQn§O®ækîjçÖ#¬)nÔÁéééÒþøã¥½yófþÉÚhnnkooÛÆÆÆýû÷KÃétº%kdd¤¦¯ÁÁÁR¡JCJÒ-X+**¤çÔ©SnU¯<§âW¯^5?':´T|Ý¹s§Öº÷Ê+nÉjÔ z-hÝz¬®^oÓWþðÃ¥SBWÚÆ6mMbi|öÙgü#:._¾üÚk¯IÂíØ±C£Q*NI;iHÙ5Yeñ«¯¾ÒºëÖ­ÎÓ§OGDD,Pæ'¿qãÆüùóu²>633Ó-¤åyø/$+0¤ìÛ·Ï8¤H÷³nÞ¼YË-ÓdÝ¿¿®)ñ)÷êvcÝEjìdÕíÀçÏwN©åö/¾0])UÃ´äå_¬À¢¥á×ÒÒ¢(a)e«4.`Ô ºîµÛí:é1JÚ°¼uë<JÚº¾Qw­Yõ.$+0Dè®ÖcÇÉíGdÞ»ï¾;aÂcÑétÑX­¬¬ìúTzØðÍ7»Æ'5+@²Ãàëá±jÕ*mL<Y;wîà6Ö1v Ê½²¸xñb# Í1Óí fHV`XÐ¿.KÚÓzTú%Y5ó¤ú4vµêÑLZÖ××Ë­¬¦|¥`í!¼©Yúôè$ó1G|òô´¶¶þøÓÔÔTCóÖÝ+W®h§¤©¬¸Ò#ñ|§5kccc©¬ã×¯_DLHHp8½>ªªªJËÂ	iiizÓé»BBBº&ë¶mÛô²¾[ÍºjÕ*=Ýÿd¸¸¸cQOÕôÎ;ï=W¯^u+[ucò§~Ja¬¬¬dddd$+$+$+ Y Y YÉÉÉÉúåÿu!w¶òIEND®B`


X¿÷Þü©©©N-·K7nÜ¼yÓØâïïG ë;ý]j³>ø@üüüÕ_tñâÅ»òs###o÷S®âúÝâvØÚÚ*çææÊòðð°,8qbÜ?DºB¶té¸ÿ#ý5åÈ#r···×õÝ´i/~¬Àý÷§?ýIU£V6²öÍ7ß4~"wuuøáAAAÛ¶m»çÎSí[)^Çþ6²Y½n¹|ù²´|ôÑGüÇNVù2Fë3¡k8YøÍo~#-²<j²ÊÂsÏ=§¤bví,;vLoüå_Æp*?÷õ×_)IÇYÖ%ìíþyB©ËeËã=pàë³édÕ».îå7WíÜÁ¬ÀÌpª]ÆÝqªÂF²ÍX9©]ÁÒ®kÙ1.9=oÞ<ù ÷ôôÆ·ß~[íÕù¤¹»KV)ì¢òäää'OÞî!êç½òÊ+²°k×.õÛ?^¥¾úº~ýºÓ&Åîh§O>¬[zzz¤¥££Ãõ[Ó2ýÕ:YÕ×&ýÅHíIÖË$+HVà¼X]pdºMÅ*fÏ­îJêHýºaÃýé?F¸ïÉr\Ä,HáeÜFâÍ5üäH²:òZPíGåQ·6®óäöÒ¥KrûñÇ»îSU¹%«¾9hêíí-/^T­%ÈeaÇ£ìNíò£¥]ý&cü!òû¨åÛÅ¶NVõµIýþçÎS__._¾L²d©322UÓ]É9µøëo/ªOðÎÎN-Âé±ú]Þ|äÈ§m¶mÛæú@]:%ë£~+uô÷Ë/¿c Zª~J>Æ©©©êhåÕ«WuâÄùÔC$_eáõ×_W±ªö»ºþ!£þòDÖÊ¿`ÔB½üòËúIòòòÆNÖúúzÕ3ò 11Q[[[Û¬ YûL"Pnív»TêÐTiRzª±ú «ñø$®ä¨ú7o,;]	íÚµKÅäëáÃG¼ã¬>>>òHñgáÛ%ë³Ï>«?øàòKJ°Éò¨%lWWz*¹­©©Ñ_ëùÕqµüJÒøÂ/¨½©R£¿úê«rWõüùÒêÉÅ·+Y+á-¡¨ª£¿ª>W¥ëáê·ÞzKÕiµñÂõ²ü®Éª¥rUû·Æp¬ YE*5(iÔÕ*ÝÔ#ûãñB©Æà*Û8OU;u59n²»7XbIòÛjµªTG1Õx%ÉµßUíÖ_å7Ñ·ÓûºU¬	ToooùZ ~ü¸¶¶6µ ß<¢££eîÑ£GcbbTÚÉ­b-¨ÿ@õGéTÀò·ë¼ÿÚe§ú²eËFMVõÏU_wÔ7¤¤$Ù$+HVàA9vìñdV×d5hjcùèWTÕÑGù¤a¬z]ÏiµfÕÃ dyÍ5_sÒõTË	&«ÄI||¼qO¬ªÑY+9§ïJþIÕ®~m5XîÊ>°çTéÊ*i±ÙlºÒ_¢T¾[¨òë¯º wê§®õt¯¿Ù¡­÷îÝLVõçIeùKU½+¿¿JhU|«: YûC>è%-ô!zç¤ÞìtÊªíT²JnI¨Ý°j£1u8ÐiçäÉ*%ÓjÜÍÎ;ï¥fÕ:«2L×ÖR,:åªj×a£6¼T%¸Üª^ÒÃýuD"SÑTÇ2åÉõ¾ÜQµêúÃþÐxwÅNÛ¨ÝjT³S×¬úl(Eí]PÁ¬v¼S³dî¿Q?ôÇÞ¬Uí¶5þH¾¶¶6uTUíGëí1^²ªñý÷ß¿dUyµcY	TíúÔ¾hõsõù¬j¿®ú;::ÔØ`ðX2xÔRSSZFC§d«ìtÞ°ÞF~ÛQÓt"Åï½§Y+ÖÛ¬ Ya"Éª> ¯"¥Ê$K£rHãqV§¹¾þfª?ã(Ö$«ñ|5¥TäÆÔÎXµËWÝU'òê´:YÕÝ£GJ|FDD¬Y³FUÒêÜVUÞnÜï¨#GÝRË;ñ¨»»dU	­fÆpMdÉ<êÐàíUO÷#Ô<*huªñ«ÆÉÆ=Ç¡<ÍfSÏ/Á©··ÛíÏ=÷aiWiÕ b=ªVeúQj|¯Â×õ­:QÕ¸`kÖ¯¿ÒAM¯¨õøñãcüuj±Éï¦êu0uìTvê:ùò!Ý¦íÛ%«îai¯¨¨ YA²lèvÉzõêÕÎÎNwppPÂÆé3Ü9ôøa§=jìÓi£N*Ý)uÚ"%ïÒ¥KÇ8l"$ÚÚÚ¬V«ñwï·ðëoáv=l¥YÒôå_v½ÂÁ¨yì²®RpÍÑxéãI>N Y»÷ñÇLÓ·®¡¤7oÞ«¯¾zGWoÇp+ßW<==å¯¿Z<î|Ë·ûUG];ö÷¯¿ÛÅ$+$+ Y Y YÉÉÉHVHVHVHV@²@²@²oT7·ßüæ7ãn¦®g~ìØ1§Ç¾ÿþûç÷¼råÊD.¨¬À´®7ß|ÓØÒßß¿lDÎ79røðaÙrÛ¶mc<ÕÐÐP@@À5k^!9')**2nsñâÅ#ô3½,ÛËÃõÆ·nÝ:=PmÙÜÜ¬·´ÛíÒrùòeÝ2<<,-òkßi=z4ÇàÈ7Ô²ô<­ä¨±SSSÕ¯#¡¡¡¯¼ò/-¬ÀT¦ÓB1oänuuµk	¨/^<Árýúuy¶äädc²nØ°Á¸ä¥18%Fiy¸T²AMMÜ?^?°¾¾^Z.]º¤~¨üQ>>>gü±´>Z»cÇõ:¥õDÊ_ãÝO?ýTáäÉc|5klÁ«$+0é´Pa&Ù£Ãï>ÛãÇ»>ð.Õø<r733sLRupWWëZùUýüü¤HU[îß¿_µKµ¶¶·¼qãl°~ýzùTx«õ¾ò</½ôÒØDGGk)((àUÊzzzÒÒÒd¡¹¹Y>÷Ï;7vÍz/ÉêT³æççßnK)F%"##¯]»&å©··÷Ù³gBK~ÕÁÁAYøÓþ¤vJ÷÷÷KãÂåÜÜU?ú^ùëdYJÏ»è·]»vÉcm6ÛØÉêT³Êö?üáyÕd¦ú+ÞÍMrKnwìØqõêU½7Õ¬Rê-¡Ë/³Ù,áª2ª*óv?BVÕêëë6kkkÓ¤%ÃÃUûùóç¥Ý¸UîÊwÙF²êÕW_5Ùj7¯zYµ³gÏ~ûí·eùâÅÇjÁµDv2F©ß311QúGP:tèÉ¬Àß½òÊ+Æ@w­Y2N°fµÛí¹#^v¡²0::Zm)yyyú£ÆçWµ¬±U_¤ðÔ×Iæ4W%¨ÕjöÙg!zl,|ùåwÚW]]]òÀÞÞ^©uLª§Õu£ëÞàõë×ózÉLjPÚãzôèQ÷eo°ÊcUäÉääÜÛ&%%éQÁ:§ÍHù«ëlYkL²QOnñóó[¶ld¼ÃáÐÕè'ùcï¨fmmmU±êÚ6mUúN;$Õö_ìÆ?üHV`*ÂNeÜÓûxUJòR%«@$j­kçº¿T`c,î9×QQÆdGI¨ªp5IQßNrü½ê¯¸]Èsvvvïª½Ájÿ³,ËCÞ÷]§ê Y)EZêË/¿t?á~%ëÐÐ:Þ)Ù6nÍ:ÁdU»Õ96zÿªS²ª?mpp0  @C¢StóçÏßQGÉ/§ûÃu²ªRuìS~*/÷|rZUÅå'ÒÑÑ!í?T'´Â¸¸8§:Õicµ×io°Ýn×Û¨ACjRooïåË-[¦Î.5îÖÇYu*Þéº±UÅ¡¡¡ßP¾^äææJKaaáÉ'ïâø.@²qf"#)ÎÆÈu,¶¢¢bÜ:ÏxVÊÉ×M6IdJ©N¹ví1Y%V?U=jÉøäÁíÒÚiVÕè4	Ôòóóã|V5«¢üÆF)^ÕTMï½÷/?¬ddd$+$+$ëôÞïð3gÎ_þòÉ¬¿úÕ¯.]zGÀ÷¿ÿã)ì5Yÿéþé?üáIVHV@²¬ddd%Y$+ÉÉÉJ²HV@²¬¬$+d%Y$+ÉÀ¤NÖ®®®ÅÍæØØØææf@²Þ	ÔÃËüAAAjÁÉóÏ?O²HÖ;sôèÑ¸¸¸¯¾úÊm4Ë/ç	 Y'dxxØÏÏOâóàÁr×áâ¿ø5+d½3Ç=6ÇY$ëc6IVÉzO"##»ººd¡µµuÙ²e$+d½''OjuéÒ¥$+de¦ÉJ²@²@²¬ddd%Y$+É YIVHV@²¬ddd%Y$+ÉÉÉJ²HV@²¬¬¬$+d%Y Y YIVÉJ²HVd¬$+d%Yw«··7''Çf³&''·´´¬$+à.DEEåçç÷õõmß¾]"öÔ©S$+É¸7oÎÎÎ6¶;µ¬$+`¢²²²êêê-R°ÆÅÅ¬$+ànHyZYYilijj'YIVÀÝ¨¨¨Ð-ÅÅÅ$+É¸Kùùùááá¥¥¥åååIIIiiid%Yw¯¡¡aÓ¦M®S>VIVHV@²¬ddd%Y$+ÉÉÉJ²HV@²¬¬$+d½­­­.4Íqqq'O$YàÑ×ÙÙ¹yóæ×^íÏþ3ÉúÈ%kdd¤úý<M²À#.77×ÝÝ=44tÎ9²°råJõÑÝìãã#·O»LKKãÕßºÒÒRIÓòòru·±±Ñl6¯^½dµ­­mýúõ²ð¦øøx±±±+V¬0¶Ü',×vÉzíÚµ¬¬¬ë×¯³7e¡¡¡R¶[¤lõòòºÇ§íëëËÌÌÌÉÉ!Yïþþþ_~ypp±Áð[´hQrr²±%77788ø^³¬¬Ìf³EDDÔÖÖ¬÷Ýn_¶lÙ+W8ëR¡zxxlÜ¸QÝ-//wwwwªb'®½½=))Éd2LNxÔ5,,ÌÍdGÜ-[$M-¯¯¯,ÈGô]<ä¨¤©dª$kKKËäêfÜg£jD__ß]<¼ºº:**Êßß¿¬¬lhhhÒýù$+àQ!Iåææ&·ÝÝÝô¯ YíÛ·K²oß¾Iý¬oYBBªyyyw·d%Y`J©®®.***,,¬¬¬¼£:yÅbill½A²îDcFFäâöqqqrw%ÕÖÖFDDH¬LÆJ$+àþ+..NJJÒQ*©©©R¿ý¨5R)--íÔ©SS¬OHVÀÝXuÚk·ÛãããÇxHYYÙÔ©D²î¿§¢SîÆÅÅº±SiÊT"Y÷_nnî¦M-r7;;Ûi3Ã¡æTJ#HVÀý100°uëÖÌÌÌ¬¬¬·ÞzköìÙ7o!íNUìT©D²î©>ÒÒÒª«««ªªd9%%å§?ýiàiïèèÐOíJ$+à>(..ÎÈÈÐ¥§,H¸¹n9åG*¬ûÀõâ3R¹fff[¦ÉH%pHjÜß+êêê¤UËÓj¤É¸rssóóó-YYYjx°Ýn@>#HVÀÐÝÝ.áÚÞÞÞÒÒwúôé¼¼<)USSS§ÏH%p^íµÍ7wvvº«$«j|||QQÑ=BBB¬Vkiiét.UIVÀm%$$xxxÌ;788ØÝÝ]òuÔÍÎ9æææ1y/TN²¬ð`%''ëRµ¼¼uÿþýÆm¤6-)):Õf³UUUÑi$+à¶$V÷îÝklY°`AJJ¾«F*L¦zdE*T§ÜÜÜ§zJúúúÔH¥øøø¦¦&údoÆ¥¥¥Æ+VìÛ·J$+àmÜ¸QªÒuW>c===%-©D²îÒêÕ«ÝÝÝýüü¼½½=<<äJ$+àôõõ½ýöÛaaaT"Y÷Êáp[,F*¬»ñÙgIyêååeµZ%Mm6Óÿ¬$+Ü¥]»v¹¹¹=öØc«V­=¶,Íf¦ÿ%YIV¸K&)::º¼¼¨üwÞqww_»v-=C²îØ¤HU'ÕèKÐJÄÒ9$+àÎýò¿LuºPybbbPPýC²îDiTTÅbqwwÏÍÍ5®2L®tÉ¾¾>RYYYR¶._¾¼¿¿ÿO>ñóóóôô¼páE²ÆWYYéïïoµZgÌ!ëÏþsIS·²¶­­"YIVÇ©S§ÔÊ,XðÝï~WÏ³Ùed%Y`¢ÇöíÛ½¼¼üñ;wJUêtºjrrò¾ûè(dñ544<ñÄîîîV«5++ëûßÿ¾Édjii1nS\,+E²¬0ÞÞÞüü|77·   ½¿×××7   §§GoAÍJ²¬0Íæïï_VVh¼®jaa¡dmyy¹º[YY)[ª9"@²¬àìÔ©SéééRªfeeIa*nÜ`hh(..N*×ìììÔÔÔ.hC²¬0Ì«Õ*QZ[[ûÅÈElúûû%;],mß¾]ªUÙjd%Y`RwÆÄÄL¦¢¢¢èèh)[ÝÝÝåÖËËKV©Â¾ÅÅÅQQQN#YIVähAAdjBBB»´HÍêáá±wï^Ynkk1cÙl¶X,III²J6ã"q$+É£«®®°T#ÔÊ?ÿüs©Skjjô6ê6ï¾û®ÝnÊë¬$+B2233STÒío¿ý¶§§§ÓÆ~~~ééétÉJ²Àè¶oßn©d$Õª»»¿±Ñl6¯[·~#YIVpÖÒÒ/¥j~~¾Ód¿÷î['|R·¯]»V¶ÿì³Ïè=d¿***2LQQQÆUcÎ9RªúûûK¬Ê­,ËRÔÎ1Cbõ7Þ IÖ¿¹råJXXÉ`«®®Ð'Õ8­8öì#_ê©§äîóÏ?/kJJq4¦²677ÇÄÄÈ·-Ý²ß¼hÒÒÒø_ªº»»srr$S322fÒW$P¥BU±ªKX³Ù¬ÎºÉúV¬Xqþüy¬_õ¯yõ,_¾ÿ%)iÏ=6Íu¤&5S£¸¿úÕ¯è@utÆ½Á¦	=ýo~~¾0///88ØÛÛ;44´´´ToÙÙÙ)­ÓJµ;wî¤IVþ6ý¯©d·ÛUãÊ+===7nÜØØØëîî.Ëú!þþþ			ú®l 7îÉJ²¦$8ÕH¥ââb=RIªRÒúúz½Yyy¹´èÉôÿüç?ÍfÉ×ääd©h%÷ïßOgNdüÍo~£/^¬F!ÅÅÅÉ¨#G-dWùùùEªO5R©¡¡AZÒÓÓgÏí´½¯¯oUU¾áÂ©bW¬X!T«S-Yßï=µ/åK.ÉruuµÕj%YÀIeeeø5¯oIIÜ-++|]ºt©|2cÆc²b&k[[ü×U ª¬¬,¹Ý¿¿Ü^¾|Y%kPP3EvæÌ5Ri×®]R¡ÆÄÄüèG?9s¦¾"Mgg§l Qª)mÙ²Å¸7S3YoÝº¥êË¬/½ô´9rÄX³¬ HmZZZjµZÕH¥ÂÂBÉÔºº:ÉÚÕ«WK666êssså³ôg$''K	[TTDNñdÕnwìØ!òZILLÔÉ*õ«,Íæçd¦¦¦øøxù¨¤J´¥¥Åf³õööªµeeeRÈJâ2wîÜùóçÇÆÆ²xz%ë¨5+@rÓb±¤¦¦>õÔSRr¸»»'%%ém$hCBB$Dõð`)jNZÅ4­YUÊ¬ F*IFnß¾]jPu.ÍO~òù¨4îà-,,4L:sæiß¾t ÉúÿõÀÒÇÞ`Ó¤ºPyvvvooïÎ;å#QA"Õ×××xÅòªª*)[ÓÒÒbbbÒÓÓëêêèÃi¬'NP;~%Pe!??_åëØ<¨¶¡f0=I½/)*µirrò%KÛHêîîþ»ßýN2¸¼¼Õ8	Ó1YoÞ¼©òµ¾¾~xxX?¯äë/¼@²¤µX,Û·oß±cÉdPÇVÇMeY.]ªT§+°b:&«"Uív»,:tHn;::HVÓ$¥:V$ùÚÔÔ$ÁùÚk¯©µj®:½ýòåËé7õïúûûåU"Ù©[N<)-GåÞÞ^µ¯X×¯$+)¬¶¶VÓÀÀ@=§Ò+bccÛHy*K,ÉÍÍµÙlRÂ²ûdý]Ámmm®õ«ªn¹xñ¢ªbIVSXOO.//Ï8GÒ$A6ÍRRR$qW¯^Íd¿$ë£dðè(++ó÷÷QÕ§~~~n#ÜÝÝyyÑi$+É£hooOJJ²X,%%%j÷ï<<<|||jjjúûû¥0|T£$zgÌ±råJºdÇñãÇå5D²>ÔJ&)--MÏ¡/===%SuKtt´«Ô©R×J	Lï¬,âÝwßÕËË-Ë¡F0=zd0e¨J!!!®$I¸[ÔHO©V÷îÝ+-½G²ãðáÃYYYÕÕÕzÞàúúz¹LÕÉzòäIÀp»J_ìþüóÏç0¶òÉ'²=]G²ÞÁUää¹äEsöìYcýúÊ+¯ïêùHVÓH%m×®]&IYÛÊÊJ½ÖÏÏOEï¬w`Ôd6Ö¬zÖÀdä:RIûì³ÏÔ:¶ºnÝ:±R¹&&&Jâzzz¶µµÑ$ë'ëéÓ§åæåËåîÇLÍ`-..v©¤Í5ËØ%«´«c®[%Yï:°*®^½ªrtÅ²pãÆ)X_zé%99Àäb·Û¥fp©ÔÝÝ-´zxx¼ðÂNäÀ*Éz¯cÏ;'·ÃÃÃ¯¾úªÓª+W®pÖÉ¥¯¯oÕªUO?ý´Ó,ùQQQùùù²Ãá°X,RÑËÙd½ûdýòË/FäFjÖM6©Å$+ÉB*Ô3fxzz>ÿüóRëµ7oÎÎÎÖwÕPáÿøÇên¿Óþa¬wæúõëN'¶JÐ2SÉ¥»»;///66VÍç iªgôíéé	¯®®ÖQêtAò'xB>úÂÂÂä±RÅrld½§d½uëVìub«¼¼¼½½«G¨Ñq$+G{HHHrr²òi&)]½½½z²²2=±¾¬ÆjDSSÓÜ¹så±ëÖ­3NÀõ.©ëß®fu*jIVEKµ*	:44²iÓ&½ACCCZZZÞ³gOLLÌÀÀ^+«èFõþ$«ªVÕ©«úìUyu:µÀ#¥¦¦æ±ÇóððPs;¤§§÷ôô¨U6lÈÈÈÐçççë»R¿JÇÇÇËjÂ¬÷!YoÜ¸ÁµnL:¿ýío%MgÍ5sæLu`Õl6ë£v»ÝÏÏOO_WWÒÑÑa|ªªªÂú"ç Yïóü·nÝ©>åæúõërW^p$+GÉdòññÛ¢¢¢	W=¾$åüùó­VküÍ¦//Y%J[[[Éúà.-G²¸RîÝ»÷_þå_äjÁíííª½··×b±x×ÖÖJ1îp8ÚG°§ßN²ÊËôðáÃÇËÌÌ/wP1Éò| Ë»wï&Y|äISOOO5øãÝwß5®ñõõÍÈÈ15Ã·¬ªBíêêÒ]¯]»&-´Ô¬*VÓÒÒzzzÌf³ºfªZÛßß/k×®]KGáQIÖ;vÈkôÈ#²ìçç'»ví=6Ø8A?Éà!«­­à´Z­z´º@íºuëÒÓÓ=<<ì1:J²Þ¼yó¹ç×èåËÕnaùnè´£øÀ$+LâóñÇÔT»õI5ÊÏ~ö3///Åâãã³hÑ"¦wÀ#¬j?°Ü~úé§ê®ª_ç³rVÙÎ;ÕuÈgÍµpáBY=¶q,_¾Â#¬.]2^uÔë³x%S¥Z---U#.]*Eo¾ù¦Ú`óæÍ²Vg­Õ)>©Y|rssÕà¦¦&ã*Õ8wîÕ-[¶Ð]4ÉJÍà[QQQa³Ùüýý7mÚ$5«ÓÚ°°°yóæmÜ¸QVd0´´4ù´ÉÊÊ*++ûë_ÿ*ÉºnÝ:½:¯ÆØLîµ««ëÖ­[$+aëÖ­V«5**ÊÇÇÇízÉÔYe±XI¬Ð×duªYå¶¹¹dpµ´´$%%L¦ùY¹r¥´ÿñT'Ûèüýý¹9&S²Ë÷£>º¿)Éàvòóó%Sãââ$_¥EÇª&-²¾Âd­Y¹¦ºº:""Âjµnß¾]Í©$Eªä¨ÓfÞÞÞ²ÝÉ¬mmmÏÈQýµ¬¦æeÀ½ëîîNOOW§Ê²nÿë_ÿ*ÿó?ÿcÜØÓÓ3((NÃäKÖááa§ÀòúÞ±c5+ûHjÓÍ7«Juuuª±¿¿_¾¸GGG§¤¤¨«ëíW¯^-EEEEt&_²:dpµ´´ÄÅÅÉgK^^^__j¬©©ñðð0Lááá3fÌÐC¥Nõööåº$+Éà8©;ÕH%§É%VüqwÝºuòáããããééi±X¤f¥÷0õÖ­[òÞ°ak²Þ¼ydpw*++ÕH¥­[·:]V)OÎO-é7LÕ¦êÔ15I-¬îHoo¯|ÈHvv¶ÓÕßÞxãôôôÿøÇR:=*<<<::ÞÃÔLÖÙ|òäIÍf6ccc?N²ÓT¨ò<00pß¾Æö¶¶6IS)UýüüÔD¯¿þºqiv-Õ|QÝ¿¿,ìÞ½Í5$+0èJ¹¹¹N¥ª0LV«Uï»²å¶mÛTèJÜJî2!HÖQ©ÓxnÞ¼öÕW_Y]È;KSÃáØ¼y³ÅbdµÛíº½»»[JØù>--þ¹ñQjr`uëããóÙgÑ YGa6÷»HIIIKKã	LUUUáááòyÓ¦MêBåJ]]]HHH~~þ=%Aõ¬Ô©ò%ûO>¡TÅôJÖ¡¡¡;z*OOO½ìííÍÞ``ëííÍÍÍLMOOooo¦¦¦+V,X°@¾=ûúúVWW«-ÛÚÚä³EZä!ªE]î7Þ 1¥õØ±cjp||¼¬?úè#¹k,CÇ5ölu®ÜÊ2ÉLU!#t|îÜ¹ÓÃÃ#66VâvÎ9òé±ÿ~½ý¬Y³$J·oß.Ë.ì±Çä³jS-YoÜ¸qßÏ¨Y»v­ä±,Èíí&&YIMÊSu¡òììl]öõõyzz¾öÚkênmmmdd¤Þ?,!ª®§Æ.É*¬b&ëÐÜÜ*ïÍÖÚÚJ²SÌ¢EÜGH*Eªnå·»»Ûßß_â³¾¾^µHKT±¿þõ¯?ùäz$+3EÓ]CC©©©GY½zµ×¢¢¢¹sç·ù­¤655%$$ÐvÉ*ïgd`Ô××'¡(9*¥ªdª1;¥*UËRÊÚÎÎN½¶±±QZ¢¢¢,ÜºÎqLdçw-_~ù%ÉL[gÏì±ÇôhfÍå´ÄíÔrll¬¯¯ïÞ½e¹¼¼|Æ)))ô!¦W²ÚíöçF¨À7Obb¢¾zEE´È$Yi¨§§Çl6ËÀO~òîîî¤¤$	×_|ñvÉ*V­Z¥®'µ¬,ÓvÉzãÆãeÏ8ÉÞ``ûì³Ï$A||$Jÿûß«Æýû÷Ë]ãLúÆ½ÁÉ:úµä?ÞÛÛ+$+0ÕÔÔX,5ã 9s¦qí¢E¤ñ?þã?%z#u#¬B-=zTÏ=K²ÓÃáèììTgÔ,°½½=##Cóòòôfêh«··wpppii)]uôÂ£AAAj&ùÆªÃÕl6?d¦°3gÎ$''P£KJJÔ Þ¦¦&¹+zã%KOZHVgW¯^=tè¼OÚÚÚTË#G$PO>-ËEEErW¾Ê$+0e444lÚ´©°°p÷îÝÿú¯ÿn2üüü¼¼¼jkkõê5¨¹¹¹6MÖJÜÒ YGwåÊy©	;::$>/_¾,o¡?üÐ¸Ùðð05+0ÙI*oóöövYÈÏÏ/---//?þ3¥EuÅO>ùdBB~`MM§§gJJJllìêÕ«Ï=Kgd¤©Ýn?vìdªÃáVz%P%zeY¾Ò¬À¤¦®æ'éëëûøã«É~%h¥uww4P9*wõpß. Y'4p©¾¾^%«:¤*áªÚ%YO>-¯¼òÉLRgÎX­¬¬Tw/_.á*ïúââbÉ$«¤øÔÛK»¼ë"""¤Z[ã%Wuê0ª¼mÉªTTT¨±ÁÒ¾mÛ6D¥UÃfÌ±víZ½*+++33ÓjµúûûoÞ¼ù³Ï>???µvhhHÖÊö¥¥¥7nÎÉúõ]ïVgÝ¬±fÍõ¥³nIGíÑMII¹páB__ßìÙ³ånUUÕ#s*÷»ßw·$«¾úÛ?ÿó?Km»iÓ&©P322(RA²r­`º-,,LHH9sæ÷¿ÿ½*??ÿ'3gNYY§´³fÍúÎw¾#uÏ=ÒXYYY^^.uj]]æd½ÉzúôécÇéã¯&YI«QQQRt¶tþüùjDÒ#'Øøúúª9òòòþò¿¨Ñ¿6M25))Én·Ó YT²§a²$+ð*sÕªU«W¯NOOXÕíßûÞ÷JJJ¾Y©¨¨HÅê%K²²²$M·nÝJï#Yå«¼÷ÕÝóçÏ«ÁM$+ð©sæÌ1L-Z°`ºD^»råJÚÚÚooo¹+±Z=¢»»F²ªSWÆ?ómHVà®É7ÝöööIS)=%_Uûw¿û]ãì¾R¶ª:544TbUn<ÔdUóoØ°aÔ¸ÍÊÊ"Yo]YYY```RRRLL¤¦ñ3O>ùäÜ¹sõf³ÙËËë'¢VXj²^¿~]M¡/®/þþûï/]ºT|=yò¤ñJ®$+ð0UVVKµªîJ²Úl6Ú¯ÁÁÁ¥ªªjáÂêçÏ§ßo'YåMxîÜ9uVuwñâÅjUMMÜU§ºÊÛX2dyJêíííïï?sæÌ?þñzßSO=e´`Á«Õ*+ùºjÕ*º¾duÚ7ß|S-8p@î^»v½ÁÀC&Õ§Ää%KêëëËËËe900PïÔ---E1rð599YÞª^^^úêod½xñ¢ñdYaðð§¤¤è»ÑÑÑRÊG·üà?ð¡×<ýôÓ§N¢ßG.Yåýùì³ÏïÆÇÇ¬ÀÃ'yáÂ·¸¸xÖ¬Y±±±ênKK°o¿ývPPÐÌ3÷ìÙCb²?~ôÒ¥KÆdÍÌÌ$Yä×¿þµ»»»ÙlV»v5OOO§+¡Î?ßd2egg§§§ûùùÉ·^5t¿§§Ådýøãå]ª1;wNîVTT<¸iHV@bUÞbÑÑÑ÷î]·n©RêµºB²¾|ñÅgÌ¡.TN7b²^¾|9((¨¦¦ÆõÒ7ë×¯gvCà(5Imkk7Ýo¼¡îvvvJÎ;wË-ò~ñöö µÛí111R¹éi<¢5+×º¦Ï?ÿ´¿¿ßØøøã/X°@ßpMHH|é¥¤®Lööv: YIV`üd		1&«¶oß>Yåïï_VVÆI5À$KÖ7oÊ»]¾¬ÀPYY)éåå%1©.N®W©½Á¿þõ¯Û9s&--JÀ$NV5jéôéÓÆÆ/¿üdîÝÆåýþË_þrÑ¢Eê<ÔÛ`Ú´¤¤Äjµ2R	dÉj·ÛõtÁÕÕÕò>¯Ò²`=ØxÑV¸kR¤¦§§ë»jöPÉ¤ÎºY°`Þ9ÌH%`'ëðð°^>|ø°ËðüùóbFCÓ¦R:59XÍËËc¤0Õé7êú¬ôÑ.dÅ4töìYÕ7ß|ÓÓÓÓiUXXñ¤Õªªªððp«ÕÊH%`*$ë$Móóó«««½½½||ªGÈò²eËHVà.8¹sçº»»ûûû«	~å¦×~þùç²jÝºu²ÜÝÝ-¥jffæ3gè:`Ò'«ÓÁÔzÃ¸¸8¸R¨Iûúú¤7Ô3Ï<SYY¹víZ)aýüüúûû÷ìÙ#«BBBÊËË)U©¬Çmnn÷|WWk²>IùIVLaMMMòUJRãÜ¿RÂJåj6õ¥ÖÖÖ¤¤$)U©LdµÛíª$¸GÝÝÝòÊÉÉ	ªTZÈ;K]	NRVÒT25**JÞt0EõÊ+½½½:bIVà.H­)êÖ­[÷ïß?þü¢¢"µG·³³S*ÔyóæQ__ß;wJ9+jµZ·oßÎî_`ªÕ¬ÆâU'kXXXttôÙÒF²£êèè¯¡éééÅÅÅ^^^§OÖkýýý-Ë'|¢î®Òl6gffªë32R	âÉª.ÎzùòeY¶Ùl¬·nÝzçw¤q÷îÝ$+ I©¤677KéYVV¦Ú«ªª~úé]ªK¿yzzJúJÄÊòÌ3ÃÃÃ÷íÛG©Lýd5dTËê­$+ ´··KJ|æää|ç;ß¤ìííÕ«xâ	yûèc«©[·nýÅ/~ñ½ïO¾§öõõÑÀ´KVyÿË§s0NEjCCCHH.R<(ßAt6ölµû×ápäåå¥¤¤¼öÚkR°J×ÕÕÑÀ4MV®"hòu3(((88X"ÓX¤JÖê"UÌf³l,íK,ÇL&F*$+ÉêÔ¡¾¾¾]¤nÛ¶-&&&??_of,ReãÌÌÌÜÜÜÞÞ^iùÙÏ~&*H6ÓÉJ²bú@ÍÈÈ(Õ«ª««e.@Eª´K²J¾îÛ·Ïf³Y­VY ?dÅt/U²³³Õ8£ÂÂBÿ­[·êµqqq3gÎT»eôôtU¤ÚíöiOMMU*w#ÉJ²bÙ¾ZZ¤¦¼»"##uIZYY)-Æ"õÏþ³ÉdTT	Ý5l ,-RéJiK$ëvåÊ°°0¢Hº3))©±±ñÌ3+V¬°Z­MMMj­¤¦$å¬Y³¤UK1/we¾MKK¤²Åb)..fú_d½ÿcbbôsÄ.âãã¥Dào]EE¼p_)RåÅ)A«78qâ§§§¬¦[Ò1²C¸¨¨HªXIVF*$ë"ßúÏ?oLÖ§]¬ø]¸paË-¯½öZvvööíÛu»¡³gÏ²U·H >ÿüóv»]BWêTãÉ3ÕÕÕRÑúûûË3ãÉú@½Áx¤H¦º»»Ì;W^¨aaaÆÃþ k%qUýê:kÒ3g233¥TJÉJ²bZºSSªUu·¢¢ÂÃÃãÇ?þ±Þ`Ó¦M?ýéO¥ëÖ­µµµ®ÅhYYµR­ÊZú Y`*$+q+V¬5¶ÈKQ²V¢´¡¡!??_"SWrÕÞÞ®.T§/ Y©Y1­-X° 77×©QÍ^TT4êÅÝ¤rU#bbb$_éFd%Y1Õ××WUU9íÎõ©§2¶yyñ<µµµV«uëÖ­T¬ÌéhË-Å×××ÝÝãÆz:Î*e«J_ooï+Wú<===YYYò5199ùÔ©St,@²¬=<<tJI*QZZZª7Ø¿¿Éd:ÕÏÏOVÝ.VËÊÊÔJRøÒ«ÉJ²búZ´hÑ%K-R¡;m&ÕªDìATR5//$+Éé.44tË-NUìØGR5ãH%=Ç!dÅ´#eaaaBBB||ü¬Y³^xáãZ	ÚqDT*))áBåÉJ²búêîîDÌÍÍmiiéèèX²d»»ûï~÷;µöìÙ³eÕªUc<©Êô¿ÉJ²bºËal3gë¤õôôc^ßûöVVVRª$+É|!ñé4ÔóæÍ[½zµª;wî¼ÝÏ9f2rrrS	 YIVLwRÖÔÔTUUýà?hii1®»ñññc<VjÓ«ÕSWWGg$+Éé®¨¨ÈÃÃcÆ77·èèhãÜÌÌLÙàvµÛí¨RªnÞ¼«¿ Y¿Mþ wóþ×ýëÓO?]]]][[.ë¨ÓöõõåååI¦¦¥¥1§øÍæ4dég	ÍQRR2j¬êJöì¡¬ÀßY,ÆÆFcKii©Dæí¶W#Ômnw8$+Éé+88X_º½zudd¤ëz¤RDD#¬ÖZZZ¤ÐJ4$$$++ËxVLnn®ÙlÖ³Jýêéééµ_F*s¢*Ó=VýýýËËË%%SóòòãxW®îîn!/¾ø¢ñáÆJN'äÉé(55µ¬¬ÌØ,Akléìì|mÓÔùµµµRæê`¦3¬ÀNgJLý(F* YÑÙl6§£ñ=öÆÄÄ8øBÎüü|whh(""¢¶¶vÔ[ZZ-W@²WSS3wîÿ¥KÎ??;;»®®®²²2...//Ïõ!£êBåÌ©d%YñÝÝÝ,YR___^^òúë¯gdddff¹£QQQ²Ù¾ûè@$+É¢ïöõõÍ1C^'£nÜÝÝ-ëææ&¬ÓáX YÿÏÃÃãìÙ³ÆU«V-Z´ÈuK©Pm6#¬$+ÆIÖ.[V¯^í¬íííiiiT@²¬ßØ%G7oÞ,ÊH%$+É	qÁ$Y«&hhha¤dÅ©©©3g···Ô¯#²²²ÔJT@²¬¸'UUU6-<<üvDÉJ²bB:::222L&SQQÑÀÀd%Y1!/¾øbllì%KÔulJKK­VkBBB;]d%Y1Q^^^ÁÁÁ®)))îîîiiiñññþþþ£Î¸$+É±HêÓU¼ÜÜÜyæ®þd%YqÇúúú¤HUSCÔÖÖ3gÎªU«è$+É;&j2N:i±XÔH¥%K¬Î@²¬¸^^^z¤Ãá0Í[¶l¡g¬$+îLKKKRR···»»û¿ÿû¿K¦655Ùl6=é¬$+&d``   Àb±deeõôôúúúJ¾JýÉ,KHVw@Oÿ»gÏzÉJ²âîuwwKj2þÉJ²â^úûûëK²655IÖÒ3HVw¦££#55Õb±«é¬VkRRRxxxBBÂ3gè%$+ÉñII¦Jêé·nÝ§ªÕ¡¡!Û¨¨(f1@²¬GUU¤RîÛ·Ï!!!§N2n YIV®··7''G]¨ÜéHj__Ä­ÓöR¶ÐoHV£(++ºÝÊe­ÓlûYYYtdÅ?°Ûí			&©°°pI¤ÍÈÈÐìÛ·O²AÂHV§"I¦J²vtt½±dª$kHHHvv¶ÜÔÔD YÇ×ÚÚºpáB³ÙwòäIuªª­­¤¼£·´´HµÚØØ¨ÎÃu|ê÷;xð`tt´,ü_¿øÅ/HÖÉ«··7++ËÍÍ-339¬ÏW_å6åËó¿,9ZYY¹uëÖªª*Ã!þþþáááuuutõ¡jkk[¿~½,üÉÅóÏ?OÍ:)444HZ\¼xñbùªäéé©.TNç Yªk×®ÉÇñõë×9Î:yuwwVWWKæççL¦ÐÐÐ¹sç2qõÓûxÕÝþþþ_~ypp±ÁZEEEvvv]]]DDÕj---u8áááNS)Éú`ÙíöeË]¹r³n&»7ß|óÉ'/LéééúÄÓäädÎ@²>TaaaÆJ$ëd444T^^>sæL³Ùü?üA·÷ôôøûûsÉÊL¸v»=..N¾åçç/Y²$;;»··WÚO:¿iÓ&ºÉJ²bBúúúòòòL&$«Úå+å©$«:Í&00°¸¸áKHV²oß¾	ÑQçT²L@²¬´´477·ÔÔTÆý YIVÜ=öH©j³Ù*++©J¬$+î^GGGrr²ÉdÊÍÍe¸/dÅÝs8ÅÅÅV«5>>SdÅ=©««²X,ô÷¤··7''Çd2¥¥¥9swOTò÷÷¯¨¨Rµ»»»¥¥Ã«@²â:uJT××××ÑÑlµZ7oÞL YIVLÔ¦%<<¼±±ñYl6ÛöíÛÕÖ3gÎÄÄÄHK_ YIVC¢TTÒ%Ssrr544H¸Ò]HV·%inn®ÉdJOOïèè0®*((Ø³gÓö¾tdÅè***ýýý]TlÝºµ¨¨ÈØ"ÑA¿ YIV8;uêTjjªª999êºo®$G%wõìCCCôdÅßéJRÖÕÕ½qee¥knnnqqq\±Ãá ¬$+þ¦½½=>>^bµ¨¨hç§vwwWTTlÝºµ¶¶X#¡(i*ÀÕßd%Yïááá!!!öìaú_ YIÖRQQa2233ûúúè YIÖåp8ª««é YIVÉJ²HVd¬$+$+$+É YIVÉJ²@²@²¬ddd%Y$+É YIVHV@²¬ddd%Y$+ÉÉÉJ²HV@²>RÉÚÕÕµxñb³ÙÛÜÜL²HÖ"zøðaYß2((d¬÷ÇÑ£Gãââda´´4þuBýüüÜÜÜ<H²HÖûãØ±c³gÏfo0d½cnßpj7Í$+d½']]]²ÐÚÚºlÙ2@²Þ'OÆÆÆJµºtéÒÁÁA@²2Sd%Y Y YIVÉJ²@²@²¬d¬$+$+É YIVÉJ²@²@²ÞW^yÅ××7ò¾7o^$&Ü]sçÎ¥&èÉ'§&hþüùt×îN£&Þ]ò¼¿ÏiµZ'²^¼xñÿÜo?ùÉO$Yÿ&æç?ÿy@@ý0A¿úÕ¯ÜÜÜè	zýõ×¥»~ûÛßÒñÞïIw½ñÆtÅDìÝ»WºëßþíßîïÓþþ÷¿ÜÉú üçþgbbâ×]»vç;ß¡&¨¦¦FÞÌôÃµµµIwõ÷÷ÓÑ××'Ýõ¿ü®ÿýßÿî:|øðÃùq$+ÉJ²¬$+ÉJ²¬$+ÉJ²¬$+ÉJ²¬$+Éd%YIV¬$+ÉJ²¬$ëøío»zõj^vtàÀýèGôÃúé§QQQôÃ:uJºËápÐ188(ÝÕÕÕEWLÄ­[·¤»þû¿ÿd`ò!Y Y Y5'O´Ùlf³966öøñã¼FÕÚÚºpáBé¥¸¸8é1úm";¦.Ñ]c¸qãÆË/¿ìíí=oÞ<»ÝNwKz)&&FõOss3=v;W®ãÓþA÷ÛôMÖ¬¬¬ýû÷ËÂîÝ»×¬YÃkqTjVÌFGGÓoãï":Yé®1¼õÖ[Û¶m»uëFDDÝ5®   K.ÉÜªä ÇwùþaïÚKºßÜ¦ókTÞÒ²póæMã·Üý6®wÞyGMèÊËl.=wåÄÉ÷uVÜªï"ô«+V?Þ¬®½ô ûmú&«Ùlu£jkk[¿~=ý66©$âããå«ßÕt×ØïÁ;wÊ76	ÎÎNºkîWÜÊ[+ÛÉêÚKºß¦o²zzzêeooo^c¸víZVVÖõë×é·±eff8qÂø®¦»Æ~VTTÈÂ¹sçä	Ý5®Å«*_"VMqCM$Y]éA÷ÛôMÖÙ³gß¼ySíe^·ÓßßÿòË/Òoy3Ñ]ã¾êºkÜ*»duí¥ÝoÓ7Y×®]ûÑGÉÜJAÆqTv»Ù²eW®ßîî]Mw¡  àÐ¡C²ÐÕÕDwKêT5éÒéÓ§¥~¥Ç&¬®½ô ûmú&kssshh¨§§§Ífkmmå8ª°°0§"~»£w5Ý5«W¯fffJíþüyºk==¨Òcr+ËôØÕµt¿1S$+$+$+ Y Y YÉÉÉÉHVHV÷ïmìæ¶xñâ¬ÕÕÕ?ýéOcl¯®-3uýöW_UÝ½~ýºÜUS@²SÊ±cÇ2GHJÚuvvÊ­º$1ëëëuKLLDoÎ#G>|X6xöÙguË»ï¾+-W¯^Õinnõ555ô?@²SÍ7eeGGÜ¶µµ©ááaÀj³K.ÉÝË/ËíÒ¥KU£ñ»ª-**rjV)®ëà+VÈò| Ë»wïæ¬ÀT088¨Î=+	×ßß/·]]]GÃá¬6MS¯~~~R¡Ê¤±±±ª`Ý¿¿´>Ú©êçÔ)~íÚ5ãs Y©Cª¯òööVõ¥¤ ÚÇõêU§dÕ5¨º´Ú¬½quÖ¸ñ;¤QBWUïÚµKZTËÂÇÌ? Y©ãÊ+6l;xð F©8%ídAªL×d»'NS;uß~ûmi<wîè5kdÁøä7oÞ|î¹çÔdõØÌÌL§çá¿¬ÀR__¯©ã¬»wïõë×«d=zô¨ÚRâSÖªýÆê©>Èªö_¼xÑ)8¥ÛO?ýT»RªêáNªäå_¬À¢22++K_OO@	K)[eáÒ¥KºUÛ¨ã²GF¨1Jj!((HÂòÖ­[ò(YVÛëØµfU«¬À¡µ¶¶¶Êíûï¿¯ïwÞ7o¾ëp8ôCT¬VWW»>Õàà 6<<<ìÔ¬ÉL÷­[aa¡ZX¸p¡,:tÈÏÏO3<<£ ÊZ¹»víZÆõGP³$+0-¨¿CCC²|üøñèèhUJ»$«Ê<©>õ¡V5IÕ£r+©]¾R°ÞÔ¬ÉLjtqÌÑ~(-½½½_sFMrr²CãÞÝ«W¯ªFIS¬¸Ò"ñ|§5kWW×©¬&=á7$cccívû¸ª©©Qe®$¢l?oÞ¼´´4µÊápÈª×d=pà@Q¶wªYÕtüGv¢££÷ïß¯ïª©Þzë-ÝríÚ5§²UíLþè£(L¬¬¬¬ddd$+$+$+ Y Y Y YÀ]ù§éÛx©¬IEND®B`


反趋势正态 Q-Q 图


ZÄêêê¬¬,Ãñ¯ÿú¯æ»íííòÝø©2º¦8p@Ïô$W"©¶-VQQ!M=wî#Gä:[ZZXù(+e]$Ç«©©i¦ÙÙÙÙqqq?ûÙÏvíÚö8áÒÒRÔZçØæôôô8Nkný~¿üN£²RÖ5¡££###C¯111¿þõ¯µRGi¡|µ-,K^¿~Ý:G¦>Ï:çðáÃ¡L'?(ÃeÖ6ÊJY×·ÛmK¦D8`[,33óòåËÖ92ÞÍÏÏ·5ôé¼^/e@Y)ëo=é·Ó íÞ½Û¶XuuµPÍ2¨ã7~õ«_Y«)3Ø²RÖ5MÆmmmÖ9EEE555¶Å¤2lÍÍÍÿê¯þ*&&æõ×_ß»w¯Óº·ÕzZ¾ËÙPVÊº¶´¶¶:NäóàÁaúýíô[wjkkþó¿úê«RS=Qâàà Ëå²|äÈ	ðOúÓ×^M¾ÇáÁ(+e][dX$!fvv¶õô¡dZçHJek«µYeä*ÓêÙß)»lô£~Â¾n@Y)+XMMÍýÙÅÅÅ¹ÝîÐ­ÁÇã±ñ$ã]Z¶ÕÎÎN3Gú?°7aèGýÈÍóù|æ£~úúúd-3å»²Ây0²b±*++%¨zô¯1ô(_º¶¢¢B~ÜR_ÛOåççÛöæ.3ëGýÈW¹=¥¥¥¿ýá0FFFäoq¹jÊJY±(R±Ys¢'è·MM8eáÆÆF=N¸¡¡ÁvþaYÀëõÚ~JÆ/°¬¡õ#?PþÌÐ3`èëñ¨(+eÅÂ9sÆ¶£TÓÚÚvy¿ß/£Ò¤i2aëL[g:oâì:::ÊÊÊdÄ)cèOèGýüvúÓ~ää(+eÅüHK¬;JÌ©'©dÖ#6nÜøOÿôO¸aµµµ.ëðáÃÒ×êêj¹Îâ_Ú/ÃhëU¦u¨É0PVÊù@ÆÅÅY£%9e.æX©²´ùõ×_õÕWåªdtëv»ÍÇÁFBòSÖ¥äÐv^HèGý<xÐülUU4U.J°sssÍ|@Y)+>laYY4å«$íÌ3¼N°ÊuÖÔÔh·d,(<®G	=Êãñ,ì2<ÍÈÈ/))ÆK>u¿r0²Êºº:"KwïÞ½x ¬aÚ#®ÈØnaûDm*++%ÒÖ9R¯ÈwaÊÀÑzÈ±fè§DîòåËx©»mËpcc£T¼¢¢bñ¯'PVÊçE"j;§;===Âïììt:###f45..n¦»@Y)ë*'Ö#GXçHh³³³#¿@½^GêY%´¶+ÊJY×ËeAÑ§Çã©««×èYÝn÷ÿùïÚµKj][[ËPVÊºF<x0>>¾¼¼üoÿöoãââ^íµ7ß|³²²r¾[t%ÒòãzUII^³·(+^bÀþçNHHÈËËëëëùùù^¯Wßùòå9Rëke Î¼(+e]£dÌª'é5¤²¿üå/e8*å½4¡gåGÂ|(+e]ý222láÐZ?ýFÆ 2*iïikkkèÉ³²²lçNÊJY×PYm»Eÿèþ(77×:§¤¤d¦®±iBBu×ìàà o¿@Y)ëÚUUUetÄÆÆÚnhhØ½÷L× Ý5É.¡õz½|ÊJY×.I©´POñßÚÚép8nÜ¸a]¦²²òÀ3]C0¸ÆÇÇ»¿VWWsøÊJY×´ÑÑQ	§ÏçËÏÏ?|øðûï¿/ÓÒKýngg§ô2#¥©²d__ùq ¬u­Ó1hRRRQQQvv¶LØÎ8i°[=|ãââX(+eÅïÈÐ³¥¥åÌ3ÖÏ¢¿¿_,?¢zzNçÂ>±e¥¬Ào+**ª««­sdÌúí@Y)+"âóùl#ÔyxPVàÊÊÊlïÕ9sæÌ¼><(+ðdÀôâðð°×ë=|ø0ke¥¬X cÇI}>_QQ¾±u²RV,Êèèè¹sçZ[[9#?ÊJY²(+e²@Y)+²RVe¥¬PVÊ ¬põêU§Óép86oÞÜÕÕeýÖ¢,(+²Î­   ¹¹Y&êëë­ßjkkÛµkcVeÄÄÄ©©)HII±~K²ÚÞÞnS"55577»@YÇápiii9992ÓëõPVe[tt´	»Ìýû÷=[5<ëAI6lÐ­Á2=ÓÌ]Ê ¬?P\ÜÔÔ$òµ  À¶5xhhHÇ¬999@YçÖÝÝít:¯bµòµ··×ív;¬¬,+ePVÎ ¬Êe¥¬ÊJY²@Y)+²RVe¥¬PVÊ ¬@Y)+Êº ûöíûñ¶¤×þé¦a½1õ°~ö³ÉCõ°Ì^ýu^ÿéeãÆKqqq«¹¬CCCÿo©ýèG?*++ûXF¿øÅ/~þó³¼*:rä«b9ÉH ¤¤õ°rssåÕÌÒ^ç¿ýÛ¿¯Ú²>RÖÿøÿ`=,§÷ÞoçÎ¬åÔÙÙ)eýÿùVÅrJLLlhh`=,§üÇ|ûí·çwQVÊJY)+e¥¬²RVÊÊJYAY)+(+ee¥¬²RVÊÊJY)ëÙ´iÓý×±SUUÕßÿýß³SOOOzzúÿþïÿ²*Ó/~ñS§N±Ó¿üË¿SV^>ÊezÕét:Í7wuu±Bfll,%%eµ:Ëzdaî&+W®lÙ²EVÇãÃ:_7oÞÜºu«®îînÖùrºxñbTTÔÊ|z¡¬a477ËDaa!+däYÆívÇØµ:Ëzdaî&´´4=é'6mÚÄ:_ò´ÛÞÞ.²æYçËfrrR^Gg¶Ú)kò255%ÖQ"·ûö;wîXËºVgYÏ,ÌÝ4ØØXÖùrºpáÇãa/Cþùçæf¥­vÊÃá;y?¼,e]«³¬çHænIooï=XçË6xZ·n<ÔO8Á:_wïÞõz½R>ó³ÒV;e#::ÚLÇÄÄ°B¤¬¡kuõÉÂÜMa=zô¨  àñãÇ¬óåtñâÅ6°ÎG~~þ¥K¬Ï0+mµSÖ0ä?dbbB·è_ÖÐµ:Ëzdaî¦P÷ïßßµk×XçËOG6¬óåyb±Z«²Q\ÜÔÔ$òU^þ³B¤¬¡kuõÉÂÜM6~¿?''gllu¾lÒÒÒnÞ¼ùlúÀlYù¬óõ³ÒV;e£»»;999::ÚétÊ?+dIÊºVÃ®gýHæn²III±½g?oW¯^Ý¼y³V·mÛ¦Xç/äf¥­vÊE½ëuwµSV¬,ü1+uÎ:ÇK½Ú)+ÊeÊeÊe²xÙÿ¥£¢¶nÝZ0­­­íøñã2çÛo¿eùÌÌÌÙ¯S?bzÿþýzññãÇr±±±µPV`µ¹xñbþ4¨ÔîÚµkòU?ýÊZÁóçÏ9n·[Ò[2íìÙ³ííí²À;ÌÏ>ûLæ<|øÐüHww·Ì1ø¡×yúôiÖ?@YÕæéÓ§Öae ¯½½½:199©ëbwïÞ÷îÝ¯Û¶mÓÖtÕQlee¥mX+nÆÁÛ·oé¯¾úJ¦ëëë¹/Ê¬ú âÖ­[R¸û÷ïË×7o^¸pA&Á ­¬N§S¦Ú×uëÖÉU&dHºyóf°677Ë7nØF½r¦â=²^'Ê¬:T¾<y2&&FÇRAÝÆûðáC[YÍT?Z·ë÷Î;Ï¾ßMk]ø/¾]Öùå2GK,§Nâ(+°zíÝ»WwâÄ	M£8¥v2!£ÌÐ²ÊÅK.¥¤¤èFÝO>ùDf$''ÊõÊ'&&vîÜ©õgóóómëá^(+°ª?ÞR¤ûYëëëebÏ=ZÖ.èOù®n7Ö]¤f'«n²SFÀòõo¾1]ªÃtÈË]PV`UÑFhü5K¶ÊÄÝ»wÍTÑý²©©©g§é1J:(±i]ÞCÇ¬ú-X%tWë+WäëÑ£GMð:´qãFs1Ñ¬¶µµ^Õô°áÉÉÉÐ|2f(+°þ£¢***tbË-2qòäÉuëÖI8].YÆì@ïÊÅââbHk&ÓÒÒÂþÆ¬eÖÝð;>>.Ó]]]6mÒñ¨Ì²jódôivµêÑL:½ví|Åt¯Xg7cV²«d=æèë¯¿9ÃÃÃÏ¾GMVVæÐºu÷áÃ:SjjÊ*Å9çùYoÞ¼9KÊà¥aN@øôéS)âæÍý~ÿ?uúôiæJeù7æææê·Á |kýúõ¡e=~ü¸@Q·Y+**ôtÜ#eÖM6577zª¦?þØÌyôèmØªÊeÊe²Êe²Êe²@Ye²@Y@Äþ?òKKÁÜIEND®B`


Sï¾ûlñý÷ß×â=×<vìXLLÌòåË­:Ûµk»LNN¹G'ö©èèh%V¯^­t~~¾ej«TwuéþE¶øØcÝµâÎÈÈðä¬÷83§¾öûýV§ëU?V9ñññ<yRí%vïÞílMüqðÉÁò®;Gå9þüaT¡~BOOOðjj(1sæÌgÖ~Î@úÞ¹sçÞé[®]»¦õ×­[7xÃÂ8zô¨J§IäiÄ#-ü·¸CÖëêêÙ¾áFùÔ6M°Ê(¤#=+«¶uW Êyë­·lñÆØôâÅ§O¶:Î*>«¸EJ×ÖÖÔc;«%-öR¢¤¤d¸f½ÈÝ¿Âi.Ë/+góæÍÁ+KZ_:Ñ®ööö:¿rlÊô|äÄöK7nÜhqdAA^Í²C<RUzÎÀíáßu¤£tH³*±lÙ2K¨£¡ª´ôïî´HÀÓ°p¯Bpå($½«ìvBØ;ýmPq¹ÖÌËËsé¶mÛ·æÕùíNp¯=·|O#³ÂÃú!U=-^¼xåÊV?¾òÊ+PüÇpNÝªwìØaõ´áHÚãU¥uý)òD¨Ê¼zõªGrCÆ¬z½fÕ»¿úÕ¯æÌ£ÍFDDÌ7Oo¿ý¶õ:æ°0Ë¹Ú¯Óï² UmP1wp§|52¬ØUiÏg¨¯fÑ¬2½;R´LOO×ïôýûuj¬¼þúëJ¼ÿþû¶WÖÕ¯¶Ep¿ûò­¬`w<$îÖ[gg§rÚÚÚÛ:2þjÇ¬:ßì°:ù:²N³fÑÂ*¬÷Þ/då¡!k.§êôômº±pG*µÈUUÛ¡Càè*ØÒò;æV;f2C^ÂTåî¡´îÅ%KR;V¶`Ú~Úîu$÷§>úè#çjqKKöSÅÅÅ¶²;woÄcV»hðàÁÏåçô«7Øúí­5¸ÿÜ°êl8ÏõáËÞ¼eÝû8Õ°PÂ´ÜiGpÃ!Ï7§áå ¯üÒ¾ýí¥ï¤mçü´¶ý?ö¬5._¾Y³BXN;æYûØ8Ù³g¢;ÕÞ²0Å©7ÝÝÅ!±ÈéÕW_µú]±;f5SÆÇÇ[tèÞawa¸1«£¥ëU.÷¬óÎ;ï¸sdS3¥vÆ¤kÑù¾û<¢ìàëmûk×®Õ«Uú×®]³¯ðtÒG³âóùûNì]»$ìÏdamë×¯>¥FZ*7ûüjã°L«ÖïüCîÔï½råJ½»ûö¸ûÚ¿u¤nV+'Ê<zôèàç6f10«]wî½«Y-	äêÕ«6,Å*k»fiõ»;ìpw·Z0äQéÍ:cÆYSa»lV»^ïBæ)fªã¬lÝæúi¥¥¥ÎgÓmÈÝ[ºt©»q#Gj#[È _Ømí°shlQÈÜöì±A=¶¥zñÅ­7UQøc=f-kxÎ,Û¸;ÄFïJÞ¢ü¶Ó£ [#Ã9âÖ±Ò¶ò´ö3dÊg;¬æÃY³Â(bBr:Q=R<wî5Ï(n³z:i³ZâÈ#é¸U$¯ë-ÌýAÕ­Ë/·HÅö§¤¤ÄºàºÒÂwìpÍ:kÖ,ÏõTÛg'Î6«¾Tá¸lq×®]ÁZaÓ¦M¶k´ÇçÒÒÒämÑ f½ko°N­èèh³ ]Å´PXGÇú]­9åì¡~¯sp-v!ûo?¥Æ¾N?ÇsæÌ7o;ÌÝ¿¿¿M;xð û ;í³gÏz¾E°~»ì´§ë~ñâÅ!Íj==[CålÎÆ¬Ya±@Ê.òIKîªÐÉwâHçÝô)M.[¶ÌéÜ$ã¾ÏÄädÃJí	Us&!Õ¡!GÞêT'Z8hã»ìjÛñ)qÏB]guÇ¬Î­JKðNÄ3BÊîNqvåÈëÎ¥Aç#			Ö°¡³VàÖlÅ¬¡USÑéè¸b%$	Ì=jLï:·ÒíC^^¾B¦´ëâZTÂ¹°jMw¤k;ìóù>s] J:dGÁéo°>9c<wÙ¼æ³/:´ÍÜòhVû9BVÖ/µSûo¶à;dfûÈ5²³³XÁézõÔê]õÉ¢l7ä¼§Åc;vìØlAÝµÿ½ô#²1:2P1zîKqvÉ=FpÌêÏLòæáÆ¬Näg=Òî6kÏ¬v©Ø9¶²|i÷êÕÁ	Ö¥L»¢i×2µq§/7ä¥VçKÝ7nl÷ØÕÕì9@ÕÓ@±Ëá&fëÞ'fÌ£ª]gHªõZ¥Cõ£³ÁOT4YÙ[¶)z-1×Y-þ"«@-âtB¨leR÷nVæ/ºÍjewª(wZ!Í2fýìÙ6l#÷hVð,Ð·îkç8ª`ùÎìØÙ¯sîgÍÉÉ±Öý¶¶6+§«_4äåONÈy6ì^,÷Xe÷í1îu³ÂcÓ¡ô'¶åàÎÌF%ZuåTUª:­®·«eVíZ§ßÈÌêÜÏêîtÆy:fUøb®/²y,3gN~~¾r¾à[bkV÷uÖàºv'®g|©ÌªßewÜJBúQzµ.Gã°bVgíÌê¾Æ4ÜÕ)ëòµ±»6	s³ÚÝýû÷ë&&&ê¸X¼n÷¶Z,x§q¿!GöîºêFfV;mfÏl0+Â¹âªk«¢·#ô»±ÿÙó²bV÷ fV÷õZ7ëÜdbµ¹¢ç;ur60,ÈvÛlìú¤Õ]Y;s9sF»*¥y>"³Zä­udJYGb9§OºYMçÖùé±¥¥Å®dÛ·;óUÍ1Ã¹¾ka³o6¾×¦ð>¬Î¸îàÞî5//ï³/¦t°ù°³ºepÞ)ævc7» ]LÜÊ¤&Ý@ìórªg»ápÃ¬YaÔ±0Ë!;;[¯ïTÇIQVé»G0-Y²ÄI;z°NÅ'OÚ¢õy:²`B	Ùmh®,âo,u)°ó!xÔ®Sÿ*&Ó.Ù$Áa±e'öµ1SßÐy§A7t®ZµÊâ`ÏGdVÊèÆÇÇ´kÛv½ÓÊ3¡Òp±_mèkoCþröÍ"dN¸»ÔÚäJ°ý÷B¢ö¨³(*Ð·Nò»ú8Ø²ò¥s¹×ÓdôÌÈo³ºoòä?f1vU=Ypv×­Õ*®9¬+W®(6þùç¥êsJXEï³ÁÏrqßôb®ï±l±Ñ³ëÁNdoû¬°Õï÷æ6SïÏauB:5ªBNl9öp«<'¥-fy»òÝ+WÞu»0+fÀ¬0+fÀ¬Y0+fÌY0+fÌY0+ÀÿÃLö«_ýÊsåÊÅäÁÞ½÷ìÙ£5ßyçA6ÕÛÛ;kÖ¬åË¿:Àô÷:.³eá¤µ¾>îì1cÆíÏÕ«WÝùõ«²«YîÈþýûÝö&Ýf­¯¯÷¬ß××gÙ³g/pßòé§jkééén]­ZµÊ½NÿíÛ·-­VÈÈÈ¸ò_jÿ~ñÅï´ÂpÊ®f¸#)Mf			rc>ø@¯þàÌêÞsrr	->úôàfµÝº¿fú®`VøÿèììÌÌÌTâÈ#RÈÙ³gYïÅ¬@°°°ðNkîÞ½[1ôÜ¹soÜ¸±iÓ&óÌ3uÆ£³ÜUÀ¬wå-½nØ°áúõëJxÑcÖþþþ8]Ç«]µäAd¦Ínß¾Ý³oß¾ÞÞ^ÏjÇsz¤%W¿ßoùçÏW~bbb°8wíÚ¥tnnî fÖÅ×!î*`VÁxýõ×ÝBMII	Y~ãaÅ¬---ù¼óæÍ³5(((p>(º·o¡dHSæääôôôÜÌ:ô]Ìp­ZäúÙc¬õÞÍÇ*!EEGG²~|||ZZ3*Ø	õÁ`q~üñÇQQQÏ>û¬¼ï1ëpw0+@h. lÜ¸Q¯îÀô>^gÕ¦äKÓÒxûí·Má5þùà»hÝj|÷Ýw^½zõ½Ç¬ÃÝUÀ¬!¸uëÑÙÙyóæM%6mÚtßÍÚÛÛkòSdy×@p¸fíæFDD¨pïfÖ®fm²ûöyÒóçÏ·íÐ¡CÁikkS¾ê»~ZW:ü[ZZî$N[-[vïfÖ®fðrùòåù%%%R"Ú+ØµØÚÚÚÁ·oÃ>þøc'G¢´JKK¥Ì½»`Á7n¸Ý,<÷FFì*`VÌY0+`VÌYÇ÷Þ/>>>`,øÚ×¾öÿùÊ¬ù¹hÑ¢³cÁ3Ï<ò¶òmÖ?ýÓ?ý`,xþùç1+fÅ¬Y1+`VÌY1+`VÌ³`VÌY³bVÀ¬³bVÀ¬0+fÀ¬³fÅ¬Y1+fÅ¬Y1+`VÌY1+`VÌ³`VÌY³bVÀ¬³bVÀ¬0+fÀ¬³fÅ¬³bVÀ¬0+fÀ¬0+fÌY0+fÅ¬Y1+`VÌY1+`VÌ³îîîòòòÜÜÜªªªÞÞ^Î3ÌYGHWWWRRÒ+ëêê@AAçfÅ¬#$//¯°°ÐYTÀØÐÐÀ©Y1ëHðù|[Ý9ÅÅÅj³¸¸8ÏÕêêê¢¢"N5ÌYGBFFTêÎIOO÷äfÅ¬C¥µµ5&&F*UäÚÕÕUPPÚÓÓÃ©Y1ëÈåªÈU~ÍÍÍõÌY&¸Y¯]»âÄ	Ï9þübVÀ¬wçÈ#ÉÉÉÓ¦ØÜÜÜ­[·*±qãÆåËcVÀ¬wgÉ%çÏiÖÙ³gß¾[¾¾>jg1úô^xCõÿßPfô¤?"===33CõîfpÒQQQôf½'³>öØcÖ¬4fÌzOf]¹råæÍÐknn.fÌ:B³Úâ#Gâãã#""|>ßÑ£G1+`VfÌY0+fÅ¬Y1+fÀ¬0+fÌY0+fÌY³bVÌY1+`VÌ³`VÌ³fÅ¬³bVÀ¬³`VÌ³fÅ¬³fÅ¬Y1+fÀ¬0+fÌY0+fÌY³bVÌY³NF³öôôtwws`VÌz¯´µµ¥§§ÇÆÆú|>¿ß___ÏÙY1ëéêê«¨¨PÌªÅÆÆF)¶¡¡³bÖPZZZPPàÎÙ¹sgjj*'fÅ¬#!;;[qª'3::³bÖ_[[ëÎéììôù|p³;w&%%uuu99yyyÒ-'fÅ¬#¤¨¨Èï÷WTTTWW§§§§¦¦^ºt³bÖÓÔÔTZZ*ÅJ®6H0+fÀ¬0+fÌY0+fÅ¬Y1+`VÌY1+`VÌ³`VÌY³bVÀ¬³bVJfÝ±cÇ´iÓúûû1+`Öû`Ö9sæ|ëöíÛ0ëðX½zõ3^ýõõõõ7oV,[ZZY³ésáÂÌzßÌºhÑ"Ìõ>õæÍÓ¨¯¯÷ô¿÷ÞÊ?ö,fÌ:vïÞÝ××¿gÏµ««kªõÜ¹s)))©©©%%%ÝÝÝyõ°!Áv7Nooï2kGGG\kkkNN0ëÝ9pà@ÎNà·ÞzËBU½¦§§Oµë¬ÙÙÙkÖ¬qçdeesòÀdB|CCêüÎÎNÌzßÌzëÖ­»lfUÀêPuò©ýÁÿ&ë×¯µà*::º¦¦³ivCµ°°põÒ¥Kîµé0+LT§ùýþööv[lkkóù|---u´Ìzùòå¹sçîÚµkõêÕ2ëÉ'§`opii©;'33Þ`4deemÙ²ÅSQQQPPYG1fíïïW¨*­:thÞÏzîÜ¹¤¤¤¼¼¼ÆÆÆ;w¦¥¥É¬`IC èèèpç455©¢Ã¬<ëfG×¬Y£à5''§ªªª··¿"L¦µ®®ÎSYYIÌz?ÍzìØ±¥8cKoÛ¶MÁ«ÒÌ09à:ë¨µ¿¿ßó@©tÃÌn0YY¿~LL­®®Ê¥Þ`Ì0ééêê²ÊsçÎMñ¢À¬ãÞ¬·oßMW­ZlÖó	cVÀ¬ÃS±LÆ¬YïÉ¬ôfÅ¬³fÅ¬YGnÖ©öäsÀ¬#7«=üÜÆ§¤¤8ãôæÍµY³ÞÓgÝ`VÉgÖiÓ¦-]º³f½of]·n;çæÍSÙ¬çÎ«¯¯ohhèêêâäÀ¬w¡¥¥eÙ6xûöí2ë³Ï>ë<½¶¶V9úÊ©iÖØØØììì-[¶pþ`Ö»v?ó|Û¶m³gÏHèeeeÉÉÉN¨ÚÚÚ*ËªµÁ)Yñ ¹Ê%JzuUZ=|ø°;§²²2??S³õ«°ôþýû>sæÌT6kLL'§©©)33S³Þ½OX=¶Í¾´÷nG®çÏfMIIJÝ9eeeõ~Ç³fÍ:vìåìÝ»WB=uêÒW¯^-))ÑbTTÖd;'Nðù|Òðüùó<è~Kæbµ®®Îï÷·µµÙ¢±±±Î"`V/×®]9s¦Ín(aH/_ü>üðC÷jýýýwÝTnnîÖ­[Ø¸qãòåËÝoIH¯¾úª;çr?ùÉOÆçØàªªªèèèÔÔÔ@  ¦'Ì­¥¥åÀrjOOBX%ººº$T©WiÉò®=¶1îëëKHHp¿%­îÙ³ÇYüýï?-/¼ðÂø,nIkkëñãÇ9ù0ë.íÛ·ÏÌjT%WËYO:¥Äë¯¿>øFÜOÂñ<gîÜ¹/VfJJÊÙ³gs<ýèGÌÁÁ¬vUaÛ¬Fmm­Vþ;ï¼3øv"""tTTTÈu®Ý&yÌêô	[¯ìÊ/_®ô¬Y³øñÇ¬¯¯Ïz¾Ójw.fÉfÖD2Þ¼y³zÍÍÍõô_¸pÁbÖÅcVÀ¬wçÈ#ñññ>ïèÑ£Î;v,999222==]rÅ¬0UÌzêÔ©8Rtèå)rYïivC%ËbVÀ¬#ylzäÈ[<þ¼Æ¬YÍá¹µf(·±bVÀ¬¡§ã_µjUHÝzúbVÀ¬ñé§ÚLËÈýúúúM6-Z´È.¾8qÂýtÌõ£lÆA§.íÞ½[6Ïç1+`ÖaöW¿ú¥·mÛ¦Å7nÐÜÓÓsüøñ¶¶6&åÀ¬ÃàÂîmNNNfSmmmLLLJJÀ¬ÃX.]ê^N¦¸Y[ZZbccm`Ö¡rðàA©ôâÅn³æääLq³ªjjjÜ9eeeu0víÚ%Ú ¦³gÏj±¶¶6Ó0[³wNSSSff&g!ÀT£®®.%%%:::11±¤¤¤§§2Á¬w|xÜìÙ³wïÞüD¹×^Ù³²²Ý9ÕÕÕùùùSªª*¿ßoÃ,ÚÚÚÔ¼ÎÎÎ¦X0ë¸fÜµ¦¦&99¹««ËÏ;çóùêëë9¦OcbbÜ,£ÈÕÓìÌzGúúú°¾òÊ+Õ(((+...**Ò¿«¢¢S`J!§¦¥¥y2KKK+++)Ì:$ìë©S§Ü7oÞÊ÷³677ë/´~ýúãÇsþL5ÚÛÛ'SMmÕ	f½ãSn©ëëë¥UEfJ¸g:t?`9`ªá÷ûëêêÅsçÎ©¤©YïH¿Þ³gÍ»tþüùðÌ¾Y`üÓÜÜ[VVÖÔÔTUUåóùÊËË)Ì:ÔÙùíYr7oÛÔÆ?EEEùùùcÄ¬CeÕªU²iaa¡N¨¨¨3fÔ ôâÅ1+`Öaà¹83S À¬Y1µ¡<zäÈIôôéÓÁfí	1+L³¶´´XHªf¸wée`*õÚµk:ÅbVÓÝÝç÷ûcbbÖ¬YCLÑÕ¼:fMHH7oÞY/]ºÄùwª"RSSW¬XaE»b5ÕÍj»|ù²Ò>Of½ûöºuë¹qãÆ©lVýOrrr¬ª©®®æ,õõõ@@~ur:;;£££éÒfu#Ì;×Òöt¹©lÖ§ÚÚÚ*¿"WðPYYYZZêÉLOOoii¡p0ëç×VóòòI466zÚ¡«Zà¦®®NMpwê5ÄÏ;Gá`V"w÷v¨þ-ôðÕ	qqqîéÊÊÊRSSÝírZfUï,<yR9---µ¶¶Vá»;§§§'66V¯Ã+¦Ôë¨zÎ¦Vä*§¦§§'%%µ··SøSÔ¬W®G;æí¨>Üfµ³³3&&F'§¨¨(++`BpéÒ¥´´´ÄÄD5j÷¬"×-[¶TTTHäÝÝÝÿT4k___ii©Ì¡sÎ&8Ü¾»=ü<!!Áy;#UDÖMIIÑ¿®`bGýyPµ¼¼Åy0f½û¶Ä¹cÇ'TÕ¦õúÊ+¯¸ã×_|qßÏªÈµ¶¶VíÐú&víÓãQ­î^(Oîo°Å©NzïÞ½Î]­Næ)&­­­Y=yyy[¶l¡p |f3gÎµk×¸uëùþûïcVpt)66Ö=ZooobbâáÃ)]³Î;W8VõjñññÎ¿ùÍo0+LD322L®===ZäNkÌjíììTúèÑ£¹nÝ:ÌI4777&&&===...++06fýýýNæï½Y`â"¶´´(f (`,Íê^áÝwßÅ¬YGruÏ=Á·å060ëïgÝ´iuÇÇÇoÝºuÕªUp¦²YÛÛÛ?Îý¬õÈ ç3glñæÍîw?üðCw_ñT6kss³ßïiiiÑÑÑë×¯çDÀ¬<ëf(N©««³Å¶¶¶ÄÄDäY1ë)...++sç455%%%q.`ÖÁÆ1½ùæî.âÑ¾Óf5;;[*õdN>s Ì´··×ÕÕéÿÈú0ÞÍjÓ¾óÎ;NÎ©S§<ÏÊf-,,¬¨¨pç´¶¶³¼¼<Ï·bÅ¸¸¸àö.À82«Ýx£DDDÄâÅíE½úê«v+Î#G¦²YåQý[ZZl±««+55ÕãZUòóó%T'Tmhhå¹â0NÍj7ÞlÛ¶Í»lÙ²`ï2op]]þÆÙÙÙj/+QTTÄ¼£áDÿ;ÏËõ7f%ãÑ¬;vìp®ªN@qjÞ¸§éâ÷³ª±ÜØØ¸sçNÉaæÒ¥K~¿ßYSSSPP@áÀ¸3«ÖåËÛÏÍ¬¥¥¥uFûrÌÁ0¡±FgGGÇèK\§Q«¦?e`<U*=ö¬^·nÝjöìqÂV%6oÞ¬üë×¯cVð §ÆÄÄddd¬X±BâââQºP"§C¸ººÚçóñÔwf½råßïw_IµÞà½Úð¥´´´hñüùóÜÈs±±±Vmq´÷EGG«RJLLLJJjmmåÀxY=c1ë0+Àx£¦¦FÁbNN¬c÷'¨çJçáÃGõ´îînE;CaõØ±cgÏýõ×gÌñÖ[o)'>>~õêÕ3gÎ|óÍ7éJcµµµò«ßïóóG¤vOæp'Q#õJÒú!<?&YõÚÖÖ¦×ûö³s¤R÷ÆâââÜÜÜ0Ç¬²»;g¸¨H¥@ ==]1·v>&&F­.L*³^¼xQ¯j?bVqNiiiee¥;çÒ¥K±±±áÜ!^gíééõeS+4448oÉ©%%%Î¢"rIbÖäädýÖÿdÖ¬Y7nÔÿdóæÍ:ïyëÖ­)nVªËTAÔÔÔpÆý1«ªªÜ9:-¥¥0ïglpÈIT²²²dP»gFÕd[k&VØíY_Û±w&¼YYvßÀÚ××çÜ3ÍªúBUBêêjÕjw´éGç¡[K:9ÓÒÒÂ¿'O¢ÒÜÜ¨»[Æ>ï®ãà.+++//çøÂ7«ñÁx¦àwæâ3E¨ÊHJJr_ÐÊËËËÏÏç1DNrrrÎ;§´´ªÑÝzü öhii©'ÓnEUó4¸ïW?Êy2À7ëÍ7¥ÒW^yÅ?ýôS-~øáöÖT6«$êéR]`n1Df²Ç¿ÈOÒ8ÔªÅÖf¨â×ØØXb´çìêçw0!ÍÚßß¿p½÷ê/úÚk¯í@Z]¹r¥[·nUZß:Ï<^1::sÆE~³²²V¬X±eËq¸6ÊéðáÃNv8##ÃY,//×~¿_¯ÁV	&IÌzûöm·håÑ>øà³02ÎÍªÀskAjj*ç"-mmmqqqÒjrr²çD'hßUëTSS#w&%%yZcý@IeÖk×®-@êºuëdÖüü|4ØÖµk×Ne³ª­ú«¢¢ÂþùMMMjïÒÓÓÝ·¸t)11qLÎLµ5KJJït´µµuÍ5UU£ÿ`JÕÆ;£,YâyWFâc¨_ìÜ¹Æ8¯²²Rzó(U¸¼~Å£jÂT7ë¡Cà<UÁ«ûù¬Û¶m³gà0Sê2Û0NèîîV#Ïã°êêê¢¢¢pîæ9R"pC*Lu³:Ñª¬.ü,ì0ÀHLLt÷¾Ú8k8÷!##£¹¹Ù³eË+Vpt`JÕáÂ2ëÑ£G1ë ÔÔÔdee%''ggg755q:ÂÒØØW^^ÞÞÞÞÒÒ¢33333Ì·¬(BõL¡ÿö£õó»W÷ïßï<óÜ=iÇõÏåTUgbcc¹¥Æ¶¶¶ÜÜÏ<óÌßýÝßÿNP§eeeî÷TÀSÔ¬¦ÕÍ7ß@9axÚù3kkk«Mãä(JpnxÃ~X§³Q1kïµ;***ºPí÷ûoªZf$QÏ=fíïïÇ¬UUUÁcCRRR?ÎI	cEee¥Lfó0è¿lsÝãÐµ9ÄítttdggÇ µ=`õÖ­[2h__¶¸téRgðÚµk_PPà5ø~ÔÔTÌcgz#kíë"ôéñ®óLKKKNNVìÙ8fÞîÁLkkk\½Áp¸G¨G¡¡g#2eÄàù«þ¬ÎÎÎYPÛ©®®Ö.Ù[¶lñÀ¬8IÕ¢ª¤ñ9S+[lÜ5kÖHc:"##~úiE111#§Öçë!>ßTROJJÒ)mWÃÑ?gì¼¼¼à'`VÌz?Q©:1+++x~A8|ø°D¨fÙ'_<½=Æú?F0·Ì':!oss³$r2eÜÊÊÊêçÞÉõË_þ²'zV²  c³GÖ`iYMÒr?Pj´wÝÝÝééé@ ´´T9o°b[.++Söóÿ<xN	ÅÐrçh³Î>`VÌ0¾(**rd&Zðê<PIII#Û²lZQQQUUr[[g ì¹µ§§gÆª_¡Ú>¨Xc³Gjkk_:1«âÈ-[¶<ûì³Òmaaá~ô£?PòÓ²³³õüüsIW¡§;çÒ¥K18LüßýîwsssíÉ²¾"i`VÌ0NQ,¢xñÜ¹s»víR8øðO>ùdZZÚÛo¿ýè£>üðÃóçÏW4¡t¡>ôÙÔÔd#¤<ónºce¿ßo7Ø¸»mpssseeåúõë¹£0+f½½½3KJJxD+#kÖ××ëÜy³EL"T¼8gÎÈÈH¥øÃÊp¯¾úêÌ3%×­[·666­y×¯ëîîÖgÝqª>ëyé"ßºyµkµÃÜ?³ªÅÒP Ê(11±°°GQBF)3''';;[»Þõõ¯½¦¦F&át:IrÚõÜÚ½ªw®ô]LYÙnÚ¦äW;W;;;¡2.	³Ti:*UÌ£(Á ý~¿3È¦	¾êéFöµÓÉyØªD«æ½[RRRYY9ø*ÖÌÍÍõdªñçvÔÖÖ&ÝJÞéééúRm!f¨bòÜ)È£(§82bEEÚ[ÒÛþánÝºÕý®¼8øÍ R Åµ===Ò°l§Ô¹ÂªÍÁw@'dtt´»çYjWv,xåÃ|³bÖ±Á0xåTF:LMMÍÌÌT´ºsçÎzè»ßý®ûÑé1ø-ªZ!66Öº|õÇÎÈÈHJJ2óI:ß2Hhrr²ÝT£Yé»FºYÇ0<7!ð(Ê©LYYBLG¥ßûÞ÷xâ	÷Þòòò»N`¤UÑªÏçäG"##òH´ÕÕÕÊâô¿QP§O.1£UÌ:ÌÚÜÜ¬JPrUeÚÕÕUZZÊ£(§2ËôQ­¶(AÞõ:«N'EÀEEEsóóóµ5J³N»nTfddÄÄÄÄÆÆææævvvrFNYRSSÝ3-|2Ð1ñøãëôÐI²k×®°íÂV)9è´ä>TÌÊL0!)À³hÑ¢|Pnë­·^~ùeÏ©srr@777+ê-//W¬ìQ>`V	À¹sçü~¿äªQý³?û³ÈÈH÷ðàêêê¤¤¤Ñ¾¿eçÎÉÉÉîy*++G0¹?fÅ¬cIMMÍ+(y	SM®2k HIIyé¥¾óïxVYCÎ)))ñÜ#ËÆÆÆrt0ë1«ª@¡±±QiÕªÜ ;ßÜî~ÈÌ(Õ3Ø¦<ä`ÖaVE«Òª[¥ªL¹ñ>¢!..ÎntvvÆÄÄvÃKFOLLtKyy9½ÁuÂUUEæÎimmMIIá¼Oa7bI´:1<o%l¢Í¦¦¦ÂÂB÷$YÇ;¹¹¹GÜ¨ðÉÀNÉUqªÜ[VV¶éyëêêôÕYYY%%%öLuÀ¬Ã¬kÖ¬ñLg¨©ÁCWWSÞ`VÌ:$º»»SSS¹¶¶¶*Z---MNN&DÀ¬uäôôô¥¥¥E«h³bVÌ0uÌzâÄ	Ï9þüñ­	mÖÆÆÆz0ëý'77wëÖ­JlÜ¸qùòå¿ÕÄ%Kyæ¦	BCCC HLLrå÷¾÷½èèè7Þx£	&#Û¶m«®®Ö§(&7,wf=öíÛ·èëëKHHä­ßÿþ÷ÓÆ111ãÎ¬!Ó!ßêâµ×^@½ÁqqqYøKKK×¬YCwÊ¤AÇ×çó­_¿ÞîIíèè°(zÃGDDâ[ñ:«Õ¹ÌNÍIÀ¹sçZ[[ßyç¼¼<w~SSäJù`ÖðñØc)ô´xTé!¾5AG0)fõ<ÎzÅÀ£­­-%%%)))==]MÀeËyV>:¥YÃÇÊ+7oÞ¬^sssøÖ5«$ªú×äÚÓÓS^^ÈSZ'z¨ëtÿþÍßüÝ­%yWGÀ¬áãÈ#ñññ>ïèÑ£ïÐ´iwzk<ù«~ê_çÙ&0q©¨¨pwÿ¶··+BMHH°ÅîîîÌÌL.¥`Vfuººº³rFNÔZrç455éK_R³IÆUJ¯Ì°Y1+À0bÖÂÂBwNkkëÜ¹så×ººº¶¶6³bÖpÐÞÞ~üøqÂÖI]g­¯¯·ÅîîîôôtéÀ¬5L477ûýþ@ íéH©ÏçsºóóóéþÀ¬5L´¶¶ÆÄÄÔÕÕ9ªwîÜÉ©9Ñééé±îßöövJ³bÖðQXXXVVæÎQÌÉ©	Y1ëHÈÎÎVdãÎéèèHJJâÔÀ¬u1«glËÎ;õøñãuuuÍÍÍltuuééê¨q³Z[[ãââZZZl±³³399Yuoo¯ÂY½µbÅ´´4¿ßïÆ¦¦& ÜÜÜ²²²ôôô¤¤$.©fÈ£±±±ßÿþ÷î¹3fÈ£ÅÅÅÝÝÝ¶NMMÏçsaÌñÜZ#äWÉ1ÀYÇRæ3Ï<ðæoVVV&&&N>½££Ã½BØ-[¶pÊjkkW¬XáÎSý~?a+`ÖqlªPÕ¹PwæÌÈÈHÏ#<sÊCVZZêÉLOO?|ø0Y1ëØá!üÐCýà?pçdff³»ï·³³3&&À¬ãÕÑ^ÄåËÏ5KµUÙñÏÓåÂI÷¬ uÅ]]]LNÅfÅ¬c*hÏ½7ÅÅÅüÇõðÃ?ðÀ3(**bLhii&>_\²3r;X½:pSý~lllYYG0ëx¡­­MUsmmmïë×¯W~æÌ§~zåÊJ(S±$ë­Jv,´¸eË-677òuÜÑØØ(¡J±©©©rmZZööv½K%>ªäääxúÔÐÉÊÊ¢d³NH.]ºä9úÔï÷ÛÅW%¯y3ß$`ÖIòäóºº:Ïò.1ëh£ð´±±ÑÓAÉfðtuuÅÅÅ¹gù),,ÌÉÉá¬UjjjmÄ¯µfÅ®_¿Ì:hnnVªÈµ¬¬,---%%oÂÀªU«¢££Uò>øà/¼@?`Ö?4GèÜ¹sú9>úèC=ôÌ3Ï444pÖ6jÊ¨óæo*Tçwü~?Ï¢Ì:yÌªZ>==ÝÆÔÔ××+¢í~ÄÄD÷#á|>%urÖòÔò£JUU3$[êêjE®jÐÐu²ÕòB=|øp\µüà¸H~ðàA¥UnC¼V**¡K¨EEE«W¯þò¿üÒK/qµ0ëÇ©å­¢IKKxòÉ'?Îé ù+gÎõÃþ00À¹sçîúqÅ©*ç6ø|>ë¯¬¬pajj*`fðttt¨okkSÔå÷û(//OOOqUï;w92Vkkë¿þë¿fff*ÎØrssÇdF÷ÉåÂ7ÞxÃH®¢ó<æNÔÖÖ>øàK,©©©¡u =Sb`Ö	*wÉ5>>þ¿üeNNNrr²==//Ï3	ß Ù§¤¤H]O?ýô¾ô¥ììlâXñ¢Ü?ñþâ<üðáÃæQ÷És§éõ=|÷»ßýéOZXX¨B¶&]Ou2 IL>]µ|UUsUÆ-((ÛÓÎHë×¯½Ï½ýöÛróÀvN!cwÉrçÎj|X¦ó@råñ·ÚÓoèg*§ÊÌ:I±<V%­1¿ì'Ù8öR`-¿6559]cccø'²wH.Z'°r|ðAíL9gÎ!Æ¬6Vë=nhhP¢µµ*0ë$¡¬¬,55Õ	Xmn¦1¯è:[:11Ñú~¥1Y¿¸¸Xïzæ=ö@rJLtddä¢Eþå_þåÛßþ¶Ò7nâvÚÚÚ´)ùÕ7stfl(äzôÑGÍ=úôé«W¯ó]RH':]¶l&³*]·n:½Â§=Ç|Ú´i3fÌS%H­û÷ïWÑëêoOO÷8ffíêêòûý:`k×®Ý´iSrr²çB`øQTÝÔÔd>KHHxà^xáW!ìÏþó?ú£?rÚðó»ßýîûßÿ¾Ê­¥¥Å¨í~ÈfºfÍËËs«Tì5æ]6ó_FFvOAêÓO?ý_üB¬íØ-[Æðá<ÚàoÏÍÍÅ¬Y1ëÿ|«»3vééé_ëêêg+NÍÊÊRTý­oë¹çÃ(_!µv©··WáõñãÇO:c·-`Ö©nÖ¸¸8Ï5Ëêêê¢¢¢ñ³ùùù¥¥¥½ÚØØ(¥¦¦ÊmÍÍÍÃUµóÔ<Â÷"BòW6ê©§|òÉ+Wò·ÌYÿ©Ôc1=´ù~øá3gÎtwwK´ÒªBízhXû-­ÊÇ7¥jmSç¬/dS²ò#<òÍo~3))iáÂ~¿ÿK_úÚ(³N~³¶¶¶*öª¬¬|ùåããã£¢¢fÎùoÿöoÎ.]:|øpø§=róÖ[o%&&ÊÚ·|ðí·ßÛfÍ5gÎ!ÎµsçN¼îÇûèîuºººÜ·ÄÈCnM´MaàÛ²eËÆÿäOþD¡°âi÷·`Ö)gV±wï^Å2ëOúÓuëÖÙ¬ñ½½½2d£|c5¥pUUUQQöG;ãtË'OT³`(Ö/))Y¿~½'0ÕÖ>~,#Mz.Y²ÄºÇeMåìsVøkÒï-!Ó+~?`VÌ:¬Y³ÆARÞík_öÙg-Zd·]e322Æ*°?úè£ËQh&ËÍÍ­©©ÑwÃÊ¬ËÝ9ZßÌªeS@(é¥£FEÈùTZG[Pò«Îx»J³^ãfffnÜ¸QÎP+[8ãPf3åòòò¯~õ«?þxSSSii©¢IíI[[Û#<¢´"ÅèèèAæeÜ¹s§vþÌ3ú¬d)æääè:Ó:¨$í<ÞD=hT>'NmÎmÄbâq2²0+fK¤ºººO&;´§ÊHZO=õt%ÑVUU9kêùÑ`ß¾Ó§OOMMñÅ5<üðÃÊ9uêÔ'_<4Æ¸_¿B»*É)Zµ»`õ3|ðÁoë[¿þõ¯¿ùÍoFDDÈÓÏ=÷ÜO~òw$ª¬iýÞN$Òô111r°Âz½ê³úÆqr70`VÌ:ÆÔÔÔHWÝÝÝò¨M,	ýâ¿0¶oßîA«÷^ûT7sæL§¯¼òÊÂÍÎ4îûíÎWÅ²Ì÷òË/[Ç¯ä¥ª ø;ßùÎ3lF'D?n¨D¿×suÖÊêoÿöoµ?ìJäãa+À¬uÊ"O>ùä?þã?æææyE1«ÒÒ­#sH<Æ´BÛ0O o=¶´))rÍÏÏwVPûÉÀCßÔJpÆèjµò¬Y³°aP£kµ?ÿó?ÿÊW¾¢XÖA)G¢MHH(--£ßubE·­Ysø'4ÌYÇoä*Ì3GR±¾ÐÎÎÎ~XAáK/½¤#*39Á©¨¨Pà(%àyÝè±fgµÎeÑ¶¶6ýO%Ù´Ãr¤k³&©Ý`6ÚHkÊ¬^myóæ½ûî»R¬ÂP4	³ÞÛ¬ãôø¤>ôÐCO=õ"Ú3gÚðZ|Êw¦Y§H%$Bgà®3q¿öP±ìùóçÈÚCÑ	´BUVr;wÈüà?øë¿þk©Z?©³bÖûÉ¥Kd¯.]ª8ÕémwãÄÄÄ¼ðÂO<ñ'"ÌËËâ÷D(:ÿüÏÿüÀØüG_þòµcÖY½bÅÏþ(ºåW´ÿÿðÿ ÅîÚµËÕ¡££Y³bÖp àÕü$ýØuÍýìgO>ù¤¦pÖÝSZSS£0ìöÄfýµË¢ßüæ7gÌ±víZÅÿþïÿe×JvÕÓÚR¯>ÒÜÜ,N>]?áÛßþöüÇØÝ8ái`Ö©nÖììlMí¤wIIIóæÍStè¾§¤¤dIï/ÝÝÝR¾ßïS£¢¢¶ºnEÎ3R¦*ÅÊ¦)ëëë¥aýµl4fÅ¬£Nqq±ªMdÿÉÏqS¦äºlÙ²ÃKK¿ýíoe/gpSØP¸éLånÝ×k¹n³¡*æ;óµ··ËX2¨¢ØÞÞÞDDDÌ=îÜ¹J<ôÐCJÈjåååñ´Ö£wïãlõòºïù.Å²cûðÌY£±±Q6ôÑG>=ÔÅò~ê©§~ýë_¿õÖ[<òÈ¢EÅ*®ÍÈÈ°Yl5gºDÅwSRRîWi³-þýßÿ½3×ÒK/½¤F·ÊfÅ¬ãjß¾Réo¾))^ºtÉ¦	ü¯ÿú¯O®ÅÚÖ@   ÖFIuÖQü³ýìûßÿ¾[uÆûÂ¡CxàY³f-]ºôßøFdd¤ö³fÅ¬ãÇ+bî¹ÿøÇg1èË/¿l·¾øâÊhóòò¬ØÝ=ÛÚÚêÜNzïÉ©¿ýíoíVÔóçÏ»§À¬ub x4))ÉBCITQlggçôéÓ,XðÆo(±gÏOæ^µjMR_XX%Ý|zÌÈÐ=WU.kÀ¬u¼#eÊV³³³KJJ|>ÛÓÓsðàAU9n¥Ûüü|­öÕ¯~UïVUU555=ûì³QQQ&F¾ÅÓ÷«-|¢*fÅ¬ã95''Çnàw¿ûâÅ¯ýëO<ñlgÓÓ+ üÚ×¾öÀüò¿lkk+**R¹iÓ&ø¾ÍÈÈ°ÇÔ8r³bÖ	IWWWKKËöíÛüqØKyyù7¾ñY³¬¬Li­óî»ï*H7o^JJ´§@öáN÷eÿææfEÃ«¾Q;#§r×`VÌ:á±L"33SU,[WW×ÓÓ£SA¶óD¨Î`ïmÇZëìfÅ¬¢¢"ÉÒ/cÌyàtÈvßúÖ·ÜDØ3j	³bÖÁ(//Gê5''çäÉ---A*LOO·ä]`VÌz¤Òà¡IÊ)--MJJR¨ìs³bVÀ¬³`VÌ³fÅ¬³fÅ¬Y1+fÀ¬0+fÌY0+fÌY³bVÌY1+`VÌY0+fÌY³bVÌY³bVÀ¬³`VÌ³fÅ¬³fÅ¬Y1+fÀ¬÷×_ý+_ùÊÜûÊ9sçBxQ«ä)0óäOúý~Ê!ü¨Øxâ	Ê!Ì¨ùÆ7¾q·=ÙÌzáÂºß|ýë__²dÉ?AxQ«ä)0£¶é´iÓª««)pòáªØÿê¯þ¢3jJ.^¼øþnów¿û]ooï¤2ëhðÌ3Ï¼ñÆCQ«ä)0ÓÜÜ¬*þ¿ÿû¿)pò¿ÿû¿*ö½÷Ra&==½¸¸8<ßY1+fÅ¬Y1+fÅ¬0+fÌY³bVÀ¬³fÅ¬Y1+f½~üã¯_¿r36lPÉSa¦µµ5))éþç(pÒßß¯boii¡(ÂL^^^EEfx`VÌYÇ9'Nðù|óçÏ?xð 2b®]»0HÁRÔCY#ÌÑ£G,X 2	*=<>záÂV&G¡ØÃÉ¦M6n+Ìú9¹¹¹[·nUbãÆË/§@FêäädçY°õPVæH3wî¼tûöíóæÍ£ØÃªÝ=ö(¡Â=6Å6úûûÕtêqXìõsôÇ¸û¶îÅ%KÎ?ï6kpÁRÔCY#583fÌ ØÃÌþýûÅ6Ö­[÷Þï9õÌ8,vÌú9üC¦a$gË¬Á;HQeeÔ ;vìµ×^£ØÃ<Í9S'üöíÛ)öðpñâÅÏ©gÆa±cÖÏpÒQQQÈý2kpÁRÔCY#u'nÜ¸ûé§RìaæÀ=öÅrrr:ä®gÆa±cÖÏÑC¿ÿö'ûbÖà¤¨²2G*$W®õÕW¯^½J±	ÙPìá©^ÜÏbÇ¬³råÊÍ7+¡W5ü)ûeÖà¤¨²2G*Å_»vb'sçÎ=úôgc³UþûXÕ3ã°Ø1ëç9r$>>>""ÂçóéBÜ/³lÈ¢¶eeT0			V<ÅN81þ|E«-²ÞLêqXìÆ6î(v&A±cV¬]»B Ø)vÅY0+fÀ¬Y0+fÌY0+fûg6máÂ¹Ô××oÛ¶M9üñ ë§¥¥¾M¾ôêÕ«mñÓO?Õbmm-¥Y&È@íN<©WôÛûöísr¥Þ¼öîÝ»gÏ­°téR'çÝwßUÎõë×9rD9Îã>l»wï¦ü0+ÀdãÖ­[î°²­­M¯Ç³D¿YP¶Õ.^¼¨ÅË/ëuÑ¢Eé~¤«E±%%%	Ø,îÄÁK,Qú>PzãÆÌ0°3gÎÈpW®ëéÓ§÷ïß¯DOOÇ¬>O©ùuæÌPPH:þ|X·nÝªS§Ny¢^mÓ±ø7ÜÛÌ0y°PU~Ý±cGTTÅ² õñ^¿~ÝcV'µç?[ï±¸çÏÿìË´î7lØ LIWiñûï¿¯3±»víâ@`VÉÃµk×V­Z%Ãmß¾ÝÔ¨S¶SBQf°YµxèÐ¡ëÔûí·yöìÙøøøåË+áÞx__ß²eË¬Ù>ã´¶ÃQÀ¬ûö9Cì:ëÆxíµ×Ì¬û÷ï·5¥O½kýÆvÔ¹ÈjýÀ.S°^?úè#'ØU¨êw²CY&æÈÜÜ_gg§)P²TØªÄÅÔÖ±ë²°1J=¶dyûömJi[ß	cV0+À$Á.µ=zT¯6mr·nÝº9sæ8===ÎGL«õõõÁºzõªîïïÖ'1+fÿÞiÓ-±`Á%vìØ1sæLÓï÷;ë8Põ®W®Ò­É¹sçübVÌ0%°ßÞÞ^¥<8oÞ<G/³ó:Zm4Å£'OÔ«V³._¬È³L~ltÌÑ~¨®®®Ï¾¸£&==ÝtèîÝ½~ýºeÊ¦YeHÏÃYO>=0+L	oÝº%#Î?¿¥¥å®Ú½·¹2¢Ö3gNff¦½ÕÓÓ£·fÍlÖmÛ¶ÙZß³ÛtÌ0å7oÞÖ­[EªiíÚµNÎ7<a«u&oÞ¼À³`VÌ³`VÌ³`VÀ¬³fÀ¬³À0ù¤Æ|Åøm*IEND®B`


血管活性药物使用时间d


正态 Q-Q 图


ïÞ=8xð l¡¦û7'É³K.Êß/&33S&¾üòKºÐ'£ÓýýýòP¶él(-säµMº¼y(÷îÝk~øÒK/Eø6=|øPÿüóÏC%;º!;ùÌåÃËoe×$Ì?>*ä·²ÎÿD^¼TóùÈ1£ ]¹±Ãd^LÞYXºtéºuëôxu<ç³¿(FYþO>±|È§üùóúÑ2¾/òáä?¢ïËúõëÃÿ/æÝùÈÃÐ¶¦¦Éy£­­ÍÈðÛ;IÍ`0(/^ÔBP¦e3*ûþÓelÐµ<úôæÍeëpÿþ³oß¾0Od$«¦/+&.,k3¶ZgºO%%ü4r]¦Ã·n¥dY;ÉÈoÙtË/Q²z<ã¡¤lJJ±ªíÛ·îõõõY¶þú_hHÈ¯,©¦M¤ûCÑ%«îQÉ1çÆæýG¬ß~ûmÑ$s¦/ØóáÊ2YebÓ¦M:!³´ìÉ´|äÌö$K!h~Þ÷ÞOßßG6~~Âÿ/²B©ËeÉÐËÚd5ï¼jq/¯[v@²bN¾©6[nn®n_¤æ3ù«ªª¯´JKKÆ^¾|Ù|DÔ¼3h¹)y ÛÉ$ËÂöì	¿¡×	ÔÔIFTLWMæp¢,,AùyàÀ"óMA"Ðrà7>>^ëosËÏ%«ù¼L=z44rvíÚeÉoyøé§y£5Y¥"Yeþ| o<»4ïªU«d¦ì¯èñãUi1]²JagÞWÐ ÏËËÓ½·)IdêóVWWËþQÊËÄµk×ôÑµ´å_b7Ìù´[~¿ß/súûûC÷B,ÿH$Ç«dÕÏ¤4xèdÅ¼IVÙ)Ö	Ù|úIMY ]½zÕHZ¶,F%¡Å9ç,Éjþ«S§NiE+?õ)õ¤0ÊË©ÍÛ·o[NÝénûn|õ)ô²nµÐºüÌÊÊÒW"¯Á¼B4YF&,5«f¥dÔÍ·9µØ5gµf5]ó2tm¡$F²J±%mõöÛocxÊdýúë¯×¬Y#í)ÿ¦¼áÕØ3ÐfÑµIáe^ÆR^køGògt4XöudzU¦§?lFØEº'$?õ$åªæüêÉ¦			ÆqÉNßCW²¸¸Ø2_Zæë+	óèLOÛF²êÑ~ýò©ÓÝãdÅ¼OÖ­NBkÁ4Y¾ÕD¯Å0;Ùºuëdß8ÿ§éq9#)õ¡ÑT_]¹rE«KÍ:åÿ¢×"Gõ(ôÇ¨JÂÜ²-ÓSÌ[g©ä¡ü/Æ3jVýsK¡l¹D+|Í*ë1þGÍxi7ËÎÊÉªág~Uæ-ÉÚ××gÊtk<£Ñ¡åïE³¹"´$kÑÓ+¶¦»ÐI«;:FòiËÇLß»wïZþêìÙ³MMMòéè÷ÞOcÕ|(Âü¤§§Oùd'fºÏî+)//¬'OÔ|ÌdæùóçÍË¬$+Z²êõ@@~¾úê«]õ¸qÏüú[ùåêGõ°Q/Ø	¬z4Û¨?ôÈ¤ù¹Ó_ikQ+¥>¬ªªÒÒAâGSDR¶ÑºùÓ¬×4U·­¡ÉªbÒðÉª`ÁCCCZëÛa²êE¹º¶¶¶ÐdDÑs÷&mÞ¼9òdMLLôù|ÒbF=&Yåzd¤¼*	6½²,ô¹tGJVe¼rÝ9=®kì9ÕÔÔèòd¦|ôhª¼/½ô|ä¡åLÄçå¬+7ß¡ä·Þò?ðFóJke)Á ù?úè#Ë!Æ´~ÿ0yß¶¼#$+É¬ò+Ù¶üöòåËOíËøø¸fQHI=ÜªY"[-JÞ2æd5qÄqqqºÓÑ²Ì(75,5Òd¦¹[$«Ì­Ê«ÕZÐga¥ÊdÎ0óÑ`Ý2-K¢«Yu¯B:Jê<2Y¿þúëHjV§Óiî$»áuÙ²eó©ºB£|d²>òh°´¿¼TyazS¯W÷Z[@ÿMãè«¼ãØ©^ª¦¯PLyüöÉräÍÕ§§ÓL¬X±B>óæ2÷Ô©Súîèg@w§dÇÂøõ2Î¤ÿÝÈûÐê6l2YµWVÏº«ß)dÅÂ<lüùºMc;]×@ùÕ~¨ÛÙ|H<Gu¢¤¤dÊ²VE²9M¤ù<ë;ï¼#tÉHY	csåz-«&Ô æg1:ÉvP§M5dBAþøé´º2Ê0yIDZCk±ÉyÖ)UOnVuÊz(ÂÕè@bìiiÓvµ0Yå]wJ>æCH`æ.Èò[É9ã¡ä|ltÿ@ÏËC0ö¥²²²,®üJæÈÛ¨¡åý=ö¬qC÷¥ôÔ¦D¸¥oLèç¡ÝiÚÜÆðLVýwôÓ¨'øe¿M^¿&´î´i3Yõº$ËA³ÐK9d«Ût½fDu^$z©y£,T­PÃgÄH7ÙÈJ¥(N£WBiDYl¹¶nÝZVVfÌ46|º¤nÄCC0U·ÔK.ÕÖZhikÞ¾¯Y¥ÙÓÒÒ,ÉúéRäÖ6d­Ç2.òdÊ²ùx@Ô5«QùéëÔ3®Å74ôìµ:Ö°Ñ%/õÝÚÆ2¤ÉåËY-è¹LY¹q¬eÊS­Æ¾úê«ææ½"ã8³Ñ'ÍÒtáÕ|­#udÅ¢KÖ)ÉVÏ¼É.Y-[ËFÙ&YõÀ nß%Å$3]ãV=+l^¹r¥a«NÌç-Éúé=MJ¶õzêtÁ-ÉªWÖTVVÏ>ò(º%Y6ÊtK²êU=Ï*¿ÒÓ®Q'«¡û3JV­ðôà¹X6^ìè|cúïèóä¿ÐãºÚ:îìý'¤1§ØÈ2ªÆtM¡Ý¸Í×*»Ç1	³¤i$G^a.kêd%Y±U6^¡èG55C; OUZÅdå¥Ô½âT6¯ºmÒÓWs½æ29BûöXUË5KvZÖoé>YÍ|h!z=±¤¦ÑtãYUö0âããKãââ"?Ïj«á/m3_ÅI²ûÃèùõ¬¬,s§Ãõ3£×îê DæÓä¬zî©S§$>å]k´ÖÓ]÷ûÈQ·%C¯<uÑ%«&´^0oMd%Y1§ÉH7ÆH²õÔ	§Ó©chGÏÐS^Á¤=¢Ð²]6×¬!)Ì5bäÉ:>)Ldù­zc¾54Y%l´GÒ¯,C	ZUþ£ììl¡Ðràñ»O÷F¬ÆÖÓ<B²ä<nm§Ì°éF2N^¤ya.¬Ï=+Q'ÿò5kfÔÕãphhHv¿´aÑ+õ0æ¦MôùÆ¥ãÆUµZ¥×÷êyúÐS¶Æ1íÐa¤Ì5«­×!txE#YCÏ4¯aÊÑ"-ô¶¹õdjøT¶4¼qÒ¡cªL9³´°ùò7dÅædR$;ï¡5«^:æQÔG7nÜ(Y7I"a±¥6®Â©^E)Q!©ÌmÓòåËuë¯ÛL¬Z¾÷Þ?L'äÓÈ8MkRÇHÒã~L²¡Çît%òòq¦¼bV¶°RuÉ«Ò."S¾°PzðÓrt:ÂKÏOy¡lo·1@®åxq	ùzZËJ3¥m¢dïjýúõ¡TÌ¯Í<F¦ìFÈ~¡ùµÉ~ÀtÃÂ²i|nÍÃ^JSëú0gý<MYùLN¹jùN·.0wò|8I¬o©Æî×ÍwèñX	*ã¬ÊS^¬zã<hø;ÛÇN)¡ôHcL6sòâCGâ5®$2Xíéé9~ü¸TçHòé;z¹ÝÕ0Óz^0t;È?FI'­éî84Â+o_\xËzi±¥Yä/uÊßÊÎÜ#×f|J¬dddd$+$+$+ Y Y Y YÉÉÉHVHV`Î~al¶>øÀ<çæÍ&üèÄ²ä§~fUÁ`pÙ²e7o.túôiãÎðë×¯od¬YÓ²¼ü¹ñÂT\¢¼Û·oç[DøÿFþ¬À´N:eIs²¶¶¶Zøð¡N$''¯]»6Âg¹ÿ¾¬-//Ï¬Û¶m3/3>>>11¡Ó2!äççOùF^Êëé7ß|sº"ù@²Ó2R³$55UbÏ¿/¿üR~9s&ô£HVózäaqqqrYëà+W®OVÙ			±'kä/ÉLËï÷ÈDOOdÉÕ«WÃ×¬±$«¥f­¨¨nÉ¶¶6©¡W®ïÞ½/¾øBshh(L²&&&ÎV²Fò¬À#ê3ù¹gÏ»wïÊÄèè¨%YÇÇÇ×N2ÇÇÇK¸êH=&ÌdµG1Ï9yòd0´,Ö××glKKKÓù×®]ùééé¡Áyüøqöz½a5ò¯¾$+ðÛ·o7jvvvhÍj7QÍêóùJ'ÐÊuÕªUº¤L(1f^¿S&eqqq ½fü YGjU+×~¼&H²Æ~4XóXkA4u:a_¾|ynn®qQ®QËç·ß~°nÝ:½Y©Y#|HV ë×¯KrìÛ·O~ÓY<Ï*«¸Òdi)eâã?Ö·,ùê«¯þ¹ù`²9?ûì3~÷Ýwc¯Y#HV`Z<Øðûýß÷L|ñÅ³¬Á`PÃO*ËGÖ¬3MV	f©2ãââdÿdHV`|+l¶'OZ¦333µ¸<ölèô÷÷ËüÈGN0ZV.ÓYYY:5´Ü=ëóù¦N-[7mÚ4[ÉúÈd¦vãÆ)ç?|ø°ººZâD*Ú)Ðs±MMMá×¯Wý|ûí·ÆÉTÉ×I¬ââbùí5kîÝ»g¶ÒÒÒÐ´3¯äqïj<Ý¬¬ddd$+$k8»víZ¾|y*sÀýÑý×ý×üNÖ_ÿú×ë×¯¿ÀðñSöêgÉú·û·¿`xõÕWIVHV@²¬ddd%Y$+ÉÉÉJ²HV@²¬¬$+d%Y$+ÉÀ¼NÖ%$$¬X±Âçó¬5&ôÑ§~:11!±.s¾ñw÷w$+dHffæÐÐñðûï¿·Måµ×^ã½¬¿wïÞÄÄD)X/]º$s!þþïÿ@²F$..®©©I&®^½ÍyVÉ^zÉ¬5&G+W®äææ¬5&wïÞ-..j5;;ûÚµk$+de¤ÉJ²@²@²¬ddd%Y$+É YIVHV@²¬ddd%Y$+É0Á[·n¬$+`øýþ¼¼¼ÒÒRdÄª¾¾>%%%++«¿¿d%Y1ªùùù6­¼¼<.òÖ Y1innvOêêê¢5HV@ô@IIªòsll!YÑóù|GJÕöövZdÄ¤¶¶Ön·çååùý~ZdD¯¿¿?77×áp444p±ÉIcc£Óéôx<.5HV@ôü~AAÍf«­­¥T%Y1éêêr»Ýiii´É^ (//·Ûí%%%|4`«.x<ËÕØØHk¬Ð¯d%Y`væçç;ºº:.V"Y1Ñ~5ô«!YIVÉØØ]ÍÅJ$+É1Ñ~5~5$+É1	R¤Úív¯×ËÅJ$+É1éïï÷x<~5$+É±ª««s:yyy´ÉJ²@ôü~~~¾Ýng`dX577»ÝnúÕ¬$+Ä*h¿&YIVUWWWzzºT«ííí´ÉJ²@LtàúÕ¬$+Äd`` ''ÇétÒ¯d%Y V---.+##£¿¿Ö YIVÞððpaa¡Ýn¯ªªâb%d´¶¶&%%I©ÚÛÛKk¬$+DOÊÓ)UËËË)UIVb¢ýj¤Zmii¡5HV¢+++¥TÍÏÏ§_ÉJ²@Lsss%Vd%Y &MMMIII¬¯´ÉJ²@ôü~¿ö«©¯¯4ÉJ²@ô¥TÍÊÊòù|´ÉJ²@ônÝºUQQÁ$+É³ »»;===--ûÕ¬$+Ä$îØ±ÃápÑ $+ÉÑ»páBVVÄêîÝ»éWC²¬¦¦&ËÃ À$+É11úÕTWWÓ¯d%Y ÖRUïWÓÝÝMk¬$+DïÖ­[ÅÅÅ6­¤¤~5$+É1éíí:UªUúÕ¬$+ÄÄèW#+ýjHVb-U³²²NgKKýjHVbÒÐÐ C@p¿dHj¿ºº:JUd477»sçÎÑ$+ÉÑ»uëVIIÍf«¬¬¤_ÉJ²@L¤Bõx<R­¶¶¶Ò$+ÉÑµµµN§3//oxx!YIVq¿úúz.V"YIVICCª¬D²¬½_+¬$+Ä¤¹¹Ùívgddø|>Zd%Y zccc^¯×f³Sª¬$+ÄäÜ¹sééé:0­A²¬;vØíöÜÜ&Yçt²>Úf³¬æ²ÞÞÞ8Ð $ëÜMÖñññ5kÖ¬æ2½_MFFä+­A²ÎõdýäOvíÚe$ë¯Cx<ÞKOßïÏËËmTuu5¥*æA²fggOLLÉúZ_~dðTèýjÜn7cÞ$kqqñÙ³gÿ÷q4Àô«©¨¨¡A0oÕöÿ"YÌ½½½iiiIIIR³ÒgÉjXjVO]0¬®®v8EEEÜ¯$+Ä¤¿¿?++KûÕp¿Ìûde¤O×îÝ»¥TÍËË»pá­¢788/¥jmm-¥*HVIcc£Óéd¬«±±±ûÕdtuu¹'uvvÒ Y z@@T»Ýîõzý~?¢×ßßïñxGcc#­bRWWçt:óòòh¬$+èùý~úÕd%YÌææf·ÛÁ YIV1	Ú¯F~Ò¯$+É &]]]éééR­¶··Ó YIVÑµµµv»½  ~5 YIV1¡_HVÀ¬ÑAsrr¸X	$+É &~¿¿  Àf³Ñ¯$+É Vííín·;--AA²¬bB¿¬$+Yãóù<T«ÍÍÍ´HV@L´_M^^ýj@²¬b288ïp8¸X	$+É &Ú¯AA²¬bå÷û½^¯Íf«®®æb%¬$+tvvº'Ñ¯$+É &@ ¦¦Æn·Ñ  YIVÑ;wîýj@²¬bbÜ¯¦¨¨~5XàÉj³Ù>üðCX»v­LôôôÈNeiié'RSS×¬YC²Åàà`NNÓéÜ¹s'ýj°(u×®]:-2=::*Ó­­­òe YDMûÕÈæ~5XøÉÚ××'Á©ªzü¡Cäç74YIVQÛ²eÝn¯©©	4~²NLLh©ºlÙ2MÖwÞyGæ8qÂ¬¢ÐÝÝ>ÉçóÑX,ÉjÞ³gL8ÎuëÖÉ*õ«LÄÇÇoÚ´d9)OKKK¥T-//§_i²NY³r4@¤B:Õív·´´Ð fý?5«¦ìíÛ·IVuuu²%¡_HVk²>|Xfs4@óóóéWÅ¬gÏÕ¿¨2QQQ!Ó.kÛ¶mGÑe¨Y<RKKl:rssûûûi,êdøð¡æëÉ'ÇÇÇõJ¾¾ùæ$+HJÕ»Ý^YYI¿¬ÿëöíÛ£R¤Ê´Ïçé£GÊOÙñ$Y'Û·ÛM¿¬ÿÇÍ7%Då»aÌ¹xñ¢Ì9qâLè±b£~%Y()Oõü×ë¥_HÖÿ(¸¯¯/´~@5æ~]«XáÂ.«©©ÖÉÊ½nÄd÷îÝ£  ~5 YIV1éïïÏÎÎX­««£5@²sæÌ¶¶6@û÷ïLÍÊÊ¢_HÖHøì³Ïé6LÒ+N:E²ÖÈÈHqq1ýj@²F¤££Ãëõ¶¶¶ã<yR~¦¦¦J¦ÉzñâEX:;;éWufwuIvë×íÛ·ã1¬Àâ!åiUUªô«É:3S&ëªU«Ì5«1jÉ,R¡z<ËuìØ1Z$k4Ézùòeùß¸qC?~XÁ`mm-ýj@²F¬zbUÜ½WstãÆ2ñàÁ)Xßyçãvr$+°àgeeÑ¯$k¬×_½zU~/[¶ìÝwßµüêÎ;ôºææf·ÛA¿¬Ñ'ëwß;©d*R³ÖÔÔè±bXÀÆÆÆä+O¿¬³S³Þ¿ßÒ±U"Å£µµ5--MªÕöövZ$ë,$ëÄÄDæ$íØ*ÉÐ:I¦W¬XA²Õ­[·***¤T-,,¤_HÖÙ<Ïª÷?®fµµ$+°0ø|¾)Ui¬³¬Z­j×U£÷ªËå²Ì$+°TWWK©?00@k³¬<à^7À¢2<<èp8vîÜià÷iDþ	)L.7÷ïßMMM$+°´´´$%%egg;wÖo²öôôH?Þ¬ïÖr$+ðùýþââbù^Ó¯xÉ*ß·Ó§OËw¯µµõðáÃ:Lùå2½oß>§ÚÛÛÝn·T«ò¦5'¬Z¡^¹rÅ8ízïÞ=#AKÍÌk·nÝª¬¬Ô~5###4ðuÏ=£'Né¥KÊÄç.skÍô¬À|¡÷«q:»wïæb%àÉ%ëÃ7mÚ$ñyãÆ=,\P|øðaGô~5RªÓ ÀMV=,?¿þúk¨õ«¹?+÷gæÁÁÁììlÃÑÐÐ@©<édMNN6ßuÊû³óEcc£ËåÊÈÈ¸pá­<ÕÔ¬À<eô«©ªªºuëÌ¡d¥fæcÇ¹ÝîúÕÔ¬bä«*ßÓ¢¢"úÕó¦f½råÊÄÄÉÌ5ÝÝÝzkUJU`.&ëáÃ²ZjÖªª*ùÙÓÓC²s§TÕûÕúý~[É:>>.ÁyðàÁÙ-LIVà1ôx<£±±ÖæhÍÊ]äùbÿþýN§3++~5ÀMÖ¾¾¾7&y'µþH§uh~&Y§Îï÷ÉW²¦¦ûÕs7YÇÇÇ-å»gÏjV`NÑûÕ¤¥¥uwwÓÀNÖ)¯f"Y¹Ã¸_Mii)C@$+ÉÄäÜ¹séééN§~5À|JÖ	IÓmÛ¶&ëÃIVài©ªªÒûÕÒÀ<«YÍi*Ó.K¯`ÒixÂ$PG=÷«B²Ær4øüùókÖ¬ÏÊÊºxñ"ÉÌTss³ìÑfggÓ¯Ñijj*,,ôx<EEE]]]4È¼OÖ+Wêë;räÈªU«HV rccc²A´ÛíÕÕÕô«AtvîÜÒÒÒ288ØØØ$Ó4ËüNV³ÄÄÄï¿ÿÞB6¯½öï%`&µEzzºl>­è¸c|$Q[ ÉÚ××·uëV8âç?ÿyAAï% d«W^^.^¯AöööââbËÌÎ,Ì­dQ¬ðÞ½²¸ÿ>Gð¤¤ðx<RgpÈ³¬EEE³¢ÉzúôiÙ»Ñk¥éëéÊÃøøø­öæÍeee·oßæÚ` ¼íW3<<Lk v###Ã£~¢ÉúàÁYïQ#;à6l¸sç½n0ü~^^lwïÞMk`566ºÝîúúú®®®ÚÚZ>vìÍòäõqHMMµ¬ÀtÛ>ÇÓßßOk`ÖuwwWTTÊO/de¤ [·ny½^ÙéM"YåÿÆo¬Àcª$´_Mgg'­,¢dýäOÌs¾ûî;Q0ÔAKJJ(U¬>oÓ$½øÈ#¬ëÖ­3îÞÔÔ$sä)IV jIIIÍÍÍ´°ÀõÁæÛ>|899¹¸¸£ÁÀlihhp¹,d½Ü3gFFFd"LoTR Ø¿EEEMMÏç(@o<ä~5ÀbLVs'S§NÉôÐÐÉDèÖ­[ÙÙÙ¹¹¹®uuuË-ûÉO~ÆFÅ¬<MNNÖÚÚÚp¿víÉ<Reeeqq±Ô¦R¹È×ç¹ç;qâ-,®d½÷îÑ£Geçº¯¯OçÈ@¶/_éÛ·oWWWËÃYdÂHOOÖûÕ¸Ýîööv©`%ë;w.]ªö÷÷K|Þ¸qCrôÀæÅÆÇÇ©YGr¹ýíou`½X©±±±¢¢WÍ*$M>ßéÓ§%S°2122"*Ñ+Óûöí#Yðdßô¹ç45f544Ð8À¢KVÉÎ'Oj²ê)U	W/ÉzùòeØ¾;ÉLGâÓét®^½ú^ÐÁd'µ¦¦&##A!E¬zU¶ædUMMMzm°ÌÿôÓOIV ßïÏÏÏïHmmm0lnnv»Ý)))III2Ã´f5	k¯·'mÞ¼Y¦-[F¯`:£Rvwwçp/Ldå^7Xø$í*++ÓÓÓ].WNNN,«Ò~5¬pùòåÓ§Oç_;::HV,[¶lÉËËéÖÖÖ¤¤¤ènmîWC«$ëa	#eIVÌwÝÝÝiiiæ´)))3ZI0¬­­5÷«@²>â¾7¦===úðÚµkzqÉ ¡¡¡ººÚ2Sull,Â5ô÷÷<ÃaîWd7jDè5À£¿É§bÿþý4)))Â³¤uuuN§3''çÂ4&@²F:nð¶mÛ¦[¯×K²b¾Õ¬j÷îÝüCíWc·Ûµ_-	¬vÿþ,Â¸ºqÛó/¾øbýúõzòõâÅæ;¹¬xê#¿4·¾¾>%%E×®®®ªª*·ÛÝßßþO].WFF¥*@²Îìª¥«W¯êýYõáÚµkõWmmmòP»ºÊ&I2dÅ-çt:wìØá;v¬¤¤¤°°°ººzpp0ÌcccÚ¯¦¼¼~5ÉëåÁ|ðN>|XÞ»w£ÁS¥jRRÒîÝ»õØ¬¤ÇãÙ¹sç,>µîI:T!5z×¯_7w¶iÙfqæúúz©&-A(õë¬¬<H9k·Û½^/ýjuvú³¾ñÆæÙÙÙ$+æÊÊÊÐ~/#ö5Ó¯ Yg9YÏ9#Q:::jNÖââbs­fµÜô´··7öUûÕ4 YcMÖãÇKêELW¯^MMMo&»wïöz½²'áô<kkk«.pëÖ-CÛ¨~5fÿÎçÉÉÉmmm¡·¾Ùºu+£â)ËÉÉ)((8vìXaaavvöÈÈHwwwJJÄaIILÈÏ¨~5~Ï½n°xTVVSS*×òòòßO^jÔÕÕÕÒÒòÈ>©Ób·¢¢~5HV,"R°öööçÈC)[c_³¬'---))~5o²>|øPváßyçs$Y-iuW«ýj¤ü?XuU¯Zº|ù²yæwßG²â	èîîÎÍÍu:RMêÚÊÊÊÒÒRó2z48:ÌYYYò48Ç¬>Ï.¸µµUbÕårÉyô`óM[IV<&½½½òÙÛ¿0ôûý%%%R°ÊDFF«T®«ºQà +L<Æd7¦;::t,Ãk×®=IV/Ég'sÆÆÆ¤rÍ$Õjt±*UXX(ïªª*.VðxÕrÇ½?ëÁÓpÁ$+¦ãv»-=gBï¨cÇ¥¤¤H±ËÅJh²nÛ¶MÒ´¢¢¢µµ5!!!11±uLoØ°dÅãÓØØ¸eËùÈmß¾]*Tc~½±¬YÊÓÒÒR»Ý.ë§TðDÕr251ÀaVVÉÇDvæ222dN>uÆiTXnoozÍ]]]²B)Y	ÀMÖ3gÎÈ¨§§GBôÊ+¡Éú8å'Y¡á'ñ922¢Q*´ýìguuu2ù]W-$G%°¥TÍËË¦<Ñdõù|ZÊÉ'¬¦¦Æ|_Õ@ ð¯ÿú¯CæwwwG·NJBbÕ¸+<¹d½sçFÄ¬OR°  @vÅ¼^¯eh¤èTWW[úJº¨W¨÷«ñx<³òò¬1]ÁdNÖÔÔÔU«VM²1%YQYY)ÕÙÙ988(å ä_Ôe¥¡©©IrÚÊsss£X¼*íW#áJ©`N$«ÞõÆ2"É:11ñÉ'ÈÌûö¬ò©Ð¢ª¥¥%ö[¢JÊ~[~~¾ôÀÀ@mm­<Ë¹sçfºÆÆF¹~5æP²ÉÖmåÊ:­wl%Y¹Ð0n·;ö®,@@UêT)^·lÙ2Ó»Ëðz½²ÿWRRB¿s7Yu;ÅL0'kè ³¬±êVöµ_ï9¬ÜE½½½`³~48jÁ`PïWSXXh~U@²bÞxW0EçÂz¿;wr±dÇ$Éòòò$_½^o×ÍJ©*¡Îýj¬$+fßïÏÍÍu8µµµd%YææfËæóùh$+ÉèIyZ\l³ÙÊËË)U¬$+bÒÙÙ)ujRRRkk+­d%Y½@ PUUe·ÛKJJ¸_d]8âããm6Û3Ï<ó7ó7OæIµ_<;od]8jkkm,Y"vûIõ~5ô«@²¬D©ªñð/ÿò/eNiiéczºááá¢¢"»Ý¾cÇ@²¬ää¨åx¬Ö¯ãé¸_d]°$S%G-3¥Ý'+//Rµªª~5HVu!dÍÎÎ¶Ìq»Ý³ø½½½²ÎöövÉJ².pF¸j	+f«L0"ÕáplÙ²ûÕ YIÖÅÂn·ÛLvîÜ9+«ÌËËs:û÷ï§¬$ë¢S[[;Ã5èýj$Yh[$+Éèùýþüü|©kjjèWd%YÖÖV·Ûþ´n$+¦.ÌJe¼^¯ª[¶l¡_d]t:;;SRR²²²rrrZZZbY[WWÇãõ477Ó¶HVuÑt»ÝFú|>	EIÇèÖV[[k·ÛÇÆÆh[$+ÉºUUUIç466Ît=¹¹¹Ú¯¬$ë""Õä±cÇÚÚÚ<,**²T¨RÅfddÌhÆ©¬ÜZÉJ²..o½õÖ%K.]÷ÕW_UTTìÞ½Û¼L~~~+)..¶Ûí555Õ@²¬(V%M¥ZÕò¦HÊîÝ»7))©¿¿_gJÑ)kw b7%%%==ÝçóÑ¼HVuÑ:Õ2Z¡âk¯½ÖØØèr¹¼^¯LÔ××?rUÁ`Ð¸XRÉJ².Ræ»«ÒÒÒÕ«Wÿ~òäkkkk$'JÏ;-ÜÜÜÌÅJHVuQ×¬_õyÎÿùKÍ:£HEët:iR$+Éº¨ýò¿p½té>Ü¿ÿ3Ï<ùÐ¥ùùùv»½¶¶RÉJ²âåää,Y²äÿø_|ñEØ±cG¨ýj222.@3 YIÖÅîÀ«W¯^¾|ùÏþó'OVWWÿö·¿5×ðü~¿,Å ÀHV¿åW$öYË%RªEø·]]]îI´$õ	¹xñbJJJ|||ffæ3gHÖ9åý÷ß4-++Ó£££qqqÏ=÷Ü#ÿ0Ûíö@²>Q^¯÷Ð¡C2±oß¾Í7ËDg7¬OÅ/¾øüóÏçÔ××KÖÿ«.x<©qiC$ë<11!>LMMýþûïmSiïÌÄÄÄ?ù?±Ì¬:D^^ßï§¬OA||¼eúa­[·R³>/¿ü²Ä¤yÎ/~ñéUûÕ8ºº:úÕ Y¸¸8c:!!ó¬sÊ7ß|#9*ù:::*÷îÝ+uÜ%úÕ YçJ²¾ôÒKRj©*Ó$ë1éUÁòÓårY _un%ëÛo¿ðàA²&Yç¡¡¡_þò?ÿùÏ;&ÕjYYY__e1úÕ Yçöôô,_¾<...%%åüùó$ëèÐ!yGÒÓÓKKKuÜ%°,C¿$+#E RR°VÂÕ¸3«ðù|ô«@²¬ÈÉ'öYËmSW¯^md-ýj¬$+"ÕÚÚ*	*ÉÚÒÒbÿWõW¿øÅ/úûûsssGCCýj¬$+Â"µ¨¨ÈãñÔÕÕ-Y²ä§?ý©<ÔÊull,>>^×étÊô«@²¬x´ÚÚZ©G5Jßzë­¸¸¸?ýÓ?­®®nkk[¶lYBBÍfãÖª@²"R«æÛÿã?þ£¦©Ýnõå_¦_¬ó/Jµ*áZRRÂ@²bþó?ÿsÍ5ÆÝâ~?Ù¯&99Ùáp477Ó>@²"Rÿþïÿn·Ûõ¨¯ü(íëëûùgy&>>^ò&ºyó¦$èþáÊ<ìèè:DðêÕ«¹HVÌ×ë3666&&&J²<xÆ3*###%%%Ú¯Æn·ÿó?ÿ3$+f¬°°Ðét¶´´¸ôôôîîî7oZ¬ÈÀÀÀ;ô¬ê-[´_MZZZ\v¬TEEÔ©ð ÷3OHHLµÛíòð«¯¾¢dÅìÚµ+))IjÓ¼¼<©îÜ)É^XXøßÿýß´¬ÞÞ^Ã!±*jãÉihhÐXµÑØØX^^NûÉHùýþüü|ÍVUUµzõjK²J¬R³ÉHuvv¦¤¤dddÈÄï~÷»;vÈ´1þ~sssRRÒàà $+!TWWÛíö-[¶8p@Ö6qqq¹¹¹zi+ YñçÎóx<N§óØ±cRªJºÝn½ú÷W¿úäëë¯¿.+73 a©¥jqq±æ]·nÝ³Ï>k^Æ2h0dÅÔ$JsssÇîÝ»ÉÉÉ®%¥l¥¹dE8---)))iiiS§+V¬æ9mmm$+¬Ö­[·¶lÙ"aYSS£½ÿþûò«Ï?ÿÞ¼y311ÑårÑn@²b]]]ééén·»½½ºeþú¯ÿZÂUÒ455uÉ%ãw¿ûM$+þ@ ¢¢Bb²¼¼|ll,üÂ_õÕºuë233õ«_qC YaÕÛÛÔÜÜLkÉJ²Æd÷îÝRªæääÓ@²¬Ñóûý«û÷ïg YIÖ´··»Ýî¬¬,$dUww·Ýn¯««i©ÚÑÑAëÉJ²N¡¿¿FË?ÿüó¶9ÑÑQÚHV5J.Kµ¾¾þ÷Ã3=óÌ3RòÒ,@²¬ÑèëëX=pà1ghhHæ¼ÿþû4¬$ëýæ7¿Y²def||ü+¯¼BãÉJ²ÎT«R¡ZN¬JÖ¾þúë4¬$k4$YR­Ê®d©l:ÕårÅÅÅÉtYYÍ$+É½¡¡!ù¼øâögöÍ7ßÐ @²¬d¬$+$+$+É YIVHVHV@²¬ddd%Y$+ÉÉÉJ²HVuNÛ»w¯ÞçóÌ3ò6dÒ$P_xá¾I.KvttÐ2@²¬ÑxöÙgþyóøøxËÉJ²FjÉ%õõõæ9¯¿þz\-$+Ée²ç¼òÊ+$+¬$k^xá	×ÑÑQ(6måÊ´¬$k4ôÂà'ÉD||¼´dÆºuë´zõjbHV@²¬ddd%Y$+ÉÉÉJ²HV@²¬¬$+d%Y$ëSpþüù5kÖÄÇÇgee]¼xd¬1Y¹r¥¾¾#G¬Zµd¬³&11Q~n^PPÀ	 Yg ¯¯oëÖ­$+d÷îÝóz½÷ïßçh0d1ÛôáÍ7ËÊÊnß¾ÍµÁ5V>oÃwîÜ¡×d©©©6@²2Rd%Y Y YIVÉJ²@²@²¬d¬$+$+É YIVÉJ²@²@²Feûöí?ùÉOV"?ýéOÓÒÒhIJKÒ±øI´_çùuv:ó>Y¯_¿þÿ!2¿ùÍol6[SSMiÃ_ÿú×´C,~ö³­]»vÅ?üÃ?,Y²vÅW_%_gÙ0Îîjÿíßþ-ÎïdEäÚÛÛåcôý÷ßÓ±6lkk£bñÖ[omÞ¼vEkk«$+íÿùÿ¯sGGÇÚtÐâ$+HVd%YIV¬$+ÉJ²dÉJ²¬ YIV¬$+í@²¬$+Éº»»322HÖIþÇüíú§Ú¾;íÓ§O¯ZµvÅÄÄ|¿ùæùdddÅqñâÅøøøÌÌÌ3gÎÐ 3råÊµk×jëõôôÐQxðàAYYYBBÂ+|>m«W¯fggK¾ùæwïÞ¥gêÎ;©©©ÆÃóçÏ¯Y³FZ/++KZò	´'ÉºÐx½ÞCÉÄ¾û¸&s¦äk¦W~ûí·ÉÉÉ´g>úè£O?ýtbbBb5==6ìÞIÈÄÐÐPee%m8#²Oìñxl¶ÿn+W®ÔÑ9¢×=îö$YÉÙ¨ÉÄÃÍmS§NÉî-íÝÞäÉXH-eL»ÚpF6nÜxíÚ5s²%&&>ö$YòwÒ</]ºT¾²KF÷	Ü»w¯l¿¤`½témÙ«»råL>|X[6q¶M¬[·níI².4qqqÆtBBÓ§O¿ôÒK´gtÀ¦¦¦~<YHFAöHV­Z%'»víÒ6=YïÝ»çõzïß¿ÿÚd]h$>|¨G94KõOFñ	¤gßï÷x<´aìÉzóæÍ²²²Û·o?í$ÉºÐ¼ýöÛ	ù);h4È¬RÂ?~Ã´g*++=úÃäÖ¹¹¹´atÃ/NLLHÍúñÇÓ1&«Ïç¯ó;wØvd]hzzz/_¢"r²9ËÌÌJkýúõºKÎÔÝ»w¥³³³¯]»FFA ---!!aëÖ­ããã´aÉj3yíI²@²@²@²¬¬¬¬dd0_cmíÚµÞI­­­9ß~ûmåõ.4aLLLÈbï¾û®>¼ÿ¾<Ô¯ YåôéÓÅ$D%í.]º$?õXæ<yò¤1ÇãñHôL:qâDGG,ðÆos>ûì3s÷î]ãOzzzdq]g[[í¬ÀBóàÁsYÙßß/?ûúútb||X7nÜë×¯×æUj[]]m#¬)nÔÁ7né/¿üR¦÷íÛÇ¬ÀB 7CCCp7oÞW®uêLK²¦¤¤HaªùºtéR©PeBJÒÌÌL-X:$s._¾l©zeFß»wÏ¼N$+°ph©*ùzôèÑ­/%õïÝ»w-ÉjÔ zÏg=z¬®ÞÓÞ³gÌÐiãÏ?ÿhËÄñãÇy#X8îÜ¹³mÛ6I¸#Gh4JÅ)i'Re&«<<öljjªÔýøãeæÕ«W/_¾yóf0¯üáÃ6mÒÈú·ÅÅÅõð.$+° <yÒ¸¤HÏ³îÛ·O&¶nÝªÉzêÔ)]RâS~«Çõ©qU_¿~ÝRËÏ¯¿þÚ(v¥T5.wÒ· YE3Òëõjøùý~@	K)[ebttÔ¨Au=/~b^£¤ÉÉÉòW2­ËqhÍª¿@²j=þ¼üüâ/ÀûäOV¬Xa<Æh¬¶¶¶®êöíÛzÙðøøxh|R³$+°¾·6[UUN¬Y³F&=ºtéR	Î´´4cãªüV¾ýöÛF@cråÊS>5+@²ø2æÌU«Vi=*ó%Y5ó¤ú4NµêÕLZ^ºtI~ÊbzÈWÖ0áMÍ¬ÀÂ§W'¯9:pàÌùáÇ5yyyæ£»wïÞÕ¦F²JâÊçÖ¬W®ÊÉ`Þ0 |ðà$bff¦Ïçä_µµµi+(Ë¯X±¢  @äWË-MÖÃë²¼¥f­ªªÒá&xGXtV­ZuèÐ!ã¡ÕôÑGsîÝ»g)[õ`òÁ)L¬¬¬¬ddd$+$+$+ Y Y Y Y@TþCÄ¯côã¡aIEND®B`


ß×ûpøáùq¿ýAÞçÍá÷Ãûñ~¾Þ¯óz;~SÇÁ&ddd$+$+$+ Y Y Y YÉÉÉHVHVLÙv8²²²ø´¦¦&yæ7l»aÃ¿ÏoiiùòË/ýþjxxøàÁò·[·nõ]ÂÂBÛÌcÇÅÇÇß¿?Àâ8p@þv``À¼ÎÊ+uúÚµk~×ú7ÞÐé+b466ÊÓ®_¿n·ß~¬ußæææzF5þÈ<[¼xñ8÷Ñwß'¯öáúþêóÏ?M=­YÛ±¼»»»pÇYë;wÊÞ7oÞ|àR­_¿>èúäOÆúpúµwï^ó©·ÿ¦OWàç,Z´È|0ün4y,0G*aC ¼¼<ðÓ4ÆL;wîðáÃ2±zõjóÿoãr¹túöíÛÇÿà$ðô¥äÈxöìYßÍ/¾¨ÓwïÞÕÝ»wËÊoØÿb¼»ä¢ümIIYììlØµk¾ïÉÂètGG<cúx¶9²lfù>ßz@~ú©õáO<1ÎÝ444$Ïÿì³Ï|%'z!'ËFYÊûöíßÊ©Iò[yÍqþ,,ªu|dGL(È_Ü0Y&V&æÎ»dÉÝã²%å/Ê§Q¿iÓ&ÛÜïÏ?¯-óÿ"þ®î¥K^ëiüÈÃÞÞ^ß­ªªâ`E²"l9rÄdCàã¤æàà LpAAÃ¨ûIr@×:òôéÓk×®£Ã½÷dÎ;¼IVM)_æò²¼9Új±*<ý òÓäºL'&&Þz,eñ(ßrè4g©©©JV·ÛmJÊ¦¥¥zë­·|OÚÚÚlG]	ù-ÕtéùPpÉªgTrcæÜ¸qÃz>ñ@õ«¯¾Z=ÊZé9ï¿ÿ¾Ìi¿É*kÖ¬Ñ	©eÈ	LËGÎZñç²Ö÷÷Ýwuÿ>péç'ðºÈJ].Ï,..ö=Ñ±½IVëÉ«÷²ä:ßv!¹Süü|=¾HÍgýç¯¨¨0ÿÒ*##Cf?¼xñ¢µEÔz3´Ü<ã¾díÉÛ·o| ×	ÔôQ&*Æª&­¿Ð(O üüüóÏ5¬G4Ý¶ß¸¸8­¿­[&pÆL(Y­mò2ðàAßÈÙºu«-¿åáÇ`Gk²J1D²Êü÷ÞOv¼»lÞÊL9_Ñö³TZÌ¬RØYÏ4ÈôìÍ/LßÊÊJ9ÿ0¥¼LzU÷¦¬m;ØVMÝO0äÓn+úzzdNGGïYmEÆÓ^mU?²ÁHHVa¬rR¬rø¶6úIù-­._¾l-$AmGSIh±eÍ9[²ZÿêäÉZÑÊOääd½$)Lùb»´yëÖ-Û¥;SßcÐ¯¾6 ëX9 ËÏ]Yëj¤ÉsdÂV³jæÙJF=|[£Q]óÐÖÕlvÍKßWó=#1É*Ål«×^ÍÃ~õË/¿hlOYMÙÕèfÑWÂËú[y­ágZò'Ô,ç:òÚ*Ó~£66æ¬HÏä§^¤°µ©jnÉ¯~½hoÚ]$;u¿ûn^È¢¢"Û|yk¯K`EôÌL¦Çm¬ÚÚ¯Ë/:=1HVa¬iiiZøÖ¸¶Æ4y~£öÅ°¶ÊlÉ%ro®ÿéÓ´]Î$¥6ÁIj®¤üêÒ¥KZØjV¿ë¢LK²¶Bûm£6uÈ±L/qire=:KU$e]46ÌUÿÜV(Ûºh®YåuÌ:jÆËv³¬øMV?ëRYlKÖ¶¶6?cmXóZFû¿~fkEhKÖ£¹¢[LlÕÑI«':&ù4åc¦ûâÎ;¶¿:wî¬þ^x÷Ýw5V­MÖÉÌÌô»r3ÖgLÏÖ­[g^¤´´4p²655é|Ìdæùóç­Ï!YIVDZ²jÈùùÜsÏÙ:»j»iÇ³¾þV~ekAõÛ¬Íz¦$µvØ	¬Úmêm´^«³pú+r-j¥ÓZ:HühhRÊ1ZzÕë&YõØê¬ºAlQ8Yõ¬ÜÝÝ­µ¸î¦q&«vÊÕW8räo²J¢è5Ë»£Ö®];þdMLLôz½²ÅL= YÍÅríA&)K%Á¦=Ë|ßKO¤ä¥ÌëÉo»®9sªªªÒ'È"ÉLùhkªìÇ'xB>EòÐv%âÑëÄòIÖ·ß¾ä·Þ²G¬x³y%Åµ²Ö?üàlûBÎ8Í´~}[í§¦íÛ¶=B²¬Àd_É!ØúdÛo/^¼(ùd;¾k&B*@Bhs«fýµ(1ß±&«µÓ$Ñcfe¦ÜÔ°ÔHÖ¯åH²ÊYlíü)K«ZÐ«°RNe²fµ5XãÖLÉàjV=«ÐVGI&ë_~9ÕétZ¿$§uÞ¼y¶ë©ú¦|`²>°5X¶¿,ª,¦ ^ÅÔþJ²¯uèjÖWYÓvª]Õt	µýÀoûí£rãããeçê[ÈÛéGE&æÏ/ykòäIÝ;úÐÓ)9±0+¨+e®RËº¼÷mT_¾|¹ßdÕoµiõ¬gùùùú?E²¬ÌÖ`óWÖ~æ;ÖWåWï¿ÿ¾÷åð!ñlZu¢¸¸ØïAY«"9É!Òzõ7Þ'|ýõ×&e%­«o_VM©A­ïb¾D$ÇA6M5dB7ü¹ñ3GiueÊ,íDZCk±2ë¬~UOßnJÕo=4ÎÕ|Äié¦óýªå8Uö²ì)ù0X4$$À¬_AßJÎò±Ñó½:.eÂKåääØ*]ùÌ¶©¡eÿ;wÎÐs)½´)nûnoÏsß¯ÓmMnÓþÉª«£F½À/çm²üÐzÒ¦u<HVDf²j¿$[£oW9æê1]ûhS§o'aß®RÖ²RµB|UVÄ$Äd¥RC§ÎÑPúDy59r­_¿~Ýºuf¦9ðé3õ î;IV=RÏ;WsÅ´ÖÚhik=¾®Ye³gddØõKWä¶ë7mlÉ*­m¦²J	eµ= èÕT~ºa¦/®ìPß«×Út¬a£O¼Ô½)?õû3¶!M.^¼hVYôZ¦¼¸ikñ©Õ¼ésÏ=gh=+2íÌæ;i¶M8Y­åX`ÖSLjVQ¬~ÉQÏzÈ+YmGÛAÙ¬Ú0¨ÇwIqdæÝµÏXõª°iÎÊÊ2fëub½flKÖ,=zô2­/9ÖkÔ±ÁmÉª=kÊËË­WIØnKV³MnKV½°ª×YåWzÙ5èdÕÑ÷ÜbBÉª6kÃ²Y$9ÐùætuôM3¬¶ëêFÐq?äìÇéw`#Û¨cmý·µ¯²õë1ÖçaÂli:Ö`¡=Ìåú"YIVDB²ÊÁË·õÛ¬©éûDë¥JÛÐS¬ò|)õ|ÊáUMzùÊv­×ºü&ïwlÉªå-;m¯oû:JàdµVð¾ o(óLM£±Æ²%«aÄÅÅ.Á111ã¿Îj«á»¶Y±'Y­ßÑëë999Ö2Ãõ3£wu"ëerMVí£òäIOÙArê »F¿^¥µàXý~8êy¦oÏÀQjBkyÛh$+É& =P"äè©N§SÇÐ/zú~cÏo&ý¦ )mÇekÍjÂZ#?YG(l¿ÕQo¬ÝYUÂF¿Q£´ãm(A[²Êåææê¶Ç~üZõrïxÕ=­#$KnÉ[èÑÖo5Rq²:äµ°>wîD¬ò¢E&ôV3Æaww·~é5£Wj3æ5ktÏÊ|Óu<11ÑôªÕ²ÌüöïÕëô¾lM¶ï0RÖU¯Öë:¼¢IVßá3­¯àw´H½mÝz15p*Û6ì8Ù¾cªøY¶°µûÉJ²"Ô¸4wßUûåúytkðÊ+%«Fé[Ä2GjÓW¦ÚR¢B=RcSjjªýµ»ßdÕZðÝwßýaô:$Ö@æ2­!¥¤í~LrÅÐ¶;Y<3ß³rªKJ¿"âwÁ|iã§­uz<^9öÛQv»Ûkko0]À¬ýim*Mn%gWK.õýuÙ¬cdÊiZMÎÆÐ49eÓ|n­Ã^Ê¦Ö+ô®ú<öMYùLú=µýO[X¿ä3þá$A²"Ü>þÆî×Ã·o¬iBÙog%ßÛËë ïlcÚÍR|iKcLs²ð¾#ñDV«---ÒvóI>sG»ké¥AßSk1­×³ÿGÑtòÑëCcÑ Ã­ì¾ßñµk±ífã_T¿¿¹¾Bñ)A²¬¬¬dddd$+$+$+ Y Yýq8Þï=ë7o.Uü£ãÇ;vLùñÇx©ÁÁÁyóæ­]»vÝ¨Ó§O;Ã×®][9Ê¼²0Óò|ùs³`*&&&11QçÖ­[Öù6Zëñ,*ÓÉ'­é%1iMÖÆÆFÛót"99yñâÅã|÷îÉ«XãjÃÖçè´LÈ-[6Vä¼åé_üâc=a¢Æ³¨HV`L&)5ÌÒÓÓ%KL¢ìÚµK~=Ö÷HVëëÈÃ¢¢¢å²ÖÁ.]¬ºØñññS¬ã_Tdð[zzze¢¥¥E"äòåËkÖÉ$«­,++ëG:++ëîÝ»;wîàìîî¬ÓZ³ú]T$+0f(¹%?·oß~çÎ¸~ýº-Y2MÇqqq®z)T[¼ì¬smOkkk3-Ò®:ÿêÕ«2?33Ó78>,Ó'@²Nèâë8ÉòÖ[oY577×·f5íÆªY½^oÉ¨u>´p¡>S&JKKÍJZ__KI¿IYTT4000ùuüd@ªU­ø±O¶²N¾5XóXQr:ozXþÐ78¿úê«øøø%KHîN¾fè¢ Yÿ®]»&Y²cÇùi-L§ð:«¼ä¥ÆLK,~ø¡F¸íÏ=÷ï[[h­ÑøÉ'ÈôÛo¿=ùu¢dü¸ÿ¾dFOOÏwß';wîòdÔðÊòàDUÒNÊÜ9?|²NhQ¬ÿ$kjj²MgggkÅvîÜ9ß?éèèùÖ¡øÐòâ2 øÓPômöz½c§­kÖ¬|²NhQ¬Ý7üÎª¬¬hÖïôZlCCCà××nG_õ#A%¡UUU%YTT$¿]´hÑÝ»w­ÙVRRâxÖA,*¬¬¬ÁÖ­[SSSÓO>ùä¿ýÛ¿w²þò¿téeBÀ3Ï<ã÷kåa¬?ÿùÏ¿ <÷Üs$+$+É YIVÉJ²@²@²¬ddd%Y$+É YIVHV@²¬d ¬õþýûëÖ­?¾×ë%Y$ë¤|ðÁüñÈÈÄjff¦ÌùÊÇþé¬u³³»»»ÍÃï¿ÿÞáÏ+ØõÁâââ>ýôÓÄÄD)X¿þúk3àãÏÿüÏ©Y$ë¸ÄÄÄ444ÈÄåËsss¹Î Y'å'°Ö¯$+dòòòÊÄ¥KòóóIVÉ:)wîÜ)**j577÷êÕ«$+de¤ÉJ²@²@²¬ddd%Y$+É YIVHV@²¬ddd%Y$+ÉÉÉJ²HV@²¬¬¬$+d%Y Y YIVÉJ²HVd¬$+d%Y Y YIVÉJ²@²@²¬d¬$+ tvvõõõ¬$+`RjjjÜn·ä+ÉJ²×ØØár¹êêê$bgìIV@¤ò4??ßáp¬Zµª··wßdD¾¾¾ÊÊÊØØØ8qâ¡,ÉöìIIIq¹55ýýýk1HV@Økoo/((p8§§§çá.Éc¦ù733óèÑ£¡°H$+ ××§¤¤$$$ÔÔÔÈR¬ðÓÞÞ®½=ÏQdD ¾¾¾ÒÒÒØØX·ÛêÔ©6´ùWÿNó/É?ÖæßÞûd±Ðoþ%Ya#,IV@ðz½áÒüK²BZØ5ÿ¬ÐU[[ë%áÒüK²Biþ-..¯æ_Z¬Í¿gÎ	÷Õ!YÍàà`4ÿ¬àõz¥Hæ_ðIJÆÆÆæååE@ó/Éx"¯ùd<ÍÍÍ¹¹¹£´´´¯¯/RWdL»ÞÞ^íýyÍ¿$+`FmÞ¼Ye""IVÀimm"ÕápHÙ%kM²¦^yyyBB$k8ýK²¬BöìÙár¹¶mÛÍ¿$+`º´¶¶æåå9²²²HüdÌ´¾¾>mþÈÁHVÀÚ³gOJJJFFF6ÿ¬)ÓÞÞ®Í¿ÅÅÅýýýl$3øC~~~ss3doÿþýIII)))öì¡ùdOÿJ©ZRRµ½IVÀèëë«®®NHH(((hoog¬àiïß¤¤¤-[¶ÐüK²×ÜÜ[VV=cÿ¬$+L½¾¾¾ÊÊJmþíèè`DT²>Úáp¬0cöïß¯Í¿uuu4ÿFZ²/Z´dÑÚÚ/¥jUU­lLÖM6mÝºÕ$ë/¸ÝîÂÂBö%Lä¨þKóoÄ&ëõë×ed]áã'?ù	ÉT__¯cÿ2øC'kQQÑ¹sçþ¿¢5¦G»öþ­¬¬dìßÈOVÇo#Y`æßÂÂÂÎÎN6HT$«5b©Y`ÕÖÖºmþek¬$+Ïëõjïßêêj£7Y)&æ_d)c=ÊÖ YIVÍ¿$+ÉSæ_d©188Hó/ÉJ²ÀÔðz½n·æ_dÉêéé)..õx<4ÿ¬$+Ï4ÿfffÒüK²¬0)¦ù·¦¦ñôIVGó/ÉJ²ÀÔÚT*TIV¦À©S§Ün·Ä*Í¿$+ÉÒÓÓãñxüi6ÉJ²@¤6Ý¶mÔ©R­JÍÊ!YIV6ÿ:NIV&¥§§§°°Ðáp¬^½úÊ+ld lÜ¸Ñår-X°æ_dI9qâDFFÓéÜ¼y3cÿ¬$+¯££cõêÕÚüÛÕÕÅ!YIViþÍÌÌB²¬¼£G¦¥¥9Î-[¶Ðûd%Y xíííÚüûê«¯ÒüK²¬¼Í7'$$äææÒûd%Y`R´ù×årÕÕÕÑüK²¬¼ÎÎNmþ]µjUoo/d%Y H¦ù×ívsë7dI9tè6ÿJ¸JÄ²AHÖqq8ï¿ÿ¾N,^¼X&ZZZrrrJJJ?¾hÑ"@´éìì,**---eì_uÂÉºuëVÈÍÍcÇÉôõë×eº±±Ñét¬¢Çàà`uuµdªÔ^¯B²N@[[§ªNè½y÷îÝ+?oÜ¸¡ÉL²öìÉÌÌLII©­­¥÷/É:a###ZªÎ7Oõ7Þ9Ç·Ö¬$+h`zÿõõõ±AHÖIµoß¾]&Nç%KL²Jý*qqqkÖ¬!YD0©M+++òòòÎ9Ã!Y§ YýÖ¬´rËÌÌt¹ÿ¬ÓX³jÊÞºudÁºººåpWZZJó/É:½Éºoß>ý¨Ñ "õ÷÷oÜ¸Qz999ÍÍÍluÊõÜ¹sÚð+*eee2ír¹6lØpàÀ5+£?$%%1ö/¦>Y4_ÍëJ¾þâ¿ YD®®®eËÅÆÆ0ö/¦%YÅ­[·$G¥Hi¯×+Ó$+!µiUU6ÿ¶¶¶²A0]ÉzóæM	QÉN3çÂ2çøñã2-'tÚVlêW@8£ÛíNKK«¯¯§ùÓ¬CCCmmm¾õ«ªsíÚ5­bIVá¨§§ÇãñèØ¿2ÍÁ´×¬Üë@¤êëë«¨¨Ð[¿1øHVýû÷§¤¤$%%Õ××së7<d=öì#GHV ³³Sÿrë7<äÕáp|òÉ'fzùòåÅ£´ÓÉ'IV!®¿¿¿ººÚétææævtt°AðpõØ±c§±±ÑÜÔÔ$?ÓÓÓ%SM²^¸pdÊ:©Í¿ôþÅÃLÖy-ÉÎîînkýúÖ[oYñHV¡¦³³3???66¶¬¬Þ¿xøÉ*ü&ëÂ­5«5d:úûû+**Ng^^^;¡¬/^t»ÝEEE7nÜ¦fÊuì_nýKV½°*îÜ¹£9ºråJ¸ÿ¾¬o¼ñ¹É hï_½cÿ"U|Y~Ï7ïí·ß¶ýêöíÛ|ë@(¨¬¬LHHÈÉÉñz½lb²~÷Ýwù£ýµªªJÛIVW]]]ZZÓé¬©©éïïg tkÖ÷îÙ¾Ø*AËHB§rtòx<þ0HÖìQúÅVùìÆÇÇ7éùóç¬¾¾¾ÒÒÒØØX·ÛêÔ)6Â#YÍýÏÇªYmE-É`fÔÖÖºFÑûá¬Z­êWWÍ·Wåsl#HV3ÆëõÊ)¾yä(Äà³d½ÿ>÷º:$G%Mµù[¿!,ÕzµUNÍWnîÝ»'HV3cppæ_DT²¶´´H?Þ¬Ówk9×ë"Uèëëc U>ÐÇ;útQQQccã¾ût0&ÞµkLïØ±d0åLóo^^Í¿dÕõÒ¥Kæ²ëÝ»we-5+iBó/"9Y·oß.9züøq;w®L|öÙgÚ+Oû[è'YLÍ¿ädZ³f|¾oÜ¸¡ÍÂEEE¶âûö¬¦Í¿üdÕv`ùùå_êC­_­ßgåþ¬&opp°¦¦æ_Dx²^¿~=99ÙzV¿5+÷g0I§Nr»ÝRªÒüÈ¯YmñIÍ`jõôôx<9äååµ··³AÉJÍ`Jæßúúz£¶¹¢¬¬lÕªUåååR§Q³R³þxJó/¶lÙQ[[+êêê¤¤¤Ð¼gÑC¨Y/]º422B²®®.!:;;SRRä§ÓØØ­Ó¬ûöí3÷dµÕ¬ò³¥¥d6ÿ:N!êêêÊËËm3srrBð|kêuxxXs÷îÝS[¬@T9zôhffflllee%Í¿øvô»òa°ÍÌÏÏomm»ÈÚ+W´ù·  æ_gÎIKK³fI¦:ÎþþþOÖ¶¶¶GyF5þH§uh~&Yø¬®®Öæß=ö°A`SZZôèÑ®®®úúz	Úººº)NÖááa[#°DéöíÛ©Y<°"ÉÉÉ­ªª¢ùc(-,,t»Ý«V­:qâDh.ä´·¬ÓÞ¿©ÍÍÍl;ÀÃ´mÛ6§ÓAï_¬c4Ý°ao²¬Tkk«©			ååå!Ø	­Õ¦2ír¹´N¬@ëíí---]µjõÿÉ:ÞdLkðùóç-ZsáÂwÛ¶m3ìÌÌLzÿd8É¥ËwàÀ¬@øòz½yyy			UUUÉúÐÕ*11ñûï¿wú]±bûYW®..Öæß®®.6HÖHÖ¶¶¶õë×ËÄ^/¼ðBaa!ûARÖÕÕ¹Ð¼-	öÉ*ÿfA¼àÝ»w=Ï½÷hÂ×ëÕÁ***B¹÷oIIÉOúÓ´´´îînvB(YO>]TT¤sssM`3½÷ny7¡½yóæºuënÝºEß` õõËÿûªU«ByìßÔÔÔ9sæ¼ùæôä«455±*Ézÿþý)ÿFó._¾üöíÛ|ëµµµRÿ-X°àèÑ£!¾¨¯¿þºÄê7ß|cæ<ýôÓ²ðìDJ²NôôtÉ29ÖÁjjj¤ý~òÉ'm7&ëîî5k»¬ü¡°°0ï Yßyçë¾¾>!¬Re¾øâ$+Ùêëë222öïß^KþÒK/É[Ëë+WÊö)B:Y7mÚdóÝwß¬@ÄhnnÎÉÉq:ÕÕÕá8ö¯dêÜ¹sSSS¿øâ¦¦¦gVÖ#G°g*Éêõz×ÒÀd]²d¹zCCÌ·$Yp'9ª?È÷+WÂwEúúú$_~úÓÒ1¡¬÷ïß·Þö|ß¾ÉÉÉEEE´dppPÿ¦¤¤:tLc²úÞKîìÙ³½½½2àÛ¨$+F¼^¯Ûíò.L0NVëdN<)ÓÝÝÝ$+¾zzzLó/cÿ3¬÷ïßMNNÖ9bÂ5..îêÕ«$+^´ùWoýR?Èùz__;¬wîÜ9xðà¼yóÚÚÚtÎñãÇ%P/^¼(Ó·nÝª¬¬ñññòL§NÒæßÐ¹õÛ;ï¼3öl) gÍ5gÎzó"2õöíÛsçÎÕ;::$>oÜ¸!9úùç[6<<LÍÇ#ÿÅò³³³3tLÎÑ%P?úè£oG¿6óòË/óUDlÍ*$M½^ïéÓ§å¿Q>ñRÂÊDoo¯ªD¯LïØ±dBÔ¦R¡º©VCðÖo>úèo¾ióôÓOggg³ãÉ*ÙÙÔÔ¤ÉªT%¾$ëÅeâ­·Þ"Y¥Í¿«!Õük%ªmPâ-[¶0p"3Yõ2ª|â­Éª´o°ÌÿøãIV Ye:d3>>Þ6Ãë¯¿ÅDdÖ¬¦MX¿uóÚ¨µk×Êô¼yóøÖB¼ù×¦  @Õônnn±¬DZ²r¯ ÌXóïþýûsssNgfffUUUÐï500ðäOÆÅÅ=ûì³ÙÙÙ³fÍZ¹r%ûQ¬/^<ú´¹þzìØ1)]]]3Öü[[[¡qGGÔåååyÁO?ýô¥^zùåaQ¬ÖadÂ¤,É<tÚü+åãÌ4ÿJ)ï%jæôõõIR_æB=Y½^¯¤iKK>¼zõªvn"YîèÑ£lRGN¦ù÷Ä7o®¯¯à`©yyy¶¯¾ú*å&HÖ	áÛx:¾oC²b'ÓüÛßß_PPSUUURR"!½gÏÏÚTl¹zõêÆÆFvHÖñ¼aÃ¿q+ÿÕ$+0ó¬Í¿gÎä«I0KÅiêÝÖÖÖ¤¤$ùàO222öïßo­b%Cù»=@¨$ë½÷t°stsÛó;w.]ºT/¾^¸pÁz'WVSÕükHöööZçTVVnÜ¸1ÀHË_UWW:uJoï¸ÌHÖÿ¿É÷òåËzV¸xñbýÕ#Gä¡~ÕUþ©$IV`ºuuuN¾ù×ª¯¯OPÛÌúúúÒÒÒ.Lyy¹,OII	íÀ YïüÞïéô¾ûäáÝ»wifÀÀÀN§3//ïÍ¿ÍÍÍO=õÔ£>:gÎyþ7ß|øù)))¶n½Ü7of³Ó¬×®]³~ÙF¦Ýn7=0¡æ_ÉÝGyDõÐ¡C÷îMKK®¢999W®uuuòW¶öadï³¾øâÖ¹¹¹$+0­ºººV­Z%ÿn¥¥¥ã¼+xjjê/¼`óøã¯X±"ð_iA/¾`ÁÀÝuõìÙ³ò¿ýúuk²¬À4ÐÞ¿RMN¨÷¯¬¶uãÆR>ðûûû;;;Có8@D%ëáÃ%GµÓåËåaCCÃôÃD²"jËÓ©PKJJþê¯þ*##ÃårmÙ²e¢9'ÉÚÝÝmóÎ;ï'YÌD²Þ¸q#99ùÈ#¾·¾Y¿~=£SåÄ)))ÕÕÕÿ÷¿hÑ"ùê©§»Ò­Y¹×ÎÎÎýû÷:uª¿¿?ð3¥*MJJÚ½wyyyll¬ÛíþÍo~#ksssïkz0É9ñ80 Y&IY\,5¨Çã),,À£äwttdddhïßÍ7ÈLIÙmÛ¶·ÉYYY			©ãùÖ¬CCCã7Þ YÀ***-[fJÕ£GJ¸5Ì½ÌÿÙÏ~&ÿ¾úªùÞË·££ ¬Â#Yµ×ÒÅ­3¿ûî;ÑVWW»ÝníÁk÷ÒÓ£eee555¶?èøÔ±ì1kà¾¾¾´´4¯×ËF"*Yå¿ÚÜØØ(±*Ç°l½i+Éh#UfAA^$iFFÆ¡CÆ9X üûHvÁäônòRÓÆla ÒuxxØL;vLÇ2¼zõêthH²"]¹rEr1777//OªLmæ333S¯ªS§Né[$#­íºß^455k»û×:øË®^½zÁË-Û¶m_*"0Ymw¼Ñû³îÞ½&Y$ü$AKJJ$[[[$b%Ö¬ªª²=YU?ÖuVû766VJÒÀDx²nØ°AÒ´¬¬¬±±1>>>11±qL/_¾dEdrSbÕ:gÕªURÖÕÕÙlõË3¨Ú7X¦_õUí|âÄ=öHîÊt?¨dµ]LÍe8So¡éÌ3O?ýôã?øV£åååÙÑ©;;].WGG/)O6õû¬R°;wNÊU%¤øÝV¬gÏmii½téo²NÇ ü$+&ïÓO?äGöYÉÔ×_=&&fåÊA'k»u¥$«L444H¸VUUÕ××Ky*ÿ¶.ÁÚ2Ü°"*Y½^¯¤2A²"¼Høæoj³fÍ:räH/5Vk°NKÍ*Óeee7o¶ÝVÉÝ´´4É]õÛ·o÷öö%Y²¶mÛVXX(gGË¦¦&)mOû£?ú£_~9×«S?§-[¶LþA¤ç­ßDEÍj-^M²¦§§/°Ì#ÉHr.??ÿÌ3]]]R;¦¤¤H)µé9slÏ|öÙg_zé¥àÞåÊ+¬Þ©æ[7~É¯***¤b¦ù Y|sÖ7nÈtZZ$ëÈÈÈ¦Mdæ;HVÌÉNÉ*§ÓQZZªÙvèÐ!·ÛmýFé-[älOÊÄY³fñÅfþ7ß|#i÷ÑGMßÖÖÖjïß=öÐü¬ã%¬¬,Ö;¶¬n­­­.«®®Nâª§§§¸¸8//OÕwd]$¾111¯¿þºD²ª°¶Q¦ÊË-ð YýÐc	3IBKbÕ:§  @æHIj/©¦É*ä	©©©³gÏ7oÞË/¿<M±ª½%Sel]¬ÜE!*%%ÅÖ¸*©Y^^>Vkð-,,TÃf,~$+ÉÐòë_ÿúñÇOHHróØ±c:3--­··×ú´Í7K¥øío÷`ÚiSÊSmþ¾Ff$+ÉIyê©§Çc=%¡%ÓëÖ­ùÅÅÅÖoêÍd=jR¶  @WÇc,i:È»jóïÌ¤8dE0$ Mªßû½ß9×¯_0ËÍÍÚ´®®®¦¦FJØÉV8µµµ.Þ¿ÉJ²"HÎ=Û6Sµ²²òÛÑN¿jeeeUUUÖ;Ïü!!!¡ººÁdEÈÈÈxì±Çl3gÍõ«_ýêá.ähIIIlllaaá´6 YIVLÇ#jww·óë_ÿZæ´µµ=Ä¥:tèPZZZRR½dEø"uóæÍ2ýÒK/I¬¦¦¦>¬ò4''GJÕ²²2[Ïd$+ÉðpýúõÇÌñ£¬¬¬²£Úû×÷þqHV¡«¡¡¡¥¥%*))ÉårÉ½¬?ùÉO/¾øb(,Ukkknn®,Oyy9½¬£^N§óÊ¨¸¸8yøùqtü[¿ Yf$Gcbb¬sfzXË£Í¿)))z/vá¬Ç:gþüù2sæäÔ©S¹¹¹Rª¾É°LV[§ß9sæÌp²^¹rEÒ]25??Á¬o:Ô¾éÜÐÐ ]glêêêÒÒÒä·mÛFó/R¶¸¸8MY!Eä¼ï'´÷¯¬3óHV3ARí±ÇÓK)))3ð½½½eee:ö/? YàÖÔÔ¸¤¤¤úúz6Þ©S§Ün·öþeì_$+¼½ycÿ YIÖÈñè£!ïj>MLóoJJJ=½¬$k5kv^µjUBBLÏÀ@KGÍÌÌÔæ_Æþ@²¬ãW^(þùçÍÉWÛ©ÕÕÕ¥Í¿4ÿ YIÖHãwX%#Åë¿6ÿ:Î=ö°ñ¬$kr¹ õÑGÚ72Í¿4ÿ YIÖµ÷nÉÑ´´43'++KæüÅ_üÅT½Í¿HV5ºÌ=[-ýîïþ®éÍ4%¯Ló/dRZ§*ký:4ÿ YIVL®®®ÂÂB¬$+&k`` ººÚétfddÐüd%Y1)Öæßþþ~6dELó¯üìììd Yý¸páBZZZ\öÙ³gIÖÈðÌ3ÏèÍVçÎ;%/Hó/u¼<ÏÞ½ebÇk×®>V®²ýFÕ¿ÿû¿Oæ%Jiþ@²WrròÈÈL¥§§ÿý÷V¬XÁ¾qqq²¿¤fÕ»víÌÈû4ÿ Y'FÄ¶é!ë×¯§f¾9êt:Ç5| ©P¥NÍÈÈ8zô(É:^111f:>>ë¬¬¥Ö9/¼ðÂDUª««iþ@²NÌO<!U©ª2M²F@²ÚrT/»óÏiþ@²NÖk¯½¶÷ndwåååÚ ¬½åaFFÆÿp`` ¢¢BêTyòþýûÙHÖ µ´´¤¦¦ÆÄÄ¤¥¥?døhí&áúÀ?9tèöþp¥ùÉÊHðãþé>øà>­§§gÙ²eÀò³µµíd%Y$©M«ªªÌàl$+É 566¦¥¥ÅÆÆnÜ¸æ_$+Éàµ··¯ZµJ;::Ø HVA¨©©IHHÈÌÌd%Yñ[$#­O:àÉû÷ïw»ÝN§SÂU"­d%Yñ[ôQIÓÙ³gÿÇüÇóÏ?ï;@ÑÙÙ¹zõjùmQQQWWÉJ²ÂIJËeJÁjóícÿJº`ÁÆþ@²¬ÓîÝ»+TÛXüÚû7))©¶¶oÔ YIV¢ªíÂªÌyäG¾íý«cÿööö²¹¬$+ÌV¡¾òÊ+2';;»ªª*!!!''Çëõ²¬$+ÆëgÑ^K.KJUZü¡²²Á¬$+&L|P®þd%Y<©MËÊÊ¸õdÅhhhÈÌÌÔÁhþ@²¬×ëÍÉÉq8çÊ+l$+É õôôÇÆÆºÝîÀÃÉJ²âêëëSRRN'? YIVLJsssnn®Ãáæ_$+ÉàõööJÆÄÄÌ7ïÌ3l Y¼ÄÄDë­âtüB YIVLLsssAA	ÔÚÚZù;¿ó;zç8¶dÅxõõõhïßY³fÙîi7ÖXd%Ya·gÏ¤¤$ËU__?88hv_¬HVÖÚÚgëýë[³®X±d@²¬¤§§§´´466677×vë·äädÉÑ¬¬,ØÑÑ¡]ÙhHVþÕÕÕ¹§Ó)~ÐØXýã?þ#ÛÉJ²ÂNÊÓüü|IJ)Xþ°bÅÉ×Y³fÍ=XvÖ±Ù @²"xû÷ïw¹))Úû$+ÔÚÚª?HÁ*e+HV©···¼¼Üoï_É©««KIIIKK£ùHVuRÌØ¿ìý Yñ.+??Þ¿@²¬S£½½æ_ YIVÉJ²HVd¬$+$+$+É YIVÉJ²@²¬3ë¥^úù¶¬¬Y³f9,þy¶	¬Rll¬¤iLLLÿæ7¿ÑpÝ¸q#[HVCrTjÖÀs$+&¬¶Ë«Ú8ÌA&kVVÉ$+¦,Y­ekzzº<cËÉ`^KYdÅd=òÈ#³FIÍÊÖd¬$+d%Y YIVÉJ²HVd¬$+$+$+É YIVÉJ²@²²/$+É YIVHVHV@²¬d Zõüùó-ËÉÉ¹páÉ Y'%++KïÀ.$Y$ëILLkdff²/$ë´µµ­_¿d¬SàîÝ»çÞ½´HÖ	süHÞ¼ysÝºu·nÝ¢o0d,¯×»|ùòÛ·oó­É:ÒÓÓ$+de¤ÉJ²@²@²¬ddd%Y$+É YIVHV@²¬dddÊ[o½5gÎ¬)9þü¬¨ôû¿ÿûYÑJÖ]¶@t®»|æåëÍÿòðÀ¿üÔ¾¦Óéûd½víÚo¦ÚÿñË¿Ùo¢REEÃáØ¹sg®û®]»dÝß|óÍèÜõ.?ÿùÏ£sÝÿäOþäÉ'Îu¯¬¬ýßýÝßEáºñÅ²î¿úÕ¯¦öeÿáþapp0¼u:üÍßüÍ%K¢sÝO>-µÿú¯ÿÂu¿ÿ¾¬ûÉ'£s×çççÿõ_ÿut®ûï½÷³ý,:×ÝëõÊÇþ?ÿó?£pÝÿ÷ÿWÖýØ±c3óv$+ÉJ²¬$+ÉJ²¬$+ÉJ²¬$+ÉJ²¬$+ÉJ²¬$+ÉJ²¬$+ÉJ²¬$+É:û·ûÊ+¯Dçº·´´,X°à¿ÿû¿£pÝÿçþGÖýÜ¹sÑ¹ëÿìÏþlË-Ñ¹îµµµEEEÑ¹îÿú¯ÿ*û»wïFáºÈºÿó?ÿ3É@ø!Y Y YCÍíÛ·ÓÓÓÍÃ.¤¥¥ÅÅÅegg=6²×=ªV.Î?¿hÑ"YÓYë¨Z÷K.-^¼X×´¥¥%?ÿÚW1ÿ÷oÝºå°ÉÕÒd0·Ûm>mÂãñìÝ»W&vìØ±víÚÈ^ý¨ZYvºÈÊÊÒÑM8°páÂ¨Zw9jPÙÉÉÉÑöù*ó±ªuollnÝC9ôEi²®òêÕ«Ö¬üËÈÄÐÐµ¬HQµ²ìtÄÄÄè'OJÉmë¾iÓ¦­[·T­»Äªík63¶úQÕzó;¢jeÙéVmmmë×¯¶uºmîÜ¹²ë¥dªu¿~ýznn®dùØGÕ~ÏÊÊZ¾|¹¬¦lË/Ïäê¬ÿ'&&ÆLÇÇÇGöGÕÊ²Ó»wïz<÷îEçgàôéÓO<ñDT­QQ~iÛ|ì£óÿæÍÚc«EÉj½í¹¡¡!m%Ð¿U+ Y£j;ÈÁeÝºu·nÝæÏ)Ñ³îßÍÿû£3¶úÔ¬ÿçµ×^Û½·LÈO9¯ìªe§ÿ0:¬ÝòåËoß¾ëuéÒ¥F;HËFÎÏ¿ùØGÕºË®¿víVÎð®'YÿOKKKjjjLLLZZüFöGÕÊ²ÓEzzº­vu¿páBvv¶T«K.Õ=?ÿæcUëÞÖÖæv»e×H¸Îäê3R$+$+$+ Y Y YÉÉÉÉHVHVS÷oìp,^¼Ø3ª±±qß¾2ç«¯¾ðüüüüÀ¯©·Ë~ûí·õá½÷äaCC[ YHsúôé¢Q¢v_ýµüÔcYS°©©ÉÌq»Ý½Å£?~ìØ1yÂ/¾hæ|òÉ'2çÎ;æOZZZd¹¾æ#GØþÉDû÷ï[ËÊùÙÖÖ¦ÃÃÃÀú´ë×¯ËÃ7nÈÏ¥KêLë½Zµ­¬¬´ÍÖ7uðÊ+ez×®]2½cÇö@²@o2*º»»%ánÞ¼)?/]ºtòäI°%kZZ¦¯sçÎU&¤$ÍÎÎÖuïÞ½2çâÅ¶ªW^Ó¤øÝ»w­¯	d"ª¯×úRRPÛxïÜ¹cKVSêí¯µõXÜ«W¯þðãeZë·oß.3%teZÃø³Ï>9Ä2qøðav@²ãöíÛ6l;pàF£Tv2!U¦o²ÊÃsçÎ¥§§k£î~(3/_¾ºvíZ°¾øÐÐÐ5k´Yÿ¶¨¨ÈÒò:ìd"JSSéR¤×YwìØ!ë×¯×d=yò¤>SâS~«íÆzÔÕvàk×®ÙS*`ùùå_bWJUÓÝIK^v@²E3ÒãñhøõôôhJXJÙ*×¯_75¨>G¯Ëfff¥t"99YÂrddDþJ¦õù¦ ö­YõWHV Bè¥ÖóçÏËÏ;wÀÛ´iÓüùóÍÃó'«¾/uëÖ-í6<<<ìÔ¬ÉDÁÿ­ÃQQQ¡-Î;W3##Ã<ÇßÊÃ×^Í¤5&³²²ü¾5+@²QA~eúìÙ³.ÔzTæK²jæIõi.µjo&­G¿þúkù)OÓ&_)X75+@²O'Yûþùç2§··÷¿QSPP qhmÝ½sçÎ45É*+s$'Z³^ºt)@*$+°a ¼ÿ¾$bvv¶×ëà_9rDËþüùóõWò«yóæù&ë¾ûtEy¾­f­¨¨Ðá&Ø#ÉDîÝ»×<Ô¡>øà3çîÝ»¶²UwïÞMa¬¬¬dddd$+$+$+ Y Y YÉÉÉÉòÿ&sþ:ynIEND®B`


反趋势正态 Q-Q 图


U÷Í·466òm¯333m®¶Î¤¥¥ùîðìÙ³·oßÖD]]»7úùçýµ@¼s´éÏ=¢Ýà¯àvË¼7Óú£¤¤¤éÓ§Û¯£åüÚ ªa¯Të¼n¿nÝºÈ;¦µµÕV`÷_éÛ#	Ëþú3gÎü»xwôo¨O;;;Ãîß°I¤¬Ú¹6DÞ©½½½8ú´5­öÊj6è6ÔVrþüùú¿µ­çöíÛ#ü WV«Y7Ö½¹ío2ÔÏ¾¤Á>º®k:111ò°­¤Ó¹lh£æö6ÁÜÜ©*îîjåÊÁ ¶¶6ßvÙ~Û|ëK¾ÞØ"²ý¡ØÊjTÚqs®^½êÝþ9´ ´¹×ª¢OÝ¢³ÐLß·h]²ßW«ýËËËõÑvvvv«qä²*ÏsyoÅÍùà4GÓaËªyóæÙFÌZÎÚíÓ´VlïqA¾ïÏÕÜÖ¢aÿ¶Fþ]tëeeeÁÝ)ß½¹²zwmp¯Gnó; ¬ñ!¾3fØ¾Æ|ÞË+V¸6£1fºo<þ¼÷¨wäØpS=Ðv_Vß7oÞyCoÔ5cÛ|5ô~)Â>	éã=,EÞm-%Ðwà7>>ÞÆßÞ%3Tcb(«÷¼¦kjj1Ø´i¯ßútÃþÐ(Sb(«æ¿ÿþûúè§kñN2E3µ¿bÇÜ£²awäjC"õ#!!Á©ðÝw¨ßv4ÿÂ6.wcVM»]ý¸°Ã¬(ËªÒ÷Hlw¡°°ÐöÃÒoa¿]EEörÜMxÑþîVYßÒö-@v#ÜÀÑÿïÐÂ¥K4çÌ3Áß/ÍñjWV[óõg¡¬£PVí®Ú6ßÞ~jXØ¢WGGw]õýÏ»|ly;ç+«÷»lø¢ö#RSSí)IqßS7nÜð=©f?4¸UÛ,Ú°È¶´!6µúoDÁ4ÝF¾1«5Ï7d´«76ØuºgmÌê»õ2xoÁ=WVeLËjÁÞ-ë'|2uêT-OýVÁeu¶XìÞ4$òÞÆ7¼Öý[~4ÝÔÔ¤êSÛ3ÓÄÞ½Ãþ _Yíye;¨`aãç/ÑÑ`íQé>í8ª¦ÃÞÆVi·ïeû[úh¾Ø[·ô¥OjÇÂÝQ;míþ-%%%¾ùúÑo$Â/bû*Û®¬¶íñkÝ¶$wæe¥¬xeÕ@ÁF'Á1®ï0n_ëagIxj£Áö¾Ýóv3;bæJiÇB;¨ªéKÚÔº´wÌöw±sÜd;öµÛÇ÷.meìÉ'+Gý ïvSã7ð²ÙscVûvß@ÙwVä1«îÇýÖx-7ßÎJØ²Zü¼Êc_YÛÚÚBáµ`ÝO´atpø4«¦VJíXtµí²wHj6´¹wGíþ×®]kGòõÑvÒG¾²FhuÅþ.Öï¡Nt²¯Úî+ÅX+³ýÅoÞ¼éû®ææfí=h¹Ù·Øó«W¯¶¬zxx¡kWi¨5Ùö/^ìî¤¼¼<rYõ'°%£¿VfÍlmmõÞ²RV<¼²ÚÝÝÝúøÚk¯ùNvµ#fî÷GØWõ%ßÔ°GízOØPV;íFvdÒû,wg_²Û VÌ>Õ¸ÊvêÛÊ[)µõ´mþìyMWVÛêËjÄÒÈeµ£=àöövÛ)Ê²Úé²vÖ0_Yµ­·PÝ4þüèË¨jj¹bØ²ÚîÖï^]Ø0ÛÁwc;ÐÛÛ[YYiÃÊÿ÷ßÃsOÉÛyjj¤îDa³ó×ßb»kzÀnùØ.`ð¸®Û?³GâÎ¥ÒÊfGSµ¶L4IëªÛðÒ@Pÿ/vçÞ!~¾ªxëïîý·rDUÜv2l±x¿ÑvA¼qí×ºiû§¾7x6ßöýÝ)+eÅC-«¾¤M°÷Æ¾¯?^òýçk£iMr©°-¬µD[.¸WËxËêÆYãââlëc±anZ,-iéYÊª9zØvZ¦­âS9l¨áÕ@'o·aÞ£Á¶õlHá=ø9¢1«íUØñ@õ`Ø²zÁF³N0ÁûÚ$íD.ëÄÏ§Úºq^°¬úÅµ>x;c7xlÓ»÷`ËVAunÆê­	¾gñeöh°þÊZ úõ­ö,¦µFÙr¶é¡~_wìÔÑ¶ì(EØã·÷OÊMHHÐ*d?B?ÎVHMdeeé?Ë;Ìmhh°uÀÖ4ÛiÓîûírÏJ8ëw÷;í;t?kÖ¬°eµ#6z¶=-gûÏ¥¬ûh°û.ïn;Ôöô¥>øÀ¶ûúÇVÝÑ`(++»¹´Q64Úxyg]¸p¡½BÃUV1öç²Z	4`òþ÷""m¡lÚjkMØBÐ·Û¶8ô|¯ ²1´#¢y5lY­|öãF¥¬aG*QYÝK;Ü-:ßYZöêï«/v¿uðÔ0ª_ÜÆjvê¬µÇ!Ûi®Á´D_V­KZ´Êy(H÷Îúª)­¨ÐÊi!ö¼>ÕÛcËÏÏ÷tíëßÇ¡SSSµ577»'JlÍÚTÂ¯	ß|9; måv§Ù[Vûul·Ó´w¨Ço¶]Cï±zPV<¼²ÚyI¾ÃYÁ¶¹¶M·³9ìPgð$áà©RÞÍ¥6v6Bü<«~WåM?µQ³9v&½4P÷¦mÊ%K´­w3Ý&Éni×àÅd]qã-~y·¼Ç¬Zì¾²Þó<Ò£ÁöJ_Yõ°íØ`E_ÖàiDÞãÁØïëRî;fuÁ³ÇöÅ'#³º-k;W«Mð9r;tl±±«¶Îè£íø.âëZ°vÃËÔ»#:aju?ôµ×^ó~êÝ÷rÇÝ+ß| Èeõí ØÓáfÛeÌJYñ5,m¼ì¡Êêûÿ÷m.eµCv¶WÅ]ÉÜO·#lngÏ»£Á999®a¾óA¼ÏûÊzÏsF=M¤­°:Ôap_YíeËy%ö(º¯¬n	»aº¯¬öÄª=Ïª/ÙÓ®1ÕsÞWV;¼oË¶¥¥Å3[Ö°cVw´?ìõ1FZVáÙÑi;|í~qíØ|w¶Ðì·s3´¬ì¸®ývuíc¹§'ô'a#ßµ;Zàöbqï¹ÊÞÇxoã.Fæ«i4GÅÎc×=PVÊ[VmVÂ>%<lÕ¾4ÐûT¥ïÒ£XV»ÄOðøl«aO,ùëõå¾¶ÇWV®ùÚé»ßËQ"Õ;÷ï;ÊÝÒ:1Ô|eÕF||¼;%8...úçY'èÚ©m¾óKUVO0AÒÎ>Ú!G%sDcVwmeõ¾Æ2ï-ÒÝÖL;w×.Bä2ÞÊjçè644(Z´b+½ËÆC÷;ìµ½Ü-ç7DN]leµBÛiù¾«IPVÊiÓ`Jw¥m=mBB»&½Ð3øZº°g0ÙkøÜ Ð·]öY¤ð£/kÿ CßWí:ïé¬Áºbc¯¨1vâo¨ä+«~£»B¡ïà½?¾`Ãûto4euÛ5ïÕ-ýÛzæ+kXzö¼¦w`ÝÜÜ¬éW:uê^Ïê®qØÞÞ®Ý/[°îêÞ²Ú~n£ûWuì¨¾_¾¬¶ØÁOïe æÍg_ÕOw'¨'&&ºçwmXæßkg¯ë/Vå³Ú9vI»¼¢+kð"ÞjÌíeOÿLödjä*ûþ@Z=ôG±ûÎù©gý½'ÙQVÊØEx'ÝêàÕÎË^ó(æ£ÁÅÅÅÚ²0ÈmCÝY¸ÚÌÙùJ6¹j«f[;Ý&lYmo×Uùltâ¦u4 ´k$Ù9IÛV=;ªfw¢ç®0mû4Ò£²o`AvXÒwt:!=s<Ô/?·»t­ïx;Ì÷Î¡Nº±]:Ûcsû[Þ?¦ «¿EËíÃÙóîéaßFÊ¼Ñ>mð5*ÞÇæ½§vV´÷é]ÚÛðL°ý"®åéþ;¼×ÔÔÎpnëq°²ZóÃîæúþsÝ[x_äÍNAY1úÇ×î·Íwðx¬Bå&Úª=Y)øö2îyÐÈïlãø¹¥Ù1À2môàWâugy)«---ÒøÉ÷æ9*]ÇN×²'í»ÞÁ´=cÜ>kïïå#÷¤²-7[µü×6RS¯8¿Òiêb¿QnµÄÅÅ¯êl§/³åCûUí2®	ÊeÊe²Êe²Êe²Êe²@YeÎ?L(ôþûïçvmÖ ²?:räH½n¹aÃwÕÛÛ;qâÄùóç/ÔØØèÞLÞ¹|ùrñ wÏâ¦u»`&...11QçÆÞù>Qþ¾Ñ?RCC7Ê¤·¬µµµ¾Û÷õõÙDjjê´iÓ¢ü)·oßÖ½zËº|ùrïmúûûlZºÁë¯¿>Tò]/õø5ýæouèEÿPV`H®ÖeÏÅo×®]úxâÄà7ÆPVïýèÓÃe_¸p!rYía'$$ÜY£(+0¤K.i¢¥¥E-éèè<f½²úÆ¬K.êuuuCçääÜºukçÎg²&&&VY£y(+0ÌøLÙÐÇÍ7ß¼yS]]]¾²ö÷÷OäÇÇÇ+®öL¤I3Ýí¼s;ÖÛÛë»Y[[;"­¶effÚü/j~vvv0Òtiii²Fÿäke±råJoPcVwÜxDcÖ¦¦¦EØÈuÊ)vKM»oTÆ¼÷oCÉ°¥,))éîî¾ÿ1kôe¡Ñªýñ ;ÊzÿG­Ç6Ôj:aÂ·OKK1c;)×õÁp~þùç			Ó§OWöFeÌå@YH._¾¬rlß¾]½ÓQ|Uw¥­!²&>üðCK¸ï¯½öZðÛ½½iüè£4½jÕªû³FÿPV`HwïÞU6.]ºtçÎMìÜ¹sÔËÚÛÛkñÓÈrØ1ëHËª0k§ýÊPVàø¯;æÎËË³Áesssð[Î9£ùÑ_9ÁZw®éüü|ß858Ümjj*6l7oÞhuØ²á]½z5ìü¾¾¾åD#Ú°7°çb÷îÝùþí¬Ï?ÿÜÍQSÕ×ÊÊJ«¤¤D_:uê­[·¼a[´hQ°vÞ;yÐ»ßì(+PV(+PV(k$6mJKKËàð§ú§¿ÿýïï²þò¿9sfïÿûa_ÕýõoÿöoÿGÀk¯½FY ¬@Y)+²RV(+²(+e²@Y)+²RVe¥¬PVÊ ¬@Y)+O@YC¡ePÖQÐßß?uêTWÖÏÞxãÊ ¬ÑZ·nÝ¦M¬¬_ýu(ÿøÇü-ux]]]nÌÚðóÿ1+²F¥¤¤¤¹¹ùÏ³(ëýóõ¥¬Ê:jeÌ ¬@Y¹R¿%²RVe¥¬PV(+ePVÊ ¬ÊJY²(+e²@Y)+²RV(+²(+ePVÊe¥¬ÊJY²@Y ¬@Y)+²RV(+ePVÊ ¬Êe¥¬ÊJY ¬PVÊ ¬@Y)+²(+ePVÊe²RVÀtðàÁ	&dggWTTtwwSVÊÑÇR]]ÝÛÛîÜ¹¢¢¢×_²RV@ÒÓÓ=ê>ÕU#×ÃSVÊ±+W®¨¬¾ë×¯§¬0b½½½'O¾~ýºwfyyù;(+eÄbîÜ¹+V¬p~ñÅÉÉÉçÎ£¬K.effÎ=[ãÔÊÊÊÝ»wÖSVÀXtåÊ7¯Y³FcÖQ¼gÊÀh¢¬PVÊ ¬@Y)+ÊJY²@Y ¬@Y)+²RV(+ePÖh]¸paÚ´iñññyyy---@YïZ__¯	=ÊÔÔTM¼PPPPTTÄß@YG ¡¡!??_*.ePÖhõ÷÷'%%B¡p4@YGGccã¤I(+²øøxÊ ¬÷%''çÂhmm5kePÖûrúôé¼¼<VgÎyãÆÊ ¬@Y)+ÊJY²@Y ¬@Y)+²RV(+ePVÊ ¬Êe¥¬ÊJY ¬PVÊ ¬@Y)+ÊJY²@Y ¬@Y)+²RV(+ePVÊ ¬Êe¥¬ÊJY ¬PVÊ ¬@Y)+VYkjjB¡P?ePÖQ(kFFFVVVØ/PVeU«V%&&®²Ô£¶¶¶ªªJcÙÊÊJÊ ¬# |N6çYuÔÊ:sæLÊ ¬£PÖ;wîÕÖÖúoÚ´Ió;::(+²@]]]___p~½ÊÚÙÙIYõ¾Ø)ÁöjÞÞ^Ê ¬Ãkll,ä;üÁØPUy@Y£r÷îÝaÏl¢¬Ê:jTÖ¥KRVeÝÕ«Wsrr:´jÕ*õìÙ³@YïK¿ªÊjss3¯gPVÞëo®¬mmms¹sMïß¿_WMSVeöØ¯ïmÒÍ73fPÖQ;%²(+eàÑ(ëÀÀjº|ùò`YÃ^O²(ëÆ©NNN¶3l²(ë£ÁÊJY ¬PÖZ[[§NúôiÊ ¬þ²èÏsrrìñ8p`Ê)0¶Êjo~nç¸óÝtUU>Õ4;OLLÔÇ¿HII)**âo	xË:ìßÏå,Y¢÷mÊx2ËúÜºuKCÞÛ·os4@Yï×µk×/^|ãÆÎõ²B¡9sæÜÏ=455Í5«§§WÝ(ëÿuÝºuÞ9wîÜÑ=ddd<(+`UãËyì4à¨Ó§Owo¾wï^ÍÑäJÊÕÁÞ÷<ß¿jjjIIÉ~"²Ì²ßHîÄpþePÖ¨auO644hº½½²(k,ÇÕÑÔÔT»úR]]k||üÅ)+²FåæÍ555'Nlkk³9GQPÏ?¯é7nTTTèÓÝ²(k$===IIIvuÃ3gÎ(W¯^UG÷ìÙã½Y?cVejÚÔÔÔØØ¨¦vwwk«ÎÎNUéÕôöíÛ)+²àÄ¥cÇYYí)UÅÕæ«¬çÏ×ÄÊ+)+²ÏFÕPÕ[V³wï^;7Xó7lØ@YudÇíU7Í?_Ó'NäU7Êú ¬ÊJYu$Î?ßØØèNkª¯¯§¬Ê::W7Ô«,ePÖXÞNN5mii±O/^¼hçSVe1»"ï¥5âe¬ðäÕ.Ç¿|ùò°¹---¥¬Ê­Û·oÛ5æ*ý£ÚÚÚ;wÎ9Ó|=ú´÷Ò)+²yÖRGG½í¹:mÚ4ûR]]>µ+H¤§§«Á@YGÚ÷ßß¦÷ïß¯OoÝºÅÑ`eÅåË½/¶Ñtnn.g0(kìÖ9sæx?-(( ¬Ê'N(¥]]]Þ²PVe±C©£vSGG>Ý»wï½e<e½zõjjjj]]]ðå,YÂÕ÷ºàQ*k__¬.¤¬Ê:ìÖóçÏgÞ¹s²(k´ïrã.mX[[«¬&''kÂ¥CïÌQVe¤¿¿ßM×××Ûu.^¼ø®¾DYOxYWç·÷«ªªzp6¤¬1QÖåË«¦K.­­­MHHHLL¬¤éY³fQVeß©ÙÜÅòóó)+²àÒêhKK"záÂ`YGýÂðÄµ©©É¤ ¬Êz_eíéééììt¥¬Ê:jg0yË1eÊöAYTTDY57»zõª¦ÓÓÓUÖuëÖiæöíÛ)+²ÆNeÍÉÉ±iw9Ê ¬±Ó8µ¬¬k0(+ï"À#PVR-Zä>=ö¬æ455QVe±k×®©£mmmÞÐîÛPVÀ(k___eeerrrYY]àðÀöæçî-ä¼ÃYÊ ¬CP8kjjÜPUw­.ô_ß|óMÊ ¬Ñ>Ãªqª>räU«¹yófÊ ¬±5++«§§GwïÞu3·mÛFY5Ú²æääÙhUmfZZ»Á-[(+²Æ2fµ^ºtIÓ­­­6sÝºu@Yc/«ô÷÷»6m¢¬Êz_eõÞà£>¢¬ÊËó¬õõõÁåpn0²ÆøzÖ;wÚ¡à´´´ûö-_¾Ü÷"Ê ¬CRAÎöövûôÎ;Þ¯îÙ³Ç¬²(+ïueàq/k(Z½zµ÷ñ¨¿Ò²ÆJYír6lpsÎ?ï_9Ê ¬#á&âââfÍe¯À9sæâÅí¥8---@YGðÂýû÷[bçÍìîH¯¬ApFFeÅ²ÖÔÔ¸gUC4N-û#ïeú£¤nnn®¾ËÍù?ÿùÏ)+à	,«¬óçÏ·7<·²VVVún3Ò·+..¾xñ¢+ë×_çÇ?þ1KÀVV®££C÷íÛgÆÅÅÕ××»a«&ªªª4ÿæÍ#½g7ýEÀo¼Áð¤õÚµkÞgRm4ÙÔÔ¤vúÒ3õ©¡1çYceÌê;G)BY/_¾LY5Ú¶µµutt¬211ñ>Ð´´´U«V%%%­^½ú>?ü²®_¿þÅ_|á~úÓ²Æ¾²êã3gôñØ±cõ5''G?ý©§zöÙgíãöövÖÀ7PÖ®®.¬­­½ÿ²~SWÐÃ¶oª©ãÆSeYoµ¬¹¹¹ÝÝÝ6­²N8qûöíUUUy÷îÝÇ¢¬Ï=÷ªÞ9ë×¯×ãg½<Ô²º«,iÚûÖ¾¾>÷Ç¢¬/¾ø¢o&e<Ô²]»vù.Áï®'ü]_Y?~¼oy7õð°ËzçÎ¥táÂöéíÛ·õé=ìKKYÛÛÛõ°¿ýíok/Aþä'?Ñ§?úÑXo©¬ýýýÓ9r$==É%G)H,ÐÄ¾û4­ú¸¼×Í-[4HuQ>:+à¡Y¼¡UvíÚuïAzWÐàõ³Ï>cu|eíéé9H#ÔuëÖ©¬-²ÛuíÚµ¼?+²@__;k©¸¸Ø÷ÕÞÞ^Ê ¬Qinn?È½«¯Þ÷gÝ¿¿]É²(ëF«6`6mÚ½²Ø²:/_VY[[[)+²ÞoYíÕ«î=Ï½g0ÕÔÔPVeYV«ªªiNlïvNYõ^ww·"ê»G_Yûûû)+²ïîÝ»*h__îqÎ9îáµk×j~yy¹;a8òû­RVeõ_ãÐ%&Î`PÖÐ½ÇePVÊ ¬Êe¥¬ÊJY ¬PVÊ ¬@Y)+ÊJY²@Y ¬XY/^üÜsÏ%&&fff¶µµ±(kìÒÒÒB¡Ê§é=ö°(k£U¥tË-nÎ³Ï>;nÜ8V#eÅs¼sÚÛÛÕZdMPÖKLL|ñÅ3UÖúúzÖ$e1eÕwì÷g?ûÊÊj ¬±°c¿ñññvÖ>é¥X5FmmmO?ýthÆ¯Z|¬CÊe¥¬ÊJY²@Y)+²RVe¥¬PV(+ePÖ±XÖ¼¼¼øøøñãÇûÛßþðÃY¥²RÖØ%''7îþáª««_~ùåP(¤iÖ* ¬5ï½÷RúÿñnÎìÙ³y#t ¬5F¯¼òÊäÉ3ÕÚ¶¶6V, ¬uÄ¦OêsíÚ5õ¿þë¿X±²RÖûÝï~§ê£óòË/ÇÅÅ±Ve¥¬12eâªÁë/~ñÉ'kúg?ûÙìÙ³sssçÎûé§²e¥¬#óÆo$%%=õÔSßùÎwÔÔ£G~ùå[·nUk>ÌJ²ÆB#T¥´³³ÓÍ9xð`zz:+PVÊRW¬Xá©Ö^¹rõö½ª««×¯_¯¯îîn@Y)ëvìØ±lÙ2ïÞÞÞë×¯³Á`ãí·ß~÷Ýw_õÕüü|v¼ÊJYtîÜ9uT½­ÍÊÊ2eÊüÉäååy¯)±9ZMOO¯­­us.]ZRRÂ(ëCuúôimâããU¦'N<âWäß¸q£­züøñÊÊÊÄÄÄP(¤¬þÍßüMRR¦ßï=Ö¹1«ººZ£UïXµ7ÖÛÛËÂ(ëÃSZZºoß>Mlß¾þüùØð£ýHÝÝýhø§ú§þðùùù?øÁÒïÿûîKÙÙÙ³uëÖÝÞzë­9sæøfª¬Ú!cáO¿ø¿x¤Ë:00 ¾¾¾¯¿þúhC<é²ªa§ý÷g]°`Á	|3ãââx§¹1«··7??¿¢¢Âÿvvv®Y³%p4ø¡RÜtBBÂcTÖºº:ß;álÛ¶MsfÎ;öì²þ5.]1cFff¦ª½Úòòrd(ëÃ6iÒ¤¾¾>;¬éÇ¨¬òï|gÜ¸qvÖÒ/~ñMkØ]]]ýå_~üñÇ'OÞ±c«àØìëÉ'½@YTUUiBKKK¯²F'ªªú¨ñ·÷59Ú¶&&&½üòËÅÅÅ(ëÃÐÒÒ¦&¥§§·¶¶>veu>ìåâ-[TÜ_|qÑ¢Eëøñã7nÜÈ	+EDåèÑ£sçÎu^¹rE»&M:uêÍY³fâÚÞÞÎJ	²ïúõëÉÉÉMMMnÀ¤¸÷_ÿú×¬@Y)kTª««'O¼~ýúãÇjÌª	ï²³³õ«_±Re¥¬Ñúâ/.]ZTT4öìqãÆýáp_ºråÊøñãy)PVÊ#P'Nh§ÿçþçóÏ?ÿÝï~5(+eQww÷+¯¼¢ë·¾õ-ÌËËãÅ²RÖQ «÷°ëëë»ººX5²RÖûõüóÏ»:?õÔS¼(+e½ëúõë5ýÙgÅÅÅ?(+eRª¬îÙ³ÇÍÑUs/^Ì:²Ø;ï¼3nÜ8ßÌøøø¿üË¿dÊJYG¬¾¾^#TßKjmii)ë(PVÊuôgqj´ªÖ~öÙg¬££â«¯¾*///((xõÕW+**¼'ce2ËZ]]mo6§éwÞy'ò·;wNßuüøñë×¯³*Gðå_N<¹²²RKìäÉEEEsçÎå-ÇPÖ'ÿU7]]]Ó§OOIIyé¥"V5ä*++KOOûí·_ýueÃw	bx©£kÖ¬ñÎÑàõã?fÉ ¬OxY>ýôÓW^yåùçÏÉÉñAYUPÝPõðáÃêñ_|Áö<|Ãú­[·®X±%²¡²nÙ²eüøñ7n|ë­·âââÝWÕÑ¯¾úÊûeËùêoYWüíoKYPÖ±UÖoë[Þw;yòä¸qãêêêþð]q233·ß½wyy9+tXsçÎ­¬¬töööææær4eCe=vìØÓO?íùòË/ÿÝßý;wÎûÕ²²2»~Ó7®»»ûÓO?=xðà3gå©ñý÷¾÷=-¢£GVWWh3PÖ1TVM½¯À1ý×ýÓþÔ¦Ñüü|w@xÇéééßø#?uêÉ3Ü	VÈë[®_¿¾fÍ^KJJ¶nÝJVPÖ±UÖ+W®¸c¿nN||ü¯ýk7çÝwß0a­Ù£púê¥£n¹ÁëìÙ³.]Ê¿ÊJY¿yvÖÒÆmæÿ©d'O<wîÜ#2«®®VJ½s.]ºôÜsÏi@ÕïU+Ã¹¹¹ÚEÐGûe;úá8PÖÇÞ¯~õ«§~:i´úÊ+¯üáxÄ°Ò¨´¯µãÇÿÇüÇÝ»wjDë=¥¹¢¢BAU85ÝÔÔä«vÞ~ûmíRN<YÍæáPVÊ:¶¨£EEEîSE455599Ù©ÿå_þåõ×_w_U5½Q¾5xu/<µ`XM5X///õÕWy~e¥¬c¢égý×ý×^xÁû<«©Ú1íÃ«¾1c=a¬ êÞAªª!¯¶,g²!Þs5Zé¥|Ï+½6N=zô¨ïIYÉÍÍÕ=hB-,,ôU÷ÉPPVÊ:æ(¥Ç¯®®Ö5??ßüV½TwÝwÂ	ÞË<xP_µÛ«¾ÁKêÞ³ ¬õá)--öÙg³²²>ü<$;ß~ûm;¨æÌ¥qÇî«¶¦¤¤,]ºÔ. ¥i;Éè½ÏÑêÛy÷7²>$¡ÿKv~off¦>ª¾|õÕWë×¯WVõ¥/¿üÒ÷½öjÝ²²²üA¾e¥¬ÊøñãUS÷É'ëSaGØÙÙÛi½§Nª®®njjâ¬`²>ÔkAAwÎ¸qã[Öo ¬5Æ²úæ$&&g(+e¶¬¾'V5'..õ(+eÅ³Ï>«Î3Ç>MHHð>í ¬uÄ4Bõüü(+e½/.(((--õ^õ@Y)+²RV(+²(+ePVÊe²RVe¥¬¾îîîS§N9s÷_ÊJYï×Ö­[SRRfÌ~òäI	PVÊ£êêêÌÌLVíÓ?þXqíììdÉe¥¬±(((8~ü¸wNYYÙúõëY2@Y)k,svïÞ]^^ÎÊJYcë5kÖ°d²RÖX¼ûî»ÝÝÝöé¹sçRRR¾øâPVÊÞÞÞÒÒÒìììÊÊÊ¥KN0a÷îÝ, ¬õ¾|úé§7nüíoêÔ)PVÊ ¬@Y)+²(+ePVÊÀVÖÊ ¬£ ¥¥%777QVeÅÅÅ/^ôu~@vvvQQKeePÖUV(ë;j(+²2fPVÊÀ*+WPVÊ ¬ÊJY²(+e²@Y)+²RV(+²(+ePVÊÀX,ëÊ+yæQ¨×w¿û]CþìÏþ,33å¥?ÿó?gqË!úÅ¥ÉÑ½Ï	&<öe½|ùò¿¶üä'*ë¿!:ÿ÷?qâDC~ùË_B!CV¯^­ÅõßüEM6iq½÷Þ,hüîw¿ÓâzçwF÷nÿýßÿ½··÷ñ.ëðÏÿüÏÓ§O¿èlÛ¶í^`9D©®®.ò[Ã«­­MëÚµk,hrEë÷¿ÿ="ÿó?ÿ£ÅU__ÿp~e¥¬²RVÊJY)+e¥¬²RVÊJY)+e¥¬ ¬²RVPVÊJY)+e¥¬#ñßüæ­·ÞbµÒþýûøÃ²¢ôÉ'|ïßc9DéÜ¹sZÝÝ,hÜ¸qCëÂ,hhqöÙgÇe²@Y5§ONOOÏËË;qâ«BX­­­S§NÕRÊÏÏ×c¹E£±±Ñ¸ÄâàîÝ»/NHHÈÊÊjjjbqKK)77×OKKKl(===¶öz¹Ý²îÛ·OÛ·o?>ëbX999vUÌL2å6¬þþ~í¸²²¸"X»víììl×°RSS»ºº4¡VXö9´ÿá=-?¸ôråuTÿÒèëëóîÝ`(,·a­[·Î.èÊj6,ÚÛÛù¯ö?ìUIúhû",± âââ/zËz¹Ý²ÆÇÇFXmmmK,a¹E¦DAAþcÝ5+òÿà-[´Ç¦H=Å5¬Ó§OkqiíÒGýK²Ä"µÍSÖàRzÐËmì5..ÎM'$$°"FpëÖ­ÒÒÒÛ·o³Ü"+))innöþW³¸"ÿîÝ»WÚ#aqkÚ´i6ÊWbí7,±hÊz¹Ý²N4©¯¯Ïhq(×®][¼xñ7XnÑü3±¸ýôXòYb15¸ôr»e]°`AUU&ôQ2VÄ°fÍÕÓÓÃrí¿ÅÁ²eËjjj4qáÂ3f°¸¥qª]téüùó¿²Ä¢,kp)=èå6vËÚÒÒÞÚÚÊVFFoÆrÑ5+7ohìUPPpñâE×°.]º¤ jé£¦YbQ5¸ôrãJPV(+PV(+PV(+Ê(+ÀèýBÓ¦M+T[[»ÿ~ÍùüóÏ#ÜÞÞ[&ÿöU«VÙ§·oßÖ§övª(+ðDill,¤ªvgÏÕGK,o;ææäææ*½e9R__¯Ì3ÇÍùè£4çæÍî[ZZZ4Ç½Ýg]]Ë ¬ÀæîÝ»Þaå3gô±­­Í&úûû­°Ý¬««K^½zUgÎi3½ï¹k£ØßØ*îÆÁÅÅÅÞµk¦·oßÎß ¬ÀàÆ6ÑÞÞ®Â]»vM/ÐÐÐ îîn_YÓÓÓ50µ¾&%%iª	IóòòlÀºoß>Í9þ¼oÔ«ût¿uë÷>PVàÉaCUõµ¦¦&!!ÁÆª ã½yó¦¯¬njïmGmkï7nOÓzo¼yófÍTt5m1Þ¶mæX5qèÐ!þe===Ë/Wá8`iÔSµÓFÁ²êÓæææ;¨ûájfGGGZZÚüùó5á½ó¾¾¾yóæÙdûÞ_¤u?üÊ<Q;æN)²çY·oß®%KXYìÊ§¾jÇí)R÷$«¾|ù²/ëã'|â»ªºÓlÈË ¬ÀÅYZZjñ»té%P±Ô°U]]]nj·±çe³³³²sl"55U±ÐwiÚnïÄÁ1«	eöTkkk«>îÜ¹ÓoÝºuYYYîÓîîn÷-ÕÚÚÚà]Ý¸qÃNîïïæ1+@Y1ð­X±Â&¦Nª¤¤$333ÓÝÆ=ª¯êÓ¸@z3öG0f(+0&ØßÞÞ^M8qbÊ)6Õ|Õ§Ñ§ªÕÎf²ñèÙ³gõQ7³C¾°F7cV²O>;;ÉÎÑ=4§³³óÞ_QSXXh9ôÝ½yó¦ÍTM]YUQG:f½páB*ÀcÃ]ðîÝ»*b^^^SSÓ°ßUWWgÃQ·ÏÊÊ***²/uwwëK'NuÿþývEÝÞ7f]±b]n¿@Y1gÊ)ûöísÚ¥Ö®]ëæÜºuË7lµÉUUULÊe²Êe²@Ye²@Ye²@Ye²@Y ¬ &ÿ2ÓíÒF#ÍÈIEND®B`


gòóó3¢££ïÞ½äî;vL~Öï÷Û)((ÐéþþþõæÍuzll,Èb444ÈÕ®bßÈ®]»&úå»999Eã~d.z½ÞÜÜÜÿGwîÜ[ûí·Ýß:zô¨,êúð¿Â1'Ä~äÈ¹r[[=S¶ûWTTTÈÿW&uæÈÈ½·ûfzzòÇ"ËöðáÃò³eeeÁ-×¯_¿ïß¾uëÖÝ¾û&zT[[k;ò«Cÿy<÷RuÛ<ü.4¹ÜaÖS@°mÛ¶àWÓöÍ®.O8!ëÖ­3ÏLÇíÈøøx:uêÔ=$xzS²Î:ö¬°víZ¾uëNÔÔÔÈº#`ì_'¿]º(?[RRbîLff¦LèÊÝ½9rgtº««K.ÊÚ6UcÜ· Á°WUrñ>°/.0Äfæ£>rK$KU6 V³Wuuuò]Ù4	òG_Ëwå6CùÂqõujjª®Óå«>f7xþüùÛ·oËDcc£¹5¹øÕW_üeØsäa&ÿî°6ÜÙ,³¯&ê©§ôÏåüÌ8¹(¿7ÈV©<æåú÷î¾a¡:::ôl-ô¿¿råÊà½1$OC¹800pûU"eÅiCð5TsxxX&Î;§AlOÔ$Y¡ë8RÖ6lç­®=+++ü"SV­,s_YnÍ¬§£Fxú-ÈWÓu	¾t-)Üq&²R3[î`/kFF¹(õù|æ¦vîÜéÞêììt¬õ¯ÐÕ·|ËÑ]Dº=YYuJ¶`Ìk×®ÙÛî,(YÝËCE.E§· 3?"%ýå!¡ÿÁÒÒRùªw;---Äqð²J×³¯ÅÌyóÍ7eL,«L¬_¿^'dÄ,ËY6ûdZØö~qû÷ÊE÷ýGè£4øß"7(ãr¹fqq±sÊqk¦¬ö&²îåë|ÇF(+ÂÞÅ·bÅæËÏ~ZîØ±Ã<Ù9d¦ùÁ/ÚDí¡ÃMé¬÷eÅê¸òÁ¯è52¡AMgVß&íoÙÑ§#!ùzôèQM½®Ñ 	tìøõz½:þ¶ÌD ¬ö>y®¯¯wÇàÀ~ËÅ÷Þ/È?Z%ÃÊ*ó_ýuùÈoÅ»lÙ2)Û+º¿ÁÜ+fÙ#WI?¢££u*¾võëÌïîîÖq¹³Ê´ÙÔ_pbY¥ôön.äååé6b@òWè_W^^.[9fL|YÿïZYÇÒv,@ì¹!Ï)Ç®¾¾>ÓÕÕåÞÖqü!¡ì¯6eÕG¾ü[Ý»a(+eÅU6WuBVßöN?iXÀ¢­··×Þ`:óf_[vçeµª¹¹Y/òUERR¾$)ÌÀÂñÒæàà ãE5ý¥îµÐÕ¢þÝ¬«HÈªV¾feeé=û`ß &M®#1«6Ï1dÔ«Fìæ`³Å®½tßÄU2&ËjãÆvõ/¾ÈÎÎå)¦V0HYÍ.½5Ù×q¯åö5?2ÝÚÚ*)ºe&ÕÕÕ£¬úº²îTÐ­Àñ3¯µ7X¶¨ä6u?ªL¼>¤MlÌ¶noÉWÝbpÄ^»%ßº7þ¢©lX½;ÒNt¹ÿÈÂÂBÇ|ùÕ2_ïI?D·ÿdz¢l²êÖû/mÝH2GNPVÊXV(èèÄ=Æuìæë7Xô(	ß©¬bd°"[ßæõ?½î13¥ÔcBÝ(UoÉªÖ¬ í1kÀ¿EE2u/tÀÔfß^²Ñ´Mãìõ¦WÌÀKk¤wÌYõÇeÇ!ZÁÇ¬r;æoÔÆËrsl¬,«ÆÏ¾Wöeíììd¢k~££ÝÃ_÷ Yjª¥í®,FÝ¥lIuÇ¬îÍÞ`½ý=öè|ùª;Ü;iÝ#BGY4F»¢ÿí÷D:éwusÊOc,fýß¼yÓñSmmm²õ ËMD_wØ½·fÕÞáaÿ!í÷M¥Éºe°eËs#¥¥¥ÁË*ÿ]2ò³Ììèè°¯CY)+^YõHE¿ß/_yæÇÁ®ºÇÌìa³~W¾åØpo°îp3CRû eÕ½Ùfd &íWÑì~KC®ZdHÀô¢«t£^ò£ky-¥¬=uÅ¤«?]ÓU×zî²êq¤4xYuå¨w¸§§GÇâúo±¬z¸¬Þ6ÌQVY×k¨nÛ°aCèejÊ3#ÅeÕÍ#y$Ø[]²èYw+ë¾áááVÞûócÍwÏ¼$¯Ç©I#åF$lzüûGtsMî°Y>º	èÞ¯k¶Ïôc©äÁ¦SåÑ²páBy¬-åù¢7nñÝä»où¿ÛO+óOëF.ûuÄþËv­Ö'oð½ñ£átÿ¶ãÿNY)+jYå[²¶¯ìøîÅ¥Og¾¬4µIf ¤ºÕÈÚ_æÝ2vYíaØãñèÚG£Ã23ÜÔXjÒd¦ý¶)«Ì»­eÊ½Õ]|RjWae 9În½7X×°öPIöÎÏ°Æ¬ºU¡û¥÷-«½6È566Ö~ol/kBBãõT½A3ÎsUþpy<ØÁ1.×½oÓÞzÐe+ÿAéÜ+¤7úHp¼ï.ë÷ËYüùZASÂòÒå¬ÓÜCùÍ¾SFërÐ½÷ßÞ?(7::ZBú+ä×éR&,Y"Ï,ÛÜÜ¬¤éFl¾?Pÿ(óª!#`ùÛíc§»îW¯^°¬ºç@GÏº!ËY¹²âaï6?eQiÖ¹½iO¾õæoêz_Øg³7X'®.uT$+YyÙ¯³nÞ¼Yß¡a*+1¶G®îcYµ2`²y¬¡tÚª+kÐ ?®ëâ´q:î1¡2»äx¡uÊë¬ËªåÓ_7%e8R	qÌjÞÚa¶´tÑ9ÒÒw§ØG®nÙ²ÅüÕîCÃä¢üá:VÓCgµ=ºYsu§%ô²ÊcIò³wH$`öå»æ­´º£Bº¢¯ÁËE0[lYYY®Þayú1tRR<ÚÚÚÌ%ºÅ¦/mJÂïqßî~;Ù¡­å6Ùß·¬úçèc^#­C¹ÿZhÝ4´÷Õ²âáUKrìÎrDd«ët=Cwuºv*e¯.ee§#Ôà¯³ÊbÊ!yÕe¥¦sôH(k Ü¬S¶nÝ*ëz3Ó¬ôºzuÁU×¡qqqÚ3ÞrÐá½æ>fÅê(ë=ëPäp÷ë;me»­ûÌ+ô²º#²÷¸c  ì÷¥Üs½pÌj§÷-àOÂ³.m9W6î×Èu×±ÆF¯,½ÔÇ|ÕÇSÌ`]¬îÀÐ×2åÆÍ/µ_úÌ3ÏØím/³Ù¼óÍñ^VÇ¾®aÖYÆ¬PYõ½Ê¨¬ç¿cui)«î²Óõ»TÜÌüvÝÃfVyúª°Ùnæ8Ä~ÍØQÖÖ=ú2­¬õÐÐv;ÊªÇ¼lÛ¶Í~ô¾Ñe5KØÓeÕVõuVù¾ìqYu0go[²êî]¶ííífAÀ²³½ýÏnYu§§u÷µùÃe@ç[Ð¦Ù!ËJ÷ëê gm,óòüËØÈqî¸¾YÜ>VÙ~s22GMCÙ,ô8v¹e÷ÎÊJYñ`Ë*«/¹÷k5Ýo´_ªtzpËª§øq*+>]kèK×zC%w9ÜïíqUkv:nßñvàeµGðà½@ÇCkj'&:³£¬²áõzÍ!Á'ô×YÝèê¡mãK¥¬:ÉÆ|Õ]Ì°Æ¬æÛÊj¿F3eÈ¼è®L=vWOBd¿¯eÕct%ò0è¸t,8Ñq¿÷=·¹¦ûøà©¬¬Zh=,ßq6	ÊJY!Y5èÒ)BÖ:!«B='¾ÑÓý^ºG0éøÌ Ð±^¶Ç¬SRØcÄÐË::.ÈÐÄñ]¡Î>Õ½BØè;jxå*9Ê*QNN¡Ð±KðÞoØ°_î¥¬f½f!Yº%¿B×vÃeuÜI]ÓX·µµIäOÎÎÎëý¬æ===²ù¥Ö½Ò.«nÈuäö¥:ºW_÷¯^VèÎOû4­­­ë×¯×ïÊo7¨ÇÄÄ×wuXfîß«G¸Ïkö»OVeYõ=¥^ÑÕNû&sÛôerûß¤/¦¯²ã$ù§èÇ|<Õ³üíì(+eEä¼ÌÊfµÌªÇåºÏyñÞàYæÓ_=Î¬CÍQ¸²Óã%²R)¨¬5uí¯Û,«®ñõ´ïòK¥|::1/Ó2 Ôs$é9)¬[ånè^5½¹æîàéºOÆCr¯ôÍïîtìeH¤¯O4æåßmN]ëØß`s¼¡s¢ntN·ØÌöý©èÊM£e¶áôõNóò°ãJáÒ%¯dNî­û=*ö³ÏÄ)+²õi/ÙÚòJ°þ!AÎåiöÉ5åªÇ9¶ÀôØ]YyäÜÌu<sÍGØoò	åD§ ¬úÈîs÷ëêÛ½?VBe&²Vx°ûãeÌë Á?ÙÆìñ3oJqÓAd²;ï>¯9È&Ymoo?qâ#åÓ³áèáZú¢SÃLë+vî5à|8þ_82/*ëra«,Ç¹¤©î÷>?Çéä<ÑçMDÿ¢ ÜÊÄãñ¸Ïê¬»O³â]ø]Ùd¼ï-9&(+PV(+Ê(+Ê(+Ê(+Êex8O¨¨×_ÝsýúõÕãtêÔ©¦¦&¹æï½ä¦6lØ°eù0y£¿¿¿`¹ea¦åúòãæ)Ç#÷gppÐïÖ_Ê]@Y	577ÛõLÚemhhpddD'rssCü-·oß[ËËË³sµûvû:£££ccc:-rU«VM|ÓK¹ÿ2ýâ/NtprWPV`B¦³i)Ê#GäëÙ³gÝ?AYíÛAË:îîî^V½ÛÑÑÑS[ÖÐï*@Yü¾¾¾üü|hooôöö³N¦¬`YYÙD×lll1tzzú­[·>,áìéé	RÖ:fxWPV`Â¢tK¾<xðæÍ2qåÊGYGGGsÇ]Ç^¯Wâª/êä 1=vì=çôéÓÃÃÃ«uvv=Ò×ÔÔTùòeæç'dº¨¨(HYÃzñ5Ä»²ÁìÜ¹ÓjNNÌjö5fmmm-·ÅEË-ÓkÊDii©ùAé¨û:XÊÂÂB¿ß?ù1kèweîCF«:r½÷ã1AºuòµÇ:	ITlllë'''¯X±ÂlÄòîp~õÕWÑÑÑO=õtwòcÖpï*ÊÖßß/-©¬¬¯öÀt_g^j®dZÈ2ñöÛokÂ×|ægÜ?nï¡µÓ¸oß>Þµk×äÇ¬áÞUàîÝ»Ò¾¾¾;wîÈÄáÃ§¼¬ÃÃÃ?YÞw nY¥v2Ìõx<²0ù²uWPV pÉN>íÎÌÌÔ[[[ûGºººd¾êûþ-´Ü¸LgeeüiÝ[[['§[×¯_?ù²uWPVÀéÚµkçKZdDðúZluuuðÛ×Ã¾úê+3GB%Ñª¨¨dÊw³³³oÝºe·­¤¤Ä<ûFî*Êe²@Ye²@Yÿ8À#àÉ'üÃþ0½Ëú«_ýjåÊ½<~ñ_|[ù4+ë/ùË?ðxæg(+²(+ePVÊe²RVe¥¬PV(+ePVÊ ¬ÊJY²(+e`Zµ»»;77×ëõfff¶··SVe	jSSLÈ½LJJÒ	ú§¢¬Êæææ¬¬¬~ø!*5kÖð¿PÖÆÅÅI>;&ý.ÿú¯ÿÊ@YÃÓÒÒ²páB^gPÖ)ãõz)+²NJzzzww·Lttt¬^½²(ë¤;w.33SF«+W®¤¬ÊÊ"²@Y ¬@Y)+ÊJY²(+e²RVe¥¬ÊJY ¬PVÊ ¬Êe¥¬ÊJY²@Y ¬@Y)+ÊJY²(+e²RVe¥¬ÊJY ¬PVÊ ¬ð`¿öÚk±±±YYY|ò	e¥¬È½üòËyyy.é'O¦¦¦?~²RV@$Î9æ÷ûÍÏ?ÿÜçóQVÊÄ~XQQá)e½zõ*e¥¬°:t¨¬¬Ì3<<xãÆÊJYa»téR|||WW³ÿþ¼¼¼GÿSVÀ#ªººZâZQQQUUõòË/§¥¥In)+eDNÆ¬o½õVYYÙ»ï¾Û××7-î3e²RVe¥¬ÊJY ¬PVÊ ¬Êe¥¬ÊJY²@Y)+²RVe¥¬PV(+ePVÊe²RVe¥¬ÊJY ¬PVÊ ¬Êe¥¬ÊJY²@Y)+²RVe¥¬PV(+ePVÊe²RVe¥¬ÊúhèèèÈÎÎöz½YYYçÎ£¬Ê:)ééézÿ;¶lÙ2Ê ¬S&&&F;êÏÿ@YÃÐÙÙ¹uëVXãòÄOPVeÃ­[·nß¾ÍÞ`e¬ë×¯oÙ²eppcu²Z[[W¯^=44Ä»nu¤¤¤DY(+²r¦e¥¬PV(+ePVÊe²RVe¥¬ÊJY ¬@Y)+²RV(+²(+e²@Y)+²RVe¥¬PVfjYëëë£¢¢FGG)+²NAYSRR,Yð[ccc@YÃ³k×®;wYjjjd,[QQAY5ÏÜÜgPÖ)+ëÊ+)+²NAYïÜ¹5®¡¡Á±7øÀ2¿··²(kGFFÜó¤¬@Y'E	ÖwãSVe½¿ÂqÀo¾ù¦Uåk^^¯³(kHîÞ½ß#(+²N)kYYePÖÈ]»v-==ýÄ»ví²?²(ë¤ÊPU²ÚÖÖÆûYÏºà/WÖÎÎÎµãÌ±ÁJ§ëêêdð*Ó@YCÝ÷ëø@IéÁ³(ëLY²ðhullLjºûvwYO²(kãT×#t²(ë¤ÊÊÞ`e¥¬PV(+ePÖÈËÊ'(kx~®ÇçääãÍtMMz½@Y§àÃÏÙ ¬|ÖWÖ¨¨¨µk×RVe²²îÝ»×sçÎÊ ¬!imm]?N>vìõ©§2^]]-säWRVeéÀ`û3ÏëêêÙ ¬Ý¬$wöìÙ¤¬Ê:©WXN777ËtOOePÖHö	KGôìK&®^¯÷òåË@YCróæÍúúúÎÎNsêÔ)	êÅezpp°¼¼FGGË5)+²344§g7ìêê|^»vM:zôèQûj£££Y5TRÓÖÖÖiªßï!¬LHP%½2]YYIY5N>­eÕT%®:_ÊzñâEØ¹s'ePÖûÓQe¨jUUWWë±Á2ÿ½÷Þ£¬ÊÞ>a×ÍÆq6léÞu ¬|ÖVÖ/¶´´Ã(+²NÍÙeÂT²(k$''5moo×/_Öc)+²MÏáxkÍx+eÌü²êéø·oß0·EEE@YCuûöm=ÓúqE?jhh8|øðÊ+õÅ×sçÎÙNYuÂ£zõcÏõbnn®~«±±Q.ê$|>4²(kØ¡ýõ×uº®®N.Þºu½ÁÊþþ~ûÍ62ÁLÊùuíÚµöÅÊ ¬8ö¬¤ôÊ+vY)+²íÄÒQ=©··W.VWW?ÐÓ0QVÀÌ,ëµk×Ý(·uëVÎn ¬¡¡¡Ê ¬÷FFFdÀºyóæo¡½½=##ÃÜ²fuYõÖ/Ú3ïÜ¹ú-è	ýõâ?üë2oÞ¼5kÖð¿ÌÀ²¶¶¶S644HãããeÂ>Ó¡ýs¡¿ÇL×º<÷Üsùùùü/3°¬£££fº©©IÏ»$ÎI½ÁYZVÇÙùõ³äjjj&yjCÊíeÝ¾»ä°¬¬¬¡¡!:::&&¦aL¯^½²(k/¦¦3ÌÊÊ¢¬ÊÆ©¥£íííÃîînwY§üÂ0cËÚÚÚªCR ¬&éûï¿õÕWä«L³@0ëÊ:44400`KYLÆñãÇ½^ïOúÓ¼¼¼ÄÄÄyóæ>ÅY7fµ¯¦¬)))Ë-ë'3óóó)+à®^½êñx^zé%3gÍ5óçÏ÷ûý,ÌÒ²êÉ]»vM¦>ulllïÞ½2³²²²î>³çHS¶bVÕ&eMOO×iýt9Ê ¸òòòÅ;fÆÇÇüñÇ,PÖÿÿÚjqq1" t26;w®ÔÒ7ß|3gÎce(+0#¥¥¥%$$|ùå2-_eÀúôÓO³X0KË*Ôsñüùó2§µµ²3u|¹iÓ¦þçÞ¿ÿÞ¬ßïÿùÏ.ãÔyóæÉWæð%ÌÒ²^¿~]:ÚÙÙi6Ü·¡¬ÀtñÂ/Hö~ö³ÉÒëõ&''_½zujÅ7ß|CS1KË:22RQQ_\¬'8<vì~øyJJù98KYiM©öñºÒÔÄÄDd@Y§¦¬cccÎúúz3T¯7o¶Ç¯/¾ø"efÌÌÌM6Ùs¤²2reÉ²NÙÞ`§éS§Nwµ¤¬ÀÌüÎ;ï8fÊÓ%Êú ÊºdÉ¡¡!¸÷®ùÑGQV`fX¾|¹cß¯vÁ,PÖ©,kzzzqq±Vå«Î[s>ø²3Ã3gæÎ»iÓ&=ÂHÝ^¯÷ÕW_eÉ²>¨1«F´¯¯O¦;::tæÞ½)+0c466ÆÇÇK_=ãÊËËY& ¬¼¬bttÔÌ<pàef«W¯öôô°WVûûöí£¬ÊÉë¬MMMî·åpl0²Fø~ÖÃë®àäääÚÚÚíÛ·;ÞCYuBRP	gOO^¼sçýÝ£GÚû)+²òY7PÖ©=gÜÜ¹sÿçþÇà5**j÷îÝö.â)§Í_ª¬Ô¨?÷Ê+¯ð0<À²êéßï=3çâÅÏ¦e]¸p¡ü!=ö£qåaxeÕ7ÞÈÇãY½zµ¾gåÊ[¶lÑ·â´··OÓ²êÕ#VÃ>aÀ*«¾ñ¦®®N»~ýzww§ïyåÎK3öYIxÄùýþCUTT´¶¶²@iSÖúúzóªªî)qjñìÓôÏ1kJJcV<ú®^½³bÅë[o½åóùä+eëôÏµ¬²uì¸ÎÜÃ,«îû¾jJe¨p<jd»¶°°pxxX/^ºtIâzòäIð¨U2ÓÛÛ+_kkkõ¢Çãijj2ÃV¨©©ù7oÞ¦ÇëAL6¬xôIG¿ûî;YwìØÁé²^¿~=55Õ~%UÃÓÚÚ*_õð¥+V´´´ÈÅË/Oë3E¤§§?öØco¼ñ L-2VuèÐ¡mÛ¶±dGÌê8F)HYûûû9ðÐ¬ZµJRjÏÉËËsÌðHµ³³³··wçÎ111o¾ù¦ÌINNÞµkW\ÜîÝ»§õÞ``:úúë¯ããã%¥2ríëë+..^¾|¹ßïgÉÓ©¬òµ««K¾>1+ðwæÌ©ill¬Ïç+))¹zõ*Ë~e½rå|mhh ¬Ê:©²fddøý~²&$$TVVÔÔÔËÌ»wïRVeï&¶ßÀ:22bÞCY5GqßOÏgPÖ°Ý¹sGRºyóf½xûöm¹xôèQýePÖæ;uêÏçÛºuë©qÕ7ÊDmm­LËo¥¬@¸ü~CCÃ»ï¾ûé§rp/0Æ¬cccvh¥£G¹÷ QVÌiiiyyy¯½öÚºuëdËõÛo¿e±³¢¬CCC+ÇÉuïÞ½RÖ=i°X÷ìÙCYpISËËËÍÅªªªÔÔÔ7n°d_V=ØµTPPàøîðð0eÂ200 uûwÅgÎaá3¼¬mmmÆOcÁ«ýù¬uuuú8Ý×_-uÌçÔ'|ÂÂfxYÍhU¬¹¹¹÷<ÊïÆñññf_ÓÒÒ¤¸,`Õèïï²vttPV`JlÛ¶M­.ÓøÑLEEEëÖ­c±³¥¬úîÕææfóçöLõõõ@EEÅ¢ERSSËÊÊø°`¶U³ZSS36Næ<O;§¬µdÀê8	ÀL.«lDKD·è(ëèè(ePÖû»÷®tddDnqíÚµæá=öÈüÒÒRsÀ° ¬ÊÆ9í31q`466dff¾ðÂçÏgõQDY1]¼úê«sæÌyúé§KJJ|òI¯×ûå_²XÊJYHHDçÎûñÇ9kÖ¬IHH`É²(--MKK³çzU°|j@Y)+^xáé§vÌ7o¯¶²xçw,X`Þ¡¶¶Öãñ°dÊJYÅÇÇ/^¼¸§§çOã	Kh7mÚÄb(+e"ôý÷ßKYçÌ3oÞ<ùºfÍ	@Y)+0Î?ÏYÊJY ¬PVÊ ¬Êe¥¬ÊJY²FäW^ñx<sæÌ¯ÿýßÿÍ#(+eÜc=õç$´<È²RÖH¬Y³FRêõzÍ+2 ¬5sçÎutô7¿ùÌa0PVÊ9sæ¸G¨ìÊJY#´`ÁéhWWÌÞ` ¬5rúÂêo~óNOOiÈò N<ÙÐÐÐ××ÇÒ@Yÿb>üðCûÀ`²:Míß¿?11qÝºuEEEñññre²þ½òÊ+önaL#UUU>ïÂzñÒ¥Kiii²ÍÄ@YH<ÿüó~ú©=çøñã«V­bÉ ¬@$²²²djÏK.eÉ!e=wîÏçóz½gÏ¥¬xÐ«ªªì9rQ²,3¤¬EEEµµµ2QYY¹aÃøÌ¥  à¿øÅçÀTøÿø¿ú«¿zÿý÷íû÷ïgÉQvvö#]Ö¤¤¤±±1IIIùá¢xÅÇÇ?Òeõz½é­[·²7SkxxøÛq~¿¥`Fíöx<f:::×YuR.£RªÊ4ePÖIÙ¸qcMMLÈ×¢¢"Ê ¬ÒÞÞìñx|>_GGePVÎ ¬õÁxì±ÇÌaÙ,àñ²FN?þüùÏ?ÿ¼×ëé¹sçò| Y(ë,ëK/½$)öÙgÍÅËÂ'|âÏÇ¬ ¬3­¬?þ¸tÔ1Sæ¤§§ó(rÇ ~öÙg2í÷û+**RSSeüÊ@YgNYããã5++GáËÉÉq|á»ï¾Ë@YgNYkjj¤£>ÏÑÚ?þñ<ÄvãåÕªªªÒÒRÊ:£`?¾¤tÎ9ý×­G3ÉÁ!##ãÂö7Þx%²Î´wÝ¤§§wÝØãWDàÒ¥K;vìxþùçKJJN<iëµ×^ËËË3'Óÿúë¯å+eåL¬««KbYVVöÙg:tH6SìQ¥¸iii2T---¯®®f¡ ¬!©Òï¾ûNò)¹µ¯#Y¹Î~èØ3²âÏøýþE9QÚ¶mÛûï¿ÏÂ@Y)+ÂvãÆÏç(kEEÅþýûY8(+eE$ÒÒÒìw¬JkSSSõÔ@Y)+;tèPNNNlllFFã<ÑÄÄDyáÂ^¾|yqq1§@Y)+&$Õôù|þùÆß6#í,))±¯ÐÚÚºnÝº¥K®Zµêý÷ß'«(+euJJJ2ïvõx<³ùqyãÆªÚÇú¸þÊJY'd>Wî¹çÑ³5ÍÚÇ¥RW¬XáùòË/;Î²ö¿ÿû¿Òüä'fÎºuëd|Ëï¾ûnéÒ¥¼ùùù£²RÖüýßÿÀOÂµÍ¬¬,ûý©'Oh²RVÊ!=aqqqUUÕ¶mÛ$«XPVÊ*Ýkæ<÷Üs2G¾ÎæGg__[KKKßzë­K.ñt@Y)k$«zTp\~ÆÜl>	(+e:N5åÑ	²(+e²RVe¥¬ÊJY ¬PVÊû·ûãÿÈ(+e,=±oß>¾@Y)ë¤²ºxñb¦j¼e¥¬2Yµç,X°G0PVÊ6¤º[Ë²RÖÈÇ¬VeÎ¼yóxe¥¬Õ~a5>>^.¾ôÒK<²RÖHÔÔÔDý¹Ç/PVÊ9°.X°`Î9óæÍtF«ßÿýÏþs¯×+wLbÿÎ;ïð¤@Y)+"ä÷ûãââ|>ß_~)å1wîÜC±dPVÊHJY¥¯fNII	Ù²RVDhùòå6mrby/ÊJYqþyEEÅ;ªªªÍü§~º  À¾æùóç)+ÊJYgµk×Ã=Ïwß7U·ºÿþCåååÉ8u``@¿%3çÍ'5µ[ûäOò¼@Y)ëôöÿðzJ¦¢¢¢¿û»¿Ó¾¶··OþeºtéRRQ\,¿ÅÙÏ~6þüÒÒR©lff¦öo¾áy²RÖéM::wî*s¢££'Ë/¿üruuµ=§¯¯/11Ñóê«¯¦¥¥ýô§?áì3gxR ¬u&õäÉöÉê¼Þ)ÃÓÏ>ûÌ3<<Ï3(ë/«cd9oÞ¼))ëo¼!ÃVÎ¡CV¬XÁ3(ë/«ýa8RY¹¸hÑ¢ÐoAF¢]]]ß~û­è¯¸qãÆÒ¥KËÊÊ®^½*ßªªªkkk+Ï ¬3Ùþýû5®O<ñÄã?îí?~µ|ùòÄÄÄO?ýÔþîÀÀ@qq±Ïç¦Êh¬e-q3g6566Öñ®£GFGGëñÃÞÎÎNó-)¥ÔÔy¤?ÿüs@YØ| S^^þë_ÿÚãñÈE×ÂÂÂªª*ûúrñùçg¹eE`Òüä'ö¹sçwÎdee]ºtÉþ®\ºt)Ë(+³G¨ê¹çÜê´Oìï<yrÕªU,7 ¬°¬¿ÿýïÿuSÖªªª´´4óº¬LÈõÃ?d¹eE`Á÷ÿiüè§øøøÂÂÂ¢¢"x÷ÝwYh@Y)ëÌL¿ýíoÝG0©qSx* ¬uÆ:zôèüùóõ=9wÝ(+ePVÊ ¬Êe¥¬ÊJY ¬PVÊ ¬@Y)+²(+ePVÊe²RVe¥¬PV(+ePÖIJII¡¬Ê:ÚÛÛ322¢¢¢(+²NË/ÛeÝàÏÿ@YCEYõA½ÁÊvGePVÆ¬ÊJYUeåLÊJY²@Y)+²RVe¥¬PV(+ePVÊe²RVe¥¬ÊJYeÝ¹sçã?>¥ÒÒÒ,Y×âÅY!ú¿ùÔÔTCþöoÿÅYÐX¡/.yJNímÆÆÆNû²ö÷÷ÿçTûÇüG)ë"4ÿò/ÿÀrÑ¯~õ«¨¨(CvïÞ-ëw¿û"ÅõÛßþE?þX×¯ýë©½Ùÿú¯ÿÞeþýßÿý©§ºÐ|ôÑGO<ñË!DÁ?r¶ÎÎNY¯_gQâêÕ«²¸þð?°(Bñÿ÷²¸Î¯£¬²RVÊJY)+e¥¬²RVÊJY)+e¥¬²RVÊÊJY)+e¥¬5¿ûÝï^zé%v!ª««öÙgY!úâ/.]ÊrÑdqùý~E(equww³(B166&ë÷¿ÿ=e`ú¡¬PV(ë£æÜ¹s>Ïëõfff=ÂDRRRXn¡èèèÈÎÎ%Å®.öövWZZZÌq,± £,sqÍÞ²ÕÖÖÊDeeåx$ë»ûW[ééézÑcÇ-[¶Å¬ÝôXMYhIII,®PÊÖyJ²ÄhhhØ²eË_dµ?Ë*Ïä±±1±Çd°|Ù.+Ë-D111,®577Ë(Å½÷êy>ÞdÕñ6¶¸foY½^oÀixXee¹¢³³sëÖ­,®PF`qqqòQ>ë¾®#m0OIXééé«W¯Å"­··÷a.®Ù[VÇc¦£££yXVÛÝºu«¨¨èöíÛ,®µ´´,Åu_mmmöS%ë×¯ëN¶¸foYå<22¢»ôYPÊÊr»ïsxË-,®°èÅußg¢ãX´£mqÍÞ²nÜ¸±¦¦F&ä«0xäXV[­­­«W¯bq"==]O!ÔÑÑ!ËÅÁS%üÖßß¯Û»ù6ËÚÞÞìñx|><±yXV[)))!+sçÎeffÊhuåÊ:ÊgqûdÑÙÙ!°¼¼<=õC[)Êe²Êe²Êe²@Ye²º§qTTnnnÑ¸ºº:óÕW_¹þ+ß¦~¼ö®]»ôâíÛ·åbuu5K ¬ÀLÓÒÒR8N"*µ;þ¼|ÕÏÆ²+xúôi3'##CÒ[<îÔ©SMMMrµk×9ûöí97oÞ4?ÒÞÞ.sÌçèm666²üÊÌ4wïÞµ]]]òµ³³S'FGGµ`½Ú+Wäâµk×äëÊ+u¦ý!²:-//wÌkÅÍ8¸  @¦9"Óü/ÊÌúQ¦¢§§GwýúuùÚÝÝÝÜÜ,~¿ßQVÏ'Sík\PeB¤:`­­­9/^tzå6MÅoÝºeß&ÊÌ:T¾Ö××GGGëøR*¨ûxoÞ¼é(«êÇeëÞcà^¾|ùÞ/ÓÚW>xð ÌèÊ´Æø£>9Zb8qâÿ²3ÇÐÐÐöíÛ¥pÇÓ4ÊSj'2ÊtU.¶µµ¥¤¤èNÝ·ß~[fööö&''oØ°A&ìY¿~½î@Ö-,,tDZnÿ@YåôéÓæ"µ²²R&¶nÝªemnnÖkJ>å»ºßX_"5/²ê~àþþ~G8e,_¿øâ3Ø¡ª9ÜI¼üÊÌ(ÚÈ¢¢"___&Pb)ÃV¸råêuôuÙ´´´Sãô%HJJXÉOÉ´^ßÝcVýÊÌúRkGG|=|ø°	ÞÞ½,Yb.úý~ó#Õ÷MêaÃ£££î|2f(+0·QQ;vìÐììl¨¯¯p¦¦¦ëPå»rqãÆ&v&ÓÓÓþÆ¬efÝñ;<<,ÓgÏ]¶lGe¾U'£OóR«Í¤ãÑóçÏËW¹îòkx3f(+0óéÑIö1GG9÷~|GM^^æÐÞ»óæM)55eâÊÉs¸cÖîîî U(+iÃðîÝ»RÄÌÌÌÖÖÖûþTcc£s¥rý%Käççë·ü~¿|+!!Á]Öºº:=¢1fÝ±cnÿ@YYgÙ²eµµµæ¢ªiÏ=fÎ­[·ÃVÝÃÀ ¬PV(+ ¬PV(+PV(+PV(+PV(+Ê"òÿkö9×zÅ´IEND®B`


ICU时间d


正态 Q-Q 图


ä«~ùË_ªXUë]½g$==Ò× bä«'O´Ë¶lÙ¢=IqqñôÉÚØØ¨| X±bLlkkÓ?dÉ%«¿çîäc²ªÁª~IëÁ¡C|OVµS®zïdDQÛ,'lÜ¸Ñ÷d]¸p¡Óéò§õÑiuÝºuj|øðaòª$Ød<i½û¶z*í«¼×ësçÎ©zÔä%ÉÄ·ß~[­M-++[²dÉÎ;å®<Ç÷Êg Ë¥¾½ÉW%¼åÑVÈ«­¿jñJ«f)Ün·þ?úè#÷"77WËëôNV5æªm³÷xÉ$k Um;Tk%u¬/_ö¥³Íf¹«í:888².^¼ØcªzB­M>7Y»6XbI^ª¼0j+¦Ú_I*»Zj6µµ¯òJ´u§ê`$õÕ~U®¿6±SnLL|,P?B~jð2HKKËÊÊÒ×Ü/fgg«´[µ|°ÐfPÍToo¯ÇO,ó®åý3¯ê«W¯4Y¥¶ªÙö¬>aäåå©Ì&YA²ÂXÉ*õHÛ¢6©ÒW&ª~S%«J>AIÖIûU;DÆRv³ÒûPKUBNâÄjµê×ÄJ Iõ÷÷kSä«sÚ]É?µm[Rí,we mXµX,MW¾$Sµ(QzõêUU(Ml|ÕÖêKãqí¤Ó<ûf¶JîÚÚZUÍÚ sªú®¼~Ðª|«¬0D²ªíméróæÍU«Vi0Éÿtu´¥þ1*5%x´Ç¤¦¦z$«ÊïóNVïµÁêHd»CCCZó=Y¥By<@íwóé§ÒYµæ§^§Ê0m_gP%^õ`ÉKu©Üªãg<¶|ßºuKeµESmË'×ÖåNº©Uû¡¯¿þºþî5k<£GR5,ºéUíµ¤MZ0«ïtV¬0V²ªõÒJ%%i¤ÆÉ?wµ[ö¿X­½.¥*åRRP+BíYSZZªßJ:UÎM¬ÚfEµÃw²ª«j;«|Imvõ;YUeÔ¶û¬ªá©½~Ôeí%É'5]5;êçjÇ³Êõºj!tvvªµý¿daNzb#³jLµ($z=öUÖ£¼ÚIÓÔµÁBU%Ïì±dÉc%«Øºu«÷Þ¶(jûè³£;ÔJcï³Ih+-å®þ¨"¨Ö»NÚUMuþ dê¦VM+&É÷í¬çjxöÍ©þô±ú¬úãaÔ0ä3>äje¬Zå«öÝU'!ÒVÒjÉªÖxQâ3==]>:¨&­mU]pªý~'Ý³wÒGªcy:ÿU%´:3ÇÙ$HV¬0V²>ØJºråÊJV%''xO)Dú¨tY	6y:¢¶YÔã¿§VMwBRYý·4Ã¦:SÔ8yUÆjË6]*µD¼ÜÜÜÏªãP>È<ªÓ	ik³M¬ÆÜ°aa®OJ¶W­ªeÚw©ýÕ)|½Wªkë´½O#¥ï¬Ï¾9¥:½¢¬ÍÍÍÓ¼-ÒÚ­_jcêô©ì±èäÅ¥ÖR¨=U²jKX¦×××¬ YY¡V~æççÏ´xIcjGY?@h'ÈõØ£GuhÃF=N¨4Sê°E*¯|Rñ>FEÿÚ´]ÔÇ³Ù¬mò9`ªÓ>ûf±þ&%MäcvWµ|oÔö5&½SVòRÛÜëñ)Íãüêìú||?$@²ð3õµJ¶sçÎ»Ú|;Í·&Éû|Ëj×bX©~uóæÍÏiÎO	¬¬¬ddd$+$+$+$+ Y Y YÉÉÉHVÀï??üP?epppõÂo?þÜ¹sòÈûöMóTn·ñâÅ7nÜ2áÒ¥Kò-åååúÇô÷÷¯ =³ÐÆòxùví)&iáÂòzôÓ=ø8¿¾¿$+0¥/êÃCbR¬Ããñ£££j¸lÙ2Ê'OäÙòóóõÉºmÛ6ýcÆÆÆÆÇÇÕXòU«VMùZ^ÊëñÛo¿=Õ|çû@²SÒReIJJÄ~Ûææfïoô#YõÏ#wm6Û4uYõàÛ·oO¬êeÇÄÄ¬¾¿$+0¥¾¾¾´¶¶JôööNßYIVÎZRR2Õ#¤Cgdd:tH³§§gd]¸pa°ÕdÓÏ$6äöàÁ?Á½÷<ulllÙmÕqtt´«Ú©Ö$Ofò´'OÔOillt»Ýkoo×ÖHK¶¥¦¦ªéwïÞééééÞÁyæÌÛíöiÕ÷¯>¾$+ð;vìÐªÕjõî¬ÚzãuV§ÓY4aÕ²²Ô#eP\¬£ÄþùU4)m6Ëå¼³úþ¬ÀsH[UÍõÙ7û©µ¬¯Vy¬º $MÍfó4_ºti^^¶S®Vå½óÚµk111+V¬ØJgõñ Yéô÷÷KrÔÖÖÊ­¾q;«<ÄJVKEÁîÝ»U<òõ×_÷þvýÊd4~òÉ'2Þ¹sgàÕ÷d¦422"±Ñ××÷ôéS:t(èÉêv»UøI³|ngi²J0KË4LòùdHV`üUDD466zsrrT¹¼zõª÷·tvvÊtßÏ %´<¹-GOõ®Þ+cNçTÁ©jë¬ÏHV`r<túèèhyy¹Ä4ÚI ¶ÅÖ××Oÿüj¯k×®iS$S%_+**$±l6|577wxxXlEEEÞi§Ùþ¨1·/ Y YÉÉÉHVHÖéìß¿éÒ¥)Ì¯¼òÊ¿üË¿v²þâ¿X¹re/óÀøÃIê±dýñüÌ¯¿þ:ÉÉJ²HV@²¬¬¬$+d%Y Y YIVÉJ²HVd¬$+d%YédÙ²eKLLLZZÓé$Y$k@>úè£ûöK¬¦§§Ëk^Þzë-@²ú$''§§§G»ûõ×_GLæÍ7ßä½¬Ïýé§.PëÍ7eËËÿñÓY$«OL&S½z­V+ÛY$k@,Y¢ï¯$+dHiié©S§dpûöí¼¼<ð]]$ë?~l³Ù¤­Z­Ö»wï¬i¸Ýî]»vÅÆÆ^¸pdåLtttX,¨¨¨½÷JÄ¬$+ÀO.«¼¼uùòåsûbHV@hs:ÙÙÙ±±±s[UIV@øTÕüüü¹ÝkdOU­©©UdPUIV¨ªóµª¬Ð«ªÅÅÅó¶ª¬PÒÔÔ?o«*É±ªZPPÐ××7ÿ_0ÉªZWW*¯dPUIV¨ª$+ÉãªJ²¨ª$+ÉTUd¡ª¬¹wÿþý°©ª$+`=6)))lª*ÉËªZXXa·ÛÃ¦ª¬¹¬ªâèÑ£a9$+ªJ²¬@U%YIV0¦ÒÒÒ¨¨¨ð®ª$+àE¸páBrrrøíL²^4ËURR»víÚîînãÌ8Éªj±XTUu»ÝwL>T§Uªj­ª$+`555eggÍfVUäªZTT!UõÎ;F^$+ PW®ªöÇª¬Y¯ªjàM6¼ª¬@9Îôôt©ª'N`i¬ÿ¹ÊÊJµ0UdäÂ©©©			¥A²ªªêÀ6ªJ²ÒÒÒÄVUJUÝ´iÓÀÀdøOílëª¬$+ÌzU-//ªZ\,cÉðß+W233N>ÍÒ YþÓv.,,4æÅjHV¦©©ImU=ö,KdTUËÊÊØd%Y 8U5;;;55ªJ²¬u±v&YIV§Ó©N«$ÉJ²ÿT¶ª¬$+Úª.ÉJ²ÿîß¿¯¶ªÚív³@HVüçt:Ùd%Y P>,..ÂÊÀ$+É*U5))éÊ+,dÿi[UÙd%Y UUzjjj*Ud%Y  j«*«!YIVNUU;_¸p¥A²¬à?m«ªTUv&YIVÃáH ÉJ²ÿ¤Úíö²²2N«D²¬íXUÎL²¬©ªQQQEEEìL²¬µUUÚ*g&YIVÈÃUUe`d@«ªôÔºº:ÉJ²ÿúúúÔÀrËVUd=zT«ÊVUdàTÕÂÂBU%YIV ª¬$+PUA²¬¨ª YIV¤ªPUIV ®®.>>>55ªJ²¬ªZ\ÌiHVSU/ÀÒ YIV ª"4õÒ¥KòËG² ªd±±±ÜÜU$kpìÙ³gÿþýZ²þÂKvv¶üò^ ªd¾÷îY­Öññq-Yßôò;¿ó;$+ª*HVØl¶«W¯þïbm0ª*HÖÀEü6@héîî¦ª¬óô¨:+SSSc6©ª$+ÉA¨ª«V­ååTU3E@ªjffæ+WX$+ÉTU¬$+ª*HV@ØP;SUA²@ 8$UÕét²4@²ÿº»»óòò¢¢¢ÊÊÊ$+ø¯¾¾>!!AªjGGK$+ø¯¯¯oíÚµ%%%÷ïßgdÿíÝ»W¸©©¥ü§UU[UÙ$+¤¦¦Fªjffæõë×Y YÀk×®ªZUUEUÉQ[U9V$+ª³³3??_ªê®]»Ün7$+øOØb±°U$+¤»»íÚµ±±±V	$+øÏívþùçñññRU9­HV@@:;;%P¥ªr¬*HV@ 8 *É*ùÊÒÉJ²ðv±¶ªd%YdïÞ½f³Ùjµ²0HV@@:;;/_ÎA²¬åv»+++¥ªæåå±U$+É  RUcccÙªdªjµZ»ººX  YIVþ(ª*±ÊA²¬SU-Kww7¡¬¿úÕ¯Ô`Ù²e2hmm_ë¢¢¢óçÏ§¤¤äææ¬f[KKKvv¶ªªlUEÈ'ëþýûÕÀjµÊàÜ¹s2¾wïü¢¬fºXMff&;#´µ½½]SªØív¹=vìÜ>xð@%kbb"É`tuu©Ó*UUUq¬*B>YÇÇÇUU]¼x±JÖ÷ÞO¦?^ßYIV³Áív8p@ªj~~>«A$«¶6øàÁ2ßï+VhÉ*ýUÑÑÑ6l YvZ¥ÊÊJvF&ë¤µÁf©ªJJ¦RUa ÎªRvhhd¯_×.VCUõøñã2±¸¸µÁ¨ªª*66vùòåTUm²^½zU­ø@AIIããã·mÛvòäIõ:+Àµ´´¨3þùçTUs²ª|mllÓWòõí·ß&YÎårK¦æååq]U²¡¡!ÉQ)©2v:2>uêÜvvv¬ÔÝÝ­Î|àÀª*¬¢Ú7nÈóçÏËx``@­+Öú+ÉÀw»víR×U½sçKHÖÑÑÑöövïþ*ªMéïïW-d0£ª/±ºwï^ª*ÕY¹Öª*@²§®_¿n±X$V«««¹X¬ÍÍÍ$+@8p 66¶  ëªdý¿Ãl>ùäm¼zõêÂ	j¦/¬¦ÒÙÙ/±ÊÀ Yÿ÷:¬v»ÝáphçnllÛÉT-YoÜ¸A²¶UcUA²þßUää¹$;zzôýuÇú»ÚùHVúªªÎÉúÿM¬YYYúÎª5d ¯ªlUÉ:e²Þºu+;;Ûf³=xð@î9sÎ`ª_WWÇÒÉ:	µaU<~üXåè5kd022"õ½÷ÞÓ.'G²Ðªj__K$ëzåvlllñâÅ;wîôøÒ£G8êU$«O>7¡p2ÒY+**Ôºb0,·ÛMUÉ:O<ñ8°U3EP:::²³³©ª YgvàMÎu`«$kLLcÓÒÒHVÀ°Uµ²²2**ªÕÏëOÕY=J-ÉPUuJª­ªCWµ£WåoÉc Yª*@²>ßÈÈ×º@UÉü3òK1Õ¹yòäÜ­¯¯'Y£UÕüü|ª*HÖ $kkk«Di[[>YgïÒr$+0«jll,gÉÌ«È;wîÒ¥K6Íáp?~IÆqmm-ÉwUíêêbdN²ªzûömm³ëðð°L ¥³TUd±J?^Æqqq2øì³Ïd¶o°þý$+@UHÖçÌºaÃÏ¨ÕÂ6ÍcEññãÇIV l8Nª*0ÉªÖËíåËÕ]Õ_õÇ³rV <¸òòrª*0ÉzïÞ½ÄÄDýEX'í¬ ª$ë«¬tVª¬ÁLV:+@UHV:+ª*:õöíÛããã$+BZZZ¤ªÍfª*0gÉzüøqí¬µ¬¬Ln[[[IV ´ªjww7dà<räHp)É¼x×¯_W[U÷îÝKUæ²³r9 Ô=|ø°¬¬Ìl6KUíèè`s¬íííë&Ø'8¾¡ÆêÔü2&Yù¬©©)==]]WªÌq²y¬(=xð ªZRR¢¶ªÞ¹sÌ²Nº7ÉVU=pàU YIVÀ£ªª®]»¶¯¯Ì¯d4Ý¶mw²¬À<¬ªf³¹¾¾ªÌÓÎªOSÇÇÇ«=Ôdæ	µ°TÕ¶ª¡¬¬nkkËÍÍ¶X,7nÜ Y`¹råTÕª*æ3ËUYYgµZCât%ó=Y322Ôë;yòdVVÉå_Uii)U!aýúõ«òA°«««¢¢B>vvv¬AÛiáÂ_ýµÙüxóÍ7ùý|¬ª©©©ñññRUYçN>-µ)÷îO$kpµ½½ëÖ­28æåþàæÿæCU-//]»v-U!A~c8àñk,µdý­du»Ý~<áðð°Ýnòä	kÿ8©ªò/éèÑ£,P²VWWë§<|øÐXÉzéÒ%Í¦ö¶Z­ÚþÀÚøÈ#r7::zFO;88¸eË¡¡!öüpÿþýââbu¬*U¡åôéÓééé¦ÚÊÊJc­	ú5N§sõêÕ=â¨ÀgÏLf³¹®®¥PTTT]__áÂÂÂBùîêê2P²Îð|Ìª*5ë×¯§ª"¤ÉçÂM6ÉorEEÅÀÀÀüÁCòé^íÌVU ÜU>/¯[·d^dU-,,²Ùl!qL=@²Î8Y÷ìÙ£òôéSíªZSSÃipHV§Ó¹aÚøäÉ¬+V¬Ð®^__/SäG¬@pÝ¿ªa²è/~üøñÄÄDù;gm00«´ëªrZ% ÜÕûZrÍÍÍ2æhTð¶UucU[ZZªªªJKKkjjôÇ±dÕ$sñâE÷ôô¬@©cUÇT©®®NJJd­««³Ùl^²H&&&ª0544há÷î]JUUÇªNsÔ¼´UUýf×U«V±IÖÇ:ujñâÅíííjÊùóçåÿÖ­[2*//»111òHð|fª|âÄéY5A?EÍåó7Y=z§NpØÙÙ)ñùàÁÉÑ/¾øBÿ°±±1:+àwU-**R×UíëëîãKKK?ÿüs©©©!qdý?¦N§óÒ¥K©ò¹X*¬äÏXU¢WÆµµµ$+àÓ§OK(&%%ÉÀÇcU%V=Î]~ýúu)»,L U²³±±Q%«Ú¤*áª¦K²ÞºuK;vì YUUu±ÍæKUÕÈ§ÛÌÌÌ²²2µK°Äªd³w0UmF?f²*õõõjß`¾oß>ðÑÙ³g'øw`Iâõë×'$$H¦ÊÔÔÔ°Hë¬Ú:auÔÍæ	7nñâÅ9êðÝÀÀÝnWÇªÞ¿?§r»Ýl[B;Y¹ÖæÄ'¬V«ÙlNOO¯ªªéç:¤¤$iÓ«ÀpÉzëÖ­K.iÛ_Ï;G²böÔÔÔ¤¦¦655É¸³³3??¿´´4gDê©Íf?M6qÊ$dò4L2ÐRdEÐ¹©ª¨ú|æ,ó¶vKOÏ=VáÕétJ¶¶¶ª»wïÞU;7¬©Ë/÷(ïôéÓ!TUív»üRUu³Fxï<ÇÛ¬P¤Z,ë×¯GªÄVUdò¼ÁÛ¶m4nåS9ÉÙà±UZl||üþRO¥[ËFqqq;Ïdòä:Yv	tí²çZ¹r¥ÚøzãÆý<Ûíîèè¼jß+W®$$$TVV655ÕÔÔøè®ªÒSå3A­µð¢UR³··W]UÝ]¶lúRCCÜUºÊ=É`>ª¯¯jµZ-üò¨½uwwMõyBÊ´Úª*/­ªÉ:³ ýðÃÕøøñãrwxxµÁÃ!eT*©vWRÖétî©ªÊVUd±þþ~ýÁ62ÎÎÎf&ÌÔÚµkëêêôS*++Cq^¤ªJ¥¿N¬þÖuëÖéïZ­V3e±Xô×îMMMp		òù@Ú6Çª$«%JïÝ»§OVÍF²ÂÎêEÕÕÕ¡ÕYµª*/­ªÉê3gÎÈ?µSoo¯Ü­¯¯½Ó0¬áÍápHÏÓÎ¦týúõ¤¤¤ÚÎªUÕ.ðn$«?W¹ILLlhhð¾ôÍÖ­[9»!ü³k×.³Ùl³ÙÖ¯_/)5ÿ§¡ª$+×ºA¤ÔéÓ§¥¿Ê?TUd%Yª*ù¬£££òÏå½÷Þ#YaTU³¬j¯¥[·né'>údÅT~ðDFFÊ¯ÜfeehU-//§ª$kpÕétj§v8ò/F>¼Ë@ö`ýE[IVèç;ß@ÿý÷e¼÷nÉZU533S;W5É:66¦Ï;§Îex÷îÝÙ8£!ÉfÔi¥ÿéþI"còå_RU7Y=®x£®ÏzäÈY:]0ÉN6oÞl6=&Ê·Þzk>¿lu`ª*YOÖmÛ¶©3£::&ÈxõêÕ$+¼ýÉüÉ<&Ê»Ý>o«ªºXUÀ¬'«ÇÆÔô	Ú	-Éoÿú¯ÿ*¿ú§ªMyÿý÷eÊ?üÃ?PU:Y%G[[[åâíÛ·½u6NÊO²Mmßoë[¿ÿû¿(ãÍ7SU:YN§ª¤2 Yá¬¬,	×ï~÷»óð0TU/4Y=z400 E,ÉpBU07U_^µdMIIÒ3A&¬-TUs¬êâ¬<qrr²$ëøøø=dbmm-ÉÍýû÷_íµèèèÈÈÈEUUUÍÃªEU0ÇÉª'É¡Æê­$+4¯¾új|||cc£?øàÉWÉ°yUU³³³;::x§Ì£dZXXÈ9àíÓO?¶*µUòñÇL¦ùSU+++Ýn7ïù¬Syçw~ô£yLdíéé¡ª YIVÌØ»ï¾£âr¹"##¿úê«9y=TU$+Éz$>%ºòòò¾÷½ïysÿÍ7ßhÑ¼ª'NHUµX,TU$+ÉJ¤J¦®_¿þúõë]]]¿÷¿'áúÃþ°¨¨(999&&æÅÙ¢UÕ²²2v@²¬!¦ººº  @¿®UzªdjNNÎ;ï¼óâ·°ªªét:yw¬$kèvèp8ôS:;;­Vë%ÝÝÝk×®UUU¿2¬$kÈJúÖ[o>Z?±££ãÅ'kMMÙlªzýúuÞ$+Éz>øàHA4L÷w§©´´´¸¸øEVÕU«VEDDHUu¹5HV5ôTWWK ~üñÇ2LHH»ó7ÓÕÕU^^Þ××÷«êx_¬$k¨Û¾»vWâw¾óEY,M6I|¯AÂ;??_JsEEUÉJ²6i¨a&-öÛßþöRUãããÙªd%YÃDtttKK~¼e¯¾úêøÑú9VÉJ²×^-99Y«­===111/`¯¥ºº:©ªééé«d%YÃÊW_7áÝwß]³fTØYÝØÙÙÙ¹|ùr©ª»víâÀHV5IJIòúü`¶/o^]]­¶ª¾ø%É°rçÎu¬jyy9; YIVäÄ			ÙÙÙ«d%YÃSFFFÄ7,XpóæÍYúAk×®U§Ub`$+É¤;JÔ¥¥¥ýÛ¿ý[yy¹###gãÕÕÕ©ªêq`¬$kX(]¶lvW«ÇÀuwwÛl6yÚââbª*dgÿüÏÿ,ç11&&&...UÕl6§§§³0dª¡zlX5LßúÖ·ríÀåååTU$+Éj¬±±±Ú]µ©µ¨¨(À§=pà@|||jjjSSÉJ²HUUÚk)111&&FÆ2ä	»»»óòòÔÀ÷ïßg	 YIVÃùû¿ÿû¸¸8É$åõwÞ	°ªÍfª*dE ºººÔVÕ¶ª YIVÃiiiY³fÍk¯½&·`úùçKßÍÎÎæºªHVÕ>þøã^z)''§¨¨èw÷w###¿üòKÿª»»[UUN«d%Yê«¯¾(ýà´)ò¾L&?Î¯mU¥ª YIVCÖÅL`ÁéÓ§gTUóòò¤ªVTTPU¬$«¡UUU½úê«ãââ;æË·»ÝnUU333;::XHVÕ¸ZZZßxãÈÈÈßüæ7ÚôÆÆFòÕW_=÷$JW­Z%UUâªd%Ymûöíùùùjjjþcb·Þüä'Ï­ª»víªÊ¬/Â7£££srrIÖy¥¡¡A¿°Ëå|é¥ÔYúÓ>·ª._¾¥¢¢ÂdõÝn?vìjkk7nÜ(^Ö¬YC²Î	Yò?úÑôSåc/ß[UU¥¶ª²0õJLLÁèèhJJÊ×_17ß|÷òÅíµ×Þ÷]Þ×óÐÙÙiµZ%VwíÚåv»YHÖJÇxÔËÖ­[é¬sâ§?ý©ÇþÀÕÕÕ111S=^ík±Xº»»YHÖ9`2´±üËf;ë¼ÒÓÓ#oä«ÚJzåÊy'°¶UUÂªd3K,VªªªIÖù¦¡¡AÂrÁñññùùù>LmUµZ­lU@²Î±Í79rDrk·ÛIÖùIÚê_~9é¡«ÚVÕª*uîµ¶¶.]ºÔd2%''·µµ¬!D«**E¶««dåLðDéòåË%VÙªd%Y(UU­V+UÉJ²Â'ê/¿ü²~zGGG^^^lllee%g@²¬x¾ÖÖVuv¨¨¨5n·[ÒT&JUåb5HV¾ª¢T´21;;[ª*§U3#9¡Ýu¹2ª$+üLV»Ý®ÆN§SªªÉdTU Yág²FFFj[UóòòÔúa¬ðÇÿù«pÝ¾»*¬Z¬­ªê/á·nÝ:¬±«Õ*Uµºº­ª@²"Ðªj6óòò¸®*¬BUçb5@²" .KUÕÎ$+ÒÒÒ)Uµ®®¥$+ªªQQQRUûúúX @²"Ðªj6¥ª²UHVøïáÃ¥¥¥RU×¯_çÎ¬¨ªZ,¶ªÉJ²ÊívWUUÅÆÆJU`ÉJ²TUÕ±ª'N`iÉJ²¡ªÚl6·ªfdd,X°àå_Þ¾;HVüVUÍÌÌLJJ:zô¨/;÷õõ©ïL&uÍ¸#¬ø_²Ûí¾oUUkooWwä®Ü²0dÅtuu=vFß"9ú³ýÌ#k%Y@²Â¬S^~ùåÈÈH¬ð3YsrrôS$V,XÀþøö·¿­_!¼hÑ"¹èÐ!¬ð4Ôv_jjjzã7~þós Y YIVÉJ²HVd¬$+d%Y Y YIVÉJ²@²@²¬d¬$+$+É YIVÉJ²@²@²¬s¯¯¯ïW^Y°`ÁâÅ÷íÛÇþ(`2Ô   Å$+ü¤2U» SX2@²bÆ:$9Ú××çµßÿþ÷Y8@²bÆ$G=&FFFJseáÉûÛ¿ý[IVý^KÒ_eÊo¼ÁÂþxé¥$Jþó« Âê½~@²b¢££#tÚÛÛY&@²"Pûöí£ªÉÉJ²HVd¬$+d%Y YIVÉJ²HVd¬$+d%Y YIVÉJ²HVd¬³¯­­-777::Úb±Ü¸qd¬ÉÈÈP¯ïäÉYYY$+dÊíF/ééé¼uÚÛÛ·nÝJ²HÖ ¶ÛíO<am0d±o¨»[¶lbß`É(§Ó¹zõêGqÔd@²r¦ÉJ²@²@²¬ddd%Y$+É YIVHV@²¬dddõË;-ZTéééiiiôÝï~7555Ã¨dÞe	sÞåw^~ó9ïFþÿÞ÷¾Ç|pÓl6|²ö÷÷ÿu°ýáþ¡üýµ!EDD:tÈó~øða÷íÛ·ó­tùñlÌyÿ£?ú£W^yÅó^^^.¿öõWeÀyÿòË/eÞÿìÏþ,¸Oûë_ÿÚívv²Î¿ø¿X±b1çýÒ¥Kò«öÿù÷÷/ó­ÏËËÿý÷9ï~øá÷¿ÿcÎ»Óé_ûÿ÷7à¼ÿÏÿüÌû¹sç^Ì#YIVd%YIVd%YIVd%YIVd%YIVd%YIVd%YIVd%YIÖøË¿üËwÞyÇóÞÚÚù_ÿõ_÷ÿþïÿy¿zõª1ßúýìgÕÕÕÆ÷ÍfÌyÿÍo~#¿öÃÃÃ÷ññq÷üÇ$Y=$+$+$ë¼ÒÖÖm±XnÜ¸!Sä699Y¦äää477÷ìjfyÓ5j5£ýlÙ²%&&&--ÍétjÞe~³³³Õ¶¶¶gÞ=z2Í¼Ù^MÖu²Ç'OfeeÉÀn·;vLµµµ7nïÙ7ÔÌò¦+cccòÁBKVãÌûG´oß¾ññqôôtCÍbbâ½÷d ·*i0ïòB>Oh¿êÎõl/Ö?[¸p¡ú¿=ê?ìëßqf7]Ù³gÏþýûµ7Æw)%===ÆüýO2[õ©Âó¾fÍ»wïêÕ®g9=YÛÛÛ·nÝ*èèhm¢~5³¼éª²X­VùW¢ý»1Î¼ËÜúé§òYJ¢åæÍ÷7nÈË.·òko¨y×'«÷ör0t²Ûíö'OÈØd2iÓcbbÂÆ5³¼éÂf³©wµ7ÆwÓúúzôööÊÇCÍû²eËT_U§Ä1Î¼ëÕ®g9(Y#¾¡înÙ²ehhHÝ]²dÉèè¨Z9 ãð^Y=Ã¾é¿ÍPó®;ÕN3ïÞÍÌP¿óÓüÇíå`ÐÎêt:W¯^ýèÑ#mÊæÍ9"¹NÞ³o¨åMôßqæ½´´ôÔ©S2¸ûv^^¡æ]zªÌµnÝº%ýÕPó®OVï¹íå`ÐdMIIñøßÚÚºtéRÉÜÖÖÞ³o¨åMôßqæýñãÇ6MÕj½÷®¡æ½¯¯OUæ]nel¨y×'«÷ör`ß`HVHVHV@²@²@²¬¬÷g±lÙ2ûÃqüøqríÚµi¯®ú2uô;wª»O<»êB§HV ¬tÉ6ABTÒîæÍr«.¥OÁÆÆFmJvv¶DoáóçÏ;wN°nÝ:mÊ'|"S?~¬Kkk«LÑ.¢³¡¡å¬@¸Ñ×ÊÎÎN¹mooW±±1Àêa÷îÝ»<Û+WªúëÔª[^^î1EX¥¸Ö×¬Y#ãÃË¸¶¶÷ Yp044¤===prûöí/ÊÀåry$krr²S¯qqqÒPe 4''GÖcÇÉ[·ny´^yN-ÅõÏ	dÂªª¯§NQýRRP­ãüø±G²jT]Z­=VW]	¦Õ?øàÁ2QBWÆ*?ûì3¢XgÎáHV |<zôhÛ¶mp'OTÑ(SÒNÒ2½Uî^½z5%%E­ÔÝ½·Lìíí]ºtéÆe òÑÑÑ6¨Èêm6GHËóð.$+Vµ]ÔvÖÚÚZlÝºU%ëÅÕ#%>å«j½±ÚDªmdUëûûû=S°Ü^¾|Y+»RUµÝTåå-HV ¬¨´Ûí*üúúúTJXJmÁ½÷´ª£¶Ë¦§§ öQRÄÄD	Ëññqù.«ÇkØ»³ª/ Y0¡6µ¶µµÉí¡C´ÀÛ³gOZZv×åriß¢bÕápx?ÕÐÐÚmxllÌ;>é¬Éàï6"¢¬¬LrssepêÔ©¸¸8	ÎÔÔTí1ÚTùªÜÝ¼y³úÌÈÈôGÐY0µâ×ívË¸¹¹9++KõQ.Éª2OÚ§¶©UíÍ¤úèÍ7åV¦VùJa&¼é¬É?µw~£/¾øB¦<ûæüü|úµ»?V%MµdÄ)Ï3í¬·oß&@ÈÐN@822"ãt:û]ªæJ"ÊãÓÒÒÔ|iñâÅÞÉzüøquEy¼Gg-++S§àHVÀp²²²;¦ÝU§júè£´)ÃÃÃµU­L>räÅ Y Y YÉÉÉÉHVHVHV@²@²@²øåÿÛdSãIEND®B`


÷]ö$ý|¦n°¶æ¿ø¬e¯e-[¶¨eÊòÅõê$·APÿuþóË¯Ó¿Û&emê+¹ïyGÇíÚ´²jß»6ÜË-WëÝîK¹¬R;õ ß¡C$cjÄY·n¬YjæemÆåÓ§O&çË/¿·k×®Õ_Ã_|1ÍMUeaÈ²ÊúÏ?ÿùêò&&&ÊÊ½÷ªGbµ[¥9ßÊ*þ¾yFFÆõë×§úI¦úºCÝ§Q£¼,Ü½WU_UÖm;¸kr×ghnÞ¼é6ô÷õõÉín×W#3y¼Z+«Ympm½lsm²²bUÏ¼É¿õM6Itg5³JÃô½t@e¥ÄÛóÔa+&&fÛ¶mú-ë¥KRRRÂÃÃm6Tjú²j»÷dY.¯®M/ýeÜÆk?gRV7_ýµzU½^FÆwl´9OÞÈÛ³gÏz>¦ªº%z6¹Ó4""BîÝ»'+¥r¯W2''Çm½|iY¯nÉ4ßÜµ<U¶µ²ªGûÕí¿û¶ºûrÿþÊÊåXVÅ¢ÿ'8«¹P¿^&­¸¸8m½ü¯ÙH?MUV?ÕHÚÜ.ìVV5aêfk_QÑã¯×¡Y?ºuÆ¨®¨;ê­©tRU³»V>ãÌÌLµ·òÑ£GnuùòåùÔ§H_eáç?ÿ¹ÊªzÜÕó±Z­^oÜ:uÊë .Ú¾»v%Óµ¹¹Ym¹C°aÃYyõêUýe(+(+QYeøáQH3,«ú¬Æ/zeáèÑ£3/«:(W]Ã¹sç<Ë*EQû,G'mÝºuæe]µjÓéáOG§)ëûï¿¯;¦)·JÂ&Ë^GØuUÚ-Wy>®+ÕA=êrdå~¨M-..~íµ×öìÙ#ïÊ¸®Ür¹ÊõÃ·'ù¨Ä[~"Úòjï¯Ú¼Rq5Yý'þò¿tûY¤¤¤hËr;=ËªerÕöÙ»]²²²ú;³ªêQG©ÎËzéÒ¥Ì¬F£QÞÕOðàÁôerÛª®P&_XÖ>,Y*7LUPíÅTÇ+ÉÈ®¶ú6µG_åhª'#©[¨«òúøí³Ér#""änúòåÔ/ëÖ­KLLÔ¹.µ·ê01¹c¡êºû¶ÛW	X¾w­÷Ï<Tß´i×²ÊØª¾Õ=ôôtÕlÊÊåUV´=jSª¾²Ríðª¬ª|ª)«×yh3«öYa÷ÙóÝOµaY%rÔÔTý#±$	Ø½÷´5òQéö®ôOíÛRªÃå]YÐv¬Úl6·IW>$kÌf³6CÇÄÄHJ/_¾¬Êg;_µGõ%ánÏq­×§Ó<þ¶*÷#GfXVõí¨]òªyWn¿*´¾ÕPV,²ªýmnu¹qãFff¦vüOWÏ¶Ô_FUSÂ£]Æb±¸UuÅ³seõ|4X=ÓÆ­¬kÖ¬wµ1næeÊíê¸¯¾úÊUüÔíTÓÅaÑí¬j×b£.,½TÏ1·êù3n¾oÞ¼©Ëj¦Ú)W®=ëuW«öEßyçý»7ov»z>:ªÙmÓM_VuÔ¶FîXhaV¼3³²byU=N(S©´PJ#cüsW5iÿÕ£2KiOHáR*¨BY³sçNý^Ò©:7UYµÝê#Ï²ª«j?«|Hívõ¹¬jdÔöûVV5á©£~ÔËÚMj½vêÛQ_W>«|êq]µºººÔ±ÁÚñ_²1½ØÈí¬SmI¯Û±Êú§Çè/#·ÖkMgòh°PÏªkv²²byUìØ±Ãóh[)Ú?úlòÙêAcÏ³IhZÊ»úgÝ¸êqW¯Ó¡ªÑTçr+«nê¡i%,,læûYÝÎÕðìù©þôG±Î¤¬úçÃ¨3`ÈäêÁXõ¯:vWHV+«zÌàÂO«Õ*wÔ$­ÛªfÁ©ûõzd¯×KªçòÎ<u¾UZÃílË«¬Ï&÷nÜ¸qÕªUÒ*³Ùäö|JAdYVÂ&Qg@Ôvºý÷ÔFgç*«ÿ¶^6Õ"d[%1Vþ´õ2RKêäf¤¤¤Ìêù¬Ú9åÞ|êtBÚ£ÙÏ&ÆÜ²eÊ°¬×Î'%E;ªVeÚg©ãÕ)|=T×Óö<~fÍÏÏöüêôZYÛÚÚ¦ù!z=[¤µ[¿ÕÎÔé«ì¶éä'K=J¡nöTeÕ¶°¬¯©©¡¬ ¬ÀP~fddÌvðmªegxB;A®Û=jvÚ¨Û	fK=mFWî©x>GEÛ´CÔÝ£Ñ¨¿mr?`ªÓ>¾X^É]¹[ ½+ZîÉ'jÇMÓcÏÊJ/µÝ½n÷ÒÜÎÈ¯În¨ÏÌO'	PV>V_éÖ­[·gÏYºÚ;Í·<ó<ß²:´Øí	¬3¿©^?ºmÛ¶^Ã4ç§(+Ê(+Ê(+ÊeÊeÊeðù&$äóÏ?×¯yðàÁ¦IùÏ?¾±±Q.ùÅ_LsU###QQQ[·nÝ>éâÅò)C÷îm¤]³ÐåòòéÚSÂÂÂV­Z%·gxxX¿ÞÍ¬¾ëÜTÒôõLêËZ__ïvù±±1µ³~ýú~ÇËµeddèsµk×.ýeÆÇÇ'&&Ô²,È233§J¾ÖK¹ý²üáNuÙÉM@Y)i¥T1hE9vì¼mkkóüDÊª¿y7''gqYÍÁ===ÓUÝìÀuæ7 ¬þD__ÝnöövIÈíÛ·§Yý)«Û XTT4Õ%Ï;'3t||üèèèÑ£G%·nÝ¦¬«V­ÓÕëM@Y)Dé¼=tèÐ£Gda``À­¬ãããë'iK®PõHò41«=uê~MssóÈÈÛÅ:;;µG¤%®E­¿÷®¬·Z­á<ö¬,çææNSÖYí|áM@YéìÞ½[ÔÔÔTÏUÜxV3«Óé,´ÝÕ%e¡°°PûDé¨þúÕ(éµ999.Ëÿuæ7e^@¦U5¹>~LzÕÿGUÕ0*(£Ñ8Íå×®]®¬Äòáüî»ï"""6lØ ÝõfíM@YïîÝ»'-9rä¼Õ¦ÜÏ*W%½T¹eeaïÞ½*án|çw<?]ÿ­>û÷ïå=öø?³Îö¦ ¬O>fôõõ=yòD=ð²¨øÉdùÂAp¶eÚÉ&÷ü/ë¬n*Êx/Yss³Ûrrr²Ø._¾ìù)]]]²^ê~	Uh¹rY¶ÙlÓ*;Î©Â©ÆÖ-[¶ø_ÖYÝTpwÿþ¯ëÇÆÆ¤E&Z¯Pûbkjj¦¿~uØÑwß§­PI´JJJ$999òÑÑÑQÛ<§¿¹àÃM@Y ¬PV(+ ¬PV(ëøòË/×®]@xã7þë¿þkqõþá6nÜx ðù^V¾ÈÊú£ýè÷wÞy²@Y)+²RVe¥¬PV(+ePVÊe²RVe¥¬ÊJY ¬@Y)+²RV(+²(+ePVÊe¥¬ÊJY²@Y ¬@Y)+ÊJY²æÔ;w(+e@WWWFFÅbq¹²üRQQa2RSS¯ÂÌJY¾ëîîNOO,))	ÎIYt´¼¼Ü`0Øl¶®®®`¾©ì:::$¨Õ²²² Ü±JYt´´´Ôh4¦¥¥ç^UÊX4º»»%¨2ªÿ¨JYA=ª©½ª×®][²KGGGBBÑh¬¨¨Zt·²hT-..QÕn·÷öö.Òï²BKKÍf3LÚçªRVÀ"044´sçNU322º»»û·CY<ªZ,£ÑXYY¹¨GUÊXøQ5??_FÕ¬¬¬%0ªRVÀBjmm5Í2ª.ö½ª°ð£jaa¡:88_`²RVX4ZZZ¬V«ªUUUKlT¥¬ùUÔ^Õ%9ªRVÀüijjJJJ2ÍKþ¥¬9Ôßß+£jNNÎÒU)+`Î9sÆjµFGG×ÖÖ.ï²ohh(///$$dùª0WêêêÌfórU)+ ðÔ^UUúúúçF ¬À¨©©µZ­­­­Ëy;PV¿d<U/Æ×*§¬H]]ªeªà¯ÞÞÞ¼¼¼þþ~6eø®ººÚh4Úl¶¶Æâ+ëÅå>e U³²²CIIËåb,¾²§¤¤PVµµµ&IFÕ¶Æb-ë¾û¾üòK­¬1"##ßï=~0×£jff¦ª;wîdT]ÄeHMMÐÊzÌCFFÝnçg	s§ªªÊh4&$$8N¶Æâ.kNNÎåËÿÿâÑ`XÝÝÝjT---å¹ªK¡¬!²À|ª¨¨P£*/²êËÌó¦««+==]FUÃÁ¨JY¾)//Q5))éÚµkl%[VÎó@R*AUU%±lÊðTJSÓÒÒU)+eªåååª²£*e¥¬N'£*e¥¬.Ëáp0ªRVÊU+++U)+eªÝÝÝlÊJYQ²RVQµ°°Q²RV«Õj2U)+eÀªv»½¯¯BY)+ø®¡¡AªÕÕÕlÊJYÀwùùù!!!ª²@FÕØIµµµlÊJY £jnn.£*e¥¬À¨JY)+OeHeT¥¬@&TFUÊJY eTÍÏÏdPVÊ¾«¯¯7Í2ª644°5(+eßÉxZTTd0U)+eªQ²RVðÚ«*£jAASVÊ~ijjQÕjµ2ªRVÊ~!UFUXÙ«JY)+øE&T³Ù,Ó*£*e¥¬àþþ~u`UØ ²ïÔiU)+eõõõegg3ªRVÊPYYi2RSSN'[²RVð]oo¯Ýn7ååå###lÊJYÀw42ªvtt°5(+e¿FÕ¬¬¬²²2FUÊJYÀ/j¯ªÕjmmmekPVÊ¾ëîîNOOQµ¸¸)+eßFUÊJYÀ_6M²Ê¨JY)+øÅårÆ®®.6e¥¬à»U%«ä`ÊJYÀ¯Qµ¨¨È`0È¨ÚÝÝÍ¡¬|çt:U)+e*..fT¥¬3ª&$$DGGWVV2ªRVÊ~ªCFÕ;wî°A(+eßuttX,ÉjUU[|722¢íU½ví|×ÚÚ*£ªÑhQ½ª ¬à;Ëåp8"##ív;UAYÀ/MMMIIIª ¬à¯¡¡¡ÂÂBÁ×××Çeß544ÍæØØØúúz¶(+ø5ª¨Qµ¿¿Ê¾;sæÅbQµ©©­ÊUsrrØ«Ê~©¯¯·Z­ÑÑÑµµµlPVÀ¨*oUAYÀ/2ªZ,Y`k²_£jnnnHHHQQ,³A@Y)+ßÕÖÖ'q0(+eàþþþììlµWQ²ðKee¥Éd²Z­N§­ÊJYø®··733½ª ¬@`FU£ÑÐÚÚÊÖe¥¬üUÃá`Te¥¬0ª&%%]»v­lZZZÒÓÓåd±XõýÊ`ª¥¥¥¼Vyª­­®««Î;wòóóm6Ûâ+e°Ä3ª9ÏggddTVVRVÊ ¸tuu¥§§3ª¹ÁÁA)«ÛÊªªª¢¢"Êúü3CB~ñ_¨õë×ËB»ÌõçÏKII¡¬ægTMKKcTrr§'66Öí±_ùñPÖÿ-ë_~©RSSe¡±±Qd¹¾¾^~×)+¹U###å¿3£ê¢]\¬½ÛÛÛ+SìâNT ËÚÙÙ)áTAUêôÖ'N·÷ïßWe¡¬U¡éëëKHHÈÊÊªªª*++¬ÊÏqñ~;,ëÄÄU£¢¢TY?þøcYsþüyýÌJY0ªÂËåª¬¬,***))YìgðG:$r·qÃZYe~ððð-[¶PVªXªæ¤¬^gV0§£ªL<ªX.3«ªìðð0e0£jFFFww7[Ë¨¬'O<QuNõ÷÷_¹rEÞ²)HY/_¾¬ø ÊBQQ,L¦]»v:uJ]f¶3ëÕ«WSRR¤Ç6íúõëtTªv»QU¿Yrsså_®Lð±±±YYYlE_Ö±±1Õ×æææññqíz¥¯~ø¡oeW×#mNLL6ÙÙÙX&®]»$ý¨®®fkèÉ0©jêr¹å]6Ë¢/«Ê*ËN§SO>-o»ºº|+«ÞªU«þð?xóÞïñ³üLVZZj0dTíëëcèIPåÞ~HÍeµZ¯ÂÆYÜeðàDNÚ©­¹~ýº¬9þ¼,÷÷÷«Çµùu¶§¡Ø±c,zø»¿û;fVQu9ëèèHOOw[_[[ËÆYÄeøyÎ¯TmÍ½÷Ô;Û+|æææ>~üý¬£*<ÉZÅâv$´v±ru®È(¼ûvõ¤Ê,·QLFÕØØXf¯ÊÈÈp8Ú»2ÜKk9²zát:7mÚôðáCu,·QõÀ¹¹¹ª3![ÉjµJ_eÄÏÎÎ6Ír¿Í²DÊÚÖÖvîÜ¹@5..N¤e®®.Í¶Ø÷ªÊ¼xðàÁ¢¢¢²²²ù9çâÐÐP]]]EEøL«Kmfîß¿_[¡3:éÂ)W.KF.£ÑÕÛÛ»¨ïÈÔWYYYRR]SSÃÏ²ÎZcccnnn½vÞàææfy+s§4U+ëT'| ¬À2×ÑÑ¡ªý´JòÈÀªÿÖäûZÔ÷°0eëvÞºuK?¿îÞ½[ÿ®v>&Ê@r82ª.½ªwîÜIHHp»sPXXXUUÅÏ²Î×²&&&êgVí¬¸råJRRÑh£ª6¡z>»´´´´¢¢7eõ±¬7oÞ¿û÷ïË»gÏefàuTÝ¹s§Á`QUæ¼¥ôÉý9åüWlhhàNYgMíX=RÝ¼y³,<úTÖ?þX99Ê,s­­­ETÏU¹Y2/ÌÎÎniiñùzd<MHHhjjeI¬ff&¯ÉCYtûömy;>>µgÏ·MÿÌTÊ,Qµ¨¨(222///¨FU¹UÒTÉaoooUUUtt´?Oû©««³ÙlòmZ­Öââbù®ùÑSÖYòäIú¤|odf-))QSV`Ùª¯¯QÕd2IxmÕ¿²©Óé"bgV·SûJG%´s&Ê,.KBÁPXXç1¨¬¬ÉÒmeZZg2Âubb"yzb«5""¢~,¯[·²ËL¨f³9::Z;GfÁ+W®ÏS<«ªªvîÜé¶Òf³ÍÏé@Y_üúçSÍ¬S½^e*)h~~¾ª2°jÏU­¨¨0òÿ!aR0Ô«»»[Â¯? WF¹7ÀaGXÈ²ªiU=uUöªÉdr[3Õé)+°ô´´´X,ØØXýq¶2¿ÊÊ®®.mXéwp.È-Û#·¶´´TB«î¬¬O>]×Ã¡¬@RæææFFF¸U355Õí	-rÏ;HÎ¢ 7¬°°0++K&lÆÂU¿·USí)7?wkjj(+°L9räõ×_ã7¼>%Ôd2¹­©®®±Ý@Y§ÔÞÞ.)½zõª¾¬|i9Ê­_ýêWF£144TÊºzõjýYé5IIInã Ãá(++cë²N÷*r/^ÌÉÉ©¯¯?yò¤:,;vLåþ,eªªª+VDGG·¶¶þ~òÔ¬V«gÓÒÒ´'ÞÈåSxr(ëtOivöôôh»]GGGeXªúûûóóó%«2êO­ÐÒÒàyù¢¢"Ég·Ûccc9Pu:?^×¬Y#5Ú±ÁúôSV`	¨®®¡Óf³ôÑG§ôúYÝÝÝuuuAxÖ Ê:66¶eËÉçýû÷ÕÃÂ999n<y²AèÖ­[³_üËUQQ!Ã¨þ^gV²ÎbkOO¼½tézWÍ¯úç³òú¬@°ùì³ÏV®úÒK/;wn£jllljjªv8Ò;wdx­¯¯Wïö÷÷§¥¥ñ¢¤ ¬¾u`` &&Fÿ"¬^gV^*CZVVæôüDÞ>®½½½YYYF£±¼¼ÜíEMMM&)333//OvîÜÉ9@Ysl°VVfV È­^½úÿñõkÞ~ûíäää©._UU%MµÙlSòwhhHúzæÌýÊ°²2³AN&TUõk8íyI)¥ÝnW£ªÛ§`fðGÍÍÍú5?ýéOãããõkFFFÔ9ôSSSµóýµ§§gbb²A"##Ãd2iG_¹r%,,ì³Ï>Ó.ÐÛÛn0<÷ª¿²<yRMV·µ¸¸XÞ¶··SV ¸7ÞxCÂöÖ[onÞ¼Yû¨ÔTFU»Ý</¤,»²K8?ØÁ²sê«¯¾úà~ò9sF^¥µ¥¥¥ªÀÏ¬¼°Ø<xP*£*Ç÷ÎÎÎ÷'åNªN-«SóË2eæL¢ÒHÁðÊ+¯üæ7¿yáå322äS¼¾Lù.ëøø¸ÛÀÒC1³bõêÕò7(åé_¬MíUÍÌÌäE¿`)«×£(+° Þ÷]·FDDÈ¯VU%«UUUìU(+e¼º½ÎÌ7äOÒó1aUåìU±¬ò§»k×.Ï²QV`Þ¬2&&Æm¥üIþêW¿ÒÞíèè°ÙlÑÑÑ555ª@ðÎ¬úÊ²ÉdRG0©eÊÌ³Ù,t¿ûÝï´5ë×¯×v¹CÜ××ÇæSYy4XÒTùÝ¿¿¼$ïªÓÊ¨*ï²W ¬ßüæ7aaa!ÏIM¥£ÅÅÅ!;;Q ¬p÷í·ß>xð`njj²X,ÑÑÑÕÕÕªe¥¬ÀHLLTöÊÛÕ«WwvvNsaËURRÂ¨,Á²ÊÝdÊøOFÏ+V|óÍ7²,M]³fMXXØTv:			ìU_Y/^¼£NMMÕÖ?.ïSVÀ¿ýíoåOéÜ¹súÚmÛ¶yª2ªò`QõéÓ§÷îõPeMLLÔ¯iii±Z­ÕÊÊJFU`Q×ºæL«¡¡¡n.½üòË6lPËjTåuUÊJY?N¥o¿ýö[o½õÏÿüÏ^/°bÅù¨þò!!!j·kCCCll¬Édª®®fKK­¬ò§þþûïSV`Vbbbäo'v,X,ÏË>|X>´fÍþð¯¿þº,Ëªùùùêµ9X²eÝ·o~Í'O(+0äääÐÐÐo¿ýV½ûÿùòî»ï¾ëyÉÎÎN¹ðË/¿¼nÝ:YÕ¨*jkkÙÀ)«ÓéÜ2I	|êÔ))ë´WA¯©©5ò%)+0ðððO>ùD¿FþFã4Â¨,Ù²>úTÿ²ç'OÉÉÉáÑ``æ´g©jþþïÿ~åÊS]^&TFU`ÉÕóµäÚÚÚúûûeaxx²¿<aVVVbbâüãï¿ÿÞó2º=yFªùúë¯^RÆSRUåRVuúoµ|áÂY¾uëeÅ2'Ó§ü-H;-KXX§ÚþT:4éÝwßUOªùáèyRý¨ÚÐÐÀxY>*ÿbbbÔ	ä?×ððð»wïRV,Oò· æKmÍ+¯¼"üôÓO%ºêîihh¨Ûo´Q5??pp,å²>zôèôéÓQQQjÍùóçåïÿæÍ²<<<ìp8äÝ¹$eÅr#c¨ÛHò"^þýä)£*°|ËúðáÃ5kÖ¨vuuI>ïß¿/ÿ5¾þúkýÅÆÇÇY±<%''ÇÅÅ¹­Ô?Áfz2ªÚívFU`yÍ¬Bjêt:/^¼(ÿ.KFXYèïï JzeùÈ#KXccãÀÀ×òÉ'+V¬ÐP8i&W[]]m2,£*°ìÊ*ÿ&UYÕ.U«Z/e½yó¦,ìÞ½²béyN&QI¬çeÑhT/¤zøða	­v¦ßªCCClg`yUíFQU_V¥¦¦F,ë¿øâÊ%ÕíÛ·Ë²üªGFFJ/öÛßþVÊª½~ðé¯¶²²RªMMMld`Î¬ÚcÂêû¶I[·nå¨¨(u¥JËª~×S*ß~û­Û+Õ¸éííÍÌÌdT(+¯uåè×¿þµçîR5_õUß®PFUmZ[[Ù¼eý_7oÞ¼xñ¢¶ÿµ±±²bñòúe``@~Ã¥¯ú+W®üó?ÿóÙ~mTu8ªeî4L² U²bqÙ»woXXvhÒ§~êy¹@DDöîÏ~ö3õ3FU²¬¬N§Sþ³´··«wïÞ½«n¢¬Xýö[ùÕMLLT»E³²²ä]i­ÛÅd`U%ýÙýL«ê5fþUºººÒÓÓUÊ:ÝY#<çÛPVÌµ¸¸¸W^yE¿æ­·ÞZ½zµ×¿ûî»¯¾úªÅb©¨¨ù(//Q5))Q ¬Ó7x×®]^sKY<Î;·yóæäää>øàÆtµÔºº:¯Ï¨ñAww·ª¡´´tdd@Y½xüø±:YöèÚË=ztãÆjgÕõë×õ¯äJY± 8 ¬X­Ö°°°'N¸]Æd2¹=yæÓO?KúÿÕe®QÕf³]»v@Y§;jéöíÛêõYÕ»ë×¯WR/ô¡êj6¥ÁhppPùÙgik~úÓ®Òåré/öãÿXjßï¿ÿ><<|Ýºuþ|é+W®¤¦¦Ê¨Z\Ì^U²Î:´þ¹Z>yò¤¼;::Ê£Á_õUTTÛJ)kss³ÛÊ_~Y¶o¿ýö+"##§?ÉÃôÊÊÊ¤©iiiªeµ÷îél#ËIIIÁ áp8Þ|óM·&éo¾ñ¼ð'|òúë¯ÇÆÆj/HîõµWÕm2@Yg:°¾ÿþûúwSSS)+Ì¦2êZjmm5S½Zª´çªvtt°ñÊê¶¶6IéÀÀ¾¬999Á#99ù¥^RCê"""¦9Ó¯Ï$¥iiiåååªeõÑÙ³g¥£ê ¦Û·oË»555sw&Êedd¬RFUÉjAAA`¯|dd¤¬¬L*e½rå[ ¬>¾ÊMLLÌ¹sç<_úfÇÝËGWWWjjªÑhiçª×º|çr¹JKK¥©2ß¹sPVÊø®££#!!A²Z]]Í¨PÖull,$$äã?¦¬X¤£2ªFFFfffvww³AÊø²ª£nÞ¼©_ùäÉÊyðWõW!Ïê4¿Óªiii&éàÁªeXYN§vºàúúzù&ÿhdAö`ý¶RVÌuVC'©å¹UªìUø²kËêwïÞ3RVLÃ-¥[·n£Éµ¥¥%))Éh4VVV2ª|YÝ^ñF½>ëñãÇý9]ðõë×Ífsxxxrrr[[eÅË*5Õ¯Qk¿ÄÐÐPqq±Á`ÈÊÊêêêbÛ²îÚµKþÕ××GDD¬Zµª~,oÚ´iVWâÄ	Y8räü¯¤¬aYÿíßþmîÊ*¿Ì%66¶®®­`ÎËê¶3Õ:I;Á¡ÍfÕµÅÄÄ¨s9ÅÅÅÉB»ÝÎÏú²êûu:z4XFÕÂÂBU³³³ûûûÙÔæ¼¬mmmÒÑöövùGÖÓÓãYÖÙ?<<Ümùo¾ù&eÞK/½¤îÞ½úê«RAµì6Åú@-¿ÌÑÑÑgÎa#²Êÿ5ª! eÓ#""x43ôòË/ëuãgVÕ¨×××Çæ0eøða¿Ø@õµ×^SË2eÅükhhßa³Ù[ËÖ0¯3«~xÕÊxk¬´Ûí³ºªmÛ¶?~cKY1äÎbNNüÞfggóYVõâ¬÷ïße¹§/eØ·o¬<räÈ¬®ª½½íÚµaaar=W¯^¥¬7555æIõõõl=ùÇ¯ÕQ¾)A®¯¯OªyyyCCClÁUVù÷Ï9°XÔÕÕL&¹GØÐÐÀÖeåUä°èFÕÂÂB«²RVø¥¦¦FFUÅÒÒÒÂÖ@Y)+üU³³³CQQUPVÊ¿TWWË¨jµZN'[e¥¬ð]oo¯ÝnQµ¤¤Qe¥¬ðËÁ£££Ø(+eïúúú233ÕÀl²Â¯QÕh4Ê¨ÚÑÑÁÖ@Y)+|×ÕÕa0UPVÊ¿8pÀd2É¨ÊÀ(+e¿£jzzºªååå###l²ÂGÒQµWÕf³±We¥¬ðËµk×ÒÒÒdT­¨¨`T@Y)+üR^^!ek ¬¾ëêêJMM¬2ª ¬~)++¦¦¥¥uww³5PVÊ¿FUuðÁUPVÊß¹Ã!MMMMå`²Â/N§3)))22RFUI,e¥¬ðT-,,QÕn·³We¥¬ðKKKÕj52ª²5PVÊª]]]l²"£ªÑY±be¥¬ÁÁA5ª¦§§L&Ô¨¨¨ÃÊ²¼e+ ¬3ÒÐÐ+sjUUÕÈÈÊªþ*®l(²âÅ£j~~¾T3++«··W­ôPer¥¬(+eÅFUÉTYY©?­gYW¯^MYPVÊ)¹¢¢"¥Ýn×FUzì7**JßZÊ²RVx×ÔÔ £jmmíTgV)¤ßxã6ÊJYñ'vîÜ9ÃÓ*iM>ú­²RVxUccceTek ¬UsssûúúØ (+eïÌfstttMM[e¥¬ðkTÍÏÏQ5;;Qe¥¬À¨æÌ¶ÊJYQµ¿¿²RVøN&Tó$FU²Â/2æååÈ[FU²Â_T«²RVL__ßàà Ûe¥¬PV(+ePVÊe²RVe¥¬ÊJY ¬@Y)+²RV(+²(+e²@Y)+²RVe¥¬PV(+ePVÊe²RVe¥¬ÊJY ¬@Y)+²RV(+²(+e²@Y)+²RVe¥¬,É²^½z5%%%<<Üf³]¿~²(«_âããÕí;uêTbb"ePÖYµjÕþð0¡¡¡ï½÷?KeÎÎÎ;vÈÂÿõ YµÛíü,u¦FGGsss?~Ì£ÁÊ:k!Ï©w<x°ûöááaPV9ÎM6=|øgÝ(kÄÅÅèPVeåLÊJY±êêê***äíàà e¥¬ðÝµk×¬V«Ýn/--ÍÎÎ6Íl²Â.KRZYY©­©®®fr@Y)+|ÑÚÚî¶RÖ455±qPVÊY;sæL^^ÛÊüüüºº:6ÊJY1kÝÝÝ±±±CCCÚõøðµk×Ø8(+e/dBÍÌÌìííå¾¾¾¬¬,Ï)(+eÅLL&Å]TT$c+e¥¬ð¬RY¶ÊJY ¬PVÊ ¬@Y)+ÊJY²@Y ¬@Y)+²RV(+ePVÊ ¬Êe¥¬ÊJY ¬PVÊ ¬@Y)+ÊJY²@Y ¬@Y)+²RV(+ePVÊ ¬Êe¥¬ÊJY ¬PVÊ ¬@Y)+ÊJY²@Y ¬@Y)+².â²îÞ½û¥^(«ÕºnÝºx,Åòñlù'¿óòÏv?øÁÞ|óM¶ÃBýÃíØë4¾¬÷îÝû?ö×ý×ò_æÿ`ÞýË¿üKHHÈÞ½ÙóO²ú£ýí0ÿþöoÿvÕªUlù÷Í7ßÈ?ýìg½Úÿ÷YÜeÿôOÿ´aÃgwwîÜ_ôÿþïÿfSÌ¿ôôôO?ýí0ÿä±Édb;Ì¿ÿùÿ8óóå(+e¥¬²RVÊJYAY)+(+ee¥¬²RVÊÊJYAY)+eý×ýè£øµ¿ûÝï¾ÿþ6Åüû¿ù°æßüÇ¬_¿í0ÿ&&&äÎ¯ýkÊÀâCY ¬PÖ`óðáÃ¸¸8íÝë×¯Íæðððäää¶¶6~3æ[_øeâêÕ«)))²m6ls¶ü|êééY¿~½ÚÔíííó¹ñiYe+'%%üï·âÄ	Y8räÈÖ­[ù¥Slm~áøøxu^ÙS§N%&&²åçäS,?ùÜøË´¬7o¾÷®þl÷	YÓßµÇkó¿­Zµ-¿ .`³Ùæsã/ëý¬ú4ááá^1ØÚüÂ/7;vì`ËÏ³ñññ5kÖÈoþ©S§æsãSÖ?Ó#""øSlm~áÑÑÑÜÜÜÇ³åÄÅ_íµùÜøË¨¬!ÏyýG#ÛllL=J ~;lí/+?yóàÁíÛ·³åPçmã3³þÑ¶mÛ?.òVî]ò8§ØÚüÂ/N§sÓ¦M>dËÏ¿øøøgGhËOa>7>eý£öööµk×Ífù1ðK9§ØÚüÂ/qqq!:lùùtýúõäädV7nÜ¨3·Ï" ¬PV(+ ¬PV(+ ¬PV(+PV(+Àý¬_¿>wRýÉ'eÍwß7ÍåÓÓÓ§¿Î	¹Ø=Ô»?wkjjØÚe/æLJínÜ¸!oÕkcé+ØÜÜ¬­IJJôæO:þ|cc£ý÷ß×Öìß¿_Ö<zôHûöövY£½ºÎsçÎ±ýÊ,5O>Õ]]]ò¶³³S-«JÕÅäÝû÷ïËÛ7ªúWUS¬Ãáp[#V×æàÍ7Ëò±cÇdùÈ#ü,Ê,ê&Å­[·¤p<·===.ËåVV³Ù,©êë5kdBIÕÀzâÄ	YsóæM·©W®S«øèè¨þ:PV`éP£ªôõôéÓj¾ªÇx=zäVVmU¯¼­=VîÝ»w=ßM«¿ð¡Cd¥DWU>,kTeáìÙ³ü Ê,>ÜµkîÔ©S*2qJídA¦LÏ²Ê»/_SêîÝ»WVÞ¾íÚµ[·nýmÙ²E=¬>7''Ç-Òr=üÊ,)ÍÍÍÚ!Ej?ë#GdaÇª¬.|ÊGÕãÆj©¶U=|ïÞ=·pÊ,o/]º¤»2ªj;©@Y%E5277WÅ¯¯¯O%Pb)c«,h3¨ºÚ/kµZÏORÇ(©åÄÄ|,«Ëk±çÌª>²KÚÕzõêUyôèQ-xûöí[·nö®ËåÒ>Eeµ¾¾ÞóªÕaÃãããùdf(+°þnCBÕBJJ,>zÍ5NÅ¢]FÛ*w·mÛ¦RÉøøx¯_ ¬À² øå¶¶¶ÄÄD5Êz)«jLÚ®Vu4GoÜ¸!oåbê!_X§73+@Y¥O¤?æèë¯¿5ýýýÏ?£&##CåPÿèî£GÔJ©©VV)®¬<Ïvfíéé¦Êe°hh' |úô©199Ùét¾ð³Î;§Æ¢ Ýºuv»]ÈårÉ¢¢¢<ËzòäIuE¹¼ÛÌZ\¬N7ÁO ¬À²xâÄ	í]uª¦_þòÚÑÑQ·±U=|üøqS²@Y ¬²@Y ¬PV@Y ¬PV@Y ¬PV@Y ¬PV(+ðÉÿþ>¬¬ÇIEND®B`


反趋势正态 Q-Q 图


x=9s/ËáùøãæÜÃárïáÂó¾Ï9÷=0l¬(+Ê(+Ê(+ÊeÊeÊxtçÎÍvðàA÷/:uª¡¡aÿTl¶ËÔÔT_¾÷äÉ²p½yæèè¨ûÈÏÏ9<<,W÷íÛç~³W®íIEEEQQ|ï¶mÛÆ»ceee²@OOÏëÖ­~¯ºÃ_¸pÁ÷åKJJtB~¡ò£ÿÙív÷µjY&99YÖLö8äK²ÜaþÖ@Yñ=cÌsÎ9#ÿèCBBGddd^^^gg§å½Ïw§ùðÃÝ¿¤AÊÌÌìêêZ3Æüï²´´T¾ZUUåûcq_@nÓo©­­Ícù£ÿÓåRVÌ²Ü`kkëàà LTVV·&W/]ºäñ!Ë1Ï	KLLÊoP¸÷ÅäÅL®R¬çgÇÈUù¹ñññãý¾¾>Y¾  ÀûÕØØ(çææÊôÈÈû+ô·¿zõjïÅübèÜ¹srµ»»ÛãëþºAY1+Êº÷nù_ÜÒÒâY/^ìt:+ÆÈL¹Móú¯¶©©ÉòYf^¼xQÿË,½+¹MÿÊ*·)_­®®6æÜ¼ySæ»/,?KÈ¿ûþþ~¹jFoAfZ¾¥¹¹Yoaa¡#·lÙ"z·ãââ|üx/«äùÅ1æÞëj1æ¼óÎ;2G¦=U&6lØ 2bõ<00 Óóv1æ+Cpãqå!ÓÆv¼Ç"7(ãrY2''ÇýååÖ²ÝÜË=×ùeÅÃ)«ÔN7Ç;vL["ØØX)£ßË*ÆèG¦ËËËÝcpôèQËO®:tÈË]ÕDÉ0Å²Êü·ÞzKüty			2óàÁºÔ¸W:Ì2$ýÒAª®½ûNæ···ë¸Ü³Ê´ñRC~ÇaeÒ_èË´´4éúxß"BÝ=ôn0«W¯êk­¬em[V ¼Àò²¡­­Í²i¡³³Sæ/Î<yõø²½Ú(«Y­Æ|ùÍÓ³¢¬2ÏüÃMOOèNjÌ*3÷Ò2o÷[Ð-Ü¼y³9ÃËzáÂäädÃ¤ôRVcÇLËòzk2$2/c^Ëík~dº®®NR*WwíÚ¥>Úã²U÷+ËpºjÕ*ñ£/eµ8uêÜ¦nGiËccóäòúõër©¯,±×nÉîí42ÑÕÕ%3¥r¿DdVVe¾üh¯÷ÄËû£ÓãeÛ(«®a½ÿú"éæÍ³«¬111æOçË(::Ú/ÿeÔb WV6Rf^ØRVa»ïn?QÑîÃ_÷WRS-¥­5ºÝ¤lé¢ü»7¶ëí8p@ÕË¾¾>ý´î#BKY½4F»¢/;´ßãè¤_Õ-Fù4ÆkÖ¬Ñ½·oß¶|W½¼zõ¦ß"Õã°4«ºÝÕý·Ý[^*ÉWËÊÊ<ÄåKyyyÆlÙ²ÅYåW kF~#+W®æe(+(+fEYeð7áQH>Uÿ9jidÔÛÛÛ+EEE¾UÕ[ÐYÊ*ÿë5Tc6mÚäY¥2,3FËª»KÓÒÒ*L¤Ã¬û¥¯rµ¿¿???_ú#$·ïÞºuëtZBFÊHØdÚãVz¯wØX?zûv]QUU¥õèz,ÕË/¿¬[Se¾xñâ½÷¯ÌäËåÒ7ñÝÉW%Þò76ûGiÅõE®ó7êKóo<99Ùûé^V«qdeÊÊ¹VVóU÷êéö@éÁe5oõ2f		«Æ£===ÞËnÙª7hóÜË*9lkk3«¶oß®WÏ9ã¾mÓüêA_ÈÀZ:·jÕ*éõ0"/epk°dIV<|­ îÅÔ¡ðèè¨®g]Æ=Çkl;Õa´®=zËãöÛcåÉýòãt;LÄÆÆ&$$¹555Ë-ÓÚÉ¥&/_¨ª££ÃòSd,Ý|ì´eÓzzºÇ²ê=ë+YÏÚlÊÊÙRV¸ûºÆ3^e¦î¯¬Z>íâ´ÕãHÅÇ1«ñÖÁî½û;ÍoÔQ¹gÈ?nY]rU¸ÕôÐYmnCÖÃÓâY%róX	¬««Ë#_5ÞJ+ä>èt)¥,WeÂØ±déêv:Æ:22RRZ__¯Êc;_pËc,ï²õøv÷7hk¹eïcYõáèy¤:Þû¯ÖÁ·y[=@YñÐÊªÂ,o±omm]³fqü·Õ÷AÑjJebbb,eÕÿøîs/«ûÖ`§¥¬¡¡¡rµ··×`ù^V÷Ãô¥ù[èÖlóûRî¹íö8f5VÞ7o>ìÕùéÚÐÇâÊ`ÑrÖÝT`ÄF^êLåR_Xö¯uY±ºGS÷eÊÛr=îj5~è³Ï>k¾iYFßõ¤G5[~AÞËjy¢»Ã5Ìºy1+(+fKYuJ¥RÆÉ¿]=¬Éø/©Ûec¼!UÒ¢ÍêàOè1/Û·o7ï%¯sãÕØá§¹Uw¬ê~Vùîvõ»¬:3ºh.«î@Õ×zgÆ+«Ç1«oïü-«ðtë´n¾6¸¼ÐùÆ-èJÓGg¼UÖn×ÕÐÒÒ¢ÇGÉ¯Ìã,çîoKz-Ç*ßc^Fw?»×Ô­ÁBß»%·ì¾ñ²²bVUè»--ä½î½7ö¾Ýhì~6	cs¢¿ëÆ<¼w»«eæ=ÓnÎñÎìc)«ªtÓ´²Ûí¾ïgu?@WOÂg9¾TÊªCáP^^&GIæ¤Æ¬Æ¶~Õü~Í¼¼²1HÐ±ºÉWÝÕi²êÉg\¼@Ññº¾·UÇã÷ëñÈ^Kê;OeÕBë1,g ¬ ¬-e½7¶T,­r:Û¶m³¼ÓQ2±¬MÑ3 Zcü_3%÷ÆÎ;!UÖÿæYÊêNXr¯t¿¦ùT2¤ÉÝHNNÔûYsÊ«yºÔØm.«¾eäö¥:#/5|/«æ~Ê0oÆÜ°a~U~ºqÖ*YùÆþ]÷MïÕSøºoº7¶»¬Ê<fÍÉÉ¹wÿzzE£¬ú¾ÛñÖáxcn3ÝMnþ5éÎTïU¶üäé!¿Ýb9æËã©å÷h~1eeÅ<¢%ÓÒÒ&;$ªyóùòÂ8u­åX-¥û:Ç;èæÞØÆÒ;vè8Øò-RV	 ¥ÑzñÅuÝßiì¶Pi²ôm3J¼roÝß£b¾oÆ!BúbEâæ5 ¯6¼ì	Öb¾ä¼,0®Ê/T^uéFò	ì^Yé¥±»×òZÐrF~=»¡ùM>OZ	PVà!æ÷Ø¸Ó£gõ,æoÑÊº³S­2²·ø¥©îï0ÇÒÅÆÆîÝ»wRß®ÈË·===v»Ýý¬Îzh±ûi¼«¿ºyóæ	oÁËY0Êe²Êe²@Ye²@Ye²@Y ¬²@Y ¬ôÆfë­·ÌszzzÒÇäÜwîÜ¹ªª*YòÐ¡C^nª¿¿?<<|Ó¦Mycjkkå[öìÙc^¦««+sqËÂååÛ;¦ìvpp°ÜÞÞ^ó|¯ïweÆUSScdÒËòÃÃÃ:êãO[KKK3uÇæeFFFFGGuZ&d5kÖ|£rÿeúå_oßù~PV`)µ%ÑÑÑ=#~'OË/º£e5ßÊÊò2qp»÷²êÝzY¿(+0®ÎÎÎhhhtttx³N¥¬1ë¶mÛÆ[²²²RÆÐñññEEEÎ+W®x)kppðtÕ;²Ï$ryìØ±Û·oËÄõë×-eIcl:v8WÝ©[½ÄLn¶¬¬Ì<§ººº¿¿ß²XSS±EZÚ£ó¯^½*óãââÜÃyæÌÎÎÎöRVßw¾úxPV`»wï65%%ÅÌjl7Ôµ®®.wL¹&$$è2±eËã%cæÛ×¡¤ÇRfee¹©Y¿(+0­êÈõÞýct+ëÔ·ku,(RÓ/ËGEE­ZµÊ8(×Ë7ºóÒ¥KAAA+W®ìMËÕÇ;²ÞtuuI9åÒ<0Æý¬rS+-«LËY&<¨	·,ùì³Ïº»yc²9é½÷NÌêû@YqI6:;;ïÜ¹#EEEÓ^Öþþ~,'³N¶¬feÚívy@YÊÌ¿­ººÚ2¨Ëúúz÷oiiiù¾9Á(´Ü¸L'%%YÆ©îÃM÷±uuuãS­6l®²NxPVÀ³7oz?<<¼gÏÉh=. ûbO>íýöõ¨K.s¤©Ò×üü|)VVV|599y``À¶ÜÜÚod¦_j<Ü;PV(+ ¬PV(+ ¬PVo=À,ðÄO|óÍ7vYßxãÕ«Ww0üøÇ?öø®îG¬¬?ÿùÏÿÀ,ðì³ÏRV(+ePVÊ ¬Êe¥¬ÊJY ¬PVÊ ¬@Y)+²(+ePVÊÀ(kmm­Íf£¬Ê:FFF²^róÒK/QVeõUAAÁÑ£Gµ¬wïÞµy²víZ~Ê:±ë×¯§¤¤cV×^1+²ú$++«¾¾þîûYuê,[)+²N[b³(+ePVÎeåw	 ¬@Y)+ÊJY²(+e²RVe¥¬¯¾ûî»Ó§O9räìÙ³ýýý²ü÷É'DDDäääìß¿?%%eÕªUÝÝÝ²üñÕW_É¥1G»fÍÊJYþxûí·óóóÍsnÜ¸!­u¹²&mûöí'N°Ìyä6SVÀ¬ YáÌsþñ8ÎGîPVÀ¬àr¹.]ºk×®7nhVeÀê>¥¬à«ÎÎÎ_|1,,L*£Õ>øàQ|0»ô÷÷?o¶¡¬PVÊ ¬@Y)+ÊJY²@Y ¬@Y)+²RV(+ePVÊ ¬Êe¥¬ÊJY ¬PVÊ ¬@Y)+²(+ePVÊe²RVe¥¬ÊJY ¬@Y)+²>dííí©©©#11±¡¡²(ëHP«ªªdBîedd$ePÖéQSS$ÿå&"""##ß%²úddd$44Ôf³ÉÕ·Ü¤¤¤PVeÚÚÚÅ³5@Y§Ãá ¬Ê:%ñññííí2ÑØØNYuJe´ºzõêÞÞ^Ê ¬)@Y)+ÊJY²@Y ¬@Y)+²RV(+ePVÊ ¬Êe¥¬ÊJY ¬PVÊ ¬@Y)+ÊJY²@Y ¬@Y)+²RV(+ePVÊ ¬Êe¥¬ÊJY ¬PVÊ ¬@Y)+²(+ePVÊe²RVe¥¬ÊJY ¬@Y)+²RV(+²(«gååå6mdd²(ë45:::66ÖãFGG'uSÉÉÉ#))©¹¹²æcY÷îÝ¼÷îlââbËæççû~SñññzÿÊÊÊd¢ÄÍóÏ?Áï0gË*ùLMMö­¾Rë»wï~ßh×®]Ëï0ËºzõêéÍjSSÓÖ­[Ùwe½sçmLEEekðÑ£Ge~GGÇdos``@napp²æãµ²²rxxØ~UUµ»»R·ÖÓÓ×ÛÛË±ÁyZÖñ	Öwãô÷÷ûþuuuééé¼ë0ïÊZ[[5Æ²øwÞÑ¡ª¥¥Mö<6ÊGeðÈ¦É3EæoY9fxÛ¶m@YýwóæÍøøø3gÎìÝ»WÊÚÚÚJYuJFFFd¨*Y­¯¯ç¼ÁÊÊgÝððÊÚÔÔ´nql°ÒéÒÒR¼Ê4ePV_·ýZ>ÐFRzìØ1Æ¬Ê:mSVe¥¬Ì²JMwìØá^Vç¦¬Ê:qªLéL:MYuJeek0²RV(+²(«ÿeÔ'SVÀ¼.«~ø¹blLËUÃAYu>ü­ÁÊÊgÝ@YeµÙlëÖ­£¬Ê:me-((0Ï¹sçePVÔÕÕm£IYW®|úéÓ§eüHÊ ¬>lþÌóÒÒÒÈÈÈ¬¬,¶(ëT·ëÉ]¼x±»»[&z)+²Ni«Ðé¾råePV¶	KG###õìKFÇÕ«W)+²úäöíÛåååáááMMM:çÜ¹sÔ¶¶6îííÝ³g%)+²zÓ××ªg7lii|Þ¼yS:zêÔ)ób###YÕWRÓºººÚÚZiªËå!¬LtwwKP%½2]XXHYu.UWWkYuªÄUçKYÛÚÚdb÷îÝ@Y'¦»Qe¨j.«:ú´,ó:DYurÛõ]7ÇlÚ´I¦ÃÃÃy×²òY7PVæXYÛÚÚjkkÃªªª(+²NÏÙeÂ¨,ePV>NNjÚÐÐ W¯^½ªÇSVe4=#å­53ñ6VÊûeÕÓñïØ±Ãcn³³³)+²újppPÏÁ´aLöEEE«W¯Ö¯ÍÍÍæH§¬Ê:îQKú±çz555U¿TYY)WõN§SLYuÒ¡ë­·tº´´T®°5@YýÑÕÕe~³L/[¶#ÕÿëºuëÌWSRR(+²úãâÅÒë×¯ËEYuÒÎ9#Õ:::äêéÓ§gô4L07ËzóæÍÈÈÈÊÊJ÷OÛºu+g7PÖY¡¯¯/::²(ë½ááa°¾úê«~ßBCCÃ²eËûSVÀ¼.«îamkk3Ï¼sçï·©'ô×«wïÞq³páÂµk×ò»ÌÁ²ÖÕÕ§6¬¨¨"ÉùLæóý=<Ætç>##cæÖÑùóçãããxâ;wò<Ð²ÓUUUzÞ%tNñìKqkpll¬ÍdÁ<o¨¬³óëgÉOñÔ«¬¯¼òüh­êÕ¢¢"¹j·ÛyÞtYwìØ!Ú¶m[EEEPPPpppÅNOOTÊ`!wçàÕ²35nÑÈ¤¤¤G¥¬,X´he&e<Ð²^¼xQ:ÚÐÐ joow/ë´@xæÊ*CmË511²ëêêtH*s ¬öÜaé«µ¤»]ÃÃÃyÞDYûúúº»»ÄÎ²þûþUCPPOÀ³¯FY£££®ÒªGî¼Á¯¿þºÜíóçÏó<²êÉÝ¼yS¦N§utt´  @frF~eõ5>>^§õÓå(+²NéJ999|²>(+`U©¹¹¹ÆÕÖÖVSWWGYuÒzzz¤£MMMæÐNöÃm(+²þÏççççåääè	ËÊÊôÃÏ£££3g)+²kttTÂY^^nUå¦åòÕW_5__~ùeÊ ¬¾îaqª1îÜ9ã]­ÆÌcÇQVeõ§¬±±±2144dÌüðÃ)+²úZÖøøø­Ê¥Î28~ü8ePVÆ¬ÑÎÎNnllÔ@Yý/«1f=z²(ëÊj^àðáÃ@YýÙÏZUUåþ¶PV?ßÏZTT¤£¢¢JJJvìØayePÖqIA%W®«wîÜ1õÔ©SæmÅ@Yù¬(+zYm6Û¾ûÌ§ý60_Êª§3<tè1§­­Íò¹r@Y'÷Æ°ÛíéééúÕ«Wçååé[q(+²Nâ7¥¥¥Ø6¸wó(«¯ÊËË½ª¶12NÍ¹Ï|þGº¬ûöíËÈÈ8þ<O ÀU¬6mÒ<×²æçç[öÀeåWl&v»½³³§`FÊ*¥éèèË½*á©ªª2­2Q\,óoß¾ý(õ³Ï>;¬5=tè¸p!O#Àôµ§§'&&Æ¼'UGuuuur©/­Zµª¶¶V®^½zõQ,«45  À<çõ×_Ã°0#cVË1J^ÊÚÕÕõ(uÁ-²ÌÔ·ñLç×­[·Xfª¬MMM»wïAÞ;ï¼#s¢¢¢öîÝºoß¾Gwkp`` Ýn7ÏÑíÃYç³Ë/§¥¥EDD8ÎÖ	@Yg¤¬rÙÒÒ"ÕÕÕsfÌª/=÷ÜsÆÃÓh>U.]ºk×.°~ñÅØ³gÏ²fÊ:#e½~ýºKø9SVñøãËý ÊàU7wË°§Ñ¼uâÄóÏ?ÿ|Å¬²NsY-[&¯åuZÊ^XXØÝÝ]\¼gÏ944ôè¾uß¾òcccå5Ï¡ùlûöíWËÌÖ@Ygä&6¿uxxØxOç`ÂðöóÎÎN§ÓÉ(ëôÜÉ'-§à7Î'Ìù1g|õÕWK,¹|ù²1''''775PÖé/ë;w$¥¯¾úª^«§NÒ/QVÌï¾ûnDD8¶bÅ7n°ZÊ:meIsîÜ9§Ó¹uëÖsc$«7oJY1g?>??_÷¹ºV@Y§yÌ:::j­tôäÉ÷feÌå²öõõ­##Ô)knn®4X¬ ¬Ê:	ÃÃÃÆQK¯ö÷÷SVeõIý¦1Æ§±ÊàÕüù¬¥¥¥ú8@Y'1ZÕkjjê½GYs¶¬®®.)kcc#ePÖ©Uß½ZSSc|æ¹ù¦òòrÊ ¬Ëjqqñè3vNYó¢¬.K"j¹EKYGFF(+²NlhhH:<<,·¸nÝ:ãáÈü-[¶o³¹¹Ùét:ÄÄÄ/ÎÎ²þþ÷¿ÿÉO~Â§rþ=CûYeTj>ÓTdggëgãJªeâM7)))qqqû+VØþ·;vìÌcO?ýô<]"##5Ò2ÿröÀÉ0ZkºråJ¹£WÓóÓéÕeu8§gÃÖàEIGÍsd-s¾ûî;6[giYÍgkUe]¸pa@@e¦uÿþý<±²ÎÒ².^¼XÏë$2=ËÇ¬ÒTÆ¬@YguY7oÞ\r=«ÊzöìYé¨¤õjCCîgåYuöUre·ÛNçxçJ|ïºùÙÏ~f96Xî0Ï* ¬³·¬³ÿLÒØØØE­[·ç²@Y)+²RVe¥¬þhmmýøã¯ÂÓ(+e¯¿þ:,,láÂrèr¹xe¥¬þ¸víZ``à+´¦2r¾._¾'PVÊê;wJJÍsª««eä*¡ÊòTÊJY'gýúõiiiæ97nÜ£g~þùçyÂe¥¬³>þøãæ9ÑÑÑ6íË/¿Ôñëc=öÌ3ÏðÊJYríÚ5nÜ¸Q¯þå/¬&$$|ýõ×2rÅxÚe¥¬>©¬¬t8¡¡¡qqq,°Ûí7nÜ0/öñÇó´ÊJYår¹N8±sçÎ_ÿú×t·ºº§PVÊ:i2Za«Ü7cNfff`` ïpÊJYýTRRðøã§¥¥ÉøU²ZYYÉs(+eõ_kk«Ü=­îÜ¹c²RÖé·~ýúØØX¹ü×¿þÅó(+eõßáÃmc,X ~ú)OA ¬Õ2BCÕÊF®@Y)«?Ö¯_oéhkk«Ì,ÏB ¬uÒâãã,X`)eå3Û²RVäææZÆ¬ýë_ÙÕ²úO:ð÷¿ÿ]¦åR¦ÝG±ÊJY¥5µÝgT@Y)«ÿ>ýôS¹·'NàÉ²@Yg±ÄÄÄ¨¨(¹d²NÉÎ;ívûÚµk9²~ýúÈ@Y)«?®]»`§êã´¶¶vww÷÷÷óÔÊJY'á½÷Þ²Ì		ÈÍÍå²RV_½ýöÛO>ù¤yÎþýû_ýõjzvvö¯~õ+ @Y)«O¾úê+Ýö«WoÝº%Vc|4ºXNgKKÏQ ¬Õ'+V¬Ò]­´ÛíË/7/ð£ýHÜ:YX²<_²RVo~ùË_J;m6´ó±Çëéé1¾´qãFiêoû[lDDû~Ye¥¬ãJJJúÓþ¤Ó2<Üþà?èîî6æHßï=²@Y)«OZZZ,YóÑGÉXV¬_|ñygyfýúõ<e²RV_ÉÀôÈ#[¶lùÍo~dùêòåË¥¸<e²RV,Ð|J&=øóÏ?ç)²úãøñãÒ´´4ÅnÜ¸QÏÈó(+eõß_~¹|ùòøøø?þñ<Y²RVe¥¬³Øûï¿ÿÂ/ìÜ¹óÚµk<w²RVÿõôô|ïß³Ùl!!!v»=  ààÁ<²RV?-Y²ÄápüóÿÔ«?ýéO¥²ÆUe¥¬#5NÖ¯dËÑÂ@Y)«?,3cbbzê)¿o³»»ûìÙ³üecÖ?ÿùÏæ9~Y95&$$ä£>â/(+ûYm2uö÷÷¿)é¨ÓéüöÛoõª¯X¯ü1À|)k___tt4Ç[~óÍ7Í|òÉ'Î12ÍÈÈð²÷^°eËÌ²644,[¶LbÌù¿n^íµyò~Ö®]»vçÎ£Ï?/AÕÏÆq¹ùùK.½uëÇIJJº|ù²åÛ%Æü1À¼(kffæÕ«W²Þ½×æÉ<?JvÕªUahVVÖ|àqaùeÇêþýû·mÛÆÌ²þÿc³^róÒK/Íó³Yv¯J;Çe]]p¿üòK½*#Ý%K´´´ðÇó´¬7Ø]\Üwßgóî»ïæçç·üç.qM#¾s¼¬Æ6^Êê£íÛ·geeÃÖË/;NcTê,üÆ¸þ1+eý_nÝº%£ÏU«V8qBF«U¹äÉ²úO|ðÁ¶mÛòóó½V3E(+ePVÊe¥¬ÊJY²@Y ¬@Y)+ÊJY²(+e²@Y)+²RV(+²(+ePVÊe¥¬ÊJY²@Y ¬@Y)+ÊJY²(+e²@Y)+²RV(+²(+ePVÊe¥¬ÊJY²@Y ¬@Y)+ÊJY²(+e²@Y'ÐØØìp8)+²NI||¼Þ¿²²²Ê ¬Ó&88X.7¹ËÈÈàw	 ¬ÐÔÔ´uëVÊ ¬Ó``` ;;pp­ÁÊ:i¶ûôjOOO^^^oo/Ç(ëTÕÕÕ¥§§÷õõñ®eÑÑÑ6Ê ¬)@Y)+ÊJY²@Y ¬@Y)+²RV(+ePVÊ ¬ÊeõËîÝ»¿ÿýïÇO«¸¸¸ØØØxø¼º|òIÖzê©Ö~úiV×¤ÈêÆzðuÉäôÞfHHÈ#_Ö®®®ÿn¿øÅ/¤¬ÿß¼òÊ+ááá¬½ñÆ6õà£ûöÉêzÿý÷Y¾8zô¨¬®7ß|Uá?þXV×ï~÷»é½ÙO?ý´¿¿ÿÑ.ëLøÃþ°råÊðÍ~øÃþõà£ÊÊÊñ>lîduõôô°*|qãÆY]ß|ó«ÂÿùÏduUUU=GY)+e¥¬²RVÊJY)+e¥¬²RVÊJY)+(+e¥¬²RVÊJY)ëd¼ÿþû7näiç£ÒÒÒçõà£.,]ºõà£o¿ýVVËåbUø¢··WVW;«Â£££²ºþö·¿QV=ÊemN§ÃáHLL¼xñ"Oe-%%%Éc½ù¢¶¶Ö8pÕåÅÐÐP^^^PPPlll]]«kB²-[¦ë§¡¡56¾¾¾èèh/ÿígz½Íß²fggÈDaaá¦Mx.z¯gÅ,++KHH`½MhddD^eeuyqàÀCJ0âââX]¼~ýºLÈ¥5æN^sÈëóaùîki¦×m>?GåOZ&Í¯n0àà`ÖÛô®<Í&$Ã+W®ðWé;yý¡ïJK-ÂsyõêUsYÝ×ÒL¯·ù[VÃáq555mÝºõæ$RRRä/Öø«fuyÿ<~ü¸¼bH´¶¶²º&ÔÜÜ,«K]r)¬1om3Õ-Íôz¿eµÛíÆtPPOD/²³³YoÞeeeÕ××ÿªY]ÞÿO>-òÕ5¡ÔÔTåKbõ7¬1_Êê¾fz½Íß².^¼xxxX·È4OÄñôôôäååõöö²Þ|ùc6cuMø7h7°º&å³Æü(«ûZéõ6ËºyóæââbKñDô¨®®.==½¯¯õæß_5«ËíÛ·ËDûªU«X]qªt©­­MÆ¯¬1Ëê¾fz½Íß²644DEEÙív§ÓÙØØÈÑ£èèhË õ6©¿jV·oßÎÊÊ±WJJÊÕ«WY]êìì ÊKfùXV÷µ4Óë3E@Y ¬PV@Y ¬PV@Y ¬PV(+ ¬PVÓ÷gl³¥¦¦f©¨¨(--9.]ò²¼~¶úùí÷îÕ«rU?NeæÚÚÚ¬1Q©]kk«Gb+X]]mÌY¶l¤7gÌ¹sçªªªduëÖs>,snß¾m|KCCÌ1>Do³²²õPV`®2+[ZZä²©©I'FFF´`]ìúõërõæÍr¹zõjiþÌ]ÅîÙ³Ç2G¬7ÆÁ2òäI.,,äwPV`.èííÕ+W®Házzzä²½½½¦¦F&¥¬N§S¦Ú×ÐÐP¡ÊIuÀZRR"sÚÚÚ,£^¹M£âæÛ@Y¹CªÒ×òòò   _JuïíÛ·-e5Æ úYÐºõX¸úyãºÖ¼ð±cÇd¦DW¦5Æ~ø¡ÌÑËÄ3gøE;úúúvìØ!+++Ó4ÊSj'2Êt/«¯¯ÖºQQQ6m	óoØ°A7 ë÷feeY"-·Ão ¬ÀR]]mR¤ûYebëÖ­ZÖ]Rò)_ÕíÆºÔØÉªÛ»ºº,á°¸pÁìÊPÕ8ÜI¼üÊÌ)ÚÈììl_gg§&Pb)ÃV¸~ýº1Õet¿l\Ü¹1zNDFFJ,GGGå»dZ7ÄîcVýÊÌº«µ±±Q.àÄÆÆW].ñ-Õ÷êííÕÃGFFÜóÉ ¬À<ø»µÙvíÚ¥ÉÉÉ2Q^^*á11v ÊWåêæÍ@3ïñG0f(+0/èßþþ~¾xñbBBGe¾U'£OcW«Í¤ãÑÖÖV¹Åt¯X½Ä1+@Y¹ON2stêÔ)ÓÝÝïþ;jÒÒÒ4æ­»·oßÖRS£¬R#yìµ½½ÝKÊàaphhHXWW7áwUVVê0W(ËÇÆÆfddè|)<<Ü½¬¥¥¥zEYÞ2fÝµknß@Yy'!!¡¤¤Ä¸ª§j:pà1g``À2lÕÉÅÅÅLÊe²Êe²@Ye²@Ye²@Ye²@Y ¬À/ÿs3X1÷çIEND®B`


rå'NgxþÒÒÒÁÁAhhhÐCCCrñÕW_õ¼Ùöööe£r½©©©Ù»w¯ülQQÑXw¬ººZ®ÐÝÝ=î²¯Y³fÂ«nûöí~ú©ÿ×¯¬¬Ô	y@åWûÿ9Ïµj¹NJJ¬Ü1È·ä:rù[eÅ÷ôìesäÈùGêt:###.^¼èãú¾çÒÌìÞ½Ûó[¤ìììÎÎÎÌQæUUUòÝºº:ÿÅórþüHcccÎ(Ëÿëý._eEÉ¨¨(Ë>úúõë2Q[[kÜâ/¼.²¬óððð¤¤¤É<¢··×ójòâ@&ÂÂÂ/^¬#ëù±QrQ~o||üX¿¥¯¯O®¿uëVß/,Tss³õêÕ2=<<ìùÄ+ô322|/ùÅÐÑ£GåbWW××7üu²bZuÃAäqkk«ÿe7oËåª%3å6Í×Ôµ---ÿË2óøñãúï[¾eéÐmN¬¬ròÝúúzcÎ+WdNEEçåwÉõ%'òï¾¿¿_.Ñ[9uê.oYY#å«Þí¸¸8?ße<¯eî½®cÎo¾)sdÚkYebåÊ:!#fYÏ2-ù7oWHeyaaþ½29^·1X6°c-Ü Ëåùùù/§,·fÕXvcp/÷[^ßOY¥vº9nçÎÚ|ÄÆÆÊLåø_V0F?2èÐ!Ï¼óÎ;ûí·ÜUMS&PV¿iÓ&Yùí²			2sË-ºÔ¸W:Ì2$ýÖAª®½çNæ·µµé¸Ü³Ê´ñRC~×aeÒ_èËôôtéúX?"K¡KWRR¢¯tLpA_[he-kÛ²å+Î=kÙ´pñâEc¼8ó:äÕñgµQV	³>¬Æ|ydiÊÊiQV.xæMþáfeeIt³JÃÌ½´@e¦ÄÛóôWKÆ"##óòòÌöZÖO?ý4%%Åét&''kÕØñ&Ór½5¯c^Ëík~dÚívKJåbqq±^¹¼¼Üë/²U÷+Ëp»dÉ¯ñ£?eµ8pàÜ¦nGi¯×6ÇÆçÉ×Ë/ËWÅ`½vK¾ust§©¼°ÎÎN)í5999ùò«e¾Þ"÷G§ÇÊ¶QV]Ãzÿ;::ôEÒ+W(+(+¦WYcbbÌÿçË(::Ú/ÿeÔb UV6Rf¾²¥¬:Âö4ÖÝ6~££=¿¯*¤¦ZJ[kt%?ºIÙ<$ÒEùwolÖÛß¼y³«%_ûúúôWX6Òz-eõÑí¾ìÐ~u ~W·åÓgffêÞÊk×®Y~êÄòêAÖþôUÃÒ¬êvWÏk»·¼TïVWWË·),,ô]VytÍÈ#²xñbÙÜÜl¾eeÅ´(«þÆ=ÉÏ²ê?G-zzzdbïÞ½þUÕ[ÐYÊ*ÿë5T£V­ZåYCBB¤2,3F^Ëª»KÓÓÓkL¤^Ã¬û+K_åbii©+õWHn½Þ½åËë´,°É´×!¬ô^ï°±~ô #Ïíº¢®®NêÑ+è±TÏ<ónMQø¼yó6nÜh¼0WZ½½½zãæ!¾'ù®Ä[wc³¿q´Vdèj1ÿ ¾1?â)))Æ´ÜOÏ²ê´#,×¡¬ ¬ie5Yu¯n[Vó6XcÖÐÐP¹h8ÚÝÝí»¬ý©zÆ8Ï³¬Ã³gÏÇUk×®ÕGñÜ¶i~õ ¯d`-[²dôFz²»5X²$+D_+¨1u(<22¢ëYW¦qeym§:Öõ GoyÝ~sô Üàà`yñ¡¿B~n'ØØØó0·¡¡!11Qk'_õ`4yùb, .TGGå·ÈXÝ|ì´eÓVV×²ê=ë+YÏÚlÊÊéRV¸ûºÆ2Ve¦î«¬Z>íâÕëHÅÏ1«ñÖÁîÍowß©+£.=rµ  À½ÿþûÕ%eÁu¬¦Îjt²æêÿË*¤¦¦·ÄJ$`Æù®ñVZ!÷A÷ K)õ0`¹(ÆÕäädËHWï°Ëå2ÆÐÒ'NèòæèÎWcß$ÜòÞË»l½¾ææ·´µÜ2Ê÷³¬º8º£ATÇ»rÿµÐ:ø6o«(+¾·²ê0Ë[ìO>iÁ$ÿmõæëh5%	Æubbb,eÕÿøó,«çÖ`§¥¬aaar±§§Ç`ù_VÏÃô¥ùGèÖlóûRnzìö:f5VÞ7¯o>	tÌjütmhÃcqe°h9kn*0b£W^êLå«¾°ì_7ë²bu¦îË7¶åzÝÕjüÒÇÌ|1;;Ûr×Õly|ÕòEwkuó>cVPVL²ê<J¥42»zXñ_R·+Ê(ÇxCª¤EÕÁÐc^Ö®]kÞK:VçÆ*«±ÃO8ò,«îXÕý¬ò-Ýí:á²ê`Îè¢¹¬ºU_455é«¬^Ç¬B~|¬ócZVáéÖiÝ|m,¸¼ÐùÆ-èJÓ¥3ÞÏ*ëJ·ëê"´¶¶ê±ÁÆQfòy=±åÜc­pI¯åXeóÛcÌ×ÑÝÏ5õgk°Ð÷nÉ-n< ¬ ¬eúnKù_¯ûGo¾ïB7MÂØ(Íïº1o~»ÝÕ2ó¦i7çXgö±UUºiZ9ÿ÷³z «'á³_*eÕ¡phh¨D¨  @¾ê&GIf@cVãÛ	Õü~Í¼¼²1H® cu¯»«'!26ÒeÕ-Ï¸¸8y¢ãuo«Ç:î×ë½^¯©ïö?u+«ZÏa9eeÅt)ëÍÑ½¤RiËå***²¼ÓQ2±¬M®£g@´Æø¿fJnwBª¬ÿÍ³Õ°ä^é~Mó©eH-»ÐûYsÊ«YFÝjlÍ6U_Èuäö¥:#/5ü/«æ~Ê0oÆr¥~W~»qÖ*YùÆþ]÷MïÕSøznº7¶¬Ê<fÍÏÏ¿ùí)ôôFYõ·c­Ã±ÆÜfºÜü0éÎTßU¶<@òôE·XùòzªgyÍï0¦¬ ¬°Ý,èHªæcÌçÏãÔµcm´oèë £K×­[§ã`ËHY%TznTTFkÅzÝßiì¶P)Pú¶%C^¹·ïQ1ß7ã!±"qóW>öëoÁ+y$/òÊ«.ÝH>n=++½4v÷Z^ZÎÈ¯g74¿ÉÇëI+Ê|Ï£yã=6ôèY=KùGt§²îìa«ì-#~iªçF¿Å1t±±±7nèÇu|pÛÝÝíp8<Ïê¬fÙÏ»êõ»yyyãÞ³`ÊeÊe²Êe²Êe²@Ye²@YèLPÐ¦MÌsº»»³FåëèÑ£uuurÍ·ß~ÛÇMõ÷÷GDD¬Zµª`Tcc£üHIIù:Ù£[Æ´~Ü¸cÊápÈýééé1Ï·h©ý¹«(+0¦s½$æ²ÖÔÔX®?44¤iii~þë×¯Ë­¥§§sµnÝ:óuGFFtZ&äc%ßè¥Ü~ægÆºB ü¹«(+0&£³èèhiQûöÉ×ãÇþàÊj¾¹ãc¸¬ãà¶¶6ßeÕ»<µeõÿ®ÀÿqñâÅeËÉDSS$¤££Ã÷u2eµÆºfmm­¡ãããöîÝ+áloo÷QÖ[:fõzWPV`Ì¢tK¾îÜ¹óÚµk2qùòeKYÓFN§ÄUwêd1­®®6Ï©¯¯ïïï·¥¥ÅØ"-qÑù.ùqqqá<räLçææú(k@;_ý¼«(+àËÌAMMMõ³Û³ºÝîÕ£<èp0!!A¯)ÆJGÍ·¯CI¯¥ÌÉÉéííüÕÿ»²ãÑª~LneüÖ`í±FeBêãúQQQK,16Äòáüâ//^,Ýü5Ð»²ÞuvvJKÊÊÊä«y`:ûYå¦¤+!²LlÙ²En¹æc=æùãæ-´æ4nß¾]¦7nÜ8ù1k we¼fxñÆ2±wïÞ)/k¿ÆOFã-«ÔN¹C^L¾¬ÝUð^²úúzËtRRØN8áù#­­­2ß|êqZnþ4[Ýn÷XáÔaëÊ+'_Öî*ÊX]¹rÅëü¡¡¡Ih½^A÷Åû¾=ìè/¾0æH¨$Z¥¥¥ÌùnJJÊÀÀ¹m«W¯öùFn	ÜUÊe²Êe²~Þyç¨¨¨h¦|ð/ùËí]ÖßýîwL>ú¨×·ßfeòÉ'ÿÓÀc=FY ¬@Y)+²RV(+²(+e²@Y)+²RVe¥¬PVÊ ¬@Y)+·uYÛÚÚÒÒÒNgRRRSSePÖI ÖÕÕÉÜËÈÈH°xúé§)+²¦¡¡!99ùo¾	òféÒ¥<Êêááá°°0ÉguuµõðÛßþ1+²¦±±qÞ¼yìgPÖ)ãt:)+²NJ|||[[L477geeQVeS§N%%%Éh5##£§§²(+gPVÊe²RVe¥¬PV(+ePVÊ ¬ÊJY²(+e²@Y)+²RV(+²(+ePVÊe²RVe¥¬PV(+ePVÊ ¬ÊJY²(+e²@Y)+²RV(+²(+ePVÊe¥¬ÊJY²@Y ¬@Y)+²RV(+ePVÊ ¬Êe¥¬ÊJY ¬PVÊ ¬@Y)+²(+e[8sæÌ=Þï½O>ù²RVÀ¤ìØ±cîÜ¹k×®---ÉÏÏïïï§¬02HÊU/^½zuÑ¢E2x¥¬0EEEöì1Ï9vìXff&e¥¬X±b¤Ô<çÜ¹s.¤¬0%%%ÅÅÅæ92ÜRVÊ¡Î?_jªG-Éø5..ÎívSVÊ ÖÖÖôôté«kÔÇ<ã²n¹ÞÞÞ¯¿þÚ&KY ¬@Y)+²RV(+²(+e²@Y)+²RVe¥¬PVÊ ¬@Y¿ÍÍÍ)))N§399ùÔ©S@Y'%>>^ï_uuuBBePÖ)¢µHLLl%² ¥¥eÍ52±ÔÃ<@Y5¹¹¹×¯_gk0²NVwwwAAAOOÇ(ëd¹Ýî¬¬¬¾¾>Þu ¬S :::È²(+gPVÊe²RVe¥¬PV(+ePVÊ ¬ÊJY²(+e²@Y)+²RV(+²(+ePVÊe`¦õÐ¡CAAAÃÃÃ@Y§ ¬ÑÑÑ±±±^¿522BY507n		Ù°aC®IMMMEEeKKK)+²@òÆ~Ve²²fddPVe²Þ¸q#hTMMekð;ï¼#ó;::(+² ¶¶vhhÈs~]]µ««²(ë¤è!Áúnþþ~Ê ¬ãkllÌeÙüæoêPU¾¦§§³@Yý2888îM@Y§µ¨¨²°«W¯¾õÖ[ºIo×®]ýýý¬Ê:qW®?räÈÆ¥¬§O¦¬l¥««káÂÏ=÷Ü±cÇ<Èj¡¬2<<,CUÉê'x?+»ÉÏÏÿÆE°ÆÅÅüñÇ¬ÊÊgÝÀD¸¯¿þÚ<gÇÅÅÅ¬Êê¯å£cNWUUÉàU¦)+?~oo¯yÎ=Ö®]Ë¡¬lûµ| ¤tçÎYØSffæ¶mÛÌs-Z$qeÍPÖILYØÖÿügËõÚk¯9sæäÉ999Ë-³bAY)+àüùó«W¯NNNNMM-))!«u"ç2®[·Î³¬^Ï'LY5qªLëL:MYuRe½½¶ïß¿ÿ¹çËÉÉyíµ×,ÇÊ@Y#A]´hÑáÃ;&¹xñ"OeàhU²zõêUcNqqqnn.Oe¤<xÐ<§««kîÜ¹<QÓ·¬ÓùÏexzìØ1ó¹·ááá<QßOYõÃÏõàÔÔTãx`cº¢¢B.:ÎéYÖ7ÞxC­æ9öìY²d	OÀ÷SÖq?ü|o¾zõêÂ¾þúk­îß¿_¬n·'àû)ëø¬®®®üü|Ë%MÑ*YPVfhY/_NYuÊÊºuëVó7nPVeõÛí^9J®®®².^¼Øøôòòr#¿²(«_?ó¼ªª*222''­ÁÊ:Ù­ÁúArÇïêêÊ ¬ÚÃ*tº¡¡A¦ÛÛÛ)+²Nd°t422RÏ¾T[[kÄÕét^¸p²(«_®]»vèÐ¡sôèQ	êÙ³geº§§§¤¤D.Ë5)+²úÒ××¦g7lmm|^¹rE:zàÀóÕ³(«¿¤¦n·»±±QÚÛÛ+CXèêê Jzeº¬¬²(k.Õ××kYuªÄUçKYÏ=+6l ¬Ê:>Ý*CUsYUyy¹,óß~ûmÊ ¬mÖwÝäZµjLGDDð®eå³n ¬Ì°²=¶±±Ñ8¬©®®²(ëÔÝP&ÊRV·ÔáÃW¬XQRRrþüyVfHYÝn·Ô´©©I/^¸pA¦¬n©7ÞxÃåríÙ³çO>)..?þÉ'Y-¸íËªg°¼µæV¼²0ûòË/çÎæÌcÎï½·páBÖnï²êéø×­[ç5·¹¹¹À-²k×®µk×çô÷÷Kk¯^½ÊÊÁíZÖë×¯ë9VÊýVMMÍÞ½322tçë©S§ÌNYLUY-3çÏOYqUªÙÑÑ¡®ÓÒÒô[µµµrQÏ ár¹¤ÁÀÔúòË/¥£]]]Æ²5·wY=C»iÓ&®ªªlpë¬]»611ñØ±cçÎï½÷ÂÃÃ?ûì3VfHY;;;Ío¶iyºs[mÇéééò'771£Ê*)]¾|¹ùbjj*ePÖ8~ü¸¤ôòåËæ²æääPVeØ#G¤£zSGG//¿¥§a¢¬YÖ+W®DFFÖÖÖz~¢Ü5k8»!²NÑÑÑ@YoÉõ^ð-455%&&'÷§¬[U÷°=Ö<óÆþßBvv¶Ð_/~óÍ7¡fÍµtéRKÀ,«Ûí6NmXSS#E	óÍ0çÿxéJO<ñÄ²eËx,3°¬ÃÃÃÆt]]wI<û[6-«åìüúYr<µ!eØ½¬ëÖ­ÕÔÔÔé¬¬,Ê ¬°ìLe4299²(k§6655IÛÚÚ<Ë:å'¦¬[V·Û­CR ¬Ê:©²öõõuuu¥¬ÌäßBNNNbbbffæ=X! ¬^²FGG'$$´Ë-£¬Ý>|xþüùû÷ï?wîÜÇ,¯¶·mÛÆjeøä®"Ó.KÊ:22²uëVYVVFY[éïï;wîgfÌ9þ|xx¸|eå²N5>>^§õÓå(+`+­­­ÉÉÉ999555¬PÖ	ç!??OìÔÔTËL=Ý)+u:¢¬À47ÖÖàsçÎ±r@Yý¤®^½Ú¸xúôiãv»)+`[¸G0%&&r(«¿º»»¥£---æÐúá6yäåõ+.¹k×.V(«_s^ZZ¯'8¬®®Ö?6>BÎ<¥¬Ê:¦	ç¡C¡ªÜ´|áÌã×gy²(«¿XejL=zÔxW«1sçÎ@Y'RÖØØØ¾¾>4fîÞ½²(«¿eÏÏÏ×Ñª|ÕQQQÆÞÿÊ ¬³jD/^¼(ÓÍÍÍ:sëÖ­@Y'^V1<<lÌ|çw(+²Nª¬æ+lß¾²(ëDö³ÖÕÕy¾-cuïgÝ»w¯nª¬¬nåM8@YÇ$p¶··ëÅ7n¿àÀó¶bÊ ¬|ÖÛ½¬AAA¯¾úªyñ¿Ó²ìRV=áÛo¿mÌ9ö¬åså(+²öÆp8YYYú+NSSePÖÞxSUU¥]¹r¥gw9o0²úëÐ¡CÆ^Õ Q2NÍÿù4ý@YÇ°®ZµJ?ðZZZj¹ÎeÌØ²JJ;::äkee¥^t8uuuÆ°U&***dþµk×(+²úÒÝÝcÞª[Ýn·|ÕÃ,YÒØØ(/@YÕßë¸eíìì¤¬ÊêoY[ZZ:::6lØòæoÊ¨¨¨7½úê«lPÖË*_[[[åk=cVe²^¾|Y¾ÖÔÔPVeTYuZÊQVVÖÕÕUQQQRR"3)+²vNßÀ:44d¼'²(köíÛg9¿q>aÎ ¬»qã¤ô^Ð×¯_ÐoQVeõËððpÚ¨£Gº5kÖ%YÍËËÊÊJßJYÕß³C+Ý·oßÍ[²frYûúú2FÉuëÖ­RÖÕ«WëIuÀºyófÊ ¬2ZÊÎÎ¶|·¿¿²(«_N8±jñi¬2x5>kUU~ePÖF«:`MKK»yëQVÀ-«¡³³SÊÚÜÜLYu²eÕw¯644yn>éÐ¡C@YËjEEÅÈ(s+>í²lQÖÞÞ^¨å-e¦¬Ê:¾ÁÁA)èÐÐÜâòåË#7oÞ,óePÖÎqh>G0(kÐÍÛePVÊ ¬Êe¥¬ÊJY ¬PVÊ ¬¨«W¯~òÉ'555çÎcm²rìØ±ðððÌÌÌçN&ûûûY-e¥¬ÀD?~îÜ¹2ZÕ]]]-Ú¶mk ¬hQQyÎ_~¹páBÖ@Yg~Y#""îu×]wñ¬ÂTY»víþýû-3çÌÃ(ë/«5ÈD.òÄÂTYÍsN<É ¬3¼¬UÃ¡eB.Þyç<·0yìg(«Ëê9HÕÖòÜÂ°¼víZ(ëÌ/«1`¥¬¸®^½*=|øð3gXeeÌü­­­zÄï;ºººX!e¥¬VÚQcÇªL°c9xðàÜ¹sKKK÷ïßwþüyV@Y)«÷¸rl0|Î?ÿäÉÆ·Þz+335PVÊêÅîÝ»ïõì³Ïò¬Wååå«W¯6Ïéíí!¬|eå²Û±cGii©efLL[ÊJY8vìØÂÍ#T·Ûír¹X3e¥¬À=5Jß<£¡-//gµ²Ôßß_ZZ*AMLL$«e¥¬Üæe=uêËår:IIIÇ§¬Ê:)¹¹¹2QVV¶jÕ*8æ!;;ûÑGýi %%eZ522rddD&¢££¿ùæ ¦±ððði]V§Óiò°fÍ¶ØìÃaL³@Y'eÞ¼y2*Õ¡ªLSVe¼¼¼¯¹¹¹@Y'¥©©)**Êáp¸ææfÊ ¬)@Y)+ÊJYáîîîwßw÷îÝ2ÁÚ@Y)+&å'ºsL¼ôÒKãþÈG|Ç¨|u²RVü¯W^yEjº~ýz½+eðê;«rijZZdU¦ke¥¬øÇÜ<'&&æÞïõñ#2´5§ôøÄ599yÜßuøðaIøûï¿ÿõ×_³æPVÌLÉç¼ôÒKN§ÓÇHG¥¦æ9aaa¾¤½½ý¾ûî5kÖøC¹²LTVV²òPVÌ@³gÏÎËË3ÏyäGÂÃÃõôéÓæ9K?"7(MíííÕ«W¯¾ã;¾úê+Ö?ÊÛ:£££çÏÿøã¬¯"÷ùçëÅÚÚZ	gnn®ëÿö·¿Éø8I2,#ãK.gFEEò` ¬îdø(åKJJZ¼x±Ãáp:ãÆõî4Jåge"!!Á÷õeÄ)W.))çü:¹(ëúàôôô¥Kòx ¬Ö~úióT:öìØØØqðõ×_?üðÃï¾û®?¿¨¨¨H*æÌcÙ8lÑÞÞ.W¯æÒZãd ¬¦$W¥^Óá-1íkoo¯X%ù!²bºáã/¾hsðàA/~ïwLjúÐCÉ=Ñï¾ûîúúz/Ó]tt´å­¨?üð]wÝ5MîY÷ìÙsòäIã a ¬Ö>ÿüs=IZÊËË[¶laÍ ¬ÿt:Î;ï¼Sþ^´÷îY³fËò^U ¬ÕÖBBBÇúõë<¸`ÁqÏèk¸téRKK+e¥¬ø/ùIII¾O"²bL÷ßÿÃ?lÊ°5²RVL°¬2H5Ïùë_ÿJYPVÊ	zñÅï¸ã©©1gÁ!!!¬²â?._¾nµûÐCû#÷ÜsÄõ'xé¥î½÷^þðÃY(+eÅÌ5KÒ¸mÛ6þÕ¯~%qàÆý©§z*,,löìÙ÷ß¿åT²RVûÊÍÍÏJÿúë¯ËÞóüÀ2S°/¿ü2+(+ö£ýhÎ92f-))aåeEÀ¶mÛ&ýõ×9?ùÉOdÎåËY9@Yñ¿¤O=õ|Õ3àûöÐCIJðÄÇÇ;N~þùçY@Yñ---C?pôÎQµµµþxîÜ¹sæÌ¹ï¾û8Ð(+þK¡¡¡ÆPuÁRYF®ÊzûuÃÒ9=!L¸Ýî©½ýTóÙ[×¯_Ï(ëL+ëÏþs£©F_§öWlÙ²eÖ¬YaaaK.åIu¦ÕR°je§ðW´´´XNòÐÝÝ-¿Â|è/²Î²Ê°Õ<GG®Sû[î½÷^§Ó©q½téÒ=÷Ü#ÙÏufÕ²cõVU"z×]wÉÍÎ5K¾Î=ûóÏ?çufÕ²íwÊ·¤¦¯¼òÊÿøGë@YglYçÌclÍôÁðtÊJY'We·«Wííí¿þõ¯xâÃóÜÊJY'î«¯¾5kÖ÷Ý÷üóÏÿìg?ñnaa!O_ ¬u¢¢¢yäãbee¥Äõ³Ï>ã²¬··÷Î;ï¼téyæC=Äéò²RÖhoow8éééÙÙÙ<²RÖ²îØ±Ã<~ã7xe¥¬!Ë(q]¿~½4µ¾¾>***""B¦ye¥¬TRR¢ïÕq:<òe·+²RVe¥¬PV(+ePVÊ ¬ÊJY²(+e²@Y)+²RV(+²(+ePVÊe¥¬ÊJY²@Y ¬@Y)+ÊJY²(+e²@Y)+²RV(+v/k___tt4ePÖ)ÐÔÔDYudgg_¸pÁUâââ-[Æc	 ¬þ¢¬Êz«ÊÊÖ`e¸£²(+cVe¥¬Øª¬)@Y)+²RV(+ePVÊ ¬Êe¥¬ÊJY ¬PVÊ ¬@Y)+v,ëî¾ûîø)oK,·+YvYöÎË3ßËnç?ùÿøÇüÉOímÞöeíììü©ö_üBþÌþÇöîÝkÃeß·o,ûúõëíùÐK]|òI.û/ùË|ÐË^RR"Oû>øÀËþáÊ²¿üòËS³ôQÿí]Ö[á÷¿ÿýâÅí¹ìòTûÇ?þaÃeeohh°çC¿dÉW^yÅË¾iÓ¦þô§ö·Û-Oû¿ÿýï6ÿûß²ìuuußÍ¯£¬²RVÊJY)+e¥¬²RVÊJY)+e¥¬²RVÊJY)+e¥¬²RVÊJYñî»ï>ûì³ö¦¦¦þóÿ´á²ÿë_ÿe?qâ=úßüæ7;vì°ç²ïÚµ+''ÇËþÕW_ÉÓ~``ÀË>22"Ëþ§?ý²pû¡¬PV(ëtsêÔ)Ëåt:?n«eïéé	2±Ï÷õõEGGÛó9`Yvû<SRRäQNNNGÜV»ç²Ûêo¿­­---Mè¦¦¦ïò¡·oYsss+++e¢¬¬lÕªU¶Zö»=âò§hþobçç²Ûç9¯gv­®®NHH°Õãî¹ì¶úÛ|êÁÀ²"##¿ËÞ¾e=22"CCCæ×òv ZßÙÑçÓGvvöÌu±ÏsÀsÙíù		±íß¾.»=÷µ½Ëêt:½NÛäU|VV,ujjjGG­Ý»=ÌËnÃç@KKË5kìù·o,»Ý÷ááá°°0yæË¨ý»|èí[VÃaLÛs%twwëK9ÕnÏ¯ûÕlòÈÍÍ½~ýºwó²Ûóo¿±±qÞ¼yßåCoß²ÊÒÍºÒíÉn¯*Ìu±Ûs`¬#Vfüs@*RPPÐÓÓcÃÇÝ²ì¶ýÛ×êwöÐÛ·¬yyy2!_å­=>>¾³³Sÿê²²²l[V»=,[mòp»Ý²6|Ü=ÝVû²°mmm7GÖýÎzûµ©©)**Êáp¸Yï¶ZöÄÄDy.`¶-«Ýæe·Ïs ::Úò>û<îËn«¿ýS§N%%%ÉÂfddè¨ý;è9SÊeÊeÊe²Êe0uÆAAiii¹£jjjªªªdÎ_|áãúK,ñ###rµ7êÅë×¯ËÅòòrÖ6@Y¦±±1gDTjwúôiùªe®`½1'11QÒ?êèÑ£uuuråËs¶oß.s®]»füHSSÌ1>Do³¶¶õPV`¦4+[[[åkKKNk%ÀzµË/ËÅ+W®È×iþÌNÅXæHµâÆ88;;[¦÷íÛ'Óeee<efý¤IÑÞÞ.ëîî¯mmm2ÑÛÛk)«Ëå©ö5,,LF¨2!CÒ¤¤$°VVVÊ³gÏZF½rFÅÌ·	²3U¥¯Öñ¥TP·ñ^»vÍRVcª­[uáÂßî¦5_yçÎ2S¢+ÓãÝ»wË-±L9r ¬ÀÌÑ××·nÝ:)µ¦QFR;Q¦gYåâ'¢££u£î-[dfGGGTTÔªU«dÂ|ãCCC+W®ÔÈú³999HËíð(QêëëCt?kYYL¬Y³FËÚÐÐ ×|Êwu»±î"5v²êvàÎÎNK8e,_?ýôSc°+CUãp'òòQ´¹¹¹¿/j%2lË/cP½î;:JQÒÈÈHåÈÈüLëõ±çU¿²3îjmnn¯÷î5·uëÖØØXãboo¯ñ#ÕÏêééÑÃ=óÉ ¬þnu"%%E&:&á1®cì@ïÊÅ¼¼<#æLÆÇÇýYÊØnøíïïéãÇ'$$èxTæKYµy2ú4vµêÑL:=ú´|«é&_°ú7cV²3d>æèÀ2§««ëæ·ï¨IOO×·î^»vMgJM²Jqeä9Ð1k[[*ÀmÃ8áàà 1))ÉívûSµµµ:Ì"Êõccc-[¦ßêííoEDDxµªªJO (×·Yõt<"el'!!¡²²Ò¸¨§jÚ¼y³1g``À2lÕÉLÊe²Êe²@Ye²@Ye²@Ye²@Y ¬`Bþ?ÎÈÀ¶/¾ÚÏIEND®B`


总机械通气时间d


正态 Q-Q 图


ÿõ¹¬[·.øß¢ßL9räÜðÿc+**X-dEJ%·Û­V7A!`²jøÒ.++øâRk×.iÈZ[¿½sçüè>Ð¯%³¯ò¾úê+yËåò¯BTàé#déÒ¥v»½y,çwô/¥Vèúµ¿,<yò¤	ù!ÕþòSyÍðU^S~zìØ1mô³,Ù³gOcðd.ÒïP»zÃÚµY#íÉ*×^M5¤bPV»¾âÏd(õ¿÷Ýwß%«CI[+a§ú[ä¥.GÿÜõÉªýíZq/ï7l$+¦Ag²Z¿~ý¦MÔðí·ßVíÛ·ë,k|Y.!¤bOmï]ý*I­aÕK©Ç8qâIÚÚVVsÒØ²e¡zÐvËòU«VÉcÔ~Kõ4Òîíí5¬sssCßÐj,U1ûGÎG$·Ë-Ó?K÷þT²ÊÖIÉ*Ëßï½åËËo¿ZîØ±CíÕÞ*æÂKVµÀäÙÙÙçÎê)ê÷«^òÒ¸víJ²~0üiRìya÷~Û®¿¿ßÿ³6¼ê	eµ¬j;L:.ðk¬µJ	¸òÅPÉÉ­ª6nß¾°f5¬ÔT«MÕñË7nH«_°X×êCKµõµ´eÉÈÈHÖ$á"Åÿ3©Y%Ãôyi(@µßkX¨U­¤¤$ÙÑÇpÀdôôô¸¸8)¦%¥'«¾'åñêÕ¤¯ô1×*ü¤p%YÔuµUÚ£>tíÕê<õ9Ê­:înØ§ªrK~ôíäAÓøøxi~]JvJì^õPûNôäW9ð¯ý!ò~TªØÖUmPª÷õêUµù¢ßC²dEdß)Î×jýâ¿-H²~ûÝQuXN=æüùój«í8ñ­[·enii©zü%K´=/¿ü²ÖIýuïÌ3A!©´RRR´·-ëz©ôeÐTÉªþXÚÿ¢§§'&àÛZí_þ,õ¡!YôÊµA ¶x¦:ÑIýTÕîZò©0ÎÉÉQG+µ--Í©S§å/RO|l©XUû]ýÿ§Óð=ÈFÌTÛajË@;À/íâââàÉ*Ûaªgd`íÚµÚdÉ¬£££*6nÜ¨Vs÷MVUÓ¨òÒ¥KÚ­ÔÚDÞhe®v<oªõøñãß~wLthhH~ÅüOõ4m«U­Õ®"õ®þL«PU«?WÙ¬(êåIÒi¡'kBBBgg§lshõhdåWT[èò®$Ø¤°UÚE¯Þ¹Ú:ñß¯+>¬p«È[ÒÏjojYYÙÒ¥K·oß.wÕæ²i¥^|ûJxË'¢pêè¯ê^IqUYúþþûï>ôôt­-ïÓ?YU[*WµÛð¬ YµÿM*cüOQ+kµUg èÏ¡¸JWÓ¯òL-¢KîJ¹ã¿7rZÉªErÀÓMåë×¯^³ª?AíuÔ¹o²8q"Õb±È]íôTÿàÉ*Õ¹áxªzA­¼o²Þwo°Ä¼Uyc*ÕQLu¾ôêõgj+ïDÛwª#é¿÷ßªñññ²Y ~ü:UÁKcùòå«V­Ò¹òQ¦¥¥©´[ulXh ú£üOXþv-ïý7õ½¬jSOUÏj#++Ke6ÉQ¬ÈTjêWvÚrÃI×#k|Y©Óú¯d5ªªYgÉSÔñ-uæÔFjo°Ëå"ÃÿiÉzß½Á÷-ÊýcLV|*£¬ë¡kVí=«=ÚfÿUBNâDÊwýX	$	0µ£^Û÷«6YùJªíu°Üv`U>>C¥«Îk³ÛíZ$QzêÔ)UP~;yðU;´)_BÃØÃ(Ûà·ßíÐVÉ½÷îUý9êü¥ªÞ÷¯Z]HVdddDß~wI~~¾VhçthKN§~ÔJJä©U[ñ©°QªLêÃ«ó¡´#5dñÅe½ïp8Éªr%¼½Áê±!YÕyUZz²J	exêmíLìðjV­òSïSev.®|³FhïUØ¨K^ª1¦r«>AÃWåâÅÚ¬íÞ?sæ¼¸¶/7à¡ÖÚ¢2<FGRg5º.x²ªï¶D6,´`V;Þ©YA²"|²bUªíµ¶:	SÖª5¬$U>YÕ.5m¡VIjª:rù­n<«$*!dí¦Úª³oüÇiD1YÕF=ufMiiéT3:¬ÚaEm¦C²ª«ê8«üHv;YUÉè?çÆ´Umè¨³~Ôeí-Éç¥k¯ þõµñ¬òW¨ýºªzÕ¹ÁÚ®xéÌ³jLÕê[¡?: £6Ù!MCÙ,ÔÞyeí¼â©6¡V«Ú$+GµCXÅZ±ªÝzRÑª]vúdÕÎà5¬Ô4úZQª@ÕUNSë>­DYºtéªU«Ô^UiêÍÈz`Ò;uòªÚs«=KúQ7BPíwXª4jþ C²Jé§úqVÃß~7Õþ,ÖPU?Fm-¹ý.P¬Úå«ÎÝU3rh;iµdUçèÊ6Ä§ÓéMý~õ	NuÞïGXiTcyCºðU%´Ã°I²dEX_ÝÚÄÿèdÚCâÄ	ýæ¼¬RÇ¨©*OýU¿Uû%oT*ÈºX]«jT?jw:ÑIU±ñÅÅÅjX­üY_«ÉüTÜ®RÖï¡ÌY¨âD^JeÿÚS?Ï°¼C	uµ¶aSÍ![öìQE¿ÔÚòS§NIÔI¾¦§§Ok<«6Çá+WdãFZàÔïÆ²^²O*!!A;«VeÚ³Ôù½j_ÿ]Û§í?¾f-,,üö»)Ô'¢%«ÿyý+-Ò@ÀÖw :<]'tWnn®ö¶§JV­ÔHVL:wC¯/éÔ8KUò¦YW'ÊË0SºÑOf$!$ /Å´¼ÑÎU$zßÿý7Þx###CJ:uäRy÷Ýw§U¶ê×A¨ÙÙÙÓ-¼Ô8¢'Ê¸­£MkØbP5´aØ¨aB¥éRÃfù¬[·Îþ½i§©ÍÅ¢o²0Õ4ß~wXÿé¿ßNÇ¤¦´ÔÎ5Çþ)+y©î5lPfäW³êù>$HV ZZZ$;ËËËÌã¿:Ó"Mí/5¬ÕÁ¿ Oä´IøÃKíM.Ã´÷¥v39ávhh(66Ö¾eµçºV²ý4Õe	2?%ÀJ¬¬¬dddd$+$+$+ Y Y YùðóÞïé­Tø#G>|XùÁy©ÑÑÑ%KlÜ¸qó¤öövyJyy¹þ1×¯_yöÊBkËãåéÚSbccäýèø÷þ¬À?®I²677?66¦IIIkÖ¬	ñ·Ü½W^-;;[¬[·nÕ?f|||bbBµ¥!ÈÉÉ*òµ¼÷/í7Þxcª.ô7d¦¤%¥Ê=-ü>ÿüs¹=yò¤ÿÃHVýëÈ]·Û¤ð¥K'«zÛñññ'kèoÉL©¿¿?77W§O,¹zõjð5d5Ô¬%%%S=²¥¥Ejè+WÞ¹sç³Ï>à¼råJdMHHV²ò¬Àê3¹Ýµk×íÛ·¥qãÆC²¯¤í:pUG"Õä a&/àÀýcÇÖÓÓ£íls8jùµk×d¹ÓéôÎ/¿üRÚ'H²~ð5Ä7dîãwÞÑjFFÍªí7VÍÚÙÙY4i³U¹®ZµJ=RÅÅÅÚ%Æô¯¯JÉIév»>_ä5kèoÉÜT«ªrýö»sÔ^ÖÈ÷«<Vµ 4$M-KÇ/[¶,++K;)W+åþÁùÕW_ÅÇÇ¯]»Vb/*5koÉsýúuIÝ»wË­¾0âqVy)+¬ÒY;vìPnxä/¾èÿtýÎd4~øáÒÞ¾ä5kèoÉLéÞ½ýýýß|ó4>ûì³¨'ëèè¨?©,ï[³N7Y%¥Êí Yð_sìØ1CõêÕª¸<uêÿSzeyè3'h	-/.mËe¨SýËMÿ±S§*[_íµh%ëßìæÍKHEðêXlcccð×Wgý|õÕWÚÉTÉ×I,·Û-?MOO¿sç>ØüÓNÿ"zSãá¾dd$+$+$+ Y Yùè£-[ÀðäOþáÝÉú_übÝºuWþù£ºgY²þõ_ÿõ×Ì/¾ø"ÉÉJ²HV@²¬¬¬$+d%Y Y YIVÉJ²HVd¬$+d%Y Y YIVÉJ²HVd¬$+d%Y Y YIVÉJ²@²@²¬d¬$+$+É YIVÉJ²@²@²¬ddd%Y$+É YIVHVHV@²¬¬¬$+d%Y$+ÉÉJ²HV@²¬¬¬$+d%Y YÑ1::J'¬èdjYYYAA]A²"ÕÖÖiµZ½^/½A²"*U+**L&SFFÆè¾þþþììlÕÊÊJÏG¬ðÕÔÔØívËuöìYzdDTªæääÄÄÄRª¬455%Ojnn¦7HV@ø¤<-,,RUné¾¶¶6§Ó)¥jkk+½A²Â7::ZYYi2rssûûûé9¬ííí111$+|ozÓÒÒÌf3S@ÌÁdOOO'YàS__o±X233W37uçÎô¬Ëý$&&nØ°Ï"§«©¬¬dý¹¬7nÜÈÈÈÐµÎÏ_ýÕ_åææòY@Ô¸ÔÔÔzcÎ&«Ûí>uêÔÞàÑ«¦Cær²Æü9¢q5ó+YõKÍÑÅ¸d¨a|OVf(ª®®fÉJ²@¨q5êÒª«!YIV×ëµZ­©©©ª$+ÉTãjWC²¬¶¶6uiÕ£GÒ$+Éáóù|ååå&Éãñ0®d%Y "«!YIV5®&;;ûÂôÉJ²@øWC²¬5«!YIVÆÕ¬$+DM[[Ãá`ÉJ²@¤¤<-))aÉJ²@tuu¥¦¦Z,ÆÕ¬$+DªººÚl6geeõööÒ$+Éáëîîv¹©¦¦q5$+Éñz½ÅétvuuÑ$+Éá.(()))aÉJ²@DZ[[¥NeÉJ²@JU5D~~>ãjHV"Â¸d¨©ªª2Íd%Y "¹¹¹&IÂÕçóÑ!$+Éáãz5$+ÉÑ188èñxbbbWC²¬§Ói·Û[[[édðIyZVVf2òóóèdHKU«ÕZ__Ï$À$+É©¬¬R5;;q5$+ÉÈÏÏWãj(UIV"âõzSSS¹^ÉJ²@DÝn·º^S@¬$+D¤¹¹Ùn·Kµ*zd%Y |RL&ÇÃ¸dtww»«ÕÊõjHV"2::ª«áÒª$+ÉéëëËÍÍ5ÍLA²¬)¯×k³Ù233¹^ÉJ²@DÕª$+Éinnv8ÉÉÉôÉJ²@øÕ¸¹åd%dtwwK©j³Ùédð©q5f³977R$+D¤¯¯ÏårY,¯×ËÉJ Y "¦V«5''§··ÞÉáëïïWS@piU¬R5999--R$+Dd``@]Zµ¨¨hxxÉákmmMÔÖÖFodL¦üü|ÆÕdhVe¬©ºº:³Ùìñx(UA²@D$Jsrr$V½^/½"-U­V+VÉQ(Ussscbb¸´*HV@¤SSS;::è¬$+ðù|¾ÂÂB)Uå) @²¬"ÒÖÖæt:¥Z=tè½d>uiUÉ×××Gd%Y¯··7--Mbµ®®@²¬"R__o±XWd)5ªeeeª YIVQãjN'ãj05&&æ×¿þµj¬Y³F§Ov¹IIIIOO'Y|Ï´q5GÚtfY²~ôÑGª!ÃKûÆÒnnn¶X,$+ï6®¦µµÞÀlJÖ	N¨ª!Ûr»wï^¹½yó¦JÖ¤¤$À÷CWËõj0ûubbBªK,QÉúöÛoË#GèkVÀ÷q5õÉªíÞµk4,ËÚµkµdúUqqq¯½öÉàA«®®µPFFä+½Y¬kVöø~¨q5²Ú)//çd%ÌÍU¥ìÈÈÉàAóz½V«ëÕ`'ë¾ûdaqq1<8ÚõjèÌd=uêÚñ+*iËÆãÖ­[8 CÍàAhkkKNNúýû÷Ó;É:66¦òõØ±cãããÚëJ¾¾ñÆ$+Áçó«q5/_¦C0§UHJ*íÎÎNi<xPnIVQ§ÆÕÍæÆÕ`&ëÐÐ¨d§¶äÜ¹s²äÈ#ÒPûµúd	m×«ÁÜLÖ±±±ÿúUU[rýúuUÅ¬"Áõj0_jV®uà ÆÕ8ÎÎÎNz$+É |Ú¸iÓ!/ÉzòäÉ@t©q5¢©©=Àw5«lQ~øáZýúõÔLÇ'YNWãñx¸^æW²>|X¾÷ÍÍÍÚ¼ÁÇÛÉT-YÏ;G²6®ÆëõÒwÉ:11!¯%ÙyåÊýúÎ;ïèïjó1¬Sãj²³³/@o`>&«¬«V­Ò×¬Ú¬$+©hãj*++9ª5æâÅiiin·ûæÍr÷Ë/¿¤f:íz5Lõ[u`UÜ¾[åèË/¿,÷îIÁúöÛok#YøÓÆÕÓ! YÿäêÕ«r;>>¾dÉíÛ·~tëÖ-FÝHWsôèQz$ë|óÍ7Yµ¢¢Bí+&Yè1®$ëîÞ½kØ*AËL`HÖû¼Y=Ildo$íåË¬ôW5ÔëOU³Úû:sæLzzz\Ëåj	._¾Ì¸¨¶¶677WV¹R uwwÓ!9YUµª®j£W­V«aý5W®ÞßV­Z%ÿÏÏ[o½E²³×ëRq53MiiiZZÚÑ£Ge»§®®NVàtËÃLÖ÷î=Ðã©			ÿó?ÿÈø,Y¡¿¿_!ù·-//g"ªÝnÐìß¿_¶~è¬ú£­òo£¹¹÷®Ümllä5zz¶lÙ"ÿçGþK©YYq53YYYa¡|XlÍd=ú´Dé3gôÉÉ¥åîÜ¹#ñ9Õ1Z³3Ïç+..6LÆ&kii)É:CUrôðáÃííín·»¹¹yß¾j2&iþùçÒÞ½wè¯644´yóæ 37¬ÀwöìÙ´´4«ÕÊ¸¬»»Ûf³éÇËçÅÞà¬ªB½tévØU*NY"AÆ«uvv®_¿>øÌM$+0UVVJ©Í3_UUÃáhhhhkk+++³Ûí]]]tËÃOÖ]»vI9rDÚÒøôÓOevn°~þûJIIÑ©D²³Wc6«««W3[:tHV×yyyåååò	Ò!?YÇÆÆ^í5À7oªÝÂn·Û°£xß¾ÌÌy«ÉdUûåöÄê®ª_õãY¹>+0·1®$kÔõÆIIIú°¬Y¹>+0=zq5 YÈ¹ÁZ²R³ó§êz5«Éú`Î=ëp8,KSS½@Dêêê¸^ð0kÖK.MLL¬ÀÐßßk2jjjW<ØdÝ·ovMVCÍZVV&·§O&YYÍëõ&''»~­1YçççWTTôõõñÁaö%ëøø¸ç=¢[¬ÀÌ100àv»Õ¸Ï7ßjiiijjjccc[[ä«Ãá`*(ÌÊõF²ß§®®.»Ý.ÕÚÚ:Ãßª¤©¼OýeÑ¤lõx<|MÉÚÓÓóÊ$Ï¤æï¨¶_Ú$+0Iy*EªÙl.**S@HÖÔÔªmÍÆGÙ¬ãããÀ¥»ví¢ff»îîî´´4¥Y4®F¶ôKFGG­V+&fS²<df»ªª*)U³³³g×@YYYú½^¯,áÉJ²DiNNÅbãjäKºÝnµA°ÿþäääÎÎN>VÌ²d4Ýºu«²¬À,"Õju¹wááá5?¤,³c¶Ö¬ú4¶ügª3Tdf¾ÁÁÁââbÉT^^ÎÀKVö³l«ëÕ´µµÑÉ |RJ©ZPP0gTÈÏËËKKKÛýû÷óyd%Y­³³ÓårY­V	°øöêêêêåËåÖétÖÖÖò©d%YªººZJÕ½|ý|%ÌõÛÑÑQQ$¢$P%V%ìÉJ­­­ùùùn·f× Y£¬íííê.i¡¬µ÷ìÙ#wãââHV`8tè2ÃÇÕH²ÊêÅ°P­Xø1õÞ½ÌÈÌÚõjÊÊÊfþ$ÀòneÀo°ÜòQb.'+×ºf)õl6ÃáE3'ÔÕÕÙívýLÙöd¦¬Ïºººô×>"Ï'EªÉdÊÏÏ@¿ânäåå¥¦¦æææ2êó4Ycbb^yåÑðð°ÇãINNÎÎÎRÂ/ºûiµëÕ:tÞfk²îÜ¹S¿äo¾!Y©HAVPP ÒTnÝnwQQQ´JUuiU©öfàÉXggçkÔ	d]»v­vôÆÆFY"¿dü=ÖétêÇ½Z­ÖÈ÷ÙÊ+K©j±XêêêMÉzïÞ=ýeÏ÷íÛ$ÝìBqèÐ¡ÂÂBÃÂììì¯zÖÔÔ$ñÚÝÝM'³,Yý¯%wòäIÙÜÆÈÈÉ×ÕÕ¡¯)¥m·ÛÃfHþû$ªM&SUU¥*0ë5fj?~W®Y $üN§~"¤¬¬¬ðBQ*àääd	f®HÌúd½wïähRR©¥¥E×¸¸¸k×®¬?¯×÷ôÓO/^¼XòUjÍ´´4)aÃ8È:<<¤®W3ëÆÕ$ë¹ûöÁ,YÒÓÓ£9rDõâÅÒ)//»ñññòHÐTUUis HÄZ­ÖÿüçaT«ò¬ÔÔTÍÆpO`Ö'ë­[·Õ½½½7oÞýâ/ô§fôúúúóöI:Êédõù|ÅÅÅRª2®;5«4B»dªüK	+	T^iïÞ½dôÎ59­Sy¥Ø§ÍfÆÕs-Y%;;¦URpUË%Y/^¼(wÞyd4Gõ¿>Ëå:öl¯ÐØØh³Ùä)«æZ²ªÃ¨RªêUÿ|un°,ÿàHV@ó»ßý.!!A¶8µ(mnn¶Ûí¡ÙÙÙòo5/­¬Qu£°M6nÜ(í%K0êÐ,ôx<ãå_6O*..®¬¬4¡2MMMêz5mmmt&0ÇkÝ¡ÓçóI[Ò±  @ÂURö¾ûeUxæ_Z Y£¬/^loo×¿>|dôÒÒÒô§ûNÁßÜÜìt:WÌÇdÕOÃ$-eIV@±Z­£R¹æææNõxy°WÃ¸`Þ%kgg§¤ééÓ§ÕÝk×®©HVÌO]]]UUU¥¥¥õõõÚþÛÃñÔÊÊÊ¯ Õmff¦±¼'+ó.YÕÐUÃ9Àb¼ÉYA]m¦¢¢ÂëõºÝîÔÔT5ÉþþýûGoo¯zXss³ÍfÓîêKÕÅ"IúPs'YÕ¼Á[·n·dÅ¼"!,5«¶D"6''Gµ¥ÈbÔårÙívÿ³|/_¾e2äYê+YïÞ½«&Ð.®]öü³Ï>[·n:øzîÜ9ýÌa«®J@JmªÅ¤4º»»¥õßÇ«æº6ÂK´ÅÉ*©yõêUuVuwÍ5êG---rWumsÉ`såûï¿/å¦a¹eð«ÐHÜÉ?¤2'+ó:Yýö½÷ÞSíûöÉÝ;wî°7óÄ¡ÛíÚtéÒ¥qqq¿ùÍo´I*AËõj5°ë×¯ëÛH;--30HÅ)ÑXVV688øõä9Àåã?V±*kCCÃT5nee¥ÉdÊÌÌTg9 Yÿ¬`åWôweýB²b>àÌËËÓîJ¾þøÇ?^¸p¡dªT«õõõÕÛÛ+[f³q5ÉÀÉ'%JoÜ¸¡OV·ÛM²b>(--õ¯J%VÏ??ÕSª««ÕIÂ«HÖ¾üòKÉQuÓÕ«WånccãdÅ÷c`` °°PNÍämUUUú%óõúûûsrrL&<R Y&))©¥¥ÅÿÒ7[¶lavCÌ^ÃÃÃ©©©%%%uuu±Y5]]]V«U?çT±ÚV=5®F^R Y¹Öæ)CôK¤Ä4,ÑSxWef#	ÍõjdÅ|)Oý£ÎãñæøÕåry¾¾¾ÆÆÆÖÖVÃ~à¶¶¶äI¡Éú¿ÆÆÆdüí·ß&Y1[tvvJi·Û%óÒÒÒô3Iyj]ÚÕÕ%î¯U×«¨fduÖÒÅõ¿ùæ3Soo¯Ífï­:HM£¯¦äõz%kõµ¬Ûí.//Ö¯8ö¬¼¼¬¼¬!]9N.¸¹¹YbUÖ ÒÐÏ¬¿h+ÉEªÒýºº:ùÞjwKJJGmmmCCCVVVnnî´¦ÈWS@dggSª$k¨ÆÇÇµöáÃÕ×®]3¬lJpÖ××k'åºÃ¹¾/_S¿¤­­­¢¢BW=V¥$6ÍÕÕÕ«HÖð¯x£®ÏºgÏ4]0ÉðH¶I%*ÕgYYd¤ÕjU¥jNNá"nþ:¯×k±X$¡W¬áÛºu«¤iIIIsss|||BBBó$i¯_¿dÅÃUYY­Uv»]¾µµµÚTidffVUUýúûûssså) 5¢d5LuNÒ&8t¹+.)¾Ï!99¹lµ²uv"655ÉKÉ0® Y#JÖ'OJ>ZBôÒ¥KþÉú &å'Yq_ÝÝÝR>Úl6I»¸¸8Ã¤mmmòSíu:::Âû]R«) ä) 5ÒsUI*3*V­V«¦RJ¦&%%=óÌ3úó*++¥<Êï/¼äwkk+=¬%ë­[·d¥E,É#''Gá¶ýû÷Ífíjss³Ô²ú9~Ã£]ZUÊ_ÆÕ$kÏ`Ò'kJJÊªU«®L²Ò!YERzJ1*gEEÅTÒ0BæÍ7ßËÌÌt¹»ÝpJp´K«2¬êâ¬7oÞ¶¬¶$Y'&&vîÜ)wïÞM²"Z¤4LMMÍËËptª¹äK888¨_R[[ûË_þRøìÙ³µË¥U<ðdÕÚÊ+U[]±dE´äççiw/ ázùòeÃÃõQ%JNgTriU!YÕÌÁ(ÒpÿþýíííK.5|[\,õ«áñRÚ=zTèr¹äa¿.­àá$+WCIæåå9Éç>66ÖplUjGÃÜ¿Ú«ªª¤Æu»ÝõõõÖRªz<.­dÅ¬'¹(ªåââÅôÙ&õèîÒÜÜÌ¥U¬l6Û+W´dõz½			ÿôOÿôõäIÂ¥¥¥YYYîx§ü5®¦°°ÐpJ¬~ùË_>òÈ#CòÕãñ¨lËÏÏ_´hÝnyyyÉ¢èòåËq5HVÌR¦¥¥I|K¦JÕ(9'å©$kSSê´.:]êz5òÉYIÒT­¯¯OUrN>zÿ±ªQ/U¥6L4¼¬$+¾?555-p]¾|ù+Ô¹Á)))/îêêzp¿WM|ÚÙÙÉ§dÅÜU	Ñääd)U³³³_õÕ¦¦¦6ø])OÔhlNV@²bîPûU ËÉ9«ÕúÏÿüÏrÛ××÷ ~©T¨êz5«@²b.PÇS%Øl6|AøÃ~=99CZZäkee¥ÃáxôÑGÄ¸U)«ªªÌf³Çãy@±d%Yñ©©©©%%%Rú|¾ØØØÿú¯ÿR±wèÐ¡êêêÕ«W755EýW_¾|9++KJäñâHVú%K.î¹ç´»RÎFÜjCCdj^^ãj¬$ëÜQ__ÿØcÍæ´´4múßû·SÓy½ÞòòrÍÝÃ¥êz5Rs½$+É:wHjJ nØ°aÿþýÝÝÝ¥¥¥²¼««ëÙg­­­-..vªË§±±ëÕ YIÖ9¨¯¯OÑ)L%_¥-'enn®:+8ºäW¸Ýn)UåÅ¹^dkZ[[×¬Y#j±XO"õùçw:ùùùQü¨££Ãn·'''KÎ@²¬sÇãY´hPIn~úéþð+V¬ØºuktÊ«I*¥jNNN?=d%Yç ¾¾¾ÇüÑGmkkSK$h£~.ÌÌ´Z­¬d%Yç¦Ë/¿ðÂf³ù±ÇäGÞ|óM¯×[ZZ*K~õ«_EñÕ××«ëÕp²d³zm6[aaannîðððÖ­[/^]]ýÒK/577Gå·Há«®WSWWG©d%Yç2	ÑÉTmW°¤lyyygg§ÕjÊ®`)Uå¥WtëÀlMÖsçÎÙíö¸¸¸Õ«W<ydcþò/ÿòoÿöo¥l]ºtéäî¶mÛGrrräÓH)¬®WÃ¸$ëÿòx<÷îÆîÝ»7nÜ(F?ëÖ­(ål´lÙ²5÷WW×+zê©göòåË¾ò¡CdLBq5HÖ?411!±±±i,ñ¿aÃ>ËYDÍúáÂ«ÕºvíZm¸ªjlllW]ò´¤¤Äd2¹Ýn®Wd5Øfoðl$WXXh³Ù¤Ûþô§iiiuT±¿ÿýïÃþR¡:NÅ­³`®%«T0Z[jSuVËÉÉ(ÕÖ+W>ùä_Oî¹-ÔØØ¸bÅð®6£]Z5++RÉ:¥¥K©½ÁÒ&Yg¯£GJI*ù§.¼úõä¾ßìß¿_LSSSjjjcº»»åÅ%V¥ðúT0§uÓ¦MöìÜz<uöª­­ýÉO~"Ái·Ûm6[fffooï³Ï>kµZKJJ¼^oqq±,clTº©¬jrD Y9úô²eËbcceu|æÌuö*--omm$Å¥äèO>yâÄÕêêêé<<<$±*/Î¸$+3EÌ/ééé>úhGG:Î*~ô£-^¼8ìI$¤Õõj´Ùd%YçÇlÃ,|MHHxê©§RRR^xá0^JÂXjÉTPPÀõj¬$ë<e±XòóóÏ?ßÔÔô÷ÿ÷+V¬ÈÍÍ-**îëtuu¹«ÕêõzéU$+É:O:théÒ¥?úÑ´wÿã?þcáÂúC)UÕ¥U322Â$+É:ë577§¦¦.Y²$!!áñÇ·Ûí¥¥¥RtJÙúÙ¼êÒªf³¹¶¶ëÕ YIÖyª­­Íf³=z´±±ñ­·Þª®®~ê©§vìØQWWwöìY§ÓbéYSSc±XWoo/½d%Yç¯¬¬,µ¿·¿¿_Ô·Û]__¯ÂRMüåEbbb*++ÉJ²Îw¦Zvªúõùç_³fMvvvjjêPyÔµI¬$+þdÅÿçÿümÛ¶íÝ»÷ëÉKÜ¼ñÆ999RÈØA»^Çãa$+É?9tèPll¬¤£ä«Ü>ñÄW®Ûí÷½`jgg§ªRï666ÒHVrþüù¾þúëY>ùäÅKÐVUUyv½¼¼¼È/~$+É:wlÛ¶MTRz.X° ÈYH½½½RÝÖÔÔ0®ÉJ²âÏ¼úê«ÙÙÙÿùÿðñuuu%555kÝÉJ²Î¿úÕ¯¬Vkgg§DfýÙ³g;°fíëëËÉÉQãj8Y	ÉJ²"°7nÄÆÆÍæòòòI/½ôáammm#99ù¾§5ÉJ²Îk¥¥¥&IW©V.»ßýN¯eeeêz5Úuåd%Y6saûoûÛóçÏWLR?íììLMMµX,ÉJ²"$=öØ[o½ìp8l6[mm­hIIÉèèhee¥ªùùù«@²¬Äç¢Eä»588øõäpËµzõjÉÔ´´4³ÙÇ¸$+ÉP577;Nu´ò-Ðb±dggsiU$+Éé©­­-//ßºuëc=(·²111Û¶mcdÅ´ñÅ?þxVVVCCÃÆ¥Zd|e0dE8<O||üÞ½M&Ó«¯¾úè£¾ðÂôdE8ìveeell¬êO~ò)^eIII	=d%Y1m>Ïl6/X°@uÉ%MMM¿ùÍoJKKé$+Éééëëægbbb$P%bûûû¥-ß³úÉJ²bêêê¬V«Åb)))Déèè¨krròòåËHVÆR5??ßd2UVVJJ þë¿þëK/½$)k·Û_xáââbz	ÉJ²"$²Ùl rwÉ%Ë-»²077WjÖ²²2:ÉJ²â>ÝnwLLLQQ¢­­->>^>¯¢¢âé§~òÉ'[[[é.$+É`Ô¥U¥0¶0++ë/¾ÈÈÈPÓDTWW'$$¼òÊ+tdÅFGG%2Ífs^^áÒªV«U~*ÕjIIÔ¬Û·og$+dÅ. %©ÉdR§þ~êt:×«ªªÒ.Ë$+É?SSSc±X233»»»> ¬¬LY-q%m6[WW]d%Ya,Ussscbb¤22U~$É*«Ô©ÅÅÅV«ÕëõÒHV¦©©I2255µ££#Ç·¶¶Ju[__Ï¥X¬$+5hAAªååå!^Zµ¥¥å¥^Z½zõ¦MèC$+ÉÿÕÖÖæt:m6Ô¬!>åg?ûÄ°Åbq8.ýýïOO YIVJU©&)??¿¿¿?ÄgIµ*±êñx´%qqqô'dï:::¤î¬««Ö³ÔDÁú%===µüãéR$+É:ßN÷)«W¯NII1,`;¬$+Âñ³ýláÂú³vìØ!5+=d%Y&Éd±XzzzT¬JÁºvíZºÉJ²"L©f³YêTÉT¹î¹çè$+ÉHqlÉJ²@²¬3ÏððpWWá7@²¬ºNVVVê¤³gÏÒ'HVaÚ¿¿ÃáèííUwìv»áÒè@²¬UFFÆÑ£GõK¥¥g¬$+ÂaµZµk+^¯·¸¸@²¬GZZá¬UUUôdE8*++³³³>ºÛÝÝm³Ùä@²¬¶ÞÞÞ7ß|sÑ¢Ef³yÝºu7o¶Z­ôdÅ´=zTÊÓêêjÉ×?üð?øÁ3Ï<Ã$+É09Nyêóù222è$+Éi¶Ûí³ëëëKKKé$+Éi599Ù¬µµµeeetdE8²³³õ3B:ÎÖÖVzÉJ²"½½½R¶=z´©©)--­¤¤n@²¬ßÀÀÀÏþsù>y<ÆÆFÃÎa YIVL¯fÍÈÈHMMÍÎÎ¶Z­$+dEúúúl6[]]JÓþþ~IYN@²¬SMMMaa¡~ÉügçdEH¤<õz½CW:ÉJ²"Õp&°dªÍf£f@²¬:ÎÚÜÜ¬îú|¾¼¼<FÝ YIV¯££Ãn·çääJÃãñh×dE8$JÛÚÚöïßßÛÛKo YIVHV@²¬ddd%Y$+ÉÉÉJ²ÎX¯¿þºÍf3ÍO>ùäÞ½é$+É0ù|¾'x"11ñO>éèèxõÕW,XPUUEÏ YIV£¸¸8>>~ppP[²mÛ¶ØØXzÉJ²"¯¿þºaáÂ¯Bç YIVLÛ/¼ðê«¯êø|¾üñ¤s¬$+¦­¶¶Öd2?^["%ì¢Eè$+É0=ûì³<òHqq±¤ìsÏ=·páÂºÉJ²"|Û¶ms:O<ñDfffGGd%Y YIVÉJ²HÖéÌ3éééqqq.ëÜ¹s$+dÈÊ+Õû;pàÀªU«HVÉ5			r»ÑÓéÌÍÍå³¬ÓÐÓÓ³eË@²FÁ;w<ÏÝ»wÙ Y§-æ;êîÐÐÐæÍGFF87@²Fª³³sýúõ·nÝbÔd@²2Sd%Y Y YIVÉJ²@²@²¬d¬$+$+É YIVÉJ²@²@²åwÞY´hÑÊ¨r:Ë/_»ë©§¢BôüÀápÐ!zúé§é®iîN£Bï.ùîkZ,Y¬×¯_ÿ¿Ñö7ó7¬ÿ¡yóÍ7,YB?è¿øELLý¢wßWºëã?¦+BñÑGIwýã?þ#]ßþö·Ò]÷wÝýùÑÑÑÙ¬Â?üÃ?¬]»ö[æÓO?ý¿øú!D---S]lþzzz¤»èPJwýá +BñßÿýßÒ]þ~~ÉJ²¬$+ÉJ²¬$+ÉJ²¬$+ÉJ²¬$+ÉJ²d%YIV$+ÉJ²¬$+É:üñOúS¾v!Ú·oßücú!D'NHMM¥BtáÂé.ÏGWbddDºëÒ¥KtE(&&&¤»þýßÿd`ö!Y Y Yg3gÎ¤§§ÇÅÅ¹sçÎÉ¹µÛí²dõêÕ'O¸tß«¨koo×Nï¢»»wïÞæÍããã/_ÞÙÙI']¦ºâôéÓ¥»æW²®RÍñxàÀU«VIÃãñìÝ»W»wïÞ¸qcÀ% [ø^E×øø¸lhÉJw÷þûïðÁN§.))éÆÒÛÒ]ówopBBúäû*±±1õø/ÝÂ÷*ºvîÜ©¦½êÛEwéI]uåÊàÿôF6>ÔØ-¹U"ßwÍÓdíééÙ²e4âââ´ªí¿tß«(J"##CÖkZ²Ò]÷ýïûäOd£MrâüùóôXpçÎ¾oÜÊ¿äCé®ù¬wîÜñx<wïÞvll¬¶<>>>àÐ-|¯¢Èív:uêOkïîºï_cc£4®^½*%ôXpkÖ¬Q%¾D¬èûï®¹¬1ßQw6oÞ<22¢î.]ºtllLívÀ% [îïUÿÚ?&Ýußÿ>CE/ñzwÍ¯µ³³sýúõ·nÝÒlÚ´iÏ=Ò[)8.ÝÂ÷êE,ÝÒÒÒJãÒ¥KYYYôXpR§ª©©.^¼(õëCé®ù¬)))åÓ§O/[¶,66Ön·9s&àÐ-|¯h²Ò]ÁÝ¾ÛívKùqíÚ5z,¸þþ~	Té.¹öCé.fddd$+$+$+ Y Y Y YÉÉ zÿÆ11kÖ¬ñLjnnÞ·o,ùê«¯<^]2%ueòíÛ·«»wïÞ»ê*¡HV`NioowO´;þ¼ÜªKbéSðØ±cÚ´´4ÞÂIG9|ø°<àW^Ñ|øá²äöíÛÚSN>-K´Ë¨×lii¡ÿkîÝ»§/+å¶§§G5ÆÇÇUJ«Ý¸qCîÞ¼ySn×­[§ê¯&«ªØòòrÃ	`âZüòË/KûóÏ?öîÝ»ù,FFFTãÊ+pCCCréÒ¥ãÇKÃçóÕn·Kaªò511Q*TiHIºzõjU°îÝ»WxÑPõÊkj)~çÎýk Y¹Cª¯Wõ¥¤ ÚÇûömC²j5¨ºÒ¸Ú¬muVÿà]»vÉB	]i«0þôÓOeJbi|ùå|ÉÌ·nÝÚºu«$ÜT4JÅ)i'©2ýUî:u*%%EíÔÝ±c,¼zõê²eË6nÜ(ý½öÚkj²z®Ûí6´¼@²sÊ±cÇ´SÔqÖÝ»wKcË-*Y?®)ñ)?UûÕ!Rí «Ú|ýúuCpJ,·'NÐ])UµÓTÉËG¬À¢2Òãñ¨ðëïïW(a)e«4nÜ¸¡Õ ê1ê¸¬Óé<2I£¤IIIò,i«Çk±Íª~dæu¨õÌ3rûÙgi·sçÎåËkw>ö«ÍÍÍþ/522¢N÷OjVdæÁÿmLLYYj¤§§KãàÁCvU~*w7mÚ¤¤>&W®ðWP³$+0/¨¿£££Ò>yòäªU«T=*Ë%YUæIõ©jUg3©zôüùór+S»|¥`ÞÔ¬ÉÌêì$ý9G_|ñ,øö»5ÙÙÙ*õwoß¾­JjÉ*+K$§[³^ºt)H*$+YCðÞ½«W¯îìì¼ï³ZZZT+(_¾|ynn®úÏç-Y²Ä?Y÷íÛ§&PÇjÖ²²25Ý@²óÎªU«öîÝ«ÝUS5½ÿþûÚ;wîÊVµ3yÏ=¦ÉÉÉHVHVHVHV@²@²@²¬¬¬¬ ,ÿ?xÊR!MFH[IEND®B`


öüýý'Olnkmg;èSý»ÑÑÑý+W¯^Õå/_n_ÙYÍßCNNu÷NË¼ã/bn)S¦tý»Øï¦ìÜ¹S6559ÿ²EEEì@YÑ£Rff¦ÙÝteµîàk¹  Àåk6r¹þã?ÖöÚöýìõë×õ¥>øÀ¾V³»ÞåóÍ7º@BBóbgOHHHHxxxE­óÍ7íWevèª¾ÃÞ_+8`"¡/9TM4úë«ºÎÞU×©¯îÚµËZ£í¬56lèæíØuYµìÌïn~`k¹[£eeÕÂ¬Y³Ì&fmscíÝ»×>ñ'upíÿîÛo¿­5.§M¤ekíìwÑj.×%³³³»¾Ýíeµ~wk¸×OnÖ;Ü	(+zÀgÚM6mÞ¼yføÊ+¯ÅÛ/¬=¾Ö+B&æþ¾Ã°kß%=¬¹*sùûö½ØÁÚÛj7§:LÖa­ÕeÌqKs*ëêêöÈéééÝ¿'aÍXfbvNÎÊ+õ1,,Ìþ]öÞ;3eÕ½^Uëß÷ÝqãÆé_÷ððÐo­Ë-3Gb­Ês½+«9àòÔÔÔcÇuö-J¦ùwÍMoFy-=ÖTßTÖa;8üjv»¸Ãáû»ÆÆFçÛÚaä5¿HwW[e5÷Ã´Á­õÚæ.ÿÊ1»$%Äå>HãÃ$§fÚ¸víËÕadv Ölj¿¼páZnþi³¬Ë¹Ö-³lí´¬5W®é`l9?r¦U³÷Òaµþ]¦¬¶u_ÄaeÕÄÄD///ÓªT×eµoI]Þ¶ý2ãµÇîÕy@ÝGÕ²ËËÝºe­9ÏÜúhww8¦jº¥/ýØñ ©Î?¯j§Bîró?	sìÄNÿtü[¿~³ÜY¶­²;æç?ú´¹ûb?CYAYÑ·¿NÎ×èlÿâ|x­²þxûQó°¹ÌñãÇÍÖ:pªñÕ«WÆÜüü|sùÀÀ@ëÈÞ/¼`-kf2ÿyïÈ#]C4iEDDX?¶öõìcPge5¿¬i¤õ¸Ì¿ÔÖÖº¹Òõkv]ÍöÐ¡¬]lÓsÀÜãéìD'óU3»[å31NKK3VZ÷´,,++Óod¾EÕî¬ã®Î¿HTTËAwb:»fîXðk977·ë²ê~Ù2ºC0yòdë²²âµµµÕ´aÎ9f7wÇ²ÆÌ'O´>jV°A´÷Æs­Çó:YwïÞýãíÇD/]º¤bvçS=Î¶5e5»`sÍ»ö3­ºSVsR®ý²ª(æ1Ëë´Ñº_V__ßêêjÝç°æÑ.Êúâ/e3 «ú©6-»aÍM`ÑÜÜ;q>®+;vì0pèGÒJmgs4µ   $$dñâÅúÔÜ=²Ó ¨»VæÊíÃ·3UñÖ-bÝpæÑ_³yUq3Y:þÞï9ÜÖ²~Nç²eM®æø¶Ã-BYAYÑ'æø&Ï=p>qÆì¬Í>Ôb?Öå.I×f]ÞôÌ-bÖ«»wFö¨¬V]nªKN6­ëÕüæ¨£ªsÇ²îÛ·¯;3«>µNOUþ».«¦sÇSÍZÓäËzÇ£ÁÊ~Tý`¦æQLs¾¶Ùæ×´6¬~ëØ©y2ýÏÃåñ[s¬ÂÇÇGwÌ?¡ÎLðZ7n¬ÌÕMgj§æ41Ý±°~AóK9®	X¿»Õç»z7½UVsWÏLÏæFJJi6eeEO;2SMûÎÎZï°c2çõh¯ÃÃ´Î»$íFÍ4£¾Å<¾eÎ|Ñld'$$hÈpþ¬²Þñhðrç'c¸,«)éb¿Õå<ÔÍÕúÍënónUSN4¾ÛÄ*H9Poû5wYýIdggûæ4`ªëUÝ|®9¯-<<Ü¡ÒòÇ_­6õGèðÜgÙº¼¼@ÛíÚµÝ,«ùuÌCúMÍ¼«ßÚü¹º|¶4@YÑ-W®#Â·O0ÉÈÈ°¦ëkMTTýY7¦Jrgeµv|&6¦©&`?vìØaÎ²^Ì¬Ý)ë³Ï>ë°RûýÈÈH²®ôîh°yÀØ¡¬þþþæ¼*kë~Y5B9lmëLìÞÍ¬Öäg~NÓ0ë\ý%8¼juôÞÄÆ½4Ï1ÕGs:ü©8qÂú­ÃûGÑ[Çr]>Ôêò&3÷¨.cdÎjvØt]ÕüíYktÇÂ³9ðÎÌÊÞÓÕ4Õ:¨½¶9	S@3Î:¬¤)Á^VsHÍ>[]9 j¹üÑö|VFÃ)önæQ[söóó4ú±¬æH£9³&??¿³WtêNY­­Wºp(«y`Õ<Îª/]]V32:¿æFÊjîè³~ÌeëGÒíeÖ[×`~óïZÏgÕoaëPWWgÎ¶Åkcº|pÁáU5:Ûæ¯Âþèýé1öËX/¶åPÓîsôE×lWÜÙD²¢»Óªu ×Úiçhl«9¬§Ö²³Õ:×ad^fÁ¾ã3;JT«¬æá4³ï³&	]			5;z3¥Fûqs±û/zgN^5Gn­ïÒýY7 9îêr:45êìõÊªÑÍËËË:%ØÃÃ£û³:¼VÃ·_êÏ~kwÊj>¹·`¿ÑusËC¾æÜ]óÖAZ«¬æ]Ý§Q>£¢¢t×Á~ßÜ÷ÇgXY4Ïåí~êzWVShóÊ÷ )+(+zõÇaÛ8?z§¶#´ûöí³ß·Êª9ÆìLÍxêûnÔïUoL´/Ö°k²j£éÇ3':)VÏÍÍ5O«Õ¿¢ýµy1?Ûèèhíß»ó&'º*Óç½§ýuõ*êfoë²a½RîIlØ°Áý­õTêÔ×ÄÄÄ=ÕzÃÝ¹1-+öÃëÍÍ¡õÖëIùúúZgÕ±Ìú.s~¯y	_çÝ:¦íü2Rö5;;ûÇÛ/é`n«¬ÎÌÛ¯Áå«E:0`Û7 y0µë*;l:ÝpÚééÖÝYY­-ÜõeEs7,öÎy¥y^fÝDª=Ã1Í³nì/f¤)öQÌêuF¨¡ô¾÷Þ³gÏNJJÒHg¹4Þ~ûí­ö½gÌÁÏÔÔÔ^æyD.Oíæërî1Úái£/¨ÔSæi3þ¦LâüûÏf"dîFøùùÙ6Ýèìe¼ý±ýÿUüØqyIKë.zìõÒz¸×á¥Ã+òW7´?É§û/'	ÊôÊÊJµ³°°°×ÄqÞYI3ÇKvÁæÁ¿.&¾³^´[°wÕ·nAÝårxÙË;2º8áöÒ¥KÎ¯·lü÷ôº¸ÿÔÙÛ.9×§Ø@Y ¬PV@Y ¬PV@Y ¬PV(+ ¬PV(+ ¬PV(+0þÃ¸¹½ûî»ö5.]Ö!û¶;wîØ±Cüàº¸ªÖÖÖÀÀÀ9sæ,è°wï^Kaa¡ý2çÏ¡uÍb-ëòúvë3<<<|õórÅ¾ÞA~ëîü¨(+Ð©Ý»wÛë¥LÚËZQQápù[·nàààI&uó_¹qã®-55Õ«EÙ/ÓÖÖÖÞÞnµ ¤¥¥u|«úùµ<öìÎ.ÐSÝùQPV SV)MÌ"""Ô«(ö>8pÀùQVûõèÓÌÌÌ.Æe3<y²ë²ÛÇÇ§ËÚý ¬þBccczzº:¤>ºëµ/euóòò:»dee¥fèèèèë×¯¯_¿^álhhè¢¬¾¾¾:³ºüQPV ÓQÝÒÇ?þøÚµkZ¸páCYÛÚÚ&u°yy)®æ¡Ps$¹éj·lÙb_³k×®ÖÖVÕÖÖZG¤×ÈÈH³þìÙ³ZåÎmÛ¶i9++«²öèÁ×nþ¨(+Ð7ß|ÓÔ¤¤$çÕ:nÜ£µºº:§Ã'f5ÔBnn®õê¨ýúÍ(é²---Y»ÿ£ ¬ÀhZ5ë·Ï	2GYû~4ØôØ£ZP¢üüüº¸|XXXJJuV°5ëÃùÍ7ßøøøL<YÝíûÌÚÓeþ¼Z²víZ´¦ýø8«®J½4¹Ò²Fd-,[¶Ì$ÜáÏ>û¬ó·ÛÐÚÓøÑGiyñâÅYú£ ¬7oÞT3¿ÿþ-¬_¿¾ßËÚÚÚjâ§Éò`OËªÚiÌõððÐý¾µG?*Ê¸.Ù®]»ãããÍÄvðàAço©««ÓzûK7Üñ0Ök9!!¡áÏDÑùhpuuugá4cë¬Y³ú^Öý¨(+àèâÅ.×ßºu«°°PiÑDëòæ±Ø²²²®¯ßvôÍ7ßXk*E«¨¨HÉÌÌÌÔW¯_¿no[NNsðìW2zñ£ ¬PV(+PV(+õX¹reXXX÷GyäOúÓ]ÖüÇ2eÊiî?ýéO]>­ü+ë_ÿõ_ÀàÙg¥¬PVÊ ¬@Y)+ÊJY²@Y ¬@Y)+²RV(+ePVÊ ¬º¬7oÞ`Ï¸qãª««)+²öÉï½÷Á´··+«QQQZó^z²(k·ÄÇÇ744XþðÃn®L>Û@YïÌËËkõêÕ¾¾¾X?®5-N^õUfVeí²²2->:))ÇYµOBBBìó+ePÖ>ÉÏÏßºu«N<BYµO®]»©i5))éìÙ³@Yy¥e¥¬PV(+ePVÊe²RVe¥¬ÊJY ¬@Y)+²RV(+²(+e²@Y)+²RVe¥¬PV(+ePVÊe²RVe¥¬ÊJY ¬@Y)+²RV(+à>tøðá3f:u²RV@Ô××geeyzz&$$ÔÔÔPVÊè¥ºº:«©­­­û÷¥¬rêÔ©¹sçª©111¥¥¥¾©0sjNNßj*eô¿3gÎdggµ9²ú_SSS^^æÔðððuëÖÁ¦RV@¿Í©¡¡¡«V­º|ùòPÞÐ§9µ  @s*M¥¬þS[ZZØ&ÐË9uÉ%SU-0§RV@ïZ\¬ ©©ÍÍÍlÊèEÔÌ©jªâJS)+ ÷M-))QP^XXHS)+ Osj@@ZPPpæÌ¶	eô©©æ¥¦¦&¶	eôCS9öKY4²RV ©²MeÐTÊJY _µ¶¶.]º¦RVÊýÐÔÒÒÒ¨¨(JY)+ôOS===³³³Ù&²@?4µ¾¾mBY)+ÐTÊJY¦RVÊ4²M¥¬h*e¥¬pZ·nM¥¬½±wï^777ÊÖºqãÆ¤¤$í322h*eí¶¶¶ÄÄD«¬ï8ÑßVzz:·%!¢¼¼<..NÅ¹sçÒTÊÚË/_¹r¥UÖ$'ÁÁÁÀijBBö3fÌ¨©©aPÖÞ¸páÚÙÞÞÎÑ`C¼©ÚÇSO:Å¡¬½yðàÁ?ÿ@À´ûvóxjVVÇ~)k?pûKÀÐ±gÏýRÖL,3+¡ÓÔäädí÷RSS«««Ù ²@_çTÎû¥¬¼RôIUUS9öKY)+ôÉöíÛÍFS)+e>Í©©©©jjzzú=Ø ²@/UWW¦rì²RVèuT5õôôä¼_ÊJY O>l5c¿²@ÿÌ©lÊJY 4ÇS9ï²RVè¦rì²RV © ¬Me¥¬h*(+eÊ4²¸oY¯MHSAY Oêëë³³³y%PVè·¦FEEmß¾ÊýÐÔÒÒÒÖÖV¶	(+ÐTPVÊ¦²RV4²Me¥¬h*(+ep¿ijjÊÍÍ¥© ¬@_566ª©4²è3gÎøùùÑTPVÊ ¯sªijhhèªU«h*(+eÐû9µ°°Ð4µ¤¤äòåËlPVÊ sjqq±DSAY)+>5uÉ%Öã©---lPVÊ Os*ç(²RVÒÔÔdæÔÐÐÐ5kÖÐTPVÊ Osj@@ç(²RVý6§ÒTPVÊ æTQ*(+eÐKÍÍÍK.ÕjªOÙ& ¬@/çT5UAUVh*(+eÐ×9ÇSAY)+>ijj2s*Me¥¬ú:§.Y²Ä£T\LSAY)+~h*ç(²RV4² ©ÀXV77·ßüæ7faÒ¤IZ8tèPBBBNNÎÎ;#"")+­­­¥¥¥¡¡¡4ÕEYW®´°cÇ-_¸pAË~~~CS£¢¢<==i*(ëÿª­­U8MPÍBVV>nÚ´I/^¼hÊLY845;;»¾¾mÊúÚÛÛÍ¨hÊúÊ+¯hÍÎ;í3+e@SAYv4øã?ÖßäÉ­²j~Õ×¬Y³(+@Si*(kÊêrfåh0@Si*(kÿÌ¬¦²W®¬M¥©ÉåËËËËKJJôSÏîjY7oÞ¬¹¹¹h*ÛdÐ¨««Ó-;cÆ%K¤§§:uÍÒÏe=xð 9ð« j!//OË-Ú²e¹3+0tìÙ³'..¦ÖûL111kÖ¬±Ö,]º499YëÙ8ýYÖ[·n¾îÚµ«­­Íº^õuöìÙRMMMM5§.ÒÔA©¦¦&%%Å¡µgÎaãôóÑà+W®èÿT-WWWkyëÖ­úXWWGY!ÕTÔ2d°úê«¯æÎë°R7ºöülþ,ë¥KôßIí´Ö;vLkvîÜ©å¦¦&s¬Ø_)+@Sñ:zô¨&Tû±ßæææíêÙ8ýVÖ[·nÕÖÖ:Ï¯ÚÊÖóçÏ)²4ºôôt­æsõ1333''Íòïu¦¢wLMCCCuëeddð¾ô@ïUUUidQSgÌQSSÃ²ª««ÙXÖTVVRV`°:|øpZZMîêÌªÿrôµ<mÚ´ìæ¦Ý»wwÿª9èååpìØ1ÊÜCæ¦©gÎ13ôñì ÜeÝ±cGVVVEEõºÁ»víÒÇ5Õ*kgt)::Úü|[¶l¥¬À=±ÿ~µÇÓÓsÐÌ©/_ÉËËknnnii)))Q_+î»²¶··ëºÔÎûüúæoÚ?µ^©§|õq¢ýHOOç¶Buuµ»ÁÔT£¸¸ØáéEEEÎOÐîqYÅeY5kÚgVëU#zú¶ê.ÔÂ»N(+Ðï=1XOÍÊÊªªª²¯ÑÀÀíû´¬'NÓýÜ/êÓmÛ¶õqf½~ýºþÜ¸q£ÁÀ]kêàSí4Û×è7ÕÝtnÜwe5¬Êµk×LG_xá-Ü¼ySë+¯¼b½^ÚiÁ]|eúê¢±jjzzúà>ï·´´499ÙþüKüà¾+«>ZÛÚÚ/^ìð¥«W¯öèÚª««§MÖõwQV _­¦FEE©:ïKêêê4§ÆÅÅ¥¦¦.]ºT¿ ærÅõ«¯¾Ú¾»¦s¬¼Ðî»²~ÿý÷)²]ÑÌZTTdwÿ:#""Ül(+ÐïêëëÍ9Jµ©ßu¼®EPPPII~Ùýû÷+¢Ú)é7]±bEfµ¬â>YUZ^) ©÷~Á²²2ëSE4<<Üá&à>-k|óÄVÕÇÇ§¢ÇGYû94:juÔáw,,,ÔÀÊ_²ZïÞÙÌÚÙù½hJKMMÍ~øóÿ|4Õhii	uøMóóóW­ZÅ_î÷²iÕ<uÕzöj@@ÃÎ.¥¬ÀÀ9sæR:jÔ(www//¯ÇìÜ¹sCç×OMM-))±>mjjÒ®éèÑ£üaà~/ëÍ7y¯à>T__¤9uüøñS5ÃåææÎ1cèlºº:­yyyUUUeeeº±téRþ0ðÕþh«Së)77nÜÐ§úk¦¬ÀÝojvv¶··÷úõë­#¢æézç/Í©º?1wîÙLÀQÖC)¥G±uàÞZ²ÂY^^^PP üîw¿³ªÿ#óçÏw¸pjjjuu5x0ÊªîØ±cïÞ½7o6/Æ¤åÏ>ûLËk×®¥¬@¿Ó@÷öÛoO4ÉÝÝÝßßßÌ©555111öSx._¾Ä»UFYÍzòäIëa×ë×¯kBËÌ+VL8Ñ<&44´¸¸866Ö:ìg^¡¹¹Y÷z322ØhÀQÖ?þXÝ¹s§uY|òÖXçÛ_ ²½¦´¢¢¢¤¤Dùüúë¯ÃÂÂ<<<ÔT­1Ý¸q£õ¶hªijjª¾ªúÿÈKFYoÝº5kÖ,åóâÅæ°°î;(Þ¼y3eú¨±±1!!AÔßÿØ±cõ?KYý§ú'/÷ìÙãp°¾«¦¦¦©©<0e5Çõqß¾æS3¿ÚÏÚ»÷g¥¬úÚk¯s,XàååõÆoØ/Ë·vY/lV3k/Þ²5óóó×¬YS[[ëçç§¦,Y²¤¹¹YxþùçGikMQQQddä3gØtÀ=³:äè7nTDËÕ«WÇÆÆ6ÌÃÃÃjª¡OÕÝ3fèb³²²8õeef]îÐÍkîÝ½·Ú©jFEEM>ÝËËËÕï:³mfVÊ¡¥ºº:)))<<;|øð¿åO>ILLTS###Íkè·´´>|Ê)&®Z£èÆÄÄh=[3ëÉ'ÛÛÛ)+ Ý­*++3/Ú°nÝ:%¶BN%Sõóó³Kc¨²º·ªÜjTÕ$''×××³ÁÍ7[ïÉê0³èã¡C(+7uQíjÔÜÜÜ¦¦¦¹sçÚßqEÌ+vÖTS£íÂôQû<ªÁWe5Ó²Ú<ÞºemkkS87lØÐ¿)eÅÕÃkTÍËËSãââ&Kç§ÚjÍ©3:ïÕV]¯)­wòóóUûÅ5<<µ¯T í/däpìW«®PAõóóÓ¬¸²¡RÖÚÚÚ;du¨¸Í,æ×2eÅ vîÜ¹éÓ§½üòËÖQ®?ùÉO¬d¹VqÕÓTûóSPÖÿ(Øá °RúñÇ3³b8~ü¸:vìØùóç1bôèÑ&®*«²ªÑ344´ FØ7ß|sÅZ ©eíÁÙLCGTTT||üw·«©«&×ï:çææjáðáÃ«V­zÿý÷õ§ËPVÊ!Ç¼R`JJuoöððhhhøÎv3Ì")·ÅÅÅ4 ¬ÝÕÞÞ®.Z´È¹¬·nÝ¢¬x (æy,úa?Å·W6lUJ-hr÷òòRõ]æ%ýµOsªµ1g0eÊûYiiix   ôôôwßWíÏµêºhýZÆûÌ3ÏhN]±bs*@Yû§¬Æ¢¨¨Hi,,,<zô¨Z¨Oýýý^×ÔÙ5lÚ´ÉÝÝþüùúöãÇ?õÔSiPVÊ!DSiVV*øê«¯j*õóóÓðªõ?ùÉO^í5û%Ý.Êjâª<»wÐBHHM(+eÅ£ j¬TYÓÒÒÖ­[W__¯O>üw÷waaaöKfggtmû(+eÅöÕW_©©K.øáËËË£¢¢JJJßyç	&¤¤¤è2Û·oÏÈÈÐÀÚÅéÁjêªU«"##i*@YïRY[[[)+îÏ?ÿ¼Êú§?ýIáÌÍÍ=sæºøá¾üòËîÝ«)6+++33SõíìUõ·mÞ»FW¥¹w(k?U; ízÌ9Àº_ol-oØ°AzyyQVÜV¬Xáîîþè£*>>>ÃûÝï~ðØc3føðáæÑÖ®9U®i*ïæPÖ~+ëÍ7yE~<(5ª¾õÖ[uuuJiyy¹îö)Û·oÐ§¼cSËÊÊ"##i*)+ïuûÜñãÇyæÑ£G;V³i``àwGq5³VUU­Y³ÆÍÍíñÇ×«ùõßÿýß»¸*c¿4eÅSYY©^ÆÇÇ¿ÿþû¹¹¹X½½½ÍÕÐÐÐ%KhNñÅÇóé§vÑT58..¦¸eÕ ýeÅ=TQQ¡fddøøø¤¥¥Yë¿üòKý¾þúëæÓS§NÍ=[kæÌÓÅ»ñx*åËÛ×|ÿý÷wGSSÓ¼yóF¹páÂ­[·º»»k0­««³. /iýâ/¾ë8Ié:ujgsêºuëx<ÀÝ.kuuõ¬æLà-[¶¨¬'O¶Þ]÷÷µFÿ$eÅ@;zôèÃ?ìííýÿð©©©4=<<>úè£äädë2óçÏ÷óóÓe]e5''ÇeSKKKSÜ²Þ¼yÓþ¶ç7oÎÌÌäh0î2åpÌ1¾¾¾§yaý7ÞxCã©yý^óÔýñtq%4À=.«óÉ8p ©©IW®¬¸kJJJÃøÃ"##ÕQÅuøðáÛ¶m[½zõèÑ£UYõµ¦êOwÆÙ¤îYÝ:åÝ»wk¹¡¡²âî5jþäRww÷§zjæÌãÇõÕWµÆÇÇG_:uªóK:4µ¦¦	àÞõæÍÚ+`ª¬¬´âêååuöìYÊ¥êíáV&srrüq__ßGTMÛ¾»ËïâØ/û±¬×®]Ûºuk```mm­Y³sçNíãN8¡å+W®êSMº$eÅyáÑaÃ?¶¸¸¸ØØX-ë/ÓåjS333i*û¥¬W¯^õ÷÷7/pXWW§=ÚÅµ«úüóÏíkkkcfEß©æÍÉ¿ôÐC¹»»O0AÜVe¿þúë.Ê±_÷ãÌ*ªiuuõÞ½µ«Ò^O#¬T¥WËk×®¥¬èßÿþ÷DSRRüüüV­Zeÿªê¨¬?^¥T2õ«_M6Mxê«ÃõlÜ¸Ñ4555¦¸ËªýÔ®]»LYÍCª«Y¯²8qBo¾ù&eE/«þþþYYYÍÍÍuuuªcYYuâââ1cÆ(®o½õÖöíÛõ-ï¼ófV]ÌºÌ=TSÓT-³aÜ¿e5£jTµÕÐ¾Ï¬õ|ðeE÷i T&ö³ÅÄÄäää·ÏÎÎÖÌj2©ùÕºðûï¿¦k``àØ±cµàååõÏÿüÏö¦j¢­ªªbÛxfVë°yÖÍ¼sæÌ1çð¬ôTrr²&ÎÈÈH÷±±±ÖdNô>|¸uùsçÎébëÖ­ÓúªEEEZcÞxÇS<¨eå½nÐ/ÔBYYYX#""/_®4Z¯A¨áµ¤¤Ä¶y¹¥éÓ§¯X±bæÌÊjxx8Ç~²8qbïÞ½Öã¯;vì ¬èÒÏÏoâÄéééZì 	Õ<1F]½zµ¾TPPàð6m9räC=¤¿=¾4À`(«ýe´`U²¢;.?eõW¿úÕ²eË<==4iõññyäG222c=ªo)//ommexàËZ]]­ýÚ¡CÌ§gÏ5'7QVtÇ¯ýkýÁ¸»»§¤¤dffÆÄÄ¬_¿Þ<ÔúÄO¼öÚk#Fxùå«ªªªi?ï÷«¯¾¢©IYÍSWÎçÛPÖÁ§¾¾Þµ¤4::Z)Õ§/NKKÐ_¦ÕâââÃw6§òx*ÁVVóºÁ-r[í)+hô,)))--ë­·üýýzè¡	&xyyiyæÌæEÍÀúÈ#8»*nýjNeTe½qãy±ë-Ð­·=_¿~ý)SÌ¯Ç³¿+e²._¾üÌ3Ï?þoÿöoö³©ú|ùåÂÃÃ?üðÃqãÆ×,¬¬¬tp333===5§êoc¿aYUÍÓ§O÷g5N4É|IûDjêªÝ¥LYÉêO<¡Ùô¹ç;w®···*«Obbbþå_þ%44täÈÉÉÉjç°aÃtûj5c¿sYCûî»ïåÍ7ëÓë×¯s4x(¨©©)//×ÇÎæÈªª*???ýI¤¥¥i<-((>zddä1cÚ~ûÛß*¨æ%õ³²²ÌUÙçT`hõüùóö'Ûh9..3Â$úôÓOGDDÌ9S#iBBÂ3g.£5AAAJéÃ?¬O4ª<òÈ#.]úúë¯¸»»«¬_ÍwåææÒTC·¬Jé/¾hÿ4))²z_||¼yúéßÿýßkÁar-))ÉËËSGÍË>>|xüøñÁÁÁÞÞÞÊª¾eäÈjªFØýû÷[sjTTTgo]¼¬PJ/`/«vuPÒ4©4jZ5çèÆÅÅEFFj]¿~@@À	ª««íÏÏÏ/---((xî¹ç¬¡vøðáºUÓQ£F)ÉjêÆsrrLSõ-£`uÛ¶mÚ½N>­OËÊÊz÷2LÇÓNÖËËK3jMYï¹£GÖÕÕÈi!)))&&&55Õ»FÏï:ÞT|É%ZÿÖ[oEGG«3knnî©S§BCCþóûùù=ñÄ¾üòKýÍÍÍS³³³õ%`HõâÅÁÁÁÎo³páÂ^Ö¦M´°víÚ9sæháÿ:yõÕW)ëÝ¡HARs¤÷÷¿ÿ½>®ZµÊdÏËõ÷÷×ðj.¯Ü¾óÎ;Zã0³ÇYÕË=öhºUz5°úøø|úé§úªæTóx*M@YûvÖfö½uëVDDÄ?üàæÊôéÓ¹-ÚW_eFÒ©S§j¹¢¢BåÌ3ÍW5eª'Oöõõ;w®É¡[GåüÒ¾V8õÕË¯ýkõ¸©©©  9e@^^^Ë¼ôÒKÌ¬­²²ÒÝÝ]=ö3Ï<£eÉÉÉiiiÖe4~ñÅúÒÄÆììlM«[·níìjëëëÍ´<²Þ¦LM¯¼òJ_®DûekÙÇÇÇYïm|ÝÁÁÁ¡¡¡qqqÅÅÅ*hFFÆ¸qã¬Ë(¥g5tjAÓ­.³jÕª®¯YÃ.s*ÊÚ-æ¬¥'NØW~ÿý÷=ºÚtZËõØ´inÊá´ÁÎ'|RÅ­©©1ÓèöÐCÍ5+&&fôèÑUUU]3gÌ«ÒTµÓw³^.¸¢¢Bûbí4µ`õ`û¶vÇ¼yó6lØ ììÕü)ëQ)5M*Ã34­nÛ¶Mkt+5J¡Urrr,Y¢è¦¥¥ýñÜ¸qãþýû­óºSõ²fÍ²ºÖÖÖf-ïØ±Ã¼áÙ³gûòÒäáá~äÈÊz×¨væ½Q·nÝêíí­[300pæÌ¡¡¡J¬§§§uâFÏ²²²íÛ·;©äâââ±Ë¦@Y»û7æýY5kÐËSÖ¥Xª¬ê«fÊ±cÇ>ýôÓªidd¤Ñ_þòÃ:Ü±£Í©4eíE©¦yyy>>>¾¾¾´<mÚ4Êú@(,,ÌÈÈðòò2oª"j;kA·¬V*«ßT¥9eí%S£:X/p@YSÍ[?õÔSVPÕW°ãÆÓBwV²öµ¬PG:¤ñÉ'Ë:/ÊOYn,OOÏ¦¦¦ï:ZÈ¸¸¸ÀÀÀýû÷w³©ÃÏÏÏ§©(k/Ï6#©(ë­°°P7ÖË/¿<fÌßþö·öìùÅ/~¡5?üpIIIccc7çÔ¥KÒTµe½zõªæ+±õ6gÎÝX)))©©©~~~£G.((pwwîæZTTDSPÖ~;É^ÖØØØZNYïOo½õÖc=¦?ª£n,óÀ­­­YYYO?ý´9+­ë¦åçç;¿Õ9PÖ>Õ¼9ëÅµ®²¶··/_¾×®]KYïCO>ù¤··wNNÎ+¢¢¢4ªº½BCCãââÌÙL]4s`Ëj§²FGGeó®5õ~£zxx(æÅ§N3bÄmUsV°6ÌááUû±_Í²]?øµßÊªrvvö¾eí¦¦¦ÀÀ@5--íË/¿¬¯¯8qâ¨Q£ÔÔsçÎËh¥FXë5µPZZªq9îAYïÊÚ;äÒ¥Kzè!Ýû.++ÓØ£Rz«¬ß~û­Iojjjqq±ÕTÍµfN5ÏÉPVüùõSRRüüü¦Oîïï¯¾ýöÛjªª¹qãÆgyF¹TS5æææ¶´´¦êS5s²â/äççkNõôô=z´¯¯¯&WÕ«W¯X±bòäÉísÏ=×ØØXSSsîÜ9kN-,,äØ/PV¸àíím^­ðÃ?Ô²:jÔ(ónqúT_=zô¨uìWfNÊN©¦*¨6Z@@ÀìÙ³ÿøÇ?N2%,,lÄ~ú©iªÒ[TTT__ÏFÊ×ÕN¡3gÎ,((PS'L°eËó²À111K,±ÎQbNÊ;xíµ×<<<Q©S§©µ*ëßüÍß?Þ²DS²âÎþó?ÿsØ°aæ]Wyä___Í¯æ-Wy_ ¬µBCC5ªîÙ³ÇßßäÈOÍ-M8ÇS²RÖ(**ÒsîÜ¹ÈÈH·Û~úÓ²²RÖÉÈÈX¼xqHH9XA4i§§'G²RÖ3ïg7fÌ%¶°°²öÌÆMM"""ÌëBhlµ^j@Y)ëíÙ³'55Õtô¥^bÅûï¿¥5ö(+eíOOÏììì'|ÒËËK+«ªªÊÊÊÜÝÝçÏÏÊJYï@ùLJJÒH:cÆºº:­QMõé¬Ë|ýõ×Z³cÇ6PVÊÚ)sì×4µ¦¦Æþ%÷#G7oÞ°aÃ¼½½Ùb@Y)«kÕÕÕ.*ß~û­y¨Õ¢ÊÖÖÖ²Ý²RVsjrr²b©iuÿþý./ìëëéÒ¥òòò,^¼Xã7ø?²þ/uÔûÕGõµKjB­¬¬´¯0aBDDÿ²RÖ?;|øpff¦§§gJJJ×M5TßO?ýtîÜ¹ú®%K477ÿÕ_ýÕ¨Q£ø?u¨µººÚ4Õåã©]Ì¬¾¾¾eeeUUUêkdd¤×SO=Åÿ ¬C·¬Ö±ß5õ»`òññÑ7¯Rq5~ûí·üÊ:DËZPP 9UeÕÌÚ£oljj5jTbbbFFuz°¿¿ÿÃ?Ì ¬C·¬X»óxª³ììì'®ZµÊ|ÚÚÚ¬OøÇY,<<ü/¾kii1kÊËËãããÓÓÓÙ8@Y)kjNÍÈÈHIIÑà[__ÿ_üÂÛÛÛ¼ê!²RÖIKK[·nÖ%K(®			cÇ]¸p![(+eíÿøÿðôô1bDllìç^PPÙÔÔÄÊJYL)5/¬¸³CBBÙ2@Y)ko(¥V­Oþy­QnÙ2@Y)k/Vû²×ñãÇ³q²RÖýõ×ÝÝÝV=²öXmm­ó±_­:u*(+eíM¨Jé;ï¼£å¯¿þÚËËKS¬Ãñae¥¬=ävÇ;Ø&@Y)k_3ªe¥¬ÊJY²>Çòððpwwøâ/Ø&@Y)k/;wÎÛÛ;>>¾¡¡¡¥¥eþüùêëºuëØ2@Y)koL:uìØ±ö5«¿¿?[(+eíGyäý÷ß·¯ÑäêüzLÊJY»Eëo¼a_süøqÊ²öRnn®Ï¹sç¬5=öXTT[(+eí¥øøxOOÏ3gÎ??00pÄ[Ù,@Y)kï­X±âgyòÉ'×ææf6PVÊÚÕÕÕ«V­Z³fÍÑ£GÙ@Y)kïµ´´deeEEEåååùùùñLV ¬µ÷òóóSSSÕWói@@ÀþýûÙ2@Y<räHbb¢WBBÂ±cÇ(kwhZUMík:°e²þm~¾-[¶ÄÆÆja©S§¦§§s[Z4¡¶¶¶Ú×æåå±e²þ__ß~øaM´Ó§Oç¶´¤¤¤Û×dff®Y³-õÕÖÖ.£ÁÝ±gÏ   ªª*-kx]ºti\ÜåËÙ2@Yÿ¿ë×¯geeÝ¸q²vSEEETTTxx¸|êÔ)¶	Ý²ºÝf>½téÒ®Â¹Á=ÕÜÜÌ¨õ/TWWO6íêÕ«<ë@YûADDeíÍ©QQQÉÉÉæWeå"z£¥¥%)))33óÌ3útãÆ¡¡¡Ä(+eí¥uëÖ¥¤¤ØÏZZZªÉ-²öF~~¾Ã««²~~~l ¬µ7;Ø×466³e²RÖÞ¨©©	µ?5777''-²öÒ+WM®¥¥¥3fÌHHHàmÏ²RÖ¾N®*k^^Þ5kÈ*PVÊ ¬Êe¥¬ÊJY²@Y ¬@Y)+ÊJY²(+e²RVe¥¬ÊJY ¬PVÊ ¬Êe¥¬ÊJY²@Y ¬@Y)+ÊJY²(+e²RVe¥¬ÊJY ¬PVÊ ¬Êe¥¬ÊJY²@Y ¬@Y)+ÊJY²(+e²RVe¥¬ÊJY ¬PVÊ ¬oïÞ½nnn@YûA[[[bb"ePÖþ±|ùò+WZeã$***==Û@YïìÂIIIííí@YûAffæÁÿüq4@YÇí6û²µ²(kÿä@Y)+²òJPVnKe¥¬ÊJY ¬PVÊ ¬@Y)+²(+ePVÊe²RVe½¼ùæ#FÆm&L;v,ÛáÐ×ög;ÜãÇ7nÛáîôÑG###Ùv~~~|YÏ?ÿ`óË_þÒÃÃípOxzzjû³î¾©S§ÆÆÆ²î¾ââb77·>úMaù×ý×ÖÖÖ»¬pðÕW_yyy±î	û·c;Üùùùiiil»ï¿þë¿TÖÿþïÿfSÜe¥¬ ¬²²RVÊÊJYAY)+(+e¥¬ ¬ ¬²øÃüq¶Ã=ñÄOTUU±î¾eË½úê«l»¯¡¡!&&æþçØÊeõAuìØ±ððp//¯øøø°AÎ#Gµ©´Ù]n|n³wï^77·ÎþìÙòáæÍ,ðññ7n[²YYY6mÒÂÚµkçÌÃ8ÑÑÑæuA·lÙërãss¶¶6Ý­±ÊÊ¿;Þï½>ø ½½]YbËSÖ¡"88X÷Z¸uëVDDäîðõõu¹ñ¹9ÈòåËW®-whmhhèzÃ§¬ýi¬<¥õî¨­­]¸p¡ËÏÍ1.¤Ý·UV¶ü]Û½¬^½Z÷#5°?~-OYkÙÇÇ2Ð®_¿uãÆc dff<xðÏ;©ÛeeËßµÝKYYN>­;7lyÊ:TÜºuëÇC1Zf¨K.-X°àÊ+m|nÙ7ý%¶üÝÜ½8Ì£lyÊ:$Ì7oÃZÐGÍRlS]]=mÚ´«W¯v±ñ¹9:±lù»)??ëÖ­Z8yòdJJ[²óðð?rädàDDD8LNÎãî-wv-33SÓjRRÒÙ³gÙòÊeÊe²Êe²Êe²¸§ÿÝÜ&MÕ¡¢¢bóæÍZóÍ7ßtqyó&]0o9¾xñbóé7ô©yÛNTöîÝÙAUí?®æ-½ìÜµkµ&..NéÍî°sçÎ;vè/¾ø¢µæ£>Òk×®YßrèÐ!­±ÞÆÄ%Û ¬À`sóæMûXYWW§µµµf¡­­ÍTP6»pá>½xñ¢>N2Å¬´¿ã¬bÖ(À¦âÖüÂ/hù³Ï>ÓòÚµk¹-ÊW®*Ü¥KôñäÉ»wïÖBKKCYÃÃÃ5¾úûûkBÕFÒøøx3°nÚ´IkN8á0õê:­_¿~Ý~(+0xQUÝºu«/UAs÷ÚµkeµfPóâæè±pÍûZiíþøãµRÑÕ²ñ'|¢5¦ÄZØ¶m7@YÁãêÕ«-Rá¶lÙbÒ¨SµÓ¦Lç²êÓFDDºË-ÓÊÓ§OÍ3Gö+¿uëÖ¬Y³Ìdó½Öõp+TvíÚeRdg]»v­.Êº÷nsIåS_5ÇÍC¤Ö¬æ8ðùóçÂ©	X÷íÛg»U­ÓÌÈËMPV`P1ÌÊÊ2ñkll4	T,5¶jáÂÖj.cÚÙÁ£dËöövÍå­Øyf5_@YAÂ<ÔzäÈ~½¼åË7Îú´¥¥ÅúÕç«ºrå9m¸­­Í9Ì¬eÀÿ[7·³¨­[·úûû+Öe¬PõU:oÞ<+öLFGG»ü'YÊ	æÀokk«8kæQ­WYMó4Zµ³Ì<züøqÔÅÌ!_¬]Ä ¬ÀàgÎN²sôùçkMSSÓ·Qjrh?ºíÚ5³R5µÊªâjòÜÓõäÉ]T ¬ÖÞ¼ySE¯®®¾ãwUVV1WEÔåÇn¾ÔÒÒ¢/:uóæÍæuyµ  À¼Ü·@Y!'66vÓ¦MÖ§æ¥Þï=kÍõë×ÆVs0yÃ¦e²@Ye²@Y ¬²@Y ¬²@Y ¬²@Y ¬PVÐ+ÿÁKlçuIEND®B`


反趋势正态 Q-Q 图


óN?÷5×9  à9ø|üqr×ùu]×ëz®_OÀä	`@²@²@²¬¬¬¬ddd$+$+¦TmmíÃÍÓCyùøñãò×þþþq,UëÖ­óÚ~æÌ³gÏZÛ»ººÌpkkkÆ°¬È·8qÂó÷íÛ<00ðäÑ£Gòöçdâ477Oá`@@RR­eéÒ¥cyídä.X?~ìù%%%2túk£ÌyºyófÏ·Ù6,Ëû÷ï×æççôÅ=*#ôöö>õ·oØ°aÂî/¾øæoÆ>~UUÈûôª¶q,Y"S&kºËfåC²bÆº|ù²üïÙ³Ç¬Ö¯_?JËwîÜ1#'$$×J»¬|½¶·µµIvÊ@MMiÿôÓO¥EW^2 §Yîv»åON§³»»Ûë´hÌ|õÕWÒ@ZµjÕÍ7SYWG¿ÖÕÕ²Î%.÷ËKd$sm-G×éò¨AÑÑÑ¶7¼télKY7PäÝäé·ß~ëõ'Û6ÂÃÃ/^<®ÍÏà9GdÙ8°°°åËëÏéüÖ0y*7Ò§ôõõÉøÛ·oÃÂl±ÉÈ¹¹¹2,[$^éÜ_±bÅè¿Åº1têÔ)yÚÓÓãuûÉ=ãd­+QÁk²Mo.**òúæRµxmß½·[Ö5àäOþ¹uý(=úÊHRAFHLLô¬4ð¬+÷8ÚaÒ¾qãFë[éªVRß¶^ÆóçÏëê[þdË¡q%ï9±d÷¿>Ú´ÈtÊÊJÏå³d|YÝËféuÏ~/êïâ^ëÈ¼¼<yÔ¯ír¹Æ¸´¬2#¬Ýú:YLn<É°×d5kÖèTÌ2uø·ö+$³mXX?WJpiñÚÇ`2lJØ~¼¡Ôå2fNNÎèK5YÍo7Å½|sm·mdÅ¡Å¬V®nÝ:]7øá:°iÓ&ëÈ²¾v	!=Ý·»Ö®ûô­tüo¾ùfõ0³lØ°Á¶]o:¥=>>^ÆÑ$Ð7¤áÛº2--mì[¦úÑÙ3vîÜi+ølyï5ídÙ:@²Jû'|+(¿Z?ûì3í#5ßJË,kåªNò#88XT)ø¼NÝPö+W®è¬75«MÏÙ1®dÕnÛæBJJäúH/_¡¿®¸¸X0í0ë×¯ë¶¦¬mjÛ& »£`ë¤±nAÞ¸qÃs²¼úCÆÒ_mU·öd¶v³^ÿY@²bæÐD×µ¶JNµ¸ÿ¾×Õ¶²ÐU©Muÿå­[·$Ëõ£½Ö²^ëZkhé°µ¶ai¹wï^ò0kI¡mû´¤f³æ¥­5kkÔdmkMVÙX²dIPPÓ£$«uJÊøún2­¬ãØÊkynll(5=2PQQáõlÉªóEÊqý«L@¯á'ãzu·½ö£Ê°×qtÑ2Ë©ótiGÝb°½æüéÉðNSÙ°7oJ£d§¹×¨öÐXÉGrxù!òtx¤Ø6ÉªSX¿ww·n$Y;HV3¹Øë#ýçv|¬O~Ø3ª;Ìü°O×¦ãTV¶2·  @Ç7oés[µjÖ8yòÃ¶ÖÖÖQÊ©bbbÌ×µ°T-Öe¤dÕ«i¾×ømmmÞ¾aÊhÏò×³h4Õ¤ÚZCW;tSÃzä¢öho°¾ÿÖ­[õX-yÔYàÙIëYÚui®¹¢ß#è¤Õ|Æ©©©º·ÒlÏ.­núÉW=KcUû]=ÈHýÞ²©4ÒÖnÃ´#ôdY SFæÈòåËÍ"J²¬ YO¿fÃÚµkuôÔdÕjCWôæQ¶âÍDkÞ2×ìi©fÕãZuhoo¯|ÄûÃ<Â´m«Éª+G¡««Kê]ëVcIV=z¬²-Ye]¯Aõ`L´±'kHH¤¦lsJÑk²j'yJJJ­$×`ÖieF|ÕÃ¼KJJ´¬Ô¸õúõV¯^­ÃÚ )o"Á&Ã^KXÑº#@§nyöëºº:Ý®#è±T27µ7UªðlÚ´ÉlXI!(púæÖßüUÂ[æ»Y<ÌÑ^âºáyô»nXçø%KÌ°|O¯½ÁOw¸hÿ¶m¾¬$+fxo°Ô"^Ïð<pFW£ºvÓcC¬ÇÐz]YÈ»ñ5Ïô8mÜBÄ³pj"Ùë 2æÊ+G¯Yõ'h äÁSÕÚ;JÍ*OÍ£ÿ£'«Tç¶ý©ú¦ÎóLVC©þ­uÖúºMãÙ·iÝzÐí)¬%ç%o´pÔÃFIÖ§öK,É¯)¨1µy¤ÓY'¦ùòMß©ÑÖÐkÿ­öËÆ~|öÈ@lll||¼µÌ&!!AÓNõ`4Ù|1?PçaçRËo·;mÛ ´-`&YuR«gÝÂé¬M²¬±´ÑlÖÕi·­2ô¸YãËêÆ¶Öse!+8­3ôàUÝó¤Ç¤ÈÚ{eóßóL²>µ7ø©E¹çi^UOsqRÕk¥2ÆÕ|gí!0AÖ=XÉT]zäêúõëÍæÑ=Ì®AóùáZ«Ywk²NØÎhK²JÈI$%%Yb%$Àtwéû5§Òù999º¢ËS0;Ve!±Uºú©¡###%J/ åá¯f×¦,ê¶sclgÙz]NL¶&·TùcLVý9º£A~©Ö»òý5¡õÂë9Ù Yá÷îÝ»§ïO~8ô###Ãl§£-LËå²u£IiñLV³JÒ°ÑLÕÕw]]®Ü=O£Í:dë­·l²Fv:¶dÕ5þÄzu±-YÃÂÂô¸*S`=Y=#Ò©mÄ¶öf[ÏKyâ±?ØkÍjO¿×OÆ[³ÊO§f9W7ÛU#Ì>YòRÏ1G]Nl¤)ÖeÂ­­­òæ¦/×ë®V¯n·yö3k:zÎ ÑÕ¶¢»Ã5µdÅÌ$«<s8¨öÔi#[÷²nÒrÖv°l¿[U;»¬[ýº²ÐU]×?±Ï*I#I 	!ëÝk«ÇÅxA1ÉªVzÌKAAÁHWtK²~æJ¶dÕ«ºUþ¤»]'¬ZÌY¯¹aUgn477ËnxMV¯5«tñ&«nNiï´v_.K¶wÐ¦¿ÎÏ*ÓJûuõ'tttè±Á¦Ã_f×]¶kw4ÁuÙ³î°cÇË¦céÚÇ#ïìÙy@²¬ÕªéÈ5«	YmézVcCWyÚá&v¦YÕºWïÉï5!«Eë*IWa¨&YuG®Ì6¾Ìâããu¬U~YÃj×ßØ/G§jºWÉõ¬k!hú]mO,»9Gº²-Y¥¨2?«çººWÛv|©$«Â¡¡¡Bë×¯GírY9®Õa;dµ£1h]´d]~´ËWÝÕë~NZ¬z®l9I|ºÙ@±îGÐåd¤ã~zSÏÔM,Y5¡õÊ¶íTdÅÓÿsÏÃe%«=´ß|óuCÛ$«TºÓòÔ3Y­+8íï¼ÑTµ¤»«z¬âµ£LtÒ*VA¯$k%ùYêeö4nãââdÍ;kê^ÞJ×Îë5ëuåJ¨ëzÐa¶dõ$[ZôKÅfÚ/ !$ùºdÉqÏj®qØÕÕ%7ºT"ÍöIVÝqäý%udBiL´±'«Ù`GÖnÌ5kÖè_åÓÍU«BBBÌþ]-ËÌwÓãõ¾é9÷¼XµfÍÉÉyòÃ%t¾dõÜýojn+ÝMnMº3uôT¶Í Y<d¦è	Ä¶c¾¼^êY,e×	HVÌ(ZâÖÎFOçÐ×vt=¼SÖ)¶ó=ô¬ëÅ$dÕl-ÅLÞc5DïÖ­[ßÿý¤¤$)étÏ¥òzùøQÊÖ®àµÓ5%%e¼%G4RÍ7mséZÛ&¥ç	#t£¥ZÛ^"É*A¥èÊLÑÐ2û×u§Ù=l» Òxéi3J3ù¶ç¨X¿9DH7V¤·NÙÚeO°þë;xe]öÇ¤Î4G´Ç)+yiv÷Ú6[mWä×«ZOòñzÑJ¬xÉ*[²³¸¸x«Õx®hL¤i©må¨»åF©E¹hÃëx°öÆÛèÑ³¶lÊZúKÙêt:m×6Lõ<gtz~YNdÃÎvqÍ§Ò_4Ê·½½½WuÖýã½Ó([i#ÝÜÉ6Î(WÁÉHVHVHVHV@²@²@²¬¬¬¬ddüî& àO>±¶ôöö®óS§NÕÕÕÉþù(oÕßß?oÞ¼µk×®vöìYyIqq±u7o®fÞYa_^n¾		ïsïÞ=k»ÍïØ¿Ñ3g¬á!1iMÖÚÚZÛø:¹téÒ1~ÊÃåÝRRR¬ÉZXXhghhèñãÇ:,2BjjêHoòR¾¿¿ÿþû#0vcÿHV`D&)5Kbbb$öLø8p@Ï?ïùÂ	$«õäiffæ(å²ÖÁW®=Yõk?²ý YÝ¸q#--M%Kº»»G¯Y%Ym5k~~þHc8qBjè¸¸¸ìß¿_³««kd		¬dË@²O©Ï$6äq÷îÝ÷ïß[·nÙuhhhé0Óu$áª"µ'y0·=zô¨µåôéÓýýý¶ÑÚÚÚL´dÓéÔöë×¯K»ËåòÎãÇËpVVÖ(É:ö¯cüHVà)6nÜhÔ¤¤$ÏÕô«fmllÌ¶ÞV®ñññ:¦äååJYß_KI¯Iév»½fû@²O!ÕªV®O~8&HY½7XóXkA4eüèèèäädsP®)åÁùí·ß/_¾Ö1~$+07oJrìÛ·O­é$îg·¸Òda)eà³Ï>Ó·ùÖ[oy¾ÜÚlÆ/¾øB7mÚôì5ëØ¿ÑÀÀÄÆ7=z$û÷ïôdíïï×ðÊò©5ëxUYªÌÀÀ@Ù> YðÿÓ§OÛ/^¬Åå<_ÒÑÑ!íc¿rIhysNLL´Õ©å¦gglccãHÁ©eë5k&+Yú¬wwîÜñÚ>88X\,q"­×t_lEEÅèï¯Gý|ûí·¦E2Uòµ¤¤D+33SþºdÉX-77×3í¬o2ÕÏ÷$+$+ Y Y YÉÉ:;wFGGÇà~üãÿÇü'ëßüÍß¬X±¢ð'ò'^Ïêö³dý¿øïðo½õÉÉJ²HV@²¬¬¬$+d%Y Y YIVÉJ²HVd¬$+d%Y Y YIVÉJ²HVd¬$+d%Y Y YIVÉJ²@²@²¬d¬$+$+É YIVÉJ²@²@²¬ddd%Y$+É YIVHVHV@²¬¬¬$+d%Y$+ÉÉJ²HV@²¬¬¬$+d%Y Y YIVÉJ²HVd¬$+`r455íÚµkïÞ½ííí$+Éx&yyyQQQEEEááá;vì YIVÀ<x0!!¡§§G^»vÍápÔÖÖ¬$+`"ÒÓÓëëë­-ååå¹¹¹$+ÉÄÄÄÎÎNkKCCCZZÉ:Z[[,Y$SÿâÅ$+Ì$R¤Z[JKKóóóIÖ)§ßïèÑ£ñññ$+Ì$çÎÓ§µµµò´££d&!!!òë!,,ìwÞaTQQèp8üýùM²¶µµmØ°AvyøÓ?ýSïYKKK»ÌãÉúàÁ¬¬¬Ò YUooïúõëïÝ»Ç±ÁõY566®²¯¯³n$ë$	° Y$+W¬$+$+$+É YIVHVHV@²¬dd%Y$+É YIVHVHV@²¬¬¬$+d%Y$+ÉÉÉJ²HVd¬$+d%Y YIVÉJ²HVd¬$+$+$+É YIVÉJ²@²¬d¬$+$+$+É YIVÉJ²@²¬d¬$+$+$+É YIVHVHV@²¬dd%Y$+É YIV¦?YkjjHVÉ:	ÉëõO?&Y$ëølÚ´)$$dãÆYµµµRË¬u$>.]Ê~VÉ:iÉºbÅ@²NB²>zô(`Xmm­­7xçÎÒÞÝÝM²HÖq8qâÄàà g]]$kOOÉ Y¬gãô÷÷¬õéÎ=9ÌÖ	üé§j©*)))ìg¬c200ðÔ#HVÉ:i$YóóóIVÉ:qwîÜ;~üø¦M$Y/]ºD²HÖg244$¥ªÄê8@²r¯_²¶µµ­fV:|äÈ)^ed¬cíûµÝÐF¢t÷îÝÔ¬uÒ	&Y$+Éo$ëãÇ%M=ÕëõIVÉ::UÃÃÃõ&&Y$ë3%+½ÁõEOÖcÇýæ7¿Ù³gÛífA¬÷Ýwß½öÚkAAA.kîÜ¹¯¼òÊ¹sçXV$ë9?ú£?2¥êï½7öl*W&«ßùüÒ¥K³fÍ²ÕÚ^ÎâîdÕë1ÀIIIæx`3)O|6Y«ªªÂÃÃm.K>Å0ÝÉúÔû~o°Ô¬/½ô­f=65+à9$ëÌ¸××ý¬·oßfq¬!kDDÇ|.YV¯^í§÷g­ªªwæ|Vo%ëöíÛ­-=âLuL×ÓÃ=*Éº|ùrsôi$Y$ë¶ÞóüÈ#~ÚÀsNVÏÉ?¾§§GîÝ»7á·êëë!Y/t²Óá3gÎÈpWW×Þ§¹¹9!!Á¼ø<|ðÁ$+`Æ&ëÀÀadd¤^éÄ&®_¿>®w[µj¼Ä$ëÿþïÿxóÎ;ï0/3-Yïß¿_SS3oÞ¼¶¶6m9uêÄÞåËeøÞ½ÅÅÅò488XÆolÿÓCVV5+`¦%k___XX^Ý°££CâóÎ;²6444±¾eö³^¸UH666=V²ÐívK	+===¨½2¼oß>@²#O>­Éª»T%]õòåË2°qãF@²>îFRÕ¬ª¢¢BöÏ?ÿd¬ãëÖCv×[»v­Ï7+EHÖñ^7üÚµk×vìØQPP W¯^ed%YLÜ¹sç¢¢¢òóó<(2ÜÐÐÀdÁLHÖË/=Öì%­««#YL5·Ûíp8jkkMËÉ'%ß¾ÍÄß'«õê2`Rd0¥kJJ­155µ¾¾ÿNÖÆÆFIÓææfª'mIÀ:vìXvv¶­1''§ºº?NV½"íÔÆJ²/¦¦¦-[¶ìÝ»÷îÝ»ÎÎÎ¨¨(ëËµ¸½½)MV½aa¡×¸ÍÊÊ"Yx*++Dd=xð`ZZZbbâ÷JzíÚ5¾qãFzzºgøM²>|øP¯Á´fXÖjkk÷ïß¿bÅÝùzñâEëÒIVjUbÕzzL^^^nnîÄÞ­¿¿¿¨¨(""ÂétÊc~~¾­Ldøk²JjvwwëmÏõéÒ¥KõO'N§z	Ã!L²P[Y[¤ÖÅ3¾­¼¤,þ¬AûÉ'èð#Gäéè`SPPP^^nkeÊdý=7oÞ´l#Ã			ÁÀÄªíTÃ/[¶)Õ^°®^½Úú4))É¿õöíÛEEEòï-ß<//¯§§Ån·[þËrrrô°£êêjÃÁ¨ YÏùóç%JoÝºeMÖÌÌL?JVùw¹¹¹---ùùùN§SäîÝ»ú_*³'Oddýÿ?~bêîî§Sz¦©HÖÜaÖù·çØÀ´&ë;w"##O8áyG¹6ø×Õe«Ùvj¹T®,1é®YgÆ½n[ZZ¬-ò4))%ð<uppPÖ?üÐïµ´´4##Ãz2Kày&«îa½|ù²µñÑ£G¾¬n·[ÊÖ´´´ÚÚÚ'O¦§§KÁ:ákHÖ	&kcc£¹´¡dÄjxx¸X¯th½Á/u#kYYYæ°mÛ¶«ç¬CCCf¸®®N¯»týúõ)ºúWÌðdµ]_ï%WYY9u6$Y/D²Jæçç×ÖÖÔá+W¬ul;S]ÃÌÅIVÉ:KJ677K^¹rÅ3Y'ýÂ$+`Æ&kcc£¤2@²HÖgJÖ¾¾¾±3#Y;;;«««8ëðjVkñj5&&&>>¾k4¦¥¥ùE²ºÝîÃ%ùÊx>Éª7»sçK8I²>~üxûöíÒ¸oß>¿HVU	TSª<y2""Âv1a¦)Y­$YãââtXï.çÉ*9ª÷a6¶lÙÂBxÎÉ*uªÔþu¦Û·o;N[ãÁóòòXhÏ9Yýñ.r"**ª³³ÓÚ"Û;vì`¡Lw²Jk^ºtIZý+Y%DMpyy¹Ãáèééa¡Lk²öööJ¶µµYvron3mgÝ&''»Eqø`Zupp°¤¤$<<<''G/pxôèQ½ùyLL¹µõýóYïÞ½ÛÔÔÔÙÙi½:Ó¬?à¬©©1¥ª¼µ<~øáÖúõý÷ßçLu¬X¥N5Ã§N2gµÆÝ»wû]²Þ¾×®]ùùù[¶lioog¹<díëëÓøÕW_ùW²¶´´8GDDÈ`5..NBH«UyÔÆèèh3Â=ü+YN§¬Ö íèè`éLwÍª!zãÆnmmÕÆíÛ·û×ù-Zd;|)//ÏµLk²¡¡!Ó¸sçN?JV©Mm%%%eee,=ç¬Ö¾øâ?JV·ÛníûËU__ÏÒdµîg­««ó<-ÇïÞ»w¯ÃáÐ[ÈIÄ¦¥¥eddpz+`ÊÕó|Öýû÷kWptttUUUaa¡í$9ë¦ºº:11qöìÙR­qtÀt$«$¨gWW>ôèõ¯²ös¥ÉúbÝë¬7o¶vOú6$+àEIV½áçnZ._¾l»¯É YÇwâ®RÏÀY±bÅúõëõTææf@²ãÄ#GhÄ®Y³Æ3wýîºÁ<·d­©©1UIóëeúIVÉúôuíÚµzÃsMÖÛ8~90cU¢´»»[«ªªôi```]])[e ²²RÚïß¿O²HÖÑôöö:NëTínllG=|)99ùìÙ³òôúõë$+dkåúÔd½yó&É YÇ¬mmmÝÝÝ7n		ùôÓO¥%::zÓ¦Maaa7oöÓÞà¯¾ú*>>~þüù/þ÷ÿwÀ´&«<vttÈãéÓ§g@ÍúóÿvLLÌÛo¿=gÎY³fUWW³ô¦5YoÝº%µµµþ¬_ýµ|ç?þØ´,000¥0MÉàv»uXuÞ¼yûöíëéé©¬¬,..Æ?JÖåË[[¾ûî;ùÿõ_ÿÅd5WYaë	¬æ?JÖ7ß|3&&ÆÖ¨¥9`ÊU8pÀv	~s=a¿»RÄo~óY³fIjZ~ýë_ËÏaéL_²>zôH²çÃ?Ô§>§Ò?ùÝ5æÌóòË/ýõ×2üÑGIÐþò¿déLy²-vêÔ)Ã±aÃSÃ$V×­['UUU2,ê_ÉÚÛÛ#*_^þó³è¦©füø±5h%8ðd*q¥ÀLNÖ¾¾¾Ã¤BÝ¾»$knn®^4XÖ­[·¬uÍQK«V­²ýµ¿¿d¬cráÂµÃÌÝX¥xµÞõÈ#z@²£ZÕuéÒ¥O¦É±ÉjÜ¼ySµµµd¬Ï¬zöê3gÌ=Ï­G0ÕÔÔ¬u|±ZYYùx´LÅÝÎIVÀ¬n·[BÔö¶dòßdíììlooÉ¢òdwzµ9BxëÖ­Òg~¬çÎs:ÉÉÉdéLyÍjªRëfÀLW¯^2w;oiiq8,@éHÖi6ÉZTTTRRbm5==@²NDFFF½­]´hdüüüòòrkmjj*d§ÓÙÙÙ©OïÞ½»lÙ²]»v±HÖ	*++x÷Ýw.Ê+¯ÄÄÄTUU±HÖÛ¼yóüùó×­[W]]ìØ1Ë%qË2 Y'¢§§'<<Üt?|´vÅ@²ÛÉ'333mYYYµµµ,Fu"Éak¬%Y$ëDôôôÌ=»½½Ý´ttt_½zÅ@²NDEEÃáØ¸qãÛo¿öÒK/½ñÆ$+d¸ý×3gÎÂÿú¯ÿúßþíßJKK].³,Iu"rss­-999¶KHVu¬-[ÖÑÑam©¯¯÷<²	@²úh²^¼xÑáp-^¼øüùóÏ=Y[ZZ¬-Çó<@²úh²feeUUUÉÀ¾ûÖ®]+V¬X!¹pZHyºdÉýû÷~ðÁöúë¯ût²FFFêMÔcbbd`àààÙ³g;§Åücý¸¹sç¼üòËòÔ	À$¹|:Y¼?¯Þ`ÑÙÙ)qýÓþTÊåùóçË½ÿè4ÃRúB²¦¦¦§---¶ë	HVßMÖjo°?÷du»ÝwïÞµ6æååq¯V$ëºuë*++e@³²²²J¦:N[cII	·øG²677GGG:ÖÖV_èv¹õõÖ*ÖÖ Y¹RÄ8ÔÖÖFDDìÜ¹ó·¿ýmvvö¢EäÓûûûY$ëúé§/½ôTÒ¯½öZBBÂ~ô#Ûå#$+É:Vn·ÛápXoËZ^^.+e+dsçÎ¥¦¦Ú%Y9ñ@²NÄ±cÇ²³³m)))MMM,OuÜ¤6·ÞõêÕ«¡¡¡¶$+É:VùùùË-Ó"µ¡¡!!!aË-,LuâJKK-Z$¥ªÄ*×¬¬>¬eee?ûÙÏÞ|óÍÜÜÛÍÂ Y'îÿø_~ùå÷Þï>;wnWWËÓ©­­MOOOHHÇcÇ1A¬3-Y_yåK.ËÇò4víÚåt:«««¯^½zøðá¨¨(yË[ZZòóó322ä±±±)	dõÝdÍËË³¶üîw¿dyt===¶Ûß655EDD<µû½¢¢ÂáplÛ¶­¡¡¡¬¬LòÍ¬¾¬ÑÑÑ¿ýíom³fÍbyt'O¢ÓÖtúôiÉNLÁóºÇ¡¡¡Ö:Uê×Ù³g_»vIdõÅdýÙÏ~ær¹¬-òæÎËò4Éikpá«¯¾[ZZj+a½æ±´X¯ö$«%ë¥K$_»ººdèYÅâÙiõiíþúë¯gÍuþüy*«D¦¤ìSó8++d@²úî±Á®R¶J ÊZ~Î96lèèè`y»vír8æ¦¹sç¾÷ÞÖ$weÑóX^.LO$«O_)¢½½]ÖéÉÉÉ9992-%¬¬Önß¾Íâ5Yô¬E¥¥¥É@yy¹mÉQ¯y,I,izìØ1ÙÚ±cSÉêÓÉz÷î]Y_ïÝ»WJ¦þùÿ¹¬ÍNgJJ¬ëóóó¹ië¤Xpµåî²eËFÏãúúzi¡l@²ún²VWW[Wñ¢o¼ñFHHM#«ä+Wêt2y%,ôæB²)sòäÉÑçTDDDFFFvvvxx¸¼-$«^Ý°´´ÔZ!IÊZþÆÚÒÙÙ)+tVâN¦°Ä¤L[ÚcôU[ZZ¢¢¢Ì8²Å#.=ÃHV­YÓÒÒ¬A[XXhRkÐbrÉt¶Þ%w$ùùù¶¬ª	dõÑý¬Û¶mÓ(-//íµ×Ì·oß¦fî¤ºmhh°5Î=)dõÅc¯]»¨,ýÁüAhh¨²¥¥¥ÉÉÉIII¯¿þú/~ñ²ìììJfCdÚÉZ[©Y¬>Ö«W¯655I	êÔ©_~ùÿðõ«_ýô§?Á¯¨'G÷ÎÎN´ÔÔT)"§3ÛÛÃÃÃëëëõ©|Éø]»vñÏdõ;ïØ±cÅÿüÏÿ,R¼JÖØ®Ý¯g»eáÌ 9j=@ÚívK5?ÆÕLsçÎ9ÎÄÄÄäädIÙ²²2þó¬þ¬×ÏëèèºKÐÊQQQ)))z72³ÉïµýÌ½÷Mÿ7jµ¥¥åEæHÖ¬ÙÙÙ¶ûrËªNOOtÍ.¶«ÝÎÔdµ]j×®]Ï%Y­Ñ*Ô¯HVNÖ.[¶ì¿ÿû¿ËËËóóóKJJÒÒÒ¿ÞÛçt:­û%oÂÃÃg|·°lLÈt0OÝn·ñÏñÊø2_$P¾>I¹ÕwU¼ûî»RÅÇÇ¾ñÆAAAü±´K-+c9%%ÅzQÿ"ÛÕÕÕ;vì6ÊÈ¯]»ær¹rrrêëë>,±*úó:I§²nÍtvvÊü¢Éê»É*±ôWõWzäÎï~÷;ÃÑÐÐ ERrr²uLI©býôÈásçÎEEEÉ¶Bii©é£Ü@Lwí¯¨¨x'øzÝ¾ù¢%,¬¾¬£¶°Ü¶m^¨VJ·üÇÔ»âHåûúë¯ûãu$$)å'Xtóóó=ïê©©©¶ÆEYï7$«o%«Tr¶°,//×3µ··GGG¾óÎ;¯¾újXX?öWWWÛ*?¹ÚÛídÝ&8|ø°$+×É@²ún²JId»hJJ¶TTT,_¾¼ªªjÇ²Bÿî»ï>øà	Úç¯64z®µG·¾¾^<èyÛµ²²2ëAIÊ_®,e«lH-?-//O[ZZX Y7Ye5.Q*e$MNNÎ²eËôââbºwïÞMJJJNN		Ù¶mÃáx.w_oèr¹$ûKKK322äkHa-ß6==]Ú%>%äçÈ6õUz?Tk×ÑÑáGWH¾víLmùi2å¹]+Õ×U«"	ÐÐP	ªÜÜJPø,((ÈÌÌ²UéûácVedyá4UÉT=)HI'¥ç¯ýki7ûê¶õ»IÊf$þ4UÙDà¾l@²N·¨¨(í2P*'	§¬¬,ý«ÔO]¬Sª§§Çví÷ÃÊJÆÛè)fm;;;[ï*¶KÞÉJ²NÒÒRÉ¡-[¶¼ýöÛRÚs+¥^ÌÏÏÎ/#Å¨íD 3gÎ[ÜòÝäûsø¬Ï¹rúïG?úÑúõë%¤~õÕWGJ/)& ^ÊzØX]]m333sïÞ½ü·ÉêÓù:þüüä'Z/_¬u8Òb;VèûáûçJq¹hXû$~ygíûÍÊÊJOO?ú´ÖzÛ5·Û-ßM>«Éêë>úè£   ½jDjjêµk×¾üòË°°0©M|Jíèt:;::ôiyy¹?¹àÒÝ¥R4ëKFëwã_HV_'õ¢$¥Ä¤Ô.K577WT=?'))Iª[ë«rrr¦â(X=wêwãHVÿ°eIÙ½Ygg§è"füÆÆÆÌÌLÇ0özÉJ²Ú555EEEéqIR­J«Úê.			Ú,±*)+Õê¯~õ+)[§ù@bÉêÊÊÊ$$_þò-24õDÒÒR½hDiEEEKK^Oª[©C¬$«ÊU5::ú£>²^¶WOtéïïOOO[¿~½¹Ð`ûï½÷·û·fç¨¶ÙÙÙRãJoÛ¶sLdAU544]$%%#Í.555sçÎý§ú§ÎÎNiÔúÊN§SÊÜ«Ãäå%%%2BcccjjjFFá$ë¬ß_æ^òROtY¶líºÚ¬µ¬Ô¦zT	Ñ²²2ùÓ»ï¾kú~ø$Ô¤¤¤Ã³|Éúâ&«ºû¶×k2èLüñ«¯¾*l=I×ùóçÛ^µwïÞ¢¢"o Y_ôdëýÙÍ3'55u×®]¦³7''GB×ºVÈ$+¬$ëSôôôDEEÙÊS©_ß~ûmë½hôXbzd%YN÷³U÷³^¾|ÙårIñZ__/*-¹¹¹Á$+ÉútR°¦¥¥i.[¶LÖr¶¸¸8==]r·¢¢Xd©MÏ;GÉJ²HVd¬$+$+$+É YIVÉJ²@²@²¬ddd%Y$+É YIVHV@²¬ddd%Y$+ÉÉÉJ²HV@²¬¬ÌKÉJ²HVd¬$+d%Y YIVÉ:!111$+dÍÍÍ			$+d«V­º~ýº5Y×zp¹ÌKÉ:V$+dªd¥7@²;GÉ Y©Y$+ÉÀ¬@²¬dd%Y$+É YIVHVHV@²¬¬¬$+d%Y$+ÉÀ¬7n;wnÜ¤r¹±±qðN§ó'?ù	ÓÁ_üaL!ÿ/Æt£ÐÐP¿OÖ7oþßÉö_üBõÿÂO|ùå7ofRø7ß|séÒ¥Lñÿðò/¶k×.&ÅXüË¿üK¿'ëTøû¿ÿûåË?èííû¶¶6&¿øË¿üËµk×2üEkk«üÝ½Iñ,HVdÉd%YIV¬ YIV$+Éd%YA²d%YA²Ç_~)ÿù,þÂív/Z´¨³³Iá/þîïþnãÆLñÿùò/ö?ÿó?L¬Þ]¼xÑáp-^¼øüùó,¾¦µµuÉ%2efyeÌD_söìÙþÅ_¾c```ýúõÁÁÁ±±±Ì/urdeeUUUÉÀ¾û8vÑÅÅÅé9=ïu1ÊÐÐlde~ù²­[·~þùç?Xu¹/urDFFÊR%111,¾,$$Äë,c&úíÛ·ïÜ¹Ó$+óËIÚÕÕ5ú*ùE²[PP×aø¶¶¶6xeÌDßqëÖ­¤¤$Yde~ùøpÏ=²Í*ë¥K_$ëä4ÃÁÁÁ,¾éÁYYY>ô:Ë¾#33óÂÿß:åde~ùø°¢¢Bº»»eùE²NjG³(ø ÞÞÞõë×ß»wo¤YÆLô¡UÉïc~ùþÐV2¿HÖI°nÝºÊÊJG©X|MccãÊ+ûúúFeÌDßXæï+((¨©©+W®$''3¿HÖÉÑÜÜèp8Z[[Y|MLL­òeÌD_NVæ/»ÿ~ff¦T«III×¯_g~¬¬¬ddd$+$+$+$+ Y Y Yøê¿q@ÀÒ¥K³ÕÖÖ9rDZ¾ýöÛQÆ×ÛBïa¾iÓ&úðáCyªwô@²3ÊÙ³g3IJÚ]ºtIõn_Ö<ú´iIHHèÍvêÔ©ºº:aõêÕ¦å/¾û÷ï477K¹Ã¾ç'þÉÌ4Ö²²££CÛÚÚt`hhHSPXG»uë<½sç<®X±B­7¾Õ*¶¸¸ØÖ"¬)nêàU«VÉðdxß¾Ìdf÷îé@WW$¯<^¹råÌ32àv»mÉêp8¤0Õ|U¤$]¼x±¬UUUÒrùòe[Õ+ïiRüÁÖ÷@²3ª¯555ÁÁÁZ_Jjïýû÷mÉjjP½'¹ök«·¼ÖÝ´ÖwïÞ-º2¬aüÕW_I&±?~¬ÀÌÑ××WXX(	wôèQF©8%íd@ªLÏd§.ÑNÝÏ>ûL»»»£££×®]+Ö7fv ëk333m!-ïÃHV`F9ú´9¤H÷³îÛ·O6lØ ÉzæÌSâSþªýÆºÔìdÕ~à7oÚS*`yüæoL±+¥ª9ÜIK^f@²3fdVVß74%,¥l[·nTÇÑý².ëÔ0=FI"##%,?~,¯aßÄ5«þ	ÉÌº«µµµU÷ïßooûöí±±±æ©Ûí6/ÑX­­­õ|«÷îéaÃCCCñIÍ¬ÀðPTT¤K,°°0	N§ÓiÆ1;På¯òtÝºu& ­1çõ#¨Yx!hÇo¿?>>>^ëQidÕÌêÓìjÕ£´½té<ÊhÚå+ë(áMÍ¬ÀÌ§G'Y9:tè´ôôô<ùáCkïîýû÷µQÒÔ$«$®´H<·f½råÊ(©¬ü¹áÀÀ$ââÅúª'Nh+(ãÇÆÆ¦¥¥éÜn·üiÞ¼yÉzäÈ½¢o«YôrÌd^8ñññUUUæ©^ªiëÖ­¦åÁ¶²U;+++)L¬¬¬¬ddd$+$+$+ Y Y Y YÀü¿[à5z(IEND®B`


÷]çÑ£G»HÖ¨¨¨Z³ýªHV ÓQ¹¥Ç·Þzëüùój:uÊ/Y¯2¦Óuìõz®¶+Ôz»3ìúõëÝC¶mÛÖÖÖæ7Z½Ó#­pMJJ²á'NÐðäääÀàüðÃÕ.((è"Y´óµ_Éteþüùî@ÍÌÌ¬Y~ãÕ¬µµµ3;°rpôèÑ6¦³fÍrÞ¨u¾A2??¿µµõÆkÖîU$+pªV­r½öÕ1AÖËzã½ÁÇVª¡îbü¬¬,ç¨`§ Öóã?7nr÷ÆkÖ~U$+£²¤¼¼îÂ´÷³ê£Wj«DVãõ×_·÷ó'|»»Öo¾ù¦Ú.¼ñµ§_ÉqùòeeÆÉ'/]º¤Æ»ï¾ÛçÉÚÖÖfá§Êòº`OUi§2×ãñhûàÆµG_ÉO²mÛ¶ùµÓÒÒ¬bÛ³gOà[4Üéëþ	Kh¸Úééé]½Áµµµ§­Ó¦M»ñdíÑW@²þÎ9txû-ªh`ûb+**ºþ|;ìèã?v(¨Z¥¥¥Ìüü|½qáÂw¶Í930ðÜr3ôâ« Y Y Y YÉÉÉzgY¶lYBBB"·Ê<ðÿù!¬ÿðÿ0~üøcÜ*=öXÐÓÊC'Yÿþïÿþn'xdd%Y$+É YIVHV@²¬ddd%Y$+É YIVHV@²¬dà®NÖýû÷gddx½Þôôô¸_jiip!Y$ëõ¥¤¤Ø÷[¿~ýèÑ£Ý/UWW»|àûßÿ>É Yr?U¬ÖÔÔ8O¿üòË`&NÈÏ YýÕ××Ï=Û¯ÍÉÉñz½ÇÓÖÏ=÷5+ ¨C­rùòå;vì»d½páBAAÁÅ¾ÚÜÜÎ~V@÷ÅÅÅÍ3§´´4999//OõX¸$«³¸¸¸¥¥¥q"##IV@7mÜ¸Ñçó©fµ§ÊÔÜÜÜ3gE²ÖÖÖæää;w.èÁM½dtBtåÊî!JYemX$kbbbà©5Ö¨¯¯OMMõz½ÙÙÙWÐMyyy[·nõÉÊ"®´´´¤¤Ä=¤ººzìØ±$+Éè¦¦¦#Fµµµéé;âââ6oÞL²¬^jhhÈÎÎV¾ú:lÜ¸±¯>d¯ÖÖÖÓ§O÷íg¬¬$+d%Y$+ÉÉJ²HV@²¬¬¬$+d%Y$+ÉÉJ²HV@²¬¬$+d%Y$+ÉÉÉJ²HV@²¬¬$+d%Y$+ÉÉÉJ²HV@²¬¬$+d%Y$+ÉÉJ²HV@²¬¬¬$+d%Y$+ÉÉJ²HV@²¬¬¬$+d%Y$ë¬û÷ïÏÈÈðz½ééép¿¤§>O/¥¥¥íÞ½d¬×bßoýúõ£Gv¿TPPPUU¥FyyùôéÓIVÉÚ3QQQî§ñññW¯^U£½½=11QGÄÅÅåææò3HVõõõ³gÏvñz½~ídff¬Õß.^¼èèñxvdd$½Áµ[[ZZü>¼½½ÝzÕ&Y$ëõÕÖÖæää;w.ð¥ÂÂÂÊÊJ5ô¨d¬×áò¿_®£QWWàñx|>ßþýûIVÉÊ"$+ÉÉÉJ²HV@²¬¬$+d%Y$+ÉÉJ²HV@²¬¬¬$+d%Y$+ÉÉJ²HV@²¬¬¬$+d%Y$+ÉÉJ²HV@²¬¬$+d%Y$+ÉÉÉJ²HV@²¬¬$+d%Y$+ÉÉJ²HV@²¬¬¬$+d%Y$+ÉÉJ²HV@²¬¬¬$+ ¬uÃW®Y$k$kbbâÈ#¾tõêÕ~Ú¹sçôÃ[ZZ"@È&ëÂ£¢¢æÏ_àR]]]YY©,--íþGÕÕÕ¥¦¦N`qq±HU'|277pw'«pÌ1Ò;iÒ¤'NMVÅjMMóôË/¿ÀëõN8p×'ëøñãûpwiÐdMIIÉÉÉQvfff;vÞ`@h&ë¥KlÇguuµ_oð²eË4¼³ìi²:ÓÓÓIV@ÈÖ¬6mjoo^SS£ljjêÛdÈÈH²ÉÚÙ!Áv6N[[[_õ766ZÍC²B-YwîÜßÁ¯øÕW_µRUÙÙÙ7¾ÕÖ××§¦¦z½^¦ÂdZ²^¾|ùºÙ»dåJpLÖî%%%$+dí½3gÎ¤¤¤|øá.T²<xd¬7äÊ+*U«öìáºÁÝpûµ¾¾~JçØ`cíµk×ªxUd¬Ýíûõ»¡¢ô­·Þ¢f¬vH0É YIVîd½zõªÒtîÜ¹ÉôzÂwW²>º¨¨ÈçóÅÅÅeggïÝ»ÅpÓkVwª=tèP;ÉÚwo²¶µµeeeåçç?~+WjrjkkY·4YC¦7¸¢¢BÉªLu(%	@²öÆ¥~U¬ÊV$ÉÚ¥¥¥eeeî!MMMqqq,Iµ7vìØ¤4uÌ7¯  %	pÛµw>¿£.))5jTEEÅÖ­[gÌ¡ =yò$Kà¦$«ÝüÜÎÌÌtvÚzêõzïêóYW­Z¥LÍËË+--u×¯ôq²^÷æç@²r¯HVzÉ1eÊ@²öY².Y²Ä=äÒ¥K$+díÚÚÚiì0àõë×+YÇçÜ½¢¢BCô'IV!`ß¾¹¹¹qqq#FÐZðÐ÷Ézùòe÷=Ï×®]Oo0Ð³k×®¡C®²­­íôéÓóæÍóù|ÇgÎàfõÛävïÞÝÔÔ¤FKKÉ L0Áï*â3fÌ(--eÎà&^I¬½ûvµ=J²*XÝ·½­[·N<9¾OÖË/+GãããíêK6mrÂÕëõ8qdýú~×¬Y£²9¾LÖóçÏoØ°!66¶¾¾ÞlÙ²Ezøðaµ[ZZ,X §dpW3gN~~¾S¶>:55µ¢¢9>KÖsçÎÅÄÄØÕgÎQ¾ÿþûîÑ®BÍ =6«ÃÊ+.]ª¶¤¤Ä¯$kô+MkkkwîÜ©LmmmU	«FSSUÑ«vyy9É 4(GU¤ªx7oÞæÍ!¸)ÉªìÜ¶m%«íRU¸Úp%ëáÃÕ?>É Y¯Ïv£ªTu'«Ñf¬áo¼ñÉ ´:thåÊË/ß±csdí³nl«v>ºÚ±±±u ¨9sfiiirrr^^JfÉÊ½n 7âââöîÝkO©¹¹¹óæÍcÎ¬$+ôFYYß:¤Ê9C²Þh²>|xçÎÎaM555$+p0gÎ¿«Ê A3$k_^ÝP'eIV¡mùòåùùùî![·nMMMeÎ¬7¬µµµJÓºº:zâÄ	;fdòN>´hÑ¢³gÏêé;×¬YÃ!Y¬vE¿Sknñi¬$+ÛèóÏ?ÏÎÎóuØ¸q#ódí²ÚåøçÎ4nB&Y6oÞ]Í½ÕÚÚªúù@²ÞP²^¼xÑ®Á4­CÁW?ï¾ûîøñãmçëÜ7H¿uùòåÚÍï½téR$@ß'«RóØ±cvÛs:fÌiÓ¦MzjWðù|Êà»7Yµ¡tèÐ!§Û'99yÝºu,L/nêù¬Ñ_~ÙÚk×®ÕÓ.@oðäÉýrtãÆ¹¹¹,L¬îmÔNMM#ÒÓÓU§ºèé¨Q£X_ÜÔóY§Lâ~ÉªÕïz=¥fÜÄdÝ½·¢ôÔ©SîdÍÏÏd­®®öù|ÎEAÕ1bGÕnV²~øáÊQ;éØ±czZQQq».ÃtVÆÅÅevPsÀ7%YÏ9¿iÓ¦À;ÊÍ=;Ä®nØÖÖöin¸¹5k;wîàðø|>¯×¶÷n®ÁýdmooWÁúì³ÏöúêêêRSSû»TUU©Q^^>útúÉjX>ìxéÒ¥îÂ¤Iìþ/ÅÇÇÛÞ·Ú/¿ü2:À&NÈÏ¸[µ¶¶Ö¹´auuµqèÐ¡j¸¯tè¾ÁÏá	èõzýÚU|òIÎÜÅÉzåÊ§]SSc×]RÑyW_¬ÇiGFFÒÁdõ»:¿ÝK®²²ò/m4YÞÞÞn½Áj¬PNÖ¹sç*KJJª««UPFEEUwP;''§Oµ°°PmÉÝÙýéHV@($«ßÎÔäNF¦§§ß`²ÚÓºººÇãóùöïßO²B3YwïÞ­Uì)ÿ9¬·òÂ$+àîNÖÚÚZ+IÕ Y$ë%ë¹sç%Y$kÁäNÖÄÄÄÑ£Gí ¹¹¹$+díÍäÎ9£¶ÏçS²^½zuÉ%X^^N²HÖÞS²¦¤¤XÛ®GH²HÖºRQQQÝEÛ¬·ÉdU:sæLçéÁ5¤¶¶d¬=ÖÜÜ¬­¯¯wmOons·$ëÑ£GW¯^½wï^$@ß'kiiéÐ¡Cìë×¯·'&&:·s³wo²¶¶¶¦¥¥õë×oÈ!Ðã®]»X¬W¯^UpnØ°Á)UõÑz|öÙgÝõëÓO?ÉúÀÄÆÆªfµÍÎÎöx<ýë_Y¤díËÞ`«Sö-[³Zo½õÖÝ¬Tµj±êP±®?Ç"$ëMLÖ#G;wNË/;ßyç»=Y«ªª£~U¶N8EHÖ>NÖ¢¢"«VõhV¬X5kÿþýýú~5Ô¬[³Z<yRm»ªK,	ý¬>ïÁlmmµ§3gÎôx<~ýÃõ¦$«rÅ¸lÙ²HV¬qqqP¾ÆÄÄ¨±zõj'À-JV÷o¾ùfÈÏZUU¥?¡Éä¨`ÀÍJV÷~ÖÀÓrBàØ`nE²Ïúî»ïZWpBBÊ»¹sçúC²HÖN)AGµ§.]r¿úþûï»ûIVÉÊ½n YIVÀÝ¬/¾ø¢»øViC²B*Yíro¼ñ3äðáÃ~÷#Y$kÏN¼QÃãñäääØ8ãÇ/..¶SqêêêHVÉÚoÖ®]k;mÚ´ÀÜëpuÃÎ^ÕªS¾â¾L?É Y¯_°N>ÝnxnÉZZZê7Î­¼É¸»UQzìØ1=VUUÙSÇSSSã­jTVVjøùóçIVÉÚæææ¤¤$÷Të®­­Õ£¾µsçN==qâÉ Y»[¹^7YIVÉÚÝd­¯¯?vìØüùó£¢¢^õUIHHX¸paLLÌ/¾Ho0díq²ê±¡¡AÛ¶m£f¬¬§NÒcuu5É Yo(YSSS[[[­­d-//ojjª¬¬`^¾|d¬=;ÉÚîXÛÛÛsrHVÉÚï½÷ß%øë	s¥ÉÚc.]R>ûì³öôâÅzúþûïÛK$+dí+W®é°eËÏ7öì-«jTUU©­¿J²HÖî^=Ø´ÊÑ÷ÞïÚmrËõÿñî¿ÿþââb* YûøÎçã;¨B]²duæÌvÑ`+X/^bÉ:xð`M×àjÜï½,X@²öå~Ööövç¨¥I&ù½ÚÖÖJÉ¢É¬©©±§ëÖ­³în- Yû Y÷ìÙ3½s7V¯îû³®]»Öî2ÉÚ¿ÿ§zÊ=ä¡8p Ë¬¬Nµjë1c®ÝV· Y5eeeî!S§Ní×¯Ë¬yÖMcc£"gÿþý!¬ª¿ï¿ÿ~÷î¹gÐ A,[@²öY²ÚÙ«Û·owîyî>iÃ¡¬?þñ5Q>úè©S§ôôßü¦¾ôÒK,[@²öM²Z¬VVV^í !·ønç·þØ`W'ÞÉÚgÉÚÚÚªhñûD¿d½råJH^©¾¬¬/_V¶··ë§Lâ!¼xñb5ksÀ°tó38àóù¼^oZZÚîÝ»Ý/µ´´¸+E®ÁÁÕ©JÝWbºvoòòrEµû¥êêêââb÷dffæææò3îîdíCñññÒ*Ý/)VkjjÜCG²HÖÿãõz¶%%%%''GU;vÞ`Ézî«5EFF§¹¹9==d¬×7|øp»®Õîl´ÎBd¬£°°°²²R=øõ766ZÍC²HÖë«««KHHðx<>Ï¹V¢cS__êõz³³³®$+d©+E@²ö½¦¦¦¶¶6, YIÖUVV6bÄ¤¤¤¸¸¸Ó§O³xÉJ²öÒ¢EÒÓÓ:¤¶2µ¨¨(++ÅHVµ7Î=üøq÷@íæÍYÂd%Ylß¾ªªØ²²20 YIÖSµ:jÔ(çÀ%5N>=kÖ¬+W²ÉJ²öFzzúòåË¨yyyqqq			ýû÷ÿù0 YIÖÞhhh1buÜ¸qÿôOÿ¤pùå([d%YiãÆ_ûÚ×_yåûöÑ±ÿÕçó±ÉJ²öÆÒ¥KKKKýªlmjjb9dí±¢¢"÷ÖÖÖ¸¸8=²ÉJ²öØÉ'ºk×.gÈ9s&OÌB$+ÉÚK[·nU¸Î1cÑ¢EYYYtÉJ²ÞãÇWTTmÞ¼~` YIVHV@²¬díÕ«W7îGùÅ/~Á$+ÉzCÒÒÒ"""âããûõë7hÐ ¿þõ¯,g@²¬½¡"UiªÕ*S½^ï#XÎd%Yã¾ûîûÖ·¾åòÎ;ï(kYÎd%YcÐ AûV#""XÎd%YcÄIIIî!Ö?Ìr$+ÉÚëÖ­SêØº|òI3 YIÖ^zþùç¦ýû÷÷x<JÙ´´42 YIÖµzõê×_ý/ùK¬$+$+É YIVÉJ²@²@²¬d¬$+$+É YÃ(YO:µ`Á^zI2 YIÖòðÃG¸L:åw¶¶¶åËäçç¿öÚk­­­Ìd½ÓõÇ?þ±ÒTk.gþê©W5ÜvÊÑ¬¬¬ÜÜÜê&LP[YËHÖ;:Y=OJJÈÐ¡CÄ¢ÛnÑ¢Eyyyî(UÐ¾ýöÛÌd½£Uê+ÜC¦NÊÍÏÑ·?¾oß¾³gÏúojjÚ»w¯¾K9ªw¹lÜ¸1??ù	¬wt²öïß_óÔ=$))iàÀ,jè£FÊÎÎ~åWl¸R¶   ..NÃG1yòäÓ§Oû½wìØ±z»ÈÖ­[UÅ2WõNÖ­ï¿ÿ¾=é¥ôÔ/kîT¥%%%QÅá¬<UXú|¾åË[îçZQQ¡¶¢tÆ6Zkkë¬Y³&Là÷3gÎÔgº(KKKÛÉz§|ï½÷*M=Ôøæ7¿ÉrÆjRRðÓO?Ý·o_~~~zzzSS2µ¨¨È=æ;®-99Ù½UmÙ»w¯ßÇj ÂõÓJ_%wg]ÇHÖ;ë|ÖuëÖ7N3·¦¦=5³JRsæÌYµjßÈÚ¸q£_â¬Y³Æo ª^Hf%+±¬	aaìØ±ª)ÝC6oÞWVVæ×yèÐ!«MõÀÙµk3 YIV Óxâââ>Þ¶¶6Åí¼yó¬ï×þJÛ¤¤¤À¬ww²655q>zÁïÄS5TZp®[·Ná:sæL>aÂ»BWQkïõù|~Ùdý_Ð:Âëõ¦¥¥íÞ½»/ÝödUÅ ï6bÄ­µ²;yò$ºOa©(µ%©ZUòäÉNÐ?~¼¢¢¢¬¬lóæÍîË=V¹«ákÖ¬¡ZHÖNTUU©Q^^>úô®_úî¹ï~÷»ßZ/¼ðbuÃjöÙg%%%zúÉ'|t6.£MÃW_õÐ¡CÌàn¡­á;:Yããã¯^½ªFbbb/ùåÜnC½£Õëõméãßÿþ÷ì±·Ðïÿû¸¸85T¹ÞsÏ=³fÍZºtiVVÖÀÕØuwt²z<§ÙÍnï~Ö#F?~Z[[kCJKKô£ù|>hö³ÞfÃooo·._µ»ùÒíMÖ¢¢¢	&<õÔSöôøÃÐ¡CÿøÇ?ÚµrXæd½+++ÕÐcAAA7_º½ÉzöìÙ´´4¥éÏþóøøøþýû5JOï¿ÿþºº:9 Yo'EQBBÇãñù|û÷ïÿß/ÑÙKwÈY7Ç<xpTTÔøÃÏ>ûLC>úè#Eì/~ñ9 Y¹RDo?^uª].gÇÉÉÉ¿üå/5]­@²¬½1gÎ¼¼¼Q£FEGG§¦¦ÚÔ¸j¬$koÙ½07nÜ×¯_¿þ4»Yìd%YÌN¼yóÍ7¦O>ùäÑ£Gõ8lØ°<x%HVµÇvíÚ¥:thQQÏç+((hmmðÁ¿ýío³äÉJ²öFttô¯ýëuëÖ544Ø_ýêW			,y@²¬½³zõj÷ç^õ+KÛÌ3UÊk¶dgg=zd%Y;¥¨PzüøñÒÒÒÉ'?óÌ3Ò#KimmÕü2dÈTÍ+îÌýÐúª/¼ðÂ¤IfÍõÉ'ðÛ YoÓ§O<¸ÿþßúÖ·òóóÐ¯_¿>öØcë×¯çÜÖ©S§*Vÿú×¿:CT¼>üðÃwÚ÷T*òcbb¾ýíoëêGÔv@g#×ÖÖ._¾üí·ßæbÉJ²Þ©©©ÉÉÉªÌ¨þóíñxÛÔÔ¶Ëß< jÕ=¤ªªJÛ"wÚ÷Ô/5vìXç©bUáX¹jS©   ))iÞ¼ysæÌÑ»ÊÊÊXË$+ÉÚÎ=;bÄ­p+**´jVcéÒ¥±±±3fÌXµjÕ¸qãJJJÒÓÓâõÐ¡C*tZ[[ïÀeE[÷îUÞ·ÉúÂ/¸lÚ´éNKÖ0ÀïGQå:kÖ,¿1-ZíyüøqÏW]]Í YIÖ¾LV»y*³·ß~[CâââÔVM£ªi4$33sÝºuûöíS­3jÔ¨¬¬,U´Ë/¿s¥©CúªÊÈÈÈáÃ«8»ñëIM:U3ÄZ<òÈÖ¬2:&&Æo fÈÄû'´mä²råÊ3g²¢HVµ/©$UÁZZZªjUõµ¯Mù´fÍµ*Vè¼øâ`¥©¯ÉÉÉz×­ü·9sæèïÞsÏ=ú2cÆQa­ï©öjÿñÔ¦À¯ýëï|ç;ñññ¾Á?ªÐJHHPzmÛ¶Me½×ë½Ó:zôh`ß¯¶~&O¼cÇm<9¯­rssYÑ$+ÉÚjkk ³fÍRÚ´iàÀO=õÖ¿ßûÞ÷T¡®^½zúôéEEE~kd@·òæåå©ûè£®ßýîw¯³gÏÖwÐðzH_xÂ	ªÀ?>räHU®Ï?ÿüý÷ß?dÈUÛ7X+§û÷ï¡GýE÷MwUÒÚÐÏgAß÷)k~úi¥¦JyýX6¶¶nÝê~£6ì"HVµ/©-((Ðê8***))éñÇW¾jÕüàFFFª¡ô²Kö»4èCåU¤jSK¨*°Í7+9|òI±Æ*Á,X0nÜ¸ñãÇÇÆÆjBÿöoÿÖë?½wï^åtJJÊÆU¹Þ±'ÞL8Q¿¾ª¶ôø§?ýÉkiËÉ6/Ö­[§ß×¹*Huuµ^r YIÖ¾§5ï~ô#­ =ªØ3gFGG?úè£zúóÿÜ/u·ì-]º´´´ÔâSUéäÉõ×¬"Lùªmeª2ïO>Qr455)Zô+**òóó¿ùÍo>Ó»?ÒÁ=dØ°awì 5~øa»9 cÎ9¯¼òµß~ûmý¦cÇÕ&æSÎ YIÖEÕ¼ÊÊÊ²²2­íÈÎZØò;å¦RFÚ6JVÐªíØ¢üàÊÑùóçÿøÇ?Vå¥ï¦ðSÖ*UÉ¥¦¦N:ÕRJ2DÕªRêN¾¤wæ¶jÕ*÷qÂi*a?ýôSÎWHVõV¨ªª«@üÞ÷¾§á&LPª©¡2èV®íÎ<*Å4»ugÏU	«/£PÙzÿý÷?ðÀJÐÜ*aò¨Nzýõ×õmõÊîÞa¦æ6)îä@jøU¢EEE·d½mì@S÷Õq^^¯wõêÕÊ³­[·ªó;sãÖX³f2RU©2rÔ¨Q=ôÐï~÷;«JXU«|ðAmm­J[åÊk¶JÖO>ùdòäÉÚ8|ø°:=¢=bÜ¿¿>G	­%ràÀúwìÿßÎÔ+Wj; /÷d½ýÒÒÒbcc->ýðÃë©sNço¾©IOOÿío¿jVÕÊÈÑ£Ggee),õÅô«î-""âÞïU[RRÒÜÜ¬PTÄj|gÛJ¨®®þüóÏ»þ£ªµµ¡ÿÙÏ~¦¶uÊ)wrWª*ÔAizGu¸Á££¬$ëRV=ðÀÊÕýû÷6ls(¬BE¡Õ¯Ê×[ÿõT/æççÛµo¥¥¥ªSý®@¤!Cyî¹ç-Z¤VÝùÙg©ÌÍÍÍUÜjCAUøw¾óëÙ¶ËbtñÜÚ¼PåªâïgÑ¶S~ïäÿm:hCäÐ¡CìL@²Þ)¦+W®t_@U ÒÔ)UõÍõ´w]¬7BQ1kÖ,%¢òRQ§Ê,°«Sqûü$//ÏÓÁn30`ÀMÑ§~:xð`ù±cÇ*õ^5º8Ë¹¤uùòå*5É/¾ø¢òØ]×nÜ¸qÓ¦MîWõêÕÏ?ÿ¼ßP@²i²Rú[Öíº®Â¬¶¶öøñãAk2åett´õµ×^ÓÓÊÊJ«³~eeevF¬YA«Æ¾ûº8È.YWW§Ç¢¢¢U«Výô§?UÉ«zÝNnQ«OÔYºtéVk >Y®z·o/h$k$«ßsÊ;óÛ4(>>>..N_rÔ¨Q±±±iiisæÌ±«^(VÕvFîâ£¢ú»T²Öûî»Oå»ÂUy¡à´É/¼ ýÍo~£WÛÜZïºþ4 YIVÿd:uªBå¼¥QÂ)ÒÎvÐSU¯¾úª¤]©QÅ«õè^÷JëÖ­ÓÄ¯~õ«òòr¥õã?7qâÄ5kÖôïßÊ)îUë¯6Ì¢ªú¹çÓÜS¾ê¥.NXR	^]]½yóf2ÉÉzìgí¦ÈÈÈmÛ¶9O¦ª>­]¶lÙC=¤ëÎÝ4Î£>ZPP 9 íä3flÜ¸Qùí>äøËE8PùêþÓ&LÐVÈ'|¢ìÔKv=)?¯½öÚÐ¡CóóóÛjÜáGH YIÖ¾qãÇ«T¥¨|²uû] ¾)Àô'½úCúÂiiij¨ZÕèU»#íírT#+_yöõ±6ÄnïùgµÛÑqG[ùþô'«¡5ääÉz¯û4_t7))É9Qxß¾#FpJ$kh&ëç³ªòKMMµâu×®]ªU«V]÷ÄÐ/:úEf*Ë=ªlÒ¤I6àÍø¶wÓëõ~ûÛßV *ØrssUn*õÞ~ûíÏ>ûLæpÑøÈÂÂÂªbÛöÑÚJÎ56mÚäñxæÎ«¶Uu­ú&ßøÆ7-Z¤ÉÏÌÌTjþîw¿sþäÉ×­[çWûÞºHÖLVwLÚñKKKí4ëv`VTT(ZTðeuX¹r¥RgÐ A«tÚå+¦NúüàÆOQòiB	­zô¥^ÒÖMµÅö>8lØ05ôZÄ*ÔcbbÇhweW¨+ËUª¨½÷ÞÝ×ÍWõï·iÂS¬á¬Îißýîw¿õ­o9gp*3»ufÁvD®ªF%êÅ?ýéOCQbÝâû¨÷&PÛ#:5J%û_]ê^pß]NsÆ&Véû£ýH1i[|ðÁý÷ßÿÔSOýë_?yò¤S³úmQ³ YC?YUW%%%)ñññ*¼~ñ_¨Vñúøã¿øâ~oY´hS#¨,ûÞ÷¾§ÀÐÇ×pEÎc=æñx¾öµ¯©»æsÈqw(###5ÓV­Z5cÆhýò¿TQk·ýý¬HÖ°MÖììlçÚCvÇ~ýúýû¿ÿ»ª1¥Â7¾ñÄÄDw¿®*9ÅªuîØ±C	Ï=÷üêW¿RÐ*_KKK~úÓ*zCø:|Ó¦M+**R`wºUé¯ëÖ­²~à×^M3c¬a¬­­­§VËÈÈP(í¢ì¾ûîs÷µ[¿¹OÍlhhP¡6xðà¨¨¨>ø ¤¤DÚÉ'9<ðß!<¡DÕêØ±c<¨éµm Øs4ÓìRP6Ç6nÜ¨úóY¬a¬ÊTó´¬¬,22Rµbw+;w®s+ÐÍ7+6ü>$++kæÌªtU³Î?ÿO>QÙªÕíJ¡JÅhZZÚèÑ£µå¡Ùb_tôsóT$køö'''»KÒøøøþýûëÑ®zûÈ#8Æ<y²ß'¤¦¦~úé§CÕO«Gµ*Ý1z¼[bêµwÞyG%ûÄµ1áT¥¶«HÖpLÖêêjeáºuëlÇjLLÌ=÷Ü£Xî¹çvìØñÌ3ÏDDD:5®²ÓÞ®ºVï-**ÊËËs+V´ècÕ!¿@«BU±nG*>ZóAE<7z@²õY77nT64Hõë÷¿ÿEé	V­Z¥:LéøÓþTÅ«så£5kÖh`YYrW¡bÇì|Ñ±ËV¡2bÄ3fèíj¸Oîm¯½öæCRRóóóÙ¥dåJÿçW^IHH°«M6í©§RT8ð>pÆQ¦êÕÉ'|úé§î·«tSù«5ï°vòäIJU$+É<YyyycÇU9«tÀÃsNÍ¬$kw<x0""â¹çS¬ÚÙ8+V¬è×¯ÊS®Ì$+ÉÚ³fÍRfff.]ºtâÄýû÷áì2,¾@²¬½ñøãÛñ8?ü°rc×`ñdíU«ÎåæMYY½Á@²¬½dw]unf§ß444°øÉJ²öRkk«ÝÖ;==Æ$+É YIVHV@²vêÀ>Ïëõ¦¥¥íÞ½ÛýRKKKÉ Y¯¯   ªªJòòòéÓ§»_ª®®...¦f¬=õêU5ÚÛÛÝ/)VkjjÜC¦HNNæ¼OÉú¼^oÐ¶¤¤¤äääh`ffæ±cÇHVÉzÇiGFF§¹¹9==Þ`Éû ¤áÃ···[o°Ú½¥³Ð%Y$ëß(,,¬¬¬TC~½ÁV³æää¬õúêêê<ÏçÛ¿¿SÔê±¾¾>55Õëõfgg+ÉÊ"$+ÉÉÉJ²HV@²¬¬$+d%Y$+ÉÉJ²HV@²¬¬¬½2þü!C¤ô©¤¤¤¿û»¿K	?ð¯ýëá6ÕdMxþÜa;áúï~ðÁÃpÂGþÐCõùGGGr²666þ¦¯õë×¯¤¤ä7aæ_ÿõ_#""JKKÃmÂ/^¬	/++·	×V©&|åÊá6á?ûÙÏøðóÃþpØ°aa8á¹¹¹ÚêÛÏüíoÛÖÖ²Éz3(YÿûßÛT·¶¶j=ûç?ÿ9Ü&ü/ù&üäÉá6á»víÒÿ÷ÿw¸MøÚµkk¶eË©lÃ	ÿçþç'xâÖü-d%YIVd%YIVd%YIVd%YIVd%YIVd%YIVd%YIVdí#£Gþÿøpê¶¶¶Q£FöÙgá6á'NÐÿ×ýW¸Mø¾û4áÿó?ÿnþ?üáG	Ã5Ûo~ó'áÿú×¿.,,$Y¸û¬¬¬wø|>¯×¶÷îÒsçÎ%&&v1á¡7+öïß¡)JOO×ÔÉTË#GÆcUWW>nvîÜ>Ë¹´´´D¸Ï_¾|¹¸¸822räÈµµµ·eÂIÖ ªªªÔ(//>z¨N¦V¯©©©Îê&èÞ¬HII±ë|®_¿~ôèÑa2Õ¢5HMMüøøøðp¹rå6§E=L&¼ººZÓõ-$'|ñâÅo¼ñÆÕ«W«vô­p5­wô«¨ÑÞÞî.éBÌ¤IN8áNÖÀ	íYS½ûvÕëa5áK,Y¶l³¨É+Vm[*ÜþÁµyôèÑÛ;á$k^¯7h;$¹5pÂCxVÔ××Ï=;¬¦Z¥[LL~qÕëá3á§NÊÌÌÔjÔYÔÃdÂSRRrrr49ücÇÏkBV¬X¡íf¬¼-N²áñxvdddø$kàê¬¸páBAAÁÅÃjªÍÎ;>¿gÏ÷¢n¿xss³õRÉk¢***ÔÐö¶*nË¬Ah½ÓÞÞn¶dðZÑ·´´ÕTöÊÉGü­ðüÅ-<ÂdÂÝr»u5ÂÂÂÊÊJ5ô¨Ê&|5pÂCoVÔÖÖæää;w.¬¦úZGßà#G®u­9>¸¨Ï/ÞØØhaõÏ3gÃ×:ÏÊÊº-N²QWWàñx|>VCá¬z³"11Ñ¯	©¾ÖqAZZ6áÇoõzLxà¢&^__ª_<;;[á>~þüùüü|ÛÁ|âÄÛ2á$+$+$+$+ Y Y YÉÉÉÉHVHV7ç_:"bÌ1ª««×®]«!üqãÛý@º`·_¸p¡=½xñ¢Ú-0¬@HÙ¹sg~¨ÒîàÁz´Ûc¹SpÛ¶mÎÔÔTEoQ-[¶ÔÔÔh)S¦8CÞ|óM9þ¼óºº:qn	b¹iÓ&æ?@²¡æòåËî²²¡¡AõõõÖ¸rå¥ ØF;uê9sFãÇ·î»ÕZ»`Á¿!`Kq§4iÚï½÷ÚåååüÉ»Óª=zT	×ÜÜ¬Ç#Glß¾]ÖÖV¿dõù|*L-_cbbT¡ª¡4--ÍÖªª*9|ø°_Õ«ÏtRüÂîÏ@²¡ÃJUåë"##­¾TZïùóçýÕ©Aí6àÖl®Ý#ÚvÓºG~ë­·4P¡«¶ñ;ï¼£!Äj|øáüÉsçÎÍ;W	·~ýzFUJ;5Te&«îÙ³'11Ñ:u_ýu<vìXBBÂôéÓÕpxû´iÓ¬ÙÞïÒú~dBÊ¶mÛCl?kyy¹³gÏ¶dÝ¾»©øÔ«Öol»H¬ÖÜØØèªõøÑG9Å®JUçp'+yù	)~'O´TXªlUãÔ©SNjãØ~Ùäää-ì%kÄÇÇ+,¯^½ªw©mã;q`Íj/ Ya»Z÷ïß¯Çwß×	¼%K9ÒyÚÚÚê¼Åbµºº:ð£ZZZì°á+W®Æ'5+@²að?1oÞ<kddd¨±aÃgRR3³U¯êiaa¡îLII	ú'¨YÖñÛÖÖ¦öîÝ»Gmõ¨+Y-óT:»Zíh&«G<¨Gf]¾*X»ojVdBä>æèý÷ß×¦¦¦k_QmqèîÝ=þ¼T:ÉªÄÕÅsOkÖ#GtÊÉà®áðòåËJÄ´´´ÚÚÚë¾kÓ¦MVæ*5þÈ#sssí¥ÖÖV½¬k×®µ(j|¿uÞ¼yv¹	~dÂÎèÑ£«ªª§v©¦Å;C.àW¶Zgree%)@²@²@²¬¬¬dddd$+$+$+è¶ÿÿþ¬±±0IEND®B`


SORT CASES BY 编号 (A).
DATASET ACTIVATE 数据集1.

SAVE OUTFILE='H:\u盘文件个人\1U盘3.0\2023年\9月份\20230901-1200-自己\20240418-1600-自己\未标题1.sav'
  /COMPRESSED.
SORT CASES BY 训练集是否好转 (D).
SORT CASES BY 测试集是否好转 (D).
DATASET ACTIVATE 数据集1.

SAVE OUTFILE='H:\u盘文件个人\1U盘3.0\2023年\9月份\20230901-1200-自己\20240418-1600-自己\未标题1.sav'
  /COMPRESSED.
EXAMINE VARIABLES=年龄 APACHE评分 Marshall评分 吸氧浓度 氧分压 氧和指数 二氧化碳分压 血钾 血钠 乳酸 血钙 剩余碱 肺泡氧分压 白蛋白 总胆红素 ALT AST 肌酐 尿素 尿酸 总胆固醇 甘油三酯 血淀粉酶 白细胞 血红蛋白 血小板 PT INR TT D_二聚体 CRP APTT PCT CK CKMB BY 训练集测试集
  /PLOT NPPLOT
  /STATISTICS NONE
  /CINTERVAL 95
  /MISSING LISTWISE
  /NOTOTAL.


探索


附注	
已创建输出	25-JUN-2025 15:19:54	
注释		
输入	数据	H:\u盘文件个人\1U盘3.0\2023年\9月份\20230901-1200-自己\20240418-1600-自己\未标题1.sav	
	活动数据集	数据集1	
	过滤器	<无>	
	宽度(W)	<无>	
	拆分文件	<无>	
	工作数据文件中的行数	282	
缺失值处理	对缺失的定义	因变量的用户定义缺失值视为缺失。	
	已使用的个案	统计信息基于个案（无任何所使用因变量或因子的缺失值）。	
语法	EXAMINE VARIABLES=年龄 APACHE评分 Marshall评分 吸氧浓度 氧分压 氧和指数 二氧化碳分压 血钾 血钠 乳酸 血钙 剩余碱 肺泡氧分压 白蛋白 总胆红素 ALT AST 肌酐 尿素 尿酸 总胆固醇 甘油三酯 血淀粉酶 白细胞 血红蛋白 血小板 PT INR TT D_二聚体 CRP APTT PCT CK CKMB BY 训练集测试集
  /PLOT NPPLOT
  /STATISTICS NONE
  /CINTERVAL 95
  /MISSING LISTWISE
  /NOTOTAL.	
资源	处理器时间	00:00:17.92	
	用时	00:00:17.75	


训练集测试集


个案处理摘要	
	训练集测试集	个案	
		有效	缺失	总计	
		数字	百分比	数字	百分比	数字	百分比	
年龄	训练集	91	100.0%	0	0.0%	91	100.0%	
	测试集	23	95.8%	1	4.2%	24	100.0%	
APACHE评分	训练集	91	100.0%	0	0.0%	91	100.0%	
	测试集	23	95.8%	1	4.2%	24	100.0%	
Marshall评分	训练集	91	100.0%	0	0.0%	91	100.0%	
	测试集	23	95.8%	1	4.2%	24	100.0%	
吸氧浓度	训练集	91	100.0%	0	0.0%	91	100.0%	
	测试集	23	95.8%	1	4.2%	24	100.0%	
氧分压	训练集	91	100.0%	0	0.0%	91	100.0%	
	测试集	23	95.8%	1	4.2%	24	100.0%	
氧和指数	训练集	91	100.0%	0	0.0%	91	100.0%	
	测试集	23	95.8%	1	4.2%	24	100.0%	
二氧化碳分压	训练集	91	100.0%	0	0.0%	91	100.0%	
	测试集	23	95.8%	1	4.2%	24	100.0%	
血钾	训练集	91	100.0%	0	0.0%	91	100.0%	
	测试集	23	95.8%	1	4.2%	24	100.0%	
血钠	训练集	91	100.0%	0	0.0%	91	100.0%	
	测试集	23	95.8%	1	4.2%	24	100.0%	
乳酸	训练集	91	100.0%	0	0.0%	91	100.0%	
	测试集	23	95.8%	1	4.2%	24	100.0%	
血钙	训练集	91	100.0%	0	0.0%	91	100.0%	
	测试集	23	95.8%	1	4.2%	24	100.0%	
剩余碱	训练集	91	100.0%	0	0.0%	91	100.0%	
	测试集	23	95.8%	1	4.2%	24	100.0%	
肺泡氧分压	训练集	91	100.0%	0	0.0%	91	100.0%	
	测试集	23	95.8%	1	4.2%	24	100.0%	
白蛋白	训练集	91	100.0%	0	0.0%	91	100.0%	
	测试集	23	95.8%	1	4.2%	24	100.0%	
总胆红素	训练集	91	100.0%	0	0.0%	91	100.0%	
	测试集	23	95.8%	1	4.2%	24	100.0%	
ALT	训练集	91	100.0%	0	0.0%	91	100.0%	
	测试集	23	95.8%	1	4.2%	24	100.0%	
AST	训练集	91	100.0%	0	0.0%	91	100.0%	
	测试集	23	95.8%	1	4.2%	24	100.0%	
肌酐	训练集	91	100.0%	0	0.0%	91	100.0%	
	测试集	23	95.8%	1	4.2%	24	100.0%	
尿素	训练集	91	100.0%	0	0.0%	91	100.0%	
	测试集	23	95.8%	1	4.2%	24	100.0%	
尿酸	训练集	91	100.0%	0	0.0%	91	100.0%	
	测试集	23	95.8%	1	4.2%	24	100.0%	
总胆固醇	训练集	91	100.0%	0	0.0%	91	100.0%	
	测试集	23	95.8%	1	4.2%	24	100.0%	
甘油三酯	训练集	91	100.0%	0	0.0%	91	100.0%	
	测试集	23	95.8%	1	4.2%	24	100.0%	
血淀粉酶	训练集	91	100.0%	0	0.0%	91	100.0%	
	测试集	23	95.8%	1	4.2%	24	100.0%	
白细胞	训练集	91	100.0%	0	0.0%	91	100.0%	
	测试集	23	95.8%	1	4.2%	24	100.0%	
血红蛋白	训练集	91	100.0%	0	0.0%	91	100.0%	
	测试集	23	95.8%	1	4.2%	24	100.0%	
血小板	训练集	91	100.0%	0	0.0%	91	100.0%	
	测试集	23	95.8%	1	4.2%	24	100.0%	
PT	训练集	91	100.0%	0	0.0%	91	100.0%	
	测试集	23	95.8%	1	4.2%	24	100.0%	
INR	训练集	91	100.0%	0	0.0%	91	100.0%	
	测试集	23	95.8%	1	4.2%	24	100.0%	
TT	训练集	91	100.0%	0	0.0%	91	100.0%	
	测试集	23	95.8%	1	4.2%	24	100.0%	
D_二聚体	训练集	91	100.0%	0	0.0%	91	100.0%	
	测试集	23	95.8%	1	4.2%	24	100.0%	
CRP	训练集	91	100.0%	0	0.0%	91	100.0%	
	测试集	23	95.8%	1	4.2%	24	100.0%	
APTT	训练集	91	100.0%	0	0.0%	91	100.0%	
	测试集	23	95.8%	1	4.2%	24	100.0%	
PCT	训练集	91	100.0%	0	0.0%	91	100.0%	
	测试集	23	95.8%	1	4.2%	24	100.0%	
CK	训练集	91	100.0%	0	0.0%	91	100.0%	
	测试集	23	95.8%	1	4.2%	24	100.0%	
CKMB	训练集	91	100.0%	0	0.0%	91	100.0%	
	测试集	23	95.8%	1	4.2%	24	100.0%	


常态性检验	
	训练集测试集	Kolmogorov-Smirnov(K)a	Shapiro-Wilk	
		统计	df	显著性	统计	df	显著性	
年龄	训练集	.082	91	.168	.952	91	.102	
	测试集	.098	23	.200*	.975	23	.806	
APACHE评分	训练集	.114	91	.006	.968	91	.025	
	测试集	.189	23	.033	.959	23	.451	
Marshall评分	训练集	.170	91	.000	.888	91	.000	
	测试集	.288	23	.000	.788	23	.000	
吸氧浓度	训练集	.226	91	.000	.834	91	.000	
	测试集	.196	23	.022	.903	23	.029	
氧分压	训练集	.157	91	.000	.825	91	.000	
	测试集	.123	23	.200*	.943	23	.206	
氧和指数	训练集	.146	91	.000	.846	91	.000	
	测试集	.102	23	.200*	.946	23	.237	
二氧化碳分压	训练集	.073	91	.200*	.968	91	.026	
	测试集	.089	23	.200*	.986	23	.979	
血钾	训练集	.110	91	.008	.929	91	.000	
	测试集	.153	23	.172	.902	23	.028	
血钠	训练集	.087	91	.085	.959	91	.006	
	测试集	.216	23	.007	.871	23	.007	
乳酸	训练集	.171	91	.000	.881	91	.000	
	测试集	.251	23	.001	.773	23	.000	
血钙	训练集	.076	91	.200*	.973	91	.057	
	测试集	.121	23	.200*	.960	23	.473	
剩余碱	训练集	.094	91	.045	.969	91	.030	
	测试集	.153	23	.171	.944	23	.216	
肺泡氧分压	训练集	.186	91	.000	.907	91	.000	
	测试集	.177	23	.059	.950	23	.293	
白蛋白	训练集	.060	91	.200*	.972	91	.046	
	测试集	.153	23	.176	.951	23	.301	
总胆红素	训练集	.181	91	.000	.767	91	.000	
	测试集	.206	23	.012	.891	23	.016	
ALT	训练集	.338	91	.000	.468	91	.000	
	测试集	.245	23	.001	.810	23	.001	
AST	训练集	.238	91	.000	.505	91	.000	
	测试集	.192	23	.028	.810	23	.001	
肌酐	训练集	.143	91	.000	.903	91	.000	
	测试集	.231	23	.003	.816	23	.001	
尿素	训练集	.121	91	.002	.926	91	.000	
	测试集	.195	23	.023	.798	23	.000	
尿酸	训练集	.116	91	.004	.869	91	.000	
	测试集	.113	23	.200*	.963	23	.518	
总胆固醇	训练集	.181	91	.000	.850	91	.000	
	测试集	.223	23	.004	.888	23	.015	
甘油三酯	训练集	.248	91	.000	.629	91	.000	
	测试集	.258	23	.000	.766	23	.000	
血淀粉酶	训练集	.205	91	.000	.709	91	.000	
	测试集	.249	23	.001	.713	23	.000	
白细胞	训练集	.115	91	.004	.916	91	.000	
	测试集	.137	23	.200*	.971	23	.722	
血红蛋白	训练集	.086	91	.095	.990	91	.699	
	测试集	.104	23	.200*	.973	23	.763	
血小板	训练集	.236	91	.000	.411	91	.000	
	测试集	.146	23	.200*	.972	23	.745	
PT	训练集	.147	91	.000	.834	91	.000	
	测试集	.352	23	.000	.535	23	.000	
INR	训练集	.105	91	.015	.955	91	.003	
	测试集	.269	23	.000	.780	23	.000	
TT	训练集	.193	91	.000	.791	91	.000	
	测试集	.192	23	.028	.817	23	.001	
D_二聚体	训练集	.185	91	.000	.705	91	.000	
	测试集	.269	23	.000	.728	23	.000	
CRP	训练集	.076	91	.200*	.982	91	.250	
	测试集	.123	23	.200*	.970	23	.679	
APTT	训练集	.308	91	.000	.452	91	.000	
	测试集	.147	23	.200*	.937	23	.155	
PCT	训练集	.238	91	.000	.651	91	.000	
	测试集	.179	23	.054	.872	23	.007	
CK	训练集	.359	91	.000	.288	91	.000	
	测试集	.185	23	.041	.838	23	.002	
CKMB	训练集	.470	91	.000	.099	91	.000	
	测试集	.223	23	.004	.725	23	.000	

*. 这是真正显著性的下限。	
a. Lilliefors 显著性校正	


年龄


正态 Q-Q 图


;ø£i`` ,|Yýh-A.;wî¶dÏÈÈpÛ.-ÛÕ3åç£Gm-YÕ×&õü/ ¾¾zdÉL©ÓÓÓÝ6º%«fn«sê ð·÷_Tà­­­*ZÛmµt-l´ÈGqÛç>zC­"tKÖQ2F=+õëïíÛ·Géè¤®R¥OqJJúµòÆn·úüóÏKJJä/R7|·ÞzKÅª:î:ô±Z­Ã>ùF"×Ê¿`ØBZ¿~½v'ÙÙÙ£'ëÑ£GUËÈ%KÈÆ³gÏê÷!YA²L"P.ëëë¥âT?ÝI&¥§:«ýÈªÿýOWrT./X°@].V	íÞ½[ÅäkMMÍ°wÜ5((Hú)Y_xáµüÉ'¨')Á&ËÃ°íííê®ä²ªªJû0ô¸®'¬«ä)ÉÆ^zIMÍÉÉýõ×_UÕzòçK;¨;×ßCÉµÞÚ¯¤ê×_UK«ÊrèÏÕï¾û®lT¤ÕÎ.ÔåyMVµ,«:¾íÖdÉL©lT§¤akV7ªtSêé/ÚhÌ®²XXÛï©ê°VM¬cXü6L*Õ¯ª¿ä½:îªKkG_åh·Óuë«X7¨òµ@=<£Zoqqqú2÷Ø±c6M¥.ÖÒÚ¨þ(í`F*`ùÛµ¼ÿvÈAõåË¬ê«¾î¨oIII*³IV¬ÀT9qâ~0ëÐdÕhjgùèWTÕ¯òI?H_õÓ2lÍªuåÕ«WÿgÈ¡C-=LV	9ýX	$	0U£+r­ä¶*ù'U»zÚª°¬ÊöÃªÝnw«tå*Ùb6µZJRùn¡Êo|Õzp·6qkêaÓ|ÿ¶Jî=öx¬êÏÊòªzW¿JhU|«: YÉ!ôÚÈíà¤v4ØmÈªíT²JnI¨Ã°ê£cêç@·£$«Pn;¨~7»vízU«üÔ¸*Ã´ÚZE·¢íZØ¨%/U	.ª´î?öuD"Sý¢©~Ë;×åûS«ö ?þñõ«+W®tÛGP½ÝnôdÕFC)êèfuà$+0ùýÐýh°JVuØV>ôUÿ#)øÕ¯ªê8êho±Uüñ$«ªðTÌ«ËÚ¾¾>µ]»u,Z=®65##C×UrKKê¬ýx,<ìÄF)))n[CSÒ÷Uv7¬í#ÏvØ4õäh°Ø¾»ºg­_±¶Éx<IVõýúu©,U&©X¶gÐÐÔÿÎê6WÃ·÷§úÓ÷bõ$YõãaÔFRëHvPcÕ!_ÕwWäÕÒjÉªúè;vLâÓjµ®^½ZUÒjl«ªGê÷;lÏÞa÷Tcy=º%«Jh53Ûl$+HVàaP?¬Út?òI-Á£VKSÕU?¹Òñ Íq(wb6ÕýKpjû×××¿øâ*e»êL«:k½jUY¦ÝJõïUSøýÉVTÕ/è¯ÒjÖÌÌÌoïOé ¦WÔõÔ©S£üujÑÉsS?j[Ô©£§²[ÓÉi®ÔÔTíi¬ZËö$+ðP©ÉFJÖ7n´¶¶j«6nécÎÁ¤õvëÑ£ú.¹uPi¼Ô°EJÞ¥Kò3°¾hll4Lúç&ßFðÛû¿ëïa¤ÖÏÒ,iº~ýú¡g86¦¬äåÐS)¸Íæ¨?u~çÓI$+0qóÒ·®®¤[°`Áë¯¿>®«oGép+ßWüýýÝåoïw-s¾åê°×þýæÛû»xÁdd$+$+$+ Y Y YÉÉÉÉHVHVHV@²æÆêÝÿûvòñyï½÷ÝùöíÛríùóç/^¼8ÊÙÚ=tçÎaÏ*÷|òäIý¹Ê§âÑÿöHV`2^Ê>>ï¼ó~|:/yß#GjjjdÏ>ø`¤ûÚÛÛµûÔï)«Û·o×¯:Yhhh[<xPÄ[eaõêÕú»5:2H®åWÔóåcÇ©®_¿.«]]]ñññ/¼ðÚAÉS§NÉµúGwµ±¥¥E.SSSÇûèzí¬ÀäY-#å3WbRÿ9®/»wïªÅXÊ]Éý«ûlll¤V«ªªÔn²*ã'®^½:4fôº»»åÚòTË/«åRöÖ­[²óæMy É¤í)qzåÊÔAZìÞ½[¤,>|x¼þø·@²LKÊ÷îÉçott´|p¯_¿^.eõO>K©íÞÐódu+%6,Y¢VUK`¨OIzµQ=ÉYÖßÉíÛ·7nÜ(×æäähOU*6µ ûÔþ:u.¿øâ¹<zô¨JJ²jí000 ÂZ ëÑ'|à¡µ@²S¢««KçM>v/0zÍ:®dxù4W,jYÅÞÁ%ÛÕáM·0P*àRRR&ÇþýûõÂ+¯¼¢ç^»vMUêp¨*U²J-+ËeeeúÃÃ6Â¸L¼ìýznçÎªÌ+WÜBE>ÊÒ	WYÐ%U«<¸|ùòèÉáÚ£»ÁÍ7µ¤¤ýKD¦Ëå:yò¤6çÎ¬êyªÃLKiÔÅ'ðè>ÃyÜÚ Y©òê«¯ê5!!aèÇ±vdÕóUqKÖÖÖ¡É!É­j,É<)+åqe5;;[+³Toõû¢,üS;gddhÝ©Î=«í&÷|øðaÙ¨z3µ··¬BûÙUKYYP­148=yôÇ¿R­ªÊõÛÁ>M²¬~vÀ£ÁúäjXeDÈÐäPCDBBBdoìSKMÖßßªeåÊ² e,¿ûî»êÈ§zþÚoÃªo°*=Õáî1UÊ²JE?º5ë#od&|ËGí=äR_Nn²:úúzYòk¤£zªºRµÜê:;´zÎÌÌT"EÛ=H©?JRÅÍfSÕÊC5)))++Kjw-YÕ7ùæ!Ù&·õüÑÇ[³>üöHV`J¨/RÕ©ªèã?ôdO+ãÔIº%m²lµZN§4òé§ªÚÛÛ?ÿüsÕ§W_Éyê&úTõ©r«or?êGSÕû×mÔªó/_.iºûvÕ·Vn«:oÞ¼YX<ºaû$+0¯WÝ§­¶¯bF>¯Þ¤¥¥EWaXòøðácÇ<yRm¹pá:©e:úÒK/iaþüùÖÖVUQ%ÚâÐ¡CÚ³üøöþ[QVV¦~qTãPUÆlØ°A¶ÈUAAA²E²VJVµç©:7©CÇm¯ÚUò¸*ÀÆõèzí¬À$éãU3)ä#X>ÄÝAýVWRRâáI&¹Ía¤eùÐÞ4ZI¶ýîw¿ÓfæS%£nAA$$$H¡©~íM	­ææfÍ¦ºÙ®vªÔç¯ºfeeëÑ=÷¨Ú Y YÉÉÉHVHÖámß¾=***ÇÀSO=õ¿üez'ë¯ýë¥K^à1ð£ýè/¾öÉúw÷w_ðøñL²@²¬d¬$+$+$+É YIVHVHV@²¬dd%Y$+É YIV¦u²¶··/^¼Ø`0ÄÇÇ744¬õH ÖÔÔÈ<Ëµàæg?ûÉ YÇçØ±cv»ýo¾ñÎ+ø_HVH|<xPV]Cüâ¿ f¬ãsâÄyóæñ;+d4d¬$&&¦½½]Î=»|ùr@²>æææøøx©V.]Ú××G²HVf¬$+$+$+É YIVHVHV@²¬dd%Y$+É YIVHVHV@²¬¬¬$+d%Y$+ÉÉÉJ²HV0åª««333ûûûIVð@ºººObbbww7ÉJ²&®¼¼<tPQQÑt,XIVÀã¢¥¥%99YJU)X¥l¾ÉxÄW^^Éd²X,ÕÕÕÓýÏ!YREEÙlp:½½½3à/"YFgggzzºOrrrSSÓù»HVÀÃÖßß¿wï^ÉYZZ:Ãþ:ðP>:!!AJÕììì÷¬IVYYét:srrÊËËõÛ%G7mÚ`³ÙêêêfêO²&T¢¥°°°¨¨HjÓôôt5,µ¤¤$<<Üh4æççOÓª$+àa«¨¨Õ&Nr¹))¿ýíoåRTmkkñ@²&MVVÔ¦ÚªÔ¦[¶lñóóUIVÀÃ^[[«ëêêìv»¿¿xxøÌ¨J²¶ÜÜÜÌÌÌ®®®ìììÄÄÄM69¯j0iº»»#""çÌóÑGýË¿üKddäLd<< R¤úøøDGG§¦¦¶´´x[;¬¥¦Ô73 *Éxª««­V«7T%YS«³³Óápøøø¤¦¦zÃ@U0U¤6-((Sê¬$+<Tõõõ6-  `¦N©O²¬ðôöö:N£ÑpúôidL¹Åb1L;vì §É¸ÔÔT5¥~gg'B²&NTµZ­Þ3¥>ÉJ²À¨¯¯OLLÈÏÏ÷ª)õgx²=váÂÁn·777¬ðtww«)õSSS;::h¬111êù<x0..d©VTTn6+++é©4U/((H.ÿßjÒgþðÎ9£ÿ2PÕ+µ±±qÃ²ðÎ			$+<©MóóóM&$+U½"YoÞ¼ép8nÝºÅÑ`tÕÕÕ±±±EEEþõd½víÚúõëûúúè«££###ÃÇÇG.9üë-ÉZ__¿|ùòë×¯3ê&Rßd2Y,ÚÚZÄ5::ÚGdwüøñØØØ­[·2PÕë"`õôôlÜ¸Ñh4¦¤¤455Ñ $+ÉWRR¢ªÓSd%Y`âÎ9ìããÅú$+É×ÛÛk4ív;=HVHii©ÙlXÍËËãð/ÉJ²ÀÄutt¨3ª¦¤¤0¥>ÉJ²ÀÄióZ,Î¨J²¬ð@êëëm6[@@Óé|ªþógÏí;è'øë_ÿJÛ¬à]zzzÔUp êgæãã#ºxñâÈ²¿¿?-L²)..)õýüüôQ*««Ýn§IVù¤<MJJäs8]]]rroüãõ[BBB­M²ÀLær¹Ng@@Íf;~üø$Þ³$«Û«ò@´9É3VuuµÕj5ùùù>PUU¢tô- Y`èêêr8u©©©mmmSñ/¿ü²R©ûì3Ymmm¥ñIVQÔ@ÕÐÐÐÈÈÈÒÒÒ),®¯¯¯ä+íO²À¢TÍÎÎîéé¡AHV&hªd%Yx»ÒÒÒI¨dà½´)õ'q *HV7r¹©O²¬0	Ô@ÕR$+ÉÀÛ=ª YIVÞ¢¨¨èáTÉJ²á´)õò@Õ5kÖØl¶äää©"d%YàaÓªJ¼ÕÕÕ=Ì®­­/((hkkNHHÈÌÌä?B²¬¦1É6«Õj2I½(]RR¢­öööÍfyJü_HVÀôÓÕÕ%¢ªÉÉÉ¤§ÊQ·8w:üwHVÀ4STT$uª[yyùT?VFFÍfKIIÙ»w¯¶ÝårEFFº%ë¦MvìØÁ?d%YLRJÂ©)õÂJÅÅÅjlAAv­ËúÕîîîÐÐP&%&YIVÓT[·n5g'½äÃÃÃõ½¢:;;%;åR­¶´´HînÜ¸±¶¶¶¤¤Dr7??ÿÉJ²T½(©¶wïÞÖSI3!!Ám£Ãá¨¬¬Ô×©N§3--mÍ5úÞL YIV©¬¬¬I/­X|hÉj·ÛÝ6fddè$+É`:Qs*Iµ:¹Sê»ûömÙ²¥¬¬lÝF:ÜÑÑÁ¿d%YL3§ONHH0Lyyy;¥¾$å¬Y³F£¶Á`kmmiç	W­ÍfÓwYÉJ²´Ã¿~øWªUÕÄÄDYP«Ï<óë(7©¯¯OOOMIIÉJ²N¤F4ÍR&JMEO¥ûöIµªbUr???)i|d0£tttHQ8ÕSê;N«Õê¶Qu9æ_@²¬f©MM&SRRçµcgggIIÜ°ººÚóêVjbÁ ¯YÏ?ïëë;ÊO­ YIVÓD©ÝnªqKKKÃÃÃ³²²òòòÔ¼ú°Üpþüù.]R±:wîÜþ$+É`ÚëîîÞ´iS@@@jjê¸z*IKkÕ­äqzzº¤¬74óóó;jÕjµªÉJ²Æ###-KEEÅx*mÝº577W¿¥­­Íl6ëNZ[[÷íÛÇA`d0íI­$¥ªÓéï@Õ©/¥ÒÚáÈd2Ñ¶$+ÉÀ»¸IS5Pu¼ÓÕÕÕ©·þþþ~~~ßûÞ÷ô×VVV&&&ÒÂ$+ÉÀkSê÷¶RªÎ5kÑ¢EêÑßÿþ÷>>>Røª·¾¾Þb±Lîô YIV¯ÎÎÎ´´4ÉÂ¬¬¬QuÝºusçÎÕoÙ¼y³Ô¾ááá©f³¹¸¸v&YIV3¨*ùg·Û¥²ðý<ûì³®nåþ»»»igdà?n³ÙL&«çMµlÙ²äädýÓ§OûûûÓÈ$+ÉÀ+ôöö:N£ÑÖÖÖ6áûÉÊÊ?¾Ùl~æg¤B­ªªRÛ%§ec||<MM²¬f¾ââb=«Õ*5ëïD²3***88Xúý÷ß|dõññyî¹çusdõÔõë×£££IVS¤££CÔTu³jÕ*UýÔH-3gÎºuëä*ÉÚ<¼u444Øl6ùÆ§m)bÙ²e©©©ü/ïô ]Ôá_ÉpæÌ2O=õÜ¡~7Éú%ëÊ+/^¼¨%ë7ß|<Á`X±bÿKÀÛHZ,ððð7w®Áêêj«Õj6'ëª¬o¾ù¦~KOOÉJ²>¿³êkVyyyv»]õ3êêêJKK[³f·íììt8êªxø×ÍªU«$æõ|¥<-ü¿HVÀãèÌ3R¦§§ÿÓ?ýSpp°þ<3ªÛmKKËwRRR"Qg³Ùjkk'ýÊÓ		*++;zôhrr²¾o0HVÀc¤¼¼³  àøñã¿ýíolÑïé¶ÅMbbb@@@nnîÔõ$êéé@|5óçÏ|åG²>h²Jþîw¿S/V½ìvVVÖ#G¢££.²ðDoo¯¥¤©O<ñ6T«òaªï¾2R*÷-êp8d *ðÈuûöíj!!!AjjjdùÊ+²HVcjii±Z­iiiyyy-ÒgF¾¯ÇÇÇËGZ­®®¶°¨*´i¬òÂUªT²²2¹¼zõªJÖf0IÇÓ§OËelllQQ?üáµN¼»K.àÜ´iÄêÐUÊÓ¤¤$ùüÉÉÉÜJÀCJÖ÷î©ú2,,L%ë+¯¼"[9¢¯YIVÃêêêJNNâR.çÍ§Åagg§ä¨õZCBBþùÿ9;;;??ßí´©À²QT)õG¬ÚÛ;wÊ¼¦,Y¢%«Ô¯²`0^|ñE	Q)RN§ªJÿð?H¦§§k;HRÆßÿþ÷¢ååå6-//oØ»âUrÝ»wïdTq²[³r4À($,õ§ijjúîw¿;gÎíÄ¨ÝÝÝòùòåÀ©©©Ãv6333  `ÂgT¦GÍªR¶¯¯d0ÂÂB·4--MêÎÃÿ$ä7néæRJ*µ,1óõÀj®¥fìÔo¹víÚìÙ³ÃÂÂÔ¡Ýí¶´´$&&FÉfz*aF%ëç®üJ Ê|»eù¹yóæª¨YõÙgI"Jj%Dåë¸ÊÚ®®®2µ§§G>jäs&%%¥©©ÄLKÖ»wïª|=zôèÀÀv¿¯/½ôÉÀÍÿüg???û¬V«T² a9úo¥¥¥¥RÎJÓS	33YE__¼=¤HåúúzY>tè´´¬ÜÈ¯¯ïÿøGYVG¹äóA>:ÊËËO>=JXvtt¤¦¦ª3ªr"qÌäd½ví¼1$;µ-ÍÍÍ²åÈ#²ÜÝÝ­kõ+Éx³ÅËÂ_ÿúWmËË/¿,[F¿6PUJ[ÿb'ëÝ»wÖ¯¨ÚË/«*d8´ýì³ÏFº³6-44Ã¿ðõQ!YéÈl6ûùùé·´¶¶J²ÊåÐzz´)õ¨d0?üPrtÙ²eÚ)a=,ªÔ©¥ººv7&ë©S§ªªªHVczî¹çT'&©C755%%%1PÔ¬f#ßFµååËgR=;F²Þãí·ß^°`ADDÄ¢E¾úê+·k%Jzê)É$ûüùÏÖ¶»§ÓÊUá½ÉZSSãp8*++µy=*ÑÑÑ©Z²677¬w½¤æO~òÁàçç×ØØ8æ­ª««­Vkdddii)m¯NÖ÷îÉÉ»èüùóúúõÕW_Õ¯jó1¬À¯Vå-¯:ÿ;ßùNhhè(7éèèPçuÎÎÎf *HÖ¿6Yãââô5«6kÉÌlRªZ,ýªªªF¬jUm6[]]­õÿ$ë¹sçä½qõêUY=|ø05+à""",Yâ¶qØde *HÖ©VÅ7T®RîÜ¹#ë+¯¼¢Ndf<ÕY³fé·8ýªdÛär`` ,,ìõ×_w»êúõëºf¶ÆÆÆç^>ÿ__ßÈÈHÕ%øµ×^Õµk×jª)õ¨uD·oßN9©YsssÕ±b©-[¦¨J*Ô¹üÉO~¢vkkkÓ¦Ôg *HÖÑÜºuËm`«-3E^BuµZSS#|íÚµ?ýéOj£6P)õA²z:ð&~Øª¾±VRcÚHV`;w®ÑhÔoùÍo~£ï²¤T-..¦§HÖñÿ|¤Õ­¨%Y$(((&&Æm£JÖ®®.ªd7U­ª¡«ÚèÕÐÐP·-dfK.½ÿþûo¾ùfDDÁ`Ð_µjÕ*yË«)õ¨uÜîÜ¹Ã¹no³eË__ß¨¨¨ùóç«nJßýîw¯òõý]¥5ùùùþÉ:q÷îÝ·6äæÖ­[²ZRRB²3LYYßÞ½Õjkk«Ô¬êÐJYÁú Y'!YäítöìY²NÝ©åHVàá»4(>>~åÊnY;kÖ¬ÌÌLÉ4oÞ<¦ÔÉ:ig«©©9qâDFFFeeåÔdL²üÉ'Èò=HV`úÖ©³gÏö$éßÿýßë¯íìì÷x@@ÀÆ¨urUU¨íííÚÏ®7oÞ-´Ô¬Àt×ÚÚ**_].¬K¸JÖ~=8¥þÖ­[dË3gh+¬¬;wî=rä,ÈÂîÝ»eÖ7X?A?É<þ$2wìØáp8222,Ë~ðýªÔÕÕÙív£Ñ(Õjzz:uÒõîÝ»/¾ø¢¼Ó®^½ªË[Ñí@ñHV`ºÚ4)))55µ²²²ºº:00Ðl6ëófgg«J©R­®ZµFÉ:ÉªËåÉ'Õªª_õãY9?+0äååIª?¾ÓéT«ÅÅÅ&ÉÏÏïí·ßúõÒ¥K´0ÉzåÊýIX­Y9?+ðzíµ×öÙEôÑGúíR°>Z[L56M¾"ËUR§ÆäädªÕ->©YÇßµk×fÏ­N÷&_åM§]è6õé§VÇ~Õ,ÁQQQª7¬Ô¬ÀcÎb±HLjr«ªªäêp8ÔjVVVnn®¶smmíO<áïï/·JHHÈÎÎ&VjVÓÙÙ©:%Iµºoß>ýUK,	ÕvZvëÖ­ÍÍÍòFóóó5kÖñãÇi@à1ªYÛÛÛïÝ»G²Óé¼fMZZ¼+ÿíßþMí¯~õ+ýÄú?üá%%V¥N/Ç4 ðõÀÚ9YÝjÖ¹lhh YGbÇ6­»»[­J^FOmèèèµ'i ïþ<dàÜ¿ÿä¦$+ð víÚ"u§dd``àÿøGíªýìgò]´hÑ×½,Y"«ûöíÍÎÎýívSSm<Ê³È[¬J¦®[·ÎårùåªHÕ=ýÑ~äs¿¿ÿîÝ»KJJ,Éd>JÀ£LÖÆÆÆ9UÞ§ÕÔü²L²T««ÚªÍf~ùåµ-RæççËû÷«¯¾jkkKIIQSêkG<²dp;,QºsçNjVàá|ûí·ËËË¥`Õ×EEEsæÌQ«ááá²O^^³v»)õÇ%YíÍD²ÅbQvÕÈ%2õ×>óÌ3R&%%Iýj6O>][[k2´¹¬þ&..ÎÏÏ¯ªªJ­Ê;ñ¯ý«Z­««Äýè£¤0mjjjkkKKK ËÎÎNZxìõÞ½òÞ¼yóÐd½÷.É<ò¾Û½·¶zéÒ%5áºuëV¬X!±*_.??_êT)[ËËËi7àñ­YõiªÎÝ¨z0©e"[¶lyã7ä½ævUtttppp||üsÏ=·k×.ÙR__o³Ù$n³³³é©L³d£ÁÍÍÍòmÚ`0È'Â©S§HV`X.+**ÊÏÏoþüù!!!ò¾Û¶m~)L-[¦µªT¼.Y¥Ø-++=ö¬^½dµhÑ¢°°0mª|U3ªU)deUýìZZZZTTDO%À5""Bã¹÷nttô7ß|cB¾z«¯R^^ o«Õêëë[WW§]õÕW_ùw¨¼×®]ÛÑÑªÆwuuÑ&«|õv[.bÙ²eòyÁÿ^¥  Àb±¨sÎ=zTB4%%E¿CrrrTTT\³üq^^d°Ü¤ººÖfT²ö÷÷ë®üýýµåÀÀ@ÃUTTHLJ^Úl6y;èÏ9#Éj6õ©9wîÈl¢6  ÀétöööÒÀtJÖ'Nddd¨>À			Z`myÿþý²ª/CÇ4oÞ<5VG.ed×RsÿJ%ZXX¸téRy+mÙ²E»V×h4¾õÖ[j5++K¾>ZÞz²gjjj[[mL¿d½sçÎ¤¨Y»v­ä±,ÈåH¬Á$ËËËåRN-J;;;¿ûÝïJÐ¶¶¶ª-.köìÙ²Eõ333CCC###KKKiF`º&ëThhhoßf³ùìÙ³$+¼ÄgRRÝn|úé§ýüüô$Éö9sæ¼ùæjõÌ3£Û·o·CnnîâÅ¥TÍÎÎæªÉÊLÀ×Rö÷÷ÇÆÆ:N50¦ªªjÖ¬YV«Uû¡´¥¥E¾qÆÇÇËnááá² TµÙlú®ÂfN²Ê·æ^xd<ár¹ÕÉÉå2((èÚµkê*LÙòÔSOiyùå_Ê_þò¥[·nU¨Ï@U`&'ë¶mÛô[nß¾M²Ã4,,ìôéÓ_ª"õ¹çÓ®(ýéO*á*Ej```rr²ºªÀMÖúúú©À·ú%K´³ ÈyHpsôèQ???m¥¦¦¦èèhRm¡»sçÎÄW½´)õ¨ÌÌd½sçþ´çÈÈÈàh00º?ýéORÎ?_¿QÊPÁ°gÏ¯KÍ¢?Ì«M©Ï@U`Æ&ëÐsÉ:uª»»[úúúHVÀÍW_¥fÏW''U­¥l:5999222--M;#MWWWff&SêÞ¬>Ôò±cÇdùüùó$+ '%©ÉdUäm2gÎíZyaÏ5«³³SjSý¯§²gè ¦Ô¼%YïÜ¹#j¦ªª*-säâÅ$+ ~ùË_JIªõþ¿ùÍoämõÌ3ÏH![VV¦¿§IIIT¼(YoÜ¸qèÐ¡°°°ÆÆFµåÈ#ò)pîÜ9Yîëës:²(¬ðBRbæååÙl6)UFãO<á¶C@@À÷¿ÿÕ+V¨NÂËå·UïJÖë×¯¨	[ZZ$>¯^½*9úé§êw f×Z³fMrr²Â×n·KÍZQQ¡ßAêÔûö¹ÝJM©/IÌ@UÀëjV!iZ__âÄ	ÉTù-%¬,twwK JôÊò=HVx'©5-¼/ÔêüÇÈ;BWm¸¸8·ãÃL©x²ÊGÀÑ£GU²ªT%vIÖsçÎÉÂ«¯¾J²Â9Ný_ýêWòÄUó.íÞ½[]%µé;$wÍf3SêÞ¬êgTùJ®OV¥¤¤Dõí|ðÉï¥ªyöÙgõWI|~ç;ßIIIj5--í«¯¾RÛëëëccc¨¬ÿç°u³vÐêÕ«e9,,Q7ð6RÊÿùçßµkWdd¤,¯R»vïÞ½úýzz´ªgÎ¡sÝÿK¢©©©Ñ¶<ùä²Eõøñã¹¹¹µ---ZýZ\l±X¨¬#:wîÜ'´ß_åódW=¶ÛFy/¼ôÒKiii7nÔ¦O|MII«¤`eJdõh&YÐRd÷$kPPÐÐdmllÔV].WAAUÕ£óÞÈ'HCCZ½xñ¢êÜD²bÆûùÏn4åe_^^®]#[´ÕÚÚZI_ÉÄ@UdÖ¡§b¼ÉÇÙl		Ùµk +V¬P½~úéU«VÍ=[×¯_ÿõàU322¤TMIIÑ~j@²6oðæÍ[ÃA²bæikkkjjÊÉÉ	ÔÏè+*ñi0üüü¤ÄUU¥¨,..¦THÖ1ÜºuKM¡];íùÇ¼téRõãkss³þL®$+¦¯3gÎX,ØØØ¤¤$Ïøøxýµ.]²U¿sbb¢dí¦M¨¬öZºpá:?«Z]¼x±ºªªªJVÕPW³Ù,L²búV¨÷îÒóßÿýßåÅ,ªô´ÛíRhJýªUr4++K2U2X?Õ>uÜAûÎ;ï¨åÈêÍ79i­°°0<<ÎÜÜÜèèhm*`§Ói0âââ´W­Z%;JL&¹-uâ._¾¬l#Ë6Ljkk%GsrrÞ~ûmÅ¢M¿qãÆüà²]ÛST^êªÓÊ+ýüü/^,[Ö¬YÓÑÑAK$ë¬/¼ð~5!!dÅ´%i*åfQQÑÜ¹söY­H-((k­V«¶sKKËìÙ³CCCåRJX¹VfHÖMÖS§NI^¹rE¬$+¦âââØØØîînµ*_.²FrÔd2Í5K­öôô$''ËµR°Úíö)gµ@²NÜáÃ%GU'¦.ÈjIIÉÔMÃD²br?~Ñ¢EÁÁÁRÎ3gÛ¶mÚUÿøÿ myÿý÷ýýýSRRÖ¬Y#EjvvöêÕ«%SR Y'çÌçUUUCO³aÃf7ÄãïôéÓ!&&¦¬¬L^ÉÚ¼áááE«Y­VëÞ½kkk+**ÞxãÙ?22)õsÝÿ#f¶ºuëÖï|ç;óçÏ×¶¬[·Îd2Ùív©J%GU'''Ë7H©Yõ3E YIVx»àà`©>µÕÞÞÞ'|RÊVÉK5¾Ô¬mÎ9Ó;(77×h4Úl6ªÚd½÷®|åWHVL_		)++ÓoßµkW@@@dd¤djjjª~ßÚÚZ«Õ*±ÊúF²ª^KçÎÓo¼û6ÉÇÍk¯½æçççs«¾C¯ÙlNLLt»6¥~ZZ6¼&9Yëëëµé+++å*44Tô³ëOÚJ²âq°÷nyM.[¶ìÚµk²ºhÑ"Y3gÎo¾¹uëÖ¹sçÎ=ûÒ¥Kú¨JúãÆ0ùÉ:00 -×ÔÔ¨¹/^¼83¬,&I?¡È4æA+V¬ÐÇª|TSêçääÐS	À'«ÛoÔùY÷ïß?EÓ¬~~~ûöíÓoùÕ¯~¥Mþ 4ÄMNNnjj¢Ý<ÔdÝ¼y³¤éÆ+++*ÉòòåËIV<VD©~Ë²eË¤Õo)..VSêÐS	ÀÃNV·S­´	ív;ÉG®¬¬,<<Ü××WVuZò¯¾úJ]ÕØØ(Û%j[[[JJ¨ÚÙÙIÓxØÉzêÔ)ÉÑù$joo¬S1)?ÉñÆªdçË/¿|éÒ%5¡ú.9H¢££e7©M¥B:Õb±hs0ÀCMÖúúzUÊÉÇVXXØºuëô[æÏ/ñùì -[¶|=8PUM©ït:i4 Y¯_¿ÞÝÝ­E,ÉÇÛùgÞÿý¨¨(µ,/ãÌÌLÉTÃÁ@U¸fÕ¯Z²FGGÇÅÅ$SSSIV<Zþþþ­­­ú-R§Íæ¯*©¹¨àñJVurÖ«W¯Ê²|`I²Þ»woÛ¶m²qÏ=$+-«Õªs_ê×Ù³g?ÿüóòµOJÕ72PÀc¬z¬ò)¦Õ[IV<~ú©¼Ü-[¶oß¾ààà_~yÕªUAÔÀÀÀ¦Ô0UêÔÌÌLæ`Â£%9*/E)IÕüÀ?ýéO³³³öY©_ÃÂÂBCCwìØáöã+<¦ÉÊYäðÈ=ùä¦555juÝºu²úÞïÉw>YÈÈÈèêê¢¬$+<%ñé6ÌÆ`0øúúÚl¶ÊÊJÚÉJ²bÜÉª-·µµ%''K¬úûûsøÉJ²bÉº~ýúþþþIÖÀÀÀððpZÉJ²b|º»»%PÕï¬r)iºwï^õ;ë®]»h^¬×¯_&Y1.¥¥¥ê«aaaóæÍS3kµ	÷Àëµ¡¡Áf³igÎoÊÿ)OkjjòóóM&STTTddäÎ;,Y"¯x­0xc²®òâÅúdýCÈg(É¯§Ñ/(((..÷Ýwå;YhhhQQQFF Yÿwº	c½½½ÉÉÉv»=77wíÚµòñ÷÷w8Ú@UÛììlÉJ²Â#kÖ¬Ú´´´4222   ((H?ÿ$îÖ­[i(^¬>÷¬ð¤éý×©)õNçk¯½6oÞ¼ý×U×9s&<<i(Ô¬$+ÆvéÒ¥àà`ÉÔÄÄÄ¦¦&Ù"Åë÷¿ÿý'xBJÕìììÐÐÐÉJ²blÕÕÕV«ÕÏÏïwÞ@Õ¶'%%½ñÆEEEÀÉÊL[WWÃáo]RæççÇÆÆªã½.KJU»ÝÎäHVc«©©¹råT¢¡¡¡6­®®Nm/..4Íááá)))ÝÝÝ´dÅhæÎë£#¥ªþð¯"J©d%Y16©P%MUO%¹ôõõ¥Y¬$+&¢±±QbuÎ9¥ººZmdú_$+Éèèèxê©§$GNgoo¯¾;w.íd%Yá©þþ~5¥þÓO?-ÉzåÊýµ³fÍí´dG?®R_U®r5ÞIö¬$+Æ¦TÕO©ÿé§Ê___ÉZY×.md%Y1m ªÔ¬nW]¹reÉ%R¹>óÌ3´´dÅhFc~~þÐª@²¬ðËår:©©©Ìñd%Yñ@***¬Vkdddiié°×nÙ²eß¾Ì¬d%Y1ÎÎÎ5OaOOÛµçÏ		1»ÆA§O¦Ñ¬$+Ñßß¿cÇÉ¤RßMhhèüùóµRuÅ².]¢õ¬$+þ3gÎÄÆÆJpÔS©µµÕÏÏÏ-G¥lÝµkd%Yñ?º»»³²²TO¥Qö,++èuÛhµZåM3 YIVüMII:jiiéjÎ?ïëë+ÚËe0öíÛGK YIVo×ÒÒ$¥jnn®ç]|ãããÃÂÂT¸öôô<óÌ3²Ja$+ÉêÕ¤645¬gÎsÙgãÆéééryâÄùóç«éýüüæÎÛÚÚJ YIVïUYY©z*íÝ»×9JJJÌfs~~þñãÇ###¥fùå´'dõ^ÝÝÝRw4PuX²dp½¾~5LrW´'dõjjJ«ÕZ[[ëù­ª««%Ý6Ê)|iR$+Éê¥êêêFcNNNooï¸n+Éá¶Ñáp¬HVÕõôôH$''»M©ßÒÒRPP°qãÆÂÂÂQíÊU&IÛ©Gö$+É:IYY^^îÖSI¶ÈU¹¹¹ÅÅÅRZ­ÖÎÎÎîgÇj´«¤©¦_"æ@²¬^DêÑ)U¥$ZJJÜê§ÑÏÏÏýGé´´´ØØØÔÔTIeZÉJ²zËµuëVÉ$I9Ò@Õ¬¬,·[I	ËlHVÿÇñãÇ-Ô££T-,,ÌÍÍuÛ(7d dÅÿPt8RÙû·¶¶Öf³é£Wª[³ÙL3 YIVüMQQäâU;;;KJJ¤`­®®þÉO~²fÍ5YDKKÝn2@²¬ÞNjÍÉ7z©*¡*i*ÊMþó[,¹ÌÏÏ§1¬$«WÍÎÎHIIq¨:lµ*ñ©Û!33Suîîîödê` YIÖ¬¢¢Â<ÈÃJJJ¤ZÕoQ³ÓÉJ²z;©>µ)õ=§ÎÀ@²Â]ÿÖ­[¥ÐLLLÔOòàÚÚZ»ÝNg`$+ÉÿQWWk2&ö³hrr²ÖXbUV: YIVoÔÕÕ¥ªÊ¥,Oø~$SÓÓÓ¥äLjµ¨¨¶@²¬^§¸¸8<<ÕÉ:eÔ»ü¶d%Y½QRRª999ôàd¸ÞÞ^IS5¥~SS$+É:qµµµf³944´¨¨	d%Y'®­­MTÍÈÈå$äãrúôéìììU«VmÝºCÊ@²z©MwìØa4-ËèSê4¾¯¯oLLLrrr```XXr$+É:UWWÛívU§ÓéùJcÚ·oÄªU)X£¢¢$eip$+É:cI©¦ÔOLLliiÜ;_¶lÙsÏ=§ßrôèQÉJ²ÎLÅÅÅ¡¡¡¥¥¥SÑSéÙg]·nÛF©biy$+É:Ó9s&99ÙÇÇ'33óAæTÄêÜ¹sõ[Þ|óÍàà`ÚÉúø&ëÙ³g.ìvss3É:&5P5  ÀjµÖÕÕMécõôôÌ5kÑ¢E.]Õ½÷ÊãnÝºÿõñMÖõü<'ÿß¿øÅ/HV¥¢¢Âb±L¦üüüIì©4/¿ü2**Ê××W2Õßß?++ÿuÚúæo|³bÅ/ÿGvuu©ª©©©mmmùÑ].Wkk+o'NÉÚØØ¸aÃYøbýìgÔ¬f³YªÕòòr^Ó@²íæÍãÖ­[üÎ:ãÇ?Ã¿é¬Ú1^µzíÚµõë×÷õõÑ7@²>¨úúúåË_¿~Q7uDGGë*¬"$+ÉÉÉJ²HVd¬$ë4TYYét:srryHVõAeddØl¶ÂÂÂ¢¢¢ôôôþþ~HVLjRRËåR«²ÂÙËd%Y'Hr´®®N¿¥¾¾^²a·ÛÝÎÞÑÑa³Ùh Y1kÖ¬ÉËËÓoÉÏÏw8´¬ÈÈÈÞÞ^ËUXX(«MMM´¬ ôôôðA©©©µ´	¬$+d%Y$+ÉÉÉJ²HV@²¬¬$+d%Y$+ÉÉÉJ²HVd¬$+d%Y YIVÉJ²HVd¬$+d%Y YIVÉJ²HVd¬$+$+$+É YIVÉúØ%kûâÅC|||CCÉ YjMM,È³ Y$ëä8vìÝnÕCX­ÖÔÔTþÕ#!!!>>>$Y$ëä8qâÄ¼yó8 YÇÍç>·íd¬$&&¦½½]Î=»|ùr@²>æææøøx©V.]Ú××G²HVf¬$+$+$+É YIVHVHV@²¬dd%Y$+É YIVHVHÖ	yõÕWc&Õj]°`A<n®ùóçÓúÞ÷¾g±Xhÿûß§¹ÆEKvð¼¹ä-9¹÷i2¦²^¾|ùíùçdý<óÿðaaa´~ýë_ûøøÐzë­·¤¹>úè#ÂÛ·oæzûí·iOìÛ·Oë7¿ùÍäÞígÖßß?½u*¼ñÆK,ùÙ½÷O>I;x¨ªªJÞÌ´¥¹®]»FSx¢§§Gë/ùMáÿþïÿæª©©y8G²¬$+ÉJ²¬$+ÉJ²¬$+ÉJ²¬$+ÉJ²¬ YIVdÉJ²¬$+ÉJ²ÇGôòË/ó²óÐ~úÓÒ:yòdll,íà¡¶¶6i.ËESx¢¯¯O«½½¦ðÄ½÷¤¹þó?ÿd`ú!Y Y Y7ÍÍÍf³Ù`0ÄÇÇ:uÂ°Î=»páBi%»Ý.-F»yâÄZÇ%kwîÜY¿~``àêëëi®1I+Ùl6Õ>´ØH®_¿=Ê§ýT·÷&«Ãá(++=ö¬^½×â°bbbÔ¬£ÝÆ400 ßE´d¥¹Fñî»ï~ðÁ÷îÝÀ°Z­4×"""®"r©J¾sÈ÷·ü¡­4ÕíæãÍ¯QyKËÂÝ»wõßn0   ÚmLÛ¶mSºò2çÏç]é9ùþ¡F%É¥ú.BµråÊ/êuh+Mu»yo²a1¬ÆÆÆ6Ðn£J"!!AÞ±Ú»æý=¸k×.ùÆ&!ÑÚÚJs©¹¹YK^]r)oIZl´lÓ%ëÐVêvóÞdõ÷÷×y!âæÍãÖ­[´Ûè222>ÿüsý»æý=XRR".o$4×/^¬ª|X5Å-æI²m¥©n7ïMÖyóæÝ½We^#¹víÚúõëûúúh7OÞÌz4×ïA·ºæ³Ê§Å&¬C[iªÛÍuíÚµû÷ï¹â°êëë/_~ýúuÚmbïjk6m:tè,´··'%%Ñ:UMºtîÜ9©_i1uh+Mu»yo²644DEEùûûÍæ³gÏòBVtt´[F»ë]MsâÆR%$$xæSWWª´2-æa²m¥©n7fddd$+$+$+ Y Y Y YÉÉ`òÞÆ>>/vª¬¬<pàlùâ/FÙ_[fêüí¯¿þºZ½uë¬ªÓ© YåÄ$D%íZ[[åRKGÕ¶Øl6ÞÌAG©©©^xámË~([nÜ¸¡Ý¤¡¡A¶h§Q÷YUUEû$+0ÓÜ¹sG_V¶´´Èecc£ZP)(¬v»rå¬^½zU..]ª6êÏ¹«ªX§Óé¶EX¥¸V¯R?ùäYÞ³gÿdf¾¾>µpþüyI¸k×®Éeû±cÇdÁår¹%«ÙlÂTåkHHT¨² %i||¼*XËÊÊdË¹sçÜª^¹O-ÅoÞ¼©¿O$+0s¨RUòõÐ¡Cª¾TÇxoÜ¸á¬ZªÎ­«WoL«ßyçÎ²QBWUïÞ½[¶¨$ÃóHV`æ¸~ýúæÍ%á<¨¢Q*NI;Y*sh²Êêç­ê¾÷Þ²ñÂQQQ«W¯ýß½÷Å_TÕm322ÜBZîÿ@²3ÊÑ£Gµ.EêwÖ=öÈÂT²;vLí)ñ)×ªãÆê'RíGVuøòåËnÁ)°<yR+v¥TÕº;©@²3ÊHÃ¡Â¯««KE ¥­²påÊ­Uû¨ße­VëAªZ°¼wïÜJÕþZA<´fUW YBýÔzöìY¹üøãµÀÛ¶mÛ´UË¥ÝDÅjeeåÐ»êëëSÝÆ'5+@²^ð¾õñÉÉÉQ.CHpZ,míT¹VV×®]«¤>&cbbjVd¼:ðÛßß/Ë§NSõ¨ldU'Õ§öS«êÍ¤êÑÖÖV¹ÝÔ!_)XG	ojVdf>Õ;IßçèÓO?-ÝÝÝßÞQ¬âPt÷Æj£¤©¬¸²Eây¼5kû(©¬¦mÂ;wîH"ÆÇÇ×××y«ªª*UæJ"Êþ,HMMUW¹¹*,,lh²8p@M (û»Õ¬999jº	þ#Éx¸¸¸²²2mUMÕôî»ïj[nÞ¼éV¶ªÉû÷ï§0HVHVHV@²@²@²@²¬¬¬ddddòÿrò-0ÇÙLIEND®B`


¶÷ïß×êëh¾Õ'O|~4ì¹¡»»[®ÜØØhÌéíí9þïB|~PÖWeÕ·MÆ#]lLSVPV`þXýB¤¬2nÓüèÐç­·ÞÒR¿îÛ·ÏxõWóæ=üÈ¼Ì×¼ùÇO@(eõ!Ãk¹C]*Ó¯3>>n1Î¯ýýýòõÜ¹sþëTµ[ò­çM###eâÁºÑZB.G	8dw»Ý>óå¡e¾> ?<*ÛFYõm>ÿ»wïêÛRVPV`¾Èx1==Ýg¦OYùÎéJàçßm_ÔWð[·niZÏmt#6Æä.øÐ¡Cþ74F>eÒVºõ÷éÓ§AvtÒï®]»VKË§1NMMÕ­?ö¹Õµk×ªªªä'ÒH_eâ£>Ò¬êzWÿÄét|òD¾+¿qùÖ=;ÉÉÉ	^ÖK.é77o7nÜ0_²²sL(_=8uÓÒdè©+c¬æíR¨¾.¯[·N¦½^¯1:zô¨æDúÚØØpç²®RþÌª¬;vìÐé'Nh#åIJØd:à¶§§GïJ¾644oü×ëyÂºb OIf¾ÿþûº65??_ÆèºÌäÇå wn|ûïJ¼%ÆVRÝú«ãc©¸,ý7Wúé§2SwÖ+'$$Óò<ýËªÓ2rÕõÛ>ûpQVPV`¾ÈÈFwJ8fõ¡C7ÝáÈ3É¼½PÆFÓîà*×_½zµÏöT]!l&§-ë´k%KÒo«ÕªÔ­º¿ô^×»êzicí«<cä-¤pÆºnó(Ö522RÞèCÈÃµ··ë¼óØ°ayùòå¸¸8­|Õ]¬e? þPÆÊåg7zÿÜo¥úÖ­[U¹úvGßa$''k³)+(+0_Ífõ/«y¦W~ÝIU·>Ê+õÆIæQ¯ÿgZYÝ dz×®]Ï¿ÛéÿQËË*$&&×ÄJ$`:FWò]éqQú'£vÚº°	cÃj||¼ÏHW¾%s1¡¿¤TÞ[èòùäÆWc@/	÷Y&>:àÇi·B[ËìØ±Ëª?*ËOªã]yþZh|ë8 ¬ÀÜz©ñÉcå¤±6Øç#+:¶Ó²J·¤ºV×1ÓÍ>+'UP>WÐýn¾úê«³#?Ý«3ÆÖ2Xôë¨Ý^Yz©CpùªKÉØýGoG$ºES·eÊërnj5ôÇ?þ±ùâ¶mÛ|®£«t¯fE¼¬Æ§¡®]Ð0ëwÆ¬ ¬ÀÜø¢|m°UWÛÊ¾î$¾öövÝªªëQýLWV2?~üEÊª#<Í¼®X6Þé|ãt]´>®ñyV·Û­ëuõGîììÔÇÒà6JMMõðú9%ó¾Ê>6®#Ï6`MCY,>¬÷lìWl²²/C(eÕèááaYj4K÷ò¤y;«Ï±w¨?ó^¬¡Õüy=¤ÈÍ+èÊX]å«ûîêy´FYuÝË/K>Nç®]»t$­mÕ±àTûýÜ³7à5õ³¼¡§nveÕBë1|&AYAYA7NUVãp?òJ-áÑÐ5ÕýWÍW6Æ1åNÞ¿Ó¸¾ÇãÙ¹s§fXæëÎ´ºS±±W­Ë[éþ½z_ÿM¶úAUóù[Æ5++ëùwtÐÃ+emii	òÓé!&ç¦N9º15x¼ùÅf<í©Êj,a_UUEYAYJ64UY?~|ëÖ-ãâÐÐÄÆç5Úc0ûûìÑ£û.ù|lÔçJ3¥Q2äÝ²eKÍÀæ]D»Õj5?7y0Õa·Ø|S-aóQ¥¦öìñ?ÃAÀûWVzé*£9O]`þOè(+0çÎ[½zõ2ý×5éÖ­[wàÀÝßÙáVÞ¯ù·kñ´Ç[ê©ünð÷7Ï¿Û·?xPV(+ ¬PV(+ ¬PV(+ ¬PV(+PV(+PV`Î<~üXOÙýÿNËgðÊO>ïÞ¹sçþýûAÎÖ¢gÏ<CªÜó+WÌç*G_Ï ¬ÀtÊËÇl#¯Î['eçÂrÍCMu?rã>Í×6_ÌÈÈÖÖV¹Õ3gô$Þ2-»ví2ßmÄ¤ä»»wïÖç#Ó/_ÖëËÅÞÞÞ7îØ±C¯`D¨¥¥E¾k~tÐò5--m¦ÿÊÌ¼È×¤ù%Û<|Tccc:±fÍM68Ø»û×ûloo_?I/644èÕä¢Ë>|èa³¾¾>ùnMMM§ª<x W¯±'OÈtUUÕèè¨<Õj5®)9·ÙlýýýierôèQy2qîÜ¹>z(þ3(+0F)'&&äõ7&&F^Í÷ìÙ#_åâ'ä«üozYXÕÍ7ëEm¹U_ý¥ô:S´!,,L¦ÍwòôéÓÜÜn~~¾ñTe0§ÆÚc¹Oã§Óõ¢òõë¯¿¯.]Ò»e,ññqó6r5£GÅÂef¬··W×"ÊxH^vïÞ½|Ì:£²ÊK¼¼ë¸J¦U§5*gÎrDFFêOèX¸©©©þå8uêùGØ½·®,Ô³®Õa´vKF2úôióª×aFâ[þÊÌüOö»ÑÒ#Gt¢þþ~lIÝ¦IÆªã«Lë$ý_ôuL¬½|ðàAð²úDÔxtW!uð7$¯×åÊ#6ÝÝÝþÝÒç©«^¥L'?Ôýû÷gñè@Ô3(+0öïßojbb¢ÿË±±Þ2ô1«ò)ë­[·üË*åÖ1Emò¸r1''ÇféÞLºýÕ¨Åºuë¤.ze·ÛmìNuãÆãjrÏçÎº¯POOOÀn	c£¦Ñ0Ð¥áÎP=ÿ1­êÈõùä>M2­]_pm°¹¬2ÖrHbýËªYµjùä¦DÀ0YGFFÒÒÒ´¸Û¶m	æÊô§~ªk>õùÛuÏ[Øéêîi»%HcZZnNQèâõ>CþìAYù"©ÚcÇÉWóÀtnËáñxdBhS­6ÓÑeåt×bÿÑsVV&Dµ>÷ ÖJ:¤yÓ=ou£²·³³³eìntKßaÈ;i¿Ü6ôGÅÂefLwx1?>çeá1ÓÏØHY|Ê*Zv:ú¡'Oêuzzz®]»¦Ì¯2G4F²¡71_AÇvº[ß=Èýè&IÝ·Öç3-:ÎÛºu«´êðáÃºo­ÜVwÓÝ·o¹X¡<z(þ3(+0Ã¿WÓ«­1½qãFùß¤³³S;çÔä%þÜ¹s/_¾råÎ¹÷®®ì5Z«+ßÿ#öwîÜ¹uëÝôS7ÆÄÙ³gg+÷,ýxþÝzQqúôiÝ"«òÔÆìÝ»WæÈ·V®s¤dr¹h<OÝuHWÌJû¥^Æ·äq53zôP,ügPV`¦zy¡å%X"ðº­®ªª*ÄW|#-÷ßÆ(ãO>1Ì§2ÚFDDÇ@23ßØ¢)êèè3¢®÷/óõj2æåùëJéììì=zo;ø3(+PV(+ÊcY>¼víÚüàß|óÍâ.ë/~ñ-[¶Ü`øÑ~ôõ×_/ú²þÅ_üÅïX~üãSV(+ePVÊ ¬Êe¥¬ÊJY ¬PVÊ ¬@Y)+²(+ePVÊÀ(kss³Åb¡¬Ê:ÆÇÇ²~íç¯þê¯(+²êàÁÖ²~ûí·@Þï=~Ê:½þþþÄÄÄ	cÌêõóóÿ1+²Äív_»víÛYõÅù¬õ¥¬Ê:geÌ ¬@Y9Rß%²RVe¥¬PV(+ePVÊ ¬ÊJY²(+e²@Y)+²RV(+²(+ePVÊe¥¬ÊJY²@Y ¬@Y)+²RV(+ePVÊ ¬Êe¥¬ÊJY ¬PVÊ ¬@Y)+²(+ePVÊe²RVe¥¬PV(+ePVÊ ¬Êe¥¬ÊJY ¬PVÊ ¬@Y)+²(+ePVÊe²RVe¥¬PV(+ePVÊ ¬Êe¥¬ÊJY ¬PVÊ ¬@Y)+²(+ePVÊe²RVe¥¬é555õõõQVÊx!^¯·²²ÒårY,/¾ø²¾J7nÜHHHïèè ¬°¸Ùl¶ðððôôôúúú%ÿ#/ô²®_¿^ß3g6lØ@Y`±èêêÊÉÉ±M*((Ëä_LkW®õÆ1,MMM2Hu82`aë²úñMYÛÛÛ÷îÝ+ïùyûí·)+¼r###ÕÕÕ)))ÅårUVVÊe¸GYGGGåíÏ'OXî ät:¥©RV³.ç¥±Ê:88¸gÏ¡¡!öÆ¼RVVVgg'Ëd¡ÕãñlÝºuxxOÝÀÒÕÕ%)Zn;(-ú²ÆÄÄXL(+¼rMMMiii2Hu:%%%Ëm¥E_Vy¥¤¤¤e»e¼(³e¼¨÷îéJaaaï¾ûî±cÇüQ)+ey¡;(KVW¯^ív»ÓÓÓåbuu5Ë²BeÞA©¼¼üW¿úU\1Tmkk³ÛíËáð¿²À©¬¬LJJòÙAI²zýúuó5¿øâììle600PRR"#T§ÊhÕg%ÍpPËr£¬_ÆJQQQYYYöèÓZ¹Inn.K²þOgg§±RðÓÑÔÖÖÆÆÆ-¬¯¯·ÛíÅ²þ@ z´§ÓYYYéõz§½Iyy¹ÕjMJJw8|²þ°ËåÒ£=?~FGP·µµÝ¼yã.QVXîÌ;(eeey<	e¥¬0]]]¶I²RV=êJ²RVx!ÆJ²RV=c%§fdd°û.e¥¬0KEEEÃf³åää°1²RV%¨¤T*Yõ?Ú++ËÍÍ-..¾yó&²RV±Ëå¸1µ­­Mr+×ïêîÁçÏg¹QVÊÿT©;(É× SN§XW¯^µÛíì!LY)+ü×ëÑ§q´àííêê±¬Ï@6--ªRVÊ¿¿ûvaa¡î TTTtïÞ½io"ÝMNNöYWWÇò¤¬Àòåñx$³8Úp%Ãæq­ÜÖg(+e°Ô××§¥¥Y,ÄÄÄêêêYí¡¼¼Ü8)5%%%//KY)+åE7¦ÆÇÇKS¥¬µµµ/r%¹¹ÜUTTy8e¥¬ÞÞÞØØX	aFFÆõë×Y&²ÀltvvæääHP£££edÊJXveµX,|òNlÚ´I&Z[[ããã³³³/@YÀãñ¤§§½þúëë×¯ÿû¿ÿ²JY§,ëáÃu"11Q&eº¿¿_¦ëëë­V+e°lÔÖÖ&%%Éã~ð7Þxã_þå_òóóeØÊz`Êúÿ´··K85¨:!_O>-_>|¨e]³fe°=zô¨¬¬,666<<«v»Ý|ù®ËåbAQÖÿ311¡CÕÕ«WkYwïÞ-s.`³RVËMoooqq±ÍfÊÍÍÕû|F³ÒZ	0K²ú®>räLX­ÖÍ7eñ«LDDDìÜ¹²X&nÞ¼iì $5oI²æççû®FY)k²³²6À²¢GÐ#(ú÷²­­M:Ú××gÌ©­­em0eiÌª¢¬¼=ÚCJJJð£=È(6..îâÅ·oß.++³ÙlW¯^eRÖéËZSS#3srrX`	Ð£=È8UF«æ½|½^ïTëx¿øâ	°ôDPÖ®]»¦+~%¨2+Óòlß¾gÎÑë0f°ôtuuÉàÁ6I&Ì»ûÊ`TÂi·ÛDó¸QÖÓ¾^ºti||Ü¸_éëûï¿OY,='++K©Î¢¢"ÓÑÈPÕåråççëõâÅØóçÏ³Ü(ëIGe*Óò'ÓgÏ¯À122"Á¨¼¾9ÎÊÊÊS+**ÒÒÒÌsêêêX5TòG&í4ætttÈ.Èt__®+6Æ¯À¢£§£êJÒ×à;(I}fZ­V#euUp»ÿøUjÌyðàb)+Eg`` ¨¨Èf³geey<ioR<É<§··×áp°0)+çº°¬wP*((0ï Üõë×£££oß¾mÌ$ggg³H)+e°L555eddLµR(JJJìv»**RRRfq'Xîemiiihh ¬¯êêjÝAÉårMµRèy.,,Ôm®^¯ÅKYgy°Ï?ÿÜÞºukÖ$ÝéòåËÀÂä³DeWYÖÆÆÆúúzã¸Á.]¯111ÒT£¬ÀBã³Rgg'Ë¯¾¬r_ÒÎ;wîÇ¯û÷ï7_4ÇDY,]]]Ò¨¨¨î ÌYEÀ²nØ°Á<f5AY¼ZMMMÆéhJJJØ··¬ÝÝÝqqqn·ûáÃrñÜ¹sY¼Z¿ûÝï6oÞüæoÆÄÄüêW¿2vPJJJzÁù-«nX?ÖnÛ¶M&=&ÖÝ»w§£¬^ÚÚÚ+V|ïßÛ´iÝn×)vPÂâ(«¸÷®|_½zõ|¾5<<Ì§n¼dáááo½õVaa¡î ôgögRÖ£G²d°ÐËúôéÓäIYÈUþ¬u]1eðÒèÙA"##%«EEE÷îÝo¼ñÆ;ï¼ÃÂÁ"³>yòÄç­Zàhjjr»ÝaaaòZTZZjÞA)&&fãÆ,",²NLLl¤lÕ÷õdzÝºuÀKPWW'oëucªL¯X±â¯ÿú¯ïúÌnYóO5fõÔRVsèÑ£Geee.+***++ëúõë:_ÞëËËÑöíÛ¥©+W®+È4K½¬:ZÕ®^µÙl>se0·îÝ»¯§£ÉÍÍÕ©f÷w§«õuéw¿û ¬Ï=ã^²«W¯fgg[­V§ÓYQQ!ÃV	NYÍ[[å]¡ñ'OÈÅªª*Ê`555Sëëë9ÚrY[[[åoýÆæ²Îß©å(+°¬èéhbccuÓ©ùh7oÞ¬­­Q,'kÃR+«ü¹766677»Ýny#YSS£cé'NÈô±cÇ(+ÒÓÑ8¨¨¨ÜÜÜÛ·oßzôèTÖétfee%%%IwÛÚÚXbX"eÕjOO±ÙuttTæHh³ÎÎNI¦Íf¬æååùï $ïã333Â2¨k²ÍK¤¬G^¸pA¦W­Z%G9Æ¾ÁæôSVÁ?^ª©§£êÐùÑÑÑ>MOO¯®®fbÑulllçÎÏêjaùðYQ'­ªªJLLÔ¤¯A6¶µµÉu|f°$±èËªëåë+Wô¢_Ígåü¬hqqqll¬Sßï½_þòyyy¥¥¥þkÍ7±Ûí>cÖíÛ·3fÅ¢/kÿ5kÌ'a8fåü¬êêêÊÍÍµZ­ºÒÙ³g£££e¢²²2''GÚd§¤ÛY99ÂÕ'Y¢©©)--M7¦÷ööz½^Ic½qº©>´*V¹ó¾ÁÆ¡¥VVÆ¬¦"^¦¤¤È+äÐ¼ÒÕ«WSSS®/eqm;A­Ü¡Ü½Á²ËËÀÀMe©;(ö êêê233fÊ5²³öôôLLLPV`òz½?ÿùÏW¯^½bÒOúSy«ð26µÙlÆÛ·o[­V£X¾e­©©1ÎÉê3fÍÏÏ¯­­­XV$o¿ý¶uÕªUEEEv»½ªªjªëçææ&%%é Õãñ$&&³±Ë:>>.á<uêÔÜL)+°xéJaaa_~ù¥±ËîÅGÅÅÅEEE¹fIbùY9ßOî $SwêJ|ðÁ|®#e5¯ò(«¯ööö2&ÕG§õÐü2MY¥¤«««¢¢BÆÆH2*-))q:ááá2ZÕùUUUÙÙÙæz½^»ÝÎyi@Y§Yì³XRzäÈÆ¬ÀRUZZ*uÌËË+,,¾÷Þ¿þõ¯m6[TTTVVùC2÷îÝkwîø´ ¬ÓïÍDY¥ª®®Îáph>;;;333W¬Xñ½ï¯¨¨(àñª««%ºÔÊÊJ·Ûír¹³ ¬Xv²³³+**ôh2fýøã×®]ä&2f-..ÎÍÍ-//çè ¬!°ûöùull²Å£IA®022£Gñ¨AÉjµ²ô@Yçþó¬FMeÚf³éL:MYN?<êp8¢££eÂÿ°GÆJ¯½öÚøCóä?=))eÊ:¿eµÁòï±qãÆÊÌ·ÎÎN=@+**äÐØÚÕÕUPP`ì tíÚ5©oii©^Y+·=þ<uáU»§OcÇíÚµ²ó-33SJi/5mkkKVÍ;(ISRR¤¯Iuuu,CPÖ]Ö5kÖèÇxÆÆÆbbb¾ýö[«==2¿K`NÄÇÇûO¦¬¬Lþõo?~*3Ù	Xeð>íçÝwßMKKãw	ÌÚ£GJJJÜnwFFËåºtéÒï'wPª­­Õ#(Ùíöúúú©ÎàUUþ3gtWaaaÆtdd$k9×××'5ÕÝëêêbccm6Û?ÿó?ë)Þ¶mÛ&s7À<µ¹¹YÞóê>ÀÆþÀÆô©S§ä¢y:­·ÞzK?«#_e²s.+++77W§oÞ¼¹gÏ×^MþUßyçÝ»w;ÂÂBðjÊúìÙ³9ÿDÍ~(=	ù:Õ)+ð"ôøMMMÛ·oê®]»RRRÊ&µµµ±WVÖùÐÚÚºvíÚ°°0ùç¿qãeæÜo¼ñ£ýHW/éJ/^LOOgÉK³¬)'RÐÝºaÃóJ¬hYå?vÇXPGxxxFFÆ¿ýÛ¿Ùl¶òòrýäL~~~ll,çLnY<hóôéSÊ¼*]]]yyyV«Õn·Ë¨ôöíÛ:¿­­-55Uú*ó¥µ½½½,+`AÕãñì¤9sFÊºyófã,èUUU2G²/Ùõë×åßP©.K§Áµ`¡õÙ³gæÓ×ÔÔ¬Y³Æív³6íííï¼óÎo¾¹nÝº/¿ü2àuFFFêêêÒÒÒôt4íXdeõ?__LQV`=zTþ³V­Zõçþçßÿþ÷eúÝwß5_ÁëõÊØÔétêÆTÇÃBwY-túòåË2çÎÊÌ×^M¬ÆÅÏ>ûLþËjkkeº···¸¸Øn·Ûl¶üü|cc*ÅZÖgÏÉø5kôLF""îß¿OY$ÿV+V¬4Ï|ã7¤µ999V«566¶´´Cä¾¬?>öìêÕ«ÛÛÛuÎ$¨ÝÝÝ2=44TPP ###å5¡ç455EEEIn«««À"+ëðððªU«ôÏJGO<i¾Úøø8cVàý×ýüsýæ7¿©¬¬LJJ9[·neáKjÌ*¤¦§¹¹YþÉå]³ae¢¯¯O*éécÇQVàÅ­_¿^F¨ôG$CÕÍ7ËÙõÃBY¥.]Ò²ê&U«Î²vwwËÄþýû)+0k]]]6MR*qÿ)ù-Y°ÔÊªQe¨j.«ªªªÒeþ¡C(+0'+++<<Üétê¡óY&ÀÒ³ëõS7NÚµkL¯^½OÝ³022rþüù=ÚLs´`ÙsÝsBF¥265öðOÿôOn·;..nûöíúÑUË«¬ÝÝÝÍÍÍÆö×ÆÆFÊèÞ½EEE¶I999]]]UUUv»]Bûöíºº:Émqq1X^e5I&ÊRV ÎÎNÝêp8$®z´¼Z­Ö«W¯W¾Ê).KX.eõx<RÓÖÖV½xÿþÝ¹²SijjÒ©ºycªä611Ñçú2xe¹Ë¢¬úÑUçãó6KíÁårÙAIÆ¦ñññ>3ÓÓÓëëëYÀÒ/«7xß¾s+ï²)+¶ë×¯çååMÔ¢¢"Ãüt4±±±æ½ÚÚÚl6§(~Y<y¢0Nnöüøñã[¶lÑ¯æ3¹RV,_|ñEtt´U¡iii2ÐxX|æääèJ¡l.½zõªÜÔÔ¤R]]Í~Y¥wïÞÕó³êÅM6é·ä¢~ÔUÞ¤K)+ÞhUg>kä3;;Û|é¢¼×ôÙA)D7oÞÌÏÏ`ËÝr¾U`¹Õ?´ü±N×ÔÔÈÅÑÑQÖc©¡ªÄÒ<çÞ½RP£©ºËåòÙA	eÉÌ¶é¸¸8ö`ÂWQQá3ÓjµêÑä_ 55UúÊ(ëì¬;vì0_LLL¤¬XÂ$«Û·o7.üìg??þôôôë×¯³Ê:---òjÒßßo.«Ûí¦¬x9úúú¥s/mO¯×ër¹òóóÿó?ÿSÆ¯¯¿þú+äý¥yË+Ê:çÎêNLwïÞUUUów&Êÿ¬:NiêÅ%«qqq¹¹¹/ç¡å¿ÿýïË_XXØªU«¾úê+~eÑ3¯Y³¦¡¡ÁÿÔ7÷îåèx9²²²Ì)Ð?~^´¾¾>))IþÔããã=ÚÝÝÍ/ ¬ëKÃáðù4Ë_|?åõzËËË¥¦z%³²Ó/@Y)+èèh³WTTäååÍí£ôõõI°õJn·»­­%`~Ë:66&ïâwïÞMYñ¥¦¦eìÿyYëêêÊÍÍµZ­6MÂ÷îÝcxeÕ½º»»Í3>JY1d)½yó¦[$gûöíiii>£ØÙijj»ûë ²ÎæÌqÆáëëë%«ò^&ÌG6´²bUVV:&Ùívßoû[ùÑjqqñfUj]]]-CaýX¶LÏI§PÖiÓz,Ãû÷ïÏÇ)+Ìdø«bæåææÆÇÇÏÉDroeeeÆ8kZV3ÞèùYO:5O¦¬0È8ÕçG2²4smzS¼±WûöIMeÜ oð###W®?I¦·nÝJY1·£Þ$%%%³»Ã®®®¬¬,«Õ×ÙÙÉBð*Ëê³1Õ9É8Àa||<eÅöÙ(33³ªªj¦÷£;(Íîo0/emii¶¶¶JDzzüË:å§¬!¦¤ÔØ°ª'	ýÃ0rÃÊÊJË¥gdéG±T¼ú²z<ÊeÅË$!LII?¿ÂÂÂììl«Õâ±eTjlLÍÈÈàoPYûúúÄRV¼7oÞ¬­­ª~FjZZZZ^^ÞÕÕ5ímå:999¶I2ÊMàeYÍW£¬1116l¸3If¦¥¥QV¼8IizzztttfffrrrlllèûîÊÀT§lL°Êª'gøð¡LËubbâàÁ2óØ±c/.+++55ÕØZYYi·ÛÜDö".KnÂ¡ó,²IY×¯_¯ÓzÆVÊ'õÙAI°Sí,£Òòòr=Ú©wYåµL	sHJë33àX¥¾2ßf³Ëß!L°ÊÊYä0¢££ö9Á¨ù KGR*A¬JÊJYLiii||¼örddDÚér¹¼^¯L?^7¦:ÎòòrvP@Y)+B¥;'%%uttTVVS¥¯ì ²RVÌG=Ï7ß|óÿðÆÆTÃ@Y)+fC/#WÉjAAG@Y)ërwïÞ½ÒÒÒ¼¼<ùzûöíÐoh:ßét°1e¥¬øßãæGGGçææVVVæääØíöi£¤ÎOJJ²X,ò£= ¬àõzG½1§ººÚårMUÊGÉØTF¨2NÑ*G@Y)+|¬©©©>3¥¬þÛJ$ÃQQQÙÙÙlL@Y)+¨««ËÌÌôrýúuãâÍ7322¬V«Ífk__Ëe¥¬Lv»Ý|qJDuNSShÃÃÃcccÙA	e¥¬¯F£$_eÀZXXXUUo±Xä[õõõì ²RVJª/#W¾ùæ?ùÉOdBOâõêUÊJY1û·kÃéhPVÊYêDaa!SPVÊY2NG£;(±Ó/ÊJY1^¯·ºº:11Q,Óì ²RVÌÆÀÀ@YYÃáÐ~9ÊJY1K·oß.**²Ùlì ²RV¼feeKVÙA	e¥¬ÀëõÊ499911QÂðÔoõõõéééÒTËUVVFSPVÊ)I2%«W¯^íêê*,,´ÛíÆ^neee\Åb¯­­e%²"ºº:	§ÔSZZÖÛÛ«Se*Ù7oÒT²bzeeeæ9ÝÝÝ+W®´Z­QQQyyyâ(+fYÖ¶¶6·Û-Ô×^­¤¤D¬ ¬ººº?ýÓ?ýÿø¤¤$iªÓéüà~úÓ²d²bÆFFFÊËË£¢¢,Ë;ï¼sòäÉ÷`PV¤¯¯¯¨¨H"*YÝ±cÇßüÍß$''ÇÇÇgff²a(+f@Â©G¬æåå±1(+f©©©)55U7¦p´ ¬¥úúúøøxÅÈéh²b¼^oYYYll¬4uûöíçÏ§©@Y1½½½ùùùÑÑÑÆX&°dËzãÆøøøÊ:·:;;ÝnwTTÝn/**êëëcÀ/ëúõëõù9sfÃ2qÑÏ¶mÛ(ëL555¥¥¥éJ¥¥¥æCrYÍV®í·ßZyï½÷ø]Èãñ¸Yh)))oYÛÛÛ÷îÝ+c~d>cÖÐuuueddÈEË·¬£££'O°@YgÌXÇ«÷ìÙ344Ä¾ÁÊú¢<ÏÖ­[ùÔ²ÎóJ@Y9R²RV(+²(+e²@Y)+²RVe¥¬PVÊ ¬@Y)+ÊJY²@Y ¬@Y)+²RV(+²(+e²@Y)+²RVe¥¬PVÊ ¬@Y)+ÊJY²@Y ¬@Y)+²RV(+ePVÊ ¬Êe¥¬ÊJY²@Y)+²RVe¥¬PV(+ePVÊe²RVe¥¬ÊJY ¬@Y)+²RV(+²(+ePVÊe¥¬ÊJY²@Y ¬@Y)+ÊJY²(+e²RVe¥¬ÊJY ¬PVÊ ¬ERÖææfÅBYu'$$PVe<|ø°QÖ]~NgZZ¿Ke^bbâÄÄePÖ9àv»¯]»ö¿OµÁÊ:;ï§9@Yç&·Y²(+G²ò»PVÊ ¬Êe¥¬ÊJY²@Y)+²RVe¥¬PV(+ePÖ`ÿþý¯¿þúú9åt:×­[·!/®?þã?f9è?üall,Ë!Dò'ÂâYÐX¡/.ùÛû´Z­¾¬<ø×¹öùRÖEh~ö³­^½å¢_üâå¢>úH×_~É¢ÅáÃeqýã?þ#"¿ùÍodqýò¿Û»ý÷ÿ÷Å]Öùðë_ÿzóæÍÏ£G¾ýöÛ,5449ß0|´··ËâdQb``@×7ß|Ã¢Åÿ÷Ëâjll|9GY)+e¥¬²RVÊJY)+e¥¬²RVÊJY)+(+e¥¬²RVÊJY)ëL|ùå|ðv!ª©©ùÉO~ÂrÑ+WË!D]]]²¸¼^/"CCC²¸zzzX¡ÅõÛßþ²°øPV(+u¡éèèp87nliiáO! 7n$$$ÈR%ÆrEss³±ã+gÏíÙ³'22rÝºuÅ5-YJqqqº|Z[[YbS	òj?ßËmù5##ãôéÓ2qìØ±]»vñ·Ðúõëõ¨gÎÙ°aËmZãããò^Ä(++O?ýôÐ¡C§ÓÉâÖ5kúûûeB¾j9Xbþä=¼ÿ0ïï¿æ¹Yóß¨üKËÄØØùÝ¦²råJÛ´<¨tåÏlZ2sçÿ¡÷ú©$ùªïEXbþ¶mÛvÿþsYýÒ|/·å[ÖÓ¨½½ïÞ½,·àd$(ÿ±Æ5+øÿàW_%ïØ$·nÝbqM«££CüuÉWùdk©¬þKi¾Ûò-kXX1Éb£££O<a¹çv»¯]»fþ¯fqÿ¬ªª»wïÊ;×´6mÚ¤£|I¬â%JYýÒ|/·å[Ö·ÞzkllL×È4SÜ³gÏÐÐË-f3×´ÿ>ã×´£|Ø,Êê¿æ¹-ß²~øá§N	ù*2þòx<[·nf¹Íî¿ÅD^^ÞÙ³ge¢§§'99Å5-§êAº»»eüÊ±¬þKi¾Ûò-kkkëÚµkÃÂÂÇ7øC(&&ÆgÆrÑ5+Ç»Ýn%&&Þ¿Å5­ÞÞ^	ª,1ù*Ó,±Ëê¿æ¹q¤(+Ê(+Ê(+ÊeÊ`îþ-M6eLª¯¯¯©©9_ýuëë¹eÐó·8p@/>yòD.êéTPV`IinnvOJínÝº%_õXæ^ºtÉ'éÍtáÂÆÆF¹Â;9þ¹Ìyüø±qÖÖVcDï³¡¡åPV`©yöìyXÙÙÙ)_ÛÛÛub||(Ö«õ÷÷ËÅÊ×-[¶èLó9wu[PPà3G¬7ÆÁÛ¶mé'NÈô±cÇø]XtâÎ;R¸ÁÁAùÚÓÓsùòeðz½>eu820Õ¾®ZµJF¨2!CÒ7êõôéÓ2§»»ÛgÔ+÷iT|ttÔ|(+°tèPUúzöìÙÈÈH_JuïãÇÊjAõºöX¸z¾qÝLk¾ò#Gd¦DW¦5ÆG9Zb8wî¿²KÇððð¾û¤pgÎÑ4ÊSj'2Êô/«víZLL®Ôýì³ÏdæÝ»w×®]»k×.0ßùØØØÎ;u²ÞÖívûDZîß@Y%åÒ¥KÆ.EºõØ±c2±wï^-ëåËõOù®®7ÖM¤ÆFV]üàÁpÊX¾^¹rÅìÊPÕØÝI¼üÊ,)ÚÈ_oo¯&Pb)ÃVèïï7Æ zÝ.ët:/LÒtbÍ5Ë	¹Lëõ±ÿU¿²Knj½qã|=~ü¸¼®[·Î¸èõzhVëëëýïjhhHw÷Ï'cV²ËàÿÖbÉÏÏ×8öìªU«$±±±Æu¨ò]¹øá4grýúõ1+@YeAWüÈtKKËt<*ó¥¬Ú<Zuo&ÞºuK¾ÊÕt¯XÄ1+@Y¥O÷N2ïstòäIÓ××÷ü»OÔ¤¤¤hÍkw?~¬3¥¦FY¥¸2Gò<Ó1kOOO*À¢aðÙ³gRÄ7z<ioÕÐÐ Ã¢Ýºuiiiú-¯×+ßZ½zµYkjjôr1k~~¾nß@YegÃ§O6.ê¡>ýôScÎèè¨Ï°UW&:u)@Y ¬PV@Y ¬PV(+ ¬PV(+ ¬PV(+ ¬PV(+ÌÊÿ¦¸íÔA¾ßôIEND®B`


反趋势正态 Q-Q 图


]ÇöA:³½Ú$«®Õ/ÝlÚ$+HV°¿<· ì Y¥¢Ò`Ð¢dìØ±zWò@ê×åËïåÂÕºãMÚÑÑÑZJJId]FÇúS§OÖøvIID©Ü]±b.çñlÉ*UnÊÖGãââ<|ÌÎ$«ñòºUÚiii±©óäöæÍr«k¶°×Ü·í4iÜ¸qCwKKãóÏ?÷¸a 99ÙÖ//-ýúN:ø ò~´Ý^ldÕÖ÷_SS£+I·nÝ"YA²b zqþüù¶N[²±ÝÒÀÜó§ß­UUUú¥/l?k¾jMÈGµ-³iÓ&k¤©&eHH®ÄnR¶¤úåëÞlÖç_·nÜê¾¼[	ÛFZ÷Ð¬d~vÝÇ¬ùÝÞD'Tw	äÓ03gî­¼wïí§Î=+k2nú#¯:KcU·»ºö¶Ëz<*¿h¸<´xñbó$K,é8YåW ##¿3fHgYYu$+@-©8u§º1ÖìdµîÄ5É.íÆÆFS£lß¾]¿è%_9âqZ-YýýýåHYfa÷dÕ¼/´äðÌ:UÇ,,ùª³³³µ¬Ô¸õøöæÍ§m,)O"Á&m%¬ä½¾añºªá¾]WÈ°èæw]@çR½óÎ;º5Uªð±cÇ®ZµÊ¬XÉ Ëhë[K|wò¨·¢ÙKjfiëJûNq]Ñ	ÒºðÔ©SM[Þ§²j[*WÝ¾mÃE²dÅ@¤eÄÇÕF*pTÒÆº'Oª'N=åGmÛªMç¬æ  ­«$õõî¡CÜ·mê&kS^ËÝòòrÉ¹¸¸8É-uQÉúÄ­ÁK²0|øpMAÝ©¥°¬UèvWÝúmÞ¡|^Sßkm¶¨[«X	T___YùÐ£Y¿<y²µÌ=qâDdd¤¦ÜêDnùM¨Êlr0¤Ïn;mÛt?wîÉªBºR¥k2ÎÙ$+HVD<&·ÜÕZ<éÂò¥¬ÓGu¿ |Fµ±V½îGx¬YÍ4(i§¦¦>þq¡õ H¬dª.éÅlÛ¶mf× ùÐÐP­Õtê¬fnCÖi®îÑÒùd8±n@Ó-J5Òyééé:8:XîJÃìX¶Uºú].©¡%JeFÊÇm;_ÍfpÛÈÛ~¡§yüãmMn©ò;¬úq¤²|R­wåýkBkñmÝV¬èÏä+X¾ÇMð2[mhÕ¥É*"ßÔºT·þÑÒ¶Ù°duF¤3b$,­?bÂàÎ;¶ãRÌ»ÒùMíÕ¬&ðô=<ø¤«5«©üt´f©à¥X´þºmÀ.,y©¾ÜêïÂLÿQf¥G"S÷hê¾Lyr³-×ã®Vó¢¿üå/­wmËèFÕlûu¬¶Ý¡Á¬÷©YA²b`ñøuÜñÖ`M)Ýl+_ÇºSJ±òòrs¬Ë§~ÚÑßúU9ÖdÕ¨z¤Jii©¼zÉê±füãÎ`§LV­ðteB7_UYÐ~óºÅ[?9599Y·ëêG¨¬¬Ô¹Áfµd°ÇÍ3ÇÖãñ<z4u®²íèd³î~vOÓÎl[¶lÑgvßx@²dÅ@×dÕ¯Î»wïêfØûmÚ³ã]Öý¬îtõ$|¶ù¥¬:Ujøðáò/^,·ºÉQ"³K5«aëE²ZÑºß@²nÕM¾:wW6iM²êÝ'NH|FDD¤¦¦j½®Ç¶j-ØÞ¼_3=.©Gw>ê¼KVMh=3íl$+HVtºÓ®½d5'âïP-¦:³Ôzr¥'~qsÊ¸~4ÛH²êþWYFøÈÑË/w>Y5ÎuãçØ±cÍò%%%o¾ù¦>*¯®Å®N]6ûwµ,3ïMç÷ê)|ÝÏ«ªZÖLÍþøÇS:èéM²êq·ía5·,µéÑ©§²í$«8òKÑms¾<êYÏb0&YA²õ4@í%«UUUæî;w¬yöØÓ©'Ü¿¸ÍüaÛMJ÷:Ûtó¸mc©î:Øö#¬T:A7$$DCË¿«û;|Çn'Tê*=lFIÉ+ï¶ÍÖ)B¢¼¼qëÈÚFõX¡½ß£õ¦Rè»_GÁc»§¬ä¥ûlgs´^ºÀzÇV$+CÙRêùÿX±q§³gmW0³tg§­aaa¶sI¦º3úl>)éÂÃÃW­ZÕ¥×OÔÁ[Y+r8¶BùñSxVçöÞªÇG;^züãÜ.þ­@²@²¬¬¬dddd$+$+$+ÏîÝ»§×ßþß'O?ýÔãÂ>G«««¯]»ÖÁÞ;éÑ£G/w*Ï|úôiëÇâÕÿø$+ÐÊ>>ü±µG¾ç¶IÿÑÑ£G9"KnÚ´©½çe._¾lÓº¤í:çr7%%E¥¥¥òSû÷ï×+rK[©©©Ö§u¶9ÚF]´h¾i8qB¹÷®Ü­­­7o.`b²¸¸Xµ¾ºí%´³²²RnºúêôÇ Yî'_²&#å;WbÒú=n-Tss³6§OÞÉrPJ_³¼¼|b½[TT¤Ý¹sGîJáxêÔ©[·n¹ÇU]]<ºwïÞÞªºqã.,·´<v^^Þýû÷ån¸¼yófB3&Û·o7)CuõÕÿø$+ÐÍLR¶¶¶Ê÷ohh¨|q/^¼Xnåî_~)·RÛ¹ÿ`çÕVVTTHlÌ1CïjK`è·¿$½vêlp8Ò¶>ÉÃ-[&®X±Â¼U©Ø´a¶ÊsO§ÛEåö»ï¾ÛãÇkRZÕCKKõ@íÒ«½à@²=¢¶¶V·sJÅ&_»555×¬]JVùos­ü¤ÝArh[coÿþým¾¾¾ºyÓZái7gÎ÷äÈÏÏ·~EéæÜÛ·okE¨CµLÔdZVÚÖÃ¡K¯þDÏ|è?Ùë¹Ï?ÿÈÜ¼yÓ*òU>½Ùtìt:%a¶ºÇÖÄ7nÜè89l!a^Ý÷ïß7HIÝñJDfccãéÓ§MØtÉ=YõêÆaI¦mÌºví¯îãIodzÊÊ+­ãþul¶¬v¾fU¶ä¨ªªrOIn­±$ó¤¬×»K,1eÎÖÑý&-ÂÃÃ%ÿtáääd3ª¬¬Ì,&Ï|èÐ!éÔÙL/_ö¬Âìv5)+my	÷àìÌ«÷þñHV GHµªëã¶9MÒÖÝ®O¹5ØRk¶I¸'"2bÄYæqÛÎN)1MBXk²¦¦¦MÄÄDiH'íuëÖéOÿfß°ÎÖÒS7w?1Y¥Ì5mÉ*kuþÕ;Y³>÷ñHV ûÉW¹|ÕæææÊ­µ0íÞdMII)))_ímí´ÒêJk5y:ë^=§§§kHÑf	!ýP?:7Xw*»'k\Ôî&YuCÖ<$Ûäg;ÿê]­Yýø$+Ð#tÂTuZíÜ¹³ÛU>SÆé1$S§Nµ%mÒÈÊÊÒFvïÞ­Ë|ùìÙ³:§×ZI¼IAýëZêVn]çÑ¦:û×vÔÖysçÎ4Ý²eÎ­ÕÄË/·&Vg^½ãø$+Ð¯o[ÓÒïk÷©¬¬Ôïq÷ó*x$_ñ:qâÄéÓ§µ§¦¦F7f,Ñ¡ï¼ó	ûêêêªª*-.õ¨³8pày·òÌÜr+t£ª³téRéüýý¥G²VJî÷©tÓ±dä«yH^W¬K¯ÞIÏq|èfí½Jq&|Ë¸Çt_]^^^'_H2Év#åî³iLI¶òÉ'æÌ|Z2Jéæt:Í9DLLÖà74%´***"##MhéóK¿.&UigÞ¿ntÍÈÈèÒ«wÞódd$+$+$+ Y YÐ-[BBBBx:ãÇÿÛßþF²>þÏÿüÏY³fÕðt^yåï¾ûdýg²þú×¿þOç¿ü%ÉJ²HV@²¬dd%Y$+É YIVHV@²¬d¬$+$+É YIVÉJ²0@µ¢¢Âår9Î¨¨¨ââbëC=Z¼x±¯¯oxxxII	É Y,%%¥  @¹¹¹©©©ÖÖ­[·iÓ¦ÖÖVÕéùÎÍ[o½E²HÖÿ,Ù)æææÐÐPëCRÅVWW»?üð'¯¿þ:õ_N§Ç¶ÞÝ¶m¿¿¿¬UUUÒÓèæ·¿ý-5+dý_Ã´måååI£¦¦&&&ý¬õÉÆÛÜÜ¬[¥m¨½rd¬-0??_rb(33óÀÒ¸|ùr\É Y¬´´4$$Äáp¸²²²~÷î%''KµsíÚ5@²r¦ÉJ²HVd¬$+d%Y YIVÉJ²HVd¬$+d%Y$+ÉÉJ²HV@²¬¬$+d%Y§«W¯æäädffÊí+W YIVïUVV¥§§ïÚµKÂuÜ¸q'OdXd%Y½½uëVs÷ðáÃ®õõõd%Y»ìêÕ«&Mjjj²v&$$;vÁ@²¬$k?>..ÎÖ¾oß>ÉJ²¬]ÖÐÐ0|øp©ã¸qã.Àà YIVÕ«W¯¾xñ¢´kkkÒÒÒ ïª««;wî³%HVõyÊÊÊZ¶l­	ÐÉÊq||¼Ëå[ù§ÎÈÈhhh`XHVõyþOÚ¦2èCäÿ7&&FVõYjÖ¤¤$	WFd%YÀRªZ$r­««cpHVºlóæÍ«W¯¶uJÖ08$+É]¶oß¾äädkOSSËå²NþÉJ²@gÕ××íÙ³ÇÄjffæ9sd/]¸p!"""666==]«µµµÉJ²÷¤T-))Ù·oß¹sçíO²¬d¬$+$+É YIVèçÎã@Udn9|øð¸¸¸°°°ØØØ+W®0&$+É^ÊÉÉÖjµ©©iõêÕ&Mâèd/7N¯¦lÄÇÇïÛ·!YIVè²úúú°°0[§­ë×¯gpHV¼d»*^^#C²¬àôôô´´4³cõØ±cL&YIVðRCCCLLLtttvv¶D¬ÄêáÃdïIÁ*iº~ýú¼¼<ªUd¬$+d%Yù¬$+d%Y$+ÉÉJ²HV@²¬¬$+d%Y$+É YIVHV@²¬µ×©¨¨p¹§3**ª¸¸ØS§Nùøø¬ú	&È·_|||uu5B²v§iäææ¦¦¦Úmii:uªIÖ5nbbbøÐH |ðÁ6l|2dÈ÷ßÏ°¬Ý&88¸µµUÍÍÍ¡¡¡¶G7nÜ¸eË¬1näÇIV«ZX½~ýºé6mÚøñãµÛ8NmqóæMÉNÉ]¶è7¤HjÕÚSUU5hÐ Fdí6Ã´­%''=ödÐ_HyºaÃkOcc#ÉJ²v§±cÇ677ëÖ`iÿó¬úÙ³gKÙjíyï½÷FÁÈ¬ÝfáÂùùùÒÛËP³è7®_¿>tèÐ¨¨¨ªª*©V%V¥`Ý±c#C²vÒÒÒÃár¹ÊÊÊ<F)É ?Lï=ÉT©VU3EÎ ÁË×4?Î YIVxéÜ¹s©³gÏ¾Þ&>>ÞétÊª7#d%Yá'Î9ÓÚaëdEgØ6ÿnØ°!$$@²¬ðÆ#þô§?Y>øàËÅÈ YIVx#>>>((¨±±QïJcØ°aï¾û.#d%Yáúúú__ß·ÛøùùEDD d7²²²¦µ±d%Y YIVÉJ²HV@²¬$+d%Y §ýùÏ<x°¹Ä5G¬$+xïÛo¿4u8_õÕÿüÏÿ5Jîfee12$+ÉÞðóó4hµÇ×××ÖdÎ>ºµGV)[dð<UWWO6-  `Ø°aS¦LéC$d·ö,X°d%YIVÏÓõë×%PCBB¢¢¢ræÌ>ñæ].äè·ß~kÍZ___~­$+Éà¹=¶õúNBÂuÂ	åý;	×#Fëôà>Ts¬$+~hüøñ~ø¡µG*W??¿>ôfÎ)uªÚ®Ä*ÉJ²xÎ¤<µ¦RPP0lØ0Fd%YÀ×¯_7=.+66!YIVðRHH«T®k×®3f¬Ö ÉJ²@×466.X°ÀÕæõ×_'VIVd¬$+d%Y YIVÉJ²èå;gÚ´i±±±k×®e@HVÊ1c|ß~ûí8Î¨¨(ëYA²¬Ð¦#F¨¯¯×»ÕÕÕ²K,adHV¼1zôèÍ7[$VûÐ¥o@²¬z??¿ãÇ[$hÇÃÈ¬$+xCÊÓÄÄDkNebdHÖ8pÀÇÇ§¥¥d¢¢¢A-Y²¤±ÍÛo¿=xðàsçÎ12$kGBCCÃÃÃ=>ÔÚÚJ²à6lØàt:%P%b$kõ	V­Zåïï¿råÊÂÂÂüü|©e³³³IV¨®®ÖÞTVV¦¥¥EFFÆÇÇ¯_¿¾©©Á!YÝ~ØÇgúôéìg'*))	ÊÉÉ¹xñ¢´çÌ3þ|Âdõ¬³fÍ"Yà¢££óòòÌÝÆÆÆ=ö02$ëÿzøð¡OÂÂBÛÖà-[¶HMMÉ¢¡¡ÁårÙ*Ô/¾øbÅÉú577»÷9rDµ®®dMÖqãÆÙuëÖ­$+Éúd:%XÆ¿!Tttô;ÌÝÆÆFéak0Éú/§NJncÛüÉ'h©*·ñññìgãüùó.+33óØ±c¨«Ì`"YÿåÑ£GOÙD²¢Û]¼xñÂYYYIIIiiiyyyÄ*ÉÚµ9ÃË-#YÑ+ûaaa&M>|øÖ­[ý?YoÝº5qâÄC­ZµJµªªdE·¸zõjPP¤©®àWVVFDDØ.!ý³fmiiRUbõìÙ³Ïîní9yò¤Ô¯þ¬)=!33s×®]¶N???F@KÖòòòymÌÜ`¥í½÷Jñ*mO_³.[¶ÌÚsþüyjVý0Y[ZZl´(ýüóÏ©YÑ½t?«¬®éÝºººØØX[F@KVSIVôcÇÎ3'--M®d%YñTÐjhh|=xðàÅIý6Y[[[%M/_î¬Ï'L²dW®ÊÎÎæZHÖÎÖ©ÒÔLÚ&YaH®]»ÖÜåZHÖN%+[ÑqãÆÕ××[¸ÐdÅS%kCCµS YIVx­ÁHÖ^¬.ËétFEE[*++:uª<-¬½3¬O¬=tåóiäææ¦¦¦Z8q¢~Èýû÷O<dím*++å×'õ+×@²Ú/~®scbbÌ|`ÓÎÏÏ»R>v²ë¹CCCÛ[Ìßß_náFÊ¦þ ½+YxñócMëö»¼¼|éÒ¥ÒøØd?ÉèuÉú9Óöõõu_àþýûR7?xð­ÁõÉÆ«'x[iÛ½ûöâÅïÜ¹ÃÜ`@NVyóæ=d]¸pa~~¾4äÖvº¹sçÞ½£n>Y7nÜhíyøða%kiiiHHÃáp¹æÈmhh¨ÉÚ566<x	ÆúC²Jiøf¼ÿ~±3fK çååI¼$g@OØ°aÃàÁýüüôÁ0&úv²>zôÈzÍó½÷'''÷Ý´$k_'uª5MwìØ!w?üðCF@NV÷É×ÕÕI£©C$+ºÅ´iÓâãã­=AAA~¬Ö='Nvuu5ÉÎu².ã0$$dóæÍÖ3gÎ:ÐçõÑ£G£ÁÁÁzö¥¢¢"®N§óÚµk$+:#µ¦ËåÛÛ5æÚhíÉÊÊ¢fÐ·õÞ½=ztyy¹ö=zTõÒ¥KÒ¾sç|ÓÉ]___YdG«W¯®¬¬¶djzzz\~°  `ðàÁ;vì0«¬É1	@NÖ»wï1BÏn(_·nÝÝ½·u±jV´§¡¡!00ðêÕ«ÖÎÃwæÇ,Y2hÐ ñãÇKÃVÂ@ß«Y¤iIIÉ©S§$S¥F]]ªD¯´sssIV´çüùóîªT±999|ªªªÛ;wñÐU²óøñã¬ºKUÂUû%Y/]º$+W¬ðHªÕÛÄ¥³V²ênT)U­Éªòòòtn°ôoÚ´dEbbbÖ¯_oî9sÆû0 ÕlÖ£n¶IMMöèÑ£9ê)[õèÙÙÙiii«¬ÉÚ455<xP*×¼¼<ªU$+É Y Y/]ºtêÔ)3­éÈ#$+dí³JÃ¤,É Y½¹¤iii©Þ½víÎ&Y$ké!lÖôÚÃXIV@¯NV=ÿòåË=ÆmJJÉ Y;ëÁz¦7Û¤ü¨°°pçÎ³fÍÒ¯Ö¤¬µÝYK555zÙs½;út¨¨¨Hîê$d0É Y»´ü±¶÷îÝ+wïß¿ÏÖ`Éê7nX¶vdd$3$«÷ë¼yó¬wcbbHVÉêââbÒ7oZ599d¬]vèÐ!ÉQÄTSS#wóòòzÿiHV@¯KÖ[·n¹_QnéÒ¥Ý@²r­zS²677KÁºhÑ"@²vÝÃzéÒ%kçÃIVÉÚÙ«ÜSJ¬JÃz¦CëæHVÉÚÓ>räwéÚµk½ùìK$+ ÷&«íìüz-¹üüü^~jCÐÛuùòå¦Ë-+,,ôõõõ÷÷/l#í¹sç¬µl;S#Ú1EGG¬µ§6---½|ù²²öÎ¬Þ¬%%%ZJd¬O¬wïÞ­««3K²HÖnÁdMÖÐÐÐÉ'W·Î@²zs!¹[·nIÛårI²¶¶¶nÜ¸Q:sssIVÉê=IÖ'j[¯.G²HVïIÎ9$+W $«©ænUUô¬µËnß¾-9Z^^nÚ^xqÐÛµ¹¹9;;;000==]Op¸ÿ~½øyhh¨¹µ%Y$k»Z[[%88`JUyj¹]´hµ~çwHV<7oÞ|é¥0öl@ßKVN©SMûèÑ£æ¨VÓùùç¬èi_ýõ 6?ýéOCBBäÏ××W²Ð·5<<üîÝ»ÒxôèéÜ¾;Éæt:R	ZùÛcdô½d8qbzzºV«r«R4¶mÛF²¢§É_ÚîÝ»­=/½ôd-# o×¬¢µµµÒ.++ÓÎ7¬xÉjÛöûÆot¬'OlYRRRfffee% W'«hii1[¶l!YÑÓt«µgÈ!#Gô¸ð=rrr$_W¯^-mi0zu²Zøì³ÏHVô´÷ß_þØ$rýúë¯&w9â¾dCCDiIIé),,t¹#^¬Öý¬òEæ~XsñÌÂU*W=QÃáØ·oõQ¹3|øð^xAþhm9áÂÆÀsNV÷ãYwîÜ©CBB/_n;dÅsñÅ_é&ßÜÜÜ1cÆdffZ;þ<à9'«$¨guuµÞøð¡õÑÝ»w[·¬x^Í4¥«W¯Jå:räÈ/j<$=ç¬§¤Ö¬¬,ÉÚ´MØ¼y3d%YÑ)RFFFÚ:þó»)Uå¡]»v1JzW²úøø|ôÑGÖMÄ½óHuÀËËË3wÏ?/5kmm-# 7&«ÎpÓ¦M¦çÒ¥K¶ëÊ¬x¾$JÇ.åiVVVPPÐáÃ½4YõÀi8¹sçê8³fÍZ¼x±SZZJ²â¹«¯¯ß¼yó%KÖ®]Ë4`½7YõÀ½÷jÄ¾ùæî¹ûÄóWTT¸§ÓU\ÜñCÞõ¬¾¬0Uõ©SÓd=MRRR¤ÚñCÞõün~ûÛß¾öÚkWx:±±±Ýy¦É-½à¹&kvv¶mÎ.88XJÍÍÍ¡¡¡?äEÏ?üà@Ïì¶d§«©©[-õrG1e«4òóó¥ÿÞ½<ÓéôØöøw=çÝ¼õÖ[¯¼òÊIÎÔ©S»'Yoß¾fÝªÑ]RR"·:)..îÔ©Sr÷Úµk<ä±iûúúvüw=ìgôý¬LÖ7ntðcÇmnnÖ·Òîø!ïzHV@_JÖòòò+WúûûòÉ'Ò²jÕª#F|ôÑGOÜ¼páÂüü|iÈmJJJÇy×C²úX²Êmee¥Ü?~¼«5kii©$±Ãáp¹æi=>ä]ÉíÞ½Ê)òg3öì7o2 zE²Ê÷Üv5Y9S¯_üâòWêt:¥1hÐ ¯¿þa¼ÐÐÐ°oß¾¹­¯¯'Y½OÖÈÈÈÆÆFmK²=:77·®®.???++K:=zD²¢wZ³f®XÃÁÈ]UYY´zõêËuåÊõ©f0iÛzkss³9&dEï4fÌk¬ºÁ:¯©©iÒ¤I_|ñéY¿~ll¬ô¬^úòË/m§à7çæüèÍüýýåÕÖ)½ûöícpÎ;wîíBÈ©aaaW¯^%Y½ôðáCù2Z´hÞðàÜÝ½·>D²¢×zñÅN§µ'''èª¦¥¥Ù:ãããKJJHÖ®iiiÞæèÑ£.kéÒ¥GÛÈÓÂ¥QPP myU½nû|Õ(RUî5ºäÂ¦§®®.00PnIVoÎlZùVúòË/÷z$+Ä¤³å6  µ«ÅÊ1cdô<~üøï¿ÿ^:«ªª%))iþüùºùWnåî²eËÂï+Ïj#êÆå)##CO¬ëºuëHVô~Û¶m[¼xqyy9CÑ%EEE©³gÏ¾~ýzuuõ´iÓ4b44h1455­X±B~éaaar9¦/ý£ö³ê©uÖRbb¢íQYè¯&L0sæLsW´ÊõmW"õ+5 ÔÖÖLíd=öljs5V)^­×gÝ»w¯^dú%??¿ãÇ»Ão1cÆË%+ÜHÖ.W«Z°N>ýqA²Oc×®]iiiÉÉÉþþþ6m2ýúÿø)dMOFFÆ)S1¬]vãÆIÖöNÒK²ýjllìÁ;öÂ/8k×®éCCu:K,1Ï9söìÙHÖ®Ñ£WO8a®ynÁtàÀèOÕªÄªõà???	×·ß~ÁR³Jûúõëú|	HÜ2n Y»«ùùù­m¤§3W;'Y>*--MªUkÏ·ß~;vìØ_~yÚ´iôÑ1c¤lRuÊ)«Öú Y¬±±QBÔö¶dmii!Y~#%%åðáÃÖ/FGG[Ö®]øî»ïZ'7$ë=zôH´¹¹YqÞ¼yfðºuë¤_VTÍa½Ø*Éô999IIIÖ¬¬,S¬Ý¼UªRëÁôc±±±R¹?^ªÕìììÈÈÈpÖuà&k_D²^kllzu\Ú¹'@²¬@¯ðõ×_¿øâ~~~£FÊÊÊb@@²¬¼÷þûïûøø2$,,läÈzÙf$+É¨¾¾Þ	¯éúlA»fÍF$+É¤¨¨(00P¯Ö"ÅÖ~Ø]Ïü§?ýiöìÙ/¿üò»ï¾;bÛã5ä­Y¬$+ú¹3gÎ4(11Q¯-ºaÃ¹+-OÿÌ,´dÍÈÈòóó«®®îß)µ©­säÈ!!!ü¥d%Y1PL6-**ÊÚ#5«YO_­J¬Jl)S¦ôû=²ê`Ûö«=o¼ñi YIVRNIjë0ðîÙêêêöíÛóÒK/½öÚkÖ¾ÿþÃñ>QUUÕÚµkeýà¹<,,ÌD©UØ¶ YIVôgR°¾÷ÞÖãÇ;N/J*ÔqãÆ¥¥¥­^½zøðá#G´÷é¾¥´Û½ýöÛò*®6ÒX°`Á³Ò_üâ>?6lXyy9f YIV 7o2d9ÛmPPÐ´iÓ¼¨V%Ìõ®¤µklllSSöHÐ£E^ÂúY$éeá>à·¬$+)½ÚË/¿<sæÌ¡CH¾võIöíÛbîÊ3èKåßOîîØ±C2oíÚµ=úAÆcËÑ¬¬,YQàW¬$+5©ó¤ÊÝ°aNö¢ö]½zµµçûï¿pÌLu8=ý)üüü¬s¦´l÷ÐÁ;wNÖ	äVjëÛ·o¯Y³&))éÓO?åO YIV<gñññÖ)[¯]»VUUÕ£/ñâE©·nÝ:jÔ(ÛñB¯¾úªuç7ß|cjhhHOO~ñÅu¶Ñ#¤!Õ¶ua$+ÉgMj>É§¬¬,Ý±*±*Åß3¨Sóòò./=zôh	Å;wêC			¨.KQIÉÝÁÿýï×GÓÒÒôÝJ§,éïï¯weyY¸ãb YIV=®¶¶VªÀ°°0)^¥ZZPêÂÅÊÊÊ   sçÎéÝÆÆÆððp	×am$,Çg]~È!:9«®®N2oOV¤H5í¹~ýºüø_þò~­ÉJ²¢HÑ¶ûö?þñÒxâÂRmrkòÕz×¦¼Mw½Ï)U­=/^p=ØF¢ÑöþgÌñüDçÏ·n»YØ³g5ÿð?ðÇ¬$+Ö[o½%4¸4.ÞRó¥¤¤HÕ()%% T~L*~ÿý÷¥4=mÑz'33sÇ¶N???mÈËYúéO*µ¬ählll@@µf:thdd$5+@²¬èfR¥I¢cW~÷»ßÉ]©_=.¦ùÔØØ¸dÉs«Õ#GäyÆ£g#Òs)HÖz÷&Íþåy­<yRRÛÁÁÁþþþ¦lÝ¿¿ÎcPö	tUàoû>¤ó¢ÙÏ¬$+ºDÑ¯~õ+kOXXnAµ9wî5G¥-=¦ì³9r¤í!!!ÞìPþ¥9sfFF<´ÿë¿þKßFee¥¼¼¼<]òïÿ»£Í3&O,Ùi_õôHåª·²¶¹Á¬ÉJ²¢<xß¾Ößÿþ÷OvxðàÁôôt[§mW¥!e;+ý¶mÛ¼8;±Ä¹¼C©YMT®Ò3iÒ$) ¾yófëòRJy*Å«d§ä¥íJvS¦LùÍo~#«µµµº°ìIIIkÖ¬éÌæÎÓÃd»ýidE0tèPë©þÑv1ÀÀ@!mÛök^kZ1kÏï½çÅi,Yb»`N½<Ï_ÿú×óçÏw0¹ººÚ½Dj511±GÇ³¨¨H^W²_Kaip,HVË¯~õ+ëÄ©_¥²´e­c`ë×¯dõx§3fÈó9rÄä¼ÊÈ#>ÜÞDbw,9s¦­sÈ!9$µ¢÷éëëÛ£'U>æ/¼`JU©åmP¹d%Y1°DEEéi4$Û[²¶¶666622ROc$l·±g		Ñë¬Iõ&É<þ|©çÎ+#ýE£F2çsPR.[¶L|õÕWN§uò;:¹¿VþdÉ>ø@2Uî	&=Ú»S9vÒ5käcZsT§Ûf,$+Éþïo¾Iiã1. µì3g4ôÔ»%%%g[½ñÆÃmsñâEíýõ×%o^yå	¿üå/#F4ûDóòòWNNÎÉ'¥,¬$ÖÚèÅm,YÒÉÏ%5«¼©$+^ KÞzë-ù,î6Éd%YìÂQ/Âª§[jÒçIJJ²Î¨6m5 %~´V®««>|¸ußíÁ%eù)I	×^;üãåMZkÖòòrY`W+HVøçQ©ðV¬X1gÎ3Qh×®]RMzqCëvc=gïÿ÷'$$$V¥qøðaÉr÷º¹OÓé5Ý¸-·þþþ§$+ÉDÂ¡Cj(8`TbÏãa6O¬YÍOé®Ç5kÖXkÖ¨¨(©«ªª:d;í+Éªi*PPnýüül»dÅÀ%iºaÃ´ÍW`"Ì:wõêÕë×¯ïêÓJ±kÎ¤¯[wÍ&ßòòrMqçýë_ÍÏÊOõèÌ£n÷Í7ßüáðxê	a©ÎGå>ç YIVôCÃ3g:RJZµV]¢WzNn÷»ßél&IS£T]]=zôhÞûö]¹rE^KÞw¯ØÉgÑiÒ:qZ>uO_Ë YIV<gò]o­W¬XñÂ/L4Iïê~V¯gØÊ3?þÂMMMRÕÉ3:T¯þvêÔ)³<¿dÏôéÓåu<Ø?ÆvÁòIåVïJ¦jÊòWdE&µÖJ"ð'?ùäl¶èv÷#U"""¬¥sÿàççg;rVVVçÏø(¼¬èèååO<Éß*HV^ÃÜT¥²RÅ~úé§öì9sæLOZÛ¶5((H÷õö'CÑùÏVLVüqãÆ­^½Z2õ/¾ñé7[ÈA²¬èç$áÆït:gÎ©Wéso¿ý¶¹»mÛ¶Á÷¿«¶3'GFFv2YÃÂÂ¬WVVÊPçO	¬$+3É¶HÚ=ÓòÉ«HFDDHÇÆÆÉÉýîXµo¿ýVîN>]îFGG?ñ/^¼è¾Øüùóù[ÉJ²UWWKO2eöìÙýõc®]»ÖÇÂvöHgëLKKë7s»@²¬¶xýê«¯¸ØÉ'õ´ãÇ:thYYy¨¾¾>00PÁÉJ²è=öíÛ·¯©©éêÕ«/½ô¯¯¯N	@ÏÌÌd@²¬:Ëår;vÌÜ|7n¸_DDÄÚµkx¡!d%YüK½$«­3;;;''ÁÉJ²è2­PmG/Y²dÇHV@g]½zUâ3&&&66ög?ûÙûï¿oºxñb`` ¹t<@²¬àÊ+R§fggK|;w.))ÉÏÏ/!!AêÔµk×íÚµQÉJ²è¬ùóçKZÞxãélÄí3g"¬Ý ¢¢Âår9Î¨¨¨ââbëCeeeS§N¢££e1èëÜw¬>|Øý"ðÉúTRRR¤jhâÄú!÷ïß?yòdi¸=vBB@_IVÛåùIV¬Ý,88¸µµUÍÍÍ¡¡¡í-æïïÿÃ?¸öõ×_çèÍ*++/ðoÿöo¶­ÁIIIë×¯g@²v'Fm«òòò¥K²5è;&¥jtttllìèÑ£Í&©Vcbbzâ²ÀNVÃaÚ¾¾¾îÜ¿?%%åÁ$+Ðçèd`sm¸	×ÄÄD	ZÉÔ¬¬,b$k÷0ÁöØ±cuk°´mKÞ¾ñâÅwîÜan0Ð­X±bõêÕÖ]»v%%%12 YÐÂóóó¥!·RZÕÛ¹sçÞ½£n>jþüùzk;iÒ$F$k*--		q8.«¬¬Ìµrj½Ê#Éô9Ë-ûâ/¬=3g#3Eð¬aaaæõõõÑÑÑ[·ned@²¬¼´~ýúÀÀÀÌÌÌ¬¬,Ë%U,×ÉJ²x*çÏßÚód%Y$+ÉÉJ²HV@²¬¬$+d%Y$+É YIV@²¬d¬$+$+É YIVÉJ²@²¬d¬$+d%Y YIVÉJ²HVd¬$+d%Y$+ÉJ²HV@²¬dd%Y$+É YIVHV@²¬d¬$+$+É YIVÉJ²@²¬d¬$+d%YIVÉJ²HV@²¬¬$+d%Y$+ÉÉJ²HV@²¬dd%Y$+É YIVB²VTT¸§ÓU\ì¾À©S§|||HVÉÚ))))ÒÈÍÍMMMµ=ÚÒÒ2uêT@²vVpppkk«4CCCmnÜ¸qË-&YSÝDDD$$$ð YÿÅétzl7oÆÄÄHî¬µ³iûúúZJNN>öì??[$k|~$í±cÇ677ëÖ`i,L²HÖ'X¸pa~~¾4ä6%%¥½$¦f¬RZZâp8WYYÇ(%Y$+g¬$+d%YIVÉJ²HV@²¬¬$+d%Y$+ÉÉJ²HV@²¬dd+W®Ø~Úf"ÚÎ1DO?DCÔ«høðá$ë?Ý¸qãÿu«_~ùÕW_ýh_ddäk¯½Æ8tàç?ÿùÜ¹sL0!11qèËåZ°`ãÐ1cÆ¼óÎ;ÝøþóHÖî÷æo.Z´qèÀ¯ýëßÿþ÷CâããW¬XÁ8tàW^ùðÃDEE­[·qèÀÏ~ö³Í7?"YIVd%YIVd%YIVdÉJ²¬$+HVd%YA²¬$+ÉJ²¬$ë³õþûïggg3ø÷ÿ÷O>ùqè@zzzNNãÐßüæ7[·ne:0þü;v0HHHÈËË#YècHVHVHÖþ§¢¢Âår9Î¨¨¨ââbÄ(++:uªLtt´cÕS§NùøøðçäÑ£G/^ìëë^RRÂ¹aÔ)--e¬îÞ½ÚÁ×uÉê½iäææ¦¦¦2 ÆÄõû÷ï<y2cåQKK¬delÖ­[·iÓ¦ÖÖVÉÈ]ppðÍ7¥!·"õYç0ÿG¦GÇdª?kù·Fss³uåVþþþG7nÜ²eùçgl¤¨®®æ?®²ÂqûömiÈ­®|0D*11ñÚµkÖdu+ÕN§ÓcFyyùÒ¥K+wRdÄÄÄÈ?¶ùçgÜÿ¿¶mÛ&kfUUU»ù[ù_cìñfIV÷éÑ±"Y½çp8LÛ××±¹ÿ~JJÊ+wÉÉÉgÏµþó3Dîÿ_zèaMM¬0Dî¦O®e½Dì3¢Õdzt¬HVï;¶¹¹Y7&H±ºûöâÅïÜ¹ÃXµ÷?oÅyüÿ²YÏu2YÝG¦GÇdõÞÂóóó¥!·R1 FIIÉÜ¹sïÞ½ËXuþ!²ÉÌÌ<pà4._¾Ç¹:UG.]ú!ê YÝG¦GÇdõ^iiiHHÃáp¹j+È«'þó3D6÷îÝKNNR,&&æÚµk»ÚÚZ	T"¹6CÔA²ºLÉÉÉÉHVHVHV@²@²@²@²À3ü?÷ñ>zJÂÂÂ½÷JÏwß×ÁòzyèeÛW­Z¥w<x wõªÉ _9uêTr	QI»ªª*¹ÕfYSðøñã¦'22R¢7½ÍÑ£G9"Ì7Ïô|öÙgÒsïÞ=ó#¥¥¥Òc.¢ÏYTTÄø$+Ðß<zôÈZVVVVÊmyy¹6ZZZ4%u±7oÊÝ[·nÉí¬Y³´Ózy]­b³²²l=Àâ¦NLLö_~)íÜÜ  YôwîÜÑFuuµ$ÜíÛ·åöòåË'NFcc£-Y].¦¯#FURFEEiÁZPP =.]²U½ò&Åïß¿oNdÐh©*ùzàÀ___­/%uï½÷lÉjjP½R´n=ÖW/9®»i­þùçÒ)¡+mãíÛ·K&±4:Ä/$+þãîÝ»Ë/Û¿¿F£TvÒ*Ó=YåîÙ³gCCCu£î§~*555!!!©©©Ò°>yssóo¾©õgm!-ÏÃo$+~åøñãfJîgÍÍÍÆÒ¥K5YO8¡KJ|Ê£ºÝXw¬ºøÆ¶àXnO>m])UÍt'-yùdÐ¯hF¦¤¤høÕÖÖjJXJÙ*7oTÑý²GÛè%mKX¶¶¶ÊOI[7±Íª$+~BwµÉíÎ;MàmÜ¸1<<ÜÜmll4?¢±ZXXèþTwîÜÑiÃ---îñIÍ¬ÀøÇöñY±b6¦N*1B3,,Ì,cv Ê£rwáÂ& ­19qâD/AÍ¬À ~¤]\<yòd­G¥_U3OªO³«Ug3i=ZUU%·²nòµð¦fHV ÿÓÙIÖ9G»wïºººÇ?Q¯qhÝºïÞ=í45É*+=Ï]­Y/_¾ÜA*$+>ÃðÑ£GQQQ%%%Oü©¢¢"-s%eùððð¨±±Q=z´²îÝ»WO (ËÛjÖ+Vèé&ød0àL<¹  ÀÜÕS5­[·ÎôÜ¿ßV¶êÆäüü|Sdddd$+$+$+ Y Y YÉÉÉÉHVHVHVàÉÿ5YlÊëÝ¤ãIEND®B`


RÒ!Ísrr²îy«»^XX(cwÓ-!ï<¤ýò³s¿÷¹Xú ¬À¼é/2fÒQã3gÞxYe8eqú)ëºuëleA­LÇÇÇïÝ»W?4rîÜ9½N__ß­[·tYëàUæÈÃÆH6ôG¬WÐ±®åÖwr;ºIR÷­µ¦EÇy7oV?~­ÕÝtwïÞm-Ö.þ#(+0Ï¿WË«­NIIÑqéYðtwwkçZ¼Ä_ºt©©©éÆ:§¿¿_WöÖêâ?þØÄþÞ½===:tÓOÝ+ºº:óhå¥/_­ÕÕÕºEV?å©ÙµkÌV­Z%s¤dr9k§î:¤+f¥ýR/sÜ¯fo^÷>Kÿé^^eè#Gy	È¼n««¬¬ãÉ+¾íA¦åÁÓBH9>ÿüssd>ÉÐÖétc ´´4ÆYÃo¶hJºººMÔõöe¾^MÆ|syüºRº°°p^÷>Ç·KüÊ(+Êeu?ãàõüú×¿þÏÿüOÊúòþé6mÚÔÀëùÝï~÷Í7ßPÖïÊúw÷wÿÀëùýïOY)+²RVe¥¬ÊJY ¬@Y)+²RV(+ePVÊ ¬@Y)+²(+ePVÊÀ)kWWÛív:)))---Ö:::Ö­['¥¦¦ÊÕ(+²ÎÎëõVWWËDYYYnn®õ¢µµµIII2ñM>ú²(ë"##'''e"x<é®¶jÕªo¿ý6,>ø?eýÓé9mÕÙÙ¹k×.ðùÃþÀÞ¬±±±¡¡!Êú®Õápéðððà+<öÌëõ>þí¬°ØrrrWll¬Ûí.//§¬ï^Y£¢¢®iÛ¥ÃÃÃ;vìaß`Xl~¿?11±¤¤D¬íííÒ××åSÖüüüªª*SZ/òù|7oåS7ðHD·nÝj#q+eÇ´µµÅÄÄ8ùåutt|ÿ4Â¾"Çº§eEU\<Baëàà eåHy;pàÀþýû­sü~¿ËåSÊJYóÖÚÚÑÝÝmÅfff®§OY)+¼yçÏAjAA^ÓÒÒÒÓÓWÈª`ÊJY`±Ü¿¿²²òèÑ£W®!ë)+e°ÜÈ¸pllå@Y)+¼®óçÏ»§¸¬¬¬	e¥¬ë×¯TTT¼C@yÀÔÆÆÆÿÚW|bbâ'OøURVÊàÇwèÐ!©ÔÊËË3226lØ°ô|^SScsêÔ)~²øµ¶¶FGGß½×Ì)(((,,;""Â6¶ÑvQQ¿PÊJYüÈL±ÎXúå·¾Ð'RZZÊ/²RV?²å[½zõÒØ[·n5ÃÖ;wî¸7oò¥¬ÀL·­Zç?~ÃKüaûý~)«ºsçÎ~²XJKK+((¸ÿ¾¥On·[÷¹]ú®rôèÑS§NÉ_%e¥¬¡¡!)«UF~ééé>eÊJY²(+e²RVe¥¬ÊJY²@Y)+²RVe¥¬PVÊ ¬@Y)+²(+ePVÊ ¬ÊJY²ó644TRR²aÃ´´´;w²LÊJY¦fgg···wwwÅÆÆ°dÊJYøòË/³²²¤¯fÄUF®,yçqêÔ©'N´¶¶²4@Y)+0-¯×[__o##×´´4Uqq±Ûí.-g­ïEÊJYÿ¯¬/^´Î¹û6eµëÍæç¡¡¡ÔÔÔ°d@Y)+:éééÖ9¬¶ÊÌÌlll´Î³¶PVÊ|olllëÖ­Ò	¹^¹r%;;[¬ìl%#Ô;wîXçÜ½WF±,PVÊLW¹æL9tèÐ'OX&VyyyGµÎ9qâ×ëeÉ²RVÑÝÝír¹*++u¯¥öövÌ2044$ï8`[3AY)+EäóùRSS£§$&&Þ¼ye²<Þ3¹Ýî½÷Ê&ÛuÊJY,®'SXË¼[«9+oäb)+eÌÛýû÷ãããmKÞ²RVÀ´··tª   ¦¦²RVÀ¼=yò$""BF®fßïw»Ý·oß¦¬°û÷ï7Vòz½yyyKêRVÊ`ø:#5:uêÔ»ey.+66VNeØJYEWWÛív:)))---¶KGGG=e°bÉð.---++«¾¾þâÅ6léwz¯é¥ùæ`ùUÞUWWËDYYYnn®õ¢¶¶¶äää°°0Ê`Å*..ÎÎÎ6)Ò¯øÁK²N+22rrrR&mxºeËXËú7A¼ãÀr%µJF®999,Ê:-§Órú§d)ëgAt%	qYm;Ð^¹r²RÖ83>sYY`¥)...,,´ÎÙºu+k)ëL¢¢¢®iÊVCCCñññW¹¶··Ëh5--ã>RÖäççWUUÉz½^Ê6÷ïß²¦¦¦JS÷îÝKV)ë,ÚÚÚbbbÛíîèè®)ePV ¬@Y)+ePVÊ ¬@Y)+²(+ePVÊà-ðûý­­­wïÞeQ²RV¯ëèÑ£éééSlßÙPVÊ`N:åv»»»»õlyy¹ËåbðÊJY,Prròõë×­søú3PVÊ`"""ls***vîÜÉe¥¬8fmmmµÎ)..fÌÊJY,Ð'$®zöÊ+ÑÑÑlge¥¬®¨¨ÈåråååeeeIVY& ¬ÀkAjMMXX ¬@Y)+²(+ePVÊe¥¬ÊJY²(+e¥¬ÊJY²x÷¥¤¤8Î?û³?ûÅ/~qøða(+e°p¿úÕ¯~ò|òÉ'555ï½÷^XXX~~>²Xþç~ýõ×fÎÖ­[eK²Xßþö·ÑÑÑ¶RÖÎÎN(+e0o7n´Î²þ÷ÿ7²·ÿ÷Ê©óÞï9(+e°@IIIW¼þñéÓ§O³X@Y)+ûè£Ö¬Yó³ýì/þâ/jjjX  ¬@Y)+²RVe¥¬PVÊ ¬XiN8QTTtàÀÛ·o³@@Y)+koow»Ýyyy§N*--u¹,PVÊ`bcceÀjíêÕ«»»»Y2 ¬À¼Ý¹s'11qllÌ:sçÎÖÖ²+äpÿþýÉÉÉ2ÄLMM­¬¬ÈØ4==Ý6³´´ôË/¿d	²RV`eÉËËËÈÈÐÕ¶×¯__À@Óï÷GDDX×ýÊ¹©ÆÆF0(+eV7oJÿ¤ÖÑ§^<y2ß:uêÛíÖÞ¹s'+++;;Û¶~ ¬Xæt?^ÛÌôôôöööÜZMMMjjêÏþs©uIIÉò¬Ä²ÖÕÕMLLPV`(///**²ÍLNNæÓ¨ÀÛ+«ÇãyÑäää¬?ÞÕÕåv»NgJJJKKËÌ-leæîÎ;¶í£çÏÿ)Öâo¯¬ûöí[µjÕ=¼õõõUUU2---ùÇåÊÕÕÕ2QVV;óESäý÷ßîVåO>ê¿ÿû¿/,,LOOÿÕ¯~%ÿÈ, ¤¿þë¿~óe|®_¿~Á?©CÛ@  Ãß/ZÀo¿ýöAdDÀkX²nÚ´iÁ?.9ò¢Íam0àYüâÅv½mmðñãÇe~ÿÌ·àp8ÌtxxøÌ-le¼KÛY/_¾ç744HYgþñ¨¨(ýq9é/ZØÊxÊ:Ý.Áúi±±±¯_UU%r*Ý/ZØÊxÊÚÜÜ3Å¶øóÏ?×¡ªfddÌz;mmm111Ãívwttm·!/ZØÊxÊ:>>>ëMs)+GPÖ¹î3uá?~péÒ¥ûöIYzz(+²¾	ªJVoÝºÅqïºàÇ+kggç¶)fß`¥Ó.Áët¡¬ÊbÝ¯ím$¥'OdÌ ¬ol`Ê ¬¥QÖÉÉI©éîÝ»ËòxÂ@Yç1NÕ/«Ó=t²(ëkµÁÊJY³'OÔÔÔ=zTNøOÊJYîöíÛñññYYYû÷ïÏÎÎv»Ýíííü3e¥¬á÷û%¥§N2s***#WxWË:ë7SÖEuóæÍôôtÛLÓØØÈÿæ«µµµ¦¦FNåÿ¥,JYõËÏuà´´4³?°®ªª³N§²þX.^¼gYPP ¯ü?`îîß¿/oÈRSSå'99YþÇY,À/ë¬_~ÎÚàÝ;w¢££<ybæèúáÛ·oóÿ9jbbâÞ½ÍPµ¸¸XBËÈø_¾ëfeîÁ$ÌÌÌ»wïÊôÀÀÀÖ­[G±xGýñôx<òæéoÿöoé^Z[[e´jí¨¼?;,¿²®Ä²ÊbIIIDDDll¬Ëå***EþÈÈÈüä')))7nt8N§sâzñâEyfáóùø-[Ö°°°mÛ¶QÖ%K¬¬¾[6>úè#Éê×_­g¥©?ûÙÏâââã¾ô[Ö?¡¡!y¯Æ¦VàmõÈ#Ö9/^¼ ¬Àb°½ÿþûÖ9ÿú¯ÿ*#×Eº»¬¬¬¼¼<Ý`/§999ü7Ï÷áÝ¸¶¶VÊºqãFóè2Gî²oÜÏþóO>ùÄ:§¦¦FF±twZÓèèèËmÝ- ¬o¦¬ãããÖï<¿páBdd¤üï±6x<Ïÿù[ç¼÷Þ¿øÅ/õNå-õÀÀËX²üãÉÄÈÈeÕ×_-ÿkIIIº×R~~¾=|ø0KX&e¢ÓMMM2ïÞ=Ê,ªÓ§Oÿô§?EâÊ2CYÇÇÇå_:22R¾tùòeW§ÓùàÁÊ,ªÿùÿéììd9Ë¡¬O>­««[»v­üWë«W¯JPezdddïÞ½r6<<IYu&£££kÖ¬Ñ£vwwK>?~,=wîõjYu®¤¦>¯¹¹Yê÷ûe+TI¯LQVeÇK×®]Ó²ê&U«Î²öööÊÄ=(+±Û·o×ÔÔÜ¼ycFõuËªQåÕÄZVUYY©ûËücÇQV,KcccÙÙÙÑÑÑyyyééé±±±ííí,²¾OÝèVÝé_äææÊôÚµkùÔ·ÌÌLs(¢·ÛÍÊÊwÝ¡ßÍglÝºõüùó,²RV`Þbccm3÷ïßèÐ!@Yß@YÍnMËËå²õwvv6cV²¾ù£Ê©,eÅ2r;«eY2eÝ²ú|>©i[[ðàî3LY±¼ïÜÚÚÊb(ëëUaûhÍý+eÅ×ÞÞ~þüù7o²W0@Yß@Yõpü»wï[¯×KYu®?®Ç`úp÷úúú3gÎlÚ´I7¾vuuY¿ ²(ë´-õ÷÷ë×ëÙõë×ëE/_³z	·Û-¦¬Ê:ïÐ~öÙg:áÂ9ûìÙ3Ö(ëB<|øÐúaNNNf&e]øuÛ¶mÖ³iii@Y¢¥¥ERúèÑ#kYsrr(+²ÎÛ¥K¤£ºS¿­¬¬a¢¬%WÖÇGFF^¾|9øåvíÚÅÑõ]ÒÕÕåv»NgJJJKKKð¡*<e¼Ûe2`Ý¾û[(«×ë­®®²²²ÜÜEmmmÉÉÉæ(+à]-«naíííµÎ|ñâÅb522R7îJÎmÃÓ-[¶è·èÙo¿ývuþô§|ð¥UVÏgmX__/1	ë­_0÷9ÎÓÖýÍtu÷ß?++?ÀÒ*ëÄÄnhhÐã.Éxñ-ÉápéðððËÊÚ`À»QVÛÑùõ»äªªªéÐa¯ÈtTTT ÐµÁ2MYËª¬»wïÕ××ËrÕªUõSdzóæÍ1fÍÏÏkÈC~]e¼«eµmLbòºemkkq8n·»££#¸¦ðNµ¥¥E:*õõõui@²bY>Ie²®LíííYYY.+::º  `ppe².°¬£££ò2jKYWfV#""ÊËËÇÆÆäaçÎ6lðûý,õu÷`²Õãñ$%%Ý"3e@CY«ÌÌLÉªuNFFmPÖÜãÇeÚívKY'''9"3ËÊÊ(ër-£UëÉjqq1KeÇH²&$$è´².Wò»¶mX=zôhII	KeeqjAAß"·È/º°°Ðòä´öÊ+,õÝû9Êº¥O)//Ñªduÿþý,õuË*T¸³===2ÇçóQÖ`ll¬²²²¸¸¸¤¤¤±±²¾nY¥£¶£û2fPÖyÏyiiiDDDAAà°¶¶V¿üÜãñ¯³g)+fÐÝÝ]^^~âÄÖÖVXÖÉÉI	g]]ªÊMËéöíÛ­ã×?þ²bVr¹ÅÅ÷îu»Ý2aû<,ÿ²j8ej¦¯^½j>Õjf<y²bf×¯_½sçJMMÁ+KÀJ/k\Üèè¨L§O¦¬YQQíÈM,+±¬			:ZSc®ðÕW_QVÌ,;;Û¶_ñÝ»wY2VúU#:00 Óú©2qäÈÊZçTTTlÝº%²~?<03?NY13¡FGG×ÔÔèYÏçr¹®_¿Î@Y¿¾Â_|AY1«ÆÆF·ÛºaÃÉêùóçY&VhY­ÛY?Ã¾Á»îîîÛ·oó¯VhY?ÏzæÌ]S]]½÷nÛp(+²NK*á¼wïñâõÒsçÎY×SVeå»n ¬ð®5,,ìÓO?µ®"^´¡¬w ¬z8ÃcÇ9½½½¶ï£¬Ê:¿ÞÈÃáØ¼y³~gÓ¦M;vìÐâ´µµQVeÇo. ýðÃ»Ëqu®êêêÌVÕ°)2N-xÅz~Ê ¬³XsssõÏµ¬¥¥¥¶ë,Í¯£¬¥XVIi¿VWWëYÃÑÐÐ`­2QUU%ó>JYu&ÃÃÃ±±±Ö-©º6ØçóÉ©î¾ÞÜÜ,g<x@Yu®#×YËúðáCÊ ¬s-kgggÿ=V­ZõùçËûö­Y³æÓO?em0²Î»¬rÚÝÝ-§×®]cÌ ¬o ¬=ÓúúzÊ ¬¯UÖääd¿ß¯ÓRÖµk×VUUíÝ»WfSVeßL:mýk 0É¡¬Ê:gÏµßO#E(ë¼½xñBRºûv=ûüùs9îÜ9½²(ëLLL¬rõêU·Û½k×®«S$«ùùù2Q]]-Ór¯@Yçzô`kh¥£gÏ¹äQVÀ-ëèèè¦)2B=räµ°°P¬ÖRVe@ `öZÚ²eíÒ±±1Ê ¬srëÖ­Ü)æÛXeðjý~Ö.èwàPVeÇhU¬ë×¯ù ¬¥XVãáÃRÖÊ ¬¯[VýôjSSùÎsëLuuu@YçÕªªªÉ)2gÉ~Û9e,õ²úý~¨íme ¬Ê:»ññq)h [Ü¶mÙCøàÁ2çÎfañÆÓØÕÕåv»NgJJJKKõ¢uëÖÉE©©©r5ÊxgÆ¬fTj=ÓÛáõzõ[tÊÊÊ$êÖôIÖÖÖ&%%ÉÄÿ	Å`)õG©9A³Çãîj«V­Ó¿	âr¹(+²þÀét¶êììÜµkkuvÖã:_áÙ³g^¯÷ùóç@YCE¦£¢¢ôPr*Ó¶kïØ±cdduNòóó«ªªdBNelj½ÈçómÞ¼yttOÝ(ëµµÅÄÄ8·Ûmª¨ÃYÇfAY#E(+ePVÊe¥¬ÊJY²@Y)+²RVe¥¬PVÊ ¬@Y)+²RV(+ePVÊ ¬ÊJY²(+ePVÊe¥¬ÊJY²@Y)+²RVe¥¬PVÊún¹~ýziiiIIIEEÅØØe¥¬X¸¢¢¢ØØØ/¿ü²¼¼<##cÃ,²b!dhMiAA×ëeÉ ¬WYYi300àr¹X2(+eÅBÈð´±±Ñ:gll,""%²RV,ÄdØjS^^Î@Y)+âÉ'EEECCC2Z­¨¨«ÏçcÉ ¬488XPPàv»¥©2Z%«(+ePVÊe¥¬ÊJY²@Y)+²RVe¥¬ÊJY ¬@Y)+²RV(+ePVÊ ¬?®®®.·Ûít:SRRZZZ¬õõõ­_¿^/jkk£¬Ê:;¯×[]]-eee¹¹¹Ö$¨2!O522R&þo?üá@Y É@ àñxB^§©©)55õÛo¿å>à@Y¿çt:CN«5kÖH>kkkuðjóÑG1fPÖ83ò:ÍÍÍQQQlgPÖÐÌd]<]>Cg)+²_UU%rêõz­%$$ôõõÉDGGÇæÍ)+²Î®­­-&&Æáp¸Ýn)¨ÔêrRRRd´ºiÓ¦Ê ¬)@Y)+²RV(+ePVÊ ¬ÊJY²(+e²RVe¥¬ÊJY²@Y)+²RVe¥¬PVÊ ¬@Y)+²RV(+ePVÊ ¬ÊJY²(+e²RVe¥¬øÁ;wÊËËO8qýúuÊJYñZ***V¯^]XXXZZí÷ûY,(+eÅB´¶¶FDDÈ©¦feeIeY2(+eÅB8p@ªÖ9wîÜq»Ý,²b!ËËËm3W¯^Í@Y)+B²ºuëVëúúú6°dPVÊðûý%%%O<³.ëÊ+,²bîÞ½!AuO¹xñ"Ëe¥¬x×¡¡!ÊJY²@Y)+²RVe¥¬ÊJY)+²RVe¥¬ÊJY ¬@Y)+²RVmY»ººÜn·ÓéLIIiii	¾BsssXXePÖ9ñz½ÕÕÕ2QVVk»tbbbÝºu@Yç*22rrrR&Çã±]zäÈãÇ²æÏÊÊâ@Y¿çt:CNG¥¥¥Iw)+²ÎÃá0ÓáááÖrrrnÝºõÝ³bm0²Î ìº6X¦C^Í²(ë,òóó«ªªdBN½^ït%fÌ ¬sÒÖÖãp8ÜnwGGGÈRVeåHÊJY²RVe¥¬ÊJY²@Y)+²RVe¥¬PVÊ ¬@Y)+²RV(ëkØ³gÏ/ùË/66ö¯þê¯XoÁ_þå_ÆÇÇ³ÞßLa9¼ò'-Ø,Õ«WSÖï<|øðO+Þ¿ýÛ¿²(Þµk×þã?þ#Ëá-øíoû»ßýåðüÃ?üCdd$Ëá?þã?ÆÆÆ(+¾ã÷û¥¬!ßjáûõ¯-oeXo×ëÍËËc9¼ÇÿÍo~Ãre¥¬ ¬²²RVPVÊJYAY)+(+e¥¬ ¬ ¬²b6cccÿõ_ÿÅ¢xÞÿýóçÏ³Þùa9¼úÓ>øàe²@Y ¬Xþ:::Ö­[çt:SSS»ººdºÝnÒÒÒÂ"z³ÃÂ¾ÿÿbQ/ñññ;vÇÅÅù|>õâÅ¬¶­­EMYñÝ¸¶¶6))éåÔÕÕÕ2QVVË"z&&&ä)+z<xðØ±còºÏ¢^<=	9õx<,jÊ»U«Vé¿¼$ÉD Ð¼)G9~ü¸)+zÈhéÞ½¶°¨¼q	9Õ71,jÊtvvîÚµK&N§iÆk7õiiiò¢cÊÊ¢^$²0¿úê+y§(¯õ===,êÅÓÕÕ%ËYþ¤åT^CXÔ?xöì×ëþü¹L;3?<<ó¦äääÜºuë»ÿ®WeeQ/Y°2Ñßß/ïfXÔgýúõºz@»qãF5eÅ÷wìØ122¢g£¢¢ÀË©92ÍòycÿTÿ?õâ±.L6±¨oõ²ÂÎçómÞ¼yttÔÌÉÏÏ¯ªª	9,h1Ë¢^TÅÅÅuuu2Ñ××Î¢^<2N,½½½2~eQSV|ÇãñØRmmm111Ãívwtt°¯¬,êEòôéÓB¥¥¥=xðE½x$¨²¨åT¦YÔÊeÊe²Êe²Êe²X2ÿçaaë×¯÷N©¯¯¿páÌùæof¸¾~uÌô«Ý÷íÛ§g?.gõÛRÊ`YinnÎ"ÚõôôÈ©~áµ×®]3s%½S®^½ÚÐÐ WØ¶móÅ_È§Oikk9æKNô6/_¾Ìò(+°Ü[ÝÝÝrÚÙÙ©ZA	°^íÑ£GröñãÇrºiÓ&iý*_ÅîÝ»×6G¬7ãà-[¶ÈôÙ³geº¬¬ß(+å`ddD'îÝ»'Ó¾¾¾¦¦&ðûý¶²ºÝnj_×¬Y##T!iJJX«««eNoo¯mÔ+·i*þìÙ3ëmÀò¡CUék]]]xx¸/¥º÷éÓ§¶²1¨~U»®=Ö®~¸n¦µ^ùäÉ2S¢+ÓãÓ§OË-±Lt_(+åcttt÷îÝR¸ÚÚZM£8¥v2!£Ìà²ÊÙ[·ny<]©øðaÙßß+Ö~ø¡®@ÖÍÉÉ±EZnß(+eåÚµkf"ÝÎZVV&»víÒ²655é5%r©®7ÖM¤f#«®~øð¡-2Ó7nÁ®UÍîN:äåWÊ`YÑFz½^ßÀÀ&Pb)ÃVxôèêut»l||üÕ)ºNDFFJ,'''å§dZ¯oÄÁcV½ ¬	ÝÔÚÑÑ!§gÎ1Á;räH\9ë÷ûÍhVëëëojddDwÎ'cV²+à;,¬¤¤D'Ö­['uuukÖ¬pÆÆÆë¨r©ÍÏÏ7´f2!!!ä]0f(+°"èß±±1niiIJJÒñ¨Ì²jódôi6µêÞL:íééS¹®òëñfÌPV`ùÓ½¬û;wNæ¾|õÍ¡uíîÓ§Ou¦ÔÔU+s$Ïó³öõõÍPe²xgKSRR|>ß¬?uùòeæJåúqqqYYYzßïÖ®].èåú¶1kIInß(+'))©ººÚÕC5<xÐÌyöìmØª+«ªªÊe²Êe²Êe²Êe²@Ye²@Y@(ÿ¶øGIEND®B`


APACHE评分


正态 Q-Q 图


¥Ú³âJÚFÖ3	ùøñãÆ»wïÊÝ»w÷²ÿNÖo¾ùf~7s&Nü±L¶ßdÆ[o½¥©¥>|(íS§N3?~ü8¹W!h~Þ?üP¦HIpïÿY Ôå2gnn®ùIåíó]¬Æÿn÷òÊuº×dÅpÊÎÎÖ-rûöÛo÷°Æ|ðàAaa¡Léåjjj´ÞÝ²eË£G´¾RHõ5YSÏ-_¾öÅ_ôJVÍ<¯âêÕ«2ýÈ#^!mdO÷ÑGÉ¿#+zùdâºuë´7|Òb.°dæJQ<==ýÒ¥KÏúL^y³ôgÍÖ­[õÕÞ¼ySS_SÖüWúîÿ5ùõÓÃôï?ÒíÕF²J0kÓ¥Ï6É#üäuhggç)S¤m^úæ®e¶^.A*ß°²N=¶î'«yÝmª¹áv»ýòãÀkº¬©eºßÛæ§0vïI;))IþwóFi.ñæ~R8ö&Y½|ñÅ²@Ý*m¿óèk6ÂÆ¨óäöÎ;r«Û¼¶©jnÉCO»wIãöíÛ2Q²Sòóh zOÿy=Ú~VlÉ*¥¶ñúoÜ¸¡?_ä§É#¬CÒaÅ¾©`Î³ööv­Yµ8ëÍN§yø¬Uç@%«nó<qâVÒ$¾.Ùårù]ÂâÅåÑôðÊ°öÁåöèÑ£^óøîÙ5W^ÉÚCÆh®lÚ´IÐôp >:mÚ4.M>ãÝ[ùàÁ¯¿:wî©üGú'úÎ~øá«ºÝu@zO,Y²ÄXÈÒ¥KNÖãÇkÏÈû8kÖ,xñâEó<$+HVÆñ&zÊÜ¹s½²Í@½°ÏMDK;¨¬|µ,jØ¼dR-ÅtË¡TQæD?²Z7öóéþK]äÖFB~U<7Y#""ªªª¤ø3êÑuÞ¼yÚþüóÏ5#c¬ü°õõõº(cg¶dä÷çÂ#Gô A^Lº5µ  àÅ_üàä®,ÄëoågÇãÑ$ù¸÷>ùä¨ÇéÌ3gÎ4Ú¾»$ý¬R¹»í½æ!YA²bDµ§äy§i\L1Ö¶æ´ÙlRñOÆxî&Y5µ°dCz7ce-§ëý »¥ÕÃzXÓÙ³gå!Fã5ánÜ¸ÑÃ+2e×þTÝ lTÏMÖçnXüÎ×^Õ½z¼RWWnwÕíÒÆÖWy%Æ¶S!	§¯P«ò»ýöi÷A¹aaaÒúòtºò¶&$$ÜRËÿnäýSêæßFæd²Uÿ©õFZZf6É#VK¾ój»ö¹KÚÈØÖs²ö<½÷G0éZØ¼éÒï©B^gwx'ê÷¯z®Yù¥½páÂ§ßïô=Õ²É*!'qlþw$$Ànß¾mLG%ç»º[þAÝ$.w¥aìXMJJòªtuS¹þBÒ÷4::Z¢ôÜ¹sZP>ý~³¿nØ¸÷4ì5¹CÉ¬úïIeÝ¼/¿äõkBkñíP:@²bHUç7ez/óì¹KÐm^Ñrùòe©ã&¿C4ô&#u¶üä'æ»æÍÚ½IV)¡¼fÐãn~ûÛßö§f5*?Ý«f+Å¢×¨ò¨n:Ö°Ñ%/u½Üêù3^;¿¯^½jT®ºGS÷eÊÂm¹~wµö¾÷ôØl=ªÙ«ëzNV=jÉ"?,`6¶UP³dÅðÓÕ¬×ôiÓ¦UfÏyÖ%èFÂÐÐÐ­[·JÌH'kv=¬ÉXR²ô&Y%<¼¶5àp²jÉ¸cÇþ$«VxzÔnX6zãÞ½:ÝXnÖç5ÎgÍÎÎÖíº§NÒázl°q(µd°ßä×¿ïBozOçWë7M³5XlÚ´Iìµ!dÉA-,,ô^PP`Úsõf	BÏsõ"q"uU_µç"µ°¦±Ô÷æ¹²öÚÏê5VÃÓïú3ÅÚd5£C%%%HfÐ±ºÉWÝÕA´F²êféOËµpáB­¤õÜV­uÜoÇE÷³÷KVMhÃk4	$+ßÌ3Msfz|¯Ûs²öf	j÷îÝR5FDDèáNùùùæðØdÕ¼7éM²>ZwþStwàssÅ+1¯_¿.ÿ¦'$ÁiÌ_UUõÖ[oiËt=¨Ê8ÀJZË2ã¯ôø^=jÌw»ºq¶×ÛOÿt¦ÜÜÜ§ßé £SÉzöìÙþ;b¢gõW×ÉgFºK/ó>Óï~=ä»´´dÉ®¹sçêy¥Ï]5S_Í¸+y°dÉ¿[¹ýÆ±ÍÙë=vÉë´Q¯úJOQRòÊßsTÌ¯Í<ÚTMMÍf3¿6ùÐÃ ¾¾ãUTï07RVòÒØÝëõËkD~ÝÐ|OïHV ¤Ü1W«ÆªÙï@­­­RCzO¿?8Öï	¬£ia*éâââ>øà>ý¹î¾íáÛì=óKõûèâÅ»¯:¬¬¬¬ddd$+$+$+ Y Y Y1ëÁz1íÿÿu²XÖ­[çwæÇË£×¯_¿yó¦4Z[[ûóÔO<ñíRYòéÓ§ÍWgùý¬À@|->úÈ<EÖÎ³»å~ïèÑ£G9óß<k92O½±LórwÓ¦Mæ»999Ò¨®®¿:pà^^[ÚÒX¸p¡y±¡ÝvGßyç=Ò>qâÎsÿþ¹ÛÔÔ8oÞ<ÁÉ³gÏÊ£æg÷zXWW'·ö^ÆþHV`àÉJÖÈHYçJL×ãæòHutth#:::%%¥å ,J¯Ë¬©©ÑMïVTTèl÷îÝ»R8:uêîÝ»¾1cÖÜÜ,îß¿¿ªnß¾­3Ë­í£G¤]ZZúðáCy"ÍfÌ)qe·ÛïÜ¹ÙÍè­[·ÊÆáÃûúì#¿`FRvuuÉú766VVÜK,[¹ûùçË­Ôv¾Øûdõ*/]º$±1kÖ,½«Y.¡kIz¨/@²!$$DÚæ<~ü8??_-((0^ªTlÚ0¶Ê2ÿN·Êí7ß|#·Ç×¤4'«Ñælj=àíCÖ?É¦¦&ÝÎ)¬voÜ¸ÑsÍÚ§dU¼¬ÍµòvÉ¡m½H¶éæM¯0ÐO¸ßäØ½·ù_xçwtsnkk«Vº9TËDMV©e¥½wï^óÆa¿Ð§g®aïdç#û=·eË=FæÎ;^¡"«ònÆ¦ãÐÐP	Wi[McIkbÍÛ·o÷^!a<»W<|øÐAJêHdz<Ó§OasõêUßdÕ×©%fu3þ©7oðìFZÿ$+0XÞÿs &''û®-«½¯YWr|Ù79$¹µÆÌ²RWî.]ºÔ(³ôhÝ¿h¤E\äÎmNuñâEc6YòáÃe¢ÍT__ï7Y±ÛÕHYiËShoøgoä÷@²BªU­vÓ$mÝíÚÏ­ÁæäjX³M"Ä79ôÈÈHçi÷ÎN)10×dííí(sçÎqÒþäOtË§¾~cß°¬¥§nî~n²Jk´%«ÌQÔûgïeÍ:ìý¬ÀÀU¹¬j·mÛ&·æÂt`5''§ªªJR~=kk§VWZ«ÉôÐYßê977W#D6¯%Hé?%I©ñãv»õØ`Ý©ì¬iiiyyyR»Éª¿0ädümï½¯5ëÐ÷@²BxªN«¢;vx²JÁgqz$ÇÌ3½C6i»ÂÂB=iä/¾ÐyêëëÏ;§Çô3"/CRPbCÿÄ<Vº[=Èrt§©ýëuÖÖy³gÏ4Ý´i[+«¯X±ÂX½yö^ÆþHV`>¯¦µ­ÑNLLÔõµïÔÕÕézÜw¿døðá'N>Z§Ü¸qC7fY¢Bß~ûm#ì¯_¿~ùòe-.õ¬cqðàAãÕÊ%?~¿åVìÝ»W÷8êy¨1Ë-)òPDDL¬ »ÆëÔtÓ±dä«ñ<¯X½±`ÏZ½Jq&¬e%îwÝWWZZÚË'LòÃÈÈrß£ilûøãù´dÒ-44ÔA$''K¡i~c¦Ö¥KÜn·Zº|®³IUÚ×¯]óòòúôì½7¬¬ddd$+$«6m6mZ,#ÀôéÓÿë¿þkt'ëßÿýß¿ñÆ7þê¯þêo¾õÉú³ýì;Füä'$+$+É YIVÉJ²@²@²¬ddd%Y$+É YIVHV@²¬d`$ë©S§,É Y@ggçÌ3dýÆÇHVÉÚ[ë×¯ß´i&ëþð?sæÌá½¬ÏwçÎäää®®.£fõøøÕ¯~EÍ Y%;;ûÜ¹s|Aìg¬ýçµÕd¬±Ô¬d¬ÉÊ	 YIVÉJ²@²@²¬d¬$+$+É YIVÉJ²@²@²¬ddd%Y$+É YIVHV@²¬ddd%Y$+É YIVHV@²¬ddÚÛÛédQRRât:èd~©««KKK³X,ùùùmmmtÉJ²@ÚÛÛ7nÜîv»Ï9C¬$+N¢499Ùf³<:d%Y @---ùùùV«5++ëÊ+tÉJ²@à*++cbbGYY¬$+®©©)77×b±,]ºTÊV:d%Y @RØl6§ÓÉJ$+ÉýR[[fµZ)UIVúUª~úé§Rª&''?!YIVÜÉ'%PívûæÍ9©d%Y pmmmK.µX,óçÏonn¦CHVwèÐ¡§ÓY^^No¬$+®±±1;;Ûjµ0ü/ÉJ²@¿èI5©D²¬Ð_µµµ¨áááÅÅÅ©D²¬8ÉQIS)U322$_édÀ;vÌétÚíöJUdÀ566æææZ­ÖiÓ!$+É+--r8TC²¬Ð/W®ÌÌ´X,Ë/güdÀÃÿº®TC²¬Ð/./**büdÀéð¿V«5;;ûÊ+tÉJ²@à*++].Óé<tè½A²¬¸æææÜÜÅ"+IVú¥¤¤$***))©ªªÞ YIVÕj]½z5*'YIVèÂÂBÉÔôôtÿ%YIVè'O&%%Ùl¶Í73ü/ÉJ²@àZZZòóóõ¤:d%Y pååå#**jçÎôÉJ²@à¤<]´hªMMMtÉJ²@à6oÞl³Ù¤Z­¬¬¤7@²@àjkkÓÒÒ,K~~~KKäñxô¤·ÛÍø Y _tøßðððââbNªÉkjjÊÉÉ±X,$+ôËÎ;ív»ÃáØ³g½×ÐÐ Eª^©#@²@àÚÛÛm6Óé<vìWUUåv»­Vkaa!UÉkiiYºt©djjj*WªÉý²gÏÃa·ÛKJJ8©$+Î8©FnGÎð¿R@'''KÍvid0jHª'Õ¨á%VNg^^^mmí²³³y¿HVÀÈeÿ;OªÉëfµzõjÞ5d0ÿ=sæÌ|¾PII=þ|Þ;d0â<yräÿ+ÅôÌS:ÍÛG²¬Fã¤?üoQQT¨FðKCªØÞDd0R'ÕìÜ¹sä¿ZÇ#Q*¿ÊËË¥ZvVVç¬$+ÁþwDTó×®Ê5;;SlIVÀ`þwDTd0ú0ü/HVÀÀÍÏÏgø_¬$+P^^îr¹l6û&A²¬ú¥¹¹9//OJÕ~RHVÀH§'ÕíÛ·R©*ÕpaaaAAAYYÀÕØØ¨'ÕäææÞõÙÙÙn·s·¤¤$ó0É`l+**²Ùl.kPÿÝ¸qcZZÇãÑ»ÒÈÈÈX»v-oFM²^¼xqæÌ¡¡¡òÃðÒ¥K$+_UUU²°Z­«W¯ìj$G½[]²w£&YgÌ¡¯ïÀ			ÒøÄGJJJff&ï%0IH¦¦§§[?H$Â½jhhp»Ý¼5Éj!·ÿÇÇÔ©SIV`ª¬¬t¹w¤¯E§lÜ¸1''·£/Ykjj-[ÆÖ`ßu_©&77×b±Ì?ÿ­««,/--Õ,/++³ÛíCS.dH>="YÄÄÄHµºgÏa9(Wwëê=ñññ#ózé YÒÚÚºdÉ÷îql00>çÏ444¤§§K©o;ºñî`ô%«ü6=öýû÷9ëo¤-,,r:rj³Ù¤XÕ5p±±±?rss322%J].WppðücÊD¬ W®¹yó¦T#îÜ9»ÝN²d%YâÐ¡C²zr8QQQ;wîÔ#ÒÓÓÏ?Oçd%YôMSSÓ_ÿõ_[,¬¬,óI5.×d%YôMqq±Ífûá8eÊ=öÓKJJâããû$+ÉÀæææÕ«WKIºhÑ"#>ëêêRSS­VkAAAKKKmm­Óéy233¥MÁdà?V].dê±cÇ$VÝn÷¯ýëüü|ÉT©JÍãµµµúé§2Ç.d%Yø+9jÜìLµÙl Ã>þ@²¬ÀèÓÒÒò]÷J999EJÕ¼¼<z$+É CjÓ»Ý%5ëöíÛ/_NÏd%YbÖ¬Yþç.¥ªÔ©zRMzzº+=dÐ7íííÅÅÅ!!!rW577755=¬ YIVSUUåv»­VkaaáÑ£Guý©·+@²¬zEséÒ¥©éééµµµtHV@ßÜºuëÚµkÚ®¬¬t8RnÞ¼ÉÅòñÇk#%%EÕÕÕIIIyyyG9s&ÉUzrª^ö1((húôéÒ?~CC5ðdÝ´i6¥qäÈiß¹sGÚåååòÓdÆj©*i*ª¬%%%&Lïþo¾IÏd0Ykjj$85Pµ¡çïÝ»WnïÞ½«ÉM²cÒ¬Y³$M¥<ÍÈÈo~~þBBBè¬&kWWªS¦LÑdçwdÊÑ£GÍ5+ÉUòí>ºÍfKMM=sæN¯?=µ¿[·lÙ"ùvÉX#Y¥~Fhhè[o½E²cOUU¬AAAEEEÆèù$+HÖHV¿5+[±ÊãñèI5N§S¾ìeeeÆCv».É:À5«¦ì½÷HV`ì©¬¬t¹;wî»¯¼ò|ßãââäë/YRSSC/dàdÝ¿¿L´lÆãJ5-jll4¦¿÷Þ±±±òZÖ·nÝ¢£@²¬çÎÓ¿òuF~~¾´åì+8 óP³c^©Æét<yÞ+Y;::4_?ÞÙÙi,Wòõí·ß&Y±AOª±Z­Ë/7T0(É*îÝ»'9*Eª´«ªª¤ðàA¹­««#YÑî¿ÿû¿ããã§L²wï^:ôdmmmì4¦tI¦=zTÚÍÍÍº­Ø¨_IV`ù·û7³pòäÉáááÒX·nÝb²vttÔÔÔøÖ¯¨ÆÛ·okK²£^©F¾¼aaaÆJ¯¿þºL1Ý0(5+×ºÆ=ö8©SZ[[Í¼ûî»t@²èÌÌL=e®¨¨ÈwøßÈÈÈ¬¬,:ôd=ölEEÉjÛ·o·Ùln·[ÿ½víD¬|µnÝº%S>ûì3úU¾o6l0Ú³gÏÎí¦G08qdF¬ºººôôôðððâââöövcºÓéÞµk´¿üòË	&HôÒ]Àà&ë#GrrrÊËËq?.·±±±©F²^ºtdF ÇSTT$yyåÊß^zé%½¹ÜN:ñAOÖ®®.Y|å®_¿n®_ßÿó]c<&9þå_þ%22RJÕµk×ö<§¬^2¬d~5!!Á£F¬ÀHÐÜÜ!ßÍ°°0iÌ3nFV²^½zÕívgggß½Wî>|:*_Ì7êuëÖÉÝU«VÑ9ÀHVÝ±*<x 9:wî<yòDÖwÞyÇ¸É¯ÆÆÆùóç[­VùVJHH<y2]d7nÜÛÎÎÎ)S¦|ðÁ^Ý¿³náÕÞÞ.ªÝnOMM­­­dõá½÷ÞB?Y?~Ö-×©YW¯^­ÛIV`¸?>99Ybµ¤¤DOª	Z³fyÄÄDjV`¤Ô¬=ò:±U" ­­mùòåV«533³©©Éþê«¯J¸~õÕWzý¬ÀÈJÖ®®®Änzb«á]ÞMÚqqq$+0ÄnÝºµråÊôôôèèhÃ±gÏßyôDÕ'êEl86AÉjüY5«WQK²ê½÷Þz488X-ì|Öð,ßÄFñ`øU«U=uÕ8Õn·M$+0¶nÝ*_·þW2uÂ	®ô0jõÉ'!®b³Ù¤`5ÿûå_JÖ20jÕ¼·U¾½Æ)7=»¥¥¥$+0$G%M%V­Vkrr²×£µ¯ô0ÊµººZ¢ôâÅæd¼KË¬¡¶¶6))IOªFdd¤ùÑ]»vQ³£2Yå«äÈS§Neggïß¿_cöç.ímÛ¶¬ÀÀòx<R§æääèI5555òuõÕW5J¥T£¯Q¬Z¡Ö××»]>|(S$h©YARYYér¹N§4ÌÓ·nÝ*i¤£¾ôÒK¬ÀèKÖ-[¶ÈøèÑ£Ò hl dú©¹¹9;;[SÖ¶¶6¿ó­Y³æÚµkt0úµ££ã­·Þø¼÷®nï¼×âýû÷¬@ÿýîw¿ÓËÔHIúæoÒ!ÀØLVÝ,·§OÖ»Z¿Ïgåú¬@ÿÍ7O¿_aaa!!!Ò2eÝµd½sçNtt´ù"¬~kV®ÏôGQQ^¨üÊ+:133S¦¬þÆZÍêÔ¬ÀÀ:yò¤Ûí:U¾Jæ&MDc?Y©YÑÒÒoµZå;õúë¯û^TUJØ	&ÐQ5+É<_YYÃá9vìV®òUúõ¯mÌÐÔÔ¤¢¯ñX³Ö××wuu¬@o444deeÉGV)[éRÊÄÄÄDiÿò¿Ô_±æ«®kÉºÿ~ã¬^5kAAÜVWW¬@ÚÛÛ7oÞl³ÙÏ9ã;Ã/¼`ù^pp°²t06µ³³S¾ç»wïØÂdÅ¸ráÂ¤¤¤ðððµk×Wª0~kV®"¬­­­  @jÐ'¾òÊ+Û·o§Oq¬555óºåt+ÿ¶uh~i¬_:ü¯Äª¬$¤áp8è`ü&kgg§×F`Y/lÙ²èYcc£üèÔ¡ä¶°°P§çççË]>áÀøMV¿G3¬@Ï¶oßn³ÙbbbÊËË¥N>ºùQy($$^HVèU©eµZ/_®Wª¯ÌÚµkÍó¼öÚk¾ãBGÉÚÕÕ%k+Vø&kGGÉ¨öööO?ýTêÑøøøóçÏÓ¥fõð!22ï5«9M¥m·Ûõ&m¬ç¾úê«¼¼<Ë%¥êêÕ«=ùQ·ÛÍ~VdN²²5P_~ù¥¤©1¼üÐlmmõmòäÉæc£££é:d%Yo¢¬?øÁJJJÎ=+ígô»oß>w·6ÐuÉJ²ÞN§Ïmm×®]2ÅoÙd%YgÕn·Kúâ/z=$Uì_~I$kàÉÚÞÞN²bü¨­­MKKoÁÒ¥KçÌc³ÙÌÖÔÔP³$kON:­Ç'''ÇíÝ»wËÝÐÐPcÇã),,:Õívëj¾úê+ÝlÌ3uêÔú YéÉ'È|÷ýð¿áááÅÅÅæ+Õ¬RÂU*W§Ó,e+Ý¬ð#..Îb2kÖ¬+W®øÎ&ë9s²²²®]»F¿$+Éø1iÒ$IÓèèè'j¸Ò-É:ðÉ*+yóæ¬óä£.·¹¹¹--- Y+Y×¯_oòøñccIûÛo¿-u§ÓYYYiLb°_dío²VUU½ÕM>pàîm2®^ZZ*Sä)IVòw»Ý òÁ>xð WF$k6_ö|ÿþýÑÑÑÙÙÙlÆØÓÒÒ²téR«ÕZ[[«Ãüê5ÌÍ'Ø Yû»5X¯%wöìÙææfiÜ»wdÅ(uýúõ×^-22râÄñqùòåÊÊJÃa·ÛKJJô¤¢¢"=jIêÔàà`=®HÖLV]ËhûÄÒÕÉQÇãñL4iÚ´i÷î­¨¨HHHÐëÏH=ÚÔÔd³ººZbU×3fÐuÉ:`ÉúäÉ½îÀ$+##Þ¼I²bDt×®]òËÏï£?ÿùÏ£¢¢ôªÆð¿111ô¡HÖ<xpÊ)555:åèÑ£¨W¯^ö½÷usÌI²bØIJRË­·nÝògúôéÿüÏÿlþwß¾'N¤÷z²Þ¿?22R8¬««ø¼÷®¬¾øâólÔ¬!^xá_~YÓTRVîúnÅMMM5ÿèÐ!ÀÕ¬BÒ´ªªêÔ©S©GJXi477K JôJÛ¶m$+F	ÈÐÐPÝÌk°R¹^¾|Ù"f)gåskþ×årýèG?¢Q²Ê:èøñã¬ºKUÂU§K²^½zUï¿ÿ>ÉaWXX(é5QrtûöíßNªoÈÔ©S###eþµk×N6MVsúÀ &«îF"À¬ª´´Té¿ùÍoHV6	¿èèè°°0¿]»vM4É<E>º2ÿùóç÷ìÙc>©F¦ÿíßþíôéÓcbbæÌã»/±f5¶	ëY7»-PÚS¦Lá¬¯¿þZ?Æøø^×ýÅ/~aLIOOgÏí÷¤ÎdåZ7^V«5((èÛo¿Õ»k×®°üéOê5Û¡Cd¶^xA2U*T)XÃÃÃ½ÿ¬W¯^=uê±ÿõÈ#$+|Þ$MÍS$2ýâùòeùüH²FEEI²¶µµÑFh²a²réÒ¥ÐÐÐÄÄÄ³gÏ¬èe²«r8~¯<ÓÞÞ^TTd³Ùtø_ºÀÈMÖªª*Y»UWWëÝ7oêÁM]NNNÎÞ½¥±mÛ¶¬èe²ÆÅÅ§M:Õk¶óçÏÇÇÇK9k>©Fb²ê©«^Çv¾Mtt´^?§££#66V¯û6mZff&ï%¯½ö|ÞRRR¾ëÞÞ«Ãù~ýõ×Æ---ùùùV«5;;ûÊ+ô¬:nð+üÆ­Ô ZZhh¨WûIII$ëø±oß>½úùÏîw¶éÓ§[L6lØ`<´ûvÃUVVFéÉúèÑ#,Â¸ºqÙó;v¼ñÆº»téùJ®=		1ÚRy°5xÓÓiäS!ùúí·ßFGGËÝÞÍ<ªCSSü?UÊVúÀ(HVYgÝ¸qC¯ÏªwSRRô¡¹«§ºÆÄÄH÷f/¾øbGGn6É:Î/0.4ûííí7n:5>>¾ªª0jÕ7h?úè#mïß¿_î>|ø°OKX¼xñîÝ»¥!·ÏÚL²«º÷Ô ësµ¡¡!))IR¹  #âd½û¶ùdi»Ýî¾.¤ººzÚ´i!!!Ræ^¼xd%Y½úÝ·o_ÉÚÖÖVXXh³ÙÒÒÒ8R	À¨OVYßÍ7Ï|799"ÐÃë(ßÐÐP¯íÃòòrËeÿKïÝÉzöìYYÞ¹sÇ¬ÙÙÙ$+ú_¶êéª)))ÚÖ«ÓxªV«uþüùÿ`,$ëáÃe§1Ý¸qCî<ÉßÊ5¨[XXØ¾û¼ÌÀð¿ÆH²Þ½7::º¢¢Â÷Ò7Ë-cÜ`ôLtP~#ìYcccNN'Õ5+×ºAtÓî_þå_JûÇ?þ±´µÕWûæÍm6[rrò3gèL$+É:Þýã?þ£DéÆ)ÒÖA»û·µµµIII«k×®åH%c9Y;::dÍøÎ;ï¬x.=è×k¢L2eJåñxôJ5YYYÀØOV=jéêÕ«æ?&YáëÏþìÏü&kttô³þ¤²²ÒívGEEíÜ¹06µªªÊ.¸¼¼v»]æÑÍm%YaIc'«Ò]­RúÎÜÖÖ¦G*-Z´¨±±Þ0fµ³³Óh9rDÇ2¼yóf_G4$YÇ§üàzHp\Õjö¤I|g+++s:.K~¨ÑiÆx²z]ñF¯Ïº÷î&YÇ'­S¹~Uz¥	ÝÂÂB)[é.ã(YW¬X¡çJUQÞMÚ³gÏ&YÇ¡o¿ývúôé¡¡¡&LHJJ`	%%%QQQÉÉÉÀ¸KV¯©®nÆ²V%YÇ½¨ªd³Ùt¯dm/ÿ¼®®.--Ín·úé§þ0¾õìÙ³£ÕÕÕ²ö¬¯¯÷MÖÁdá¬VkPP¥ÿñÿ¡×èî¶µµåççëð¿ÀxLÖªª*-I¥A²Â ïûÌS¦OþÜñ;&¨¨¨²²2úÀxLÖû÷ï777K²Â¬^Û~_íµµ©©)++KþjéÒ¥T`¬æâÕHÖØØØëÝdbff&É:Õëråz(ßKKK¥Ngø_$«÷ÅYïÞ½+íIÖ®®®õë×ËÄmÛ¶¬ãT¨òÖ§¤¤|×ðäÉ·.¤§§¯^½j¬Ï$É:cÆmë[IÖqÈív[LòóóÍ¶··¯]»V25--­®®î@²öDV£¹¹¹Áïºþúë¯½&9s&))Én·oÜ¸+Õ Y¹×ÖÖgµZ³²²8©ÉJ²¢_JKKcbb¢¢¢:Do YIV®¡¡!##CJU)Xé$+ÉosæÌ6mZIIIÏ³µ··oÞ¼Ùf³ÅÇÇ_¸p~@²¬ð6cÆóA¿=ÿpþüy·Û-¥êêÕ«þÉJ²Â©ªiª§Êè5à|Ãµ¥¥¥  @25==j¬$+IBT¢Ô<eâÄ^SÊÊÊÝnß¾;'Õ YIVôÄoj$kSSSNNÜÍÎÎ6Ýd%YÑçuÚ´i:¥¤¤DêT§ÓYYYIG YIVôîgÕ»¦ZÅêI5ùùùTd%YÑ7&M²ü©'ºÝîªª*:ÉJ²"¿ÿýï%M'L`³Ù8R	ÉJ²¢_<Oaa¡Õj?~CCd%Y¸ÊÊJËåp8ÊÊÊè Y8ã¤¥K¶´´Ð!@²"pzRÛí>sæ½$+WWW^\ÌJ@²"p£¦6-55µ¶¶«ªªr»Ýv»½¤¤RHVhÁÑÝBBBrrrþHVèäÉ^+­n2UOª	65+¬Ä¢E$G_~ùeóI52eÖ¬Yt¬èÚÚÚ´´4½<×J21**.½bÿúÑGI²<yÒ<5+¬è-þ×|RîdýÝï~÷]÷@!!!r·¦¦¾=1ÿõ:©FÚºMØ°cÇºHVôdÏ=nÒð;Ô¬³fÍúðÃ9*HVô¤¡¡!33+ÕÉJ²ö1ü/Wªdí/þ×jµ®Zµê·¿ýígÖÚÚJ·ÉJ²öYKKËÒ¥Kõ¤7ß|Ób±wÆâÅé YIÖ>(//w8zR¯düî»ïÊÝ5kÖÐK@²¬Ï×ØØ+¥ªqRMDDÄë¯¿nÇétN<¾d²²²¨¨(¯jeºy¶÷Þ/44îd¦´´4¿'ÕL0!++Ë<%11HVÕ¿¶¶¶¢¢¢NªÉÉÉ	ÚµkÞÝºu«ðªU«è: YIVo¥ñññáááÅÅÅ^Wª1õÕW%M#º1Î>¬$«---yyyV«533óÊ+Ïÿ«¯¾ÊéVQQÁ×HVõO:¨¨¨gÿ YIÖ^ijjÊÊÊ²X,z¤ÒÖ­[¦Núê«¯rÑ7 YIÖÞ2ÿu¹J?ýéO%eccc_ýõ'y` YIV?jkk­V«ªmmm:q×®]^£)½òÊ+!!!|Êd%YIrtùòå©éééuuuæfÍe·ÛÍSnÝº%YíÚ5>è@²¬~»Í¶yófßj~ô£ÅÆÆzMdeo+¬$«RuÑ¢E:üoCCßyV®$uª1å½÷ÞdåS$+Éú'¶oßãp8¤fíyÎ'JúêøJï¾û®íøÉJ²þ?RfeeIXæåå577?wþÖÖÖØØX	T½üª×øÀuü&«Çã)**²ÛíIII.µµµeeegÎGùìÉJ²öÖùóçõ¤Õ«Wk¶··Ï?ßáp,Z´(--Íétq YIÖgjkk+((Lø4g§LÌÈÈ0N]Ý¹sgLLq@²¬~:tHòÒf³mß¾Ýë¤¨¨¨ÆÆFó)a%HVÕ¿ììlÅå ßu_ÇÆétzM,***..æ$+Éú'¤6-))túôé|Ölv»Ýë4VÉ`jV YIÖ?Q[[òb7Ãáv»«ªª|çô»U/n YIÖ?T³zõêððp§Ó)õè§~ª;VËÊÊä®o¸ú|þüy>¾@²¬tìØ1=©¦°°påÊùùùæG7oÞ,!ê÷/°gÏ3gÎpT0¬$ëµ´´äååI¦fffê~S	Q	Zó<2=>>&¬$ësH¹c·ÛÍWªuûöí^mFFM YIÖgª««ÚÔb±äää455:yò¤Óé¼råQÔ&%%IôòÑdõoãÆR§ÆÇÇmõ5ËË//,,¢VªXßk¯HVõ'Õ$''K©ZPPÐóú.ÜíÌ3|(d%Y½IJjµZSSSý YIÖÞ:vìËåò;ü/d%Yû ©©);;[JÕôôtã $ÉJ²bçÎÃétîÙ³RHV5p%77Ñd%Y'µéÚµkm6Ëå:yò$'É¸óçÏ»Ýn«Õ*áÚC©úÕW_­Y³¦¦¦O¬ÃïâÅ3gÎMJJºtéÒIVÇSPP áuÙT3ISÇb±è­Ýn¿ví9 YÓ3ôõ8p !!AO|üÝßýÝP&kyy¹ïð¿~Øl6MSIÙ	&DDDðu¤XúÃþ`ñgÎ9CÐY---¹¹¹~ÿõõÙgµ¶¶KXùÛ/¿ü¬ÃObiÙ²eÒ8á#++khjÖÔÔÔ¨¨¨ÒÒÒÞÌ¼`ÁÈÈH¯V«uÕªU|ìdf>2ñÑ£GÃ»µªªê¹¥ªaÍ5ÁÁÁæõÖ­[Ô¬@²c¯ÞpZ²dÉ½÷FÝü¬/½ô«ÜN<yÂ	|ædNR&Î=ûþýû£ñZ7AAA¯Ò¥`uÅÆÆTu×gRuÕªUYYYkÖ¬1o¬ÉÉJ²HVd¬$+d%Y Y YIVÉJ²@²@²¬d¬$+$+É YIVÉJ²@²@²¬ddd%Y$+É YIVHVHV@²¬¬¬$+d%Y$+ÉÉJ²HV@²¬¬¬$+d%Y Y YIVÉJ²HVd¬$+$+$+É YIVÉJ²@²¬d¬$+$+$+É YIVHVHV@²¬ddå½¬$+dyÞÿýI&ÍP.+..nÓéüáH?=ùÌË'~z¯¼òÊË/¿L?×Gú`i³ÙF²Þ¾ûßÚßüÍßÈZæß1äþõ_ÿÕb±¬[·®z«?ûÙÏè¡÷«_ý*""~z»víÎ?üÃ?ìb÷íÛ×ÞÞ>ºu0üÓ?ýÓ¬Y³bÈ566Êýþçè¡¶jÕ*úaèÉºØn·ÓCïÿ÷esäÈ¡y:d%YIV¬$+ÉJ²¬ YIV¬$+HVd%YIV$+Éd%Yûâ³Ï>ûÅ/~ÁÇnèûí·ñññ·nÝ¢+Þ/ùË7ÒCï÷¿ÿJJý0ôºººdóÿù$+£ÉÉÉ:Òt)&&&44411ñìÙ³|Æýû÷cccyØÅgÎ)ý$NÏúúúíçêêjz~è:uÊb±ñgü&kNNÎÞ½¥±mÛ¶òù²fq»ÝÆ§waÈÌ1CG7=pà@BB=?dd®Ç£JÿGGGÓóC¬³³S~Së!ëüñ¬ò)ïêêFGG¹Âà;wîÍ7ÍÉÊ»0ô"""èù¡wâÄ¤¤$z~­_¿~Ó¦MÆ:gÈ:ü&khh¨ß69YyXMMÍ²eËèù!."##åcàÀz~(Ý¹s'99Y¢ÔXçYçßd		1Úaaa|%YyÒÃsrr=zDÏ½S§N½øâôüPÊÎÎ>wîy3d?~U>åºY@?ñúdå]2­­­K,¹wï=?H¢çrUc6?~uñâÅ»wïÜÊy>Ã¬¼C£ªªjöìÙ÷ïß§çØ3êëëv-o=?¼ë!ëüñ¬ÕÕÕÓ¦M		=¾aIVÞ¡ëõã.]JLLjõ7ÞÐôü0®s¬ó)¬¬¬dddd$+$+û[,)))9ÝÊËË÷ïß/S¾ùææOKKëyz½è>ø@ï>zôHîÒÛÉ5§NÊî&!*iwùòe¹ÕkcSðøñãÆ·Û-ÑÛíèÑ£GæÍgLÙ°aLyðàñ'ÕÕÕ2Å¸.³¢¢þHV`¬yòä¹¬¬««ÛmtvvjJëlwîÜ»wïÞÛ7ÞxC'¯ÕªUlaa¡×	`Mq£;w®´?ÿüsioÛ¶÷ Y±@/ó)®_¿.	×ÚÚ*·õõõ'NÇãñJÖ)L5_###¥B¤Z°îÝ»W¦zÕ«êe)þðáCó2¬ÀØ¡¥ªäëÁÃÂÂ´¾Ôm¼<ðJV£Õë?ëÖc-poÞ¼ùôûÝ´æ·lÙ"%t¥­a¼uëV¢I,ÃóF$+0vÜ¿ÅpÐhSÒNReú&«Ü=wî¬nÔ]·nL¼qãÆ´iÓ.óÂ;::Þzë-Ý¬íÒ²ÞdÆãÇé~ÖmÛ¶IcÙ²e¬'NÐ9%>åQÝn¬»H¬ºøöíÛ^Á)°Ü>Ú(v¥T5wÒ· Y1E32''GÃ¯©©I#PÂRÊViÜ¹sÇ¨AuÝ/ër¹vÓc´-aÙÕÕ%%mß(kVÉº«õâÅr»cÇ#ðÖ¯_gÜõx<Æh¬û.êÞ½zØpgg§o|R³$+0¾·KAA6fÎ)FFFJp:Nccª<*w/^l¤9&gÌá÷)¨YtÃo»´Ï= õ¨LdÕÌêÓØÕªG3i=zùòe¹Ùt¯¬=75+@²cd>æè/¾)ÍÍÍO¿?£&==]ãÐ¼u÷Á:QÒÔHVI"ñÜ×µ¾¾¾THV£1á'O$«ªªûWZæJ"ÊüqqqúÇã¦Lâ¬û÷ï×e~¯µ  @àHV`ÜIHHØ»w¯qWjúäO)>ô*[ucòîÝ»)L¬¬¬¬ddd$+$+$+ Y Y Y Y@@þ/	0b]4éIEND®B`


ê@Õ¼¼¼oG^^¿ðÂú5êmWFuºsVUÙ¡¡!ÊÌ~åååV«5Þ=&¤:úæoªüã-Zd2dPÖéµ¶¶VV°5ÍÚÚÚ´UãÚS)+WÊÿÅ'%%©Ý/_¾ÌP²ÆêÂjÃ¯UÔËUyñ»gÏúúzuæ¬À,488(ÿaFã¦Mâ:P5öÃá`<²ÊÇ«¾>zttT»]éë[o½EYÙ©ªªÊn·OgO%O«¬bhhH:*TY²|ìØ1ùÚÝÝMYÙ¦§§'33S½Y3=<­²Þ¹sGþJ;µ5.]5'Oeù«¶kóWÊ</¡P¨´´Ôh4&v *gQÖÇwvvFÎ_%¨Ú7oªY,e#yùët:-ËØüÌê9+uÌròJ7//O¦ª¹¹¹ýýý@Y)+8¡Z­VÃ!sVFKe=þ|cc#ef¦¦&·Û-SÕâââ»wï2 ÀÜ³Ï>ûL[^¿~Þ8µÓ3gb¿©K.ÉKlÉ&Í¦¬@ûûsrräÿ]vvvww7Ì±²677û|¾í¼Á§O¯)))ÒT­¬ËØoSn°¦¦F>¼ûvÊÄhdd¤¢¢Âf³Ùív6ÿsµ¬cccr[ÒÎ«W¯êç¯ï¾û®þ¢v>¦X$''«§üø±ZþOyîðz½ü[@  6ÿæççs *0Ë*&,ëêÕ«õsVí¬±0LaËDHOO§¬288XPP Tíèè`@yRÖ+W®ÈëåÛ·oËÅ'N$<g]²d¶ÄÖ` êêj»ÝnµZ9O!0ÊªÞX÷îÝSÝ¸q£,<zôH&¬ï¼óöqr1zåWÔéå«,SV`B^¯Wþù|¾`0Èó§¬âÚµkòutttùòåï½÷^Ø·ãºµ;v=zTä«<ePV ÌMËÊÊ,Óéþ'ª]eøðaæ¸¼Èµ¸¸Xm+ý6ÛÛÛW¬X±dÉÃqñâEÊèIJ].Ñhôûý¨ÌÏ9ëÂlÐr¦`ÆAÏ'ÿÅ²²²ººº`Þull,m:°UþÛ'%%5å+WRV,p¡Pè«¯¾Ú»woMMMÂ7RQQaµZívuu5CÌó²j>Ù5lRKY± 466¾ðÂË-Sp_~ùåÁÁÁ¸nA¦§Ú'ªÆû³æ^YÕlUºª½*¯¬ÃÖÊèÆ&iÛ¶mÚäõÕW_ÿ21þ¸tÔï÷KÝnwkk+ã	,²>zôÏº&³wï^Í¦_óûßÿ~Ñ¢EØ)V¨j6ËÊÊ8PX@eÕ¿Û*SíÈÅªª*ÊìÍ7ßÌÊÊ[)sPékÒ¨ÚÓÓÃ0´¬íííòD $£Êúô>Z²b®ÌY_~ùåØç¬¨PÖ¿8Ø¦¹¹ùìÙ³999µµµêdL²üÅ_ÈòáÃ)+7nÈ5Æ÷YµSês *@Yÿ4CíííÕÞv½ÿ¾¬Ð2gÅ×ØØh2Ô¾Á²0á¾ÁúSês *@Yÿ¿JGO<)Ëò"5Ú¾ÁúôSV,42U=räÈÞ½¿úê«ÈíÀê@UN©PÖ¿8uëÖ­ÏÛ·o«ÍÂ999akkk)+F;PU^rJ²þE8åë¹sçÔE5ÕÏ×ç³RV,Y,ªub·nÝJNNÖësÖ¸>²bº÷î©S§úúú¦ySêú¨PÖ©g®ZY³b©®®¶Ùl[¶lÉÍÍµZ­EEE±¿¿_Rßëõr *@Yã(+sVÌ'­­­Õ@  .dddÆ;åååR ¬ÌYÿ),,(ê×ttt¸ØoAª,1VªrJ²ÎÌµ··wll²b.Ú²eKKKKØJ³ÙËÏB!	³4Õãñh³^5Ö²ÖÖÖjÉ6g-**¯ííís<zKJJôk$´n·Ê¬®®v86mß¾ì©PÖøJ8=:³SÊÙ ¯¯Ïn·×ÕÕ©ÝÝÝ.«ªª*Êtuueee©Sês *@YçÊg©µµÕétz<ÌÌL«Õ*sÐÉ®dk6¥¾§NbèÊÎÎÎÍã|ã¾¥Õ©ùe²b®ëéééèèr®ü¦¦&	°Åb)--åXPÖI7m<x9+¾¾¾£Ñèõzûûû²ÎðÖ`ÊcddäÀ6Mf«UUUì©PVÊ$®µµ5==]¦ª|¢*@Yg²¬cccRÓ=öDõñãÇ³A[[Û÷¿ÿý_|ñ¥^ÊÈÈ¸qãÆtnmppP¨êõz§&auyª,[­VµZ¦¬xîËÅKP?^SSãp8$±	ÇµªªÊ>îÈ#lþðÊÊÖ`Ì*+V¬xã7ôk^~ùå6Ä;Á`pÓ¦Mò/((`ó/ÊK&¬a3ÔÒÒR¹Æ~27-++³X,n·UPVPÖÅW¯^Õ¯ùå/Y<¨²²bÅÿøÇú56-­ÁÁ`Pí©ÃJfKYå5>eÅóÕÖÖöÂ/¼öÚk¥¥¥~¿Ù²eRÖ)÷`ª¬¬«ñªiYÏ=+¯åÕ>ÀéééÚþÀÚòÑ£Gå¢Éd¢¬x¾¤£o¼ñL^Ç¶mÛ¢°§§'33Ól6°§gZÖGqF~Ì'ÒÑÝ»w«Í¿ÒWÀ³.+uù¤ººÚétÚl66ÿ ¬À´ô÷÷oÙ²Å`02 fQYå¹ióæÍsÅÈÈÈ¾û,Çãéèè`@ÌÆ²~òÉ'ú5>¤¬)2¡lkk©Oj©©©êÃÌùDU³¢¬òÄ´uÚ¸¾¾^ÊºnÝ:íSÐ«ªªdüJÊéÛ½·L.333NgFFÆt0(((0yyyÁ`±0[ÊúèÑ#ýÇ×ÖÖ&''çää°53Îï÷<5[)))q8SYYi·Û].WSS`v5ò³äÎ?/³Y¢¬AV«5ì0¬¬¬xwâíîîÎÎÎæúæFYãÔò3gdùêÕ«3bppÐét­ikYYY·ÔÆä¶¶6À¬.ë£G¤£ÉÉÉêLZ&Óõë×)+¦Ïf³í¸´eËç¬òÈt¹ÕÊÊJN©`VõÞ½Ç[¾|ygg§ZsòäI	ê+WdyhhÈï÷ËÅ¤¤$¹&eÅtÈ3;;[Û+t8S>Ñh,((`O%³½¬ÃÃÃË-S'8ìîî|Þ¾[:úå_ê¯6::ÊÓ'sM¤ÚíöÜÜpôºrýÒÒR§¦¦¦òªæÌUHMÀÙ³g¥©¡PH¦°²000 AôÊòáÃ)+fJGGGuuukkkôýä1ér¹8PÀ,«´óôéÓª¬ê-U«Z/e½rå,¼ûî»Ï¼ªËËË36mbó/¹WVõ6ªÌ	ôeUªªªÔ¾Á²þÓO?¥¬xÚFFFÔ'ª¦¦¦r *9<gÕ¶	«£nvÛ¾»,/_¾£nðltuu©UØûÀ|(+uçE¨j63228PÀ|+ë+WÎ=«½ÿÚÜÜLYñTUWW«UËËËªeÕI´ÊRVÌ8íU9PÀ¼-k §¹öövuñúõëjç&Ê½_þò/¿ürRR|õûý	Üöª¨`>Uº¶ðÓ8Þ²Îi6l0÷îmmmÄÅÿìg?ëº»»322Ìfsqq1ª·eUçÞ³gÏ¹õù|¢­­mÑ¢E§OÖÖ|õÕW²æÆ±üøàà úDÕììlé+ã	`ÞõÁêdÚG kþùç¿þúëêÍ×K.é?É².@¥¥¥#låK/½TSS3åÏVWWÛív§Ó)+öT0ÏË*Õ¼víú|VuqíÚµê[rQê*O©Ò`ÊºýêW¿Z±bEØJ³Ù|üøñ(?Õ××çõzeªê÷ûùDU¢¬¡ýàÔrmm­ÿ>[!nÜ¸±hÑ¢#Gè[»dÉÉÞ.¹iYYÅbÉÈÈèêêb,Ä²Þ¼yS°,»Ýnö`fïÞ½/Þ¸qã¾û6lØ ¡	¯äÁcµZ+**Øü`áURºyófýÅôôtÊ½´´4Íöýï_?ÕAuJÏÇªtYÏ?/)½uë¾¬999±ªÌS].§Ô°ÐËzâÄ	é¨ÚéÚµkr±ªª*áÓ0§¤¤PÖ¥««+33Ól6±ùÀ.ëíÛ·#?úf×®]	Ü`»ÛíÖÎ(þoÿüçuÞB~¿ßh4z½ÞsÖ¶qãFufDuño¾1LdÃü[ÎMMM.Ën·WWW3(ëS¤³vDøéOÊu®SªªSê2 (ë?~,Oï¼óÎÌ÷Yçýª­­­Ê:)µ×Ò+Wô+>|ãÇÏékJYç+T@Y§øä8ítÁCyÆýÙÃÉuÁÒN©Ïª(ë¤FGGµåææfu.Ãë×¯OÿuÑN©Ïª(kx£>õèÑ£OétÁuöq·£®®®¬¬,N©²ÆgÏ=RÓÂÂÂ¤¤¤¥K6åõë×SÖù$æää¨	¨|­ªªìÚªRVN©²Æ!ìÍT×8m£®Çã¡¬óÄ255µ¨¨HÍ>Àdx9PeM°¬çÏ'ÐöövhooodYÆIù)ëóräÈM6é×Èëª°I­Ïçã@U5ÁÕT(ëB°÷îÈ¨±X,Ú²:¥¾Ûíæ@U5î²h¥¬Aé¸°ªÃá¶¶6¼rJufö`Ò5%%eõêÕWÇÉJ¯×KYçÉ§ÝnïëëÓÖäååªU9¥>Ê:3eUÎzûömYullìO>¦¬óÉ¾û$®2s­¬¬Ü´i¼úîw¿Ëª(ëS<#¿uÕªUjYb+e3W)ë;~ðÈTu÷îÝ¨²>Å²Ê<5//ï©~e¾FFFJJJÌfsvv¶T@YçÀ§ÈQÖYëÔ©Sn·Ûf³UVV²§PV$NÉ`0äææö÷÷3 @Y8õª.Kæ¬PV$N¨*YÝ·o²"qwïÞUªz½^TÊi©¬¬´ÙlêúLU².2¬««kiiÙÃI»ºº2228¥>PÖ¥°°Pæ¹¹¹^¯WfÒ×éß¦º¤¤Äb±¤§§þ·e](8àv»ÔÅ¦¦¦°Óù& ººÚápX­Ö#G°ù(ëÂ"YSÊ¶¬¬,±[$kªjµPÖDfak*++â½[,­r *PÖ=g;aïîÝ»ã³vttdffÍæââb¦ª@Yy5ñ÷Yåek4³³³»»»ù_ÿÿ«V«UÛ78öm¹ÕÕÕrN©²SÇ³Ê5Æ£N@FFLU8¥>PVÊ8I¯Úü+eíêêb@²RÖÄ©Í¿V«µ¢¢Í¿@Y)kâúúú¼^¯Á`ðù|Á`ÊJY$sSõªN§³©©ÊJY×ÒÒâv»F£ßïÙõ(ëÂ>Á``O% ¬uº***¬V«Ýnç@U ¬uZdzÉ'ªe¥¬Óü~¿Ñht»Ý­­­PVÊ¸êêjËe6ËÊÊØü²&N;PU¾öôôð°ÊJYÄª@Y)ë	¨²ÎN©²ÎÊÊJN©²N­¯¯¯¨¨hÓ¦Mùùù---WÐTåú@Y)ëZ[[e&ZRR"MÉ¨Íf«®®Ö¾«?PuÂè(+eýN§³®®N»ØÝÝmµZd¹©©U²RÖ8ôôôx<°[¶lùê«¯Ô)õ9P(+eÌP322ÂVþèG?zñÅív»~³0²RÖ©B!Å"UÛÚÚ$´2Uûí·9¥>PVÊÃñ»ßýÎï÷/Y²DB»ûv @Y)kâöîÝûÂ/,Z´Èf³½ÿþûì¬²&¨¿¿_í©Ïª@Y)ë´TTTX,·ÛxDe¥¬kjj ÍæN©²&N:o4½^/§ÔÊJY§¥ªªÊn·óª@Y)ëtõôôlÚ´É`0äåå±§PVÊ¸»wïÆÌÌÌ¶¶6y@Y)kâêêêN'¨²NW___NNÁ`¯ýýý<à²RÖÉÜT¨*³Õj@Y)kâ$¨Õ²²2TÊJY700 TÍÎÎfó/PÖYWÖ/®Y³Æd2y<K.Íò²VUUÙl6ÃQYYÉJ@YgcYW­Z¥î_ýêÕ«e¡&Âo¼áõzï8vttÈ©jaa!²Î­ÁK.ýæo^ 3Ú6<¯ÔTå<9SÖÎÎÎ]»vÍ¶­ÁU»Ý®öTbó/`Îõþýû>ïÁ³ðÖ#G°§`VÕð-uñÎ;;wîÇ| ¬ÏH X¿~ýððð<þäsevRRR:@Y9#?²RV(+²(+e²@Y)+²RVe¥¬PVÊ ¬@Y)+ÊJY²@Y ¬@Y)+²RV(+²(+e²@Y)+²RVe¥¬PVÊ ¬@Y)+ÊJY²@Y ¬@Y)+²RV(+ePVÊ ¬Êe¥¬ÊJY²@Y)+²RVe¥¬PV(+ePVÊe²RVe¥¬ÊJY ¬@Y)+²RV(+²(+ePÖY^ÖÞÞÞµk×L¦´´´öövÊ ¬Ó"Amnn¹ÉÉÉ@YgÆ3g<,làr¹¼^/ÿÊÑÑÑeËúúzÊ ¬3ãìÙ³¯¼ò[5no­7L@Y§eÕªU½½½²pñâÅõë×SVeK.¥¥¥Élõõ×_¢¬ÊÊ"²@Y ¬@Y)+ÊJY²(+e²RVe¥¬ÊJY ¬PÖ¼ûî»/½ôÒªår¹V®1×k¯½Æ8Äè¯ÿú¯N'ã£¿ù¿a¸â"Ã%Æ8Ä>_rfoÓb±Ìù²Þ¼yóßgÚ?þã?JYÿ±ù§ú§åË31úÅ/~a0½ÿþû2þõ¯Xìß¿_ëßþíßX|õÕW2ò/ÿ2³7ûÿñ###s»¬OÃ¿þë¿®[·î	bsèÐ¡ïïCå?3ã£ÎÎN®;wî0±áúÃþÀPÄâÿ÷e¸Í¯£¬²RVÊJY)+e¥¬²RVÊJY)+e¥¬²RVÊÊJY)+e¥¬5¿þõ¯·mÛÆÃ.Fµµµÿ÷Ï8ÄèÜ¹s©©©Czzzd¸B¡C¡¡!®ÞÞ^"ccc2ùÿIY(+Ê:ÛtÉápL¦´´´óçÏóPnhhÈ ÃLfxx8%%YbÃÅÃ,º/®Y³FNGZ<Àâ®gö[¸]ÏWSS#Þ¾;ÂèvîÜÉ8D×ÞÞîv»õÿcyÅ5Ì¢[µj:KmýêÕ«yÅ;ì¶pË<66&?Ö¿jÆäáøÌvX»6nÜxýúu*xÅ5Ìb·téR`ñ×3-Ü²L¦	1Ù«¿õë×Ë@¥§§_»vöJfq³uvvîÚµX¼ÃõÌ`·¬K,ÑxðÅèÎ;q1<Ìâ.f±¸ÿ¾Ïçðà°xë=ÀnY_yåÇ?ß"Ë<þbÇàØSÁÃ,±²ò0òÒvçÎCCC<À®gö[¸eÝ±cÇÑ£GeA¾Ê+Ñ­ZµêæÍêº~ýz$ÆTð0k¸xEdXy%6ì¶pËÚÞÞ¾bÅ%K8/ò(®³³ÓívL¦¬¬,Îì*xÅ5Ì¢KII	;hXõÌ`1e²@Ye²@Ye²@Y ¬²@YÌÜcaíÚµ¾qµµµ²æë¯¿rýÌÌÌè·966&Wï½÷ÔÅÈÅªª*F ¬À|söìÙqQ©ÝåËå«ú(1O>­­q»ÝÞ¼q'Olnn+lÞ¼Y[óÙgÉ÷îi?ÒÞÞ.k´OQ·ÙØØÈøo=z¤VvwwË×ÎÎNµ0::ª*(VW»uëû¶|ýõ×ÕJýVªY¬ßï[#V×æÁ7nå/¾øB>Ì¿@Yù`hhH-zUwçÎùÚÛÛæÌYBaeu821U]¶lÌPeA¦¤iiijÂZSS#k®6ëÛÔ*~ÿþým ¬Àü¡¦ªÒ×cÇ%%%©ù¥TPmã½wï^XYµ9¨úðgµõXMp¯_¿þäÛ·iõW>xð ¬èÊ²ñ¡Cd*±,8q²óÇððð=¤põõõ*2ãÚÉÌ2#Ë*/¢6ê~üñÇ²òÚµk+V¬Ø¾»,èoüñãÇ[·nUÕÏæääEZn²óÊéÓ§µ]Ôû¬]»v©²9sF]Sò)ßUÛÕ[¤Ú¬j;ðÍ7ÃÂ)3`ùzîÜ9m²+SUmw'5åå ¬À¼¢éóùTüÁ J ÄR¦­²pëÖ-mª®£Þu¹ÔBrr²ÄrllL~JÕõµ	qäUeæ	õVëÅåëç®ïO>Y¹r¥v1i?¢²ÚÐÐySCCCj·áÑÑÑÈ|2g(+°þßEEEjaÍ5²pìØ±eËI8N§víTù®±cH&W­Z5á¯`ÎPV`AP~GFFdùüùó«W¯VóQY/eUÍÙ§öV«ÚIÍG/_¾,_åjj¯LX£Ä9+@YùOí¤ßçèË/¿5O¾=¢&++KåP¿u÷Þ½j¥ÔT+«WÖHã³öööF©2@YÌÚ	=z$ELKKSþTcc£æJåú+W®ôz½ê[¡PH¾µ|ùòÈ²ÖÖÖª(ÊõÃæ¬EEEêtüXpV¯^]SS£]T§júè£´5÷ïß¶ªÉGebPV(+PV(+Ê(+Ê(+Ê(+Êe	ùO¥`P-rIEND®B`


反趋势正态 Q-Q 图


GÛ&-ªôÿÌ#G´	qqq999.]òÝJØÑ3ïò´ésçÎùÒ¢-caa¡Ù¹sgèmUWWë¢µk×Ú¯Z»Þ7ÊK,éé8qbU7ßö:11Ñ¶é:ÕºÒøøxßÖ××ß¸qCeeeîÚtö/¾½!5XÝõÎÕc7 §¡¿Bkkkèbö÷0mÚ´Ø¯ÓØØø³n:«ÛMJJêéVÚÚÚ´ü;zbq'=Y´hQï¿÷ÉÐÑ£Gu¶©©)ìó¶ ¬èmÊÛj÷Þ³~^lÞ¼9ô´!®««Ú²ªÖ.®Þ´x7¬¾ÞôM»áQï-|ìØ17çêÕ«STTº°Ò¨åÝ½öövu±kÐLßÔÖÖZ®´Îm¹~ýzZeçÎÛÏG¹÷²*ÏOwóö^ÜNo¾ù¦æh:lY5ñì³ÏÚFÌZÏ×¯_×´òï¾yófz7ßïíj®9ú1òþ.ºBËµä5k¼7ª?Ðkseu¿»ÜëÛ|ß²"<¡lNî¹^¶e×®]ËËËÓ`0ØÏk¨©©±ñî;ï¼c!ÑÈcÎ9©!Î@ËÚÖK/½d9s¦¯¬Ö<ßÓ9ö¬æû"íÚßËÍ½þúëúu´	Öï¬Û·o·µáÊaÃ,ïÈÕDêGTT=/±µ¡	³Cp¯Q¸íþuÏ]ì°ØÊfõ³¬*½w¤hO222Ôõ~D¿ývú°'Oùùùv¯.^¼hÏ-¬²¾ç¾¨çX½,0Ç(ôéÏþjWVÙV7_¬¦¬ ¬èmÝ:;;§O®iï¦-´g¶©Òbý¼BwÀjk©èyY½[UTïDJJJØA§øækªùawnoÂ½ð¦é´´4JºÒ6_áñþÔÉ'-?®¬¬TJuvÓ¦M¶paaaØòU«]ÓvéÂÃÆOÇþÕgÿþýºNÛªé°ËØq±qã<^¾|Y§öÁë.ºÝý¢©Xh¢±±Q3ÕNz6Tý"º?6ÝS¶]YmÛýohh°'IzBFYAY1Úº¹'õ7n­·gííí6fµÁY®!11Ñ»mêi£6Teõ²½Ç·´6ñ¡C(»æö©®^½ZôrÏ]4øða=zÔ·ïjj¥·èÚ;vìowº~mîÝÞ`»þmÛ¶éÔ6úmmmv¾´¡#B_YiueÏ=®ß=èdÚKÂ®|ã%KØ«×®]óýÔéÓ§õìAëÍ~Äþ~^õUËªíwÇÈäää¸+Y¿~ïeÕC`kFÈ4³ººÚ»eeEßlÀdGØ"Ë/÷µ-¶õý¼aû,bD¯úW ½×¬Ú Éöéi|ãÝÖëIÂ ®ÎId°Iè+ ¡eV55,s#Å°eµK322J=T°a¶CuÜÂê«Ý­[·Ú°ÒnB¹÷V¬XaÓv²éä;Uïí»ÆÛAFaÛA=¶K¥'1¶7U£ð3gnÙ²Å=ðÒ­ÖÖV»rïS±P~ì)ÅfÏ7ÏM¾ðá^gÕÈÕà[²²¢Ú®©7ÞMçÌ£9n;è£±÷m^ÃÈÕâê¶ÚjgtsQ5Ï¶Èö<À^ÓpÖvÛaMºHit·híihhèåO>Ý÷zªívã¼Ð²*gÏõ«ìµáÛÝÇîÛÔûöís;xu¶¦¦F[¸p¡zcÇÐ¶eíso°²¤g	zí±³W1m(ÜÕÕeû]mï·»úÝ¾SFÛz°£·Âî¿½ÝPnTT/»	Ý½¯	ýñ$''úú1ÒX¿»÷Øiß®ï30oYmÏíÖ³5²²¢o6	å6Óîíó4jq/tÀÞ`·ôîTû¦åÁ÷¾ß;8ÃþTïcV·¼¦³³³oÿ¡÷Mv°uÙ«999n@öî»ïúÖÎ&$$Ø3;tÖÚcûí0×°ùïgY9å$==Ý»Ò$¬±±Ñ»ï×½öv÷álö"ºV£íx×YM¸VÓÒÒ|#]»Ãö<ÌþrâââÒÓ§OÛòö÷/.ØË~,öVnwÀZeµ_GTeAÏÃtÿ­Ð6ø=ô ¬øÕí¢æ÷³g^½æ½Æ¾ÃqSØhèO#m±ýìgÞ³ÞÝÚý)kèaDvDböÎ´´´øÞâF¥v|SOcV<[-=½Ah@cV7ò³=ÒÖ0w,®¾O°]llaõÒ^Ð©=	ð½ÄîëJ¦½¢i¯eêÊÝ¾Ü°/µöÿ1²#Àí¨fßÔYOPìåp³Û#Â½±C¾ùñññnÌÔÏús¶û.äçç+3Ãik5¹Mä0Õ@ÊªÍºï8Xï[/]VÌ¹.zï½jïT©ªª:|øp/e;fý¸»;,«ðlï´í¾vël¾»Ûãm¿?ëªU«l¿®ýuuuvl°;`[ûÁFzåö±îÏcdËØËÏ¡5íÏÞ`Ù³g]sèÎÊÊÞØÁyyy¾ù6mröÞ³þØ[-´¡×g eíý"ÜöÑ¶ûn×¥wÝK#½<÷s3ê5ô]û>ßñ¥*«FlQrrrtj»ÌYÝ¶(«÷ý0ù´´4o´íµ]¾vì®ÛIëÊj;'´ÎÏ¹sçfggÛxÝÞÛjcÁûíåèë;|WV+´2ïÓ$(+(+z3oÞ<·ÓÌËïµÂí½¬ý¹STT¤DGGÛáN¹¹¹ÞÍúÐÕbãýÌ¿>Ëjo0õ~à¢½P×çß·±vqxþüyýö¨æû­ýUË¨ªcsôl£ÿeµÛ÷±¶óÙgµKuë6ØuqÙó¹ûfÇ÷Ú±i¡ïÝÑà¾ÃÂoÿîg0­Y³æö÷é`åÊjï»íiö4æ¾óÇÈ÷é/SÅæ;æ+ìëv`¹1eeÅx´|ùr_iMoõwV[j"Ãîå»ávûÇÚX)CßÐÙË®iöìÁû~DeU¨ìÝøøxÖÓO?mØëÒÒÒr;äÊÞ6c4äÕ½÷¾y?Óª¦¦FqïÐ³^^	¶_$ôS±ê1òÇî*«^ºOã|ÈonèOØ­eÆ2DB?¡¾§hnnÖÚ7»ýýa«aßÀ:ÿ÷Ø²£gÝt÷#v½Ø©akbb¢ï³ÔÔÐ÷Ä6Å3¤3gÎ-[ôãöõrÀí>F=¾»tõêÕ^ïÝÆe²@Y ¬²@Y ¬²@Y ¬PV@Y ¬PV@Y ¬À½áÚµköÍØÿûï±ûö°ß¼yS?þâÅhnn¾¾uëVØ/"Õ5<yÒûàÃqë£ý?å×_Ý;G[çÌnk¾wôèÑòòr-¹sçÎ®GË;wÎ]§wIß7ëlVV&ªªªôS%%%ö]ÙÖDvv¶÷jÝvÓ¥Ï?ÿ¼ÝM?~ÜikkÓÙK.¥¦¦®X±Âp¬¨¨Ð¥Þ[÷ÝÍ¬««Óé²eËzëýt×@Y¡§¬k¤¶¹Ê¤w;î?~?º*]¿]gMMMR7;[VVfµ´´è¬'N¸zõjhf¼téÁ¹«¦±±ÑÖ©BãÆM^¿~]7ãT®bcc/_¾¼¬['ùùùº8räÈ@oô¯²CÌ²««KÛßm¸srrtª³ï¿ÿ¾N5¶ýÁþÕ7¬­­U6,X`g­åmýUziw@mÔ´÷JnÞ¼«K7mÚäîªFl6áöê:ÝogûEuúÅ_èôØ±cVJoYÝzèììôÞaÔÝú ÷Øú(+0,.]ºdû95bÓf·¡¡¡÷1ëÊªM¼¶æ6òÓt/å°iË^IIÚe»71°à,YZ¢¢"ï¯ðüóÏÛîÜææfÚîP&ZY5Õtqq±wçpØ0 [ïÓ]_?eçOöûñÜ;ï¼cÇÈ|ÙmÊçws»âª	·×44K6&¶466ö^_$Ü­ûbpýúu·Ô½?	P2[[[O<ébsöìÙÐ²Úý´Ã*Ónîºxñâ n="Ñ¶~ÊÍ7º9vVû?f5¾rÔ××Cå¶1§a¥nWg×¯_ïYv´½¾èj1gÎõÏ^µj;ªººÚ-¦k>räfÚÑLçÎ[Vq/»ºÊjZ7ak#4ý¹õÑ¿~ÊVmäz»û&MÛË®w¸7Ø[­mJHh9ì-"Ó¦MÓ2·»_ìÔÓÂ;&koo_¶leùòåÐ0NÓÛ¶m³=vÿÝkÃvl°=mwweÕ0×M«UÞõÿÖû9f½ëë ¬ÀÐÓ¦Úz¦C[Ö¬¬¬ÊÊJMhøÕÓÞN/]ÙXM×`Î×¬Yc	Ñ ÍwýR*¥å'%%Å¶CËºpáÂµk×jìîÊjÏ0ôÌCmÓÏöÿÖ:fùõPV`XØ/ÕÙ¨hß¾C^VøÜ0ÎÞC¢rÌ7ÏWÚ4=wîÜ¼¼<ÓÈþýûmsçÎ>ÚéõÎ4GwCT6ìG¼ØèÓörÛ³]½hjGÿúÞucã¼ÌÌLÕtÏ=vl­~Ö$Þ¸q£·Xý¹õ~ºë ¬À0ü½z¶¶n:55Õ2£íuèÔÕÕÙv<ôsÂÒ&þÈ#Ç?yò¤Íihh°®%¶#ô¹çs±?þ|½.í]%n9|ø°»·ºfõãö÷n¥¸¸Ø^q´÷¡Zc6lØ 9º(::ZsÔZJgÝý´l×±Ú¦¾ºt»°Ýz?ÝÅõPV`õ´yÕàL#mµ»½VWXXØÏR|aäZz4kÚöæoºOæ³!£n@À¤§§k é¿ESÑª­­MIIqÑ²ë×|[L£ÒþÜÛéºvíÚÝzÿÝ­õPV(+ ¬PV(+ ¬PÖ!°gÏøøøÇìÙ³ÿõ_ÿuõOþäO-ZÔÀðøýßÿý/¾øb|õþè~ÀðøÙÏ~FY ¬@Y)+²RV(+ePVÊ ¬ÊJY²(+ePVÊe¥¬ÊJY²@Y)+²RVe¥¬ÊJY ¬@Y)+²RV(+ePVÊ ¬ÊJY²(+ePVÊÀ.kmmm0©©©¿s="##5§¥¥Å;²(«_VVVqq±&²³³Ã.£t©&JKKsrr¼5øå/IYã·¬qqq]]]èèèHHH]@±hM+«åååî¢ï¾û."¥KòÀÆiY@ØigÕªU5556©ÅÒÓÓ4§>ÄÏþsÆ¬ñ[VÕDEEù.½xñâBª¹¹9--×Y=|òÉÂcbb×¯_ßÚÚÊ:¡¬3sæÌÛ¬iß¥Û¶m÷ÝwÃþ`h)+]iié3:ÔÞÞ~áÂ§»±Z(ëÀ¬^½º¨¨H:ÍÊÊò]ª_WWçÎ&%%566Ú533²cæÎûñÇ»³ê«o(kßªªªâãã###Á`uuµ¿ÚñM¦¦¦&%%%ddd(®ÀXòí·ß&&&úfnÝºu÷îÝ¬ÊÊ'EÀµ¶¶Î1Ã÷ÂjnnîÛo¿ÍÊ¡¬cÉ%o¼ñ;áÂØØØ/¿ü5CY)+Æ×_ýÈ#<ýôÓ÷îUb5Õ«²RV¼ÖÖÖ÷Þ/77wëÖ­§NbPVÊ ¬ÊJY²(+e²òÀ(+ePVÊ ¬ÊJY²(+e²RVe¥¬ÊJY²@Y)+²RVe¥¬PVÊ ¬@Y)+²(+ePVÊ ¬ÊJY²(+e²RVe¥¬ÊJY²@Y)+²RVe¥¬PVÊ ¬@Y)+²(+ePVÊ ¬ÊJY²(+e²RVe¥¬ÊJY ¬PVÊ ¬@Y)+²(+ep¯jmmÝ»wonnîÖ­[+++Y! ¬0xW®_¸p¡âúñÁ`P§¬ÊJY`Ö¬Y³jÕªööv;ûõ×_+®ôk²RVuôÂÞ9³nÚ´5CYGoYkkkõRSS+**¼µ´´Dxô¾0e0fÍå¬fïÞ½/½ôk²Þ²feek¢   ;;ÛQiiiNNN?¦¬Ã%KRïßPÖÑUÖ¸¸¸®®.Mttt$$$x/RVËËË_øOB¤¤¤,[¶À8sæLll¬Rªë¥KÖ¬Yóøã·¶¶²f(ëè-k ;-IIIÞÐÐvá¥!|ðAÊ`:uJ5	k×®½råë²ê²FFFºé¨¨¨°Ë477§¥¥õsaöÆuYgÎÙÑÑa;x5ÝÓbÖÑþ,LYãº¬«W¯.**ÒN³²²|mÌÙûÂ@Yÿ¿ªªªøøøÈÈÈ`0X]]ý?w®û=6555)))@ ##CqíiaÊ ¬|R²RV(+²(+ePVÊe¥¬ÊJY²@Y)+²RVe¥¬ÊJY ¬@Y)+²RV(+ePVÊ ¬ÊÀ=QÖÃGDDtvvRVe²&$$Ì3'ìE]]]@YfË-ÑÑÑ7oÎò(---**ÒXvëÖ­@Y@ù?>¯³(ëuÑ¢E@Y ¬7oÞèVZZêÛ¼gÏÍohh ¬Ê:eee¡óËËËUÖ¦¦&Ê ¬wÄ	¶wã´··SVeíÛ'Vuóí~óÍ7m¨ªÓ^gPÖ~¹uëVG6QVe2*knn.ePÖÁ»zõjRRÒ#G¶lÙ¢²Ö××SVe½#ª*«§Oæý¬ÊÊwÝp÷ÊZSS³¢;6ØØôÁ5xÕ4ePÖþîûõ¡RúÎ;ï0fPÖ!;$²(+e`tµ««K5Ý¸qchYÃ~0ePÖS5kG0Ù4ePÖ;*+²@Y)+²RVe|YùæseØÛ1Àéééîx`7]TT¤³@²(ë|ù9ïº²RVÀØ+kDDÄ+(+²YYwìØásóæMÊ ¬ýRYYùl7;¸¤¤De]°`ûôÂÂBÍÑMRVeí×ÁÞï<?xð`\ÜªU«Ø ¬wº7Ø¾H®¢¢¢©©I---@YïèV±éãÇkúüùó@Y³OX³O_*++sq/^¤¬ÊÚ/×®];|øðôéÓkjjlÎÑ£GÔ³gÏjº¥¥%//Og£¢¢´$ePÖÞ´µµM6Í>Ý°®®Nù¼zõª:ºÿ~ïbYµ¿TÓÊÊÊ'N¨©­­­Âj¢©©IAUz5]PP@Yu.;vÌÊj/©*®6_e=ö¬&6oÞLYµoö2ªªÞ²ÂÂB;6XówîÜIYu`ûí]7«»eggkzúôé¼ë@Yù®ÆPYkkkÁ` HMM­¨¨ð^T]]=oÞ<]¦Åì=÷VYóóóßzë-Mðw	ÕïìÙ³'Np5îz²²²5QPPí½())ÉîzIIIrr²&JKKsrr¼ËþÂ/¼ðÂ(,ëÊ+µ&v³½èüieíñÓ5á*;Pqqqö)ÿ			=-­SeÕðï¾û."¥KªÇàW^Ñ½zùåíìºuëtVãWþ:²þï×É©UUUvöâÅvÌð ®*öª©©Ù°ab333µXzzzCCæü:ÄÓO?=ÚÆ¬zZðäOzç$&&ÞÿýüueýckÍ ßÆé¦£¢¢B¸~ýzVVÖ7¼3ÓÒÒî×Y'NxèÐ!ï_|QÏøëÊú?Ç¿qãÆ°¹Uz3gÎìèè°½Áö]ªæääýº°e<y²ÖwÎc=Ë_'÷²jàhÁôl·¬ïîÛ·oÑ¢Eö2gmm­÷Ò·zõê¢¢"MèÔæÊÊÊÌÌL5ÛLScc£WÝ+e]¼xñ	ÊÊÊì¬Æ¯ZK¶ò×	ã½¬ê½ºiáÔÙùóçÛEÊÎÚ'HAßÎÛ^TUUÅÇÇGFFê§ª««Ýé4!!Á÷@ ¡¸ÞCïºIMMÕ¯ÝM,àO(kÐ¾þúë6ðàA½~ý:ÑO?ýÔønð ¬ÿ«±±ÑûfMk4Ég0(ëà¬+V¬ðMOO§¬Ê:JéåË½e]µjePÖ;rä:j1544èlaaá|eÓ²^½z5..®¬¬,ôåì3(+²ò-r²vtthÀúüóÏSVeöëÙ³g½3oÞ¼IYµ¿ßrã>Ú°´´TYÕ÷ùZrÊ ¬¿£³³ÓMÛç.]¼xq4|úeÜeõ:¿Ñ(ùhCÊ¸WËºqãFÕ477·´´4***::º´¦úÊ ¬áù^LÛÍSOßINY5üGª£UUUè¹sçBËzw?@²î¥²VVVÚT@Yï¨¬mmmMMM.±õ®¸råÊÛo¿ûÆoüË¿üâpoY½WWÖäääóÝ4sÙ²euÔÕÕÁ5kÖüíßþm^^^llìÇÌ_9²ÚÉ]½zUÓÚÖ«¬]]];vìÐÌÊ:LÒÒÒ4`ugO:5kÖ,bõÕW?üpttôÔ©Sì±ææfþ`´ÕKeMJJ²iûv9Ê:.ðÈ#´··g.[¶Ì7lýôÓO'L³nÝº+WFFFN<¸À½TVS×¬YÃg0·3gÎ,Ð7SkþÐ¡CÞ9Ó¦M»ÿþûÝY5UqÕÈ¸gÊÊ·Èo¿ýV#Ñ/¿üÒ;gÖ¬Y¾ãôD§¦¦Æ;G#×èèhþ`ôUÛîµk×º³õõõSYYIYÛ_ýÕ_=òÈ#|òÉo»fZ¶lß2z,¾ùæïuëÖM2¥emnn¶Q7´wñËmÆÛ»n:¦RÎ;7//Ï÷²«äädï©S§&$$ðÏ£®¬[·n]³fÀaIIù¹6Üî+ä¼ÃYÊ:òÞzë-=Ø¾LLÌÄ?ýôSþ`Ôµ««KìÃ»¡ª®Z§Ï?ÿ¼wüúÜsÏQÖ»«¬¬LO&và¾úê+þ`4ÕÂ©qª>zô¨W«ùÎ;ïPVeLYçÌÓÖÖ¦[·n¹ùùùõpàÀ<úè£/¾ø"kîZYÖ¬Yc£UÚÌøøx·À»ï¾KYG¿ÅëÁMHH0aÂ)S|GîÂÕ"zéÒ%MWWWÛÌ;vPÖQ.??_Ô¯~õ+;«¦Y³f±f`TU:;;ÝÌ=öPÖQ.99Ù÷>häÊÑRVï»ví¢¬£\|3õØ±fà.Õû:kyyyèÛr86øNpáÌ3ß~ûí°ÞÊ£>êû¼ÃuëÖ1f.kèûY÷íÛg»ããã7nÜèePSÓÒÒ322bcc·nÝúùJC¥¦¦FÕmÙ[]_|ñE]ºt)ÿ0wÝý÷ßñ½V0ÆËªê¿ýüùóvöæÍÞK÷ïßïÝWLYDMOOwVxéÒ%ã7ï:4iÒ$·_¹r%ÿ-wÝäÉõX<óÌ3Ö©¦õ±Z±¾ëføj¨êóå_Î1cø­æo¾ñ1î]»v)¥~ø¡£iÍÑó-V@Y)ëíÞ½ûµ×^óÍLLLÔà?âq"---ô¥nÍIJJbåc¿¬zýê«¯zwßÝwÚ²:thÕªUÞ9W®1+Fùóç-kJJ+ãeµ3Ü¹s§söìYß÷ÊQÖjjjRG?øà;ÛÚÚÕ¿àñã³Ï>ÓÿÑO<áæè/YsöîÝ;|7ÚÜÜü«_ýê©§Ú¾;p×Êjo¼ÑDdddff¦½gÑ¢E999öVªª*Ê:gÎ	.Ô*Õ²ª¾ò<Þ­úÒÖ¬Y³tªé´´´á»¹üü|uCÓ¦MÓD èéÛ¿úê+øÐ¡C<FÀÐÕÞxsðàAKì³Ï>Ú]>7xÐÒO>ùDÛ¯ºº:þvÇ§]»vÅÄÄ¨vS§Níåàp=»<yò÷Ý÷òË/îKÕô±Çs×þðºÎÐ%­÷Z~âÄàaeÊ²>|Ø½ªjïÖÐ8uÍ÷¼ÓOYá`ÓíR§3fâzzê)_G¿ùæ]Û?þã?zg&''«¦eeevöáÖ*Ã< ¬CöIÙÙÙöçVÖ­[·ú¹»_!GY1æÙ^b÷^©eËé¬Nz=©©©úfN4éW^ñÎ±ïô-£*ó@²MYõ?ÖÐÐ Óââb;«òòòr7lÕDQQæ_»v²ÃAÿ_/¼ð¯µø@	Õ1ô>fU¿C?MZ=ÖÈuÊÚÜÜ¬ÿ(ï+©¶Kª²²R§vøÒÂO8¡³/^¤¬*kûoB÷~(«ï]Î÷Ýwß >ó¹¯³zÇÇ&&&fñâÅ<  ¬C|lpemll¤¬cÃ«¯¾ª­íÄu:èe nid]VV¦éèßÄ·ßµÿe=¶oNTTTèO>ù¤*¨Õsâ°´zl°ïEVÑEº÷ÂªþtsîeW²YYõ_ÚÐÐ°yófýÓ¾ùæ¿eËýjCÌÞà1ã'?ùMmsmT4¸×ó ä)`ÞWÍÄU=ðÀz RSSm¬êìßýÝßùB>eÊ]ÿÊ+×­[§ÿS=7û-©À?õÔSC=.IC[°õã			së¶V¯^ÍÊ:,eÕi]]N;ÆuL²GÙ;<1cFè®Hô3n¿«KÚ ¿×GOg¨»ÊªÑ¸ó?üá ï¿j¬_Ñ*0¼e½|ù²NKKK)ë°úf>þÐÏF¾'.wrûöíëéÛ¦Nêûò¢íÛ·kÜÉÞ²¦¤¤´¶¶Ú´Ê:úô¦¦¦¢¢¢¼¼<Í¼uëeÃ¬°eý?øVÏOúSï 2ôËìp½ûcgÍ¥þÓýÑ5û>3??²£º¬îSì½nî¢÷Êz¯ÓØTæ'|âæìÜ¹Ó7.«QQQôQaa¡½ ½÷nßb÷ß¿.Z¼xñºuëbbb4z¸ÐPÑø8x_7»½Á·¬æý÷ß÷¿û<a>)bÌ°HØËxvlÚàºÛBÍµÃ¾BTVÕÔ)S¦$$$ôô	½CÂEÖmß¾ýÀ<ðÀ° ¬CSÖ7ojóñüóÏÛÙ7nèìþýûí"Ê:6tiâÄn'§¦9|)lY/àóàÞák¨wîo¾ILLT_õ¨)ç|>0zËÚÙÙ9¿ÛÑ£GÁàv³cñ5Q\¬iÝ*eKvîÜIS)«oßï÷Ýw×Ëà^³vuuyC«-Èûï¿Ô ¬ù²z;ªñ«ÎFGG·õÐÜÜÿÖ[oÝùçõ_¹råóÏ?÷í	ÆrYÛÚÚuÓuÇÚ¬]»Ö>4Ø¬Û¶m£¬?-®QQQ&M²éñV+WÚëöòÁ ?²¿½½=77wÆöuÅMMMüaìÕvG--_¾Üw©þ7(+ÆuÔ÷Y³f·_?++K÷á¯¼òÎúÞPÛOÊê%K4fuÕYþÀ0ÆËzúôéìnîÛX5xõ~?ëÁíRÊ'O~òÉ'½stÖ÷E:ý¡ j´jYuCØ¹sç~þùç¬då²ºÑªXçÏ¡¬À8q¢ïØcÄ×ï9s&##Ã7SOÙ?øàV2ÆrYÆÆFµºº²b<¨¯¯omme=ô4fõ½°ºråÊAYÁ o=?þøã§Nb%cìÕÞ½züøq÷çÞ#>LY1f,_¾²I&iöÐCy÷UÂdeeiå8pÀ;`ÜAL³æææ¶··ÛÙ½÷Î;u±_VËjQQQW7Í¹ëßvNY1LôÑ)S¦;vLÓçÏ=öïýÞï±ZÂ®(¯h"99yp×£.[¶L5ÕuV5ñõ×_³z1ÆËÚÚÚªß5úÊÚÙÙIY1(¥yÑv?22ÒÎàõé§fu»óo;sæÌ|pêÔ©o¿ý1^Ö[·n© ºÆ+V¸#·mÛ¦ùë×¯wK?¯³¶¶6ÔÔÔÞ/êeaÊ!W\<mÚ4ßL¢ô7ÆÊ(ë¿ÎªQ©÷îáÚwã(Õ½_:çW!ÒÓÓµí¸c¹¹¹'Nüó?ÿsïÌÉ'ÿñÿ1+gp~þóë?4))iáÂ7ndàõ£ýhX*qqqiz¿(tÎOBÌ1cúôéÀP4i­îl00aÂã?Îz(**JYMKKðÁõEÿ°¬Ü´)Õea§Ã^ÔËÂìÆpøÍo~£ÄÆÆêiöìÙÊjqq1«ep/êYï_~éæ¼ýöÛ<òkìzÞOkÒ&¬÷zY²b´¶¶æåå-]ºtýúõ|Ð ½÷Þ/½ôwN»ZË1J ¬CoæÌö¹N:Õtïõ²0eFyY7mÚä9kÖ,ÊÊ:ôV¯^]TT¤	feeõ~Q/SV`4;sæ:êýâC±7uXTUUÅÇÇGFFA÷Yö¦ÐÂ.LYÂK/½òñÇýõ×o¿ývll,[Êzo ¬À¨µ÷îõ5++­AY)+ÊJY²(+e²RVe¥¬ÊJY ¬@Y)+²RVe¥¬PVÊ ¬@Y)+²(+ePVÊeúÖÚÚzþüyÖÊJYG÷Þ/..î§?ýé=tÔøøø	&L4)22ò¿ü%#ÊJYGÅ)Âã¡º'îöôéÓuWmÀZ\¬¾W²Þí©¦IIIÞÊ¾ðÂ£ünÿå_þå)SZ[[ÝÅU¿(ÊJYï2utêÔ©¾9êë(¿ÛÏ<óÌO<á©ak=)ÊJYïrYslØ:ÊïöúõëçÎësåÊÝsò ¬õ.Õí¾Æ¬þ¹îäÜa§OÎ²RÖ»_Vùû¿ÿ;¯³:ý÷*«ºvíÚ`08yòdåe¥¬wY]]]Äï8qâ½rç5f]¼xqjjê/~ñÞÕ²RÖQD¾	ÝÒÒÒX(+e²RVe¥¬ÊJY ¬PVÊ ¬@Y)+²(+ePVÊ1áÃ?LKKKIIÙ»w/ke¥¬¸#Ó¦M³/³³/ÕYÖ	ÊJY1H³fÍRMóòòììo¼¡³÷ß?ke¥¬SçÌã¤¸²fPVÊÁPD5NõÎ±a+ke¥¬äuÖ¬YÞ9:«¬²)öÙüùóSRR|Ê!§?P/^ì=ûÄOð ¬uì3g÷`Ý)S¦ëÍÙ«ÎìÙ³yPVÊ:vüâ¿PÞÜã²wï^õdäìÞ½;==½°°°÷ëüðÃÿíßþ­téÚnõõõ<(+eS"##cbbBÇ¾Å^íµßõÑG^½WÕhüÿð¬a²/êïeÎÐu/`¥ÔÎjÌjíÕgyFÓjêÄu¶÷Á+PVÊ:Ç¬S§NõÎ=¶¯qqq¾9×Ý»wgº¬zçô´c(+eìuÖùóçÛÙµk×êlRRwI&¾1FýøÇ?vg5HÅFEEù²²/ö¾ªªoûî»/ìþá?ýÓ?õµÖ÷ÂªzYÃ(+eòòò4~ýì³ÏB/Ú½·ªùüÀeÕÞc/»:'NÔ|÷ÂªÆÁZf×®]¬[²ÂïÿðÇ¿öÚk¾eTe»(**ÊÒÆª@Y)+ÂÓõÇ?þñ÷Ýî­:°ª¦S§NVPVÊ ¬Êe¥¬ÊJY²06ËZ[[@jjjEE÷¢êêêyóæé¢´´4-¦9---Þ7;RVeõËÊÊ*..ÖDAAAvv¶÷¢¤¤$»ë%%%ÉÉÉ(--ÍÉÉaÌ ¬=ëêêÒDGGGBBBOEGGëTY-//w3¿ûî»È&LXºt)<`5öª©©Ù°ab333µXzzzCCæüßÊê²eËxàã´¬eºé¨¨¨Ð®_¿uãÆïÌæææ´´4ö(k÷mA9sfGGíÖ´oI4''§¥¥%ôJÂf²ÆûuõêÕEEEÐ©Æ¦Þ*++333ÛÚÚ¼Ç4566ZquePV¿ªªªøøøÈÈÈ`0X]]íµ:MHHð½Ç¦¦¦&%%%ddd(®@Yù¤e¥¬PV(+ePVÊ ¬ÊJY²(+e²RVe¥¬ÊJY²@Y)+²RVe¥¬PVÊ ¬@Y)+ÊJY²(+e²RVe¥¬ÊJY ¬@Y)+²RVe¥¬PVÊ ¬@Y)+²(+ePVÊe¥¬ÊJY²(+e²RVe¥¬ÊJY ¬@Y)+²RVe¥¬PVÊ ¬@Y)+²(+ePVÊe¥¬ÊJYuTÚ¼yó~ð¤¡óðÃ'&&&a¤üèG?b³ÂYáÍ+<&&f|µ±±ñÿ©_~9""âoþæoþFÄýÙiïÛ·U12ôdT+|ïÞ½¬§þ×ý×¬ùùùCx~øaû8*ëûäOô¨ü÷ÿ7«bdüÓ?ýVøý×±*FÆ©S§´Âÿó?ÿU12~ýë_kÿÇü«bd|úé§Záÿþïÿ>·EY)+ee¥¬²RVÊÊJYAY)+(+ee¥¬ ¬²²RVÊ:<>ÿüóGyäÖ­[¬ñÏÿüÏZá7oÞdU3gÎhóTfÄüæ7¿Ñç©Ì©©©ÑÿíoKY¸ÇPV(+õÞU[[@jjjEE+dø´µµ%$$°æGFuuõ¼yó´zÓÒÒ´ªYáÃíÜ¹sóçÏ·Õ[UUÅ'NáMeí[VVVqq±&²³³Y!ÃDÛ÷?ÀnIIIö§%%%ÉÉÉ¬ðá¦­yyy¹&´ÚãââXá# ³³SOÝVeÄV8eíþººº4ÑÑÑáQah-_¾üâÅÞ²²æGLtt4+|Ä?~<-->vìØ±gÏ·U±NYûÂNcXþ"=eeÍ6°ÂGf5mÚ4ý°ÂÛåËÓÓÓR·U±NYûé¦£¢¢X!#VVÖü¸~ýzVVÖ7Xá#æÄ3gÎd·U«V>Ú»U±NYû¦ÿÛ`ÿ²²æ[sssNNNKK+|Ù>Ü¯µo«W¯.**ÒNõì2beeÍ«ÊÊÊÌÌÌ¶¶6VøÈHJJ:wîÜíî£²µæYá#¿U±NYûVUUõ/Á±²²æUBBï=+|XÕÖÖ¦¦¦j´ºhÑ"ÛOÀá­Ê­pÊe²@Ye²@Ye²@Y ¬²@YóÿvDÄüùó³º<xPs¾øâ^_¸paï×i_"½eË;ãÆ-,,dmkN8±ª"ªÚÕ××ëÔ¾0Ë[ÁcÇ¹9)))JïnG-//×+V¬psvíÚ¥9×®]s?RUU¥9îKBì:ËÊÊXÿeÆ[·nyuuu:­©©±ÎÎN« l]¾|Yg¯^½ªÓEÙLïwµÚ(6//Ï7G¶»qðòåË5ýþûïkº  Ç ¬ÀX`ß÷)çÏWáuzîÜ¹ãÇk¢µµÕWÖ`0¨©õuÚ´i¡jBCÒÔÔT°kÎÙ³g£^]§«øõë×½×	²cUÕ×ÃGEEÙøR´¼×®]óÕAíK¡mï±p/^¼xûûi½¿óÎ;©èjÚb¯9VbM9r ¬ÀØÑÖÖ¶qãF®¤¤ÄÒ¨§j§	2CËª³§ONHH°ºÛ·o×ÌøøøììlMx¯¼££ãÙgµÈö³«V­òEZ×Ã£PV`L9vì;¤È^g-((ÐÄ¬¬Ç·%O]jûí%R÷"«ínllôS#`<yÒv5Tu;Ù ¬ÀbÌÊÊ²ø]ºtÉ¨XjØªË/»1¨-c¯ËÎ;÷h7;FÉ&âââË®®.ý¦my7 ³ÚE(+0FØK­ÕÕÕ:Ý·oÞ;æÌãÎ¶¶¶º±¬^UKK6ÜÙÙOÆ¬eÆÁ?sDÄ¦MlbÞ¼y8|øð´iÓÎÄÄD·UêìêÕ«] ½LJJYÊ¶ã·½½]ÓÉÉÉ6Õ|Õ§Ñ§©Õf²ñh½NµíòÕµx3f(+0öÙÑIÞcöïß¯9MMM·¿GMFFåÐ»w÷Úµk6S5ueUq5GyèõÜ¹s½T ¬îîoÝº¥"¦¦¦VVVöùSeee6ÌUµü9s-[fµ¶¶ê¢éÓ§õàÁöZÞ7fÝ´iÜ(+q'99¹¸¸ØµjÚ¶msýúuß°Õv&10(+Ê(+ÊeÊeÊe²Êe²¾ü?nsÀqIEND®B`


);;ûÊ+r¶¿¿²(k¤#×iË:88HY5Ò²vvvöõõíØ±#..nÏ=2ÇåríÜ¹sñâÅô[ñÉ/¸yóæ·ß~+¬uú²Ê©<oÊiKKcVØ;w.999kL466²NPÖéËz÷î]9'MÊ«öööóõ±.]¸Êë0ÖÊ:eY322ü~¿NKY,YR]]=44T[[[YY)3>JY_['O´ÎÙ½7ßy²N¿N[?ÀÌgr(ëk+33óöíÛÖ9/_ÎËËcÍ ¬á8qÂv~s<añ[¿~½íÕ#G°fPÖp<y")Ýºu«üø±=uê^DY_g'OLKK»sçñ«Ûíf'&5´ñññ.^¼(OÛ¶m»8I²ZTT$uuu2-·JY_g/|ìØ1Ö	ÊîèÁÖÐJGO8ñlÎ£¬/ÙÐÐPã¤ÖÊ:Í7¯$#Ôýû÷KYKJJô Á:`Ý»w/ePÖf¯¥üü|Û¥ccc@Y#ríÚµÂIæÛXeðjý~Ö3gÎèwàPVeÁhU¬+W®|ö ¬¹XVcppPÊÚÑÑAYõyËª^mmm5ßynÝ©¡¡²(ëÌ²Z[[;1IæÌÙo;§¬¹^V¿ß/µ]£­¬ããã@Y§÷ôéS)h kÜ°aÙCxïÞ½2¿¬¬Ìì0,f=]]]n·Ûétz<¶¶¶àOÙ¦¤¤PVÀ+6f5£Rë^¯×«ß¢S]]-Q·^äóù222¬9ÿ¿A²²²øºÀ-ë"11Qs.fÛð4??¿¿¿ßZÖ¿@YõÏNgÈiëçkÙ ¬²×)66²(ëÅü@¦ôPr*Ó@YKQQQmm­LÈ©×ë¥¬Êú>Ëår8n·ÛUÑZSÊ ¬)@Y)+²RVÊ ¬@Y)+²RV(+ePVÊ ¬ÊJY²(+ePVÊe¥¬ÊJY²@Y)+^5÷îÝ;wîßïgm²Ï¥¬¬lÁo½õÖo¾ép8<È:(+e¢tìØ1ÉêW_¥gwíÚ%geðÊ(+e¢áv»KJJ¬srrr<k ¬³OnùùùM6uwwÏËßñÍ7ßüúë¯­s<À_ ¬³ì·¿ýíV¯^-Cºüä'N§óúõëóï×|çwvíÚeóÿøééé<ÊJY1$¢.ü·û73gÝºuK,¿iYYõEÓÙ	 ¬/¤7¶qÛ½÷d+§óïÿý÷åWs»Ý.K&äñÉ ¬³lÓ¦M«W¯¶Í|ã7æë»­2f­¬¬Üµk×|ýÊJYñ#ûì³Ïbcc­M¨««s8¬²"Jñññ?ýéOÿ0ùîão¾ùá²ZPVÊ(ýþ÷¿².X°à7ÞÓuëÖ±NPVÊYÐÝÝÍÑtPVÊ×Ñõë×Ï=+§ccc¬²RV zwîÜÉÎÎÎÈÈ(..Î$sX-e¥¬@4dºlÙ²ÊÊJ3TÝ·o_ZZÚX9e¥¬À]¿~]©¶-À2Çv@`²9wî±m¦Ì9ú4+ ¬±o¿ý6--Í:fi3/¿Be¥¬xòòò¶lÙ¢o¬úýþ²²²µk×²0@Y)+æK.8pàäÉ·oß~	7'M-((HHHÈÉÉINN^¿~ýÐÐ²RVÌ9É[fffUUUIIÉo¾yáÂÈ¼¥¥åÃ?üû¿ÿû(¾ÊMjÚÞÞ>00À_ ¬óGqqñ-[ÌØ¯¿þ:>>>ÂÚåçç/X°àç?ÿùêÕ«N§Ëåß[²RVÌ@rr²-Úi°²²ÒápÈUÏÊ$$$Ø¾&(+e½HÝn·mæîÝ»÷íÛ7íÏJDm_§#å«ëPVÊúºæÍ7­srrrÎ=;íºÏ>ûÌ6sÁ¬R²¾Ö8`=l¯]¶lY$ß³zõjÇc³k×®·ß~U²RÖ×ÝîÝ»-Z&Yýî»ï"ù©îîîæççk<(ÖO>ùõ	²RVüñ³7×¯_¦ÎèpMMMñññÒWÇ¤ÊÊJÖ$ÊJYñ¼îÝ»×ÛÛËz@Y)+²RVeýÑuuu¹Ýn§ÓéñxÚÚÚ¬utt¬X±B.ÊÌÌÅ(+²NÏëõÖÕÕÉDuuuaa¡õ¢ôôtý%ëëë/_.ÿ/È¯ýkÊ ¬811!@ %%eªÅâââ¾ÿþûPÖ­[Ç@YÿÄét¶êììÜ¶mL|äW¿úcVeý3Ãa¦cccxôè×ëüø1ï³Ú<yrË-»wïæ[àµ.«Ù+ÓIII@@·Ë´mÉáááÒÒÒö¶)//_¶lYMMÍ¥K¤¯K.åD1ëÕÖÖÊÊØÔzQnnîèè(º±¹|ù²¤thhÈÌ©¨¨µÇ?PÖg>Ïår9·ÛÝÑÑaµrbÝS²UUU´ÎÑïå(+GFeeå#G¬sÆÆÆâããùÇÊJY£qöìÙÌÌLëáé¿üòË¼¼<þ1²RÖhHS¥£k×®ýúë¯¿ûî»Ý»w»Ýî7nð²FÉï÷KP³³³eðºeË¿µ@Y)+²RVe¥¬ÊJY)+²RVe¥¬ÊJY ¬@Y)+²RV(+ePVÊ ¬@Y)+²(+ePVÊe¥¬ÊJY²(+e¥¬ÊJY²(+e²RVe¥¬ÊJY ¬@Y)+²RVe¥¬PVÊ ¬@Y)+²(+ePVÊ ¬²(+ePVÊ ¬ÊJY²(+e`¾µ««Ëív;NÇÓÖÖf½¨§§gåÊzÏç£¬Ê:=¯×[WW'ÕÕÕÖ$¨ÍÍÍ2!¿jbb"ePÖéI2'&&d"¤¤¤µµ533S&¤¥¥åååñPÖ?q:!§ÕøøøâÅcbbêëë)+²NÏápéØØØËr%))­ÁÊZÌdZtkðTù9¥¬ÊBQQQmm­LÈ©×ëµ^ÞÓÓ#¹¹¹@Y§çóùÃáp»ÝRP3¨Õäx<­®Y³fdd²(+GPVÊ ¬ÊJY²(+e²RVe¥¬ÊJY ¬@Y)+²RVe¥¬PÖ¨ìØ±ãí·ßN=ÿgR:¦Ê*b=ÿ*JKKc=°æÔ*Z´heý£ÁÁÁU?ÿùÏÿöoÿöß1µ¿û»¿c=ñù¹¹¹¬0~úÓæçç³Âp»Ý6mb=ñÎ;ï|ðÁ³x¿ûÝïÆÆÆ(ëìÛ¼yóÖ­[Yaüò¿üçþgÖC999¬0~ñ_ìÚµõÇãÙ»w/ë!¿ø¿8xðàK¸!ÊJY)+e¥¬²RVÊJY)+ee¥¬²²RVÊJYAY)+e¥¬²RÖë_ÿõ_«ªªXaüÓ?ýÓ=Xa8põÆ?üÃ?|ùå¬06nÜxìØ1ÖCyyy555We²@Yç®®.·Ûít:=O[[+$ØÈÈH+Äjtt4%%Sä«MGGÇ+ä1)E¬¢ó(âÑ=¯×[WW'ÕÕÕ¬`¥¥¥¬`>/##ÃúÍÃiÚUÄÃÉ&==]Z[__¿|ùrE¬¢ó(¢¬ÑKLL@ `eCÁÍÍÍ¬`ùùùýýýÖlðpvñp#..GQ$«èå<(kôNgÈiX_0æææÊÊÉÊÊêëëcØÿý,Ùàá4í*âá4ÎÎÎmÛ¶ñ(d½GeÃá0Ó±±±¬0333Ya²ÁÃiÚUÄÃ)¤Gy½ÞÇó(d½Ge^RRR x6¹áE¦Y!áñ><"/+'kJKKGFFxE¸^Î£²F¯¨¨¨¶¶V&äT^±B¥§§ê;77&<¦]E<lÚÛÛe=ò(|½GeÏçs¹Ãívwtt°Buvvfdd8Îy³BÂdÓ´«MJJí$<¦]E/çQDY ¬PV(+ ¬PV(+ ¬PV(+PV(+ø³råJï¤ÆÆÆ3gÎÈo¾ù&ÌòÙÙÙá¯sbbBÛ¹s§üø±­©©amo®R0I"*µëîîSýr1k[ZZÌIoñ¤/677Ë6l0s¾øâóðáCó#>Oæ/TÑëljjbýo>jVÞ¼ySN;;;ub||(ÖÅîÞ½+gïß¿/§kÖ¬ÑÖ¯´ÔQlee¥mX+nÆÁùùù2âÄ	®®®æoÊ`>ÑÞÞ^)Üðð°öôô´¶¶Êßï·ÕívËÀTûºxñb¡ÊI=XëêêdÎ­[·l£^¹NSñGY¯ ¬æªJ_bccu|)Ôm¼>´ÕAõ«¡uë±pûûûýð6­uáÃËL®Lk=*s´Ä2qþüyþ ¬æÑÑÑíÛ·Káêëë52âÚÉ2Ë*g¯]»¢u?ýôSÙ××çr¹eÂzå@`óæÍºY¶  Ài¹þ ¬æ³K¾ÏZ]]-Û¶mÓ²¶¶¶êO¹T·ë[¤æMVÝ<88h§åôêÕ«f°+CU³»yù²W´^¯Wã700 	XÊ°U&îÞ½kÆ º¾/vqî£¤Ë	ù)ÖåÍ8xÌªÀ<¡oµvttÈéñãÇMðöïßjÎúý~ó#ÕÆÆÆà«ÑÝÇÇÇóÉ ¬ÀkðSQQ¡+V¬ÅK8.]j1o Ê¥r¶¨¨ÈÒÉôôô7Á ¬ÀkA7üÉt[[ÛòåËu<*ó¥¬Ú<·Zuo&vwwË©,¦|eÀ&ÞYÊÌºwu£S§NÉ¡¡¡g?|¢&''GshÝºûðáC)55eâÊÉóLÇ¬===aªPV¯sÂ§OJ=Oû´?ÕÔÔ¤Ã¢,§ùý~¹hÉ%Áe=sæ@Q·Y+**ôpüE@Y¼v/_^WWgÎê¡öîÝkæ<zôÈ6lÕÉµµµLÊe²@Ye²@Ye²@Ye²@Y ¬²@Y ¬ ÿî¿b¹.IEND®B`


Marshall评分


正态 Q-Q 图


wõ DfÖU÷D­!ùt¹KÑ´>×Ñ±à«öûc¿èn½ÐÊªÖ#cM²²"¬ËªÃD3±~illLú¡ï@=vì¾ßQßKÜ9)ÜñãÇ·nÝªË[,su·2ëèFÌÞ1ó)ëÝ»wõÅ?³F_|mW`qØÓÓ pË755ÉóÍ°¬×ªÌVZhïÒ¼õ¾Á/ÙêU­Ö/1kAAÁËïé G§2emllã§ÓCLÌ-´­°éäÉl.Ý¿Lïö«Êj¶°¬/++£¬ ¬ÀÒÒÁ®¾wîfãÊ+æ-=/göÄ9xð |Kbbâ|9ç=ztß¥·PéMéÛfywîÜüë³mª­­Í¼åIï<ã ZßàãU-ÖÖ³îßTVzi^îxÊpD~=º¡õM>ó?$@Y7 ÃëhÕ<4Ïz ááá¨¨¨¡²ÏúÖÕ5	atÛ¶m;~üø»¾|;Ç·¸õ¬wuÖ¯ê¬ç¾tÊeÊeÊe²Êe²Êe²îÙ³gúÚ¿ÿw²Ù¾úê«Y/üüùsùjOOO¿,/ä¦'''gýS¹æ»wïZ?K|)n=ü·@YÅøS¶Ù>ÿüsëytÞ5£àuuuµµµrÉÓ§O¿êzä2ÝÝÝæ:­³gÎ±õx<²ÐÒÒ"ßUYY©²-Ë²°oß>ëÕFÏ¨!_Ý¿¿ÞY¾û¶^fllLÎ¤§§ïÙ³G/`2ÙØØ(_µÞzÀMèÊ9ÍËËÓ[§Ü>e<ÈFÊc®dÒú8n©/^èB||üöíÛç9«ë×ëlkkK¡g«««õb£££rVO>ÎÕàà |µ¢¢b»ª?~¬S	íÄÄ,Ë9NsIÉUllìÐÐPÞ³M. wRnÞ¼ù¦·þÛ ¬À"3¥ÇßÄÄDyà>xð ÊÙË/Ë©í¿qþe¶··K6vìØ¡gµåôÒëJ½Ò¨¨(Y¶^ÉóçÏ9"_-,,4wUFlº`fGå:ÍO§ó¢rúàÁ9½uëÒZV³¦¦¦¬wØõn=äyeÛ>eÄÀÀÎsÊMvç³¾QYå!^Íuä'ËsC5Ò¶Þðt+W®Xýû÷ëtîðð°u:TZVËÊòÕ«W­Ã³n7ºõ×ZñíPV`iþd¿Ï;wN÷<oa¦£££%®²`fM³¤cbíÁãÇç.G@$Ì­Ä`||ÜÔs?	dú|¾»wïØtuuUï§NKvÌ0?T·nM¸m²KåØ±cÖ fee?ÕùYU@9=z)·±¤y2¬Û³2Ã,Ý[G__4µØ¶môO/ìv»ÍîT­­­æbrÍ7oÞº7Sww÷¬eæeWSYYÐ­ÎùÜzøo²KBF«:r9³O,ëË®¶CFÃÚ6IHp9ô-"7nË¼y±S¦Ö1ßïÏËËÓ¢ìÞ½[d'Ë_~ù¥Î|êý7¯ë¾Á:ôÔéî×U¹fYZeMÑüocÖß>e<ËCíÅåÔ:0]Ü²z<¦¦&Yá×«f;­tt¥c5¹Ýu6xô 	A[À5HôRj~ÒÒÒtß`Q9¸¬ï¼óÎdìnÊªÏ0ä´M¾wþ·þ¦cÖåß>eîð"£:]ºtiÑË*>3Ó÷H9233Ê!6Yv¹ú¦o¿ýV/ÓÝÝÝÜÜ¬ûôZg²FîTP²¡ßb½>u[=Èõè¦º÷oÀ»nt·k×.©é3gtßZù^ÝøèÑ£ÖbÍçÖçi·@Y%øµ<ÚåôôtÍ<^KGG>WaVòóæÍÛ·oß½W×ôööêd¦iN~øá&ö====ÒÁ¥¾«Ä~ÝÜ[¹féÇËïgnÅÕ«WõGª6æðáÃ²F¾´aÃY#­PÉYs?uç&:¶I_Íäv5`otëó´Û ¬À"ÕÃ«Îd`$Áò >ëôµº²²²yÞ4)àF¦åÁÓIÛ¾øâsd>2ÊÐ-::ÚAdeeÉ@Ó~ó¦D«½½=--ÍDK¯_ÖëÅdT:û¯®x£[¿Ú>e²Êe²ÊeÝ3g¶nÝ@øáøïÿþï«»¬ÿ÷¿sçÎ^ÂÀ_ÿõ_?xð`Õõoþæoþ0ð³ý²@Y)+²RVe¥¬PV(+ePVÊe²RVe¥¬ÊJY ¬@Y)+²RV" ¬6²(ë"ÊÌÌ4eä> ¬Ê:_'O<sæõ»ï¾³Íæ½÷Þãw	 ¬¯7445==mÆ¬¾ |ò	cVe·ÛÝÜÜü»;Äë¬Êºp³¾@Y-±Y²(+G²ò»PVÊ ¬Êe¥¬ÊJY²@Y)+²RVe¥¬PV(+ePVÊe²RVe¥¬ÊJY ¬@Y)+²RV(+²(+ePVÊe¥¬ÊJY²@Y ¬@Y)+ÊJY²(+e²RVe¥¬ÊJY ¬PVÊkIGGe¥¬E000k·Û;;;)+eÎçóÆÆÆº;wî0f¥¬ÐIJGqq±$6~4ÊXVÇn·ïÝ»×ëõFÞHYËDÆ¦EEEN§3###¦)+`ÔÔÔHPÇÙ³gý~ÿ¤°´¼^o^^Íf;pà@___Äÿ¼°T|>_IIÓéÌÎÎàéßUVÖîîîíÛ·GGG§§§·´´PVX-jjjWllìùóç#úwUZ[[+r/ããã)+?3ýèÐ¡'O¬µÕÌß¾;##C~$..N~ü)À±é'tú÷Þ½ks#¬²NMMmÜ¸QûTVVÊÙÏdeeQVXqkvúwµY6oÞÌl0!¯×ëñxdTPP000°Æ·ÆjÚ78::²@X1Ó¿iiikvúw599¹»»[Z[[wíÚEY |0ý»*ËÚÞÞ.£Õ;wRVLÿ®â²r¤+LÿRVÀ¢aú²Ó¿°8þ¥¬EsçÎ	*Ó¿°P:ý+§LÿRV@ètúWÆ©2Z];ýFY)+,	3ý+qeú²BÇô/e¥¬°8dlzöìY¦)+eEpÿþýììl¦)+eêëës»ÝLÿRVÊàüùóqqq)))UUUlÊJY tõõõÙÙÙN§?PVÊb¦åÔëõ²A(+eÉØ´¤¤DÆ©III555lÊJY tõõõ)))Õ³gÏ°A(+ey½^·Ûm·ÛóóóûúúØ ²@|>ß©S§dÑÔÔÄ¡¬BWUUàp8%±lÊJY D¹¹¹zð¦)+eÐùý~þu¹ü²RVXI©é_ÊJY`¡¼^¯ÇãÑ½;;;Ù ²@dlZZZª`ú²RVXúúzË%Y-))áà²@è¼^o^^ÍfËÉÉaú²RVßï/..Ö½¯]»ÆÇÔPVÊ¡+// ÚíöÂÂB¦)+eÐõõõéô¯Ûífú²RVÏçÓ~KII	Û~¼ÿ¾òû¢¬ÖôØ¿:ýûäÉ0¼###gË-999qqq÷îe²@8êììJÙl69çéßüüü>úHk*§n·ûÀüú(+×(°¤¤Äáp$%%]»v-ïêÃåNZ÷Oull,ÓÂÂEMMMZZÓé,--ÿiÕ7nÈ5`eNNKY`åuvvºÝn»Ý.e±àª¸Ï÷ïßçw¬kdü*£X>º²ÀJé±SRRV×±å»óçÏ5'NÈÎÎæøVLMM9øÃjRGGRóóóKJJòòò¼^/¿VÊ+@c³Ù$KÒ§ÕûvíÔ©SåååáùÖ ÊJYD8sðáÕ××³A(+eÐÝ¸qCêp89¨e¥¬:sðüü|ö¥¬Bgþàr¹dÌÊ¡¬Bgöþ---õù|lÊJY Dæàa~ì_PVÊ Üùýþââb§Ó)£Uöþ¥¬D§GQQÿRVÊ¡óz½Çf³ôÑG²RVßï?qâû7Ì?úõwZ[[333£££322ÚÛÛ)+°¢Ó¿±±±çÏç`ôXeMNNÖûWYY*W¼ûî»yyyü.,'3ý[PP000ÀÁª)«Õ¾ûî»·Èö½÷Þãw	`ycÿ¦¥¥Ý»wÕZÖ¶¶¶Ã3`e1ý)ëøø¸Çã ¬V×ëÍËË³ÙlòpÄô/VwY<8::Ê¾ÁVuú÷Î;l,wYå	Ý_|¡Û·oÔÕÕ%&&fffÎÿÚvíÚ566Æ»n¬3ýâÄ	¦±be=sæ.deeÉBmm­,ÉrUU<ïÿµImÀ²Yéß>øàOÿôOÇÛo¿]]]Íf§¬;I85¨º »¤_½zUN>ªeçHÂÙ²Mÿ¦¦¦ÊÃã;~ùË_nÙ²E/Àö§¬¿7==­CÕM6iY÷ïß/kêêê¬cVÊ /Ïôï?ýÓ?Éc£5?ùÉO¢¢¢øPÖYfÏ;'òtO²ÊøU¢££ßÿÊ uvv.çÞ¿ò)#ëááa¹õÿüÏÿäwAYË:ëÙ`aËçóÙíöåÜû÷¯þê¯Þ~ûíòhÙÖÖÆo²¾fÌªÕ·ÍPVáfÙ¦|öÙgÕSøPÖ×µ¢¢BV:tÙ`ae§IÎ×¯_ÿ·û·×®]ûÉO~"÷äã?æ÷BY¯¹¹Y'~%¨²päÈY¿£GVVVêe³O<Ñé_­ÖÔÔ¬à=IOO»±nÝº?þã?þõ¯Í¯²þ/^h_oÝº555e®WúúáRVa¢¼¼|Ë-? ÜË*FGG¥£2HÕÃ'Éòõë×å´££²Xq>ÌÉÉÑéßÎÎN6Â½¬º¿¸´Ó¬ioo5uuu²<88¨sÅfüJY,0þe©à¶¶¶àñ«Õ¬yüø±b)+åtíÚµ¤¤$¦±úÆ¬+²xßþö·º÷¯2ýÊJYNÆ¦GÑéßªª*6VqY«««)+TSSâp8JJJ|>«~Ìj³Ù¾þúk³¼k×®ºÓíÛ·)+%âõzÝn·NÿÊ2«»¬µµµ§ªªÊ7øÖ­[r(M5emoo§¬MO8át:þEäuzzZ®KÚÙÓÓc¿;vÌzÖ²X,ååå			Lÿ"ÒÊ*f-kjjªuÌjAY,õàLÿ"bËÚÕÕæv»>*goÞ¼ÉÀ¢óûýzðþ-«¾°*=¦Ý½·,LNNÊuÿþýæãä(+¸sçÕét2ýH.«èííÓ©©©M6?~<àKccc¼ëÀBx½Þüü|¦±&ÊúüùówfÌFÆ¬ÅÅÅ:WLY@Æ¦%%%2NeúkhÌ:11ðÆV	-G°pRËöìYý5TÖéééôúÆV)kLLLÕYÞ¶með¦¼^¯<¤ÈcÈÞ½9ö/ÖÍç¿jÌ0¨¥¬æàóùJKKNgVVÖ;w^u1ùRrròøÃÏ>û*«Võ­«æÝ«±±±keðZÒË´´´×îýg³°Ûíl:DNY'''ù¬×××çv»Ç¡CdyKÊ³y©é;ôìéÓ§å¬Äm)«õÕVùã6o¹³eeeÀÜü~¿Nÿ¦¤¤Ô××¿öòëÖ­û£?ú#ë·ß~[pØ´²¶´´È_vkk«µ¬K÷Ñr:ý÷ßüfÿÊc[­k>ûì3Ê,«üY×ÖÖ644¸Ýîªªª=,_¾|Y/^¼HY^¯7//OäAcpppþß¸~ýú¨¨(ë?ù?¡¬´²êµ»»Û¼ì:>>.k$´Y0ÈÎÎ¾wïÞ~»PðèYÍsÀ(Xõe=wîüe×ÕÕÉòÆeáÂ²Æìl=@?eÖ2sðóçÏ|ðþô§Ößzë-6,"ª¬/^¼xÿý÷åûéÓ§:-ìv»&+**(+°ÆéßC=yòdáWøw÷wrs¼çX­eÕy`9½÷®Õñ«õý¬|>+°ÉØôÄ!Oÿk«¬CCCñññÖauÌÊç³kÖ¢LÿkkÌOÆ¬9ö¯<°A@YC/+cV`3Ó¿iiiLÿ²2f° Lÿ².Çµ»»zz²é_PÖE.kEEùLÖ1kaa¡¶´´PV "1ýÊºÈep^¹req¦XþdÌÊ§ÈkÓ¿Àµ­­mÏÏªïé²_)+Iþ°¬SSSÀÒsçÎ1f"~ôÓ¿ÀRuÖ½(+túWNþ(+Ðéô¯Se´ÊAð%/ëôô´ÔôèÑ£ÁeñâeV;3ý+qeúX¦1«µ¦²,ÿº.SV`bú².p6xll,11²+HÆ¦gÏeú²¶´´È²eVÊýû÷³³³þ"¤¬»wïîïï7eýî»ïAìvûï½ÇïXtn·é_ ¢Êj®Ä,_òî»ïæååñ»×ùóçãââRRRªªªØ@øÕï÷/°¬ÌK­¾¾>;;Ûétrð`ËÚÐÐàv»uà¬¬,³?°Y¾rå¦¬@x2Ó¿rêõzÙ ÀurrrAY¥&cÓ¤¤¤6e]:XRëÖ­³YÄÅÅ±M/+G×ë5Yý×ý×ææf=ûãÿcYåÿsÏ=C>ïÔ©SN§SþO¥¦Ö/ihÙD@õäÉÖ5Ï?§¬À«ªªJHHp8ÅÅÅò*CUëW£¢¢(+emjjzî	)ÿ;vì0^VV&kä&)+°Rúúúrssõà²¬#Ô¹_f[+_ÖÉÉIëÇWTTÄÇÇ»Ýnfpà÷ûuú×årYþ s¿fØúóÿ<x~ÀÊ5ø³äeatt²+HR*AÕé_ÏgýNkMÍÞLóÃV¾¬úÏ©Ë·oßåÊ,?¯×ëñxìv~~~ggç¬1»3ZÂ±¬òÏ¯`ª®®6qîïï§¬Àò±iii©ÓéLJJâØ¿Àª,ë³gÏ®_¿¾iÓ¦¶¶6]SWW'AíêêåÑÑÑ¢¢"9#¤¬Àª¯¯w¹Õ6°úÊ:66¶qãF=ÀaGGäóéÓ§ÒÑo¿ýÖz±©©)Æ¬Àòz½yyyòßóªé_«cÌ*¤¦MMMò_íóùd+TI¯,_¼x²KÄï÷ëÞ¿×®]ãcjH(«´óÖ­[ZVIUâªë¥¬]]]²pìØ1Ê,ºòòr	ªÝn/,,dú²êË¨2TµUé¾Á²þôéÓXD:ýëv»þ"mÌjæu÷ýgìÛ·O7mÚÄ»nÅ%OdKJJNgJJýDrYù¬`è±uú÷É'l òËÚÕÕÕÐÐ`^­­­¥¬À¢èììÜ»w¯ü[É)Ó¿À*«õ0L²`*KY8¤¤¤k×®±A5TÖ¦¦&©iKKíïï×(+²´´4§ÓYZZÊÞ¿ÀÚ*«¾u5`à¥x¿eÅÑÙÙév»ívNNÎÃÙ ÀÚ*«7øèÑ£³æÖãñPV`þü~¿û7%%cÿk±¬z°óèæcÏ/]º´sçNñµ½½ÝúI®UMM9øTÖhY¥½½½úù¬zvûöíú¥êêj9«ouMHHSVàU¼^oNNü³äççwtt°Aµ[ÖàÐ~þùçº!gÇÇÇæ`þ O@ëëëÙ eý½Ç[ßl#ËiiiìÁÌáÆTÃQ\ÌÞ¿eeÀºgÏëÙ¬¬,ÊÌÊü!??¿¯¯PÖ@ò144d-«Ûí¦¬@sðË%cV6@YgqóæMé¨îÄÔÛÛ+gËÊÊî0L«Ùû·´´Ôçó±AÊ:Ë§ÜÄÇÇWWWôÍáÃ9º!`?pì_²òY7Àøýþââb§Ó)£Uöþ(+eD§GQQÿõÍ¼xñÂf³íß¿²ÿ;sðÇ#ÿô,³AÊúÆt¯¥®®.ëÊçÏSVD¤uëÖÙ,N8a¾ä÷ûå¬û~(ër9<¸ÄÆÆÊõèÁÖm¥¬°¬Êi]]Ý-[ôï¤Ó¿ò¿pþüyýPÖ7355ekkkõXýýýKqDCÊ°¢Y5g¥¯ºF§ØJe~â~>ë+WèpÁab×®]fªdlªÃÖ´´´÷î±ÊºÐ²=zTS9RUU³aÃª²,AFG¨?ÿùÏõì;w$¨:feú ¬PÖS]3Ì322(+"þÍèô¯ÝnS§ÓÉ(ëBËÚØØ(miiîîîà².ÅAù)+VÜ~ô#³W°ÙÍPÖEØ7X¤²@Y±vèô¯Ãá0o¼Y¿~= ¬-ëØØØàà I,eÅZ`¦å½,þÕ:x5eMLLLMMí!+óòò(+"ü!66VF«2feXÚ²ê³>úT¤¬ÓÓÓ'O/^¤¬XítúW²*qeï_ËQV+)krr².ë'¶RV¬^LÿXù²êh8V;¦KYù9D¦¦&¦PV`Øív¦PV`Adlzþüy§º6ÊN§Ó¿(+° ÖéßÎÎN6H+kBBBtttzzzcc#eÅbú@äUÆW¯^/îÛ·O~$+++//ß%¢©©);;é__Öøøx=¾Ä/eá§Aâââ(+B688xèÐ!»Ý.ELÿü²FGGÏºÌl0N÷þgf.«ªª`M5**Ê,ÇÄÄPV,ÃQRRâóùØ ÖJY7oÞüâÅeÊóz½n·[?yIÙ ÖVY?þøã+W®Èz<Ê:uêÓédúÀÚ-kKKËÖ­[£¢¢Z[[)+BöðáÃ¦¬õ²r¤,ÜÈÈHaa¡üé_²bAÊËË2228øÊJY± ÷îÝËÊÊr:¥¥¥Lÿ ¬u-úå/¹î÷îùzÍ±þ@Y)ë%KÛr8oz%~¿ÿìÙ³±±±Lÿ ¬uMkhhÊPÕ¬eY#£Øù_É½÷$¨U+Çþ@Y)ëf·Û¥£+eÍúõëçóí+Ù»w/Çþ@Y)+þoë(öUdºeË¤¤¤úúz¶$ÊJYñ;#`îWçe:ÇwÝ¹sÇìý;22Âf@Y)+þ`*~ðÈrZZ¾Îzá·Û­ýÖÑÑÁÖ@Y)+é Õê7¿ùMðÅÌÞ¿ûe¥¬°¾j¨zçÎùªÃáøôÓOþ@Y)+Bçõzóòòd ËÁPVÊÐù|¾§ÓTVVÆUPVÊÐÕÔÔ¸»Ý^TTÄô/ÊJY:3ý+§üe¥¬ßï?qâNÿ³APVÊÐéô¯Ãá())aúe¥¬×ëõx<Lÿ ¬eþå£ßPVÊaúe¥¬XLÿ ¬é_²bÑ0ý²RV,3ý+§Lÿ ¬¡3Ó¿2Zeúe¥¬XóÑoW§²RVn``é_²bèôoll,Ó¿(+eÅBéô¯dé_²bA¬Ó¿²Ì@Y)+B$cÓ³gÏÊ8UF«2fe ¬¡»ÿ~vv¶Óédú(+¤¯¯Ïívë±e¡;þ|\Ò7ªeEèêëë³³³ív;ÇþÊ±Nÿ>|ø!òûý2Bu:qqqååål ¬]JJNÿ>yò!òz½n·[ýÛßþ!òù|§Nr:IIIUUUìý¡&$$ÈPõÈ#ìý¡ëëëËÍÍÕ½½^/(+Bä÷ûuúWF«ÿðÿÀô/PV®ªªÊårÙíöââbÏÇÊy½^Ç#MÍÍÍeï_ ¬5t26---u:[¶l1+(+eýeee6uëÖÍqáúúzË%Y-,,dëÀZ)ëØØXbb"ejTT,KV_W¯×'_ÍÉÉéììd»À*kKKKZZ4À¬ù |ò	eë×¯õ_üÂ¬Ñ¸Z/ã÷ûe*£ÕüÇdï_XseÝ½w¿)ëwßgÍï½Çï2¸£:MMMÕåòòr	ª¬),,äà°FËú÷Æ2fä>`Ì:kYõe×;wöõõéôïÞ½ïß¿Ï¶ÊjãuÖ×6/²Z[«ýRSSÃVµXV3ÇKYC¶êKfÁn·òÑoÀ1k(dÌj²ªÓ¿ìý²ndd¤¤¤Äáp$%%]»v#E®¦¦&--Íét²÷/PVÊºÎÎN·Ûm·Ûsrr>|È_6PVÊ"¿ß¯ÇþMIIáØ¿@Y)ëÔÔÔèG¿r@% ¬5t^¯7''Çf³åççwttð§²ÈçóéÁêëëù#ÊJYCwãÆ	ªÃá(..fï_ ¬5t÷îÕéß¾¾>þv²RÖ?¸³òW²Îìý[ZZêóùøÊJYCdþÀ±²RÖñûýÅÅÅN§SF«ìý².Nÿ:¢¢"öþÊJYUÝû×ëõò×	².ßïgú(+e²@Y)+²RVe¥¬PV(+ePVÊe²RVe¥¬ÊJY ¬@Y)+²RV(+²(+e²@Y)+²RVe¥¬PV(+ePVÊe²RVe¥¬ÊJY ¬@Y)+²RV(+u­­­ÑÑÑííí@Y$99Yï_eeejj*ePÖE³aÃ9ÝÄåråååñ»PÖ7ÐÖÖvøðaÊ ¬`||ÜãñLLL0 ¬oÌö==;<<|ðàÁÑÑQöPÖjjjÚµk×ØØïºPÖEh³ ¬ÊÊ"²@Y ¬@Y)+ÊJY²(+e²RVe¥¬ÊJY ¬PÖ;vì­·ÞJÆ÷þüÏÿüÏþìÏØ+B¶¼l¶ÃòûÑ~´mÛ6¶Ãòû¿ø¤¤$¶ÓéeüøñÿÅ/~ñ¨¨(¶Ã°Ûí²ýÙËïÝwßMMMe;,¿ÒÒRÍöõ×_³)þçöûý«»¬pãÆèèh¶Ãùù¶ÃòûôÓOsssÙËï?þã?¤¬ÿõ_ÿÅ¦x-ÊJYAY)+(+ee¥¬²²RVPVÊJYAYAY)+(+e]3þíßþíÇ?þ1ÛaEüå_þe=ÛaùõÕW|ò	Ûaùõôô¤¤¤ü÷ÿ7²@Y ¬²®V­­­ÑÑÑííílå×ÐÐ`³ñï³¬&''<³mÛ¶¦¦&6È²­&8ééé---lÊõ`©©©le655%Ïl(ë2ûòË/O>===-ô.²lâããdANÙ 5òmØ°°ÌN<yæÌÊºÌdÀÔÓÓÃvX~ò<fxxXäç45òµµµ>|í°äiVV(ë2þæoä©¤<¸?zô²lÚÛÛe³Ë¼Êc²F²ññqÇ311Á¦XNn·»¹¹ùwÿ<uyEEEÉBoo¯<¹a,íÛ·ël$vÇlÊ±<8::Ê¦Xîÿ?ÄY67o¶_Ù Ë9[À§¬¯©©i×®]ccclM,a9úé§×¯_îîîwÞy²ld*ÛºººdüÊ¡¬)11ae]k=æv»eÌÕßßÏY6TÙòr*ËlÊe²Êe²@Ye²@Ye²@Y,ã¿±Í¶ûvÏªªªYóàÁ9.ÿÚÏÑv?~ü¸³úñ¨(+QÜ3$¢R»GÉé+WÌ´·nÝ2kÒÒÒ$½3êêêjkkåöì1k¾þúkYóìÙ3ó----²¦µµÕzÕÕÕl²frrÒ:¬ìèèÓ¶¶6]ÒJõbCCCröéÓ§rºsçN]ðá¾ò¥¢¢¢5`­¸ïÞ½[/_¾,Ë/^äwPV êBOOnxxXN»»»oß¾->/ ¬			20Õ¾nÜ¸QF¨² CÒôôt°^½zUÖtuuzå:MÅÇÇÇ­×	²CªÒ×ë×¯ÇÄÄèøR*¨s¼Ï=(«êGµëì±põóÃõeZëÏ;'+%º²¬1¾pá¬ÑËÂÍ7ùEcccGÂUVVjeÄ)µeUÎ677'&&ê¤îW_%+·nÝºoß>Y°^ù/Þÿ@Öïu»Ýëá·PV ¢ÜºuËìR¤¯³^¼xQ>¬e½û¶^Rò)_ÕycÔ¼ÈªóÀ?§åôîÝ»f°+CU³»yù(ÚHÇ£ñÐJ,eØ*CCCfªÑ×e].WÝÝGIâãã%ÓÓÓò]²¬7âà1«~	e"¾ÔÚÚÚ*§.]2Á;yòä¶mÛÌYÏg¾E³ZUU|U£££ºÛðÔÔTp>³Xÿ·6[aa¡.dffÊÂõë×7nÜ(áLJJ21/ ÊWåìÇliÍdrrò¬7Á ¬À ¿~¿_SSSu<*ë¥¬Ú<Zuo&>zôHNåb:å+Ö9âÍ ¬@äÓ½¬ûûí·²fppðå÷ï¨ÉÉÉÑZgw=¦+¥¦¦¬R#y~Ó1kww÷U(+UÃprrRÞÔÔôÚïª®®Öa®Q.¿mÛ¶¼¼<ýÏç/mÚ´)¸¬zE¹|Àµ°°P7Áo ¬ÀzõêUsVÕôå_5ãããÃVL¾råS²@Y ¬²@Y ¬PV@Y ¬PV@Y ¬PV@Y ¬PV(+Éÿ@áöpòSIEND®B`


õÌ·àu²@Y ¬²@Y ¬²@Y ¬PV@Y ¬PV@Y ¬PV`öì¾¥öÿý9ED|öÙgÓ^ùÅòÕ®®®ÞÞ^Yä[Mû¦rË·oß¶¾øB|÷åqÊÌö_9"â£>²^"Î[¦~¯®®®¶¶V®ùùç¿êvä:æ6­×OO:eý´  @å_UUUélË²,ìÜ¹Óz³ÑSê¦ÈWwïÞ­÷GoÞ¼©×Oûúú233wìØ¡W0º÷®|ÕúÝ½¾^ØÚÚ*].×»?Bÿy5Ç¤õ!Û:>ªññq]HJJÚ°aÃÜÜ¾ÞfsssÚý´¦¦F¯644$ÊXV__ÿäÉß[õ÷÷ËW+++g¸«êñãÇzeù(þü¹,Ê73×ÇÇÇ¸¦uröìY¹²píÚµ¹~wþ=(+0¦òø,æ÷îòéÅå£LN¾ÿÐÿ²zX---ÕM6é§Úr	ª>úKéõB½ÒÈÈHY¶ÞÈ/ä«ÅÅÅæ®Ê0§fë±Ü¦ùét»¨|¼ÿ¾|¼qãvÈÚ-³&&&¬wØäjNßÝ¡ÊÌY__nEyHv»»»gYçTVyGs«dy²ê²F¥ªªJÊ£[>½b °¸¹¹¹¾å(//·þ»wïÖ¥:1ëæP£µ[2)ÊrEEuÓë´+aNßÝÏ§!~ÊÌý¿ì÷ÓÒéÓ§õ¢¯lIÝ)fÓqtt´ÄUÌ6Ißµ?¹¬^5ßÝ+£££æ2RÏü$@äñxnß¾mbÓÞÞîÛ-½ºéUÊ´iù¡zçñÝ#¦R÷ ¬À:tè5¨N§Ó÷áØl·ôfU^eôèoY¥Ü:cIQdhï+îÛ·ÏYz4î5µHMMºèóóóÍáTMMMæjrË×®]õX¡i»%ÌNMÓ0Yo¡kÃ7þ|wþ=(+0g2­êäúrê&YÖÝ®n¶U¦a-$Ö·¬úÕ«WËu^NíJÎÂ:³¸-î¶mÛdAÆþäOtË§Þ³oX¼ÕÁN7wÏÚ-"Í²´Ü"ÿ¿»3ëÞCþÛ²ER'µçÎÖÁ4¸e-((p»Ý² #Ú«¶[ét¥³¬ÜZì;=jBd¨õº´þPÒ!Í³ÃáÐ#ou§²o·²³³÷ìÙ#³»é>ÃgÒ~ù·þwþ=(+0gzÀÌL:5^¸p!èeqÊqú)ëúõë½Ê*C­,ÛíöÑÈ×_­×éèè¸wï1k^å¹ÒÉþët¶Ó­ÜúìAnGwIê±µ^¯iÑ9oË-ÒªS§Né±µòoõ0ÝZåÏw÷GèßC²süÿjy´5Ëú .=óý'­­­Ú9ß³LKâ¯]»vóæÍÛ·oë%ÝÝÝº±×´V7¿÷Þ&ö]]]=ÒÑM_uc® ®bî­Ü²ôãå÷ÛEEEEîÕWyjcöïß/ÈV­Z%HÉ$äò©¹zènöK½ÌäûjöæôÝýú÷ ¬À¼êáUFå!X"7ít_]YYßHñ½ÎdZî4)ãã?6gæÓLFÛèèhsát:e³ßìÑ>µ´´8u½¹&3?÷_7JïÙ³gNßÝÏ§!~ÊeÊe²XÖS§N­[·.ðæoþæ7¿	ï²þâ¿Ø¼ys7!àOÿôOïß¿öeý¿øÿ ¼ýöÛÊJY²(+e²@Y)+²RV(+²(+ePVÊe¥¬ÊJY²°ÊZ__AY5&&&Ö¯_oÊzßÇOúSÊ ¬þ:~üø©S§´¬ß÷]Ät¶nÝÊï@Yg700àt:'''ÍÌêññóÿ@YýïÞ½ÿ¹CìgPÖÀymõ¥¬Ê´Ä2³(+ePVÎeåw	 ¬@Y)+ÊJY²(+e²RVe¥¬ÊJY ¬PVÊ ¬Êe¥¬ÊJY²@Y)+²RVe¥¬PV(+ePVÊ ¬ÊJY²(+e²@Y)+²RVÀëééÉÍÍmhh ¬¨²²²ÄÄÄ;wîPVÊ¿þþ~ËQTTôôéÓ0ºçrÎ?/£êõë×ÃîÎSV@hª2ª×¨JY!çÌ3v»=öªRV@(êééÉËË*..ÓQ²BÅùóçãââ²²²ÂzT¥¬¥×××·ûvU9âñxÇEYK£´´4>>>==Ýív/§²`Tu¹±±~úéÈÈÈ2ûé(+`	FÕ¶¶¶eùRVÀbªG÷)+ $FU§ÓÙÚÚº¼RÊXX:ª;vlùíU¥¬EuæÌ¸¸8U<x°B~dÊX¨Q577w½V²xT]öUÃ¯¬6lÎÌÌlll¤¬.£ê'VÂ^Õð+«µ¶¶Vä^&%%QVýQÕáp444¬Ø6[oÞ¼%oùHLLt¹PUKJJVÔ^Õ°,ëÄÄÄêÕ«#""ªªªäÓ|8NÊK>ªnÜ¸qå¼fÖúúú5kÖ°5BsT]i/²èèhÊ¡6ª:²6Â¦¬iii²ÐÔÔ´eËÊ¡3ª.×7«YæemiiÉÌÌiuóæÍCCCÔQ5ËÊ" t´¶¶fggGEE-ï7«¡¬uT]É¯U¥¬ ªËþU)+eÅUív;£*e¤§§'//×ªRV@?>>>ÞáppZ%ÊHgg§ËåÒy­*e:ªòZUÊJY Z[[srrU)+e@IGO<©UU)+e´µµmÜ¸Q²RVÂ¨ª¯U²2ªRVÊJPy³ÊJY £êÑ£G£¢¢U)+e ªN§Q²RVÂ¨ª3ªRVÊÒU)+e ªLY)+SVÊAU9²RV¦¬U)+eUÓÓÓeT=sæ£*e¥¬0§¤¤DÓÖÖÆ¡¬æÏívÛívFUÊJY Pf¯*£*e¥¬(s0£*e¥¬QUË¨JY)+¤µµuãÆqqq'OdT¥¬UO8!MÍÎÎ¾²B(+eFUÊJY`©ékU¥©999¬ÊJY`þÜn·ÃáÐ3KbY!²À<KS].£*e¥¬deeñf5²@FU=­¯U¥¬¥§Uâ`ÊJY h£ª×ªRVÊyøðáÆ90e¥¬QõØ±cúZUöªRVÊimmÕ7«9qâ£*e¥¬0GjµqãF^«JY)+äáÃúZÕN«DY)+D÷ª:NöªRVÊyðàU)+e Ð÷U¦òZUÊJY PúZÕ¨¨¨cÇ1ªRVÊªzZ%^«JY)+aT5ï«Ê¨JYC«¬MMMë×¯ÎÊÊjii¡¬UAY¦÷¯ªª*##C*|¼óÎ;.ß%ÐU9²ÇÖàU«V÷ÝwoøvëÖ­ü.Â¨ÊÀ²677ïß¿­ÁBÛíÖQ6e-((xþü9eR<OIIª¹¹¹ª²îÝ»whhcà¨wòäIFU,`Y#"">þøc]Ø°a,466feeíÙ³§®®.99yýúõþßüÇÝ²eËðð0¯º£jNNNOO+^ÖS§NéÓéÚÚZYåêêjy~çÿ­I#,(+FU¬ ²677K85¨ºPPP +**äã'O´¬III)@XªÙÙÙ¼VQÖÉÉIU´¬»wïKêêê¬3+eÀ¨Ê:·­Á§Oùÿ·iÓ&SV_e!::úÝwß¥¬ÂqTÝ¾gg'+KPÖigV¶ëQõüùóªU+«/¡¬ÂnTÍÎÎfTEhµ²²R.Ü·o[×¨æÌFÕ=úôúõë2Y­'(Á,ë½÷tÃ¯UdYþ_<x°ªªJ¯ÃÌ ôGUdTu¹ª»téRbbb^^Þûï¿/E8vìeññqíë7&&&ÌíJ_ßï=Ê ôÝºuËn·K¤ªAYU·Û­öõõ93gÎPÖ9Êª§Oå+W®ÈÇÖÖVÊ ,FÕüü|N«,EEE'N°^rõêÕÜÜ:üJD¥æ¹¤®®Nûûûu[±_)+PU98¸òòòdÝZ/éììLOO§¬þnnnnö_%¨æÇëKYÚ¨êr¹úúúX!ÁU\|ôèQë%¥¥¥Û·o§¬¡²Ã±víÚK.±6L¨²z/_¾¬>xðÀf³yM±²X&£êUÁ;wRRR²²²²³³uû²ÿ_Ö»wïÖÔÔPV¡üX.ò¥¥¥¬ÅÑÖÖ&ëÓ§OWÂ» 3kDDÄ_|a·lÙR8E`ºyó&e°T£ªVQáQÖÚÚÚêêjsÞà7nÈÇäädiª)kKKe°øÜn·Ì¨°)ëää¤Ü´³««Ë:¿:tÈú©9e°h£jqq1£*Â¯¬bÚ²fddXgVsÖÊ`~½ªû²¶··;üüü'OÈ§×®]cf°ø>ª0ª"ª;VÅ³gÏ´£Û¶m±±1XwïÞmÞN²XPwîÜ±Ûí¼Va_VÑÝÝ-'&&>ìõ¥ááa^u`AÓ*1ª"ìËúâÅì)ÓõÈ#º­²X ×¯_gTÅ²Y?îõÂV	-g°Ø«åYÖÉÉÉÌ)úÂV)kLLLõYNMM¥¬Â­[·l6£*aYÍû¿jfõj)+y<=ö0ªbyU§Uéªyõj||¼×%²o¾ù&%%eGÕ/¿üRBÿé§JÚùE`¡Ê:66ÆÝXß~ûí,ÍÎÎv¹kûöíN§shKPVëÞVLÍKn?.QVÁU×NYü½ªGÍËË±ÕkII	¿,lY%¥MMMÖ².Ü[ËQV`åé°°°p	÷ªÊÀúàÁë%ò©­üj°°eÖÖÖÖ××ËS¹êêêÊÊJ=,_¼xQÏ;GYÌ<è^UYê>lÜ¸±µµÕzI[[[VV¿,`YuBíèè0»]GGGå	-3+ù1Ue`å%¼'öì)**²^R\üþûïó;ÂõôéÓÒÑºº:Y^½zµ,=V.1Ç[OÐOYÌJ&T­êªFOOÝn¸>ràÀù´³³_ª¬ãããï¾û®äóÉ'ºY8??ßkCqee%eàç¨*OÊõ©ùÒª^÷Jê"4/¢ÅÂU·ËÇÛ·oë§:¿Z_ÏÊû³ðG(ìU¸¬IIIÖ7avfåýYø3ªÂ^U`égV¯|2³Ç¨*sjbbbYYku²2³ðy­ª|dç%(+3+@GUÍÏÕ²Îyfíèè¤¬98//¯¿¿ßú¥'N8p@>Ê2ë+º¬æ=Y½fÖââbùØØØHYèÀ2­^½zÕëKwîÜY»vmQQQiié¾û½N+¬²NLLH8ËËË;RV`YªQQQùùù¾ó¨ÇãÜJwÍ%.]JOO·XA3+ï"`ÖQÕ¼YÍ´±577×ëB)k[[k+«¬ÍÍÍ;¦L©þ.ë©ùe²+9xûöí3¼VõêÕ«¾'ãÍÉÉihh`beubbÂk#°¤ôôéÓÌ¬þëûýy_UM­ï(Þ××ÇcÅuÚ£(+þþ~=ØårùùZU¹~nn®î2°9r5	ÊJYüÏ¦]SãããKKKýÿW###ÅÅÅ2¹¦¤¤ÈGYæð%¬Ð²NNNJM<è[ÖññqÊ¬´QuÏ=¯:ØO2ãÒT¬ôÕZSY'ªz.SV`¥ªgÎ! ¬Á,k[)+F>ºoß>UóòòxWpPÖÐ*kcc£Ãá¡¬@¸øæol6[\ÜåËY ¬!WÖmÛ¶õöö²~÷Ýwq>äyñÖ­[ù]KîÛo¿-**Ò×ª2ª²îÖ`½³ãwÞq¹.%UõUKKKÙ«Êºxe¿·ËÊÖ` GUÝ«êÿkUÊêúúúüü|=ØétãÍryy¹|MYò»ßýnëÖ­6íÍ7ßüÙÏ~æñxæ4ªêûª2ªÁ/ëØØØ²çÑ£G?øÁ¤©¿üå/KJJÞxã?ø?ð'®æUå©3£*° e]8X8v»=33Ó|*M¸Êä:ë¨jÞ¬u_Y9S°p"##»ºº¬Èäj³ÙUP,«üííØ±²¡ìõ×_÷z7·ú§Z·nÝÌ£ª,°ê¥)ëñãÇ­¼xñ²!%!!aÛ¶mÖKÞ|óÍ?û³?Õ¨:óûªfYÝn÷»SôHàªª*ù#Ü´iyô²²2¹D¾%eBÇÕ«W###%¥555?úÑbcc=zd½Î¥Kx­*°e³¾íyeeeRRR~~>[×ÐÐ&Aýá¸qãÆßýîwæKòÌXçåå±WXì²ú¾ÜÝ»wûûûeahh²aÇªeee¬`Ë1EoÞ¼)Ë]]]fT-,,dT¸¬cccò×¤'`ª©©1qîíí¥¬@XªâêÕ«¬`)ËúìÙ³+W®$$$477ë%uuuÔöövY*))Ocbbä`T(ë,ïO¾zõj=Áakk«äóÉ'ò÷ùõ×_[¯611ÁÌò¨_]]ÍÚBbfRS·Û]__/Mõx<2ÂÊB¿UÒ+ËçÎ£¬@ÈªyyyòËB¨¬òyãÆ-«îR¸êåRÖöövY8tèeBmTµÙlìUB®¬ºUFUkYUYY,þùç©Qõý÷ßgTBtf5ÛõU7»¦ìÜ¹SxÕÀ¨PVÞëX£ªËåç»EEE§²¶··×××ý¯µµµXr¥¥¥ñññ2ª^¿~µYY­§aSYÊ,í¨ZXXÈ¨_YÝn·ü766ê§½½½zpepT]»v-¯UÂ²¬úÒU¯câõ6ðT-((`TÂ²¬zÞàN[ùÛ¦¬Xájkk÷îÝûë_ÿz¾l·Û¿ùæÖ<eþü¹,Â¼ºyÛó.lÞ¼Yw¾¶´´XßÉ²b¸|ùòk¯½&ú166v```¾W~~¾|ûö=úkYåÏ¸»»[ßU?Ý°a~©¦¦F>ÕºÚl6i0eÅÒÕÕ%ÿùßxã­©L®Ò×þð7ª¦¤¤p0öeõíG¤Ëòéèè([±bÿØ$¥ÖKN8!Á[zzòòòUåYÖÇ[_l#Ë#°b­[·.>>ÞëBù»¸|ùrGUÍÆ¨,Û²ÊCÆ;¬:NÊë­·Þzýõ×­HS5³öõõ±WXæe½÷®>dXË*ù+Vss³üÈ@©Ê_Gdddlllà·,iQXÎe½ví<èALÝÝÝòiYYÙÂ²"øáz`ptt´,ÈÛÕÕÈö÷÷ë^ÕFU`yõÉ'III555¾o³ÿ~ÎnÈ¨ú7ó7o½õÖÞ½Ü,OXÓÓÓïÜ¹ÃùÌÊÝÊ¼¯*£*@Y)+¨K.1ª+´¬ãããòz÷îÝ=0£*°BËªG-µ··[/|ñâeæáÌ3qqqªÀÊ*«Ûí6§®®®¬ÆÇÇËõìÁÖ7m¥¬?:;;sssUXÖ	³[«ç2ìíí]3RV¬QU¡2ª+´¬^ïx£ïÏZ^^¾@§¦¬`T°RÊzðàAy8(**ª®®YµjUõYÞ²eefuþüyöªõyíLµO1'8ÌÊÊ¢¬Àzzzrrr¢¢¢UÊú¿§666JD;::|Ëº'å§¬X6¾üòËøøxÃñðáCÖ@YÿçØ`Ie²sÒÖÖær¹dT=zôèÈÈ+ ¬/ûûûMb)+à?­*£*@Yg^MY322º¦Èò¬²FkkëÆUÊê×³>yòDm6urròøñãrá¹sç(+ ¤£'NeT(ëÜHYÓÒÒtYß±²ªe?S9`Uãââ¤¬ªeå]äàªàµªe¥¬@@N<©§Uºuëk ¬¿üü|Ý«Ê¨PVÊ¤´´411Ñáp444°6ÊJYFUËË¨PÖ*kKKÍfÎÌÌ¼÷.eEªñññv»Q ¬¡UÖY8wîÜÎ;eáCN§S&~Û·o×=+ ¬¡UÖ¤¤$=¿Äøøxrr²,¼å#11²"¤FUy¶Ç¨PÖ-kttô´ËlF¨Ñ½ª2ª9rQ ¬¡[ÖÈÈH³CYôÍjìv»Ûífm5¤ËºfÍññqÝ,Ë¡¦³³S÷ª1ª²îÚµ«¼¼äcAAeEèùòË/õU[[[Y!Â£¬ëÖ­´ÙlMMM!¢¯¯/;;Ó*¿²r¦ ÒÒÒµk×r`T¿7«@Yàª6QeÒÖÖ¦¯U-))aT@YèkUÓÓÓ>|ÈÚ@YùëììÌÍÍ`T@Y ªwîÜam ¬@ £*UPVàÿüíßþmÄ÷^ýõ¹ª½ª(+àÕØØXÉª.û?ª=ztddÕ²ÿË+¥¿üå/åÓüà3üO?ýQe¥¬xeY¥¦ÖK^íµW­­­­æÀª(+eÅôeõzóWUGÕ72ª ¬3UR:ó%:ªÆÆÆJUPVÊ¬[·NS*ùü«¿ú+Ýíúoÿöoª(+eÅ<½ñÆf·+£*ÊJY4:ª¦§§»ÝnÖÊJY1Ö9­ÊJYQÕáp444°6PVÊFU²b©è¨êt:UPVÊ<|øÐápÄÆÆ;vPVÊFÕ£GFEEÉ¨êu& ¬ª(+e£*ÊJYÖ$¥2ªÆÅÅ<yQe¥¬hT=qâ4577Qe¥¬ÈÃÓÓÓ90ÊJYQõÈ#ÒTÞ¬e¥¬Â¨ª3ª ¬ªGåUPVÊàªN§Qe¥¬Â¨ZRRÂ¨²RVÛíÖ½ªgÎaT@Y)+æÏãñ9r$***''§­­²RVÌ_CCCVV£*ÊJYQUÏÌ¨²RVÊívÛívFU²"£jqq1£*ÊJY²RVtQe¥¬p0ÊJYO>Ý·o£*ÊJY×¯_OOOgT@Y)+U8 £ªËåêëëc ¬ówçÎÃ!£jii)ke¥¬hTÕUPVÊ@Ýºu+%%Qe¡²'''SÖ°óí·ß1ª ¬¡UÖÆÆFÃñ÷ç?üüç?§¬¡æÎ;k×®eT@YC®¬Û¶mëíí5eýî»ï"¦³uëV~!Bß¬&66QeÝý¬Öõ¾þô§Ì¬!ÂívëkUUPÖ°)+ûYC90£*Êre5Ûx)k¸ÐÓ*ÅÆÆ~ùå¬ógÎÛÚÚÊÊFÕ¸¸¸'Or`§²r¦P#ÕU²"Pn·Ûáp0ªeE <Oqq1£*PVÁ;wìv»ª¼¯*PVäéÓ§%%%QQQÙÙÙmmm¬ ¬¿èÕ8qQ(+æÏãñðf5@YYYY2ª²W(+bN«$£j?+(+æïÖ­[v»]FÕóçÏ3ªeE@£ê=dTÝ¾;e¥¬éììt82ª^ºtµ²jddäÀóÛ«úá¦¦¦&%%ýÉüÉoû[V&ÊJY1?þñ#""¤¬?ùÉO¢££_ýõÿøÿ`µ ¬óñ÷ÿ÷ÕË/K~ï÷~oõêÕ¬²b>eZµ^òïÿþïÒZÖÊJY1ø¸iÓ&ë%e¥¬§wÞy'22Rjj.ùÉO~"°fPVÊù¦JGW­ZUSS#nß¾]Öø`Í ¬óôÛßþö÷ÿ÷_í5iªTv×®]¬²@Y)+²RVe¥¬PV(+ePVÊe²RVe¥¬ÊJY ¬@Y)+²RV(+²(+ePVÊe¥¬ÊJY²@Y ¬@Y)+ÊJY²(+e`Yµ©©iýúõÑÑÑYYY---@Y¦÷¯ªª*##²(kÐ¬ZµJ>îôa·Û].¿Keæææýû÷SVeÑÑÑçÏ³5@Yç,âúéàààÞ½86@Yåv»·lÙ2<<Ì«n5#,(+²r¦e¥¬PV(+ePVÊe²RVe¥¬ÊJY ¬@Y)+²RV(+u^:ôÆo¤ÝnOMMMß«ëG?úëÁO?þñSRRX~úã?þcV×ÈêÆzðuÉdpo3...ìËúøñã	¶¿üË¿²þüó×ý×			¬?ýâ¿`=øé>Õõ«_ýUáS§NÉêúðÃYþøçþgY]÷wÜý×ý×ð.ëBøÇüÇM6½Î=ûGôG¬?ÕÔÔ¼êÍá«¹¹YV×àà «Âß~û­¬®ßüæ7¬ü÷ÿ·¬®ÚÚÚÅùv²RVÊJY)+e¥¬²RVÊJY)+e¥¬²²RVÊJYAY)+e¥¬²ÎÅ¯~õ«ýìgü·óSeeåÿù³ütûöíôôtÖÚÚÚduy<V?duutt°*ü199)«ë×¿þ5e üPV(+5Ô´´´Øl¶èèèÌÌÌ»wïò_afMMMë×¯Õ%«âúúz`òÇØØØÞ½cbbRSSÝn7+dV²>|566²B^exx899yñöWîAAAEE,;wnçÎüYZZ³ªª*##2«	y.BYýñÉ'|þùç»ÝÎURRÒÀÀ,ÈGk9`%Ï9äùõopÑö#VòMùKññqþkÎÉªU«X	³:~ü¸ÙU1+ ºººXþçúò$ùÈsWÙ¶m[oo¯õopÑöWîttô´ËYssóþýûY3IÂétÊß0eõóñ«¯¾glG±BfÕÒÒ"«KþwÉGùdÌ9Ëßà¢=ì¯Ü?ûÈÈH³Ãÿ?<þU1³üüü÷îyýUc?Æ²²2Yèîîg$¬YmØ°A§|I,çºñ¿¬ö°¿rÿì×¬Y3>>®dÿ³Ü»wïÐÐ«Â?f+VÈ¬3I,§)56².ÚÃþÊýßµkWyy¹,ÈGÃøÿ73·Û½eËááaVÅ¼ÿªñ*¸rå,tttdgg³Bf%sª©½½]æWVö°¿rÿì×­[i³Ùøÿ7³ääd0Êº@=/³ÓéìííeÌª¯¯O*kL>Ê2+ÄÏ¿ÁEØçÏÊe²Êe²Êe²@Ye²ÞqDÄ¦TWWWVVÊ%÷ïßáú³¾¥¾mûáÃõÓçÏË§ú.ª(+°¬Ô××çOJí=z$õ½±¬¼qã¹ÄápHz§ÔÕÕÕÖÖÊvìØa.ùâ/ägÏÒØØ(÷ÑÛ¬©©aýXnÆÆÆ¬cekk«|lnnÖ	­ X¯600 >yòD>nÞ¼Y/ôz«]ùRII×%`­¸·mÛ&Ë/^åsçÎñ»(+°éBWWnppP>vttÜ¼yS<WYm6¦Ú×Õ«WË*2fffêÀZQQ!´··M½r¦â£££ÖÛ@YåCGUéë+Wbbbt¾ê6ÞgÏyÕÌ úÐºõX}qÝMk½òéÓ§åB®,kÏ=+heáÚµkü"Ê,ÃÃÃÂUUUieâÚÉL¾eOïÝ»¬u?ûì3¹°»»Ýºu;wîë¿ûî»ºYÿm~~¾W¤åvø-XVnÜ¸a)Òý¬çÎýû÷kYoÞ¼©×|ÊWu»±î"5;Yu;ðãÇ½Â)°|¼û¶veT5;éÈË¯ ¬À²¢,((Ðøõõõi%2¶ÊÂÀÀAõ:º_Ön·×MÑct!))Ib999)ÿJõúf öYõK(+°Lè®Ö¦¦&ùxáÂ¼ãÇ§¦¦O=ù'ÕêêjßÒÃ'&&|óÉÌPV`üÝFDëÂúõëeáÊ+«W¯p¦¤¤ë¨òUùt×®]&ÖL¦¥¥Mû-YÊ¬ºáwddDïÞ½¡ó¨eÕæÉôivµêÑL:>zôH>ÊÕt¯¬3Ä ¬Àò§G'Y9úúë¯åþþþß¿¢&''GshÝºûìÙ3½PjjÊ*ÅK$ÏsY;::f¨2@YsÂ±±1)bff¦Ûíõ_ÕÔÔè+Eë§¦¦ºýÇã/%$$øµ²²RO (×÷YõtüFÊ¬8æS=UÓ'|b.õ[ucryy9)@Y ¬PV@Y ¬PV(+ ¬PV(+ ¬PV(+ ¬PV(+ÌËÿþMÒ$ IEND®B`


反趋势正态 Q-Q 图


uß¾ÚÌ·BKÖ³gÏª/;vìHÚ¯@²dÒ¥¥ÉX¶§úÃsJQ²D¶ÑZi«FªrMBE:bffF¿QríÜ¹sï¾û®vÌrÏ=Ú8)-MÖuëÖI0Ë³éÓÎ¬o¿ý¶j«WW`ScS_KÂ^=Ü>úÙ÷R÷ë5¨G­ oIÊ/¨¾ÔÔÔ¼ñÆjØ³<IÒc¥L$êÉ>Î]?û~l ­VÞ¶mÖ÷¬ª-«Ú¿4dÉ,NÂC¶³j¿¥ºíxmm­4ÔÁ¤dht:jãûìûÃ¥sÚWÅÕ7RCNª©ÔQÀL÷oØ°!éxª*µµjrÑd]to°Ää·ÉdR)¨bªñJòÕ~WÕ!ÚÞWy'Ú¾S!	§Þ¡*úçÜûlvPîÚµkåkz	y¹+W®¨Æ¦M¶lÙ¢/s³îp©åw×òþYÊNõ]»vÍ¬ò½Jý:R=«o%%%*³IV¬ÀâôûSw*j;EI¤©I>çÝ¡R!é­JVy6	¡EG'Õ¬ÚK¨ª÷Ù÷!SËë4UÞÄÛíÖ÷¼IµÃ+É9í®ä_ P¿,w¥¡Xu¹®Ú% f1©º°°P¢ôÒ¥Kª |6ðU;´u«°WÉäÈ4Uý:BRY~SUïÊûW	­oUÇ$+°P²ªy ªØ­°ÑªU]j¼ë÷æoêïîÞ½;u5U]½z55YÕä¤©,'«Ps&÷Ïþó¥Ô¬Zå§âªÓÆâJ±tÖUska£V¼TsLåVÍÑÿ(×®]Ó*WuDSUóòäÚ¾Ü9µ¦ßáªKÕ¨æ¤®[8YÕ¨¥¤¿fõí$+m,Ï5«Úõ:ç¹$	Î.0âiÇú%ú½ÁªJèÊ:YUÉøÕW_-%YU§Fý¨ËÚÆ©©)µ{µ/Z½®6µ¼¼}8þ¼£Æk½'<ç¼^oÒ9ÏN«u´aIiÎÞ`qèÐ!õÌÚ¸bm$+°·gé7ñÒPåÚÉ'%ÏòòòöÔÜRSKÞòª]µI­OVYA?=fÎdÕgM-pÕ©þRgÇ.¬úù0êF.K@²Ú«Êk5vWHÛI«%«£îÜ9O»Ý¾gÏUI«¹­ªoÜï#çOZ!dU	­F%MdÉ,$éô©çôQÐ¥KTxè]¸pAÉÓ¨csÎ·ì,¥?ÄÏ¢Zj$hç8°·Z­êúØ~çwÔsÊr5V*ÖFÕª²Lß«NázÈVMTÕ7ô?ÒjÖ@ ðìûS:¨Ó+jÉºpE¾ð.÷L;|®/Ò]>OÛó%«ÖÃúä$+HV Ãè*ÕÍ¤Ì;zô¨¤vW6îûöíu.µ:6*8»7¦ 7iD*¦&cÈ6£HÉ»sçÎÔ9*ú÷¦R»L&þ½É÷Îû¨ÒWÿsJ¿ÃSó85e%/µÃ½úêÏæ¨¿t~Oú§HVä:µ;tÎªpÌx6a5kÖ$ÕmÏ¾é42G¾h%Ý¦M>üðÃLw,<àö9vø|gÔÒ~ºwïÞE!éÉÉHVHVHVHV@²@²@²¬¬¬¬wïÞ=u]îßü;~úé+?|øP~zýúõ7oJãîÝ»KyéGÍyTyæ.è/Hþ"^u¼Cdû(ô~lwÍ|ïÌ3===²æç>ßóÈ:£££Úsê×»ÒßõûýÒG8qB]©[ÚÒØ³gþióf%?ï½÷Ôûö¹sçÔ:ÓÓÓr7mÝºõí·ßV+h!tñâEù©þÕ^B-D"rëóù2õt,ÿw¬@fd#«e¤ls%&õlù¨<~üX5·oßf±%O%Ï¯óÊ+g©»§OV«MMMÉ])ËÎ?çÎÔÖÇãòÓãÇ/ðV[·n©åVbìÁÒnkk»ÿ¾¼ÉdÒÖ87Í·oßöÍÒúäË/¿7)S§NeúêéXþï YhIùôéSÙþÉÖ|ß¾r+w¿þúk¹Ê)õé'kR522"±ºcÇuWe¹ªÚúKÒ«êH6¬Y³FÚú'yøða0ÖÔÔhoU9ÕÐöËsj¿Ú/*·ß~û­Ü=Vå>·´~xòäþkqÑ«§cù¿Cd2ÅÔ^D©d³ãÆkÖU6ñ²5Wu´HVÕV¡râÄ	Iµk×ª=Ia *`Uàz½ÞÔä8zô¨þWxï½÷ÔÎÒ»wïªYíUe´Ê-©¥ìØ1ý®×9;!£WOó«Æ2Édþý¾Z:|ø°Ctûöí¤M¶DÝöYÚ®ã¼¼<	Wihû$S7úª&VyyëÖ­5)DµWOû÷ïk+HI½ð	¤D"qáÂ-l®]»[êª]¯L;fi¿ÔÍ7³xuÃÕ;HVàúàôêv»S7ÇÚ~ËôkV%)Y¯^½¬ÜªÆD¢M^WîVUUieÍ¤¿ji±iÓ&Iµryy¹6êòåËÚjòÌ§Nj¬Ðèèè¹%´ZI[^BõFjp¦óêéXþï YIµª*×g³c¤­».qo°>Y¥VÉ!¬jÈúõëeg³¥ÓB_³ÎÌÌø|>¸»wï¹ÒþäOÔOõþµcÃjä­*ìÔîîEsKH­-Y®¢ô_=Íõ¾C>ö YE¢N6µG[aú|Õï÷÷÷÷KCJ´ùöë©êJÕ²òjhqjõTHQôÒêRñìt:ÕÈ[uP95·JJJ*++¥v×rKÃoýòØô_=Ëÿ$+15àEj&U5~õÕWÏ=Y¥ÒÊ85ÇFuÛ¶mIÉ*E­´ívmm­4òÍ7ß¨uFGG/]º¤FÌêWY"oC2FbC=D¿ªíÔ^nõíAGTckæ´¨:o×®]URckå±jîôÎ«§cù¿Cd2ü¼ê¶¶ZëÖ­j#.yúH$¢r.õ¬sMü©S§Î;wáÂµäÆjg¯µjGñ»ï¾«ýõë×¯^½ªJ75ëF[A<yR·òÌÏ¾ß/*;¦ÈªY*cöïß/KäGëÖ­%därWjèÚ1+Ù/é¥ýH^WÅ^F¯åÿÈÀ|W)¤pM°Ü+¨cummmi¾lñÎ¤eyêh-!$9>þøcíÌ|ª Ò6//O;p»ÝRÆé_;¢)ù422ât:µPWÏ/ËÕjRó¥óþÕNéÊÊÊ^=Í¯Ëü$+$+ Y Y Y Y@.&ë¡C6nÜXÀ2ððÿñÿ±²õïþîïvîÜyeà?üá·ß~»âõ/þâ/þeàÍ7ß$Y YIVÉJ²HVd¬$+$+$+É YIVÉJ²@²¬d¬$++:YGGG·oß·uëÖÁÁA@².jOO4ä]ªFüä'$+dÍÌ¹sç×wßgË[o½Åß@²¦åÉ'ë×¯ø<qâÜM¤øéOJÍÏÌÌÉ:óçÏ¿ñÆgd$÷öö¬sÈËË#Yékkk3L¬ÑhdýµÍ7JãòåË»ví"YéF£±¶¶6H¼Ì^îÉ:22²uëV©VwîÜ955E²m³º»»_þ«s¦Àê!åiUUªåååñxü¼°JD"§Ói6[[[_áÛ Y«ACCÉdòx<±XìÕ¾°²Iz½^£ÑX__ÿ§®¬Õ¦µµÕl6;áááeòHVÀ411CUUÕäääòyc$+`å	ÃY/óäJ$+`©¯¯7~¿ÿV"Y+WÿjçÕ¬Õ ¹¹YxùV"Y+Wc0É¼°µ··[,ÃÑ××·"Þ0ÉX¦W#·Ëj^ÉXyúûûN§T«¯äz5$+É«W³NL²V¶H$RRRßÜÜ¼ü+¬e­µµÕd29Îe>¯d,w±XÌçó­y5$+`YS'¶ÙlËð$À$+`%I$UUUF£qeÍ«!YËÑðð°Óé4ÍËù$À$+`eXÑójHVÀ22>>îõzóóóVú`%ð©y5cEÏ«!Y¯ÞÄÄ:	pUU´WñoJ²^¸®®.«Õj³ÙVÜIIVÀò299¹¯WC²¬°Øíöx½dåeff&L¦²²²Õ7¯d¼Tccc.Kbµ¥¥eUÎ«!Y/¤©djIII4ÍÍ YÏ¬d4WÁõjHV^±¶¶6Åb·Ûûûûs¼+HVÀRKÕÊÊJÁ ·92¯d¼(Ú¼®®.zddOWãóùrm^ÉxÎÔ¼üüü¬D²5¯ÆívG"zddOWSWWH$è½ÎÎNÅb³ÙÂá0½A²TªA)U+**âñ8B²²§æÕÍæzdd/HÔ××«y5ãããtÉÈ6¯&1¯d,I»ª¬ÃÃÃôÉÈ^</--5µµµÌ«!YKÒÕÕe³ÙúúúèdìÍÌÌÔÔÔÆ@ À¼d%D"n·Ûl6·¶¶Ò$+ÉKÒØØh2£Ñ(½A²¬½X,æóùWC²¬ð´µµI©êp8èdìÅãqu½¿ßÏ¼d%	ÃV«µ   »»Þ YIVÈÞÌÌLmm­ª¥¥¥ccctÉJ²@ö"Hqqq~~~kk+HV¤¹¹YÍ«á$À$+ÉKÇ=Ñh¬©©a°ÉJ²À´¶¶Zg1¯d%Y`I&''+++CEE'&Y]¾|yÛ¶myyy.kddd9³ÙÜÑÑAo¬sØ¼y³z'NØ²e4þ¿n·Ûçóñ·ãfffêêêòóóe8>>N¬[·nÜºS¬rUój8	0É®+W®ìß¿½Áª½½Ýl6Ûl¶¡¡!zdMËýû÷ý~ÿHVÐ¡¶¶VÚtÉ»wïîÛ·ojj±Á ×ÙÙiÅIIÖô÷÷ïÚµkzzY7 I$Á`Ðh42¯dÍXQQAd)9N§Ùlnoo§7HVÎKR__/¥ªÇãa^ÉJ²ÀD"üüüææfæÕ¬$+,Ikk«Édr:d%Y`Ib±Ïç3õõõª$+ÉKÒÝÝm±Xl6[oo/½A²¬½D"¡N!·d%Y`IÔ¼©VWC²¬°TÚ¼X,Fo¬$+dy5zÑh´¥¥¥©©)¬$+dy5©½QYYYWWg·ÛËÊÊÉJ²@ZWdhhÈl6¨»©Ò?Á`d%Y`qíííÌ«IrðàA)UõK¢Ñ¨Õj%YIVXójæS]]ÝÒÒ´Ðd2¬$+Ì+Ûív©V¹´j*ÕÒÒRý®®®ââbd9ÌÌÌ¨y5>y5óUó£¦¦Fò½½½+è+É/O$q:ùùù­­­ôÆÆÆÆ<ªuVggçzó$+¼$ÍÍÍ&©¸¸y5é¯+îm¬ðÂÅb1¯×Ë¼A²À¥æÕ8¾¾>zd%Y Ì«!YIVxnWC²¬ðÜB!æÕ¬$+<HÄãñ0¯d%Yà9hkk3Ív»½¿¿Þ YIVÈ^<///7Á`p]ï$+É`9ÃÅjµr½¬°$333ÕÕÕF£±¬¬LÊV:$+doxxØårL¦z$+,©TUójNg4¥C@²@öÆÇÇ>Á`¨©©a°HVXöööæÕd¥¨¨¨RÕï÷¯ÄKd%Y,#½½½R§Íæ®®®ôD$N§Çãihhàúq$+É¿¬T__o4ËËËÇÇÇ3ãP(FûúúÜnw  ?IV@Nv8ùùùMMMVRã¶µµiw'''9ÉJ²Èi¦©ÅÅÅccc>VåhR×ÖÖ666Ò±$+É ç½^£ÑØÐÐÝ¼yÅbIJÖêêjIkºd%YäÉd·Ûò<'iwãñ¸Ùl¦IV@®êK|¶H$"ek0ìíímkk¨N&YIV¹"[­V)+;;;×sJZ[[[ZZZQQ¡Íd°%ºº:£Ñèñxb±d½h4êv»óóó9HVÀHJ¦:NaY'«Á`øøãUcûöíÒt¹gÎ)**Ú¶mÉàÕÅb^¯W6SõõõªXÉzèÐ!Õp»ÝÒèééöíÛ·¥ÝÕÕe2HV¯Pww·Åbq8ôu²^¹rESªjøý~¹=vìÜÞ¹sG%kaa!ÉàH$j^Ü.^ðÂõéÓ§ªTÝ°aJÖ÷ÞO9sF_³¬^§Ói6¬ÚÞàÃKÃd2íØ±CKV©_¥÷Î;ï¬2233³K¢ÊÃÁ WFéO¬¼d³feo0,Äb±òòrÅb³Ùä¶¥¥%ÓgPójä»~kk+°JjV²SSS$+H*ªkphhHîêOÆ»¨ææf³Ù,ÉJ©U¬ÇUUUì¦¦¦ÒÒRý	×tJOIey¬Úø0X	+5Y/]º¤vüJ J#J[¾-8pàÄjjVé«®®NÝý+eë¢ç ìîîÕ$;::èF¬àdüø±Ê×³gÏ>yòD^É×wßd©P(TWWT.JyF£¬+>YÅÔÔä¨©ÒîïïöÉ'å6¬2%ÉQýuR%5ËËËç[¿¯¯Ïn·ÍfJU¬d½÷®¨d§¶dddD9sFÚñx+ÖêWÀ¢$#%++ëëëÝnwIIÉÓo¤­©©RÕëõÓoXÉúøñã+W®¤Ö¯¨Ú[·n©*d>IÊ¶¶¶P(ÔÝÝH$RWF£ÅÅÅ«°fåZ7^¾¦¦&ÉTÃ¡ßi¬$+MLL¨ëÕÔÔÔÌYË«$Y/^¼xúôiÀÕ××§ÎÊÔÙÙIo`õ×¬òò/¾ÐÚ»víÌR#Î;Ñ³MOO¬í$ÀåååÌ«Á*OÖ¿ßßÕÕ¥7øìÙ³r+¹(ª%ëÈÈHúÏ988èt:åQ$+°ÒMNN644HÊ"ë¡FÃÃÃn·;???+/Y>*Ï%)xýúuýúÁèïjçcJÇîÝ»oÞ¼©OÖÿ¢  Àçóñ·³ññqÃQQQÑÛÛÛÑÑár¹äÛv¦á$SKJJ"]HV1g²nÙ²E_³jgÈh÷²Öþ(|%YeN25jw%SívWWWWójFc=ójÉzíÚ5§ÓY^^~çÎ¹êÔ©¬kÖÔdeo0°Y,¤3<466ÖÔÔ¤óØîîî)yûûûéIäª«âÞ½*wïÞ-GIÁúÞïi#YÖ¤Î477/¬D"È@n¹^r4YÅ7äöÉ'6løðÃ~4==Ý`cXÑÊÊÊ<¨_âv»ÛÛÛxH8¶Ûí9¬>,Ô¬uuuj_1Éä±±1)[e#F|>dí|GLey½º^M<§÷Ó5ëQ3EøÙáÁ.KªÕÚÚÚùÎ¤Í«	BVÉú«7[g©­¬k×®í%íM6¬ÐÔÔd6%%_é¬ÿÏõÏç«YZÅJKKF£ª$ë¯©jUM]Õf¯Ê×Ï¤%4%YÕ*ÌyuU5¯ÆjµÃa:$ëo<zôkÝ³õx<r+ß¶+++µ)4ÒòÛï÷3X	$ëBG[åÿDróàÁ¹ÛÖÖF²9hffFYRÃ¤f---p¶°N§SªÕÖÖV:$ë"§Ñ(½|ù²>Y_Ü¥åHV`9ëêêRU¿DÂU*×÷ßßh4q½¬iM?íéé9þ|yy¹üS?~IÚ_ýµ´9B²9¢±±±¾¾^¿$®_¿~Í5Y_ñÈ­dUêèè¨vØõþýû²DÈAò%[»+ij2¤Zý×ýW:$kZ>,9zæÌiË×Ri|ùåê´jl°þý$+°êÅãñööviøý~ÉÔþð?úÑ¨VA²¦;õwÞø¼sçÚ-,ßUv?~drÊðððÆ_ýu©Vï÷~Ïëõrl$kGXGGGåöÂê®ª_õóY³¸>+É¬D¢ººZJÕ·Þzë¿øÅÀÀÕ*HÖtÝ¾»°°PÖ9kÖ,®ÏJ²+·ZµÛíf³¹»»ÞÉº¤±ÁZ²R³¹I»^×ëÏçs¹ ¿hhÎÉº¤d¥frÐøø¸ÇãXmhhØ·oÓéìííS'Ü§~ÉJÍ *>¥<íëë³X,úÓvttX­Vz	$ëó¬YGGG>J²«ÏÄÄWsðàA5L©¹¹¹¦¦&i5ÉÚ9ÏË¬%ëñãÇµk²&Õ¬òo&·$+°twwK1*©)uª¶°¥¥¥ººZ¿$nAAvF~d]Ü'O$8=ú|SX¶Dmm­:!LÒDÕh4*9*·Ú¦¦¦ââb:$ë²F²¯v½9WBRËJñkjj¤¨D"ôHÖt]¹råíYþY]ßSmuj~i¬À*033#©_ZZºð9:;;¥Õ¤´£ë@²f¶+8i'°DéáÃ©Ynrr²··W¾"«hº³¸¸Øh4666rN%à&ë£HV`¥koo/(((++«¨¨0Í?þñM&Ëåâ´É cápXbµ¿¿_Ú±XL¶¯½öÚîÝ»)U¬O>4=pà@j²>~üdV`0Tåj±XÇ?ýÓ?y½^zxy5«>M¥m6Õ&Õ&Y¥¬¬ìßþíßW399966&ùJÏ¯,YÙ¬hù¹aÃ©Vµóý¶¶¶Ò3É 3êz5kÖ¬yýõ×[ZZÔÂ¡¡!«ÕédH$ât:óóó¥Bíëë³Ùl.«¤¤Äl6k)dISÉT\<<<H$$Y;;;zz¤`å¿ÀòJÖXÎb±ÏçÿÜúúzùUû~¥TÒ¨¨¨ ¥¬çÏ///WcÝn·6Xk=zTîæåå¬ÀòÔÝÝm±Xl6[oo¯ÜÇãr·¹¹YýT2µ´´Tþé(à%%ë£G8#?°BIjêçÕ¨Ic¥¢5Í#xIÉÊµnª¿¿ßétJyÚÞÞ®_ÞØØX__´²T´ YIV §IvFÇ]]]²D]oxeÉj0Þ~ûmXÆÆÆ¼^o~~~ssóa)].Wmm­ú©D¯Ûí>xð ]¼Êdýì³ÏôK>|H²ËWãp8Xm||Õf³Iñj6µð2µ¿¿ÿYj$ð'$YwìØ¡]½­­MÈK¬@:$Õªªª¤L,..H^I¥ôIùgLÿ9¥º`&+ð²õÑ£GúË?~¼°°°¼¼½Á@$Ì,K]]]4T%Ø¥g[8¶ÌRój,ëdM½ÜÅãñ¸4¦¦¦HV #eeeIG4KKK²~ÂD"!°Ñhô0°ÂÕ0KµÏ;'íë×¯¬@ú¤¬LªP»»»¥rÍîÙÔIÍfskk+¬°dôèähaa¡:ÓéÓ§µpÍËË»yó&É¤¬I¥üOe¬RéL¦9çÕXÖÉzïÞ½'OnØ°áÊ+jÉ3g$P¯]»&í©©©ÚÚZ¹»víZYdöKz½^£Ñ¨NL¯+)Y§§§×¯_¯NpD$>ïÜ¹#9úÍ7ßèWòä	5+¥`jmm5ÍÎ«°|kV!iÚßßþüyÉÔD"!%¬4âñ¸ªD¯´9B²©¤ ìêêBmmmÚ>ÛñññÊÊJË%Ñ¬YSÍ«©ªªbª°²UþÏ=«URpUË%Y¯]»&>ødô$JÕEÅ¥B­¨¨Bs)SbÂá°Ýn·Z­Ì«V|²ªÃ¨RªêU¯ájl°,ÿüóÏIV@ÏãñÈ¿vWQÂ5ÑFRøª1¯X%5«¶OXÍºÙ;kÏ=ÒÞ°a³nTñxÜf³%/ÊU¾fô<HD_ÉÔÔÔÄ`%`µ%+×ºÒ744TRR´PJÏ777K¦J²Ñ¥ÀªMÖk×®?^;þÚÓÓC²©&&&R÷ýJÖvtt¤ópy ÏçSój¬¬òdÕIZÊ¬@êêjÒH$¢6JõH$ ¤¯Åbq:RøÒÀ*OÖþþ~IÓÁÁAu÷æÍjpÉ00 Y(·©ÇA¤rµÙlåååñx|á§ò´²²Rþ­¥*°úUM]Mü"æÛ¬X)$ü¤*µÛí®Yããã©«Åb±tIêp8¤Zíîî¦oÕ¬ê¼Á3ný~?É$ehEER¡J¸f77L&yBNäD²>xð@,B»ºvÙó¯¾újçÎêàëÈÈþJ®$+V·x<záIÖþþþgllL_ÕöövzÈdÔ¼qãº>«º»ûvõ£Ó§OË]5ÕÕjµJ¬ÈCCC'ia È( ÛÚÚÔõj¢Ñ(]äP²¦íG¤ÚÇ»÷ïßgo0r°f-((HªYNg5k,óûýùùùÈõd½uë~²´eSÂ&ä¦9³¦3¯&[­VYy5Éú«(ûí·õwÝn7ÉP¦Í¤^ôdI»555F£±ªª*°ÊõâÅ¥·oßÖ'«|m'Y±©k·Y,Ç#·¥¥¥I§ÅWóYûûûÝ©+ª¤ï/z`õ$ë©S§$GÕ ¦7nÈÝ¶¶¶ìNÃ422bµZóòò¶nÝ*iM²b9(­¨¨PÇS¥ÊZÓëõfñ<¡PHJU·ÛÍËõWW¹),,<útê¥oöïßÅJpìØ1i9rdÏ=Òø¿)~úÓ¬xå$ív»¾¶,:5ý'Åbeee«VHÖBrZÕ¾?.**úî»ïsyë­·ø[âÕêììI3ZÓÒÒb6%ûúúèOdQòòòÚC)~òP³âÚÔív'ÅÅÅéÄäÄÄd°|G Yç%U¦l)Þï½¥<É5k´öÚµk9ÎeKíûÕïÅmll%IædYÍb±tuuÑÉº5jéÚµkú>ÌèIÞxãIhÓÒ&Y±uddDTËæÔD"Q]]m4>ß¢·ÉÚßß¯.X¾K¬ÍfièÏ¬¿hk:öîÝôèQiÈí|gó'YñjÅb±òòr):m6Õjýøt¦Ö¨ëÕÈÿHss3unO<ÑÚ===ê7oÞ7nÜ¸fÍÙ`]¾|dÅr311!ZSS£J^ÊÝP(´ð£Z[[M&Ûíæ$ÀÉÁoÔõY¥Ö|A§&Y±455&£ó¡ñxÜçó1¯ Y3sàÀ5Ê±««kíÚµëÖ­ë%í]»v¬XMª««[ZZJÙ:çÅS;;;­V+ój53ISí³´º«I(ª««Ó/H­Y'''+++¥TÌ«HÖÌN,9:88(!:::¬/â¤ü$+^¡H$"9ª?Ñdgyy¹~©P¥TÕW¬V%©4HVäIM)Iëëëå^RR¢ÍaÊµ¦¦&??ßçóÓWÉéééx<®E,ÉTù<L&ù¿ÿþû¿øÅ/B¡Pww·vÅ·h4*)++0¯ Y:I¬EEE[¶l¹>KÊ7w«¥ÍfëííÈvYYYÒj^×«HÖç¬êâ¬wîÜ¶Õjdúôég&9B²b¥ªÔl6G"ý©µTÇÇÇ½^¯|Úëêê(Uõù_uóæÍª­®ZC²b¥L-))IZ(9ÚØØØÑÑa±X3ºx5òÍ=¼Ðëá¬xÉ¢Ñ¨ÓéLZø7ó7;vì0±Ú¡V$ë²»ÉåÉn··µµiw;ö[¿õ[V«5Ó9ÉJ²²X,GùÑ~d0þôOÿS@ Yì;wÎãñäçç¿þúëôd²×ÚÚºnÝ:ÉTËUZZêp8¾+dæÕßßo4%YÔ¼ºººââbzÉd¬££ã?øÁïþîï&Íg5Íg@²àáÇ?þqmmmÒO­V«vº`$+É,b``ÀápH|vww÷õõÙívý¤ÕÞÞ^ù½d'	zðàÁüüüÒÒÒx<®úý~ÇF¥ÝÕÕUPPÐÙÙI_ Y´JU³ÙÜÚÚªßÙ+q[]]m³ÙL&Sqq1^@²khhRuëÖ­ò'bµZ-Óéìïï§g¬@fÆÆÆ¼^¯:	ðo¼Ôì©_è"$+®ÖÖVÉäp8¤<dú655ùý~z	É,nbbBÍ«©­­USËÊÊÔUWõålêndÃaË,VVVJ	«_­¯¯/õâ¬@²¿H$¤H5~¿?éÔÙÙép8´É6²¦×ë=xð dæDNg~~~Rmª©ªª²ÙlÍÍÍn·»¬¬Lf«VPf¡¡¡Ád2iç|Ooo¯µ555mmmÄ*«:oAAréÚX,¦æÕÔ××HVÿ#¹XQQ¡V©8Ýnw]]]moo·X,cxx@²¬øÕîYË¥¯5¥Mç:nDBÍ«[.úd%Yñk©ªÇãXàQápØn·KµÚÝÝM YIVüF[[I%5ç$Õm½Ñhôù|Iójd%Yñ?ãããf³¹¯¯O[ÇcW$+É_íÚ-((ðûýRz<UIÐÔÕÔI¬d%Y±x<ÞÞÞ.ÕjGGGêp¤X,æóùój¬$+æH$ZZZÁ`]]ÝJíîî¶X,6-é|ú@²¬ø)LÝnwIIkCCÕj55zWd%Yêêêòòrm×îØØ«~T±N§SªÕöövºÉJ²bv»||D*Wmb«WãñxWd%Y³Ù4©µµ5Jñêõzóóó¬d%Y®¤Ý¼eeeý×m28	0dEÆÔV5Ü7HüýßÿýúõëCmm-¬$+²¡.ScµZð¼þúëÌ«@²¬X©Vßÿ£Ñ&&&è$+É¢Ñèðð°âgllÌårL&Nd%YsT__Ífs:%%%MMMY?UCC:	°ä+d%YsD ÅbéèèPw#ÝnokkËây$FcýÒ_ YIÖª¦¦F²P¿$KýÑ´´´H©*¼Àd%YsBYYDiÒÂüüü4ÇKKK9	0dÅ¯Á¤«jp:íêê5¹^dÅoôõõh×$ðx<Q333µµµF£±¢¢BÊVºÉJ²â7ÚÛÛ%%&ÍfsuuõÂ§ör¹«---ôdÅ&'';;;£ÑèÂkJ9_\,ùJ¿ YIVdOWÓÐÐÀõj¬$+¤­­Íb±0¯ÉJ²bñx¼®®NXMºè[ªÉÉÉ@ `0*++Wd%Y,Hõ¥µÙló­ÜÙÙ)+h'idÅÿÃãñB!ýj6µY7úRUT®Wd%Y±D"!5hÒø£êêê¤E8NIÜ,N $ëRMOO¬+T¢V«5)Yëêêµ»j^MIIÉ¢3pdþ¥¸1~ó~¥øó?ÿsÏÇßr°Ûíú¦RÅj§'¿ÉdBÌ«@²¾»wï¾yó¦¬ß÷Ýo§ÈËËë­·ø[.¢E²SJÒ¾¾>¯×$G%SåRê1W Y_6ÍÊÞàåO²Óï÷«K<xp||¯¦ªªy5HVKÒÝÝmµZWd5¼ÚUHÖ-HA5¯&Ñ!HVjVdohhÈívÍfJU$+É¥B&ÉëõÓHVÎE¨KÂuuu%ý(y<£ÑØØØÈ¼$+ÉÅI¦Íf©GÕeÌkjj´U×«q8ÉJ²"-ãããR­ª»ñx¼¸¸8I£¼¼Ü`0ÔÖÖ2¯ÉJ²"]¢Á`P¿dhhè÷ÿ÷m6>qd%YêêêÖÖVíîÌÌ­ªR°JÙJÿÉkÖªª*ÕD"ÅÅÅkÖ¬±X,ô¬È:Î*eë?ÿó?L¦-[¶Øívýul$+2söìÙuëÖ½öÚkEEE®Ä*¬È:	°Åb9vìØÐÐÃdE&&&Á`[iÓ!@²"ápØn·L¦ÎÎNzHVdoff¦¶¶Öh4z½Þh4JÉì¹)U9	0¬XP(/É:<<LoÉìMLLTVVýÙö$+²¡Í«éíí¥7dÅRKU£ÑXVV6ßIDKKK0¬««ãRq@²b^ápXJÕùöÇb1ÃQZZ*ázðàA³Ù¬?;?dÅ¯577çççû|¾¯W#µlMMv7J¸Ñ@²â×$½^¯Äj(Zx°üÔb±$Î°ªªJêWºHVüJkk«Édr:CCC®<11aµZÖ××K$Ó@²æºX,æóùC0Lÿ¬ú¬ápX_ÅJ*wwwÓ@²æ4ÉBÅ"1Ù××ÑÛÛÛåQjÓøøxEE×ëeÂ+¬¹+HÔÕÕÆ@  ekÏ 5kqq±Éd­¬¬'¤WdÍQÃÃÃCB±­­ÞdÍÞÌÌL½djII	×«d]H$âñxFã¢ój$+ÉºÎÎÎ§Ó900@oÉJ²forr²¼¼Ü`0ÔÖÖ2ÎHVuIºººl6×«d¥juuµÑhôûýt¬$kö¢Ñ¨Ëå2L­­­Vd]P($Z\<>>ÎÇHVuI¥ªÏçc^¬$ësÐÞÞn6ív$áÓ$+É½D"QVVf0ªªª¬$+Éº$ápXêTÅÂEÜd%YZªÖÖÖªy5Ù]¯@²¬¿6<<ìt:Õ¼>»@²¬KR__/¥ª×ëe°¬$ëÄb±ÒÒRÕºººLçÕÄãñ@ `µZ<ÏÐÐz Ys:Y;;;-ÝnïïïÏô±#NLLH$755IÄrV Ys7Y#ªRtfw½VTTèÔ××'-¬¹U³.¥ÄôûýI»Qçæs$+#²!åiGG~ÉÀÀÛí¦gd%Y³ÑÚÚêt:'''µ%åååµµµô¬$kÁ Ífklllii)))ñù|Ù²¬øµp8]]-áJ¬ÉJ²HV@²¬¬$+d%Y$+ÉÉÉJ²HVd¬KqùòåmÛ¶ååå¹@².ÉæÍÕû;qâÄ-[HVÉúÜ¬[·Nn÷¤°Ûí>¿%dÍÀ+WöïßO²HÖçàþýû~¿ÿÁì¬3|OÝ½÷î¾û¦¦¦ Yª¿¿×®]ÓÓÓÌº¬ÏAQQAd¬)@²¬¬¬$+d%Y Y YIVÉJ²HVd¬$+d%Y Y Y³òÁüöoÿöæËn·ÿáþáf|Ïf³ýñÿ1ý ÈgC>!ôò¿fÑÊýÑÉ?ý°7&iÅ'ë­[·þÏJVQQñ;¿ó;ÿ³þñÿÑ`0|úé§t²aÃ¿ú«¿¢?û³?Ûºu+ý ¼ÿþû¯½öý üË¿ül:jkkÃùå/933³²u¥Õj¥ññqù÷øÏÿüOºB»Dqss3ý TUUíÞ½~Pººº$YéåÞ½²éèïï_Îod%YIVd%YIVd%YIVd%YIV¬$+ÉJ²¬$+HVd%YIVd]®:::<ý ü÷ÿ·Ãáø¯ÿú/ºBÙ¹sç/ùKúAùè£þöoÿ~PÎ?¿eËúA¹ÿ¾l:þýßÿd W¬¬¬9hjjÊ #KFFF¬Vk^^ÞÖ­[/^¼s65kÖÌÙE¹`zzº¨¨H»ú©È©ÏIRo|yÛ¶mò»»é$õÛ¤Y)ÛõêêêÚ·o~ßï?vì49²gÏíéé9»hÕt:úM@ê§"w>'©½±yófu"Ö'N¨a;¹ó!Iíß¤vÈJÙ¬/ü½zzôK>*Çë¿åx<¾sçÎùºhÕÛ½÷Í7õÔOEî|NRCoÝºu9õ!IíßÌ÷ñXþÛõoß»víÊËËs»Ý7nÜ%ÒÖ~ªoçòòò+W®Ì×E9B¿±HýTäÚçdÎdÉþýûsðCTÁ³Iýx,ÿmÉú2Ü½×årICPÖ®]]!ß@wìØ±@å`²¦~*rísºé¼ÿ¾ßïðàA~HæüËÛ¤YÛõ%Qÿo¼ñÆãÇÍîÉvöÃ'|òóÿ|.ÊÁdMýTäÚç$iÓ)ÈûöMMMåæd¾ã9»Iê±!Y_ Í7ßºuKm)víÚ%½÷=zTr+_És°OÞ|óÍH$²@å`²¦~*rís¢ïþþ~ù$LOOçì$io0Û¤d]ÛõºråÓéÌËËóx<òW6;ÔmãÆkÖ¬±Z­/_ÎÍÚ]¿¯r0YS?¹ö9Ñ÷FQQQÒ$èmHj²®mÉÉÉÉHVHVHV@²@²@²@²Àóû76¶oßîÕÕÕuüøqYòí·ß.°~IIÉÂÏùôéSYíÃ?Tw<x wÛÚÚèmdVóçÏÏ´»zõªÜª+éSðìÙ³Ú§Ó)ÑuæÌYáí·ßÖ|ñÅ²äÞ½ÚCev5õ§O¦ÿXm=z¤/+#Ü^¹rE5<y¢RPX­vûöm¹çÎ¹Ý¹s§Z¨¿ªªbkkkH«×êàÝ»wKûë¯¿ö#Gø[$+°LMM©Æõë×%áîÞ½+·£££çÎF"HJV«Õ*©Ê×õë×K*)I·nÝªÖcÇÉk×®%U½òZß¿_ÿHV`õP¥ªäëÉ'×®]«êKIAµ÷Þ½IÉªÕ êÒjï±*poÞ¼ùìûÃ´ú>,%t¥­ÂøË/¿%*¥qêÔ)þÉ¬ÓÓÓ;qâF©8%í¤!Ufj²ÊÝK.©º~ú©,¼qãÆÆ÷ìÙ#ý?~üøwÞQ;ÕcËËËBZ¿@²«ÊÙ³gµ!Eê8ë#G¤±ÿ~¬çÎSkJ|ÊOÕ~cuT;Èªößºu+)8¥Û.hÅ®ªÚp'Uòò'HV`UQé÷ûUøÅb1R¶JãöíÛZªÖQÇeívûYjjJX>úT%mµ¾V§Ö¬êGHV`PZ/_¾,·_õxöÙ¦M´»DBÕ®®®Ô§RÃ<yÔ¬ÉäÀÿ­ÁPSS£Û¶mÆÉ'×¯_/Ái³Ù´u´¨òS¹»wï^- õ1¹yóæ9_ Y vüÎÌÌHûâÅ[¶lQõ¨,dU'Õ§v¨UfRõèÕ«WåVVS»|¥`] ¼©YXýÔè$ý£o¾ùFÄãñgßÏ¨ñx<*õwïÝ»§JjÉ*+K$3­YGGGHed°bh' |ôè$âÖ­[ûûûÔéÓ§U+(ëoÚ´Éçó©%	ùÑRõøñãê²~RÍZSS£N7Á_ Y³eËcÇiwÕ©>ùämÉýû÷ÊVµ3ùèÑ£¦ÉÉÉHVHVHVHV@²@²@²¬¬¬¬ +ÿ?è1/`©±Å¾IEND®B`


反趋势正态 Q-Q 图


½Êöì±ÊwÞy³Þ<þÒ[6@_6«¸råSùÁ`VÀ¬7dV÷ï¿ÿ>fÌÚë¬7oö½-¾ÁY»y?ë+ìTpLLLEEÅ¼yó¼nÂÁ¬YÛE8=jo/]ºäþtÕªUîsÅ0+Ïºé¬2'''++káÂ4fÅ¬ÝGB8qâ5kªªªä×¸¸¸úúz ·5  àõ×_w"¾wÚ`ÖÒª´ÚÔÔäÔdggÓ2½Ú¬6á»ï¾ëÔ:tÈë¹rõ¶ º~ýzwMcccDD-Ð«Íj7Þ¨8eÊ»gòäÉ³fÍ²[qjjj0ëÍæàÁkÖ¬Ù¶m;¡*nÚ´É=YKKKxx8ÿ½×¬vãÍêÕ«M±Ï<ó¯w»4nðþýû=OPPÐ¸qãvîÜéþèìÙ³.0«#KÍ¨ÑPÓÓÓIwïÞm-Z´hÚ´iî,YÊ¿Ü)ÐÿîF³®[·Î¹ªjÂSNÍû÷0ýD1«¢¢BeËÍ1ÃýQee¥r°»æÿùðòË/ßmf-((P¨ªä*ËÚ[½N8QM*×*Ôjß¤ïôòCÆÌÌLmÀë×¯¯ªªhèãfU`üìçfÖ¢¢"¯iºú¹ÈÈHÍVÖÖÖØØX÷GÒª§¿þõ¯þxê©§îª¿¨BêñãÇÝ5ÚöÙgVnnnPSµWÒ±¿üÊÿÜzõ¿Ó<ölZú¸Y¥±cÇéÕR¦[U(//Wýùóç;9Ï   ¿e8eÊU¦¤¤h¹ª©õáÙg½«2kccc\W¥Tºxñb¶u¸£ÉÎÎöê§pýïë8²¤¤dîÜ¹z=räÊ¬gÎÑ>Ý%Õ"cuuµ^­û¢ÒöíÛõöÄ­ÜìÛ[´×Yððp¯ýKFFY¿n;«&×&%%ªÝÜôf³VVVºkêêêtx¿råJå×é¶>Y½ú(u`Ö'OvrnQQQ­­­v6Xåö&kOºw¡YÕÎÎøK.õx<î¾999iiivxÓ¦M:ò½ÒÒR#ê¸Ð©PutèÖ­Gí ïuïÞ½Ç?~HHÈo¾©½þúë]:[^^®^uÐêu6Ø­Ì:eÊÌê Hª=ü ûbê;TÙÜÜìÔlÛ¶Mêå?z9ò¥6éÌÌL¥RmÒ:Ôv«ý×d^<@2«^ëêêôúùçßHf­©©õ_´gÏgzÕRÀ$WÌê¦©©É:¿+ÂyMìjz'^ýï>ùä½zM£½ÝÝ¡/õÔ©Sz­¬¬¼³2RDÏ²|ùòüü|¯(áîr	pG Çððp÷7G	ec¾iVEI]ZYf>|ø²eËôP^^^XX¨ÊË/cÖÛµ3GÝ§ËäÚ'Ò2p'¢ÃDm½R«««SRR-ZD³@ß4«3ÊÊîX[[[r0ëí¢´´ÔãñH¨Û¶m+**®««£YàeÉ%:4hÐèÑ£µmÓ Ð7Íj|úé§^Cð;ã	3"ÿmGN=ö´iÓtÈ¿oß>à0ë¥K¤Ò^zÉÞ^¼xQoW­ZeaVÀ¬âÊ+ÚØ²eÇã3gÎ6¤ÕÜÜ***TÖR1ëm¤®®.''')))--mñâÅÜÿÐ«3«óë~ìù§~zí6Y©ªª())9xðà;RRRfÎI³ô^³;wnrJ¨ï¼óÌª·lµ¸¸³ÞFUËÊÊ·MMM§ººè¥fµ>ÀN¯¥©S§zÚÒÒYoæQ¯Ó¿tªè¥fÝµk×6§±*¼ºÏºzõjf½-477GGGuîÜ¹K,¡qz£Y´juÂ	×n+Õ´´´+744TUU:oz©YN<)³:#ýbÖÞC]]bë?ýÓ?=ùäC			Ñë´iÓ: ÷Õî^Ýºu«óÌsw¦uëÖaÖÛ¢jBBÇãÉÎÎ.++knn=vzz:-ÐÍjZ-//¿Újºú´sÌz³Ù·o_\ûj«Êr-	èufUúD½æèeÖ+W®`ÖÛËúõë¿÷ÙgÑ8½È¬/_A[[[5ÇéÓ§;=U?öl§Ã°=`³Þ.MSSS½*U³cÇ weV'ºGb¢SoÃÎý.]ºÔ©Y¹re\àfsüøñ¹sçêõÈ#4`Ö;ÌÚuuuRiFFÆÂ333=Omm-Í7«ÈËËÓä½mÛ60+fí;455?ýôÓ/¿üòÿügn6ÉÉÉî1I6mÚ$¹r¦0+fí#477ggg'$$åçç/_¾fÇñãÇµ½yÿ^UUEãfÅ¬¹sç¦¥¥É¯ööàÁ+=àæQ[[ëÛo.//oÍ54`VÌÚPzM­]9räHw·&¤©©IGoJ®î'5³bÖ>öqv^®¬¬,::zùòåÅÅÅÉÉÉ´Ü.Ìéêëë§Mæ_5fÅ¬w*©©©kÖ¬QhµÀ­¬Daa¡êâââ"""òóóë³ÞñlÛ¶M»¶_ÿú×)))¯/NJJjjjúÚçq­³gÏVU½ûT@·Q`õêÊY1k_@Ê1bDPP«ûÉ'AÇÿäOªFÒ;vìý÷ß_VV&DGG×ÕÕÑtÚ%66ö·¿ýmIIÇãY¾|ùêÕ«û÷ï?`Àß¶~Ï=÷86]²dÉÄi4Ìí²cÇaÃþîw¿YåÑ¨¨¨÷ß_-***--t322lâiüóöÛo?üðÃÒçÌ3³²²LÍ ¯¼òÊÒ¥KUv&V´eÐÌ!ZÙé¾$~òÉ'ÉÉÉòèÐ¡C®bÅÕÓb:bÇN]¿~BBÂêÕ«U£äúÔSO4hðàÁùùù&Mêß¿ÿ«¯¾J`V¸3gÎLII©ªª:rä:`ÀToccc'Lý»ßýA0+fí¥¥¥iiifÐ×_=''ç»ßýnHHÈ¡CÜµAs`Vè26SÞ½÷ñãÇW®Oã`Vè2ë_~i8qb\ÜðáÃß|óMZ³BwPB0a2kssó"""FÁ-­º:dÈ¨¨(W95==ýøñãiiiöY¡ËÔÖÖÊ£*üå/Ù°aCIIIYYYAAÁâÅiÌ]¦¡¡!::ZZMIIINN.**ÊÉÉ	Z°`Y¡;deeÝÿý3fÌ°Ç~­2""bèÐ¡õõõ4f.ÓÔÔ4pàÀØØX¥ÕÔÔÔ¸¸¸Ý»w«ã`Vè2vWkmm­Òê¢EÞzë­5kÖüìg?ãR+fn±nÝºiÓ¦-P¯J±ï½÷-Y¡;H¢R©õZRÍÏÏå®VÌÝd÷îÝñññÑÑÑvWkVVVCCCjj*Có`Vèë×¯ÏÉÉùºíéIII¡¡¡zôÑG/³Bw¨««SZåW$Tå×¯Ûé:pàÀ³Bwxê©§¤ÒÿüÏÿTù7¿ùÍ1cFÌ¥VÌÝáølzÏ=÷þýßÿý¯~õ«ÐÐÐ'|²¹¹öÀ¬Ð5ªªªì	&89uìØ±'N,,,¤0+tÙT	õÁ´þÀµµµ³gÏ_¾|yJJíY¡;±uÀ¹¹¹²lHHHNNÎ°aÃ¢££i¾Pµxñâ¬¬¬ììì¥KÚðÑY¡gxáÆ;|øð¢¢¢ãÇ«æ§?ý©,[YYIãôISRRt,¥?ñúõë'N¨2ÝÖ0+fí1dÓààà¼¼<í[KJJ"""Þzë­3gÒ8¹sçfff:9UÉ!£0+fíIYÓÓÓï¹ç°°°ÄÄÄ?üðü£r-Ó'GkkkÝ5J®YYY´fCÕùQTµ®Âiii*ÿûß§eúªY÷íÛç®Ù´ifÀ¬µ'Ù±cG`` 3úÒÚµkCCCzè!:¶ôIæÎëuª_GTÀ¬µ'Ù½÷¨Q£SGÔ¿ÿ°°°àà`	ú			«kmm­ÒjJJ=0+fíIÖ¯_·bÅûî»¯¢¢âÐ¡CÅÅÅ		±'á¤¥¥ÙØÂÐ78~ü¸Ì,§¢UÌYeI´ººzÇÑÑÑÚí*¹8pÈ!555Ë/×§´`ÖÞhÖýû÷k'4nÜ¸;wvò#ÌzSIOOWHýÿùµ¿bëÃ?õÚk¯½øâ£Gnii(À¬½Ñ¬ÙÙÙÚw«°lÙ²3ftüQßþö·åÝÐ£,Y²dÀ¡¡¡ÎIà'|233sØ°a?úÑ>þøcÕÓPpwòàöj³FFF^½zUÖÖÖØØØ>úë_ÿ:Ä%Ú[Hxxx¯6«Ôè·ÜñG¾ÙÔ××ßÿý,--é¥úõërèÐ!mOMMMÊ¯jll¤¡³Á½Î¬N988¸aÖ[@sssZZZTTÔ/¾2tèÐèèèGyDÆ5jÔSO=UVVF+fíufÕ»µµÕNùªÜÉ0ë-cÉ%RiÿþýcbbF9útmUóçÏ§³ö:³æææ« ×ìììN~Yo%òè<°téR§2..N5Ìµ×µ¦¦Fa(00ÐãñìÙ³ço+ÐÞGõ¶ðüóÏÛUÙtáÂ#F°'Í`VF®±víZ%ÔÁ=zØ°a£FúóÿÆxY1+tûöÅÄÄÄÅÅ?~Þ¼yßýîwõvÈ!4N/¤©©©ªªª²²òÈ#´fÅ¬½î¹G±õøñãëÖ­»ÿþûÃÂÂc¶²fÍÌÌÌðððEÑ&³ö:MG;pàÀþýûI«Ê¬ÚÓ8½ÚÚÚèèhç,RR&ÌYãþ:---???==ýÐ¡CGÙ°aC||¼vâtîUèoTRRâ®Y¿~FF-Y1kï¢±±ñ6TX´hQxxø³Ï>8wî§÷¹mÛ6wFMË`VÌÚëÈÎÎ6lØ¿þë¿z<ûöM>äÈ=ôÐÀhÞY/^ì®Y³f³bÖÞHýàÁ0vìØ!CH¨?ÿùÏ'Nuï½÷2ðÍ£©©i÷îÝìåËuVÌYï$ÒÒÒ"##KJJôªý»ï¾+¿~ë[ßÍÊÊ¢njm5ojjêè6öíÛwÝ¯¸û«°páB³bÖÞ[ªµûè£öë×oæÌyyyÉÉÉýû÷¯¬¬¤z92..®®®ÎÞ._¾Üãñtæb»uýúõ¤0+fíÕìØ±C9õ±Ç<xðïÿÙT¢Õ®Ð AôîÔÊ©ùùùK,yøá½º#é8Æºþ:cA0À$fÅ¬w</¿ü²jxx¸¢ª"výÚ¿O8Qo;s®:©:R)**Z¹revv¶ÚÙKª=¶×ù^ÆÀ¬õÎ¦¹¹YB1bDaaaRRÒj``àÌ3%WÌz#H¢Êý»wïvj¢¢¢&MäFm>gÎú(`VÌÚ×Ð&Õ¿&Yöý÷ß×Î=88XÒ¥qºMYYPÜ5¿øÅ/~×µµµjpeYÆÀ¬µ¯ÑÔÔ4pàÀï½wÕªUGY¹rå=÷Ü3xð`§¯tÒÒÒ¢¢"wMKKKHHë<0|øðhg,ÌYû O©©©ôÑÈ#µë2dÈ>òÞïÑ8Ý¦ªªJtçþêêêððp577÷Í7ßT'$$<þøã^cA|öÙgdVÌYïl?®]üý÷ßÿòË/«ýï»ï¾¤¤$¥XÅV¯wK,ÉÏÏ_´hÑ­¿l<­»UF¢µgâºïQ<ÕqLXX>µ­¤þY1ëODDÄ¤I.¦µiÓ¦àààüä'ç½±u÷îÝË/ùÎw¾£BQQ~cYY×dõõõìþóíÞÒÒÒ|HmKK«äü_üÂwììì·ß~;..N¤¦¦FGG¯³bÖ;zhXÅÅÅYAAAÊ¬òÁSO=õüàÎú-¥%QÅÆÆÊ¦*Ø½.2hxx¸3¬ *SRR¤4¯_¿~?þø+ä9EÆ®>`"ÊÄ:(ÑóòòÚûºW233½*åZ;vQÕòÌYûRÑÿøÇA©ýüÑG­]»6##cÂ	÷Þïõ[&NXXX(ÛI«*ßwßÊßJ¥ßûÞ÷_-MÊúH>ÓÄæÍõVvÔ§Cíß¿ÿðáÃ_xáN.Q¾TîØ±Cs³pì·óÒ°ZØRZ¹e³ö.=|ë[ßìT±téÒ_üâ2sðÖ ­Y³F"~WïüÙ½wRRÄ)§z<©K½$¼¹sçJ¢³gÏÖd_·1h÷ÆH!!!rê/¾¨ÉdYYpÜ¸q×]¢VRKt¯§Ö¼½.HeeeZ+M°mÛ¶ÒÒRÍôY1k_CbJzè¡ÀÀ@	õG3f$4lØ0½J3¿üå/_í5Å²·s,(½ÅÅÅ½òÊ+6T¼#ÏåååYYÞ5jÔ%KTóÙg©æÐ/Òe5Ú^?ÖîQX7]QÓ=zT­ñßü¦ã%*øj^)·7mmm~~¾~©Ö 0+fí(ç)K0 _¿~z½÷ÞÝÛbccU3räH$Ý1K;s[QQQPP ½?~êÒwY%cñøý÷ßúòË/U³eËùU+?~üøâââ_ÿú×999_· _´zõjý:QiR¶¹IíÏ=÷ÜuÍ*O»k:0+`VÌzw×ÆçþüàÑGTs:18p`È!òM/WSvf	Ïj$-eGYªKÏ°¨úÒa||¼×8pàÀ°°°Á1bÂ	CU8¶®CÙÙÙiii=ö&«ªªÒÏÿÝï~'µëgjz¥Þ+%ËÍîGJÃÜY1+üDmØ°a?üðøCÉ&$$Dª¯¯·	(|¹´q$¼iÓ¦ÉpÊÍöä5géAÛiä_|±ÿþZÿI&Í5Ká[?*88xÏ=îðáÃu ¿öÚkJäLÇQQQ|ðL9sæÌÿ÷××eªc¹jâ¤¤¤²²2YÉX_çqo³ÂßDÇ+-Z´Hyñ^xðÁgÏ­ ûýïÿ¹çz|¹ééé;vìSG)J¨¿úÕ¯´¹+búÍ¬vÁ233S¯¾,wïÞ­(JZsOIôïÿþïeë¥K~Ý6ú ~tXRR²iÓ¦æææ_þòR©ä*1ë£<þøã_·uýõ§ÐÍG¶)ðXÀ¬õ'%%eòäÉÎ@<òLóÊ+¯È¸ýúõSæYË6nÜ('­²GnçRSS5+¥d¹üèÑ£r¡T§õ4hïSKeM§íâÅuà5ú®¡y.X°@³ÒÊ=ZÓ¯]»Ö.Üjzßs¶²ûàÁÙe­Òï¨Y1+tKj±T÷uÛ%Cytüøñ2l-ÿÙ­,3gÎTÁwl£n0mÚ4%ÔqãÆÉ¦¿Vcøðá²¦ç5uRNu/ÌËËÓo´Uú´[K%WËµUUU¦IYÖîÀñ±Ò°»FÇ]êE³Âÿá¹ç0`Àc=&)þìg?Ö°°0½>ýôÓÒíÙô6¶ûA¤ÝC^|õÕWCCCm$©QÛ¬|ùôéÓ¥L­ªÜ¨uöÚþë¶+ úH	X?§¡¡ÁºJiÝOEu£	Í|Þ¼yÊ¸ú]Z±cÇ:Y1+tÇp&LxòÉ'×®]»oß>%B%È_|±¢¢â/ù>U^Ü´iÓÀí¢6¬ç¦×î=»[æÓ²$3»UËúñÜ¿ÿCÝvªÖÆ×êäQiÕ#©êâujÚ?¾hÑ"ÅÜþô§ÁÁÁÂÚÆ¤L»ÃÕý¢ÈÈH7|òÉ'úE­bº³bVè>ÛÜ¹sSÚ=¶ìòÈ#ê=*¥M<YTA¿óï¨l]íF¯»<½äm=¼Î»j¹²cLLÂ««Ü,jVR³#MØòeP«V­²/ê@1÷å_öÒH¥§§k>Z½ï~÷»~ú©|ÙA'#ówIIEs-ÔîÛILLÔô6;;»K³bVð#Ç#FDEEÉ:ýúõPüïÿþï?þø¹çäm¦JfúH2s÷<Ì¾üòËeËýð?¼çâããíiÏñD&«If@¹YÂÓQPPàî «æ¿û»¿q>59uáÂ7þØ»¥KÚ	d½*ë@¿B?ÿ¾ûî3gÜ¯5Q#Ð³bV¸¡ï½÷K0JovÙÕBjff¦Ý	ª|Pú=z´=ÞUúY±bÅ×módå¡C0@óÑv¬¼»hÑ"WeÓÔÔTë|$])jJ÷U[Åe¥XÅM÷ðÅ§BªÖGËÈÈ°ÎÌJØ6mºÁ+C+¯Ë¦úu¦OEaýFu+Øªv±³bVè&K,6m)ÖóÌ3ÏH¥r^åÑââbg£oûÛJò¥$ªJW*RÆÍÊÊúÓþ¤ÀªilÄ|ÅÜææfÙÔI·2åC=¤JPwÔ¨Q&iÍÁëL¯ÅVSæ¦ìúFÐõ»á¿nêá6¡ÂÆY1+te8·Õ$ÎïÿûAAAÏ=÷ªU«VÉ¬Ræ¤IbccåÅmÛ¶)FFFJÃªðô-éSfÒwUV*]¼x±%¿´´4§îo¼¡o6Lß)µ%]ù[ÓÈpÎØFZ¢ªþdAÕëUÚî©3´%%%¡¯_QPP u°ë»:D°^ÊzË8³ÂÆòS¢½÷ÞcbblHBåEÅÖ±cÇöïßß:4M<Yuüøñª4hPbbâ×m£;É¯ûöí31+Ë®2??__×Ge¥ÕW^yeÈ!?ùÉOþã?þÃüúàjþZºVFÑV©Ñz&×××ßxTõbõêÕÄRaa¡¨eÕÔÔØ(Z~#÷á`VÌÝÇDãåËÐÐPéíÇ?þ±L)A>ûì³QQQvNXáµ¬¬¬¶¶V(×Ê¯ú,%êëÊÛ-[Txï½÷í	©Òê#<¢-aÑ¢E2·°/«©PZZªô¬W	¸Çmêitt´*þøÇ?1B¿ëÕW_UæVµÎ7éY1kG¥À*ª>öØc£Fºï¾û~ÿûßÛ#bÂÂÂ¤ãÇ+Ê@Òâ©*³³³·mÛ&QI®o½õÄ¬ÉäfÍ$//Ïý 1þ|I´_¿~òª%jn*(&ÜÊ_í<Kõå_5kkVVV­`VÌ=|é¿^A6---88xÀ?úÑdY¼ë?üÃ?È¦÷ÞïÈ#òÃþP1T>>ºlªlØ0YJ>öeT¶Ý=zÔ&¡ÊÍ·Þ¬Y1+ÜjÎ9óÉ'Ì;WÎq>Oï¿ÿ~	RéVqó7¿ùQ]]ÝÀ§¤(%+ Ê¾999Ï#GdÛ8	³bÖ>,¨ºyóæJ¢rí¯~õ«®ÎGáXxÔ¨QsæÌ³ÓÓÓs9³Þu(­þüç?MMMMHHHNN~ã7j»1«ºº:¨¸fµhÑ"ë	Y1ëÝÇãillljjÚ½·ÅÖÒÒR®fÅ¬ÐMòòòòóó·6FÄ:Y1ë]JCCÃèÑ£srrªªªÖ¬Yì÷ã³BgijjZ´hwfÅ¬Y1+fÅ¬Y1+`VÌYù3fÅ¬Y1+`VÌY1+`VÌ³`VÌµöïßïñxÆ·sçN÷GgÏpY³^ììì-[6cÆ÷G³fÍ"³fíW¯^U¡µµ566Öý´ºyófwÍ222ø3fýAAA~Ë"11qÊ)ªLII9vìfÌzrpp°ßiÎ9ÌÙ`À¬þqwJjmmµ³Á*·÷ö¤Y³þrssËËËUÐkvv¶×Ùà'OZf2efÌzjjjbbb=Ï=P«×½÷&%%¥¥¥I®0+#EfÅ¬³bVÀ¬0+fÀ¬0+fÌY0+fÌY³bVÌY»ÅüùóØ£ÄÅÅ=ðÀp=âããi6*6ªÛµQ5v¸.÷ßoT¡¡¡Ù¬'OümOÓ¯_¿üüüßÂõÈÌÌôx<´ÃuYµjU@@ÀOúSâºL:uäÈ´ÃuY±b6ªW_¦¸.:®íÙyþÛ¿ý[KKK5ëÍ@fýÃþ@;·Þzë¡¢®Ëÿþïÿj'XUUES×^mÂ	´Ãu¹xñ¢6ª/¾ø¦¸.?ûÙÏxâ[³,ÌY1+fÅ¬³bVÌY1+fÌY1+fÅ¬³bVÌY1+fÅ¬³bVÌÚC3æOúíp]-[öôÓOÓ×åÊ+£GþòË/iëòá~ïß£®Ë¥K´Qù½ñ¼xï½÷rss1+ÀfÀ¬õbÿþý'((hÜ¸q;wî¤A=ö?^Í¬&òÛP4íÛ·´·QÑVâòåË³fÍ¯®®¦¡:@ídíPSSC[yqîÜ¹ØØØÎìÏoA»aV?dggWTTë3cÆÄHLL´k×®3fß¢é®¢Ç¬´_ß÷Ý«W¯J			4TDFF:uJ½Bh+jè°Ãùwëx~Ú³úßõ¯®Bkk«ß 		ñÛP4Ã;ï¼óÁ8ÿê´_=ÚñeèÈãÌ3*èÕBh+©S§8qÂ¯Y;ÓJ=ÞnÕAAA~Ë`ìÝ»wÎ9~¦3*RRRô¿êü«ÓVíý¯ôÑG:P*8@CuÀþýûÕPÚ¢ôªÿAÚÊÏüµ3­ÔãíYýèi7.ÎÎ¾xñ¢ß¢é¬¬¬]»v¹ÿÕi«öþ×ÊÊÊT8vìEh¨0aå)vÒ¤I´U'ÍÚVêñvÃ¬~jmmµ3*Ó gÎ5kÖÙ³gÛk(ÎùwC[uð¿æh¨ò=mÕ³v¦z¼Ý0«rssËËËUÐ«òbTWWO2åÜ¹s4M×Þ¿:må¹sç®[·NÃ§¦¦ÒP ªVRáÐ¡C6ô#mÕ³v¦z¼Ý0«jjjbbb=Ï=h#66Ö+ù6M×Þ¿:måóçÏgee)¥¤¤8qêúúz	Um¥Wi«ëµó»©o7ÌëhyOë@ß£¸¸³ô0+fÀ¬0+fÀ¬Y0+fÛñ/0aÂì6*++W¯^­¯¾úªéí!3`p_°`½½xñ¢ÞÚsU³ô)¶oßÕ$*Û8p@¯ö<,·?ÿüs§&))IêÍkcË-7oÖÓ§OwjÞÿÕ?ÞùJMMjgØ<7nÜHû`V¾ÆåËÝ±²®®N¯÷îµÂ+WÌ°MvêÔ)½=ú´^'OlîgîZ-,,ôªÍâN:uªÊ~ú©ÊË-ãoYúgÏµÂÑ£Ge¸3gÎèõðáÃ[·nU¡¹¹ÙË¬GÁÔü¦ª"é¸qã,°VTT¨æÐ¡C^©Wót,~áÂ÷<³ô,ªÊ¯ëÖ­¶|)Ú9ÞóçÏÕÉ öPh;l×<niÝøáªtU6üñÇª1«°aÃþ ïpîÜ¹yóæÉpk×®55*qÊv*(eúUowíÚk'uß~ûmU;v,&&fÆ*¸gÞÚÚúÌ3ÏØ	dûnVV¤5þ Oñùç;]ì:ë²eËT3guëÖ­6¥ô©Oí¼±]"u.²Úyà'OzS	X¯_|ñvUîNyù`V>92;;ÛäW__o,[U8uêAm».°¥ë£dÈÈHÉòêÕ«úÊ6½3« `Z÷ìÙ£×+V8Âçwâãã·ÍÍÍÎWL«¾³:ö¬u¾rå¯>É¬à.ø(((°ÂøñãUX·n]XXÄçLãÕ§zëÒ­ÉÄÄD¿ ³`V»;ñÛÒÒ¢òÎ;ÇcyTõ2«9OéÓ¹Ôj½,8p@¯ÌNù*°v o2+fèûXï$w£U«V©¦¡¡áÚ7wÔ¤¥¥ÝgwÏ?o²©cVW5ÒsW3ëáÃ;°2f;gÂË/ËãÆ«®®¾î·6nÜh1WFÔôñññöQss³>>|¸¯YW¯^m(jz¯ÌZPP`ÃMðÀ¬wcÆ©¨¨pÞÚPMÅÅÅNÍ¼b«L.//'`VÌY³`VÌY³`VÌ³`VÌ³`Vè4ÿrÕêh¶IEND®B`


Ãþ«øúë¯/_¾ýý¼¿èh×bqqñññá¯ªï>K,Ñ+S2K÷Ñæÿ2(+¦;vhÅTVV¾Â²ºwçÕQ¹:xð ¾töìYÕ¢µµ511qÝºunM÷å_zÊ;w4S«]MYì[J¦¾TQ|ëÓÜÜ¸~ýzcc£&^¾|©Û9sæD¿âÎÏÏ÷Í¸Þ¿téRñßú:==ÝÖéº=uêæ¤¦¦úðÖ­[öÄ´áM_~÷Ýwá?HVw½sôfggOà~¤EßrÛ.sçÎ]¾|¹-NooïúR?733s´¢_´î¿k×®±7,~AºsEEûåú¶H"²Í+W½,Þ!ý±éË¾¾¾Û7ü_eÅðôéS­Ô­Éü	z*«¾¼yó¦ûÖÆ½w~öìföôôØÚÙm*wïÞõÞÿÕ«WöºµPÙ8UµµµÑ<=ý 7J6?Öúúúð;+º¿r¢Õ½ª¾ônIØó÷ý=7Ë#×¯_ïòåË8vYç¢ÞÞÃiB1£éeÕÄ§~jÚR×_¼xa¯ª÷ÕðmXx®ànÛhìØkÚaG[= Æå¶ÃÃûCmÊ÷h®¬nÙÝà^ÏÜæû6Ê÷g4²JR6_Ç©+«[Ói®GSÏìþº½|ù²R5Ì]³f­½#ý­mýþÅ_¨^hii±Q=Ã6DÁ¼y»yáÂº[||üâÅ5ó«¯¾²¤®6ÌòÕ=7¤êe±,,,ÀÙ6íþucVè[ÆxÑU¥÷ms!//Ï½ÔÇß¶tÕÕÕ[¶lÑÄÁíYiëÄ¶-¬²¾mß¨ÁîwðíÐ£í'ÍéììßÖñ-H4û«]YfûµºùúÍÿ½9«Z-*Zkeí=¸öÔÕÕyÿVÍ#ÜÁ-k^SSweê]ÓÙpÐÆ¬îHdø Ö7'ÊÕ+lX344¤ùª]ZZ»¿í.·úÎÉÉ±¡¤wt®/ï¿²äÛàX/Rª/7oÞlwö½îA|eµÑ¶6ÆxÑÕçèÑ£zLÛªé÷±×ÊýÜ8O·>Ô­m1øboÝÒ·Þ4ÕK­ÞÞ^û½ØÎðñÈââbß|ýhÍ·g2ÆèùØôhÙveµWØWWm$=~ü²²bÑºLëMÐÉÎ²Û_ê"¸µ¶<öíÛç©ØzYcÍÑÖtáõÞ;|óÍ7÷Y«,!0Ýfee¹1ÆSÞ¸ð=Öw=öxçØÈ[?(55Õ¢«üØ.eïTÔE­îÝÞ`|;bm+ýû¾´á#B_YÇhuÅ^7ë÷h':Ùwí°+½ùùùv´ÒýÆ«W¯jëA¯ýõÕÎÃ²¬Ú®ðm¿wYY¾âÄq«²²Ò=ÈØ»FôRëW`¯~#Ë/·£ï¾]/ü§eÅb¬´¾s!	ùùF*6 S,£/k0´>Ù@J+ßø¶^¸p¡÷Îj­ÝSkv&5Ôã»$wq²Î=[ÕÔt#Å`K5|y¨Ãlíww¶±»yMM½ö#ÛOoÍ56m§©zMÓ°¶a¡'ìo'ï×óöèµ=w.ÕgfJ/X°`ëÖ­nÀKÁþþ~ð±Àë»·¢èvr¸³½¬â¶a/÷Ú& mw^²dÖó/«Mkäjû·çpQVPVL!ZG»³^íV«ËêêjMØþ·eUElwjëî1ÊªR&½'ÛZ·"ÖêÞÎýqã-[ÛÕRêö¯vwwÛ;"ÜãÏ7Ïw<ÕFçn^VåÐ»GÚ»ËZ/Nø¾Mï)ÍvV¯:§õÆ$â¼ewo°^m%Ì3Ç*hG1m(¬MûEØoÊ=C-¯ÛwjÃhloDÄý·oFu'&&jãÃ~~mÊhB=/ös/`ûìTg;îö:¸í®®.ßOÑvÝî´o×AAAÄ²Ú=Û^gû;¡¬ ¬B¼»øÂ[¾ÏöÍÛÓíÊjï±ARxYµê·ÕÚ¸á£a[ï»ÃfZAúé§Þó`]5|Tn#)cÌêÞÚ¡éÒÒÒ7oz¬äbog®VVVºmØ¡A÷OÒÒÒl¬f§ÎZlèo§§%ú²*rö'ï¯IAÒëcòÝ/ÑÀ~3ò.¦òòrýÒNÖp/¶i|#]Âöö*C'''+¥W¯^µå¯îw¤ûÞãmÄ·Ó¼y»CÛÊíÎñ·¬¶8¶÷BKjã]=+´¾½ûêÊ»;dh'ºÁVêÛêÞÎáVvçÛÃÁ^V5ÛU¾[·nYl¬iÏÄ>îÔWÖðÓlDn;Ãc`[Þ÷¥¸Q©÷"ácV<ß|2Ñ1«ùÙik;Â­Á¢ïªî´Ûñ®;ëWfï1Õ­m¸íãëö¿yûNb=¸ÛñP«û¡+V¬ð~¹jÕ*ßì]OöGâû]Vß·0ÛîÆ¬ ¬ZÜ)3Ç¬¶ÕE$wìÍ»7ÕVvîý0áo/«âvðÌ·â¶;@ëækz´7VF_VÌy/OáÊj[rýÇ(kÄ1«Ø4|Û%+«ðlï´½àî@£¶l¾Û±¥s@ÅÅÅ¶_×¡³³ÓFüî×ªG¼°Q~~¾oNÄëlØïÈ®rø¹ov;ü^Óhö¿yÞ;ðí< ¬ ¬´¶]3Â»&ÕTÅ)!!áÍÛx5Õ;fõ]ÛoBcVGs´VµºÛ¬5¯ïÔÐéòg?A×Ù¾óKUVØì2OPee¥nm£9¡1«;Ãveõîå¶Ìçääx_4ÝÁvÆÚÞÎÝµ¹´®¬vv^yå3##£´´ÔÆëöÞVvÞïG²÷´wGºÉÕþìÊ¾«IPVPVL	¾«ï½´^½zu´ÓpÜêÌVînDâ=h¬Xnÿj4eUË]í¦6 «««³+ND|iÄµ»Æ¡3ØP%Í÷OTV;þªûè©ª:JÍ¹÷nôeuu»`ÁwïñcýtìÚ©Ëîø®ËÜs³óí¾á'ÙAhï÷[nÌjoO²K:Øå]Yí¼°Ñ^ÃÑÆÜ^ö«qÛ=oÞL»Ê¾_]ÀÒÞ@ìûcx©gûÛpSõOêW;zq`EÔJPk=»÷ð½Á¶_ZëýÐ`0è»@:ç-:Æû,Ý¥kçÚX)ÃßÐ9ÚI7oFvnÚ´ÉÆÁ¾¢ç£'l'è¦¦¦Z´ìv¼Óm4ø6e&ÊÞ6c4äÕ³÷¹y/ÙÑÑá®·l¯¶6Æ8l2î$ëëëµYà¾TM5Ð·äãö8¼²úSq½ßõ^ÍÑûÑÞ7ùD¼h%@Yñ1¹+3DL£÷¼ßI°±¯÷j¾1,c^ibEøu!à1®«0ße¡¼ììYßÒî³z>ééé¾3ÕÔð÷~í$7¤[¸páÖ­['º?cìÓÄ´ï(¿yjqøe£|ª¿[VV6î#øÞmPV(+PV(+Ê(+Ê(+Ê(+ÊÌXÏ?·Óþ¿ÿNqq_õUÄ;¿zõJß½ÿ~ww·÷ÃÛ'çõë×?»T|ùòeï§ÿ?f<C²ãý)ÇÅñÅÞ9Z;(ëìÙ³ÍÍÍºç=FÝçîÝ»î1½÷ôh¹¾,))ÑD[[þÕ'ìãµ5­ÒÒRïÃ&8;Bß]·n=M_¸pÁî300 /zz²³³×¬YcwpjiiÑw½?Ý÷#lfgg§n'úÓ£1õ!@YÑJÖ5RëÒ»ÊöÍàà M$''/]º4ÊÁJoÙÑÑ9Â¾<ú´ÝíéÓ§úRÃ²K.=~ü8<Ã^únccãOÕôööÚu«½|ùRÓuuu/^¼Ð3g»§rôðáÃÂî59xð ¤&N:5Ñ©ÿÊL+åðð°Ö¿iiiZWVVêV_~ûí·ºÕÈ)üF_VßëæÍÊêòåËíKk¹jkÞfÚPâãã5íW¯^UUUé»7ovOU9põnél¿¨n¿ûî;Ý?Þ:äí¼OØåjB?=Sÿ°Û¨ñV»]]]cY'TV­âµ6·q¦Ç(«M[TN8¡r$&&ÚO_llÜüüüðrÔ××aÝºu¶³ôÉ'6b¶Ý¡6¶ni¤¨éï®×/Â~zSüøìÛÑÒì¢úVÙJÝÒn×qBBâª	·O2|¥ocbëeooïØeõEÔýt_^¼xáî !õØRÿåË]lîÜ¹Þ-¶ëUeZ>Â-Tww÷$~zSêømÙ²ÅÔ`0¾:vû-£³_YoÝº^VÛÆX*mú¹úrýúõneg3ÙñWWª.vçââbw:Õõë×ÝÝôÈ§NÒL;WèîÝ»»%î ¦k¦õ#ìÕg4?=Sÿ0Vmäúfä&MÛa×wÜì-«FÃV%6¼¬ö¹sçê>oF%jçá³>ö¬°°Ð»jÕ*Mh«é;vØOþîØ°yk;ÛÝ=n·4tÓj¹7EÑÿô(Ç¬ñògÊüX:­jkkkuë¾ß²´¶¶jBC´ÑöÙèÊÆ²z;µ8|ô^n	Ñ Ö÷´-:dyÎÊÊ²3oí rx·rss+**4vwÝ²-my¨ýú·ÑÿôhLýgPV`ÂìlÔxøðá÷^V§Ü0ÎÞc£².Y²ÄWVj5Q]]mo9zô¨ÝçîÝ»W¯^µ3f½WÍÑÓPcû'Þ;ØØÎörÛÖÇIÚ¹µ¾÷´Ø8¯  @­Ú·o[«k§énÚ´É[¬h~z4¦þ3(+0Á¿WÏÚÖMgggÛJÿ'Ö¹ð«D¤Uü©S§.pùòeÓÕÕe;]kmGñgæbÿþý[·nÙÐÍÞuãî MMMîÙêÕ7o÷JCCµwyZc6lØ 9úÖìÙ³5G%SÈõ¥vêíUûU/÷-ýÞ~z4¦þ3(+0£­^5ôÑÀQ«`E.âìX]]]]?Hk|ßÃÏ¦qP9¾üòKwe>ihà® Á`PÃ8oøÝMõéæÍYYY.êöøowÓ/ço;¥+**&ôÓ£ÜìâÏ ¬PV@Y ¬PV(+ ¬¿bß¾©©©i|(¿ÿû¿ÿ¯ÿú¯3¶¬ó7³råÊ.>þô§ß÷ÝL.ëþéþ'Ê+(+²(+ePVÊe¥¬ÊJY²@Y ¬@Y)+²RV(+ePVÊ ¬é^Ö´´´ðùO>ó ¬Ê:¾¶¶¶¬¬¬áBÞ9ßùó?ÿsÊ ¬ÿgÕªUÝÝÝËª¬677»/øá¸H>ùä~ÍÊú«Ï&RY333Á`WWæôùË¿üKÆ¬ÊUY'Oäääp@YßOY%11²(ë»îîííµ1kAAePÖIÕ¾ìèèÈÊÊJHHÈËËS+²r¥e¥¬PV(+ePVÊ ¬ÊJY²(+e²RVe¥¬ÊJY ¬PVÊ ¬@Y)+²(+ePVÊe²RVe¥¬ÊJY ¬@Y)+²RV(+ePVÊ ¬Êe¥¬ÊJY²@Y)+²RVe¥¬PVÊ ¬@Y)+ÊJY²(+e²RVe¥¬ÊJY ¬i(­^½:++K·'O¤¬0yû÷ïOOO?~üø½÷;¢[ÊJYÑ××tûöm7çÚµkóçÏïïï§¬0agÎ)..öÍlÙ7(+eL¦¬EEE¾Á`²RVÀdôõõÍ5ËÛÑ+W®°7²&¯®®.ìÞ½ûâÅ;wîLII	BSöÙRVÀ4ÐÞÞ^UUUXX¸~ýúÖÖÖ©üT)+²(+ePVÊe¥¬ÊJY²@Y ¬@Y)+²RV(+ePVÊ ¬ÊJY²(+e²@Y)+²RVe¥¬PVÊ ¬cHKKóæÍ@ ÝÒÒBYu|mmmYYYqqOIIICC&jkkKKK)+²oÕªUÝÝÝË<<<¬ÁÁAÔþMU¹°°_3²þê³TÖßô'a~ï÷~²(kTewÓìPÖw*ëmo°¦)+²¾SYËÊÊêëë5¡ÛÊ ¬,«ÙÖÖ®_¿NY+E(+e²@Y)+²RVe¥¬PVÊ ¬@Y)+²(+ePVÊe²RVe¥¬ÊJY ¬@Y)+²RV(+Óº¬MMMqqqCCC@YßCYÓÒÒ.ñ[ÃÃÃ@Y'fëÖ­³gÏÞ²eKG(ª¯¯×X¶¦¦²(ë(K.å8+²¾·²®²(ë(ë«W¯âFB!ßÞàûöi~WWePÖ	8úôàà`øüææfµ¯¯²(ë;±SíÝ8Ï=£¬Ê:¾K.ðíþòË/m¨ªÛ¼¼<³(kT^¿~=îM@Yßµªª²(ëä=~ü833óÔ©S[·nUYoÝºEYõi¨ª¬^½z÷³(+uÀÇ+kGGÇîÜ`cÓ¼j²(k´û~h£8p1+²¾·S)+²RV¦FYUÓM65âõ)+²N`ªé¤¤$;É¦)+²¾SYÙ ¬ÊJY²(ëäËÊ'(ëÄ>üÜÎî|`7]__¯/(+²¾?go0²òY7PVÊye[³fePÖ÷VÖ]»vyç¼zõ²(kTZ[[?a§8qBe]¾|¹ûôºº:ÍÑ¤¬ÊÕÁÞÏ<ollLNN...fo0²¾ëÞ`û ¹¾¾>M<ú²(ë;a¾pá¦ïß¿OYu2ûÕÑääd»úÒéÓ§]º»»)+²FåùóçMMMóæÍëèè°9gÏUPïÜ¹£é§OVWWëËÄÄDÝ²(ëXæÎkW7ìììT>?~¬=zÔ·¡¡!Æ¬Ê-Õ´µµõÒ¥Kjj¿°èëëSP^M×ÖÖRVeÀKçÏ·²Ú!UÅÕæ«¬wîÜÑÄ-[(+²Ï£j¨ê-«©««³s5Ï=@Y'¶OØÞuS6¢´´TÓóæÍã]7ÊÊgÝ@Y)+`õÎ;.]r§5577SVe?W7Ô«,ePÖÉ|jÚÖÖf_vwwÛ9Ã@Y'Ì®ákÍ~+eÌ²Úåø7mÚ1·%%%@Y£õòåK»Ó§#JÞB^¹r¥|½yó¦÷Ò)+²zÖRWWì¹¹téRûÖéÓ§õ¥]A"¨Á@Y'Ú/¾øÂ¦õå/Ø ¬ÑÛÛë³¦³²²8	@Y'?`]³f÷Ë`0HYu2ZZZÒzËZ\LYuÂN:¥ÚIL]]]ú²®®îc]²¦qY?~|úôéðOÛ°aW7PÖïæÍ@ !!!;;»¥¥Åû­§OÆyPVÀÌ/ëàà ·nÝºI?BIIICC&jkkKKK½ßBY1TV;ÂzçÎïÌW¯^EÿÉÉÉvÈVNKKó~KYõ~8Ý?ü0'Ì¯ÿú¯òÉ'üÓµ¬­­­îÒS*«IIIð^ép·á"NKfffAAfA»öSC?ù?),,ä×®erÓPÚuº»»'õ¥øøx7ñ>O<ÉÉÉao0`Õwu~û,¹úúúI_ÚpÁ¶7XÓ£Ým´èRVÀ)ë¦MTÓªªªP(¤ìÍ=;4BÓÑ?NYYÂlyö]fffoo¯YGLÊ	eõLÍá.Æ4ÚÛÚÚÚRSSãããÀõë×Ýè¶££#+++!!!//Oq¥¬YÖuTETÿîÞ½^ÖyaÊÞemmmµ!©&(+²¾SYúúú)+²¾·3¼eMKK[¼xñýYXXHYu2$÷øñcMuxxx×®]Y[[KYuòTÖÌÌL¶KRVe<SËËËù9eö(+`&UÔ÷å­[·4§µµ²(ë=yòDíèèð6ú·¡¬Êús^SST^^n8<qâøyZZû9ïp²(ë¨Î¦¦&7TÕCëvÝºuÞñëgFY5Ú#¬§ºé³gÏºwµº ¬Ê:².p``@¯_¿v3<HY5Ú²fffÛhU·6355ÕÝáo¾¡¬Ê:1«E´§§GÓöéªØµkePÖÉUÜÌûöQVe§²zïðõ×_SVeÌqÖæææð·åpn0²Nòý¬¶]Á©©©6mò½	²(ë¨TPóþýûöå«W¯¼ß=zô¨w_1ePV>ëÊJYÓ½¬qqqþ¹wñ|§eÌ¨²Úå÷ìÙãæÜ¹sÇ÷¹r@Y'öÆMÄÇÇØ;pV®Åikk£¬Ê:7Þ466Zb?ýôÓðîrÝ`eVSS;ª7BãÔò·¼é§¬Ê:þµ´´Ô>ðÜÊZSSã»Ïü9ÊÞeUJ»ººtÛÐÐ`_ÆÇÇ777»a«&êëë5ÿùóç@YÇòäÉôôtïTÛÜÚÚª[;)77÷Ò¥Kú²»»²(k´#×qËÚÛÛKY5Ú²vtttuumÙ²eöìÙ_~ù¥æ¤¦¦nÝºuîÜ¹þ9uÂeÕmgg§nÏ?Ï@YßCY>|¨ÛP(DYõÊÕßßoÓ*ë¼yójkkûúúêëë«««5óõë×@Y'vMßÀ:88èÞCYu¾ýö[ß%øÝõ¹R²NØ«W¯ÒuëÖÙ/_¾ÔGµoQVeÊÐÐÐÒgÏ6l8;BY-++ÓDCC¦õS)+²Fõ`ohÕÑo¿ýöÍGBYÓ¾¬+Ghºk×.µ¢¢Â.lÖ;vPVeÁÁAwÖÒªU«|ßöìePÖ¨zµtû4V^½ÏÚØØhCYu£U°.]ºôÍGEYÓ»¬Noo¯ÊzýúuÊ ¬ïZV÷êÜgÏ`jjj¢¬Ê:±¬Ö××Ðüiç0sÊÚßß¯úÑWÖ¡¡!Ê ¬ãýúµ:88¨Gf;CxÇ¿~ýzwÂ°PV¼wçÎÛ½÷#GîÝ»Ç«`YÝ¨Ô%&Î`ÂíÑ£Gyyy999555³fÍB¼,fNY§Ê#Ö®]ûìÙ3ûòÊ++#×©ìûï¿?wî6ø5²RVL9êüùó5lõÎTh:Ä359s&)))??_¿&MlÞ¼Ùm²âãSSoæ¶mÛvïÞÍ3uvvÎ3Çí®ïëë[¶lYuu5¯(+eÅ¢²^»vÍ;'j`Ä+3i§ªªÊ;§½½=##W²b9räHzzºÅµ¿¿ãÆ¹¹¹ì`ôÛÑïË7sÖ¬Y¼2 ¬S.®)))¼Î????¿¯¯×djÚ¿yy¹wÎÅ³²²xe@Y)+¦"UcV^©Ì«¯¶S¡³³3##£®®W²¤öööôôôEåææÎ3gïÞ½¼& ¬À»ºû¶ûý÷ßóR²RV(+bÛêêêöîÝæÌÎ@Y)+ÞÉ±cÇæÏ_QQ±mÛ¶ÜÜ@Y)+&éÚµkIIIÞëHçççóÊ ¬±ûöïG©µ¼9e¥¬7_?==Â(+e9<x°÷n5O·þQ²ºzõjïöööð+øe¥¬ÓÕ¹sçRRRªªª9²~ýúùóçû.¦ÿ~õ÷÷/Z´hóæÍö¾I»F0²RÖByKJJr ö#'îfddü¨ïééé)**RÂÕTVþóó:ô«÷¶.@Y)+&6`?/WcÊ7n|58ÇV§mñ§¤¤hG·²b2Î9³víZßÌÜÜÜööv^¢qªêöÒ·¶¶êËcÇñÊ²RVLÌ¼cÇÛ·oÏ3ç#^<VO«×¾K '÷þ¥ýû÷ûÎ,B|(+eÅdTWWkjg-i¤¢éõQ'Á`0###//O½¯©©áÂÑ;wîÜ¢Eìq-[¦sBÿ<â»¡øüsPVÊIÒxEAÕjT«fM¬Á÷SBzzTÙíÛ·óÛr£DA­««6B/cJJÊÞ@µ÷nß<nß¾­­^[PVÊiòòrß]µ`Øµk×îÜ¹Ó7­®®hÝ®ô²mÞ¼×²bºR	9â®Á+/Î¸rrr´!âsñâEßqÓq?~ÜûI	ùùù²bÛ½·oõèÑ£7fÕBi ¸lÙ²`0¸~ýú÷õ¾#UðÊ+Þ9Ç?ëé~ú]9s¬²~ 7oÞ			ÙÙÙ---Q~²b½ÿ9ò8ÅÅÅîËP(¤)À?ö%$ÚJPSµíííZÞªª*ï ¼µµUË¥LNô¢T÷îÍÍÍu-T¿õ8¼!e-))ihhÐDmmmiiéØß:fÕªU?ýéO/üíßþíoÿöoòÉ'jjffæýÑýò¿´o)9iii[·nUB4=oÞ¼£GN»üë¿þë¥K?ÞÍù³?û³5kÖhB¦úÙÏ~¦±æßýÝßýîïþ®^	=øòåËòhãU?EÿìýA@,[²dÉ.krròðð°&µ¦ã[?üðC[RRÒ.kBBBÄéß³aÃöc¢jjj|o±öìÙüùó§ÝÕ$JJJN<ésãÆ`0hoä÷ÎfæÞàøøx7å·8ÎwQ]]~Õþ@ 0í.¯íÂÂBï9YUUUë×¯¿ûvNNïÎEEE¡PHË¸ÿ~ÝmûöíàbÎeý,X ¡§G5å·(+ÞÅñãÇoEM»ã&Mh1Ý=õÝ¤¤¤Ë/kbíÚµÚ°ÐÀÝ®Áß0ÓÊZVVV__¯	ÝDù-ÊwÒêÕ«í%÷îÝÓN½9wîÜtA7nÜ¡Ñª;1X¦pnÛ¶íâÅê¨ðØ±cÚð^K=Vnõð'Ì¨²¶µµ¥¦¦ÆÇÇëþõë×ÿ÷ÉÅÅö-Ê÷e÷îÝyyyYYYÚnûQ?ýcQ2ÝÂÂÂÊ<x q¹ïí¼1Ì´²r¥àÃÐ577×7SZmaðâ²FKV´ê¬««ãª~1îûï¿3g÷úóÐýÌ3¼8e¥¬QyôèQ0ÌÉÉ©©©Y»vmRRëÐ§m¬EÙe%¶¨¨(??((+eVqq±êÖZjÈ2M/ã÷ÅÎ5kVFFÆæÍù@x²RÖhÙ¥|oÐTh9]å½koo¯ªªÒøO·­­­¼ £ÆïÜ¹S%%%?ÿùÏI²RÖ)Ç>÷Û7ÓUÞ»ºº:½ÎzU/^¼¨Ûð®Ã¸ì¼e55<yrÙ²e§AY)ë£5¾ïÍ$Á`C­ïQ__ß9s¼ãT_gÍÅ.wçÊ+ùùùzÒÓÓ×¯_?Z,mÄïÝã¢¸ú>z ¬õãÓàI«3«°ååå¹¹¹ìd×ÙÙ©á¦Öì÷îÐçj3EI^¼ªÕ¤¤¤Cé¯®§§G999ãª±¾ïÈF®Þü(+e*­Ú4x?¾ÖSïëc±gãÇëÅ©©©ÑHIIIFFFô#N5|ío»4yaE[r¾ãúyyyáWl¶²úñµ(+e*4¨"ªuºwùÎ;óóó£üç¶7ØûÑ÷îq½@G/ïO¡õîõõô·oßî³zõjö²RVL?uuuÞ9ýýýÂê6ÊG°kkà«êVC^ÎsÂw(555á÷Ô«§MêêêÎÎÎöövVÁ g0²RVL?÷î_Ñ§§§Oh·y(ÒkÑ¢EÞÁÆÕH7lU>µråÊÑöh+'''GMUbÉ*(+eÅ´tîÜ¹¬¬,ïKìÝJZÑ«Hi«pzwù"Jª£2iG[5ZÕË^PVÊ///oíÚµvbjgg§ÆLÊ 1hUUæ÷÷÷ïÞ½[%® W¯®®N¯dMMÍh£U²RVÌ´qÊªp¦§§ëÖUÛ·o×LïÝìÚËÑ<`__ßµk×|ï!@Y)+bèvø>]VgÇ~ÕT#àÝ&%%UTTp° ¬øúÎEÒ04ý¯rssõ­¦ªìêÕ«Ã¯#²RVÄ¢#G,[¶Ì;â,..®®®ã(½Þ¡WX²RVàh¸©¸<yòÌ3ûöJÝ³¼¼Ü73//O¿(+eþF÷î-±sçÎqjÌã³ö÷÷§¤¤0f(+e&É.q`qÕíúõë5Øãþ7nÜØ¼y3ìPVÊDf2ªkyyyFFF~~þÖsçÎ%%%mÛ¶íâÅóÁ®e¥¬@ªjôyüøñk×®ñ)úÖüùó½§óÁ®e¥¬ÀäÙÅ|3ù`W²RV`,÷îÝÛ¼yóêÕ«+**Î9ã+kø»cù`W²RV`TçÎKII©©©QS:¶mÛæ¾k½×Ñå]ÊJY±Ø¶º/<x pÞ¸qÃÍ9yò¤âzäÈÕTÓ|°+@Y)+0ªQ7nÜ¸ÿ~ïgù`W²RV`ü²F­®®öe¥¬@TÂ£>zô(p9²RV`ì0ê¡CîÝ»wîÜ¹ªª*^²RV`ò4B-**Z´hQ~~þþýûÇ¸p¼4¾¿víÍe¥¬Þ6>4²OJJZ¶lYzzºny(+e0y%%%ßkÌjÝ¶m[ ÷SÊJYDÐ××§ÑªeÕÉËË;vì/(+e0aíííê¨o¦­;wîäÅe¥¬&3fMIIñíû-**bÌÊJYLRqqñÚµkÝIÔG	¾ýÃe¥¬¢¥kaaazzºú«k×®ñ²²RVï¤½½ýØ±cW®¬`PVÊ ¬ÊJY²(+e²×®];~ü¸nù!PVÊà<xð 777''§¼¼<+++öõõñ²²RV¡ê¢Eª««ÝPuãÆ-#WPVÊ`2®]»¦Ñª·£ýýý)))|R:(+e0'O,//÷ÍÌËËkmmåÅe¥¬&ìÆÞ1ë£G8ÔÊJYLRaaáÚµkíªÂº-..®¨¨àee¥¬&Éj7þü¢¢"®ÝÊJY¼«¾¾¾ÖÖÖ^PVÊ ¬Êe¥¬ÊJY²@Y)+²RVe¥¬PV(+ePVÊ ¬ÊJY²(+e²RVe¥¬ÊJY ¬PVÊ ¬@Y§RYoÞ¼²³³[ZZ¼ßzúôiePÖñ444h¢¶¶¶´´Ôû­P(TYYÉ@Y' 99yxxXiiiÞo)«ÍÍÍÞ9¥a222ù5(ëÿJHH8-»ºº(+²/>>ÞM'&&F¼Ï'OrrrØ ¬yOJZ°`Áàà íÖôhÿd´èRVeýeeeõõõÐmIIoopoo¯Y(+²¯­­-555>>>~ÝjuÛÑÑ§¸RVeåJÊJY ¬PVÊ ¬@Y)+²(+ePVÊe¥¬ÊJY²@Y ¬²eËßú­ßÊ|¯ÒÓÓÿàþ 3öhÁÿðÿ0ÖZ¬Á_wÌ.¸þwÿä'?ÁÏÈÈX¸pa.øÿûÿï½/ø9sfrYñ¾ýÚ¯ýZUUÕ/bÌ?üÃ?ÄÅÅÕÔÔÄÚïØ±C¾÷îX[pmjÁ:kþWõW¿ñ¿ñØóñ¿ó;¿^XX¨Í©÷ûÿøÿøìÙ³[ÖÊúË_þ2Öº¿¿_ëÙùµÿ÷ÿw-xOOO¬-ø+W´àÿõ_ÿkÞØØø¿ù1¸fÛ·o­1¸à?ûÙÏV¬Xña~e¥¬²RVÊJY)+e¥¬²RVÊJY)+e¥¬²RVÊJY)+e¥¬²RVÊú,^¼øÿùcm©=¶hÑ¢û·µïîîÖÿÇüG¬-x»ü¿ÿû¿cmÁÿéþéÿøcpÍö_üâO>Áÿû¿ÿû²²2ÊÀôCY ¬PÖiäæÍ@ !!!;;»¥¥e/éÀÀ@ZZÚ>ó^ë×¯/Y²DK£¥¥»wï.]ºÔª­­-vÜt)...vþÎåéÓ§q±³à¯_¿®¬¬LLLpakkëGYpÊAIIICC&jkkKKKgêbjõåV7|æ½vÏ'N,^¼8FZ´innÖ?999v´9åþÔcdÁC¡3ömF.ø;öìÙ3<<¬¬ÚYÐ~Á)kZïè·¢ÁÁAïnYµjUww··¬á>³_Ù³gÇàR_¸pAãõZð]»víÛ·Ïý©ÇÈ+«¶-kÿÁµyÿþý»à5Ó3·¬á>_6ÄÔRkè6wîÆ5^øða0ÔjÔý©ÇÈgffhq´ø]]]±³àZo¾ùFÛÍ°Þºuë£,8e >>ÞM'&&ÆNYÃ|¦¾/^¼())yùòeL-µ¹téÒbgÁ¯^½êýSµßø'Ol/E,¸ª®®NÚÐVÅGYpÊÖ;¶£ÀÖA1RÖð/V4O>©¥ß+#÷«bó7nñ÷.ÈÇúS§¬Õ××kB·ÙÄNYÃ|æ½­­­1µÔoFöÞ½÷ÍÈÙÑzbgÁÃÿÔcç7ÞÛÛk1õß¸qcSSÓóásss?ÊSÖÚÚÚRSSãããVC±SÖðy/EZZoKýfämÙÙÙÚ_¹r¥×cdÁÃÿÔcdÁ;::²²²ôÏËËSÁ?^\l»»»?ÊSV(+Ê(+Ê(+ÊeÊàÇù/·téÒ¡P¨±±Qs¾ûî»1îo2ûØð­[·Ú/_¾Ôö(+0£t©x"ªÚÝºuK·öñXÞ?ÞÍÉÊÊRzËG=¶¹¹YwX³fóõ×_kÎóçÏÝ?ikkÓ÷ ö§Oæõ(+0Ó¼~ýÚ;¬ìììÔmGGMY`»ÛÃõåãÇu»råJéý´ZÅVWWûæ(ÀVq7^µj¦¿ýö[M×ÖÖò»(+0Ø'­Êýû÷U¸'OèöîÝ».D¿¯¬@@SëëÜ¹s5BÕ¤ÙÙÙ6`mhhÐ;wîøF½zLWñ/^xefªª¯MMM6¾TmïóçÏeucPûpÛlh;Lë½ó4SÑÕ´ÅøàÁc%ÖÄ©S§øE96mÚ¤Â8qÂÒ¨§j§	2ÃËª/¯^½f;u¿úê+ÍìêêJMM---Õ÷Á?ýôSÛlÿ¶¸¸Øi=¿²3ÊùóçÝ)Evµ¶¶V6l°²^¸pÁî©|ê»¶ßØº¬¶¸··×Nuùòe7ØÕPÕîdC^~efkdIIÅ¯§§Ç¨XjØªº1¨ÝÇËfddaç(ÙDrr²b9<<¬¥i»¿Yí[(+0CØ¡Öë×¯ëöðáÃ.x»víZ¸p¡û²¿¿ßýËj(¨§OÚiÃCCCáùdÌPV þÇÅmÞ¼Ù&,Y¢¦¦¦¹sç*éééî>îª¾«/ËÊÊ½ÌÌÌø#³	¶ã÷Ù³gniiY¼x±G5_eµæiôéµÚÙL6½uënu7Ûå«ëñfÌPV`æ³³¼ç=zTsúúúÞ¼GM^^åÐ»w÷ùóç6S5ueUq5GyèõîÝ»cT ¬¦wÂ×¯_«ÙÙÙ­­­ãþ«Ó§OÛ0WEÔý.ßêïï×·æÍ^ÖÆÆF»¢îï³nÞ¼Ù.7Áo ¬@ÌY¼xqCCûÒ.Õ´cÇ7çÅ¾a«íL®¯¯g`PV(+PV(+Ê(+Ê(+ÊeÊeQûÿWk1qLÊIEND®B`


二氧化碳分压


正态 Q-Q 图


¥xÕªv»]m%æ`Lýd½÷.×ºðH¶oß.Eê¦M¤`ÍÈÈÈüÅ/~ñáäwynn®LÑ®WUU:Õ`º$«~o«¬Ú)7wîÜ»eee$+½)RçÌc0fÎ¹k×.³Ù¬êÑ)a¥½jÕ*Uª20¦u²9sFV³gÏêuü.-G²T«¾¾¾ï½÷ÞÏ~ö³ÈÈH>úHRV¯$^yå­¼¼T)aÓ7Y%G9RWWg·Û«ªªöïß¯cö'|"í=ö¬ÀtVXXh³Ù$;%P¥rmkkà|õÕWýýýÃÂÂº»»¢££¥<U×U/JULßdUêÅµÝ®·oß)´Ô¬¥Õj]±bÅÓO?<kÖ¬êêêÚÚZ)Lå»"$$$))IbUêWb6)U1ÝUVY7=*mYg¤±÷n¢¬ d¦úúz©J;::Ö®]»dÉÜÜÜððpIÐÖÖV)då+âßûÞ§~*õ«´³²²(U1ÝõÞ½/¾ø¢¬×¯_Wív»Ëâýû÷¬Àô±`Á5T¡¯¯o^^^qq±¤©Lß·oÏùóçKJJüýýcbb,Á`¨¨¨àºª Yÿ/8/^¼(·'NPwUýª?ë³Ó¤©Äjzzú®]»ÔÆÞï~÷»999êÑ¥Klß¾Ýd2I	+A»xñbõZz$ëýk×®Í;WÖ!kV®ÏLË/UþÒ¥KÚùóçKÐJÄ¶´´¨)¯¿þºÑhL5kVHHT«tHÖ!*W-Y©Yi¨´´4##C¥ÚÙÙ©Moll/³Ù[[+¿ÈW­Z¥ÎUíîî¦÷@²>$Y©YéC2???66VjÓO?ý400pÆRjªÔ¯ò% )Lå×vjjª|EÌ=[¶ªªÉJÍàI­ùâ/>ñÄ/½ôÒ9s¾ño$$$,]ºTVùüà)))j6µ5Xµ[[[¥HºV¾Ø«uT5ëÅûûûIV`Ê(//9sæË/¿ðÜsÏõööJ^fee©#üýýwíÚ%¹«]]]Mdu+Y÷ïß¯]Õ¥fUCl9sd¦5ÂÃªU«þùÿYî¾ùæ-ÊËËkoo7Í2Eª:ëFR5--M°W$«[úúúdÙ»wïØ¦$+à¤BõññQÙ)åé7¿ùMÅòµ¯M2r[PP m.))1Lª Y=ÉLßÿþ÷¨.Ü¼ysffæo¼!wÃÃÃ%k·nÝºdÉüü|©h¥TMJJ2ÙÙÙª YAccãéª¾¢Újh~i¬ÀÔâëë#É)9*±ìïï/kFFFKKKii©Ô¯f³¹¾¾NÉúÈ]6K~ôÑGÔ¬ÀÔóíoÛÛÛûÉ'1cÆ×¿þu«Õúê«¯Î7OÖú§~ÚétÊ<«µ2ÅápPªd$+0%=ÿüó~,djPPÐìÙ³,X µ©:cUËÝèèècÇÑc YIVôÖ[oIµ.ëøo¼!©¬/9s¦Édjnn)!//R$ëhµ¿¿_Ö´M6NÖ÷î¬Àd÷ÛßþÖÇÇGªUu²Í9sd5;w®§·¾¾¾?ùÉO$Te¯*HÖ±<UKSiË:¦`RmÔfÌ!ÉõÄOÈÝçÞßß_&Î=Åêú$ëx%ëh¶;wÎl6Ë¯cYO<I²¨­­mÑ¢E²RK.Ðf³Éf	×ÅKÁªF	WÇ.$«&«¬·ûöíÆ=Ö­[G²¢³³óÍ7ßìôõõýÇüÇ3gJþð?|úé§½È/àíÛ·Sª¬sçÎU§ñÜ»w/<<ü¯ý«qÁ°jÕ*þÀøÅjttô3Ï<<þügVòuÛ¶mR¶JÖÉj.Ó)UÉ¬ò+Ø¥½oç^;À@ß¯ª0õññõ¥^õk_û×PÂR°dííí¤EÉOc­ÀÖ`à1;|ø°¬Å/¿üòÚµk+**Ü"UVIIVÛ:d­««³Ûíêàøøxíx`­½wï^¹«/CjÞ¼yê¹6É<6RJ Jµ*ñùo|ãW¿úUBBBww÷Ò¥K%MÕüR­JôÒWÀx%ëÝ»wÇü×^MòXrû IV`ÌuvvJ²FGGKy*íÔÔÔððpµcU~430¾É:Î9#+°¯¯¯Ùl>ö,É<­­­£jªäh^^«DìÌ3ýüüÔ[¶l!VI¬<6mmmãgø³ÿò/ÿ"Ù)*+kÚ¥#õë7¿ùMºdõòòZ³fÉx¾ÎÎNÅõÒK/½ñÆR¡>õÔS¬R¿ª|òI5Äüùó9ÈdÝ±c~Ê_~I²héÒ¥sæÌÛ¨¨¨àà`Y³L&ÓÓO?-ýýýgÎ)E-±<¾du:/PG8p@uùòåÚUÐËÊÊd¼$Éx®®.©DeÝ;w®4-ZTQQ¡N`õë­·ÞyÎ?Ow5YïÞ½«¿ìùþýûe-µÛíl<Ô£áÙgmjj»k×®p)±±±*P||æÏO¬¬¯%wòäÉiÜ¸qd<¦/VãÊª¤Æ4ý»¿û»5kÖØl¶ÌÌÌÞÞ^:àdUg«öñãÇ¥éÒ%ð4GÕSÖ^xAU2ÕßßåÊÒ7oÞo¾YYYI¬¬wïÞUAV0>|XW??¿+W®¬hkkSXî¹ððp__ßÙ³gKCJXNW&>YoÝºuðàAY3ÕõsøÂÒ¾qãF^^Ü9IV`ÂéÇ)¾ÇKHH»»wï¦	NÖ7o«%>¯_¿.ëç§~ª­¯¯ðûöí5ÔÇÇGÖÜ¨|UAEÿQ³IS§ÓYWW'+gOO°Òèèè@èö=HV`¢Ô××'&&ÆúúúÊ*ùÔSO¥¦¦ööö~ñÅ³gÏ7oÞ¬Y³ÊÊÊè+ÀSUVÔ¬jª«.ÉzáÂilÞ¼d&$V¥<Ýµk4¤*UWªùñ8 ¤¤D"VjÖåËsÈà)Éªv£J©ªOVE~«ceúûï¿O²¬&êµÕ7%%EjVÍ&Y»eËY+×®]b08p	ð¬UÛ&¬ÖÞ×¬[·NÚ²ÒrÖ0!Õé4V«õ'UÚ-2ÍßûÞ÷²²²$bãââdâË/¿Lw¬ð(RÎ?_~à.ðßÿýßedêêÕ«¥l5Lï½÷Þ[o½5sæL	]Æ<:Y/PWW§í=räÉ<~¿ùÍoÔA¿¢±j(`©S£££ÕÕËåVvíÚµÄ*àéÉªIZÊ¬Àã!I©eeeÝÝÝ£QQQj|%ÿíÛ·ÓKÀ¤IV§Ó)+ó3gÔÝ+W®¨HVàñ8tèT¨êråF£Q¿ýíoe,..3g¬o½õ½LdU§®º<çÛ¬À`jÑ-[¶øúúÊÉ$S,Y²xñbiÌ=&M²ªq7mÚ4dÜ¦§§¬Àø©®® *Õª­êp555Ë/Z»víO>¹zõjúÉzçÎ5Xv	tí²çüñ+Ô.sçÎé¯äJ²cRªÊº¦~¿Úl6ÕÓ§OËt	Q	Ú;w:×_]Ú4É*kõåËÕõYÕÝeË©>,wÕ©®f³Y2dÆÊ~ð³fÍdX		iiiQ&%%©!%Sýýý÷íÛG&Yí/ùKÕÞ¿¿Ü½û6[QÖ¦EEEÙÙÙÛ¶mkjjjooÿú×¿.+Wjjjç»Ý.ÉZPP k[[zVdd¤ßo~óN­&q²^½zU²´­V+G0£ÑÜÜl6¥*ýøãóòòfÌ!µ©£~ø¡~6YÝvîÜ¹uëV£Ñh±XÃÚµké@`r'«¬ÛkÖ¬Ñß'YÑxòÉ'#""æÌóÔSOÉj6x¶   /¾ø¢»»ûôéÓßÿþ÷/]ºD8YO<)ëüµk×ôÉj·ÛIV`Ä6mÚð_ÿõ_µµµ©+V¬XµjÕ¬Y³:;;õs®_¿þ'ZvÑ¢Eª¨ýì³Ïè@`'ë¡C$GÕAL/_»eeeã7Éé@ªO)CöÙÌÌLÁÕÖÖ&Õê/¼°lÙ2Ã¡ÍY__o4[ZZjjjÞzë­mÛ¶Q­8Y¯_¿>wîÜÃ¾ôÍÆÝ±ooo³Ù¥&¾úê«&ÉétÆÇÇÛl¶üüü¢Í`Ô¬[¯¼òÄj\Ô©µµµ8)44Týó÷ööVVVnß¾½¬¬L;ÉJ²C¨®®:5""BÕKgþüùF£ñ/¾ é¬÷îÝýúõ$+0âRÕápY¡$J333¿÷½ï©p=vì½L£dUG-]¸pA?ñË/¿$Y÷KUµí÷ø¬M|ðdmaa¡ÄmNNLÉÎÎ¦£©¬N§S.¸ªªJVu$~ô`ýE[IVàA¥ªD¦:¸££C¦¨Ë¬ºÌæããIwS9Yûúú´ö#GÔXW®IVLUR§JµªSR¤Zê¿ÿû¿õsªq¹é1`*'«ËoÔõY÷îÝ;NÃ¬b¤<µÛí²¾ääätww»<*Óýýýå¡ÖÖV©VeÊþðúÉºiÓ&µ¨ªª*   ((¨j´W®²L&Å¢/U5F£QíLñõõU^x~¦E²ºìLµÐ8´Ùl$+ 'å©VI~>èB4²?ÞjµJÄÎ;wð¦f²<yR¾Î9#!zñâÅÁÉ:ò¬¼ä¨:WUtúôéÕ«W/Y²DnÕuËEKKKtttoo¯þ)))´Lýdu:ª$ÉOÛ«êp8Ô^Õ]»vùøøÄÆÆfee-^¼ØÛÛ[]¢¼¹¹911Ñåét#0õæÍòM¡E,É£´´4444,,LKÇÎÎN__ßýìgÚ<òíïïß3Àd2I¾êSÙe©Y³êW-YÃÃÃcbb.)))$+¦³¶¶6Yd]ÈÍÍÕï(5$$ÄefIÖikçá444$$$äääÐÀ4JVuqÖë×¯K[¾$YûûûwìØ!÷ìÙC²bÚ***R»Jxþüù¼¼¼E¹Ì/3k×UpµZ­ÑÑÑ.»]LñdÕdRmuÅVÓP»Ú«*¥ªv®ê_|!)ëíím0Ôhû±ÚSêëëe"î$ë yyeff2¦³!U!!!R§ª4­©©ññññõõUEjIIÉ3þyz Y¹ðÿ+USSSVé½÷ÞÔÀúÇ?þQTuMÿ_~HVø?Ú^Õ!Uúîw¿k³Ù¤mhhÐ&êw¬ YIVà577'%%©1>VIf5333"""99Y]ÓÆÏÏOd%Y¿éíí-,,Ô~ÐeÉ»»»¥ýñìíí½k×.yÃáHMM]½zµ:nHVø©>ÕÀÃ¤DoLL³/¼ðëÂe=ôññõðáÃt#@²¬ÀßÆ!÷ªj***d6YñJKKÓÓÓ%V×¯_/ÕêUd%Y¿innV¥j^^Þàëªêµµµ½ûî»)))jÊöíÛå¹Rà¶··ÓÉJ²bZëííhÔf³éñ²²2©SåvöìÙC	,úÆo¤¦¦ÒÉJ²bZkjjR5??ßÍÃäUb8##ã?üa@@´×­[g0¾ýíoS°$+ÉJU£$«ÓétóYRFFFª!åöå_â' YIVL_ÍÍÍ			®tLEET¨R³ª«Ü:uJÖÝ»wÓ«ÉJ²b:Ýºu«ªÑÑÑMMMôÒgyF»páÂ   u±°³gÏf;0@²¬N>m³ÙÜ9xHÉÉÉ+V¬ïíímhhX-,,xcS0@²¬^$ù¤T5´WUïØ±cÊRòÊÔ¯®óæÍKLL|¥dÅ4"(¡8½ª8U.®Äg~~þ_Æ5ÌÈÈÕÇÇGM@²¬$GóòòT©êÎ¹ªzÍÍÍò¬èèè¤¤$£Ñöúë¯»®ä+C$+Éé¢¾¾^¢QJU÷ÏUÕtvvÍæ¢¢"µµ½½ýgEÉ2µyrrrégd%Y1õuwwKìI© ¥ç ©ÒÒÒ2kÖ,É±uëVÉìÄÄDuÙ8$+É)^ªZ­V£ÑX\<âCv%KKK]&FDDüñ,+++((¨®®f;0@²¬â$êrssÕ^U÷KÕÚÚÚüü|y¢D©Ä2E?[ggghh(g×$+ÉéÂétZ,G-U·nÝ*y¹mÛ¶¤¤¤µWY¥]KN2;ý¬$+¦>ÉQ):¥TMNNnmmuÿ«ú§dffjñ)ÍfY¦LLgó/@²¬úÔ^Uí8^÷IKµªÒÞÞ.Y«Ý(|­¨¨ÙaPHV¬TUÃ*¥¦¦¶µµ`	yyy%%%.Ë4Lô-ÓÓéR5,,¬¼¼üAótf!eeeúJWV¦Ð½HVL#Ú°JéééºÂän||¼Ùlè~Ð@Á©£v»]J^iK¬JÁ:²Q¬$+¦l©ÚÜÜ*õ¨*FeNÉËmØÙÙ©Py$eU$+(U]eddè§qÂx´RUc³ÙZZZôSZ[[åét#ø[©êp8Ü)U5ÉÉÉÚJ=Ç% Y¿Õ`±X¤T­®®vÿY£Ú¨Ò¬u9oHVLÓR533³³³óQn·Û­VkÑ´´4Æû@²bZÔðËÎÎ²©©i4¥ªÊÊÊÜÚAÂ0uõìÙ³qqq~~~6íÜ¹s$+ÖÖÖÔÔÔüüüÐÐÐ]»v¦Té¬QQQêý8p &&FÇY½z5É:Ý$%%é¯Ôö«_ýÊÛÛ[òu4¥*LdÕúë_ÿê5U«Vñ·>zzz$DÕèj¸ùaï½÷duWccãÆ¥qoNÍ:­H¦FDDHC*Ô°ÒÈÏÏ/,,¤s¬n¹ûvzzú;wØÏeÑ¢EÏ=÷ªj¯ªT±åØ±côuÚ6^u·««kÃ7nÜàØ`(ååå³gÏöññùéOÚÛÛÛÒÒÂé1HV·8Î+WÞ¼y³nð«§§§«Rõ_ÿõ_m6[`` T«¹¹¹Ã_ôHÖÿ®?Rdæ¥jXXXDD Y)cSª:jS$+ÉQ)--5LRªrdÅ¨´¶¶¦¤¤Pª YIÖqQYYo4-K^^v=©ª¤¤$44RÉJ²ªª*ýI&SõÃ¶µµ¥¦¦Rª YIÖqä2ÂAJUÉd6)U¬$ëxÑóÓzùµ··'''K©-mVN$+É:^Ôô.;V%~¦Ìg,--Ï(x=«%dwIIIÛ¶mÓî¶´´Fírß¶Wq¬$ëã£.ô¦]è[SàsiSª YIÖÇ­³³³°°ÐápHñÚÐÐ0Ù?NJJÁ`ÈËËÆªd%Y1rjX%Í6~"É¤íUÝºuëìÉJ²bÜKUöª YIVM©ªFæ`$+É1(UM&SBB¥*dÅ¨´··§¦¦öª YIVM©j³ÙN'½d%Y1rÍÍÍRª²WÉJ²bTz£££9WÉJ²b´¥jBBºXU¬$+F[ªFÎUcSªmÛ¶±WHVÊöíÛ¥T>ú4½$+F®µµU¼uëVJU Y1r½½½RªZ­VÎU£ÒÒÒB©$+ÆÆ¶mÛ-¥*¬¦¦&u0¥*¬ÞÞ^Uª&&&J¾Ò!@²bäN§ÕjU¥ªD,$+F¨§§GÒÔh4ÆÇÇSªÉQ9ú´ªùùù$+F%77×`0$$$477Ó@²bäÔÀRª³WHVvpRRUd%YGE,±ZPP@©$+É:rÚÀÉÉÉª@²¬£¢6LìUdîîîÁ ¥j[[$+É:rõõõªT-))¡Td¹Ã!¥jRRRKK$+É:rÕÕÕÅh4RªÉJ²¶Wzzz;$+É:rÍÍÍ6-,,¬¼¼Þd¹¼¼<Á@©$+É:ZµµµEJÕêêjzHVuä:;;ÕÀÒ¦Cd%YGÎét¨¬¬¤7d%YG®§§'??_ªìUdcÇEGGÍfJU YIÖQÑFÎÎÎf¯*¬$ë¨TWWKÊ^U YIÖÑêèèÈÌÌRÕápPªÉJ²T¨ê`ÎUdîîî¬¬,///JU YIÖÑªªª0ÍõõõüãÉJ²§Rªr®*¬$ë¨¬¬®­­åÿHVuäÚÚÚÒÒÒ¼¼¼¤TíîîæHVuäÊÊÊ¤TµX,ÇãßHVuT¥jrr²ª999===üÉJ²ªT5LRªr®*¬$ë¨´··§§§¼¼<öªÉJ²Jyy¹ªìUd///JU YIÖ1PUUÅ¹ª@²¬c©··D YIVHVHV@²¬dd%Y$+É YIV¦L²ÖÕÕyyy¬uôõõÅÅÅ¬ulìØ±cçÎZ²®Äb±¤¤¤ð·¬wíÚµøøøþþ~@²»Ý~êÔ©¿½!¶HÖñú¾­M!Y$ëØÄ-5+d%Y$+#E@²ò·¬$+d%Y Y YIVÉJ²HVd¬$+d%Y Y YIVÉê	6oÞ<sæÌ¨1e±X"##£àvw-Z´~pÓSO=A?¸éé§¦»ttýà~wÉ*9¶Ë4>Y¯^½úÿÆÚ?üÃ?H²þ?¸çÕW_		¡Üô£ýÈËË~pÓÏþsé®?ü®pÇÎ;¥»~ñ_Ðîøì³Ï¤»Þ|óÍ±]ì¿ýÛ¿õööNîd?ýéO/_~îÙ½÷è7>|xëÃEcc£tWWW]áÎÎNé®?ýéOt;þçþGºëÈ#çåHVd%YIVd%YIVd%YIVd%YIV$+ÉJ²¬ YIVd%YIÖGñá®]»;7íß¿ÿ¹ç£ÜtâÄèèhúÁM---Ò]===t;nÜ¸!ÝuñâEºÂýýýÒ]¿ûÝïHV&ÕÝ¼y3<<w=6..ÎÏÏÏf³;wÞÅ-[&ÝæÌ:Äuuuðå¦7nxéÐ!Ã»÷î"##N'É:äûÎjµòOé¦¨¨(5<æbbbèáI ª£¥ÓæÎK<T__ütctSUUDýà¦wÞyçý÷ßïïïXµX,$ë8Z½zõ+WXG ((NpÓñãÇ¥Ê§jÇj Â«í©ñK÷Ò¥Kó§ûÿ1kò£jllÜ¸q#ýàN,ÿ`RåÓÃ»víZ||¼¬nZ¹r¥ôÛåËéáIGíÚµKJ)XÏ?O²¬åöíÛéééwîÜ¡+ÜTWW7oÞ<úaxv»ýÔ©S¬#ÐÕÕÅFòõõ-++üß"$+ÉêYëðnÜ¸AW<êïe:á¡«!ÇãX@@0<ý¯ÛÇ³>¬¬Ænq:+W®¼yó&]á¨¨(58ÎÙ³g¥ßèÖÇ1ÿ»zõªú½Ë?ØCåää<xðþÀAû$+k²§§ªpß¹sçbccå×ñ+¨òYÇ£Õj°¤¤$FZ~¨[·nÙívµ[úÊ+$+í7"]ÉÉÉHVHVHV@²@²@²@²æ«±×²eËÒTUUíß¿_¦|þùçÃÌÿÐ+~¨ëoÙ²EÝ½sçÜU¹@²SJ]]¨¤ÝùóçåvïÞ½Ú*kjj´)V«U¢7sÀÑ£G9"3¬Y³FòÁÈ[·niO9sæL9ö¬~¦ÿjîÞ½«/+å¶±±Q5úúúTJ«Ù®]»&w¯_¿.·+V¬P].µ+ååå¹LV)®ÕÁ«W¯ö'|"í=öð·HV`*Ð.øzéÒ%I¸®®.¹½xñâñãÇ¥ÑÓÓã¬f³YS¯ÁÁÁR¡JCJÒØØXU°îÛ·O¦pÁ¥êej)~ûömý2¬ÀÔ¡JUÉ×¨úRRPmã½uëK²j5¨º®¸Úz¬hµV?óG$%t¥­Âx÷îÝ2E%±4:Ä Y©ãæÍ6m;pàF©8%í¤!Uæàd»§NWuß÷]xùòåùóç¯[·Núß»wïÅ_TÕsív»KHËrø+$+0¥ÔÔÔh©ý¬öìÆÆU²?~)ñ)ªíÆj©¶Um¾zõªKpJ,·'NÐ])UµÃTÉË Y)Eedzzº¿öövR¶JãÚµkZªæQûe-ËÑê%Õ;w®e¿<KÚj~­ ª¬À¡vµ=Vn?þøc-ðvìØ©ÝíééÑ¢bµªªjð¢nÜ¸¡îëëÔ¬ÉLõÖË+77W5âââ¤qðàÁàà`	Îmmª<*w_í5- õ15äKP³$+0-¨¿½½½Ò>yòdLLªGeº$«Ê<©>µ]­êh&U?^ne6µÉWÖaÂ Y©O¤?æèÓO?)÷¿:£&))IÅ¡~ëî­[·ÔDIS-Y%qeÄó£Ö¬/^&À¤¡@x÷î]IÄØØX§ÓùÐg>|X¹2dddJJz¨§§G		¬û÷ïW(Êü.5knn®n¿@²ÓNLLÌ¾û´»j¨¦wÞyGrûöm²UmLÞ»w/)@²@²@²¬¬¬ddd$+$+$+$+ÿF¿§üxIEND®B`


ôÐ¡CÏÂZ½y$+$+ Y Y YÉÉÉÉHVHVHV@²@²@²@²Çýû÷Õ%»ýuÊÊúøãWùÑ£Gòè7nÝº%÷î%òÒ?^õ©2åË/ë¯U¾¯ï Yç³²>øàýùuÞ·¢ü;.íí1?ýôÓµ¦#ãLNNjÓÔ)w?®¿ëóù¤1<<,ÏêììTñ¶4<¨lÎ+äÑwßW½i÷õõ©qåîÌÌÌÎ;8 FÐBèÊ+ò¨þÕÃ^Brëõz×ûê±Hýw¬ÀúÈ¬ò+1©ÿÉÖÊ'OT#//o÷îÝ1[2)¾æèèèöêîùóçÕhrWÊ²»wïFÆ°Þìì¬<zæÌ(oU¹û¶Yn%Æ>|(í¶¶¶ÈFmLsÉtçÎïm|þùçò&¥qîÜ¹õ¾z,Rÿ$+°ZR>úT~óóóå×üðáÃr+w¿üòK¹Ê)ò±'kX5>>.±ºgÏuWe¹ªúõ¤WÕlÈÎÎ¶~"=òûýòhuuµöV¥Smë±LS;µ]Tn¿þúk¹½téÊ!niËayyYÿµ¸Z×«Ç"õß!@²ë633£¶"J=$?»7oÞ^³®+Yå'^~ÍU]%í(ÉªÚ*T:;;%96mÚ¤¶|ªU[TT§NÒÏÂ»ï¾«6Þ»wOUÌjs¨*£UnI¥(íÓ§Oë7½®ºÖõê1þÕHñw¬Àú?²ßUK'NPÇÝ¹s'ì'[¢n÷mÓqNN«4´m?úª&VyyûöíèÉ¢Ú«Á´¤¤þ'@)]¾|Yë×¯GæzjÓ«$ÓÚLÝºu+WÏZMJ½Cd6Ðï½§T·Ûùs¬m·½fUÂõo¾LVInUcI¢HÑ&¯+w+++µ2KÍ¤ö¿ji±mÛ6I5rii©v8Õµk×´ÑdÊçÎêX¡ÉÉÉUsKh;5µ¶¼ZÁË«Ç"õß!@²ë&Õªª­Ó$mµÛ5Á­ÁúdjX%Dld²ªSD¶lÙ"ã<[Ù(úuiiÉëõªÄÝ¿¿4¤ÌöG¤¶|ª÷¯íVGÞªÂNmî~nnI©µ%ËõQû«ÇX³¾ÄwÈÇ$+°Q$êä§öäÉr«/L¬>/HCJ´µ¶ë©êJÕ²2uhqdõ^®"DÚ°)HH«Rñìt:Õ·j§rdny<©ÝµÜRÿ0äd¿<7öWEê¿CdÖMð"5ª¿øâ¤'«SZ§Î±dÝµkWX²JQ+m»Ý^SS£Nùê«¯Ô8W¯^UGÌêW"oC2FbC=E?ªíÔVnõïA¦£vIªckÃÎiQuÞ¾û$«?®­çªÃt=ªO¬X^=©ÿXççU÷k«µwîÜ©~Ä%Ï"UÎEöZ°*ù?wî_ßåËÕ7oª½ZÖªÅï¼óö7nÜøæoTé¦ÎºÑFgÏÕÞ­LYòãÙwÛEÅéÓ§ÕYu§Ê#GÈyhóæÍ2DLjïS:¤6ÌJöKziÉëªØ[×«Ç"õß!@²ë°ÖÏ«>R8ÊO°Üª#¨ummm1¾üâõ¤eyäÑ4ZBHr|øáZÏ|ª Ò6''GëA¸Ýn)ãôÁ¯íÑ|w:Z¨«éËp5Ô|±¼µQº¢¢b]¯ãßÉÉHVHVHVHVÉzüøñ×_=ðÆoüû¿ÿû«¬ñ±wïÞ¤ßÿýßÿúë¯_ùdýñüRÀ~ô#d¬$+d%Y Y YIVÉJ²@²@²¬d¬$+$+É YIVÉJ²ðJ'ëäääîÝ»srrvîÜ9<<L²HÖH öööJCÞe^^jùÉO~B²HÖõéëës¹~ûmÖjÞzë-Ö%dÉòòò-[$>;;;ån(ÂOúSjVÉº>¯½öûY$kÒäää¬5!Û·oÆµk×öíÛG²HÖïÜ¹SªÕ½÷.,,¬"$+ÉÉÉJ²HVd¬$+d%Y[ZZ'YIV@LLLx<²²2d$ª¹¹Ùh4z½ÞééidÄoffF577·¡¡aiiB²â×ÑÑa±XÇÆÆX$+ ~óóóeeeYYY555¡PB²âwñâE«Õêp8FFFX$+ ~RVWW)XgggY $+ ~CCCN§SªÕþþ~ÉßÒÒÒ±cÇrssKKKgffX $+ ~###Çh4¶µµ±4HV@B¥jSSªEEEWC²2==-¥ªÉdjnn¦¶¶6£Ñèr¹Á KdÄo~~¾¸¸877·¾¾. HV@Bººº,Íf£dDKÕÁPSSÃ5VIVÒßß¯º¥A²¬P©*Ejnn.½¬$+$*ÚívÉÔÞÞÎÒ YIVHH]]ªÓÓÓ,døMMMº YIVHHkk«Ùl.((à¼dÌÎÎK©ZWWG$+É	éîîRÕjµÉJ²@üæææ*++¥T-//ç¼dHêr¹,]@¬$+$ª®®NªR¶²4HVâ755åñxrss[[[Y$+É	ijj2¬ccc,døMOO«ój$d%Y !ê¼·ÛY$+Éñ÷ûýYYYµµµtA²¬Íf·ÛzzX$+É	©©©1eeeWC²¬`0¨ºàÒª$+Éjhh0^¯wff¥A²¬¿©©)	TÕÆÆFÎ«!YIVHH[[Åbq»Ý,døÍÎÎgee544Pª¬$+$¤§§ÇjµÚív.­J²¬¹¹9UªVUUÍÏÏ³@HVâ×ßßoYA$+É		BÁçóÑÉJ²@BÓé4­­­¬D²¬?ÉÑºº:)U½^ïôô4d%Y ~Á`PJUUÎ«!YIVHTss³ÑhäÒª$+É)**RV¥T%YIVHHkk«Éd¢d$ª>. HV. HÖéÚµk»víÊÉÉq¹ãã$+WÖDyy9]@¬/ÇöíÛÕûëììÜ±c4þ6Ûíöz½¬K)Në¢©©¥A²¦Í7Ë­;B^^É i]@q^É*FGG9ÂÖ`¯ÕDnnncc#çÕ¬©âÁ>ïáÃ$+WK½êbhh¥A²¦÷î>|xaac¼BT¡¶¶6±@HÖTIÖ@ °oß¾ÅÅEÎºðÑº¸xñ"KdM­dÍÏÏÏÒ!Y¤~©ªº¨¬¬ä¼" !===t K©*«­,døõ÷÷ÛívÉÔÞÞÎÒÉñ[ZZª¯¯§¬CCCn·;77·¡¡. @²@B%SÆÆÆX Y ~^¯×`0ÔÕÕQªd´··ÍfÃA©¢uQSS3??fUxKKKSSS?+d%Y¼/^´Ûí6-ýz+lhh*¼ªªª¶¶Vf°¼¼MÜ$+É`Iy*ac0ä6ÍJUÑÝÝmµZ¥fÕf¶  @²õN²¬6D à±X,éÚDEEEKKKXuîñxXõ$+É Éª««¥Tõz½iÜ[aIIIØöí©©)§ÓÉd%Y$ÓÄÄÃáÈÍÍmmmMï9­­­õûýú!MMM>ÏÉJ²H£ÑXXX¨íLc³³³EëCª§§Çjµð1 YIVI0==]PPi]@Aù!ùj]ÁõïHV@r´¶¶L¦í"qvd(^¯7-»ÉJ²xÑÔÅ´ì$+ÉàBiÜHVÀ588èp8,ìd%Y$DÊÓªªª´ï$+ÉàEs¹ÐHVÀÆZZZª¯¯LÍ. @²¬6ÐÔÔªÄª+HÉJ²HHss³Ñht:ÙHV@ÒÌÎÎdeeeTo YIV¢§§Çb±Øl6Î«ÉJ²H§~¿ß`0IÙÊÉJ²ßÐÐÃá0Í,¬$+;v. @²¬`bb¢   77@²¬ÕØØh4Ýnw0|tnn®ººZrWF¨¬¬dÏ+HVÀfff|>Á`X«T@-))Üõûý6mjjEd®««Ëb±Øíö@ °Ö8Çóz½úÐp-++céd%YüJT]¯Fb2ú¥U¥¢íîîÖÊÕår±A²¬~ellÌápL¦þþþç,å©¶ú!###n·ÅdðP(TWWg4¥å)­­­úº¶´´´¦¦	d2]0t:«ë½´jII«T®===ÅÅÅR°Fß¬$+þÔy51ªzKKK¥+êëëU¬$+Ñ&&&¼^¯ÄjCC]@d%Y$¤££Ãd29Î¡¡!HV@üæççKKK³²²*++)UéÉ*ß?üP5vïÞ-ááaËUQQqáÂüüü]»v¬¢èîî¶Ùl#ójHÖãÇ«ÛíFoo¯´ïÜ¹#mùÂFÀZ¥jyy¹Á`ðù|tðõÙèè¨§TÕïÜ>ZnïÞ½«5//di]]@¬O>U¥êÖ­[U²¾ûî»2äÂúd&ùý~ÉÔ8Î«Ò6Yµ­Á'NÑhÜ³g¬R¿J#''çí·ß&Yh´. Ö:¯ÍÂ YW¯YÙ êÃa­. äÑ³Ùl³Ùä¶ººAÍú«U¥ìÂÂÉ@#Qêñx¢µ¤¤¤¨¨H%®Üúý~HÖ_&ë3gÔIil ´··Kêp8ÆÆÆVA[,O333ò«ÂadV²^½zUmø@ü»¶Éd:zôhgg§Èpª(]øvuuE^Ö(9Ò0Y<y¢òõÒ¥KËËËÚtå+ôÎ;ï¬ä@þmÛíöècy<ý¥¥%ÍÆÃÈ¬d£R¤J[þZJûìÙ³rIV IyZYYi0¤`åj3£ÀÍÍÍÚúúúbBf%ë½÷$D%;µ!ããã2äÂÒUÛµúd2üÏ4Íííí±G£ü#"µ¸¸¸®®ÎëõJjjJÖ'OFÖ¯¨ÚÛ·o«*d2ähmmmnn®DãÌÌLnGGGCCD2×UE&Ö¬ÞØØÓé4LÍÍÍlÅÉJ²HF£ÑårAHÖõÊ+çÏ'Y555UTTd0ª««C¡$kjÖ¬¬¬Ï>ûLkïÛ·¯|:©¯¯o]S[\ÌÏÏ'YWBKKÉdr:ku¬ëÐÛÛëóùº»»µ~/]º$·©Z²Ç>ÍááaùÊ³HV ÅÍÌÌ¨GVVVÎÍÍ­÷é³³³µµµÅÅÅeeeííí,O¬¿ºLK¾W7nÜÐ×¯ï½÷þ®ÖS,öïßëÖ-²þ0Ùlöz½¬Kà%êêê²X,V«õâÅq<ddDî÷ûåémmm6MR¥õVMÖ;vèkV­×um^ÖÚDp»Ý$+ð²ÌÏÏË·[¾¤¥¥¥qW£x<ýj2Øõ×Ézýúu§Ó)_³»wïÊÝsçÎÅ]³F&+[Ô188¨.­*f"Ù,kØi9UUUMMM,a¬ÏÔUqÿþû÷ïÆãÇ¥`÷ÝwµËÉ¬À+-UWW¯×;11`ÕkµZÃµ¦¦dÉú+7oÞÛååå­[·¾ÿþûa-..Æw°1É¤`0XPP ¥jkkkR&èr¹ôU¯ÊZ.kõÙ£G<+ÊW#5kmm­ÚVL²¯(©,%SG»5ÍRþJlss¬¿òðáÃ°D ¥§ =HìIæººº¤÷â+9íóùN§üh;vÞA²þúÄ+Ô­¬6mê^!ímÛ¶¬À+ª­­Íd2Y­ÖðâU»þùZ5kXQK²©Ouë÷ûãè YãOVU­ªSWµ³WåOnØè;MIV ¥´··[,³ÙÜÓÓÃÒ^t²>~ükÝiCÊSÕÜRª/'Yõ[åÛ¨róðáC¹ÛÖÖF²¯þþ~ËJU %uxxX¢ôÚµkúdÝ¸KË¬@B¡Áàóùâî­ Y¹ÞÞÞÒÒÒîîî3gÎ¨Î¤ýå_JûäÉ$+Á Óé4LÉê Y¬ªBÔv»>xð@HÐR³©¬¾¾Þh4ÆXªþÝßýüc®¨¨dé¬'N½pá´·lÙ"Ï?ÿ¡ÖwÐO²©`jjª¨¨(77·¹¹9.þã?þCêZÿþà¶oßþ¿ñò5d1¬O<yûí·%>ïÞ½«6m(>sæÉ¤ÖÖV)UNgìWm³Ûí?øÁB¡ºúôi	W¹eaÉOVµXn/_¾¬îªúU>k×g%Y ºoe]]ÝºzüÞ÷¾wãÆý)^ß|óM)äd½sçN^^þ"¬«Ö¬qd®§§Çb±8õî%LÍÎÎXQQñ¿÷,Uàwl°¬Ô¬@ª	BªÊÊÊø:ÖÜöûü`ÿþý,[àE$+5+RÓéjõâÅqOäOþäO~ó7SëÜÜÜ[o½óÍ7ß°xjV ,--ÕÕÕ%«ïïÙÙÙR¿nÝºuhh%¼´urròéÓ§$+ð"mPR§Ò¥0ðõÌ3Ú5YÃjÖêêj¹&Y£¹¹y]]@H­d]^^à<uêTrSDéºº¢5+WRº´jAAAì]@H¹d=°Â·¢û;ª­ºæ6Él(íÒªëí@Ê%ëòòrØF`ùn8qX¯ééiù?ÚÓÓ³ÞCÔ¥Uãè@*&ëªG3¬ÀzÕÔÔH:Íæ/?®]Z5î. ¬$+ÒP]]ÓéUwëÈÈHôg%ÞTLÖ§OJ=z42Y<yB²Ï%ÖñBUUÕ±cÇ¢±ªååå]¤gÍªOSiL&uj¬@tò5	ÒÚÚZYY¹êÈGJÕööv)ÉÊÖ``]Ünw¿~£cÊ@£ÑX\LÉJ²kêèè°ÙlÁ`PÝbÔjµj»]õ¥jnn.çÕ$+É<_ss³Édìt:v»=l·k[[ªCK_$+Éô$(§Ü&^GB¡±±1ihçççý~¿Á`¨ªªâ¼dý%ù¹!Yæææ¤Äú²¼¼Üµbzz:¹/Ñßß/Ó·Ùlaa¤m²ªcÝn·v<°Ö>uêÜÍÉÉ!Y~ËÊÊ´Rµ¾¾^R0Ye¥Vªr^YÉúøñczäGfµX,a9*ekRúT¥ªÉdâ¼ ãkÝ cHÍ6PêË³P*`ÕçÕ$+ÉÌ277g6õÛi%#é]`0XPP ±ÚÐÐÀy5ÉúëC8@²"øýþ¢¢"®¡P¨²²RîÆÍÍÍF£Ñãñ<·»`¬|ò~È£GHV¤%	QÏg2-KqqqXß1)))Rµ¾¾RÈôdo¯PGwvvJ²îÙ³G»z[[$Yñªhmmt:v±ë"i*_¸÷vuu©K«Rª$ë¯Ö_öüÌ3yyy¥¥¥lÆ+ªººZ2õâÅSSS±f³¹££c^k~~¾¼¼K«$ës®%wåÊù/¯©ÃzëU×IÕ÷,2e»Ý.Õjww7K Y×ÜÃ*T»¯¯OÚ7nÜ Yñinn5l`AAÁØØXrKÕªª*Î«HÖçlÍËËS0?^×[·n¬xUU2/l ÛíNb²J©êp8¤¦THÖÕÝ¿ÿìÙ³[·nUC. zýúui/,,ÔÔÔÈÝM6É$+RÜÈÈÅbÙ ­ÁªÜÜÜ¢¢¢¤w/ MuqqqË-ªÃ`0(ñy÷î]ÉÑ¯¾úJ?Úòò25+^G0%¥AÕÄjSSçÕ$ësHÊÿúÉTùk/%¬4ä_¿ªD¯´O<I²"ÉÇµ£££¡¡¡»»[b¯¥¥ÅëõJ¾KÄ&>uáUËÅy5ÉëK.]RÉªv©J¸ªá¬×¯_Æï½G²"5Z,²²²ºº:)+Ýnw/,#-ñl0¤Þ¤a²ªÝ¨ò¡OV¥­­M,Ã?ýôS©Y­Z­VýÁD~¿¿´´4)ÉÍf»ÝN©¬qnVgÝZqðàAioÝº³nÊ:::¤ZÕQýì'¸+T]ZU¾rKÉÊµnAkkkÃÚl¶DN3U¦@Éd½~ýúÀÀ¶ÿµ··dE*ð+((ÐW¨Á`0µ¦¦Æ`0H_×üHÖhÝ0ICKY©IÔívWVVª£$V=OCCCçºJUdMf²IÓááau÷Ö­[êà&©ìêÕ«òøþ÷¿ÿÆoH.666Æ1	c£Ñ(!M©¬IKVuêjØ1Àq¾Éd/..¶ÙleeeRªæççËÿÂõNdjjÊëõÚÚZÎ«HÖ$÷|ôèÑUãÖçó¬HA¥¥¥©Ú.ÕÖÖV«Õº®CyÛÚÚ¤Ì¥INÖªÎ"´K k=ÿâ/öîÝ«v¾ë¯äJ²âåPËÑû2§«K«RªøÅFôÁtóæMuVuw÷îÝê¡óçÏË]uª«TÁ$+RTaëêêêëëûÜù<SªØ¨dÚ>ø@µÏ9#w<xÀÖ`¤`Íj6ÃjÖâââè5«ÖDYY¥*¬·oßÖl#m§ÓÉLHM«îgÒi°êÂh4¶´´p½/(Y%J8 ¿ëv»IV¤ éééÿüçø¨,¡¡¡µÆW]@x½^.­àÅ%ë+W$JïÜ¹£OV)HV¤`0(øìvaa¡Édúó?ÿóù¨`Õ¥*¬çÎU1Ý¼ySî¶µµÅ×Óøø¸ÕjÍÉÉÙ¹s§¤5É$OvùñIÙcÇ­5¾êRÀMÖ»wïæåå?>òÒ7Gc>ïôéÓÒ8yòäÁ¥ñÿ"üô§?%YÉÔòòrýU;V]@Pªxi5kIN«Ú÷É'ùùùß~ûmÖjÞzë-Ö%Ö«ªªªµµ5l`äÅmT2<°Ð¼òÉÖðüqhhh¨®®Ö	» «êBþºUVVriU/?Y¥Ê¤wß7dggkíM6±IFc¿ºTaê®êBJUi°¬¤D²ª£®_¿®øèÑ£uMäµ×^V9-m±ý"3«RJCbUòU.u4SZ`Ã5hÝwwwËÉd¾÷`ýE[cqèÐ¡S§NICn×êÍdVtJ@Íf)4å6ÊQ¾aÏ|íèèVªER¶««Eà%'ëòò²ÖîííUÞºu+_ýõììlù¥»víÉ(JJJÔ)1n·Ûï÷¯+UÅÅÅ@J$kØoÔõY¥ÖÜ îIVè]¯fffÆh4ÆCCCN§SÆommå¼)¬G4r¡»»Ó¦M7oî^!íûö¬Ø]]]eeeaªäh]]ê­PmÔJÖ°©öZ.dÅF¢ÓãñE¦ÍfÞ_D©ª¹¹¹'¶@J$ë+W$G%D'''#u#:å'Y¡rT>iÍÍÍÚ©D¢lÚ­¯¯72ÎØØ@*&k P%©4HV¼xf³¹¸¸X2µ°°ÐápLLL¬:æÌÌLQQÁ`1Ù« EuqqqvvVXI1??_]]-!É$Åås;maCCCGGÇZ½&µ¶¶ÊÔ$w)U¤zÍª/^µdÍÏÏß±cÇ2Ðëõ¬T§´´Tí+íêê4ÓLçææTo555ôVàUJVuqÖ»wïJÛjµJ²>úôO>'O$Y£¶¶6IVýÖÚ/Ê'*¾©õ÷÷«õe",[¯X²êÉïàöíÛU[]µdE¤²lii	(¨õö8*++¬ª^ùd:U~Î6ôz8$kºª­­mhhÐúÕl6¯+ÇÆÆN§T«ííí,Ré¬/É®%õWK­¯¯/,,ZadÍP¥®Çkmm---êsjj*'AÇCHV5#ô÷÷×ÖÖVWWÇÒIïÐÐ$«ßïonnq«LÖh4Ju»Öù¬@²¬éC2Òf³566¶´´x½ÞP(¬ÏÌÌÈéÉJ²f©&vý	¿âââ¤L¼½½Ýb±ÐdÍ eeemmmú!@Àív'8YuÍsº@²¬ÇçóuÔ011ár¹f¿Ýn§$+É;vÕºººÈëªÆH»´*]@ YIÖ%ùçp8ü~ÿÜÜäbss³Õjï^uiUÅòÜnúd%Y_qÖ;;;+%¦ªÉdòx<CCCq¼´º´*]@ YIÖ4ÑÝÝ-¥§Íf3ÍR>¾°VV¥$+É>úûû%PÕáBKKKMMME]èm£©K«ÒdM+§££C?¤ºº:Yç¤F)U>ªõõõtd%YÓTaÙ&UlqqñVÉR;Nº@²¬iÈn·ußÖÖ÷3ÑÍÏÏ«K«Ò[!dM[UUU¥¥¥ZÎÍÍÍI5Ö³RRÍ&A>88Èb@²¬iKêHÏI>¿ßÜR¦VSS+¥0]@ YIÖ47;;+É'Eª¯ÕÕÕIï¨!ºÝn£ÑØÔÔÄ`$+É¶Ti6Õi¬I¯SÕKHTÎ«@²¬é¯¼¼¼¨¨H·:33S\Ü£FFFçÕ YIÖ ¤ÕjÕïòB2$Y½/IJ¦J²r^dÍ]]]ªT±a]FÄA¿êÒªqôB$+ÉúJ,,,XTTà%QÛÛÛÕÛþþ~2dÍ RMZ,	BmT«2dvv6¾	ÎÍÍUTT¹å¼$+ÉÆÆÆ¤¸,..®««[«ÕßàTºòt	æîîn,dÍ©ÚÐÐ Å«qLaiiéØ±c¹¹¹%%%/æò8@²¬)!µ´´øýþÚÚÚ@ iªójF£Lój¬$kq8ÅÅÅRbZ­V©S VªºÝnº@²¬§¤¤¤ººZ»;55%áw·øcccRªªëÕp°dÍ8R_Z,°¬¯¯¯­­cjmmm&Éét°l¬$k&5l`kk«ßï_×t´. ¤ü¥T@²¬M5ì¨¥æææØ§ÐÓÓ#¯ÍfKúpd%Y_=R¡J(ªÓUC¡Pmm­Ãá±îÑTeeeq÷#$+Éá*E§¯f³Y»ÄÍsHËSïOHV5IÑc/ùÚy5^¯wffEd%YñLLLÅØ]@É5©N%)bÙ®ÛÜÜ,¥ªÇã¡ YnjjÊb±hi$ºÄ\êëë)UdÅ*ª««ëêêôCZ[[%>W-UF£Ãáû7@²¬é¯¤¤$¬BÂTâS?d~~¾´´4++«ªª. dE4~¿¿©©I?¤§§§¨¨H×ºb­MÄ@²¬øµÁÁA³ÙÕÝééi)XÛÚÚ~±Òë¡©AVº±jmm5L%%%>Oêúq¸v»]îÊ£," Y±>RöôôtwwKÍªuQXXÈy5@²"!SSSF£Q*WÎ«	illLdÕv»HV¬O¿ßïóÍ7û·;;;»®®.Æ~$+V)Rm6ÛáÃ¿ÿýïÍfÉÄ©5@²"N¯½öÚ;ï¼c0*++çææº»»­V+»WdE<$M·lÙ"¥ªª6Ðår±p ­uqq1??dÝ8óóóYYYv»=¬Ç322Â"ôIÖááa§Ó)?úÚÓÞ|óM¯×ËºÏàà ÍfRõoÿöo­VëÜÜödªÑh¤g`H«dÝ¿ÿ­[·´dýöÛo+BNNÎ[o½Åº¥¥¥ºººÜÜ_233óÂn·ûâÅSSS­­­´---,(H«dýÕ»ÉÊbkprI=ZPP ±viUR	Z§ÓY\,ËÏ'õ¨dª§¬Yß!Y.®Zª¨YIÖukjj2LH$+É¨ÒÒRYë$+=E$D]oÕf³=÷¤þþ~úd]ÅÜÜÏç¥T]ZZ*//·Z­eeeEEEehh$+~M*TÃa6ÛÛÛ;ruuµª¾R¹JÊ²ÝHVüR(ª­­5%%%ªçÖçÆÉ5ÍI©j4b|ÊÜÜÍfXWWW__Ïò5s©Þ%S=ÏÄÄÄºk2¦¦¦ôC¼^/5+¬kzzZÕ`0ÔÔÔÄÑ<K®m:nnnë@²f¶¶6³ÙÈß´z×n·»õV½5MJÕ¬¬,):?	u~~~hhH2dÍÄdíêêRÕápòQd§RªÏÎÎò9d_ °Ùl&IjV>Á@²¬ñ[ZZª©©ÉÍÍ-..;I@²¬ë.U].Á`hiiá# YIÖÔ××K©ày5dýå¥U²²²;ÆÅÝd%Y¢.­ày5CCCÊUUUÍÍÍHÖMV)U½^¯ª~¿?8lll´X,¬Ò2AËE@²f¶··KZ­ÖþþþD¦#ÕªLGqeeeEE Y3%Y¥<"UJÕ²²²Ä·Ü[VK`ó¹5#UJLÃa2:::2Áªªª°F£Ï=¬é¬Á`Ð`0%±·BÕÂÂBýööö>÷@²fDÍzñâÅävÜnwyyùôô´ÜRØjµÊ«ð¹>â¤vÜÚl6£Ñ(ÕjOOËHV@²¬dd%Y$+É YIVHVHV@²¬¬¬$+d%Y$+ÉÉÉJ²HV5Ý577;N£Ñ(·, YIÖøÕÔÔH I;®@²¬ñ6Í³³³Ú	)^çççùÉJ²®[OOOYYYØ@Ç322Â§HVuÝ.^¼X\6Ðétñ)d]·ùùy£ÑØßß¯éêê²Z­KKK|Êd%Yã,[Íf³ßïomm­¬¬¶:	@²¬qnhhX­¯¯bÉJ²HVd¬$+d%Y Y YIVÉJ²@²@²¬5¹®]»¶k×®Ë5>>N²HÖlß¾]½¿ÎÎÎ;v¬5i6oÞ,·#Øív¯×Ëº¬ë0::zäÈ@²&Á|>ßÃÙ Y×-ë;êî½÷>¼°°À±Á5Q@`ß¾u Y ???Kd¬ô YIVHVHV@²¬¬¬$+d%Y$+ÉÉJ²HV@²¬¬¬qyï½÷~ë·~kRÙíömÛ¶mÏH¿ó;¿c³Ù¶g*wY9ïòO~fÎ&å÷w¯|r§i4_ùd½ûö?'ÛýÑÉ×ì3RuuuVVÖ_|óþå_Ê¼ÿÕ_ýUf®zIÿøÇ9ïüÇüÆodæ¼×ÔÔÈÇþÿñ3pÞÿéþIæý/ÿò/;Ùý×]ZZzµu#üÍßüÍ=2sÞä£ö_ÿõ_8ï?yïëëËÌUïñxþú¯ÿ:3çý>øáóäcÿÿù8ïÿû¿ÿ+óÞÛÛûb^d%YIVd%YIVd%YIVd%YIVd%YIVd%YIVd%YIVd%Y×ãïÿþïÿôOÿ43çxxØápü÷ÿwÎûÿüÏÿÈ¼_½z53WýýÙ566fæ¼777fæ¼ÿÛ¿ý|ì<xóþôéS÷ÿüç$+¯5vm×®]999.k||ÐÂÂBNúÍûäääîÝ»eÞwîÜ9<<¬HÕjU]¹r%£æ=í×»¢Vöë=úì§ýª2i¿ê£ÌûYï¬Û·oW<vvvîØ±CÿPww÷áÃÓxÞå+¤%§Èçó>Z'O<xð`FÍÚ¯w±¼¼,(#DÒ~½Gý´_õQf0íWy1ë=s·oÞ¼YWõ; ûåêëë]?DÂæéÓ§ÒxòäI~~~FÍ&¬÷O>ùäøñãÑ!ë­ÙOûUeÓ~ÕG÷³Þ34YGGG9VÎîÛ·/''Çívß¼y3]ÿ¼oÙ²E~b¤d×¹^µ	óöëýÎ;2kò3-°Þ£Ì~Ú¯ú(3ö«>Ê¼¿õÉúàÁÏ÷ðáÃU½wï^XYf^í5ýììl­½iÓ¦÷´_ï¥¥¥ê´ÝÈhÉõeö3ç+9ó²r7t½gÊÒ<|øðÂÂBqÒû£ù/UÂæÉ'ÏV6­<é:ïi¿Þ³þ¯L[ïQf?£¾òa3Q_ù(+wãÖf%k Ø·oßâââª[nß¾­¢WÆIËÍ#ÏVÁC:uJr+Õ|FÍÚ¯wÆIûõö3á+¿ÖfÂW~­y1ë=³5???ò¬j:N)he§ß¼ïÜ¹SfpïÞ½ZÉ®æxxøõ×_ÏÎÎ¶Z­=5ïi¿ÞWYïÑg?íWýª3!«>Ê¼¿õNO¬¬¬ddd$+$+$+$+ Y Y$ïkµ÷nßîîî3gÎÈ¯¿þ:Êø'ú4ÕÕÂßÿu÷áÃr·­­¥¬@º(]!!*i÷Í7ßÈ­º(>/]º¤q:½å+.ÐÛÛ+#8p@òÙgÉû÷ïkO!ÚPÔ4Ï?ÏòHV Ý<~üX_VA¹UååeÀj´;wîÈÝ»wïÊíÞ½ÕÀ°«ËC555aC$Ukuðþýû¥ýå_JûäÉ¬dÒvÙ7nHÂÝ»wOn'''ûúú¤ÂÕjµJaªòuË-R¡JCJÒ;wªõôéÓ2äúõëaU¯LSKñè§	dÒ*U%_Ï=»iÓ&U_Jªm¼÷ïßKV­UW~V[UëÖ­gßí¦Õ|âÄ	(¡+mÆþ¹QI,sçÎ±"HGëììTÑ(§¤4¤ÊLV¹õêÕüü|µQ÷ã?7oÞ|ýõ×<(ýÄ<yòöÛo«Èê¹¥¥¥a!-Óa-$+V.]º¤R¤ö³<yRGQÉÚ××§ÆøGÕvcµTÛÉª¶ß¾;,8¥ÛË/kÅ®ªÚáNªäe$+VTFú|>~333*%,¥lÆ;w´T£öËÚíö+Ô1Jª'aùôéSy´ÕøZAY³ª¬@P»Z¯]»&·_|ñx|òÉ¶mÛ´»¡PHÕîîîÈI-,,¨Ã#ã YøÞfeUWW«Æ®]»¤qöìÙ-[¶HpÚl6mmª<*w:¤¤>&·oß¾êKP³$+Ôß¥¥%i_¹reÇªá¬*ó¤úÔvµª£T=úÍ7ßÈ­¦6ùJÁ%¼©YHêè$ý1G_õöÝ5*õ[wïß¿¯JjÉ*+C$×[³NNNFIedðÊÐ: |üø±$âÎ;ÀsuþüyUæJ"ÊøÛ¶móz½ê¡P($mÝº52YÏ9£:PñÃjÖêêjÕÝk Y³cÇÓ§OkwUWMô6äÁae«Ú|êÔ)Sddd$+$+$+$+ Y Y YÉÉÉHVHVHVHVÿéÛ<yócY»IEND®B`


反趋势正态 Q-Q 图


e#ÏÎ^?»ûvss³,ùí·ß.u=²ÌÀÀqæ%-¿s.gÝn·LtvvÊ_566ê/rË´Läææ¯ÖæuÛK.=xð ÞnmmÕe¦§§åìððpjjêt#Br©ùÖ-7¡3ûúúä4++k¹·õÊ,<Éç¤ù)Û<|Tóóó:»k×® [rUrýz===É^zöæÍºØää¤aÙÝ»w_¾|éa³ÑÑQ¹ôêÕ«îªÑåT2öúõk®­­Ú¼y³±¤ä<::úÅY^Æ6¹páÜI¸qãÆro=ëÿX£òü ÏæEEEr*g/_¾,§2ròýÃàËj`õööJV÷ìÙ£gµåTöÒëL½ÒÈÈH6_É7oåÒÒÒRã®Ê`N'½ÇrÆÚé~Q9øð¡Þ¹sG;dî±ÌwØÈÕ²n=ëÿX¶ááaÝ(ã!yÚ<f]VYå)^ÍuÓÊªÓÆÆF)GTTîù´Ä@GÀ:ÀÍÈÈð-G]]y<¨;KÇÇÇuÄ¬»Cu­Ý¢L×××w½úÝËºõ _j¬óPV`ùÿd-=V!zñâå)[R·ËËØul³Ù$®2aìôÒ×1±ördd$pY-5nÝÙÙYcR~ Aºwï'OøvKï§îz2íñ2Vêùóç+¸õÖÕ=(+ð=zÔTËåûtlì·~Ìª,eüø±oY¥Ü:Æ¢È MnWÎ:tÈféÑLúþ«QíÛ·K]táãpªîînc1¹æ7nÈL=Vh``Ào·ñ¦¦Ñ0Ð­áÎ`n=ëÿX6­êÈõ­÷&Ö·]W¹7Øk9$±¾eÕlÙ²Eyë+QpF!ÌcÖ¬¬,-î¾ûdB¹2âÄ	Ýó©÷ßxoX¼Õîî~g·diLKËÍ)þÖ³~ÄÈ?PVàÔÉSmuuµ¦k[V·ÛíñxdBhKí6ÓÑeåôÐbßÑsAA&DµkHëJI4ÏC¼Õ7»VXX(cw£[úC^yHûåo¿õ`¬ÿPV`Ùô3é¨ñÒ¥Kk^VNÃ8ýuçÎ²Ê V¦ÊÊÊôC#W®eîß¿¯GÌ¯2Gî4F²¡b^@Çvº[_=Èõè[zl­å3-:ÎËÌÌV9sF­¿ÕÃt9b.V0·õÊ,óß«éÙÖNMMÕ'qéïôõõiç|¿µÀ/y¿qãFkkë½÷tÎÐÐîì5Z«;?ÿüs#öO>üø±ÝôS7ÆâÚµkÆ½k~¼ýy¿¨¨¯¯×wdõSÚÃË¹hÓ¦M2GJ&!³ÆýÔCtÇ¬´_êe·«Ù[Ö­cýßC²Ë°ÔÓ«dà(OÁ9¿èuµµµAÞ<ã[¾!Èh¹ïÑ4F!¤_ýµñÍ|: ¡­Íf3¾A¸ÆÃo¼£)êííu8FÔõúe¾.&c¾`î¿î.,,­ù²cßC²@Ye²@Y ¬²¾Ã3gâããx?þðÿð_þå_Â¨¬û·»wïÞ!Þ?û³?øðaxõ/ÿò/àýøÍo~CY ¬@Y)+²RV(+ePVÊ ¬ÊJY²(+ePVÊe¥¬ÊJY`éëëôèL°5(+eUiiis:»wïihh`PVÊ+488(Y5jêñx$®mmmlÊJY`%JKK+**ÌsjjjöïßÏ¡¬V";;Û2BQlJJ[²RVXâââªª*ó[·nedd°e(+ehooéëëÓ³Ï=kmm-[²RVX¡Í7ggg»ÝîèèèÊÊJ¶	e¥¬°*£££M^2fekPVÊ ¬@Y)+²(+ePVÊ ¬°)koo¯Ýn·Ùl©©©ÿã.DFFÊÉÉIóLÊ ¬Vn·»¾¾^&ª««sssý.#È¥2ÑÔÔTTTd¾hÔÇ_|AYá[ÖØØØÅÅEOHHð]@b¹wï^¬677ýøãþ|úé§<ð0-«Ífó;mÈÉÉéééÑéäääÌÌLYÌårÉÇ>þê¯þ1+ |Ëªo ª¨¨(Ë¥Ï?ß³gï_;NÞgPV«mÛ¶ÍÏÏëÞ`¶âÄsçÎùýCßSVe_WW'rêv»-Ê½ïëë3Î&''è533²(«Uggg|||dd¤Ýnïîî6>ocLõø&ÕÓÓãp8l6[zzºÄ²(+ß ¬Êe¥¬ÊJY²@Y)+²RVe¥¬PVÊ ¬@Y)+²RV(+ePVÊ ¬ÊJY²(+e²@Y)+²RVe¥¬PVÊ ¬@Y)+²(+ePVÊ ¬ÊJY²(+e²RVe¥¬ÊJY ¬@Y)+²RVe¥¬PVÊº/^¬ªªzðàÿ²RÖU9~üxLLLIIIYYÝnþ'e¥¬+qýúu©i¿s:ÒZþ'e¥¬+QXXxñâEó´´4þ'e¥¬+-)5Ït8üOÊJYW¢¼¼¼¸¸Ø<§ªªÊívó?(+e]ÑÑÑ¸¸¸ÊÊJ=j©¡¡!::º««ÿ	@Y)ëIGÓÒÒâ¼RRRÚÛÛùo²®ÖÛ(+ePVÊ ¬ÊJY²(+e²RVe¥¬ÊJY²@Y)+²RVe¥¬DYív»ÍfKMMíèè0_499axaÊ ¬?q»Ýõõõ2Q]]k¾¨©©©¨¨(È)+²þ$66vqqQ&æççÌIV/ü·>GVV< LËj³ÙüNäääÌÌLér¹ü.ü©?ø? ¬ð-kdd¤1åwññq§ÓäÂìuY·mÛ6??¯;xez©Å´£Á,LYa]Öüüüºº:S·ÛmÙ<22¢cÖÌÌÌÀSVeýIggg|||dd¤ÝnïîîþÿwÎûÃa³ÙÒÓÓ%®K-LYoPVÊe²RVe¥¬ÊJY ¬@Y)+²RV(+ePVÊ ¬@Y)+²(+ePVÊe¥¬ÊJY²@YØe½víZDDÄÂÂePÖ5(kBBÂöíÛý^´¸¸HYuy;¶iÓ¦£GºMêêêd,[^^NYu$»víâVe]³²îÝ»²(ëõÍ7^MMM½ÁgÎùCCC@YáæÍóóó¾ó¥¬£££@YWE	ÖOãÌÌÌPVe·»wïæxYvýõ×:TÓôôtÞgPÖ ÌÍÍ½óÈ&Ê ¬kFÊZ\LYuå^¾||ãÆcÇIY?~LYuUd¨*Y½ÿ>gPV~ëWÖ^Æ±ÁJ§¯^½*W¦¬Êì¾_ËÚHJÏ=Ë@Y×ì`Ê ¬õQÖÅÅE©é#G|Ëê÷û)+².c*ÓÑÑÑzNSVe]UYÙ ¬ÊJY²(ëÊËÊ/(ëò~üv¹ñÀÆt]]µÙl@Y×àÇÏÙ ¬üÖ²B¯¬ ¬ëßØØXUUUqqññãÇ=zÄõ[ÖS§Nç¼yó²®7v»½   ¦¦¦¬¬,&&¦¶¶Í¿¬ç3/=¸±±QÊºgÏã'ÐåùZæÈMRÖuÅétÊÕ8ÛÕÕ=88È¹¹9óo_½z5666''½ÁëÙ³gÏRRRfffÌ3:tñâE6|ä²úþÇèè¨LLNNRÖõIF¨iiilXGeðÒéÖÖV~úô)e]&&&6oÞÜßßoÌñ«Ãá¸uëÖEYçææ¤£±±±úíK7oÞ4âj³Ù?NY×¦¤¤´µµýà=);;;##Ã²ø/þÛaZÖW¯^]»vmëÖ­===:çöíÛÔ'OÈôäädYY%)ëºÒÐÐàt:?ùä¤¤¤ÒÒRÈ°tuu¹8/ÃáñxØ&¯²NOOoÙ²E¿ÝPÆ=Ï/_JG¯b^laa1+w VVVêîyñ-­eË ¼Æ¬Bj*¯+ïÞ½+M!¬LÈÿ	ª¤W¦«««)+w*///..6Ï©ªªr»Ýl]Y¥wîÜÑ²ê[ªW/eòäL=z²,;;»¥¥Å<gppÐáp°e^eÕ·Qe¨j.«ª­­Õceþ·ß~KYVXXXSScÓÞÞîû	1 ôÇ¬Æ>aýÔM¾Wnn®LoÝºOÝÒõë×SRRFGGõ¬¼dÏÈÈ8~ü8[aZV~ëÀê:t(11ñôéÓçÏw¹ÙÙ|uôööÚívÍÚÑÑa¾¨»»çÎrÓéÅô=&k©Wmm-YE¸õÉ'wïÞ5kjnn^Ùõ¸Ýîúúz¨®®ÎÍÍ5_¬w½±±qÇ2ÑÔÔTTTd^Æwÿæoþ²6^YÍ£F0*»±±ú-ÿóóó			K-¶iÓ&9¬þã?Føóé§òÀ6RY=¬³³SÏ>þ^ÁUÙl6¿Óf===ÖQlff¦,ær¹dÎodgg3fl¤²ê7BX>Z³â±FFFÓQQQ¾ÌÎÎºÝî×¯_g;NÞglø²ê×ñ9rÄon%Ë½ÂmÛ¶ÍÏÏëÞ`¶-**òûu~3LY©¬2pÔï`úÌËý³¦¦¦K.íÝ»Wßæìíí5ÿ@z`ùùùuuu2!§0<ÌÌLi¶ù¦-®»¬RMwSÃ)gwíÚ¥Ý¼ySÎê7HØívËÎÛ:;;ããã###å¯º»»ÓËglzzzÍfKOO¸RVà;wîÜ-[~ñ_üò¿üã?þãý×e²®åçY¥v_õN_½zUÎÎÎÎòM@¨ª¯¯¦~ñÅSSS¿ûÝï~õ«_±e@Y×¦¬###æÛÈ´&ù& mÝºU²jmPÖUX8`>ër¹(+Â~ï÷~OF«æ9Õ?ù?aË²®AY;::$¥/^¼05''²!,22òñãÇæ9ûöíûÓ?ýS¶(ëjËzãÆé¨Ä444$gkkkWó5LØ³O>ýå/É¯Ó²®ª¬/_¾½yó¦ï/Êéw$QÖ®¯¯///Ïáp¤§§<yoH¤ô÷ÿ÷·lÙòÅ_|úé§2ý¿ø6(+¿"%y<ÊÊÊþþþööö~Õcccý×úçþççÎc²®YçççeÀzðàAÊNgmm­qvjjÊårÿý÷løpeÕwX<ybùæÍÊºáLLLØívËõüùó¥¥¥lxeõx<ÆW655IV£££eÂüM~²®ç²ÆÅÅYÊZUUEYàýuaaÁnnnÖï]zþüùzøö%ÊºJiii/^4ÎNMM9Ny©Ä÷XVË·óëoÉÕÕÕ­¯6¤¬«ÑÕÕe·ÛKJJZZZjkk%«Á¨¬GË&**jÓ¦MM^2½Ô¯ÐPÖõott´¬¬lÿþýyyyW²¨¬7S¼/cZê7É)+²úÿjCéhgg§Dt``À·¬÷)+ñàÁ9e×ÖY=Ie²X&&&ÒÒÒäI©  ÀéõìÙ36ÖiY§§§GGGÄRVëPNNN^^1T=yò¤Ä+ÖïÕ<x5Ê°cÇ§^23++²Xyu¾yófy½^^^¾Ü"Ê«ÿ¸¸8¶gJYå)mõ^Vý!¹/_Ê´Ýn²...:uJfVWWSV+pþüùÄÄÄ¶¶¶¼¿^RR²¬kèêê¿²Ì,((à:±Êj&eMNNÖiýu9Ê`¹¦¦¦d¨*A5æEGG÷÷÷%ò'¾cVÃÁ¬¬2N|Õ¦îÞ½Û23//ïúõëËºùI¬ÖZF½rµ¼ÏVV~EÀêÉØÔétZffgg/÷+6%¢n·[»ééé2~Ý¿ÿèè(ë½¬2H-,,4Î>~üXæx<Ê`5Ì£X	äððð®Jj*OJ+û[àCu||ÚÓÓcíGüqÊööö¶¶¶óçÏÛív<Bu~~¾¼¼Bè666ê'$$?!gÎRVË288XRR%Ï$üÔB¿¬Îk×®CU¹j9=xð yüúùçSVeöV§Ó·oß6>ÕjÌ<ö,ePÖuûöíÓÓÓ2177gÌ¼páePÖ`Ë £U9ÕñññÆçÎ£¬Êº1«FtxxX¦»»»uæ©S§(+²®¼¬baaÁyæÌÊ ¬«*«yï¾û²(ëJÞgmnnöýXÇ(ë?ÏzéÒ%Ý___äÈËp(+².I*á|úô©óæùÒ+W®÷SVeå·n ¬°ÑËñå_wÜOÚPVÀ.«~á·ß~kÌyòäåwå(+².ï72©ÀÙ»woQQ~§³³²(ë2>xsõêUMìgæÛ]¾7@YuíÚ5ã]Õ/§üÌü5ý@Yß=`ÍÍÍÕ<×²[ù¸?!GY©¬Ò¡¡!9­¯¯×³ÍÍÍÆ°U&êêêdþ«W¯(+²2>>h~'U÷<9ÕÃÒÒÒîÞ½+g?NY5Øë;Ë:22BY5Ø²öôô=ztÓ¦M_ýµÌ?vìØ-[¾üòKö(ë²Ë*§rzçÎÆ¬Êºeñâ655QVe]UYÇÔÔNKY·nÝZ]]=::ZWWWVV&3çææ(+².ï&6u~~ÞøLePÖe¸|ù²å+øïæ"BÃèè(>ß¼y#)=xð ýúµ½rå^DY7®¸¸¸ÄÄÄãÇ³Mà=uaaa×íÛ·ívûáÃoIVóóóe¢¾¾^¦åV)ë766&Óýýý.«¼¼ÍïqÌº¸¸h­tôòåËo×Êº---N§sffÆ3<<-Y6¼¯²NOOïõê©S§¤¬ú¥Á:`=qâeÝ N>í;BMOOðàÞWYõ`ã¨¥ûöY.eÝ jkkåEefRRR?ÞKYïß¿ëeü«^Í¿ÏzõêUýÊºé¾ßöövcNee¥eÿ0PÖµ,«1ZÕë®]»Þ®3uÚÚÚbbbòòò***ÒÓÓSRRúúúØ,ð¾Êj²vwwSÖÐ3::úý÷ßËhµ¡¡càCU?½ÚÚÚjüæ¹ù¦k×®QVe]^Vëêê½dÎGÿµsÊØ¨eZ®ÑRÖÊ ¬ï677'k<pàqð'dþ¡CE×ÙÛÛk·Ûm6[jjjGGGà,LYrÌjJÍßÄ´n·[§ººZRø"ß9ÿÛËåJJJªàýøõ¯ý^^+±±±i'$$¾ÈwÎÿò³uëÖtÞ»Ý¾®Ëj³ÙüNû½(ÀÂìlà½ÁkÈümMQQQ/°0ePÖlÛ¶M¿×INe:ðE¦¬Êúüüüºº:S·Ûø¢SVeýIggg|||dd¤Ýn7¾+Q?´ãß)+²òMÊJY ¬PVÊ ¬@Y)+²(+ePVÊe¥¬ÊJY²(+e²RVe¥¬ÊJY ¬@Y)+²RV(+Vaff¦¯¯ïÑ£G2ÁÖÊU¹~ýz\ÓéÜ½wLLLCCC¨®i[[[yyyiiiMM¯!PVÊú^x<©i»ù¬(ôÖ´¸¸811ñôéÓ/^×YYYSSSü@Y)ëËÉÉyÝ¿­¦¬TJJÊèè¨«¬cYYÿPVÊºÆNçàà yØjæååÕÖÖZë.(+e]c2t»uëyÍÈÈ±Õt»Ý---æ9ýýýòª(+e]c555ÃØM:66&½©ªª±Õ<~ü¸[Ís***,s²RÖµQZZ'§eeev»½¸¸8ôHIIU²vçÏ5a+>ÊJYß®®®*/ã áÐ#ãò	jtttZZÚxÜPVÊ ¬XZUUUVVÓét»Ý]]]l²RV`åJJJGKKËàà $6:::÷Æ²ïPív»qT¶hhh½O²ÈùóçeÌj711ÁÆ(+eVRÖÒÒRÊPVÊ¬®®.é(ÊJY5ÃLe¥¬À fddH_Ýn7_^PVÊ ¬ÊJYáWKKKeeeMMå'f²RV,ÏØØXzzºÓé,///,,üäOÿsñþýûGvvv[[Ûe¥¬áNgüÚ]»Ä5Èkii©4õÖ­[²¼$6&&¦¡¡M²RÖð%AÊ°Õ<SBñâÅwþ­ïwz<¹¶©©)6,ÊJYÃ4UêhYQQQYYùÎ¿õûÍG»wï~ôèe¥¬áKÊjùô§ËåºuëV0eõý¶^ù[Ê²RÖ°VSS¨qX¦¥¥o»ÐÕÕ3<<lÌiiiao0ÊJYñSââdð*]ÌÈÈ0¿uØÉ'¥Ê2xmkk«¨¨+¹~ý:Ûe¥¬øuÃMiê¡Cöïß_\Ì~`²(+ePVÊ ¬ÊJY²±±±<öì#Þ¦¦¦²²²ÒÒRæó©@Y)ë:URR²yóæ´´´ÄÄÄÝ»wßeËÉÉq8U^)))Äe¥¬Ree¥ÓéÔÑªÄ¬¢¢BÂö«vúôiéºñáT»T[[Ë£²RÖ'..®¿¿ß<'==ýÿ¶ZFFF»yÎ÷ßÇ£²RÖfll,11Ñ2S­'OüwCF¨º·µµíß¿e¥¬OLLåwÝn÷Þ+ÃSÉ¹yÎ¡CÊÊÊxtPVÊºñHØ7V[ZZ¢££?ðAÂqqq^ú½ù÷@e ¬X!)Ëår:åååXÉj0¿uºæ³³³c¼dÂ²s(+eÝHdÀzýúu)ÖÖÖ2RQÖÞÞ^»Ýn³ÙRSS;::ÌuwwïÜ¹S.a,&s&''#L(+²Z¹Ýîúúz¨®®ÎÍÍ5_¬w½±±qÇ2ÑÔÔTTTÄ@Y»¸¸(óóó			K-¶iÓ&9¬6773üñÇH¿øÅ/>ýôSx@Õf³ù6ëéé9|ø°b333e1Ë544$sþ¯ÉjVV<V`ll¬´´t÷îÝòìÐ¡CÀÆ(«2é¨¨(ßfggÝn÷ë×¯Í3ÇÇÇN'7®¦¦¦ò]Ä~ÍÌÌHS³³³»ººúúúy°l²AÚ¶mÛüü¼îiËRÐ¢¢¢ÉÉIß+ñaÊº!èGc322ôÃ<2L_²úôé¬¬,ó=¸ÊÈÇÀ³æçç×ÕÕÉÊØÔ|ÇãÉÌÌ6Ó422¢Å(ëFôìÙ³­êÙÑÑQ)VVV~ô;&ÿü¥däêr¹u%²:ü@Y?²ÎÎÎøøøÈÈH»ÝÞÝÝmjå4!!ÁòÃa³ÙÒÓÓ%®õcx­ìo%¢24ÏéêêJIIYe½~ýºyÎ£G/kMMÝK^7ÈØ÷hÊÊ7E (ÃÃÃ999qqqrº¯.))Yf~òÉ'ÕôëÌsÜ,k$äÁ?xêN^:X®e¥¬ðC!ËÒÒÒ	9ëñx$'ËýÊC³Zr%×³Æ¬2ß¿¿QF®²RÙÙÙ2`rè)CUÍªAþö£|$ÊJY7/Z~Á­©©i÷îÝËºUyú~d¿ÃáñâzXA¹KrOr¼N<©/ ÞillLÊjYQQ±Þ<@Y)ëºVRR"qµÌÜ¼yór¯§««K»2N¢üù:Éêjz,¯,o<ún+²â8~ü¸å×Reê;V%±A×¹ìììÒÒRóªEGGó³<e¥¬x÷XSÑ××gÕòòò,ú§ááaïß¿_Æ©òúCww³YÊJYñnßÿ½d£°°P¯N§3##cjjÍò÷ð®óçÏËëòòòööv6@Y)+õìÙ³ÚÚÚÊÊÊ[·nU²@Y)+²RVe¥¬ÀÔ¯r	>E²+÷ìÙ3iêñãÇ'¼***øX¡ÂÂÂ²²2óòòr¶Ê¬ÄîÝ»ïÄP---ÙÙÙlX´´´®®.óë×¯çää°ePV`%***dª?Èc´öüùólX©©)IiVVVWFFLC²RV`yÌ?×*¡ñ³<(+ePVÊ ¬ÊJY²gÏUVVÈéàà e¥¬X¹ööö¸¸¸âââ9é¶¶66ÊJY±SSSv»½©©ÉsëÖ-ëØØe¥¬XÉ5##Ã23++«¥¥²RV,Ûõë×óòò,3Ø8(+eÅ²õ÷÷GGG¬tbb"..îÑ£Gl²b%wïÞýàÁÓôôôÂÂB6ÊJY±rUUUãO>III©¬¬äÛêPVÊe¥¬ÊJY²@Y)+²RVe¥¬ÊJY ¬@Y)+²RV(+ePVÊ ¬Êe¥¬ÊJY²@Y)+²RVe¥¬PVÊ ¬@Y)+²KY=ú«_ý*yíüÑýQbbbr	Ïµþõ¯ÍZ³Ö¬5kýN7o¯²ü5õw÷wÿøÿøÂIii©¬õ¥KÂj­ÿþïÿ>×Z^ÊZ_¼x1¬Öº¬¬LÖººº:×úþáÂp­/°×ùOÿôO333aTÖ5×ÖÖ&Êý×ÕZß½WÖú?ÿó?Ãj­ïÝ»'kýúõë°ZëöövYëÿøÿ«µþío+kýÃ?ÕZ<Yëÿ÷«µþçþgYëû·û·EY)+e¥¬²RVÊJY)+e¥¬²RVÊJY)+e¥¬²²RVÊJY)+e¥¬ïÇRRRæææÂj­;;;e­ÃíõÄÃe­ß¼yVkÝÕÕ%kn¯¢º»»e­ÃíõÄï~÷;YëÙÙÙ°ZëYëó*²@Y ¬PÖðÕÝÝ½sçNÍæt:ÍMNNFÒZìÚµKÖ:55µ³³Ó|l»Ý®uttÉZðc­ô5ËÌ~¬¬u?ÖV-ëkýkÊê_rr²~dccã;Ì555äZËÿ®æææ·ÞCxbccÍ¹Ýîúúz¨®®ÎÍÍµáÇZ,,,ÈËGßg~¬¬u?ÖV-ëkýkÊún6m2DCXkk«ÖÍs$921??&kÚõ©S§Î9ãÛÐ~¬Zë~¬¬Z?ÖÖú<Öõzzz>lÎfffÚl6Ë544z¯è·lÙ"Ï;2X7Ïõõ;ÚkÂõ/d¥äYÕ·1!üXXë~¬¬Z?ÖÖú<Ö5ÙÙY·Û½Ô7[8!ãîÝ»Û¶m3Ï4¦£¢¢Âd­Cø±ÎÉÉ¹ÿþOO>	áÇ:ÀZÃÿkßUÿ×Ð÷÷XSÖ@GQQÑäädeBõß¢ïXIÎüüü[ï^£¥òzkÂuÄÿ&uµÿ×Uÿ×Ð÷ôXSVÿ<Offæôô´ß###^Y&Äö¼õmYµüüüºº:SÇÉZðcmeN?ÖÖ:´ÿ_/µj¡ýÿz©µþ5eõ/!!Á÷µ­Nôôô8Ü¤§§ËJkÝÛÛ*«¶wï^c°®kÝÙÙi·Û¥@a²Ö!üXûmLÈ?ÖÖ:k¿«òuµþ5e²@Y ¬²@Y ¬²@Y ¬PV@Y ¬Þóÿí]»v¹½®^½*s>|`ù´´´À×©¿~ìØ1=ûúõk9[[[ËÖ(+jîÞ½ã%Ú=~üXNõÂÌ¼sç1ÇápHz¼nß¾ÝÜÜ,8pÀóÝwßÉW¯^ÒÙÙ)sEÑë¼yó&Û ¬@¨3+ûúúä´§§G'´`]ìÅröåËrºwï^iùAb¹¨¬¬Ì2G¬7ÆÁûöíéË/Ëtuu5@YP`ü²ìÓ§O¥pãããr:00ÐÚÚ*SSS²Úívj_·lÙ"#T!ijjªXëëëeÎ'O,£^¹N£â³³³æë@YÐ¡CUéëµk×¢¢¢t|)Ô¼¯^½²ÕêoAëÞcà>þüíÏoÓ>ö¬ÌèÊ´ÆøÂ2GK,7nÜà(+:¦§§9"kllÔ4ÊSj'2Êô-«½ÿ~BBîÔýæodæÐÐP|||nn®L¯|~~þ³Ï>ÓÈú·999HËõð()wîÜ1)Ò÷Y«««eâðáÃZÖÖÖV]Rò)ê~cÔxU÷XÂ)#`9½wï1Ø¡ªq¸yyÊm¤ÛíÖøk%2l/^cP]FßMJJºí¥Ç(éDll¬ÄrqqQþJ¦uyc@ì;fÕPV Dè[­ÝÝÝrzéÒ%#x§NÚ¾»qvjjÊøÍjSSïUMNNêaÃ¾ùdÌPV þ3GDêÄÎ;eâÚµk[¶lp&&&Ëo Ê¥r6??ß¤9ÉÉÉ~o1+@Y° ;~gffdº££cÇ:ùRVm>·Zõh&>~üXNe1Ýå+ÖñfÌPV ôéÑIæc®"sFGGßþüôôtÍ¡yïî«W¯t¦ÔÔ(«WæH;fPe²Ø0/ "¦¦¦z<wþÕÍ7u+Eå·oß¥MMMÉE[·nõ-ëÕ«WõeyËµ´´T¿nG@ØÙ±cG½qV¿ªéÄÆÙÙYË°Uw&×ÕÕ10(+Ê(+ÊeÊeÊe²Êe²wùQÝ<ãÚúÍIEND®B`


血钠


正态 Q-Q 图


«®êyïòËãòåËàNxèÐ!­eõüîù¨+´æÓO?÷¿Åþ3eÇºÚÜÜÜñÍÏÏçmèG[·n]·nÝ=WÓw´¾?þøãòòr+º¬­¹k×.kÉ´¤+ÏÞdýì³ÏÒÛÙë3±j85Þyç-Q»ÓdUã¹ç3UÌÊõ«W¯ª½gÏkå/¿ü2¡_!hÞ_üâZ¢ôa¯¶UÂÞéoÑª.×ö'Ý°aCÇG³ÕúÛ­â^¯¼[?èæû5  ))É¤Â/¾hÿÊÎÍÍµ¾íëÛëZÕ^*aÍ#x<+·Î?ßéÓ½ýöÛ'OÖjS§NÕÂ÷ÞÏlµòÉs=KVvöJÑyrròÑ£GïtE¦yÞ¼¼¼×^Íüt0¯öÌ3&õMÊÚïuíÚ5¿?MÅî]V°8qB+oß¾ÝZÒÔÔ¤%õõõøý!]Ù^m%«Ùt¸µ}ÞÝÉt;YUÊj£Û¶MµQQQÀëïØ±ÃºZSS£%'O´Yêt¿¦÷IbÅ*¼ìë(Þ:Ç®$«O?ýTh¶£ªÝé:7oÞ´¿~«Î3¿t¹eËÛTMné¦Ûí;MCBBÔ8wî*;äj¬²Ó½ãj=µWr?Äü^QûN±m%«Jmëõ>Úü|¹páÉ¸oÉêr¹LMi1;íTPÔq»Ý°'ë+¬d½téR§OgÚ6mòj³üÃ?ìxG«"ôKÖ»dÉóªT|ßå@'së	nÛÑ2a¬Þ0ø+Wüî¥?¿¼¼¹òU_üâ&VýÊëQ¿uú.[7nÜØi!®/^l=HvvöÝu×®]¦gô`Ö¬YZxèÐ!û:$+HVà>'«©öÝx¦nóù|¦ô4ûº¬aaaªzõ V=zdöÙgMûO>1©'2»;-a÷æ¡t¹uëÖÛ_dÔq»®lß¾ÝÔcVÐKÒÂ_|ÑlMÍÍÍ?~üo¼¡«z¿ûªT·ßéV·BÑÚänöþSÊRÚÚÚìw|÷ÝwµÐ mV>ºÕÖëì¬ÖÍlßö;dÉÜÿdµÒúâ¾yó¦¬ª>ãââöìÙcí¼S²;ÖoªIe«¼g²Þsk°bIùíp8L½æx¥[·ní®f»´µõU¯ÄÚv*J8óÍqUw:ÑHxHH~§ÐÓ>|Ø4&O¬Þ³¹»wïVÝþêêýû÷kMý°°þ@óG>ÚïYTëo·òþvê³gÏî4YU¶?GÕ³ùd2dÉÜÿd5GÃ¼	4'¡ú²¶n5_åË-»ÓÓÙkVëëL³²ã©]LVâD¯Ó¾%V¤;wîýoQÎYWæ'9XWÕ°v¬z<¿J×#­.²jèuÙ<®òvûÎWk×¦"ÜïÜ¿³l;=æöW´Mr¯ZµªÉjþQ*ë/5õ®^¿IhS|: Yû¬·¿Ú£Ùqk°¾µU«%44íxï¬*¡üV0Û?úè£ÞÔ¬Vågvâ³ÅU±èwtÙUlYYyiÎ1Õ¥9Æ:üÇ8qâU¹Z=sèÐ!=¸µ-·Ó]­Ö~ë[ß²_;w®ß:æ|$sT³_×Ý=YÍQKÖs 	fó¡fÉ<ÉjÝÅ/Yý*Ô®ïgí¬¦d4/©ÇÉj*<sÔÙ°líhÔ«2Ë­G0Û¢ÍóZç³fddíºfûv½96Ø:FÜéÀFê:¿%aª|û±ÊöÓcìëèÕv¦]Ù,+V¬0ìwÉ¸oÉªÈñ;Ä|q«$?~¼IV«zëJ²Ú÷³úÕpû«¡þìG±v%YíçÃ!<=´Ùk6ùcwÍ DÖFZ+YÍ1º»wïV|ºÝîùóçJÚÛjjÁ;÷Ûé½®é÷«åQ×³d5	mÎ0öMdÉô1Å7k¤ei83Qól×EYÿ 4gÝt1Yí`qxêÔ)¹NHÁi­_SSóÜsÏÔrs0­9¨Ø:ªÖeÖ½Ìñ½fß»lÍªöý&«fÍÌÌ¼ýÕfxE+Y÷ïßð3CLÜ^Ùuj-1;SïÊ~]§ê®ÔÔT¿°nßafõ°¬ Yþr]÷¬·­ä³'«¾îóòò´¤ãÖ­rýè1Ç.ù6ê7 RwÓf¼O?ýtÇsTì¯Í:DH>¬_ö×¦ßwðöWíÐ©uëÖéguUiºxñbÝ1**êyÜ1e~nÛFºè8uý$®'	¬@ÿ²jiµ¬¤tGéCðÑµtúeðÆotëîf÷í]¸½xñb`` _¡|û«CýN`íúKíôÖÞóüèHVHV@²@²@²¬¬¬ddd$+Ð+fnWû¦ºúÞïuºò_~©[O:uæÌ5.^¼Ø§¾~ýz§3¤ê÷îÝk«¼?ýÁïdúâ­ðöÛoÛèÛyv»Ì¯ìØ±cûöíZóÃ?¼Óãh'OZi_SWW¬Xa¿êõzÕ8xð îµqãF3·ÚjÌ?ßþ°Áív´Ó­-2¯GíÝ»wu._¾¬«MMMñññÏ>û¬YÁÉýû÷ëVû³û=YX__¯ËÔÔÔî>ÝÇþHV ïéKÖÊHç*&íßãöòÈ¸qãiDDDÌ9³å JoóðáÃ1íÌÕ­[·Õ.]º¤«*÷ìÙsáÂ1c×ÜÜ¬[7lØpj;wÎ¬¬Kíµk×Ô.//¿zõªÈápXk*®NçùóçSÛYòñÇëEª±eËî>ûß?Éô1+)oÝº¥ïß¨¨(q/^¼XºúÉ'èRµ]Ç;v=YýJÀ£G*6fÍe®,W`o%½Yh^²!00Pmû|ùå999º577×z©ªØLÃÚ:ªÇ´þ:³]Tö.wíÚeÒ¬V?Ü¼yÓþ­@íÖ³÷x;Áõ@²ý¢©©ÉlçTÅ¦¯ÝÓ§Oß½fíV²ê+^ßæ¦òSû.ÉaÚ&ö6nÜ¨l		17ýÂÀTx¦KIIéëÖ­³ÿ	-2s/^¼h*B³9Ô&YUËª½~ýzûÆáN;¡[Ï~O÷½è·ìWõÜÊ+Í12çÏ÷Ïlgm:V¸ªam5íK¦&6ypîÜ¹»'_HXÏîW¯^µVPI÷LÏ·wï^+lN8Ñ1YÍë4L³ÚYÔ3gzðìyÐú YþòÚk¯Ù5!!¡ã×±µeµë5«áÇëJnSc)óTVêyu5;;Û*³ÌÑ:fÿ¢'OVþ322¬Ã©:d­¦GÞ²e£N<Ùi²µÛÕJYµõ¦7:gWýÁïdúªUS¹Þn?¦Im³Ûµ[íÉ¡jØd"¤crSDF­un·ïìTi%½&kkkKMM52wîTÆ©ýî»ï-æõ[ûÍ±Á¦ô4»ï¬*s­¶²ÊE]ö.Ö¬÷½èú*×WíªU«ti/Lû6Y½^oMM*¿î´µÓÎTW¦VÓ#Cg;VÏ&BT´ù=BÈüQJJ?qqqæØ`³S¹c²&%%eee©v·ÕüÂÐ/eîÛõgïnÍ:ðý¬@¿0¼¨ª3UÑ5kú<YUðYe9DÉ1út¿äPÑ¦¶ÛíÎËË3'|úé§f'O8pÀÓk/Î´D/C)¨Ø0w±¯`ªO³ÛüzÐã¦æè_¿³nL7öl¥é+Ì±µº¯9xÙ²eöÄêÊ³wÑìdúáýjû¶µÚñññ&fôÝñ.õõõæ¼ã¸ÒWü-[vïÞ½wï^³äôéÓfc¦%fCè/¾hý©S§;fKsVµlÚ´ÉzµzdåÇí¯¶ÜÊúõëÍGsªÉ%KhnÓe­JW­×in2mÊWë&=¯	°n=ÝÇþHV ÝéëUÅ#ëK¼ÓÌ¾ºòòò.>2Éo#+Ë;Mce²íwÞ±Fæ3%£J·àà`kIHHP¡i~k¦BëèÑ£qqqVhÇ×r³ªÒ®¼~³Ñ5++«[ÏÞu÷«¬¬¬ddíÜ+&LÀ`âÄÿýßÿýp'ë¿üË¿<ýôÓ§x<ùäöÙC¬ßùÎw¾àð­odd%Y$+É YIVHVHV@²¬¬¬$+d%Y$+ÉÉJ²HV@²¬¬¬$+d%Y$+ÉÉJ²HV@²¬¬¬$+d%Y Y YIVÉJ²HVd¬$+d%Y Y YIVÉJ²@²@²¬d¬$+$+$+É YIVHVHV@²¬dd%Y$+É YIVHVHV@²¬¬¬$+d%Y]×ÖÖF²¬¾Q]]WQQA²¬^ñù|AAAÙÙÙ---$k?Ú³gO@@ÉØ¶mÛÜn·Ëåªªªz¨ÿ YoÞ¼9út«ÖÖÖÏ/X° ¹¹ùaÿsdýàV¬Xa%kD¡¡¡sæÌá­	#U¨ªS£££«««Ç_ô 'ëùóçnÝºe%ë'$''§¦¦òîKSSSZZZPPPnn®ÊÖAów=èÉqàÀ¿½ ¶À RYY©RÕívïÛ·oýiz²ü#v©©©*UsrrS©úÐ$«=b©Y`ªqqquuuõo$Y¡¡¡aPîU"àáURRâp8<Ï#GýK²úQJJJ@@@^^Ïç2ÉèÊÑÂÂÂÐÐÐÚÚÚ¡ó¬~)U=b5??ª$+ _´¶¶æåå%%%?~|öÉè3uuuÑÑÑ*UKJJê9VIVî3ÏcJÕA|®*ÉÕÕÕG±ZZZ:dKUÐZ[[³²²©iiitÉè¹Í7»§Óù°ÏUN²¬pµ´´,]ºRdôÊÊÊèvª$+ W¼^¯)UéÐs«W¯v:ª$+ ·Uªdffî	àHV¢T¤T%Y½U__ZPP@©J²z¥¬¬,<<êd%Y _JÕÔÔTJUdÞjkk+,,t8ñHVz¥¦¦&!!Rd%Y (M©©©©Cs®rd>S[[èp8ÊËËédkmmÍÍÍJII9räB²¬Ðsûöíu:ÌUN²¬ÐÛR533S¥ª×ëmll¤CHVz®¬¬ÌårEFF2T!ÉJ²@¯455eddèRd%Y ·¥ª®²²Þ YIVè¹ÔÔTªÙÙÙÿ@²¬Ð+¥¥¥#66vß¾ôÉJ²@¯JÕ   ¼¼<JUd^)**R©Çø$+É½R__¤Rµ  ñHVzN9jJÕÄÄDJUd^QÆÅÅ*UIVzUª%''3ÉJ²@¯ÔÔÔ¨TeTdÞòù|yyy*USSSèd>(UËÊÊèd^ªÙÙÙf8JUd^©®®v»ÝôÉJ²@Ïµ´´R533Sm:d%Y ç¶mÛ¦:5::Zzd%Y W¥ªT&#YIVè*Ud%Y ·¼^¯JU&#YIVèR5..Rd%Y WSSSd%Y 3ÉJ²@hjj¢T%YIVèÅÅÅN§3!!¡¶¶Þ YIVè¹úúúÃQRRB©J²¬Ðs>¯°°044411QùJ¬$+ô­ÛíV©Ê$+É½-U©©©©tÉJ²@¯JÕ¸¸8ÅêêÕ«)UIVz®¥¥%'''(((==½¡¡!YIVè¹mÛ¶¹3W9¥*ÉJ²@Ïµ¶¶R533³©©!YIVè¹Í7»Ýîèèèªª*zd%Y çÍp*Ud%Y WV¯^ít:Ù«ú TUjJUöªú1'ò&%%%$$¨Â¹¼$+ôJyyyxx8U;ÕÚÚ^WWwüøñÜÜÛ=èÏ>"Y iiiAAA999ìUíTaaajjªÛøÒ¥K,X@²¬à¯¸¸ØápÄÆÆÖÔÔÐwâõzýJùúúzÇC²¬ð¥jJJswÊÓÍ7ÛÔÕÕ%$$¬$+ür´¤¤D¥ªÛí¦Tí²²²ÄÄDû¦rem^^ÉÚ=ï¼óiÌ9SªöÏÊÊÚ±cGTTÔôéÓIV[ú*3¥*U».##CáªÊuçÎ^¯W8è¯_uÅ¦¡_íÛ·«þüyµ«ªªôsdðpªf®ò¤¤¤#GÐ!=¨ª¦§§ççç777ú¿·/õðáÃN¨¦aÆ"Y¿~½./`5""dð°Ø·oÊ,Ë/g¯*:YoÝºeJÕ±cÇd]´hìØ±Ã^³¬­­­YYYAAAÉÉÉLû¬ÖÖà+Wª¡x³fÍ²Uõ«ÁÁÁÏ=÷ÉàgÍU^VVF©û¬Ö¬lðPPæååRõøñã[+°)IÖ>¨YMÊ^ºtdðÓw)UKKKûöNgRRÛíöx<ÙrÉºaÃ-ÌÎÎfk05_\¬4µ¶¨¨(66Ì$ë½8pÀløU ª£¶~£-[¶lãÆfîÖ¬>ºòXoÊ£G¬úCee¥Ëåï§½ª~Ejrr²'YïáÆ&_wíÚuóæMëq¯/¾øbÏ5&&Æ<²yêÔ©jìï ==dÐËR533³ö¶´´DGGû-,((PåJÿ¬÷véÒ%½AU¤ª]SS£ö¦MtY__ß³dµûë_ÿÐ9sæð¿Ð]%%%ª&`8UÃ~ó¶*ÎËËËù¬÷pñâEÞ Ö£GjÉ;ÔÖC³­Øª_»;Å%KÔ¸ÚÁücjVÝ¢ò4))É2í© NOO÷ù|æê¶mÛµCapµWÉzãÆ_ÇúUj-9wî©b»ûàOý¾»víûYô5W¹ß4,ýGªpU¼`ÁËU[[Ë?d½oT/^¼Ø´C²è¦¦¦´´4Sª¶´´ôìAtÇÜÜÜÄÄÄ=N×Â=~üxee¥Vô'Yï§Ù³g_¾|³nôF[[[QQÓéT©ºoß¾?b5666++ëH»= ß>T¬¬û÷ïßºuk_%kTTýH%@XÀ-]º´õ¢A±j_¢pUåJ'£kVEà¯~õ+«­¢3³9i÷îÝ`ÀJU3\\oJUKbb¢rÚ¾DkBB]~IÖíÛ·½Þªª*kÜà]»véRu§2ÕJÖ;ø@²è[ÕÕÕBª¹¹¹µkSè7Ek=ÉþJÖ[·né±§N²×¯¯½öýª5É ø|¾üü|eª2Ï:·OÄgk04Y¥Ód:uª½fµF YôÅgll¬bµ¨¨ÈªÐ> ¾níñøÁû¬'NËÈÈ¸pá®nÙ²ÀPì©|T¦*AíÙÙ·âëYT¹&´ÓÓ«èßd5;VåÊ+&GçÎ«Æõë×U°.Z´ÈNdÐ·¶mÛép8JJJ¬ÑÄÇC¬rúôi]Þ¼ysìØ±o¼ñßMw?3dÐªï2âã!NÖ/¿ü2©]fgT³æççmÅ$+¾RQQÙN³¤ººZß6¹¹¹Öp¸fõÚW9ª íïqHV`Èª©©©f8k¨BsHQqqñêÕ«q²Þºu+¾9±UïøªvjO<dÐ'T¡ö±±±ö|©ú:ò_ÄÇC¬ÖüçwªYï4_É L©Úq8§ß6^Õµ*OMÛ¿Çcñ¬¦Z5§®Zg¯êG¥ß;ÿK²¸§ÒÒRÃ¡RuçÎuuu999éééº¬©©ù¢ï©Û×okkÓ·ý5Y¯_¿~_æÃ!Y¡ ¾¾ÞÌU§RU	ªÈ,((¨®®^¾|ydddYYYaa¡ÊVû½V¯^­ÑxXÕ¾·Uï~ëk×®éjyy9É Ìp*U­Qõµ$<<Ü~Bªê×ÐÐÐcÇÅÆÆªmnnÖ:U¥¯)g;Y<¨(=tè=Yûpj9ô«ªª¶mÛ¶wï^jPPÊSkÔ$Õ¯Çï.éééº9Øår±UdUnß¾Ï=z£oØ°ÁÆ¤ö'|¢öªU«HVwRXX¨þùçcbbôáv»»2½9%ÞÃ LVS¡<yÒÚízõêU-QÐR³¸§òòrU*UCCCõ¯®®¶¯c¶Ûç[mhhP÷ràMÖ+W*GwìØ¡öèÑ£ÕøøãÍ©ÜæØ`ûý$+0¸577oÛ¶M¥d×­ÿîw¿ûýï?((È	uùòå~·ÉæÍ®eeeJSµU×ÓáÉzãÆçNñyáÂ³Y8##ÃoCñHV`(())Qøe´s8JÁÞEµé#BBBJKK­½ªZÚqevZZZll¬nexÚd5Ûu¹wï^sÕÔ¯öóYÑÑÑÖ3úà»»OdM7~üø÷ßß~SAAANN]¡¬çÏ°OÂÚiÍÊü¬ÀP O¥R_WW÷EûF]-éte3ÓéT]«ôµïCUBëª.Å­YýâBBByæÕ«WnÔµ&Ó¥µ/¶¼¼Üáp$$$x<Uº~/C=Y©Y!(==òäÉÖ´nÇW¸¾þúë~5kÇ	à,>Oî#G¬½­ÉJÍQæõë×Û÷³êGö1c¬ý¬V©j YX³<yòÖ­[$+0X))].×íÇ«T5Ç½ðÂf3JUûpÉzdÝ°a5'«_Í«Ë¬À ôÓþ400ðÑGê©§ÌÍßøÆ7£ö	à(UA²vÕÍ7õ±Y·n]ß¦$+ðP2e¾"""BBBÛégôÒ¥KÖ¬YcMGGd ¬Àâ¿ü¥bÕÚPVV9¬]JJÊìÙ³­	àè(¬ÝpøðágÛyÛUÅ´ÍÐüj¬À 4¹RVúÐÐP·Ûmæ Y»±)Øo#°>T+W®¤f+ÏwäÈúúú¶¶¶Y³fY7544$''Sªdíã­Á$+0'%%ÅÅÅ¹øøø   /ÚO¼ÑMCÕêðáÃé(¬$+Î577×ÖÖêRíÕ«W+M­q+**Æ£9rä)S±QQQúxåWè7¬½JÖ[·né³´lÙ²ÉzãÆxH©õz½N§3999222--mÚ´i~ã¾üòË³fÍøJ``àë¯¿N×díóY­4U[Esi¬À©µµuçÎú¨v:xNNNJJ9	Õçóyiì+¨4ijÖüü|$ÉÚ¿ÉÊÖ`àÁ§LÕ_eçÔÈÍÍµ§£5<<Ü>¶n1bÄÚµkM;//OA«ZvéÒ¥t&HVêªVÍÕæææÄÄÄââbkºººääd¿=ùä=öØÞ½=Ohhèë¯¿®dí´ÞHVZ¢~S+Jccc­«ÊZËåóùìëÌ93&&Æ¸¤¯Uº¬þæ?üáøñãÃÂÂF9cÆ/j¡ÊPû:)))J_kñOúÓàà`UºEEE7nT½k0ÐÉª%É<P¶nÝ:lØ°   ¯×ûüóÏ*2öõö]­IIIn·Þ¼yTÝåûßÿ¾5W9~IÖ=ödddc¬ã­öºuëtUZxp81cÆ(,ËÊÊôÛW«ÂUëòåË;®üë_ÿúÑGu:kÖ¬¡ë~OÖë×¯3"?ðÀÚ¹sgqq±âÓï #ýÞ=|øp]]]tt´êT¦ª_õØïîªY³²²tÓØðP²2×ð`R%æv»óóó³³³UqVTTØõÏþ³i?~;oÞ¼°°0û#TVVF¶c8dðÅo¾hÙ«ì×¥¹ê7AÍ#¦LbÚ*O½^¯ÒW+Uû¬ú4>ûì³$+p¿|þùç.Ë4(/¿ü²YWXXhÕ£Záþé>þøã÷ÞOÕêðáÃM[UU¥»<:xPõ>°/ùòË/IV``´´´¨ú1cÆ#<¢«ë×¯2áZVVm­¹uëV§Ó9¼"V±ªò4--MáÌÌL&î[²êWísíÌÀ7nÔÇrÖ¬YÖ,èåååZ¢§$Y W#!!Á¯pUåªòÒ>ÄTuÇØØXæ*îs²^¿~Ý>íù"""222ØÜÓ¦M3jeeett´îMeë;ï¼ãr¹:=¾·¡¡!55Õáp1ª>pÿµãû÷ï×§WK.¬ÀægNç¨Q£F©(MLLÔçqÊ)ÖáKvf®rÇÃØ¿À¬fvFÓÞ½·Ú§N"Y4|øpôÆ÷Øc¾þõ¯+k;£MMM]ÅjII	¥*ðÀ%ëõë×ÍÝf¦­[·Zá|æÌ&L6lØìÙ³¯*^_zé%ó·¶¶ÖoÍââb3ùñãÇé7àÁJÖ+W®lÚ´iìØ±6KvìØ¡Oò'Ô¾téR^^®hMèWU¯×«ÆÚµk¬ñññ&MÒBû:JÙÅjii)¥*ðÀ%ëåËGm8¬¯¯W|^¸pA9úé§ÚW»yó&5+Ð£/¼ðÂ¼yóôéó;ú÷£>ÒBÓV¦¦¦666ÒoÀZ³Ò´¦¦fÏ=úû|>°j477+P½j¯ZµdúÉO<1|øð3fL6M·   û­áááæH=JUöªA²êÃ¼k×.¬fªÂÕ,W²8qB×^dúÜ¢Eÿõ_ÿµ¼¼¼©©éÇ?þ±>n#G<ÜnÂ	ºúóÿ<''GÁPÀC¬f7ªJU²ú¨cµüÃ?$Y¾ÕÚÚª¼T|æçç/X°@õèÎ;|òÉ¯6,++Ëår©rµÇàA¯Y­mÂæÃ¼°ÝüùóÕ;v,gÝH¿bßzë­¹sçfggïßüÃþ`nR¬*~ûm38JUà¡LVæºúÛçjÎ8q¢~¼=Úp_¢±±±ñññ*U###«ªªè7àáNÖ'NìÙ³ÇÚÿºûvè+ÊQjNNÎòåËõYäGµûè£ÌO=õ®ê£¡R533¹ÊÁ¬öaÔ°RdºK¹¸mÛ6f[î+ôÊËË+++óz½n·ìØ±*Lxâ	¥éÈ#UÎªÍÀ IÖò<h®9sÆÜD²ÝU^^ît:ÓÓÓ¢jèÒápX+M2EKõ)ûäO"##õåW$ÉjN]õ;¸?Î·!Y1¸=váÂ!!!óæÍ;uêÔísÑ?þÿùU~þùçf5¥ixxø#¾öµ¯ê.Öfa²q-[ÖiÜêG7ÉtÅÖ­[ÃÂÂüñ	&(/7oÞ¬å?úÑ´dÚ´i£GÞµk(t«¡¡¡³fÍÒ§ìÇ?þqKKd½ví,ÂÝö|Í5O?ý´ÙùzôèQûL®$+àGe¨b5>>^!úóÿ¼µµÕ6£*ö·¿ý­rT+Ì3G«êrøðá#Gd®r`&«RóôéÓf~VsuæÌæ&ý×Usª«ËåR¬À¼ðÂÊËÂÂÂ'xBÙÐÐ "õ£>ª¨¨HKK3SÓäçç«ZU²ª¡¸¥ëÁ¬öí·ß6í6èêÕ«WÙÜ]uuuXXØc=¦v]]ÓéüÁ~èv»_zé¥ÈÈÈ7*GcbbT¼*'LPSSC¿?YÏ;g?ÙFí¸¸8`î)77wäÈf®òðððÿ÷ôÑGÕV5ÊMX[[«ªÕ¼¼<J²êáÙgµ_MHH Y»;uêæ788Ø¤¢_¨Ã;v¬94©¸¸Øáp¨=rä=dÝ¿¿¾Î?oOÖ¸;§Ó©Uj^¼xqÆUsÜV±ªRU7°WBÉºeËN>­«åååý7ÉÁDQ:mÚ4§'NüÍo~£%QQQúølØ°!//OÌ^U`%ë"""¶nÝÚqê%K0º!Ð©¶¶¶ÜÜÜW^yE¤¤¤ªªªoûÛÊÑ#F¨UÜºÝîÐÐÐÒÒRæ*bÍÊ@·¤¥¥ÅÅÅ(>¬JSÍÍÍ<òÈðáÃµ055Õfd%Y¿ËÊÊ7nÈ	ÌË/Wêk¹råJå¨Ãá8|ø°*T³õÕW_¥ëõö7ô°hÑ"0fÌ¡Lÿý÷÷íÛ7oÞ<£×3pRkk«ËåRµªOuàÌ3®HÖ¿1G-8qÂ¾ðË/¿$Y1Ô=VEçôéÓ»wï¶çåå1ÂãñXyÝnweeå[o½¥VýºvíZ¦t²ÖÔÔXÃWUU)VN§öÑí¶¬jkkõY7në×¯·nUÙQ\l®þîw¿ÕgäñÇÿýï¯r633C¡¬7oÞ´ÚÛ·o7c9s¦?F4$Yñ «««ËÉÉIOO×¥211QçÍ7mÚ´¬¬,¥©ªX+Y÷íÛ^^^¾fÍ3ñªÙ¤¤¤ÂÂBbÒÉê7ãuÝºu½.øèÑ£.ËLú±ÿ~>e¤Þ´*C«««ö³éý¿zõj-W©ª=vìØèÑ£ßÿý/¾Ú¬Æoû[3¡bxÆõõõt#@²þeËé;B¿Ö«ªªBBBÂÂÂªÚ©=öìn=×ëÕ÷«V­?>É³Ò1!!A¿.¦TK¿øê¦Gô¥^zùåÍL¥¥¥Z'::zóæÍ¬ðÛêngpèñxºõhf,§7nDEE©ÕAllljj*ÿK<|òIsÒý«Ëääd.Ìú±h*7nÜ[o½¢«ª¬w.X9zðàAY<y²c²vwP~ùµ¿ßÁ¤IHV<>þøc½Éxâ	sõâÅªVÍQKï¼ó|þùçT½cÕ.**R©ÊÉzcMIªF_%«¾¬vHH[ñ :uªËåR^ZgÑ¬]»V5«.((HñññüãtR YïæòåËÍÍÍVÄöU²?þÆfk°Ú$+dvßøÆ7ô^­¬¬lhhÐ¥Þöæ¤¬ªb©qqqL¬Ý+^­dÒùSíÌØ§Ýz¨®[·ÎcìõzIV<ôö6Gá&NûØcéªYG3WyQQçÒ$kO&g½páÚ.KÉzëÖ­>ø@W­ZÕ­:xðà	õ8"Yñ`2G*ýÇü.£££¿þõ¯ëÝþýï_WçÌãóùÌpÌU¬@cÚæ(_FÀà£ýôÓOÕø¯ÿú/3ñÔSOUWW3@²öe²êË%3310©^¯W³äâÅ£FzôÑG³³³Uª2@²2ÐUf®r3©ª.###«Z>lØ°§ÓYVVF/$+ÉtÉÖ­[Mµ:nÜ85Þzë­ÀÀÀ³A899¹©©^HV¸³gÏ¾ÿþûÊÑèèè1cÆ*Ol~ðÐQÉJ²÷öê«¯6lÂ	&MR5ÊçóÑ>bðóÏ?obõOú¬$+pof¾3w<þøãJÙ¹sçnÞ¼9²Ý¢E¬X¬$+p7Ôàà`%ëØ±c_~ùå/¾ÚÏjÈÌÌÜ¹sg``àÔ©Sé+d%YÈËËS¦éýVXX¨U©ZYY9jÔ(ÅjPPÃáP#""Âd%Y;RX«×Gy¤¢¢b÷îÝfjJJÊ#^yåç~íÚµô¸·§~Zzüøq³UuêÈ#U¶¦¥¥©­â.@²]µoß>U«¡¡¡j766N<Ùì[5sÃM6Í$+Ð%¥¥¥yyyÑððpÇ³k×.Ã¡%+V¬ ¬@÷Ì9Ó>Dll¬×ëUcÞ¼ytèßýîw&P|òÉõë×ÁCBBdº§¾¾~øðáÒþðN§3??¿¬¬ÌkCCýdº¡¸¸Ø×ÜÜ|äÈ¢¢¢9sæh!ýdºêøñãIII¡¡¡ÒáíWxxxjjjSSÓ3Ï<C² Y.ikkSeddüþ÷¿7+Góßø|¾üüü'êª9HVànêêê<jÓÿ÷ÿþ?ùÉOÍøf«JØ¿üå/t¸#Õ£¹¹¹¦Tmll|çwS§NU¦*_+++gÌ¡[Ç£999ô¸£êêêo|ã£G5kUEjXXØ+¯¼¢Ün÷-[®øÃNgkkkQQQ~~>dþ®¹¹¹¶¶¶¥¥EI¹téR£&Løö·¿]ÕnÜ¸qÒ+WjMÝª¬ý·û·´´4Ëõùçërß¾ô!ø¦¦¦äääèèh]5jüøñÊÎôôô¶¶6³Çã4iÖÑEoB»±cÇ>òÈ#UÕ¬t#øÅ¤jÓ¸¸¸ïïÏÀÀ@%¥Þ9uuuÖj*O_ýu­JÜÏ§%Ãá¨V¬Àß)AGýæo*5ÇäÉÏ9ãt:¿ùÍo9rÄZ­¬¬L<gÎU®#GTu»dÉ:É|aÏËñãÇ7NÕçO~òU¢,ÈËËKNN~é¥²²²ì+ýë_Wªfõz½Þââb:ÉüU«æTÔ±cÇ6¬°°°®®núôéúÓbcc®GÚeff&$$:uÊÐÔØØHï Y¿kjjR|*Vþù'®^½Z»nÝºG4--Më´´´,]ºÔ¬ÝÜÜL¿ YIVt¢¢¢"<<ê3Ï<£­¯¯w»ÝIIIª¸Ê]z	ÉJ²âÞ¥#Føå/©dmmmMLLôz½¬¬¬4in:qâd%Yqo¥¥¥*Il^õUÏWPP Õãñ3æ©§¢£¬$+î¡¾¾>%%Åoø=ölXX®îÚµË¬cö³rr*dÅÝ	àGrrr``à9sì·*J®&M7n96@²¬¸#3bµ¤¤D«½xñ¢§zêGÉËËë­·;F YIVÜ±T-..6;444|Ñ~õ^Øºu«µÚ)S¢¢¢è.$+ÉªqqqÑÑÑfÉG4¬]``à¤I´ºÕÄD YIVÜ±Tµæ*omm5kkk¨o½õÖÿüçáÃ+YÍÑLªYé4$+ÉÎíÜ¹SujddäæÍ­ÙßdîÜ¹ñññ¦öìÙ3f8NÅªÞ!tdE'ZZZ233Uª.X°À>¡Ïç;uêÔ´iÓüÆÖ­üùçÓuHVþ*++].Ûí®®®¶*5µpøðáf÷ê1cìwÑ­Z¨Ü¥÷¬$+þ¡TÍÎÎVvæääXUÍò#FÌ1ãìÙ³ºúæo$''[·7ÎÚ>$+É¿ªÑÑÑÛ¶mó»éå_VvÚè pu:*m'N¨|¥¬$+þ¦±±ÑëõªTÍËË³ªVIúØcÅÆÆ9rÄZ>lØ°åË¿úê«öóYd%Yº²²2			öÔ´455©ÿÚ×¾¯Fyyùí'Þ¨T¥÷¬$+þîøñã*U:ªFzzznnîÚµkU¡ª6­««S;vlÒ¤I111ô!dÅÿ1£ê'%%),ï´N[[[dd¤	]ý÷®JÓ	&;«HVsäÈªJÕââbûø¦¯9$8++ËçóÕÖÖfggO2eÎ9Ä*dÅßjÐejjjªUÿ.âããµfhh¨þéN§sÔ¨QfîÇÓñàa YIÖ!§¦¦&..NYVVvÏU¤ªZUjåèèhU«n·û±ÇËÌÌT½÷JHVuóù|yyy*@½^oSSSWî2iÒ¤ììlÓ^¾|¹òxüøñöÁd%Yh©YQQa-lll¬««»ÓñÀ2qâÄ÷ßß¾¤¡¡aØ°aô'dÒ¥ªªÐ^ªÖ××'$$ÄÆÆ&'';üüüN7í>óÌ3*[íK^~ùåÑ£GÓ«HVuÚ¶mÛíV©j?Ú¨¥¥Åår4UÜ*e;ÞýìÙ³#F?vìZ±ªuõêÕt,drÌpºô;7Fº`ÁûãÇwZ¶*SÍÀÊTU«Ä*d***T§v:ª¾üìg?7o×ëÍÈÈ(**2¾iå.Ö$+É:(*U³³³;=4IõëÄüñªv)íÎ9s§HVR5zçÎwZgéÒ¥Ï<ór´¶¶Ö,IJJúæ7¿©ú@²¬ø©ªRµÓ	àìëêê*++®YYYn·[mÆ)@²¬ø?¥¥¥N§366vß¾wZÇçósl¾÷½ï544466kùsÏ=G7 YIVümÜ;ÍUnikkSE¤èÍÌÌ6mkpzzzQQ	d%Y:3\\§sÛ©6ÕjæàU·3gÎT¾êÚ»o@dä¬	àºr@¯*ÚëªÂ5'''00PÍÀHVu0Gfeeå¾ûLÙ5â=KUK~~þòåËíK®ááát8d´Z[[ÓÒÒÜnwff¦RS3§*JãââBCCºuîiuuutt´½6ÍÍÍõz½t;d´222,X`å¥JLÇc]µ&ëV©j[^^¾sçN=#. YIÖÁLÕddd¤ßaDJÖ/¾N¥jiiioIR¬*SÓÓÓóóóÙ·d%Y³ºººääd¿eeef8Ýzüøq:HVtµf÷«Y£¢¢Óéìe©$+É:kÓôôt]-½3óbÐçóM6-  @ýÌÞP YÑ¹ÊÊJÕ ÅÅÅÕÕÕEEE*RÕ°nUU­5>>>000((¨»ÉJ²!­­­RºsçN­=;µÎÂ«³fÍêxÊdÅöMOO÷[èñx¬ShÊËË###U³QªÉØ¶m[Çêêê322Ì$+º¤©©ÉápØ·ñªZ_·n«¼ªª^ÝPTTäv»+++¯JSÇ£R533©fdÉzÏññ»KiZZZL»#GRªÀàOÖCM>=88XåÔÑ£Gl²¶µµ¥§§GFF.X° ))IXWW×'ÜØØ R5''Ç½ÌÉªZÊ¼¾7N:uÈ&knnnJJµ¶¬¬Ìårõr­Òº¸¸ØétêWê`>0$Õ.,,ì¯ýk`Ã3gÎàþ?«¸´/Q	[QQÑãTÉ´téRöªÀPLÖÃ/Y²Dßw XMMMÄÿ¤èèh¿EEE½,Uûj2à!KÖ«W¯z½Þk×®Ù­ÁB¿QÒÒÒzP³VWW«Tu8WÆ¡¬_1W/^¼¸xñâK.åc;ÝÏÚ­£ªqzÍZSS3öìË/ñ³n:[ÛÝR544Qõ`¨'kTTTÍ?µ®®®¢¢bß¾]?æHu­×ë¥T"ú)UÃÃÃW¯^M©$+ÉÚs¦TMMMõ;]@²¬Ý.UU§FFFöæWÉ:Ø´´´äääZÓ¦Þ]CC)U333HVõïêêê²¬¬,//O5¨w¿VÐj±±±Û¶mã$+Éú¢££U°ÚÖápÔ××wºòñãÇSSSUªæææ2T!¬$k'I©ÒÓïhÞììlÖZJKKCCCÝn7£êÉJ²vNµiRRßÂüüüåËÛ9rD«©TÍÉÉ¡Td½#Ïçt:íÛ~µDUéÎ;ÍU3ª¾2UkjjxÉJ²ÞCii©Ëåª®®6%ljjjzzºÙ>[M©$+ÉÚUÇìC5&QõµDÉÚ­q$+Éê¯¦¦FAÄ¨ú@²¬½âóùòóó©*U«HVµWöíÛg¶	SªÉJ²ö¶T]ºt©JÕ.n YIÖÎmÛ¶Íår9ÎÒÒRJU YIÖkmmÍÌÌ`8 YIÖÞÚ¼ystt´JÕJU YIÖkii1¥jZZZSSoM YIÖ+//w¹UUU¼)d%YNåiFFJU¬ª@²¬½RVVæp8(Ud%Yû TMIIQ©êõz)Ud%Y¥¤¤ÄétºÝîmÛ¶ñdí´´´   ÜÜÜÞ@²¬½¥:Õ>¥9d%Y Y YIVÉJ²HVd¬$+d%Y Y YIVÉJ²@²@²¬d¬$+$+É YIVÉJ²@²@²¬d¬$+$+É YIVÉJ²@²@²¬ddd½×^mÔ¨Q1ÊívO<9÷Cttô×¾ö5úaàé=¯w>ý0ð¦L2iÒ$úá~á¨ÿûö1ÇC¬çÎûm_ûîw¿«oßbÀ½ÿþûï½÷]1ð«ßùÎwè÷£ý(,,~xk×®ÕÎÏ~ö³¾Øßýîwmmmw²ö7ß|sÖ¬Y·1àõFÿÿùºbà%%%½þúëôÃÀÓw±Óé¤Þÿþïÿêgûöíót$+ÉJ²¬ YIVd%YA²¬ YIV¬$+ÉJ²¬$+HV$+ÉJ²vÇ¯ýëyóæñ¶xùË_bccÏ=KW¼üàË/§ÞþçÎ9~x·nÝÒÎÿøGÉÉÉzß]¾|9**ÊºzèÐ¡éÓ§<£Gj.].ÄÇÇïß¿7GÿuþÉ'gÎiºúàÁtþõ¼±gÏÿûìÓóÓó.]°¡ç²ó¯_¿¾xñâÉ'×ÔÔ@çdÕ×w\õm"111f¼Ç7N:U¯×»~ýz5V­Z5þ|Þý×ùz7ôô/ ó¬çåæÍúMi-¤ç¦ç«ªªôån_°Î÷Ýw?üðÃ[·n)VÝn÷tþPIÖ¹sç9sÆï+Æ¦KÅ«ëÕ¸qãFÇùèÎß½·Çã¡ó²ç?øà+VXéùéyÅªß)ôüu¾~Ê:uj ;hígíôËýðáÃK,Q#88ØZho£?:_ÓèÑ£µpãÆtþõüùóçôb-¤ç¦çcbbfÏ­Vÿ>ÈÎW÷~ôÑGª T°;vl:¨'ëÕ«W½^ïµk×Ô´ðÖ5öì?~<?`=qàÀûBz~ßó/^4Ûièùë|uuyy¹úM£_6ÐùC:Yõ_¼xñ¥KÌUÅß¸qÃl0_÷èïoë#?0=ðèùûò7_åôüu¾½ægè&kMMÍìÙ³/_¾l-Y¸páºuëÔÐ¥YÞý×ù111'O¼Ý~¶þtþÀ¿[éùÏ;wÎü ç=?À¿téÒM6Ýn?+!))i:è&kTTß÷N0!00Ðåré·fÿuþÑ£Gãããõãñé§6Ûèüû¬ôüÀôüáÃãââôONNV¸ÒóÙùW®ÈÈ0;¹Ï93ÏH¬¬¬ddd$+$+$+$+ Y YôÝÇ8 `æÌÞvUUU6lÐÏ>ûì.ë?îÂÌþÆo«×®]ÓU3É%TöìÙÑN!ª´;vì.ÍXöÜµkµ$..NÑÙnÇÛ·o×Ï>û¬µäW¿úrÅºËÁµÄÄ<æÖ­[édë×¯ÛËÊúúz]>|Ø4nÞ¼iRPlV;þ¼®^¸pAO?ý´YhØT±yyy~KÀ&Å­:xîÜ¹jòÉ'j¯Zµÿ@²ÑVN:¥»xñ¢.O<¹÷n5|>_²º¦&_G­U¤ñññ¦`]¿~½8qÂ¯êÕcZ)~õêUûc YÁÃªÊ×M6úR)h¶ñ^¹rÅ/Y­ÔÌm¶×ÌmvÓÚW^¹r¥*tÕ6aüñÇkIb5¶lÙÂ? YÁãòåËË-SÂmÜ¸ÑD£*N¥ª2;&«®8p **ÊlÔï½÷´ðôéÓ&L?¾ö¿qãÆsÏ=g6 ûfddø´ÿ@²Ê®]»¬CÌ~ÖU«V©±dÉ¬»wï6k*>u«ÙnlvZ;YÍvàsçÎù§*`]îÝ»×*vUªZ;@²ÉH¯×kÂ¯©©ÉD ÂRe«çÏ·jP³Ù/ëv»w´3Ç(FDDÂòÖ­[ºÚf« îX³¬À avµ:tHkÖ¬±ï><y²uÕçóYw1±ZUUÕñ¡.]ºd¾yófÇø¤fHV`|nrssMcúôéjlÚ´iôèÑÎèèhkkªnÕÕZiÉN Y!ÁlømkkSÿþýS§N5õ¨+YMæ©ú´vµ£L=zìØ1]j5³ÉWë]Â YÁÏd?æèÓO?ÕæææÛ_QlâÐ¾u÷Ê+f¡ÒÔJV%®(»[³<yò.©¬Ö×¯_W"ÆÇÇ×ÔÔÜó^[·n5e®QëO<955ÕÜäóùtÓØ±c;&ëÌZß¯fÍÍÍ5ÃMðHV`È:uêúõë­«f¨¦wß×ZrõêU¿²ÕlL^·n)@²@²@²¬¬¬ddd$+$+$+$+èÿÓX`V$3IEND®B`
[truncated: 1,014,160 more chars]
