# Supplementary material for: Application of continuous renal replacement therapy (CRRT) in patients with severe acute pancreatitis: an analytical study
Source: BMC Gastroenterol. 2025 Aug 18;25:592. doi: 10.1186/s12876-025-04198-y (PMC12359950; doi:10.1186/s12876-025-04198-y)
Supplement: Supplementary file 5 — Supplementary Material 5 [file 12876_2025_4198_MOESM5_ESM.docx]

|  | improvement | poor | z | p |
| --- | --- | --- | --- | --- |
| Urinary amylase | 1103(484,2810) | 933(371,1016) | -0.353 | 0.724 |
| HBP | 92.83(51.59,146.82) | 105.08(48.94,192.25) | -0.301 | 0.764 |
| Toponin-T | 0.01(0.01,0.03) | 0.04(0.03,0.06) | -1.328 | 0.184 |
| Troponin I | 0.03(0.01,0.5) | 0.14(0.02,1.11) | -1.292 | 0.197 |
| BNP | 57.7(20.3,300) | 97(24.5,800.5) | -0.936 | 0.349 |
| Pro BNP | 585(118.3,1348.8) | 1789(777.1,2002) | -1.540 | 0.123 |
