# Supplementary material for: Application of continuous renal replacement therapy (CRRT) in patients with severe acute pancreatitis: an analytical study
Source: BMC Gastroenterol. 2025 Aug 18;25:592. doi: 10.1186/s12876-025-04198-y (PMC12359950; doi:10.1186/s12876-025-04198-y)
Supplement: Supplementary file 6 — Supplementary Material 6 [file 12876_2025_4198_MOESM6_ESM.docx]

| APACHEII Score | n | OR(95%CI) | P |
| --- | --- | --- | --- |
| <15 | 160 | 0.263(0.125-0.551) | <0.001 |
| 15-20 | 72 | 0.350(0.233-0.667) | <0.001 |
| 20-25 | 36 | 0.391(0.256-0.730) | <0.001 |
| >25 | 14 | 0.445(0.329-0.856) | 0.016 |

**The P-value of the interaction is 0.127**
