# Supplementary figures and images for: Application of continuous renal replacement therapy (CRRT) in patients with severe acute pancreatitis: an analytical study
Source: BMC Gastroenterol. 2025 Aug 18;25:592. doi: 10.1186/s12876-025-04198-y (PMC12359950; doi:10.1186/s12876-025-04198-y)

# Learning Curve (10-Fold CV ROC)

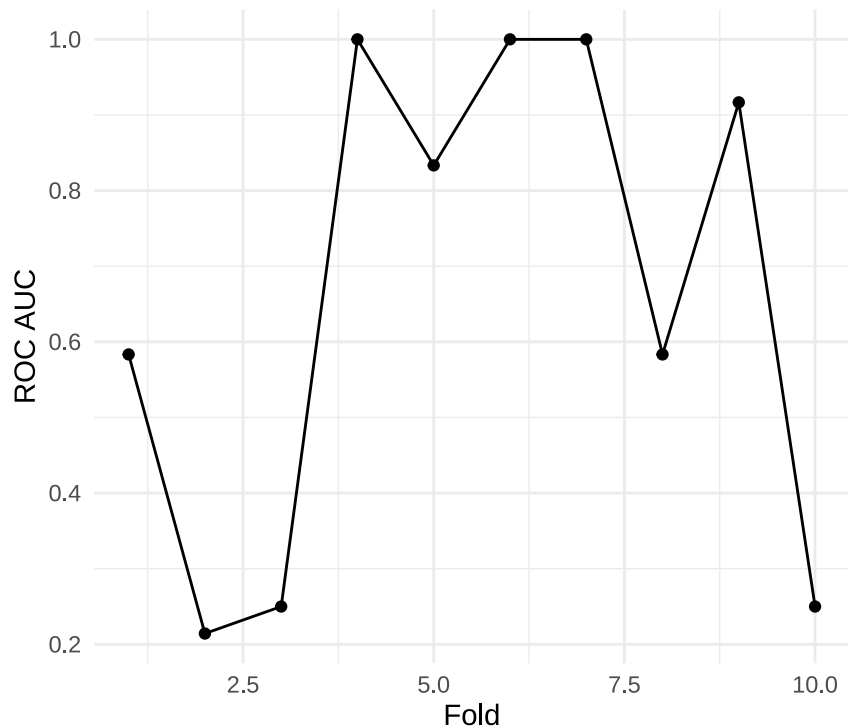

Supplement: Supplementary file 7 — Supplementary Material 7 [file 12876_2025_4198_MOESM7_ESM.pdf]

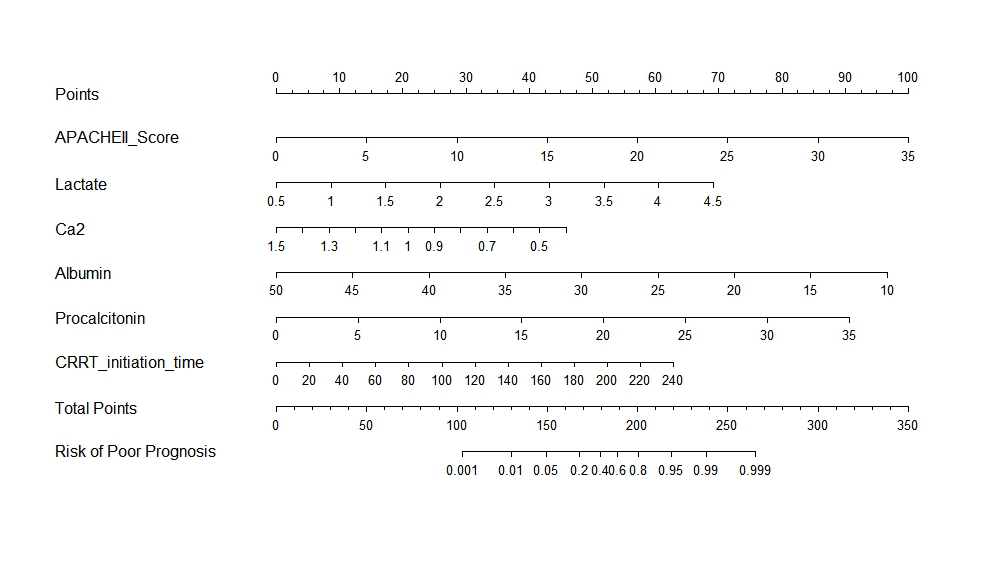

Supplement: Supplementary file 8 — Supplementary Material 8 [file 12876_2025_4198_MOESM8_ESM.jpg]

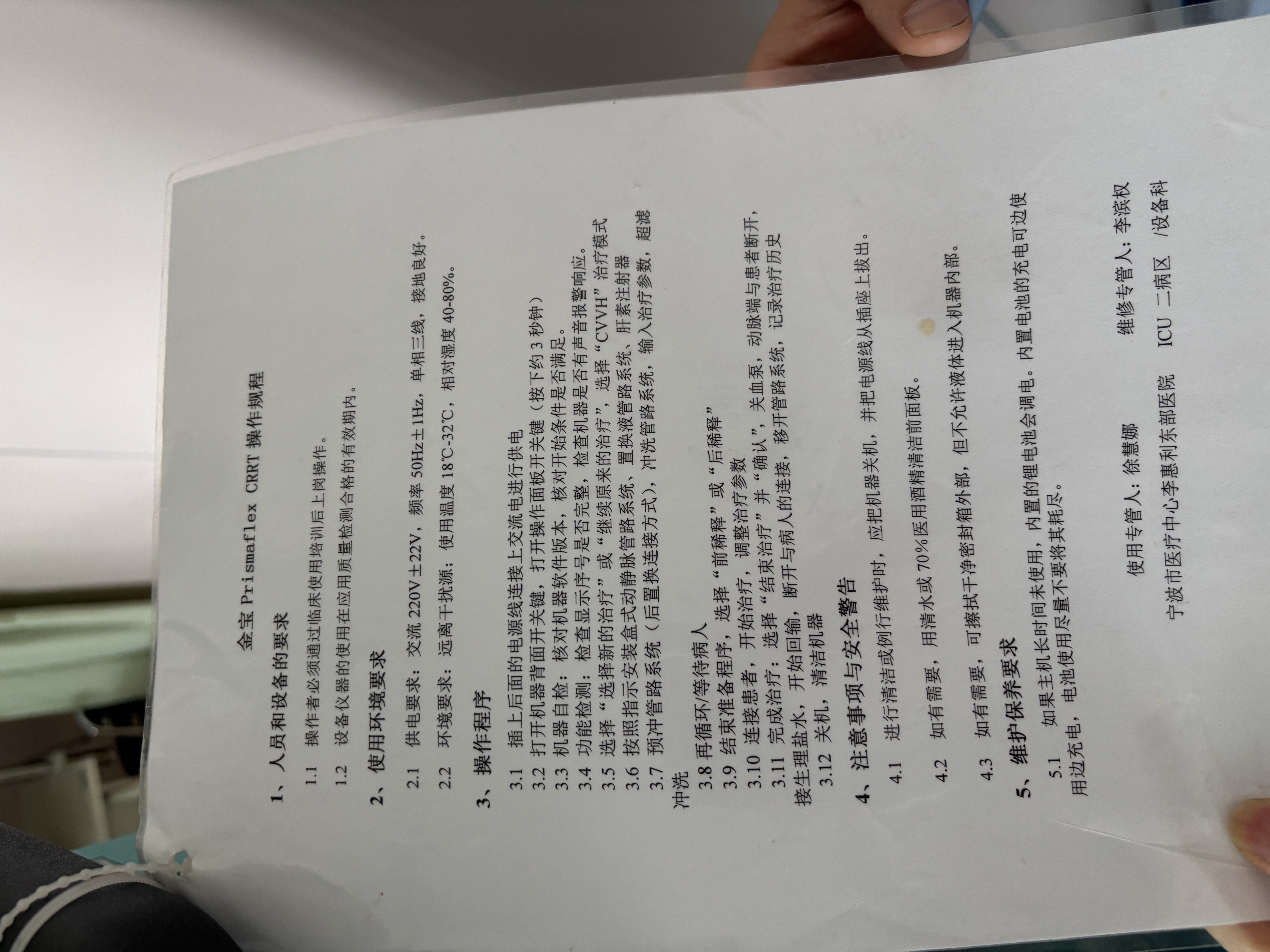

Supplement: Supplementary file 9 — Supplementary Material 9 [file 12876_2025_4198_MOESM9_ESM.jpg]

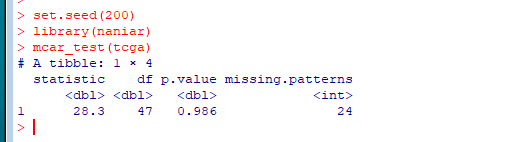

Supplement: Supplementary file 11 — Supplementary Material 11 [file 12876_2025_4198_MOESM11_ESM.png]

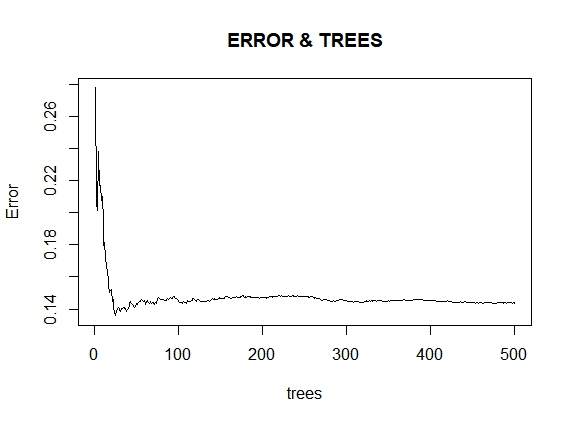

Supplement: Supplementary file 20 — Supplementary Material 20 [file 12876_2025_4198_MOESM20_ESM.jpg]

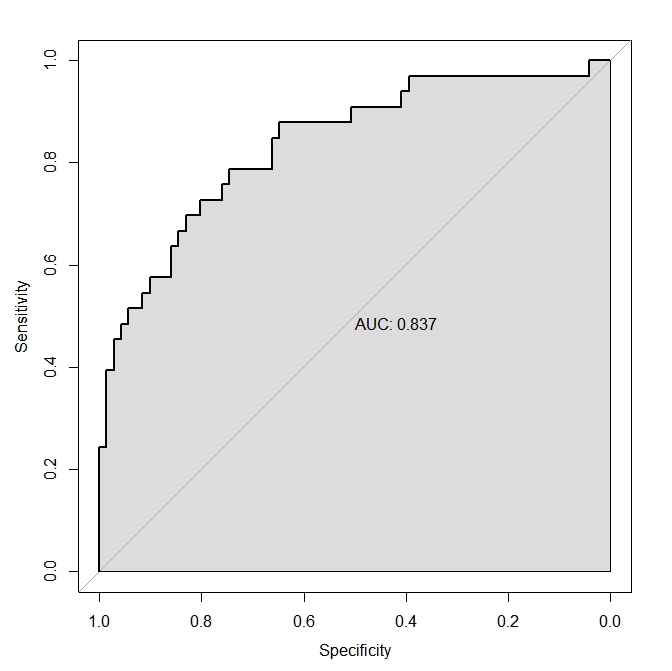

Supplement: Supplementary file 21 — Supplementary Material 21 [file 12876_2025_4198_MOESM21_ESM.jpg]

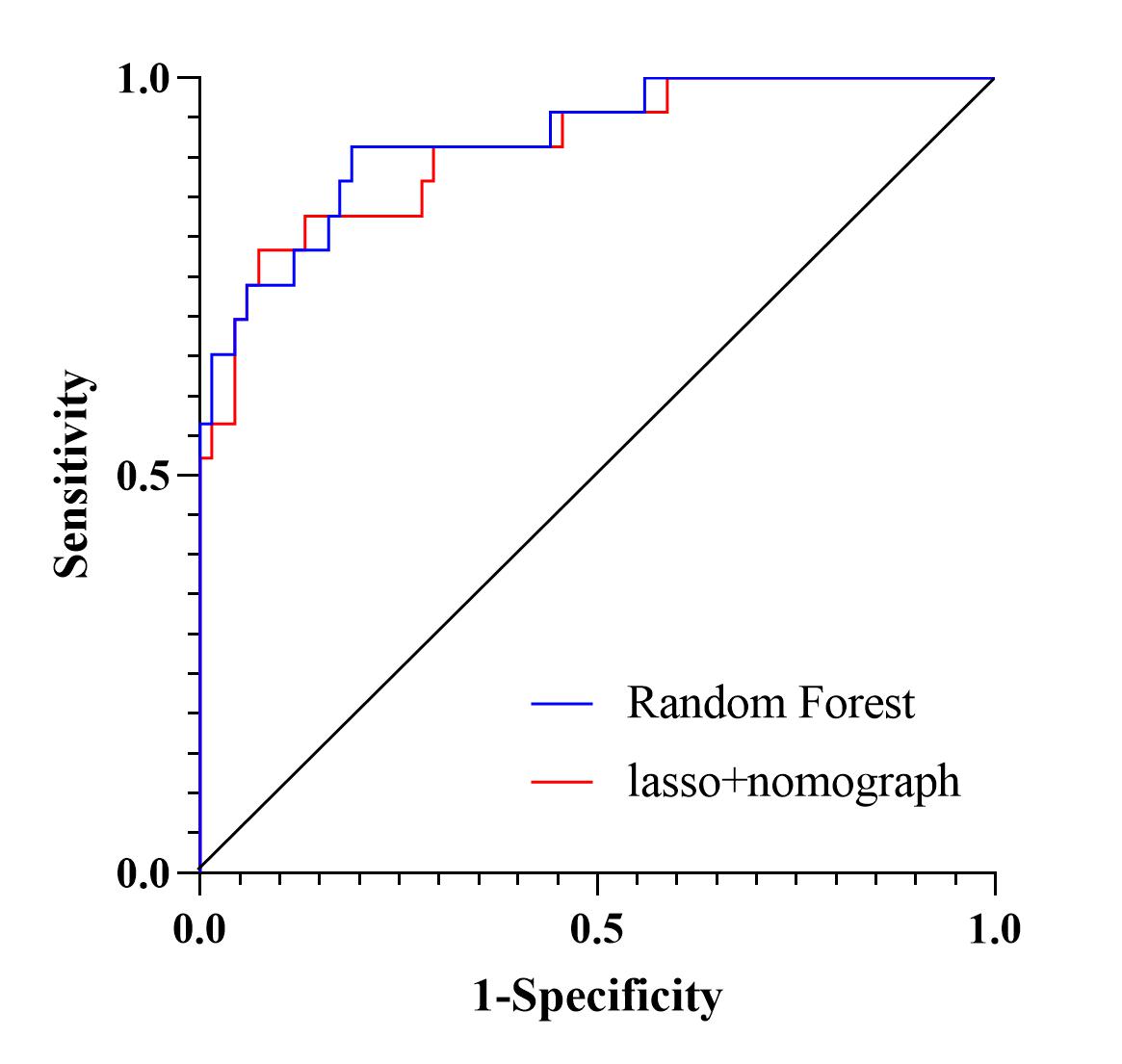

Supplement: Supplementary file 22 — Supplementary Material 22 [file 12876_2025_4198_MOESM22_ESM.jpg]

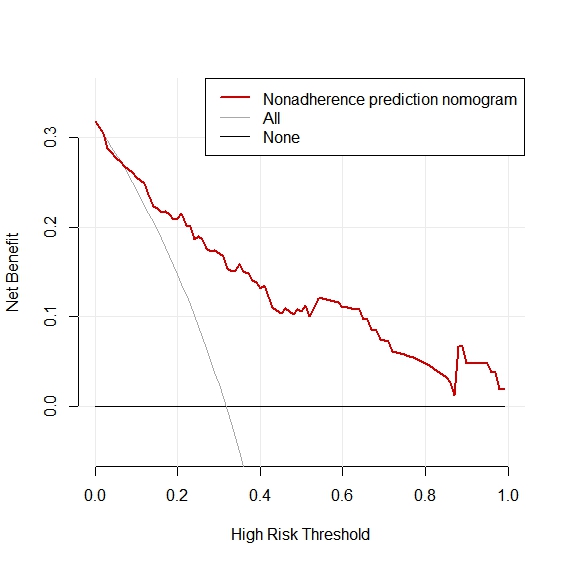

Supplement: Supplementary file 23 — Supplementary Material 23 [file 12876_2025_4198_MOESM23_ESM.jpg]

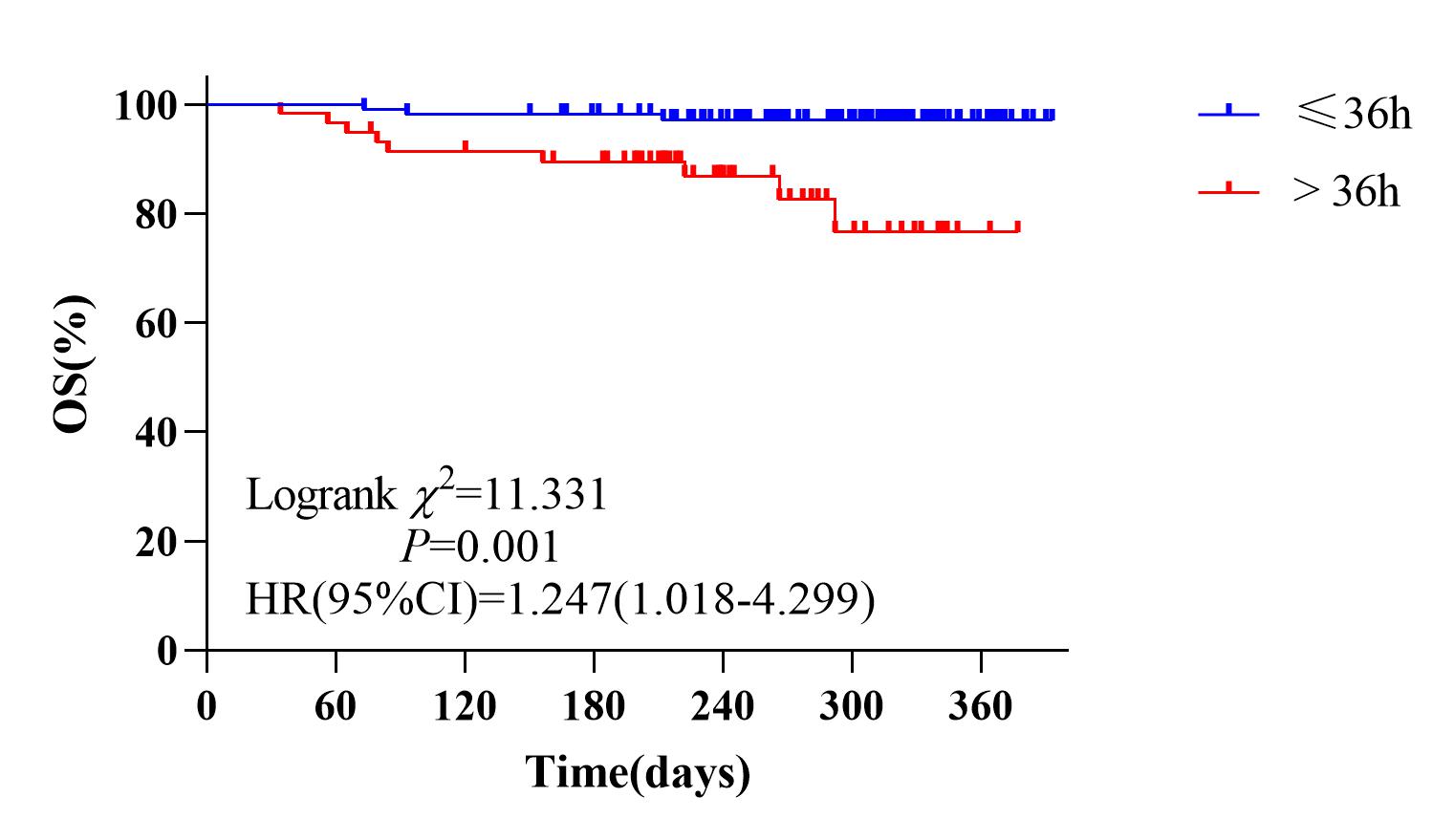

Supplement: Supplementary file 24 — Supplementary Material 24 [file 12876_2025_4198_MOESM24_ESM.jpg]

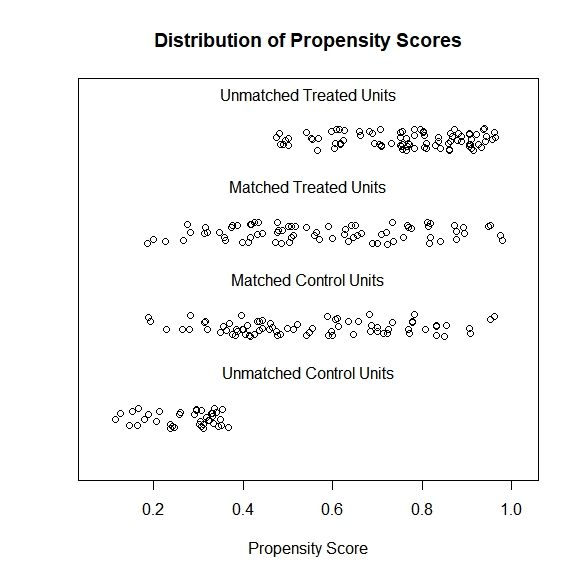

Supplement: Supplementary file 25 — Supplementary Material 25 [file 12876_2025_4198_MOESM25_ESM.jpg]

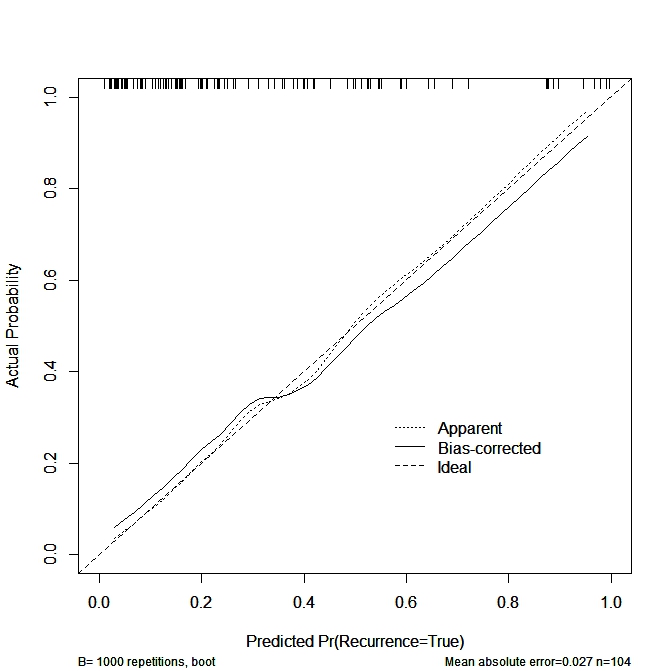

Supplement: Supplementary file 26 — Supplementary Material 26 [file 12876_2025_4198_MOESM26_ESM.jpg]

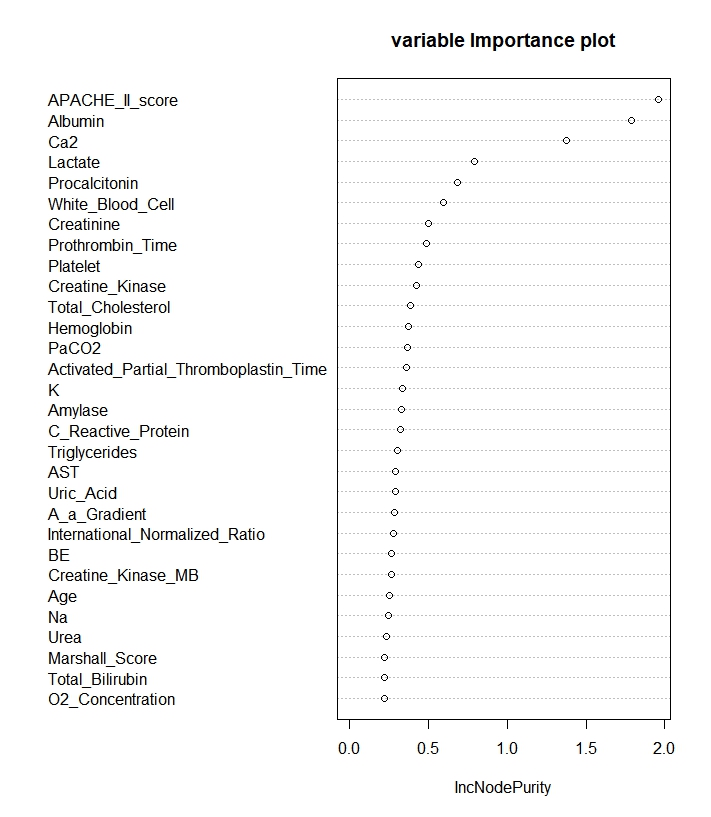

Supplement: Supplementary file 27 — Supplementary Material 27 [file 12876_2025_4198_MOESM27_ESM.jpg]
