# Supplementary material for: Application of continuous renal replacement therapy (CRRT) in patients with severe acute pancreatitis: an analytical study
Source: BMC Gastroenterol. 2025 Aug 18;25:592. doi: 10.1186/s12876-025-04198-y (PMC12359950; doi:10.1186/s12876-025-04198-y)
Supplement: Supplementary file 10 — Supplementary Material 10 [file 12876_2025_4198_MOESM10_ESM.docx]

| Outcome Measure​ | Group​ | n（%） |
| --- | --- | --- |
| 90-day Survival​ | Alive | 180(100) |
|  | Deceased | 0(0) |
| ​​1-year Survival | Alive | 180(100) |
|  | Deceased | 0(0) |
| ​​1-year Readmission | No | 177(98.33) |
|  | Yes | 3(1.67) |
| ​​Renal Function Impairment​ | No | 180(100) |
|  | No | 0(0) |
