# Supplementary material for: Application of continuous renal replacement therapy (CRRT) in patients with severe acute pancreatitis: an analytical study
Source: BMC Gastroenterol. 2025 Aug 18;25:592. doi: 10.1186/s12876-025-04198-y (PMC12359950; doi:10.1186/s12876-025-04198-y)
Supplement: Supplementary file 12 — Supplementary Material 12 [file 12876_2025_4198_MOESM12_ESM.docx]

| ​Model​ | AUC | 95%CI | z | P |
| --- | --- | --- | --- | --- |
| Random Forest Model | 0.923 | 0.858-0.987 | 1.135 | 0.257 |
| Lasso + Nomogram Model | 0.912 | 0.841-0.982 |  |  |
