# Supplementary material for: Application of continuous renal replacement therapy (CRRT) in patients with severe acute pancreatitis: an analytical study
Source: BMC Gastroenterol. 2025 Aug 18;25:592. doi: 10.1186/s12876-025-04198-y (PMC12359950; doi:10.1186/s12876-025-04198-y)
Supplement: Supplementary file 13 — Supplementary Material 13 [file 12876_2025_4198_MOESM13_ESM.docx]

| **Variable​** | **​​Missing Rate**（%） | **Before Imputation​** | | **After Imputation​** | | **MCAR Test p-value​** |
| --- | --- | --- | --- | --- | --- | --- |
|  |  | OR(95%CI) | P | OR(95%CI) | P |  |
| Urinary amylase | 86.06 | 1.000(1.000-1.000) | 0.942 | 1.000(1.000-1.000) | 0.947 | 0.086 |
| HBP | 73.94 | 1.003(0.994-1.011) | 0.531 | 1.002(0.995-1.010) | 0.549 | 0.116 |
| Troponin T | 88.48 | 0.578(0.001-266.65) | 0.861 | 0.599(0.002-213.776) | 0.864 | 0.204 |
| Troponin I | 61.82 | 1.008(0.938-1.083) | 0.835 | 1.008(0.937-1.085) | 0.827 | 0.175 |
| BNP | 70.91 | 1.000(1.000-1.000) | 0.953 | 1.000(1.000-1.000) | 0.950 | 0.093 |
| pro-BNP | 85.45 | 1.000(1.000-1.000) | 0.162 | 1.000(1.000-1.000) | 0.185 | 0.063 |
