# Supplementary material for: Application of continuous renal replacement therapy (CRRT) in patients with severe acute pancreatitis: an analytical study
Source: BMC Gastroenterol. 2025 Aug 18;25:592. doi: 10.1186/s12876-025-04198-y (PMC12359950; doi:10.1186/s12876-025-04198-y)
Supplement: Supplementary file 14 — Supplementary Material 14 [file 12876_2025_4198_MOESM14_ESM.docx]

| Etiology​​ | n | OR(95%CI) | P |
| --- | --- | --- | --- |
| Biliary | 30 | 0.429(0.110-0.748) | <0.001 |
| Hyperlipidemic | 41 | 0.139(0.013-0.265) | <0.001 |
| Alcoholic | 10 | 0.521(0.206-0.836) | <0.001 |
| Other | 10 | 0.564(0.219-0.909) | <0.001 |

The P-value of the interaction is 0.027.
