# Supplementary material for: Application of continuous renal replacement therapy (CRRT) in patients with severe acute pancreatitis: an analytical study
Source: BMC Gastroenterol. 2025 Aug 18;25:592. doi: 10.1186/s12876-025-04198-y (PMC12359950; doi:10.1186/s12876-025-04198-y)
Supplement: Supplementary file 16 — Supplementary Material 16 [file 12876_2025_4198_MOESM16_ESM.docx]

| Variable | Before Matching | | | After Matching | | |
| --- | --- | --- | --- | --- | --- | --- |
|  | Treated | Control | SMD | Treated | Control | SMD |
| Age (years) | 49.35 | 63.23 | -0.76 | 56.87 | 57.19 | -0.02 |
| Gender | 1.28 | 1.35 | -0.14 | 1.30 | 1.32 | -0.06 |
| Pancreatitis Type | 2.05 | 2.06 | -0.01 | 2.09 | 2.22 | -0.13 |
| APACHEII Score | 14.9 | 12.90 | 0.27 | 14.90 | 13.66 | 0.06 |
| Marshall Score | 4.13 | 3.27 | 0.46 | 3.92 | 3.60 | 0.07 |
| Medical History | 0.61 | 0.48 | 0.26 | 0.57 | 0.55 | 0.05 |
