# Supplementary material for: Application of continuous renal replacement therapy (CRRT) in patients with severe acute pancreatitis: an analytical study
Source: BMC Gastroenterol. 2025 Aug 18;25:592. doi: 10.1186/s12876-025-04198-y (PMC12359950; doi:10.1186/s12876-025-04198-y)
Supplement: Supplementary file 17 — Supplementary Material 17 [file 12876_2025_4198_MOESM17_ESM.docx]

| Parameter | Improved | Poor | T | p |
| --- | --- | --- | --- | --- |
| Total CRRT sessions | 3.08±0.99 | 2.74±0.90 | 0.499 | 0.618 |
| Mean session duration (h) | 14.23±2.11 | 13.74±1.96 | 1.413 | 0.159 |
| Clotting events | 0.96±0.54 | 1.02±0.51 | -0.707 | 0.480 |
| Peak TMP (mmHg) | 188.5±14.71 | 191.26±17.8 | -1.039 | 0.300 |
| LMWH dosage (ml/kg) | 0.41±0.11 | 0.39±0.09 | 1.016 | 0.311 |
| Mean MAP (mmHg) | 74.90±10.41 | 76.66±8.62 | -1.052 | 0.294 |
| Fluid balance (ml) | 549.57±1113.82 | 500.00±1028.97 | 0.269 | 0.789 |
