# Supplementary material for: Application of continuous renal replacement therapy (CRRT) in patients with severe acute pancreatitis: an analytical study
Source: BMC Gastroenterol. 2025 Aug 18;25:592. doi: 10.1186/s12876-025-04198-y (PMC12359950; doi:10.1186/s12876-025-04198-y)
Supplement: Supplementary file 18 — Supplementary Material 18 [file 12876_2025_4198_MOESM18_ESM.docx]

| ​Variable​ | Improved​ | ​​Poor | X^2^/t | P |
| --- | --- | --- | --- | --- |
| 72h Fluid Balance (mL)​ | 1533.33±300.09 | 1477.45±324.85 | 1.458 | 0.146 |
| ​​Antibiotic Use (%)​ |  |  | 0.044 | 0.833 |
| No | 150(83.33) | 84(82.35) |  |  |
| Yes | 30(16.67) | 18(17.65) |  |  |
| Colloid Use (%)​ |  |  | 0.829 | 0.363 |
| No | 94(52.22) | 59(57.84) |  |  |
| Yes | 86(47.78) | 43(42.16) |  |  |
| ​Vasopressor Use (%)​ |  |  | 1.645 | 0.200 |
| No | 83(46.11) | 39(38.24) |  |  |
| Yes | 97(53.89) | 63(61.76) |  |  |
