# Supplementary material for: Application of continuous renal replacement therapy (CRRT) in patients with severe acute pancreatitis: an analytical study
Source: BMC Gastroenterol. 2025 Aug 18;25:592. doi: 10.1186/s12876-025-04198-y (PMC12359950; doi:10.1186/s12876-025-04198-y)
Supplement: Supplementary file 19 — Supplementary Material 19 [file 12876_2025_4198_MOESM19_ESM.docx]

|  | B | S.E. | Wald | P | OR | 95%CI | |
| --- | --- | --- | --- | --- | --- | --- | --- |
|  |  |  |  |  |  | Lower | Upper |
| ​​Pancreatitis Type​​ |  |  |  |  |  |  |  |
| Biliary |  |  |  |  | 1.000 |  |  |
| Hyperlipidemic | -0.916 | 0.922 | 0.988 | 0.32 | 0.400 | 0.066 | 2.437 |
| Alcoholic | -1.099 | 1.08 | 1.035 | 0.309 | 0.333 | 0.040 | 2.769 |
| Other | -1.386 | 1.323 | 1.098 | 0.295 | 0.250 | 0.019 | 3.342 |
| APACHEII Score | 0.387 | 0.143 | 7.343 | 0.007 | 1.472 | 1.113 | 1.947 |
| Marshall Score | 0.32 | 0.44 | 0.529 | 0.467 | 1.377 | 0.581 | 3.262 |
| Lactate | 1.753 | 0.788 | 4.953 | 0.026 | 5.773 | 1.233 | 27.036 |
| Calcium | -3.829 | 1.714 | 4.99 | 0.025 | 0.022 | 0.001 | 0.625 |
| Albumin | -0.104 | 0.047 | 4.947 | 0.026 | 0.901 | 0.822 | 0.988 |
| PT | 0.179 | 0.144 | 1.558 | 0.212 | 1.196 | 0.903 | 1.585 |
| PCT | 0.434 | 0.219 | 3.911 | 0.048 | 1.543 | 1.004 | 2.372 |
